# Potential Multiaxial Molecular Ferroelectricity through Chiral Cation Replacement

**DOI:** 10.1021/acs.cgd.5c00666

**Published:** 2025-07-21

**Authors:** Sam Y. Thompson, Rebecca H. Abeyasekere, Samuel J. Page, Paul Hodgkinson, Cameron A. M. Scott, Nicholas C. Bristowe, Oliver J. Wagstaff, John S. O. Evans

**Affiliations:** † Department of Chemistry, 3057 Durham University, Lower Mount Joy, South Road, Durham DH1 3LE, U.K.; ‡ Centre for Materials Physics, 3057 Durham University, Durham DH1 3LE, U.K.

## Abstract

Molecular ferroelectrics are an important class of materials offering chemical versatility, low toxicity, and tunable functional properties. A major design challenge lies in achieving multiaxial properties akin to inorganic perovskite ferroelectrics. Here, we report a series of new potential multiaxial molecular ferroelectrics obtained by introducing chiral cations into a structure type known to undergo a phase transition that raises the symmetry significantly. Three of the compounds studied show an Aizu *m*3̅*mFm* phase transition, resulting in 24 equiv polarization directions in the polar phase. ^1^H solid-state NMR was used to study the dynamics of the organic cation, confirming rapid rotation about the 3-fold rotation axis of the cubic cell. This blurs the chiral center to an X-ray probe, making the distinction between Sohncke and non-Sohncke space group choices redundant.

## Graphical abstract

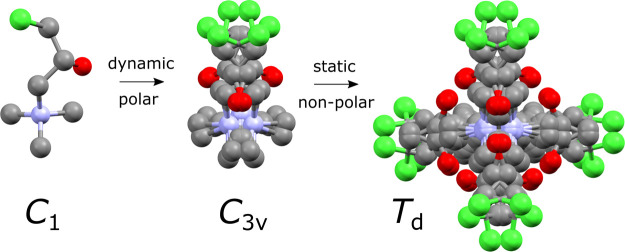

## Introduction

Ferroelectric materials, those which show switchable spontaneous polarization upon application of an external electric field, are essential in a wide range of applications including sensors, actuators, memory devices, and energy harvesting systems.^1,2^ In recent years, molecular ferroelectrics have emerged as a promising class of materials due to their structural diversity, low toxicity, and the potential for low-temperature processing for thin film applications.^3–5^ Unlike conventional inorganic ferroelectrics (e.g., barium titanate^6^ and lead zirconate titanate^7^), molecular ferroelectrics offer tunable behavior through molecular design strategies such as H/F^8–10^ and chiral substitution.^8,11^

Chiral substitution is important because introducing chiral molecules into otherwise centrosymmetric structures breaks inversion and other symmetries, increasing the likelihood of the structure adopting a polar space group. More formally, chiral compounds can only crystallize in Sohncke space groups (i.e., those which only contain rotational and translational symmetry).^12^ A high proportion of these are polar, meaning that chiral species have an increased probability of ferroelectricity.

Previously, a group of metal halide anion salts of racemic (*Rac*-CTA)_2_MCl_4_ have been shown to undergo dielectric switching (CTA = 3-chloro-2-hydroxypropyltrimethylammonium). These are hereon abbreviated as *Rac* **-M** and are known for M = Mn,^13^ Co,^14^ Cd,^13^ Zn,^15^ and Cu^16^). These compounds have been reported to crystallize in the nonpolar space group *C*2/*c* (structure type A) at room temperature, and all show a symmetry-raising phase transition in the temperature range 360–420 K.

Chiral replacement was used by Xiong et al. to produce enantiomerically pure (*R*-CTA)_2_CuCl_4_ (***R*-** **Cu**) and (*S*-CTA)_2_CuCl_4_ (***S*-** **Cu**),^16^ which crystallize in space groups *P*3_1_21 and *P*3_2_21, respectively. They were reported to be thermochromic and ferroelastic. Upon warming, both showed phase transitions associated with an increase in symmetry from trigonal to cubic. The structure of the high-temperature form has not been solved, to our knowledge.

In this article, we report the effect of introducing the chiral cation (*S*-CTA^+^) into the other known metal halide racemic structures.^8,11,17,18^ Four new crystal structures were found and solved using single-crystal X-ray diffraction (SXRD), two of which have potentially ferroelectric space groups. The structural family was also extended to the chiral nickel analogue *S* **-Ni**, which also crystallizes in a polar space group.

Contrary to previous claims in the literature, we found that the high-temperature structure is common to all members of this family. A cubic structure (*Fm*3̅*m*, *a* = ∼13.5 Å) was solved from powder X-ray data and can be related to all other known structures through group-subgroup relationships. The high level of dynamic disorder in this phase, which we studied with ^1^H solid-state NMR, nullifies the chirality of the *S*-CTA^+^ cation, allowing adoption of the non-Sohncke space group. The significant symmetry change from cubic to monoclinic on cooling makes these materials, like the inorganic perovskites, good candidates for multiaxial ferroelectricity.

## Experimental Section

### Synthesis

CTA-Cl (2.50 cm^3^, 1.73 g, 5.0 mmol) and MCl_2_ (∼0.3 g, 2.5 mmol) were dissolved in deionized water (5 cm^3^) and combined under stirring. Upon standing, the initially clear solution became slightly cloudy. A small amount of HCl (5 cm^3^) was added to dissolve the precipitate, after which the solution was stirred for 10 min. Allowing the solvent to evaporate over a two-week period produced plate-like single crystals. All samples were produced by this method using the respective metal chloride. Reagent details along with the resulting crystal color can be found in Table 1.

**Table 1:** Reagents Used during Synthesis and Crystal Colors

reagent	supplier	purity	crystal color
CuCl_2_	Merck Life Science	99%	yellow-green
CdCl_2_	Merck Life Science	99.99%	colorless
CoCl_2_·6H_2_O	Sigma-Aldrich	reagent grade	dark blue
MnCl_2_·4H_2_O	Sigma-Aldrich	≥98%	yellow
ZnCl_2_	Fisher Scientific	98+%	colorless
NiCl_2_	Sigma-Aldrich	98%	colorless
*Rac*-CTA-Cl	Merck Life Science	63 ± 5 wt % in H_2_O	colorless
*S*-CTA-Cl	Merck Life Science	99%	colorless
hydrochloric acid (12M)	Fisher Scientific	extra pure	n/a

### *S*-CTA Conformation Search

An initial CONFLEX conformation search of CTA^+^ was performed using the MM3 force field^19^ inside the Scigress software.^20^ To validate the energetic ordering of the found conformers, geometry optimizations were carried out using density functional theory (DFT) with the B3LYP functional^21,22^ and the Grimme D3 empirical dispersion correction with Becke–Johnson damping (B3LYP-D3BJ),^23^ as implemented in Gaussian 16.^24^ The 6–31G­(d) basis set^25^ was used for all atoms. Each structure was confirmed as a minimum via vibrational frequency calculations (no imaginary frequencies). Gibbs free energies were obtained at 298.15 K by including zero point energy and thermal contributions from vibrational, rotational, and translational motion, based on the harmonic oscillator and rigid rotor approximations. The DFT energies are consistent with the ordering calculated by using the MM3 force field. Both are reported relative to the lowest energy conformer.

### Powder X-ray Diffraction (PXRD)

Variable temperature PXRD data were collected using a Bruker D8 ADVANCE diffractometer (Mo Kα radiation and LYNXEYE detector) equipped with an Oxford Cryosystems Cryostream Plus device. The samples were loaded into 0.7 mm external diameter borosilicate capillaries to a length of 30 mm. The capillaries were sealed and attached to a goniometer, which rotated at 10 rotations a minute during the measurements. Diffraction patterns were recorded between 3° and 30° 2θ. Data were analyzed with TOPAS-Academic.^26–28^

### Single Crystal X-ray Diffraction (SXRD)

SXRD data were collected by using a Bruker D8 VENTURE diffractometer (PHOTON III C7MM CPAD detector, ImS-microsource, focusing mirrors) equipped with an Oxford Cryosystems Cryostream 700+ device using Mo Kα radiation. Crystal structures were solved within the Olex2 software package.^29^ H atoms were placed in calculated positions and refined in the riding mode.

### NMR

NMR data were collected on a Bruker Avance III HD spectrometer at 400.17 MHz using a static (wide-line) NMR probe. ∼1 g of dried *S* **-Cd** was packed into a 5 mm glass holder. The sample was measured at 10 K intervals between 183 and 393 K. At each temperature, static solid-state ^1^H spectra were obtained using solid echo (SE) with a 50 μs interpulse echo delay followed by relaxation measurements.

Scripts from the dipolar_averages GitHub repository^30^ were used to analyze the second moment, *M*_2_, values of the ^1^H spectra. Direct integration of the ^1^H lineshapes (to 99% convergence of the area integral used for normalization) was used to evaluate the experimental *M*_2_:

$$M_{2}=\frac{\int_{-\infty }^{\infty }S(v)(v-v_{0}{)}^{2}dv}{\int_{-\infty }^{\infty }S(v)dv}$$ 1

where *v*_0_ is the center of the ^1^H line shape. The expected *C*_3_ rotation axis of the cubic cell was transformed to the monoclinic cell setting. Two boron dummy atoms were added to the monoclinic structure on this line. *M*_2_ values were calculated for the rotational averaging of the CTA^+^ molecule about the line defined by these dummy atoms (dynamic limit) and for methyl-only rotation (static limit).

*T*_1_ relaxation times were measured using proton-detected saturation recovery with a solid echo. *T*_1ρ_ relaxation times were measured by using a solid echo sequence with a variable length 50 kHz spin lock pulse. A 50 μs interpulse echo delay was used for *T*_1_ and *T*_1ρ_ measurements. For both sets of relaxation measurements, data were extracted using the Python module, nmrglue,^31^ before integration in a Python script to determine the *T*_1_ and *T*_1ρ_ relaxation times using a stretched exponential and exponential, respectively (fitting parameters are reported in the Supporting Information). An Arrhenius-type dependence of the motional correlation time on temperature was assumed:

$$\tau_{c}=\tau_{\infty }\exp\left(E_{a}/RT\right)$$ 2

### Computational Polarization Calculations

Polarization calculations were performed using density-functional theory (DFT) in the CASTEP software,^32^ which uses the Berry phase formulation^33^ to calculate the electronic contribution to the polarization. The Perdew–Burke–Ernzerhof exchange correlation functional (PBE)^34^ was used as well as the Manybody Dispersion Correction scheme (MBD)^35^ to correct for long-range dispersion effects. Theoretical nonpolar aristotypes of the polar phases of *S* **-Cd** and *S* **-Zn** were produced using FINDSYM.^36^ A CIF “movie” was generated in ISODISTORT^37^ with 11 frames and a linear change in order parameter across a half-period of the transition. This generated a series of structures between the nonpolar and polar forms. Cell files were generated with a *k*-point grid of 3 × 3 × 2 and an offset of ( 14 , 14 , 14 ). Calculations were performed with a plane wave cutoff to 1000 eV and to a convergence of ∼0.01 eV/atom. Calculated polarization values were plotted modulo the quantum of polarization (*Q*_*i*_), which was calculated using

$$Q_{i}=\frac{eR_{i}}{V}$$

where *e* is the elementary charge, *R*_*i*_ is a lattice vector, and *V* is the volume of the unit cell.

## Results and Discussion

### *S*-CTA^+^ Conformers

The three lowest energy conformations of CTA^+^ are shown in Scheme 1. These generally arrange as linearly as possible, with the key difference being rotation about the terminal C–C bond. The energetic ordering of the three conformations can be rationalized by steric considerations: conformer 1 positions the chlorine anti with respect to the hydroxyl group, with conformers 2 and 3 having a gauche relationship. Conformer 2 is next lowest in energy due to the anti relationship between the chlorine and R-groups as compared with the gauche configuration in conformer 3.

**Scheme 1:**
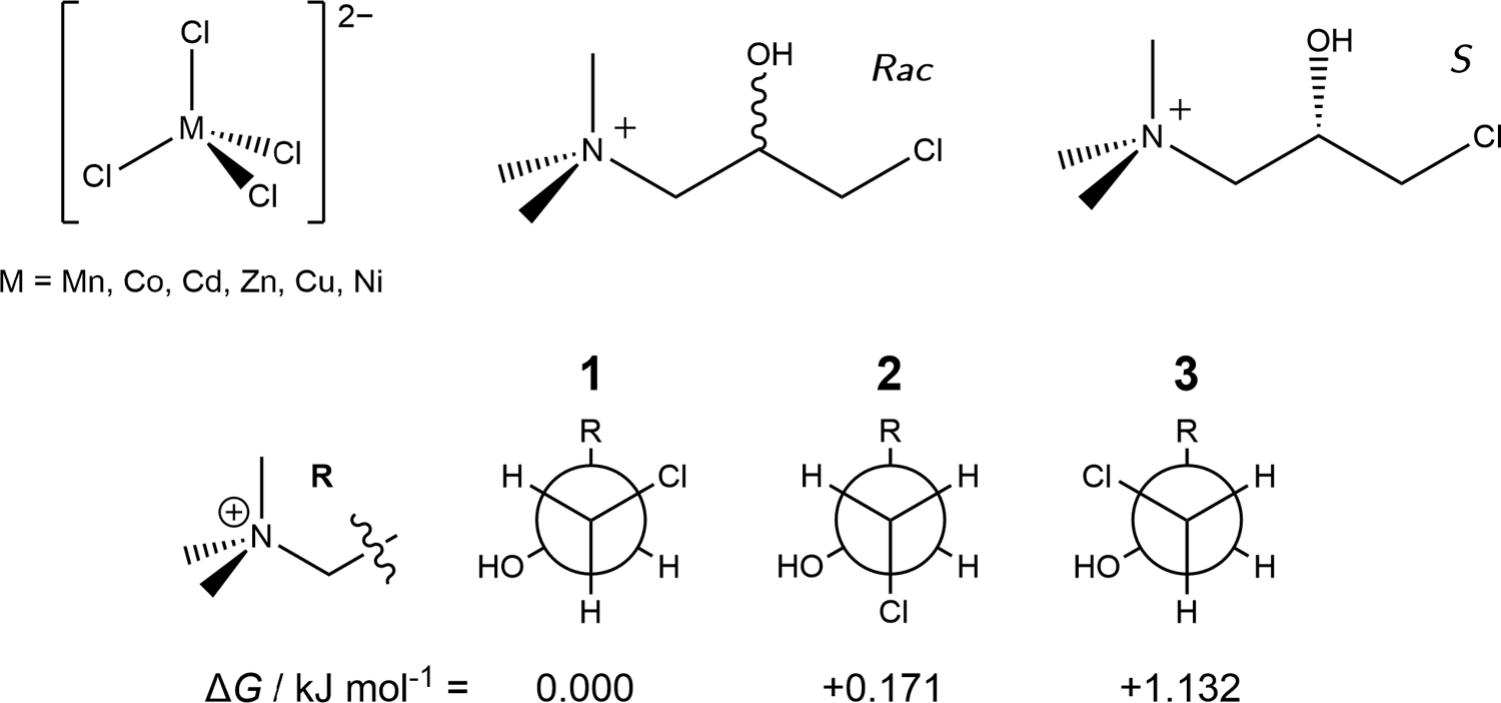
Molecular Structures of *Rac*-**M** and *S*-**M** ^a^

a All discussed compounds crystallize as a 2:1 ratio of CTA^+^ to metal halide anion. The three lowest energy conformations of CTA^+^ are shown.

### Racemic Crystal Structures

The crystal structures of the racemic compounds (***Rac*-** **M**) have been reported in the literature for M = Mn^13^, Co^14^, Cu^16^, Zn^15^, and Cd^13^; the Ni member is not reported. All the racemic compounds crystallize in the centrosymmetric space group *C*2/*c* with two distinct pseudocubic structure types we label A and A′. Rietveld refinements were performed using the reported structures. No major discrepancies were noted between the experimental and calculated patterns, confirming successful synthesis of pure bulk samples and corroborating the literature structures.

Structure types A – *Rac* **-Mn**, *Rac* **-Co**, *Rac-* **Zn**, and *Rac-* **Cd** crystallize in centrosymmetric space group *C*2/*c* (Figure 1). The slightly distorted MCl_4_^2–^ tetrahedra lie on the 2-fold rotation axis of the unit cell. The organic cation sits a general position, and due to its racemic nature, the hydroxy group has two equally probable locations. The CTA^+^ cation adopts solely conformer 2. This and other structures are pseudocubic as discussed later in Figure 5.

**Figure 1:**
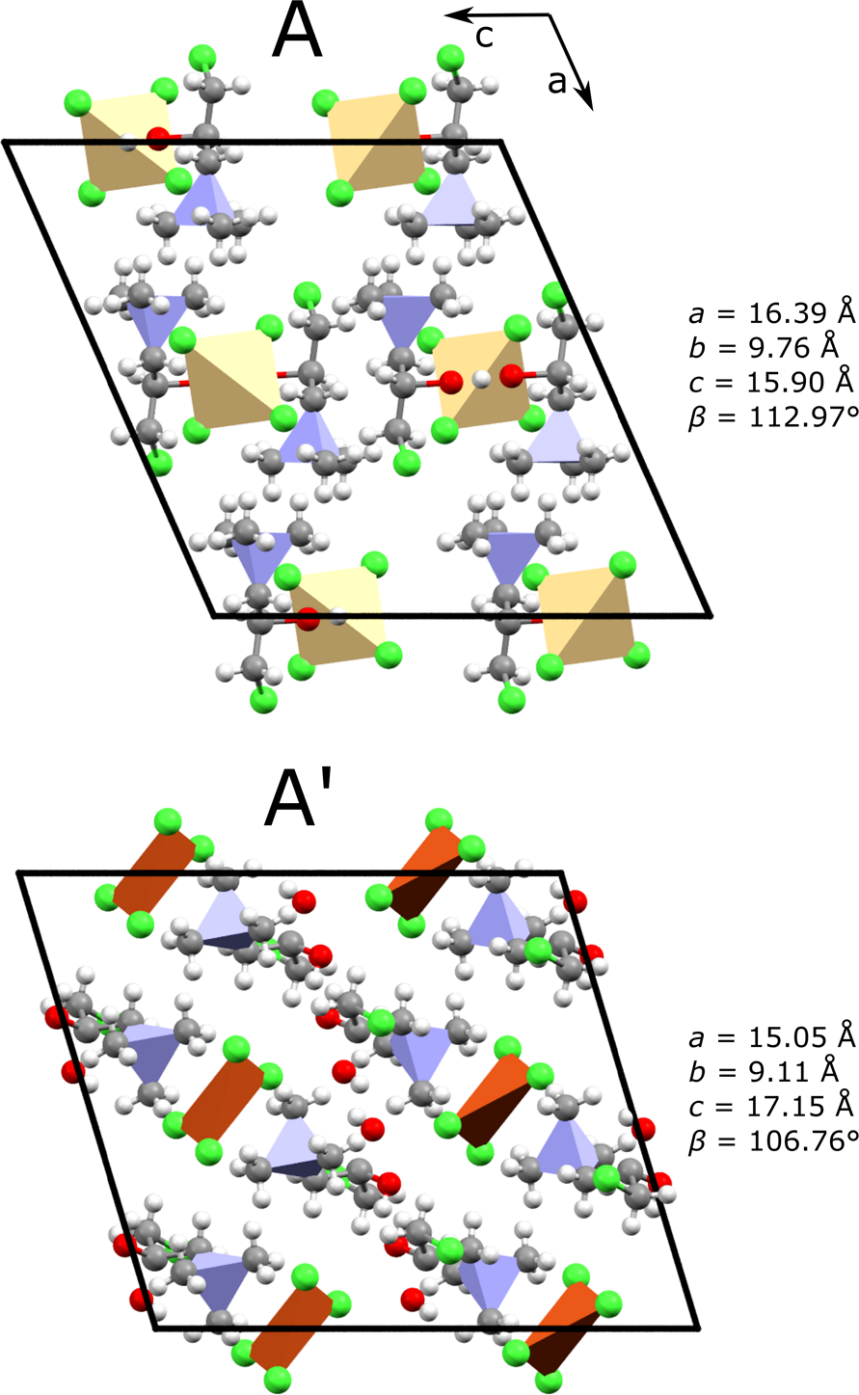
Structure types A and A′ viewed along the *b*-axis. CuCl_4_^2–^ tetrahedra (orange-brown) are significantly more distorted than other MCl_4_^2–^ tetrahedra (yellow). Colored spheres throughout this publication show carbon (gray), nitrogen (light blue), hydrogen (white), chlorine (green), oxygen (red), copper (orange-brown), cadmium (yellow), manganese (purple), and nickel (dark green) atoms.

Structure type A′ *– Rac* **-Cu** also crystallizes in *C*2/*c* with similar cell parameters to structure type A (Figure 1). Although the distribution of metal tetrahedra and the ammonium end of CTA^+^ is the same in both structures, there are several differences: the CTA^+^ molecule is rotated such that the propyl group points in a different direction and the CuCl_4_^2–^ tetrahedra are significantly distorted (bond angles range from 94.25(4) to 145.31(3)°). In addition, the CTA^+^ molecules adopt a 1:1 ratio of conformers 1 and 3, whereas in structure type A, the CTA^+^ molecules all adopt conformer 2. The average energy of conformers 1 and 3 (+0.566 kJ mol^–1^) is marginally higher than the energy of conformer 2, suggesting that the unfavorable CTA^+^ conformation arrangement allows for an energy-lowering distortion of the CuCl_4_^2–^ tetrahedra.

### Enantiomorphic Crystal Structures

The homochiral members of this family also crystallize with pseudocubic structures. The *S*-enantiomer was used in all of the syntheses. Depending on the metal used, different distortions from the pseudocubic structure result in three different room temperature structures, which we label C, D, and E. All three were solved from SXRD data. The differences between these structures are summarized in Table 2. O–H···Cl hydrogen bonds stabilize each structure. The CTA^+^ molecules sit in general positions, and the MCl_4_^2–^ lie on 2-fold rotation axes in each structure.

**Table 2:** Summary of Room Temperature Structures A to E and Structure F Reported at 437 K^a^

structure type	compound	space group	cell parameters/Å	volume per *Z*/Å^3^	O–H···Cl distance/Å	*Z*, *Z*′	CTA^+^ conformers
A	*Rac*-Mn, Co, Zn, Cd	*C*2/*c*	*a* = 16.39	281.1	2.311	8, 1	2
			*b* = 9.76				
			*c* = 15.90				
			β = 112.97°				
A′	*Rac*-Cu	*C*2/*c*	*a* = 15.05	281.4	2.283	8, 1	1,3
			*b* = 9.11				
			*c* = 17.15				
			β = 106.76°				
B	*R, S*-Cu	*P*3_1_21, *P*3_2_21	*a* = 8.95	279.7	2.525	6, 1	1
			*c* = 24.22				
C	*S*-Mn, Co	*P*3_2_21	*a* = 9.48	293.2	2.217	18, 3	2,2,2
			*c* = 67.81		2.412		
					2.432		
D	*S*-Cd	*P*2	*a* = 10.00	294.0	2.330	4, 2	1,3
			*b* = 9.39				
			*c* = 12.60		2.439		
			β = 96.24°				
E	*S*-Zn, Ni	*C*2	*a* = 17.14	288.6	2.361	8, 2	1,3
			*b* = 9.13				
			*c* = 15.10		2.537		
			β = 102.23°				
F	*Rac*, *S*-M	*Fm*3̅*m*	*a* = 13.56	316.9		8, 1	n/a

a Multiple metals exhibit the same structure type, values are quoted for the first listed (*Rac*-Cd for structure F). Data for structure-type A were taken from reference 13. Data for structures A′ and B taken from reference 16.

Structure type B – The only previously reported homochiral members of this family are (*R*-(CTA)_2_CuCl_4_) and (*S*-(CTA)_2_CuCl_4_), which crystallize in space groups *P*3_1_21 and *P*3_2_21, respectively. A Rietveld refinement performed using this model confirmed the literature crystal structure.^16^ As with the racemic A′ structure, CuCl_4_^2–^ anions show significant distortions (angles 93.51(3)° to 145.28(3)°). The single unique CTA^+^ molecule adopts the lowest-energy conformer 1.

Structure types C – *S* **-Co** and *S* **-Mn** crystallize in space group *P*3_2_21 at room temperature. Their cells are similar to those of *S* **-Cu** but with a tripled *c*-axis. One of the three CTA^+^ molecules in the unit cell shows disorder of the propyl group of the quaternary ammonium ion across two sites in a 70:30 ratio. All three CTA^+^ molecules in the asymmetric unit adopt conformer 2.

Structure type D – *S* **-Cd** crystallizes in space group *P*2 at room temperature with half the CTA^+^ molecules adopting conformer 1 and half conformer 3. Polarization is present along the *b*-axis. MCl_4_^2–^ groups lie on the 2-fold axis.

Structure types E – *S* **-Zn** and *S* **-Ni** both crystallize in space group *C*2. The molecular arrangement is equivalent to structure-type A, but the enantiopure *S*-CTA^+^ molecule breaks the inversion center and glide plane, and thus the disorder in the OH group is no longer seen. This produces polarization along the *b*-axis. As in structure-type A, the CTA^+^ molecules adopt a 1:1 ratio of conformers 1 and 3.

### Structural Phase Transitions

Variable temperature (VT) PXRD data were collected to confirm the temperature-dependent polymorphism of *S* **-Cu** reported by Xiong et al.,^16^ to investigate the structure of the unknown high-temperature phase, and to investigate the temperature-dependent polymorphism of the new members of this family.

VT-PXRD data were collected for *S* **-Cu** between 290 and 430 K on warming and cooling with a heating rate of 16 K/h and are shown in Figure 2. The abrupt changes observed in the Bragg peaks indicate a discontinuous phase transition upon warming at *T*_c,warm_ ≈ 417 K. We label this new structure type F. On cooling, the transition (F → B) is reversible and shows a significant thermal hysteresis (*T*_c,cool_ ≈ 359 K: Δ ≈ 58 K), which is consistent with the first-order nature of this transition. Xiong et al. reported the transition to occur between 393 and 423 K on warming. The high-temperature phase F has higher symmetry than the room-temperature phase B, evidenced by the significantly lower number of Bragg peaks in a given 2θ range.

**Figure 2:**
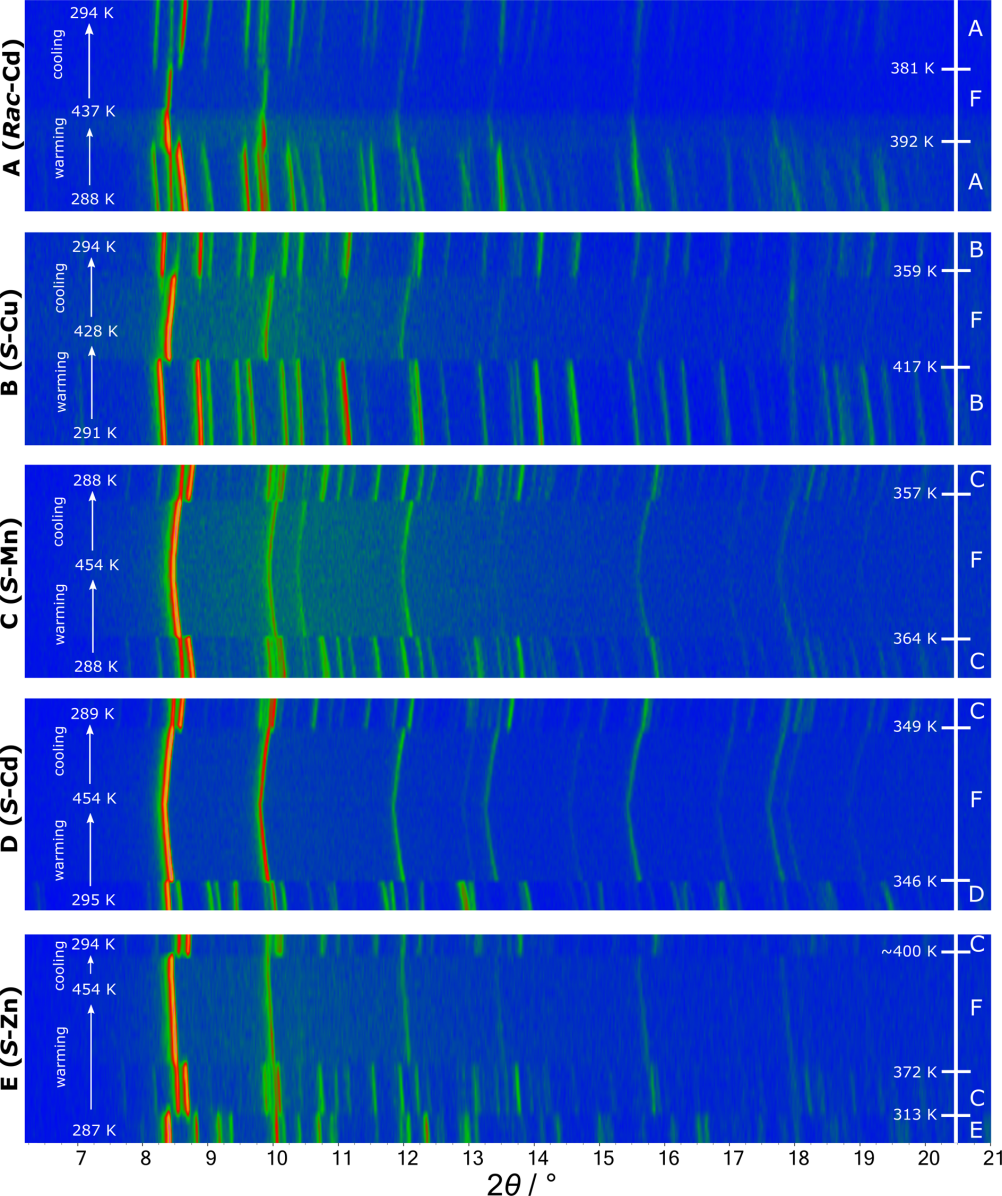
Surface plots of variable temperature PXRD data on warming and cooling different samples between room temperature and ∼450 K. An artificial color map represents the intensity: blue for low intensity and orange for high intensity.

One sample representing each of the three new structure types was selected for VT analysis (C: *S-* **Mn**, D: *S* **-Cd**, E: *S* **-Zn**). VT-PXRD data were collected on each between ∼290 and ∼450 K on warming and cooling with heating rates of 18 K/h (heating and cooling) for *S-* **Mn**/*S-* **Cd** and 19 K/h (heating) and 85 K/h (cooling) for *S* **-Zn.**

Phase sequences and temperatures of transitions are summarized in Figure S2. All samples ultimately transition to structure type F upon heating. *S* **-Zn** (structure E) goes through structure type C in an intermediate step. Upon cooling, all three samples transition to structure type C. Two weeks after the VT-PXRD experiment, a PXRD pattern showed that *S* **-Cd** had reverted from structure C back to its original structure D. This suggests that phase C is a kinetic polymorph formed upon cooling, whereas polymorphs D and E are thermodynamically more stable and form directly during the slow evaporation of an aqueous solution.

VT-PXRD was also collected for *Rac* **-Cd** so that high-temperature structure type F could be compared between the racemic and enantiopure compounds. Again, at high temperature, structure F is observed with phase transitions occurring at *T*_c,warm_ = 392 K and *T*_c,cool_ = 381 K. The high temperature pattern shows no significant differences from those of the other compounds, suggesting a shared high temperature structure across the family.

### Structure Solution of the High-Temperature Structure F

The large discontinuous volume change (∼+30 Å^3^ per formula unit) between the various room temperature structures and structure type F leads to poor single crystal quality at high temperatures and has meant that previous attempts to determine the structure were unsuccessful.^13–15^ Xiong et al. reported visually similar powder diffraction patterns for the high-temperature structures of both *Rac* **-Cu** and *S* **-Cu**. They reported a cubic cell in point group *m*3̅*m* with cell parameter *a* = 13.4408 Å and *Pm*3̅*m* as the most likely space group. Point group 432 was suggested for *S* **-Cu** due to the expectation that the chiral CTA^+^ would require a Sohncke cubic point group (23 or 432). This unit cell was also found for *Rac* **-Mn** and *Rac* **-Cd** by Xu et al., but they suggested space group *Fm*3̅*m*.^13^ The molecular arrangement was suggested to be related to the NaCl structure type and arises from large molecular displacements after the transition.

We have determined the crystal structure of phase F from high-temperature PXRD data of *Rac* **-Cd** at 437 K. A Pawley refinement (*R*_wp_ = 4.64%)^38^ gave a cubic cell with *a* = 13.5655(14) Å, and the systematic absences were consistent with *Fm*3̅*m*. Simulated annealing of a rigid body model was used to determine the structure. The face-centered cubic cell and the 2:1 ratio of cation:anion suggest an antifluorite-like structure. A rigid tetrahedron of CdCl_4_^2–^ was therefore positioned on Wyckoff site 4*a* with refinable rotations around all three axes. A semirigid model of *Rac*-CTA^+^ was positioned with the nitrogen atom near Wyckoff site 8*c*. Two hydroxy groups and the two adjacent hydrogen atoms were included with 12 occupancy to account for the racemic nature of *Rac*-CTA^+^. Four torsion angles were defined, so that the full conformation space of CTA^+^ could be explored. Rigid body rotations and translations of CTA^+^ were allowed to randomize and rerefined to convergence against the powder data for 100,000 least-squares iterations. The best structure obtained was found multiple times and used in a final Rietveld refinement (Figure 3). The final *R*_wp_ value was 4.7%, with only minor peak intensity discrepancies. These small differences are presumably due to the complex dynamic disorder present at high temperature and the likelihood of diffuse scatter due to correlated short-range order that is not captured in the average Bragg model. The space-group symmetry produces 48 orientations for the CdCl_4_^2–^ tetrahedron, which form a ccp (fcc) array. At the measured temperature, it is reasonable to expect these groups to be spherically disordered. The refined position of the organic molecule results in 24 orientations. The Me_3_N^+^ nitrogen sits at positions close to the tetrahedral holes in the ccp array (Figure 4). The propyl end of the molecule is directed toward the octahedral holes such that each octahedral hole can be thought of as partially filled by chlorine atoms of CTA^+^ molecules sitting in the 8 neighboring tetrahedral holes. A unit-cell search of the CSD found an analogous structure for (C_5_NH_13_Cl)_2_VOCl_4_ above 365 K (Refcode: AVUHEH02/03).^39^

**Figure 3:**
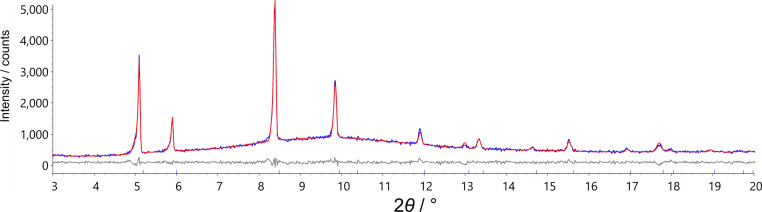
Rietveld fit of *Rac*-**Cd** in structure F showing the observed pattern (blue), the calculated pattern (red), and the difference curve (gray) at 437 K.

**Figure 4:**
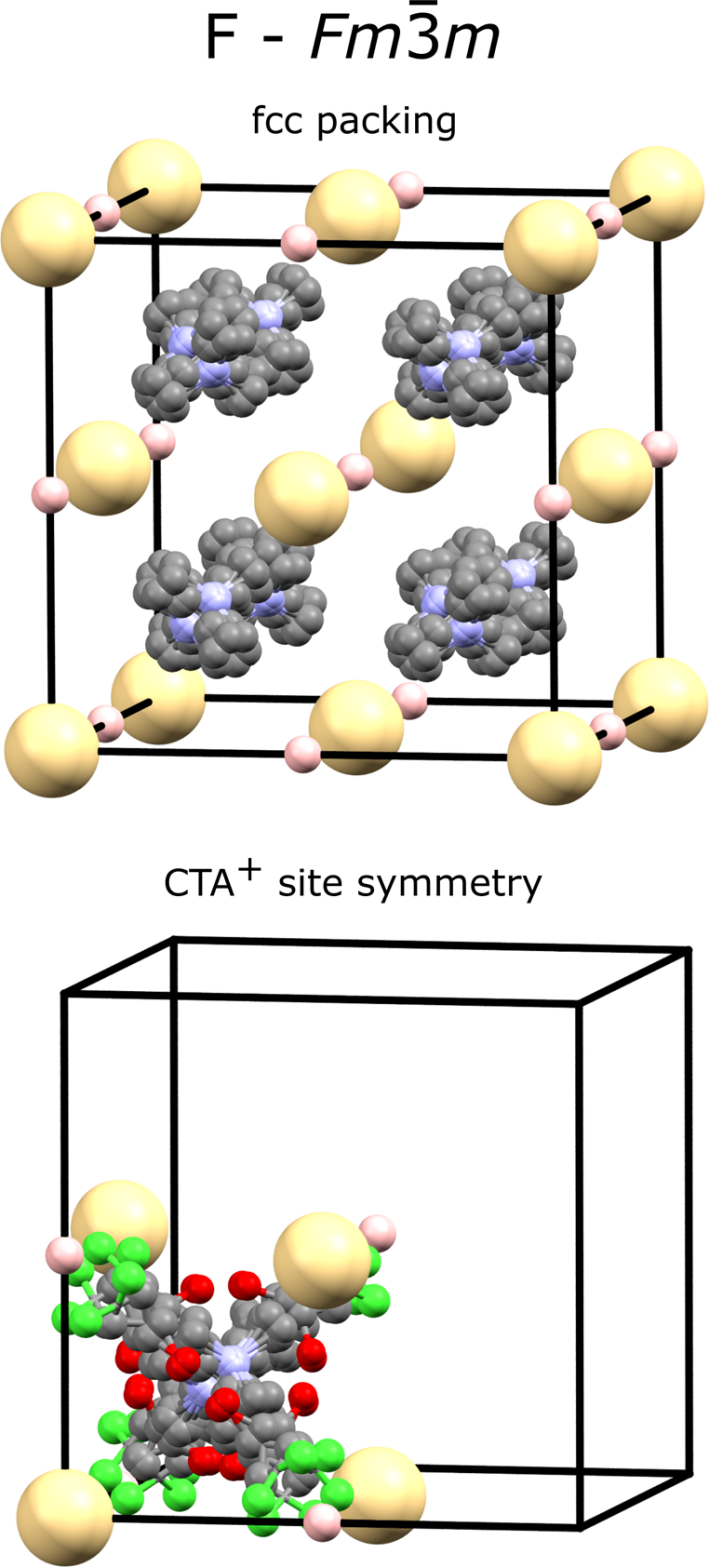
Molecular disorder model for *Rac*-**Cd** at high temperature showing the fcc CdCl_4_^2–^ array (yellow spheres) with the nitrogen of CTA^+^ close to the tetrahedral sites. (top, only showing Me_3_NCH_2_^+^ section) and the disordered molecular structure around a single CTA^+^ (bottom). A combination of rotational and static disorder increases the point symmetry to *T*_d_. Pink points show the locations of the empty octahedral sites.

It might be expected that as the enantiopure crystals contain chiral molecules, they should crystallize in a Sohncke space group at high temperature, the clear choice being *F*432, a *t*-subgroup of *Fm*3̅*m* that does not contain symmetry planes or inversion centers. This would be true if the disorder in phase F were static or dynamic and slow. However, if the thermal energy available at high temperature allows essentially free rotation about the 3-fold axis of the cubic space group faster than the time frame of the PXRD experiment, the electron density becomes evenly smeared around this 3-fold axis and essentially satisfies the inversion and mirror symmetry of *Fm*3̅*m.*

A representation of the close-packed structure of F is given in Figure 5 alongside equivalent views of structures A-E. Their structural relationships are discussed later.

**Figure 5:**
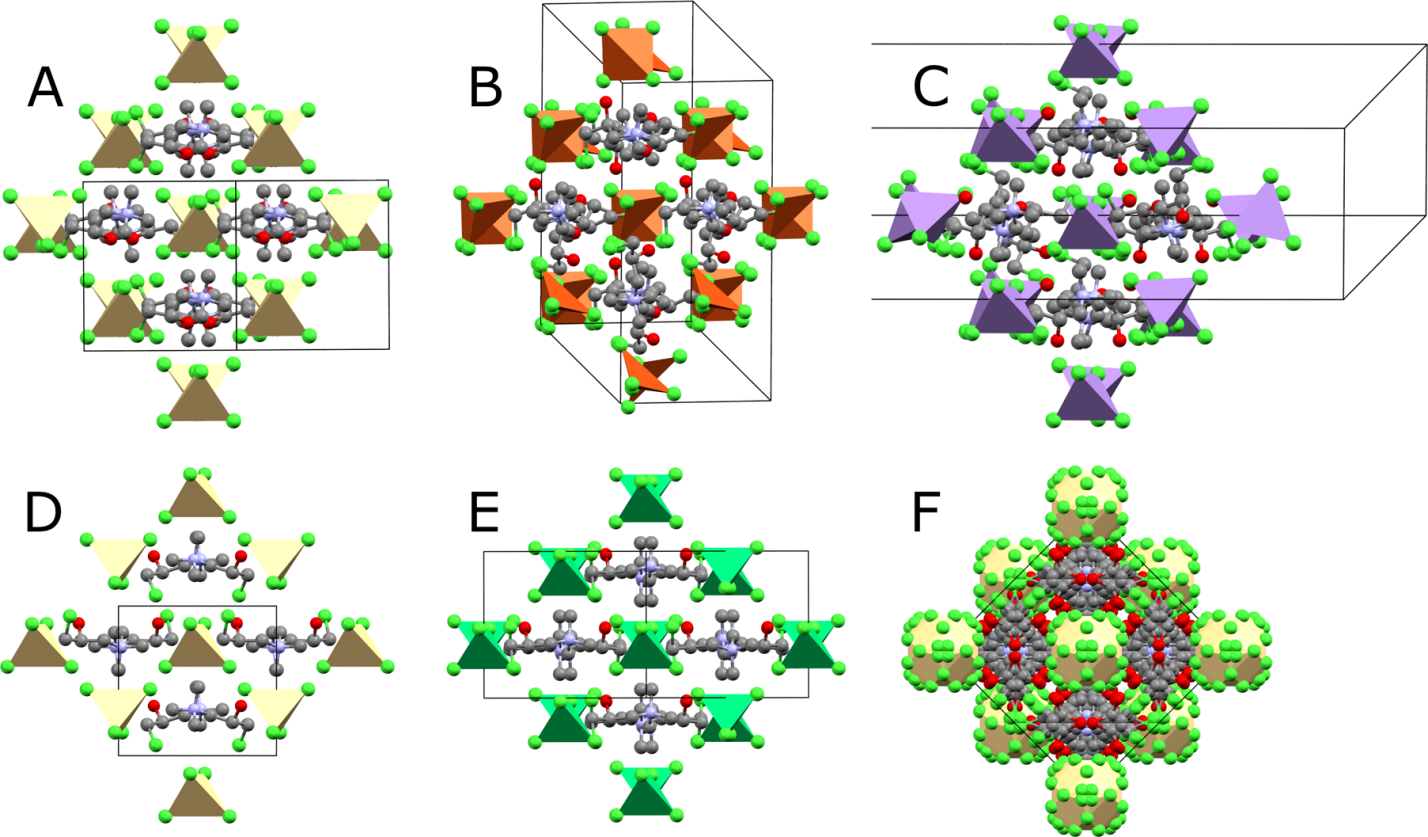
Comparison of all observed crystal structures viewed along the pseudocubic [100] direction. Equivalent portions of the structure are shown to emphasize their close structural relationship.

### CTA^+^ Dynamics Probed by Solid-State NMR

Solid state ^1^H NMR was used to probe the CTA^+^ dynamics on either side of the observed potential ferroelectric-paraelectric phase transition for diamagnetic *S* **-Cd**. As shown in Figure 6a, the ^1^H NMR spectrum narrows significantly at the temperature of the transition from phase D to phase F observed by VT-PXRD (*T*_c_ ≈ 346 K). This implies large-scale motion (i.e., dynamic disorder) at frequencies greater than the NMR line width (about 25 kHz) that averages the dipolar couplings between ^1^H spins; it is these couplings that are responsible for the line width of ^1^H spectra in solids. The data also show that the cubic site symmetry at high temperature is not purely achieved through dynamic disorder. In this case, the ^1^H line width would be close to zero, analogous to solution NMR peaks. It is likely that the dynamic motion causing the narrowing is a rapid rotation about the long axis of the molecule. This rotation axis is collinear with the *C*_3_ symmetry axis of the parent cubic structure (F).

**Figure 6:**
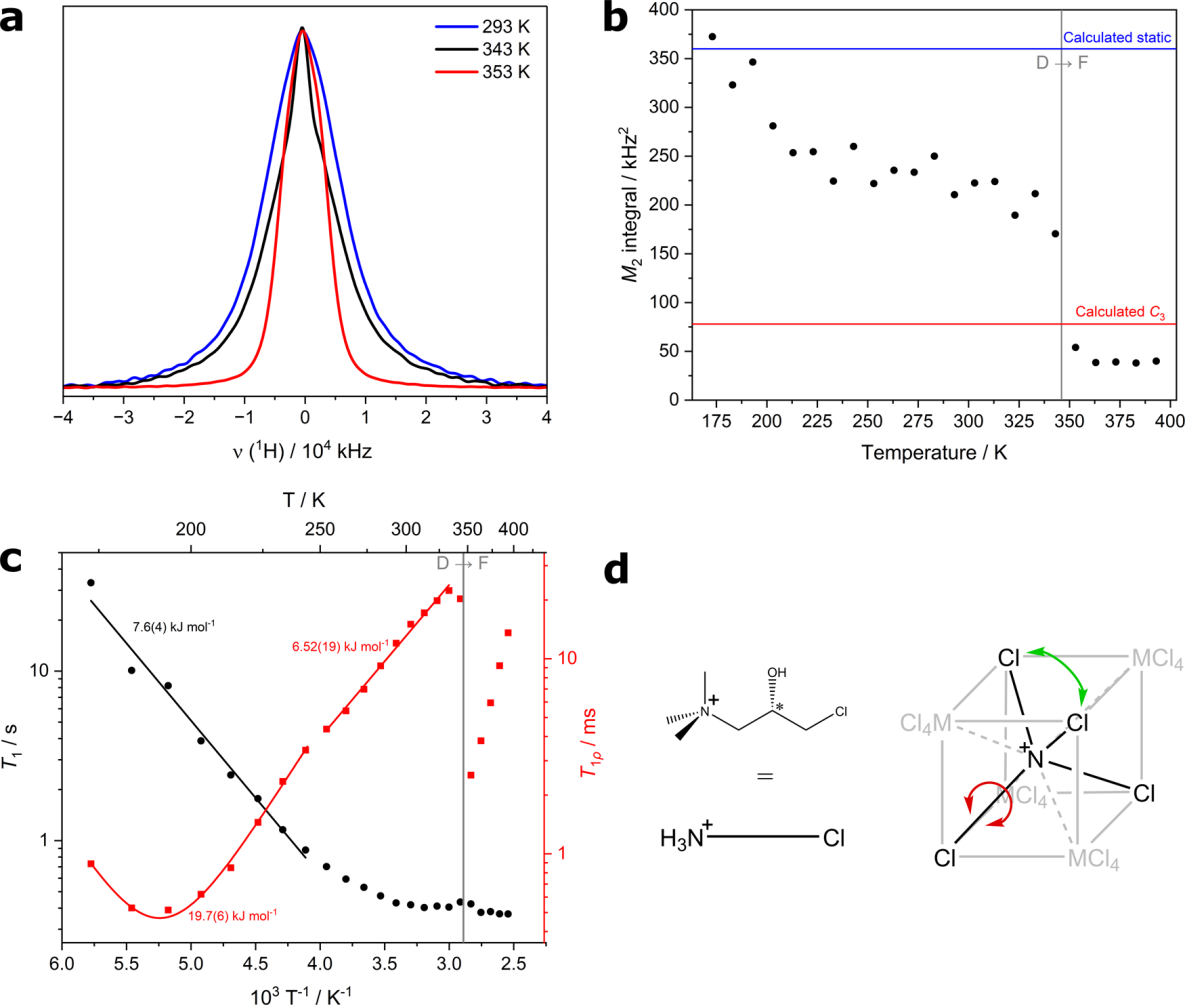
(a) Static ^1^H NMR spectra of *S*-**Cd** measured upon warming. (b) Effect of temperature on experimental *M*_2_ values (black). Calculated *M*_2_ values are given for the static (blue) and dynamic regimes (red). The vertical gray line indicates the phase transition temperature of D to F from VT-PXRD data. (c) Arrhenius-type plot of ^1^H relaxometry data measured on *S*-**Cd**. *T*_1_ data is plotted in black and *T*_1ρ_ data in red including fitted activation energies. (d) Average site symmetry of the CTA^+^ molecule in phase F. The red motion occurs rapidly, breaking the chirality. The green arrow represents a static or positional disorder in the crystal. The MCl_4_^2–^ and Cl positions are at the yellow and pink points of Figure 4, respectively.

More quantitative insight into the dynamics can be obtained from the ^1^H NMR data using the second moment analysis proposed by Van Vleck,^40^ which has previously been used in the context of molecular ferroelectrics.^41,42^ This extracts information about molecular dynamics from the line shape of NMR signals. The use of second moment analysis has traditionally faced practical challenges associated with both reliably quantifying the experimental data and with calculating theoretical values for extended systems. Recently, however, an efficient approach to calculating second moments in the presence of dynamics has been introduced, and it has been shown that second moment analysis can distinguish between different motions using diamantane and triamantane model systems.^43^ We use this approach to provide evidence that the proposed *C*_3_ rotation of CTA^+^ is, in fact, the process that becomes dynamic during the phase transition.

The calculation of the averaging effect of dynamics on ^1^H lineshapes is complicated when there are multiple motions occurring. In our case, in addition to the *C*_3_ rotations of CTA^+^, there are expected to be fast (compared to 25 kHz) rotations of all of the methyl groups. To make the calculations tractable, the previously developed code^30^ was extended to handle methyl dynamics as a special case.

The experimental *M*_2_ data are plotted alongside calculated values in Figure 6b. The static limit calculation (methyl motion only) of ∼350 kHz^2^ agrees well with the experiment at low temperature. It is notable, however, that the changing *M*_2_ values show that significant dynamics are present in the 200–325 K temperature range, and careful examination of the ^1^H lineshapes shows subtle changes that are more clearly observed in Figure 6b. This is likely due to a wobbling/crankshaft-type motion of the CTA^+^ alkyl chain. This motion can be probed using ^1^H *T*_1_ and *T*_1ρ_ relaxation time measurements, which are sensitive to dynamics on the order of the ^1^H Larmor frequency (here 400 MHz) and the ^1^H RF nutation frequency (here 50 kHz), respectively. Figure 6c shows a reduction in the measured *T*_1_ (corresponding to increased relatively fast dynamics) that coincides with the reduction in the *M*_2_ integral, supporting the conclusion that rapid motions around the backbone of the CTA^+^ molecule cause the observed decrease in *M*_2_ values for phase D. The modest activation energy of 7.6(4) kJ mol^–1^ obtained from fitting to the Arrhenius plot in Figure 6c is consistent with these types of motion. *T*_1_ stays consistently low above 300 K (rather than forming a well-defined minimum, as observed in the *T*_1ρ_ data), which implies that multiple fast processes are active in this temperature regime.

The change in ^1^H line shape at the D → F phase transition temperature (∼346 K) is clearly seen as a discontinuity in the *M*_2_ values. The calculated *M*_2_ value for a dynamic *C*_3_ rotation of CTA^+^ (around the red arrow in Figure 6d) is consistent with the experimental value, noting that the overall molecular motion is expected to be higher (leading to smaller experimental values) than predicted by this simple model, which does not include libration of the rotation axis. Slower motions like this can be probed by *T*_1ρ_, which is plotted in red in Figure 6c. At low temperature, *T*_1ρ_ goes through a minimum which we fit to extract activation parameters of *E*_a_ = 19.7(6) kJ mol^–1^ and log_10_(τ_∞_/Hz)= –10.90(13), cf. eq 2. This activation energy is on the order of magnitude expected for the *C*_3_ rotation.^44^ Pratum and Klein studied choline (from which CTA^+^ is derived) halide salts using ^2^H and ^14^N NMR. What they describe as a 180° flip-flop rotation is analogous to our *C*_3_ rotation: full molecular rotation around the long axis of the molecule. The calculated activation energies for choline iodide and choline chloride were 24.3 ± 4.1 and 46.0 ± 6.3 kJ mol^–1^, respectively. Our value is consistent with these, considering that the much larger size of CdCl_4_^2–^ over either halide ion increases the void space in the crystal and thus lowers the energy barrier to rotation. A similar effect explains the higher activation energy of the chloride salt over that of the iodide. The slightly larger volume of CTA^+^ over choline is a less significant size effect than that of the anion. Note that the ^1^H line shape cannot discriminate between the motion of the complete molecule and the motion of just the NMe_3_ fragment. Given the evidence for wobbling/crankshaft-type motions of the alkyl chain, it seems more plausible that the entire molecule is rotating about an effective molecular axis.

The *T*_1ρ_ activation energy observed here is similar to the activation energy of proton conductivity extracted from impedance spectroscopy data on a related compound, choline­[FeCl_4_].^45^ To ensure that the NMR data is probing molecular rotations rather than proton conductivity, we recorded impedance spectroscopy data on *S* **-Cd**. Data recorded on a dry sample at 330 K showed a conductivity of 2.29 × 10^–9^ S cm^–1^ well within the insulating regime.

A discontinuity is also observed in *T*_1ρ_ at the D → F phase transition. As noted above, multiple motional processes are active above 250 K, so it is not meaningful to analyze the data (or the apparent *E*_a_) in terms of individual molecular motions.

It is important to note that if a pivot-type motion such as the green motion in Figure 6d was present and rapid on the NMR time scale, the NMR line width would become close to zero. We conclude from this that positional disorder must also contribute to the cubic site symmetry of the CTA^+^ molecule and may impact the multiaxial properties of these materials.

### Relationship between Structures A–F

All members of this family adopt the same high-temperature cubic antifluorite-related structure. However, a variety of apparently disparate structures emerge at lower temperatures, which depend on the metal and thermal history. They can, however, all be related using group-subgroup relationships.^46^ A group-subgroup tree containing each observed structure and its relationship to the parent *Fm*3̅*m* structure is shown in Figure 7. Matrix transformations relating the observed low symmetry cells to the parent are given in Table 3.

**Figure 7:**
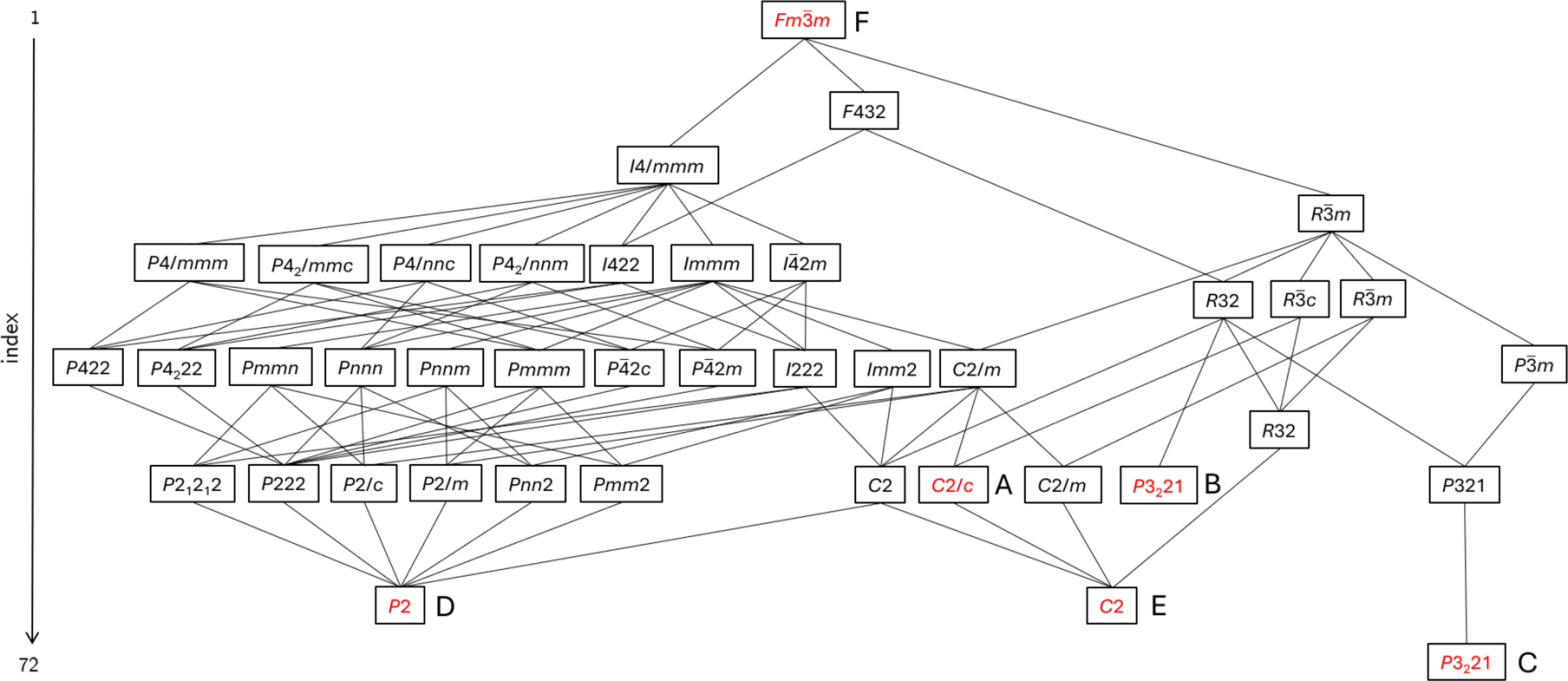
Group-subgroup tree from *Fm3̅m* to selected subgroups. Experimentally observed structures are colored red. Structures are ordered by subgroup order.

**Table 3:** Low Temperature Unit-Cell Relationships to Cubic Phase F

structure type	metal	space group	transformation matrix
B	Cu	*P*3_2_21	[12012012−12−111]
C	Co, Mn	*P*3_2_21	[12−120012−12333]
D	Cd	*P*2	[12120−12120001]
E	Zn, Ni	*C*2	[−1212112120−1212−1]

In the transition from structure type F to its child structures on cooling, the MCl_4_^2–^ anion changes from spherical disorder (presumably due to rapid tumbling) to adopting a single orientation at low temperature. The CTA^+^ molecules also adopt a single orientation of one of the three low-energy conformers. A ‘memory’ of the parent cubic structure is retained for all structures via the pseudocubic-close-packed arrangement of MCl_4_^2–^ anions with the CTA^+^ N-group in approximately tetrahedral coordination by MCl_4_^2–^ and with its long axis directed toward one of the four neighboring octahedral holes. This is shown in Figure 4.

The polymorphs A-E are all closely related on the subgroup tree, which is clear from their similar crystal structures. Polymorph C is remote from any other polymorph. This is reflected in its significantly different structure and its different thermal behavior, which suggests only kinetic stability. Polymorph B is distinct due to the distortions of CuCl_4_^2–^, which allow all CTA^+^ molecules to adopt conformer 1.

The CTA^+^ conformers present in phase F cannot be directly determined from the PXRD data due to the data quality at high temperatures and the rapid molecular reorientation that occurs. It is likely that the available thermal energy leads to molecules rapidly interconverting between conformers 1–3 (there is only a 1.1 kJ mol^–1^ energy difference between conformers 1 and 3), as well as higher energy conformers at this temperature.

### Polarization of Structures D and E

Of the new structure types discovered, D (*S*-**Cd**) and E (*S*-**Zn**, *S*-**Ni**) crystallize in pyroelectric space groups. Their significant symmetry increase from monoclinic to cubic (*m*3̅*mFm* in Aizu notation^47^) at the high temperature phase transitions suggests they have the potential to be multiaxial ferroelectrics with 24 equiv polarization directions. The moderate temperature of these phase transitions indicates that the *C*_3_ dynamic rotation driving force has a low energy barrier and can operate as a ferroelectric switching mechanism.

The polarization in these structures predominantly arises from the parallel arrangement of the C–O bond vector with the *b*-axis. CTA^+^ rotation of 180° about the *C*_3_ axis reverses the polarization such that the net C–O bond vector component points antiparallel to the *b*-axis.

The spontaneous polarizations of *S*-**Cd** and *S*-**Zn** were calculated using DFT across the ferroelectric to paraelectric order parameter. The predicted spontaneous polarizations for structure types D and E are 2.25 and 2.11 μC cm^–2^, respectively. These are moderate values for molecular ferroelectrics and are in the range of notable examples such as triglycine sulfate (2.8 μC cm^–2^ at 293 K) and the chiral plastic crystal (*R*)-(−)-3-hydroxlyquinuclidinium chloride (1.7 μC cm^–2^).

## Conclusions

We synthesized five new chiral compounds by introducing *S*-CTA^+^ into previously known achiral structures. All undergo a symmetry-raising phase transition on heating to the same high-temperature parent structure facilitated by the rapid rotation of the *S*-CTA^+^ molecules. The rotational motions that occur have been probed using solid-state NMR. The high-temperature structure was solved from PXRD and shown to be related to the antifluorite structure. The symmetry relationships between all observed structures were rationalized on a group-subgroup tree. Three of the new structures are polar and potentially ferroelectric. The large increase in symmetry at the phase transitions and the calculated spontaneous polarization values suggest that these structures may be useful in devices.

## Supplementary Material





## Notes

Single-crystal X-ray diffraction structures and data; powder X-ray diffraction data and Rietveld refinement information for *S* **-M** structures at room temperature, *Rac* **-Cd** at 437 K and *S* **-Cd** at 449 K; variable temperature static ^1^H solid-state NMR spectra including the *M*_2_ integrating script and CIF file used to calculate the dynamic *M*_2_ value; and *T*_1_ and *T*_1ρ_ fitting graphs and parameters including Arrhenius fitting scripts
NMR, PXRD, and SXRD details (PDF, ZIP)
