# Correction to “Potential Multiaxial Molecular Ferroelectricity through Chiral Cation Replacement”

**DOI:** 10.1021/acs.cgd.6c00647

**Published:** 2026-06-01

**Authors:** Sam Y. Thompson, Rebecca H. Abeyasekere, Samuel J. Page, Paul Hodgkinson, Cameron A. M. Scott, Nicholas C. Bristowe, Oliver J. Wagstaff, John S. O. Evans

Some of the information published in the for *S*-Ni (, refcode KAHXEB) contained errors. New versions of the have been uploaded to match the correct information in the CSD. The changes have no impact on the conclusions of the paper, and the corrected coordinates are unchanged within errors, with a root mean squared displacement of atom positions of 0.003 Å.

## Supplementary Material





## Notes

Single-crystal X-ray diffraction structures and data; powder X-ray diffraction data and Rietveld refinement information for *S* **-M** structures at room temperature, *Rac* **-Cd** at 437 K and *S* **-Cd** at 449 K; variable temperature static ^1^H solid-state NMR spectra including the *M*_2_ integrating script and CIF file used to calculate the dynamic *M*_2_ value. *T*_1_ and *T*_1ρ_ fitting graphs and parameters including Arrhenius fitting scripts (PDF)
NMR, PXRD, and SXRD details (ZIP)

