# Supplementary material for: Potential Multiaxial Molecular Ferroelectricity through Chiral Cation Replacement
Source: Cryst Growth Des. 2025 Jul 21;25(15):6237–47. doi: 10.1021/acs.cgd.5c00666 (PMC12332970; doi:10.1021/acs.cgd.5c00666)
Supplement: Supplementary file 2 [file cg5c00666_si_002.zip › NMR/jse_20240130_3_to_118_T1_output.pdf]

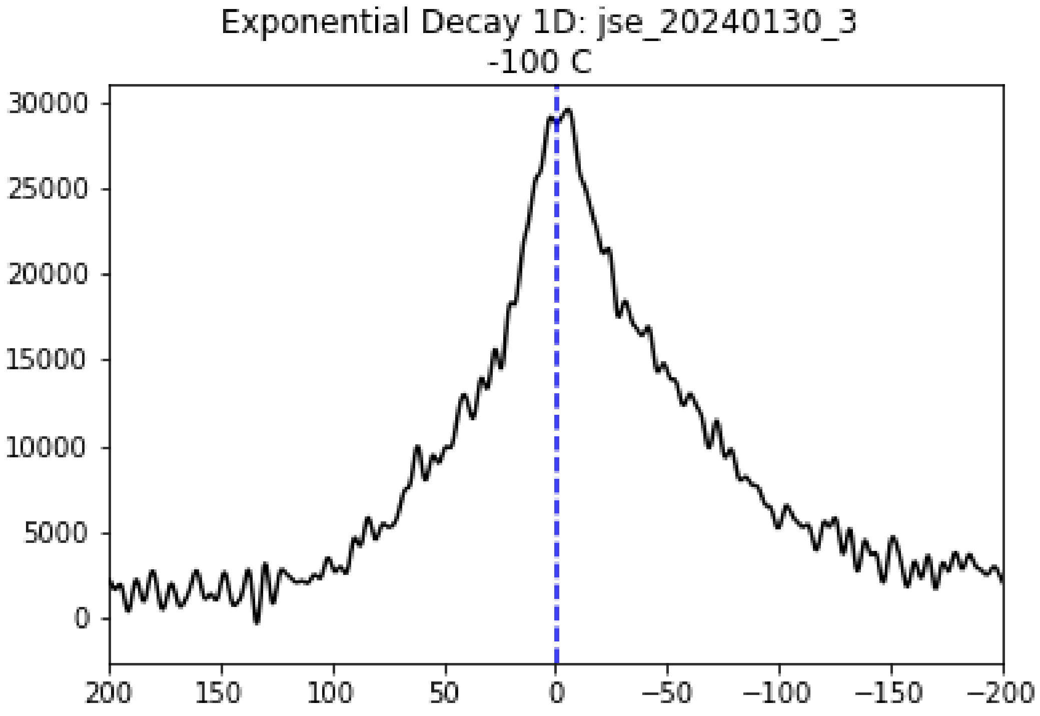

File 1: .../T1_plot/jse_20240130_3_figure.png

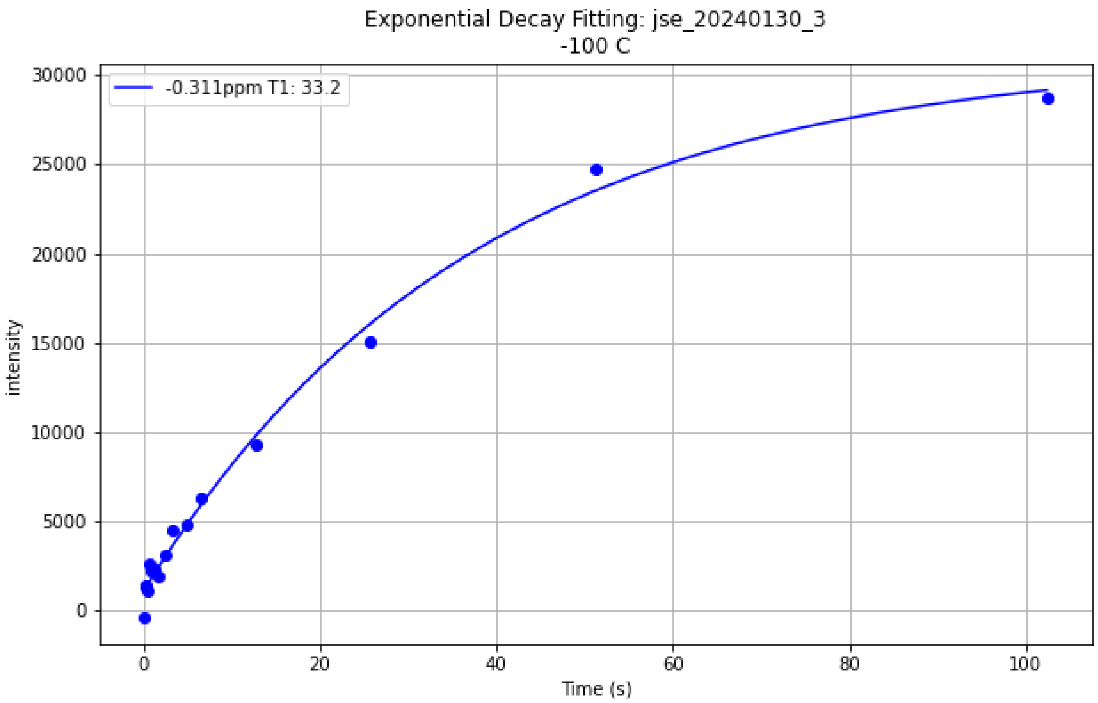

File 2: .../T1_plot/jse_T1_3.png

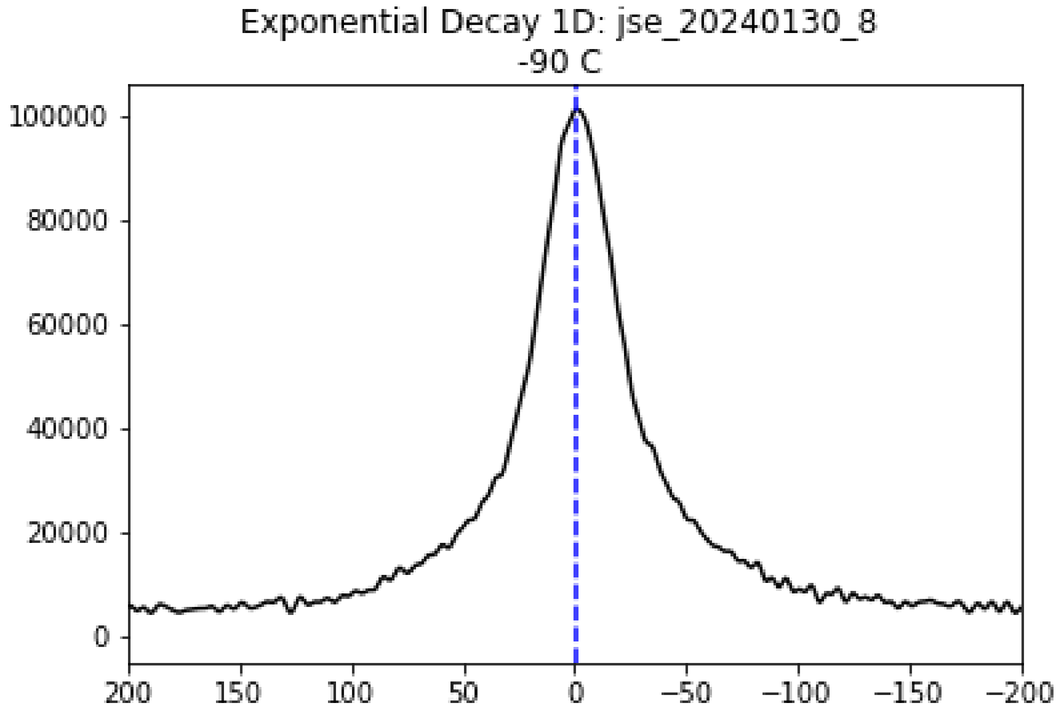

File 3: .../T1_plot/jse_20240130_8_figure.png

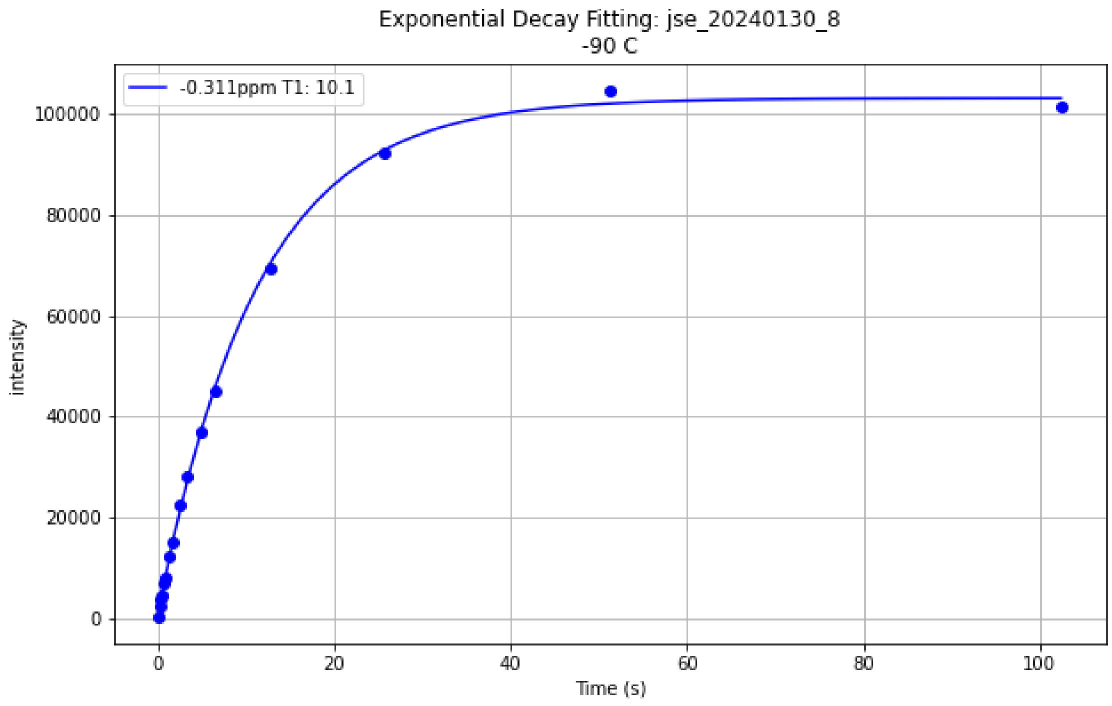

File 4: .../T1_plot/jse_T1_8.png

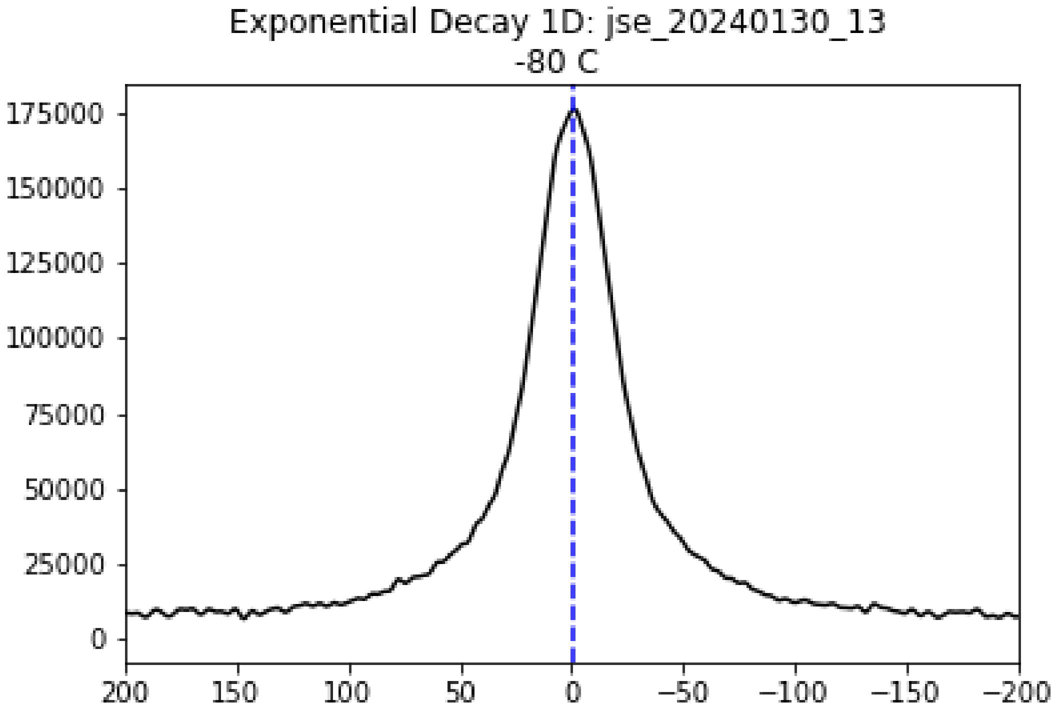

File 5: .../T1_plot/jse_20240130_13_figure.png

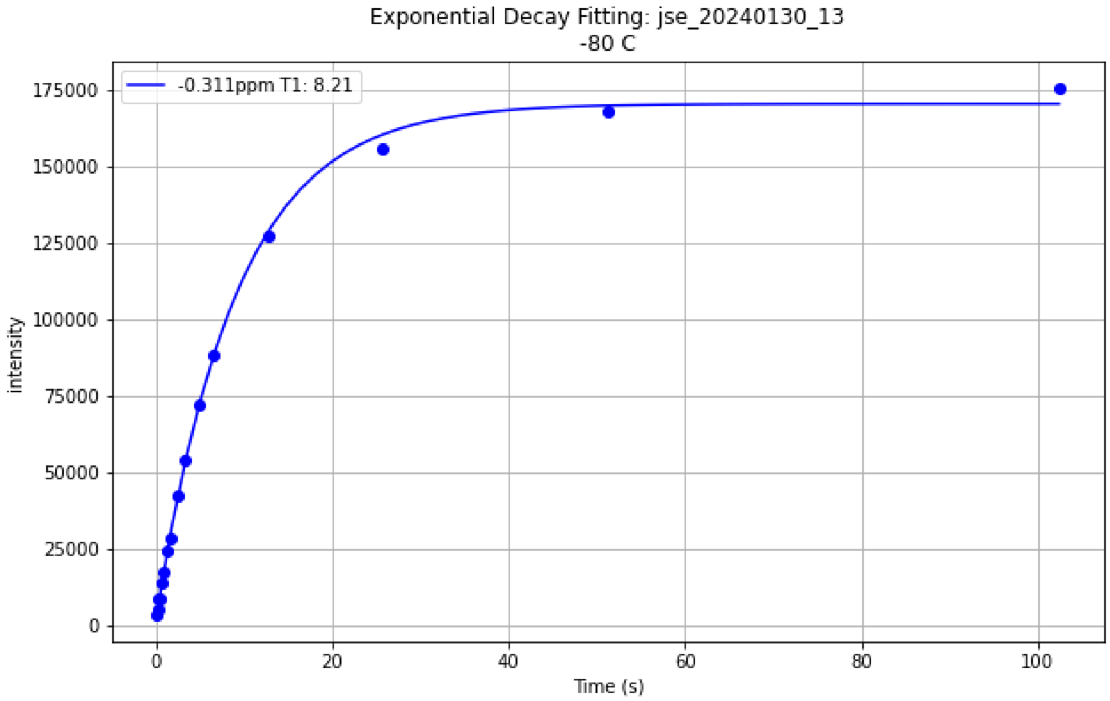

File 6: .../T1_plot/jse_T1_13.png

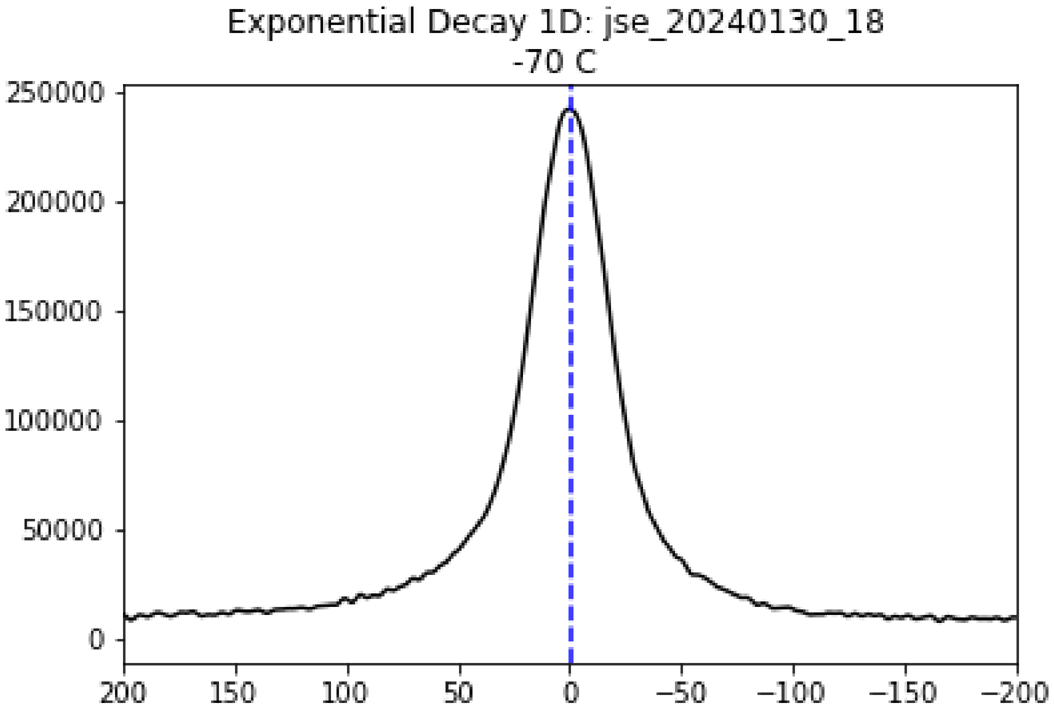

File 7: .../T1_plot/jse_20240130_18_figure.png

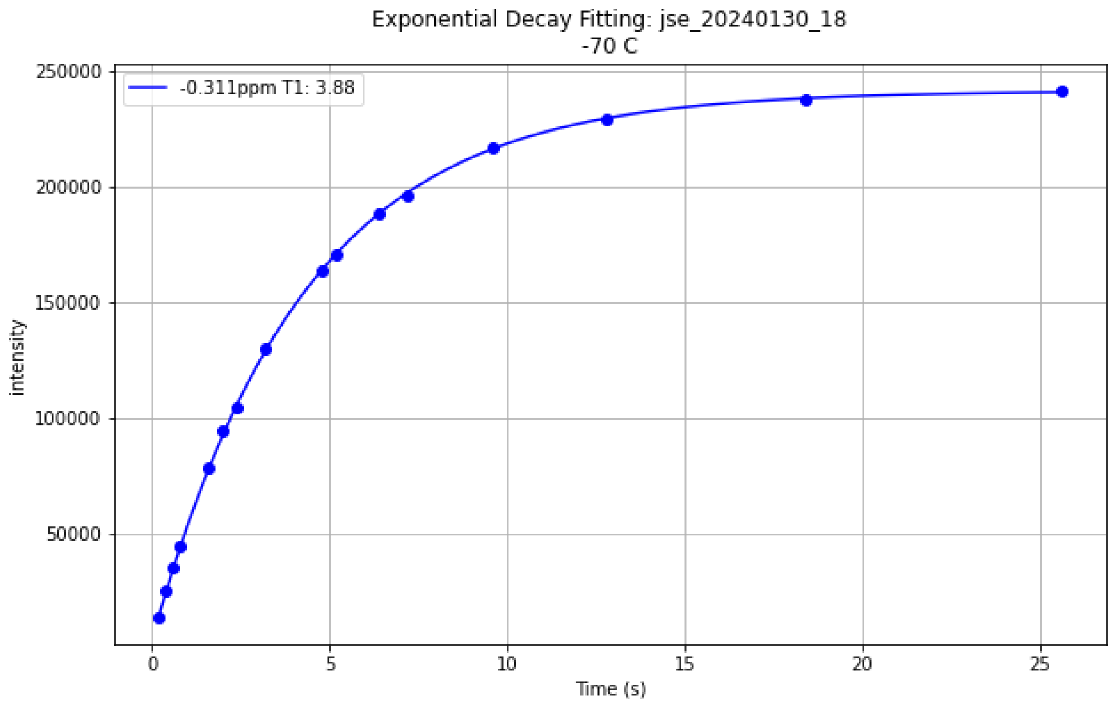

File 8: .../T1_plot/jse_T1_18.png

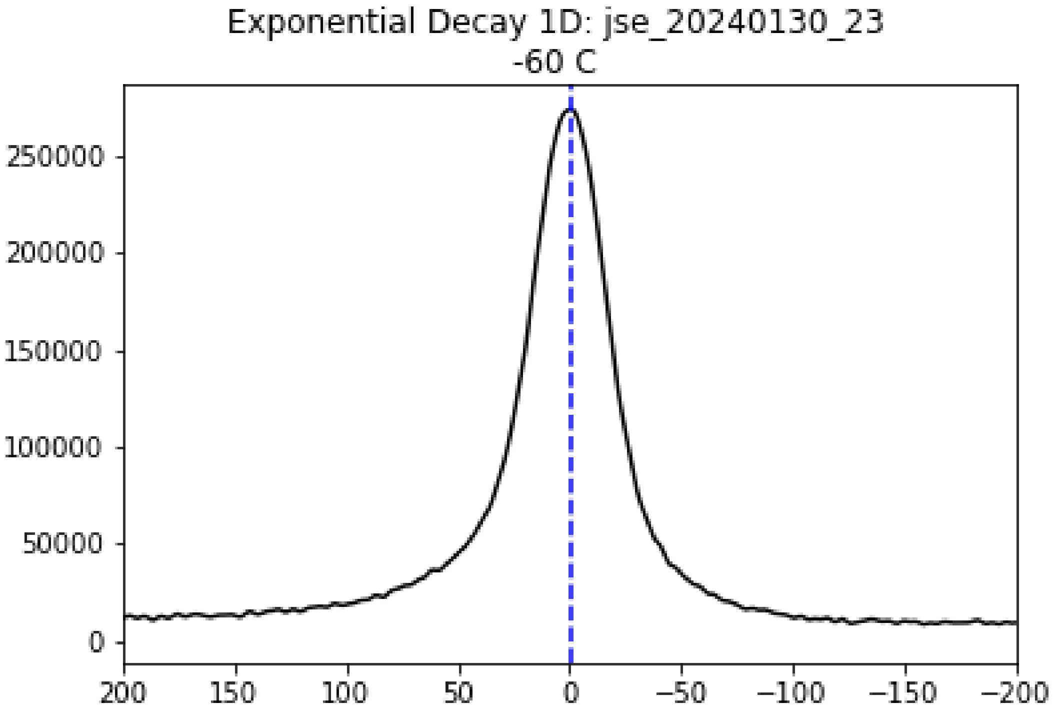

File 9: .../T1_plot/jse_20240130_23_figure.png

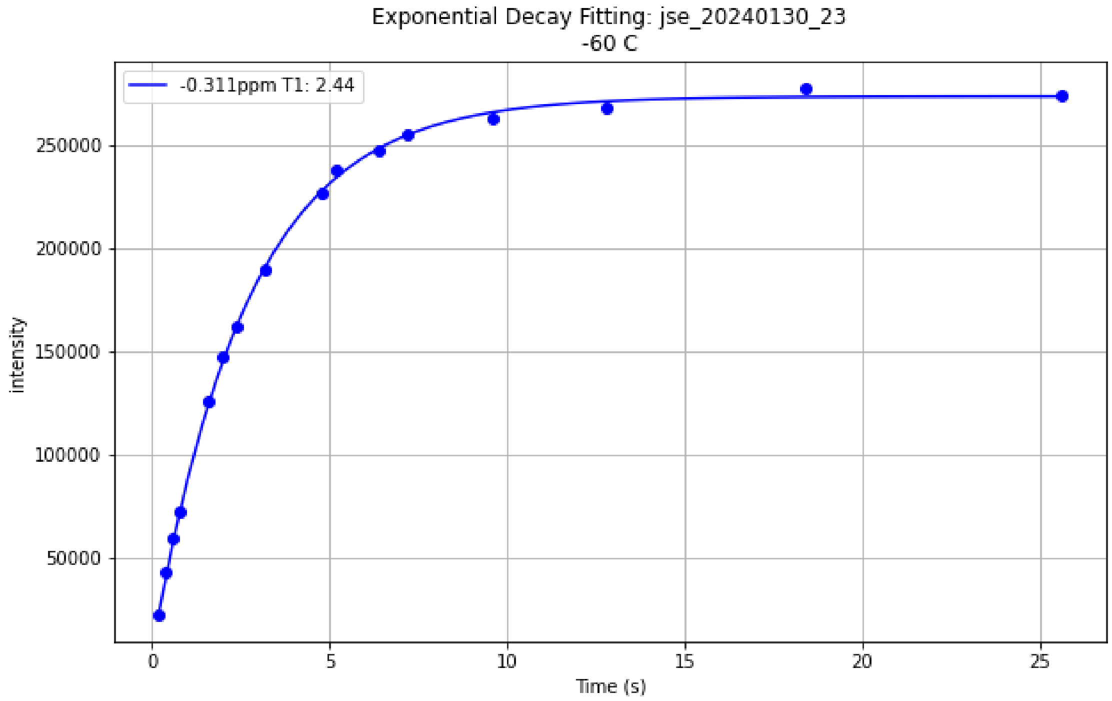

File 10: .../T1_plot/jse_T1_23.png

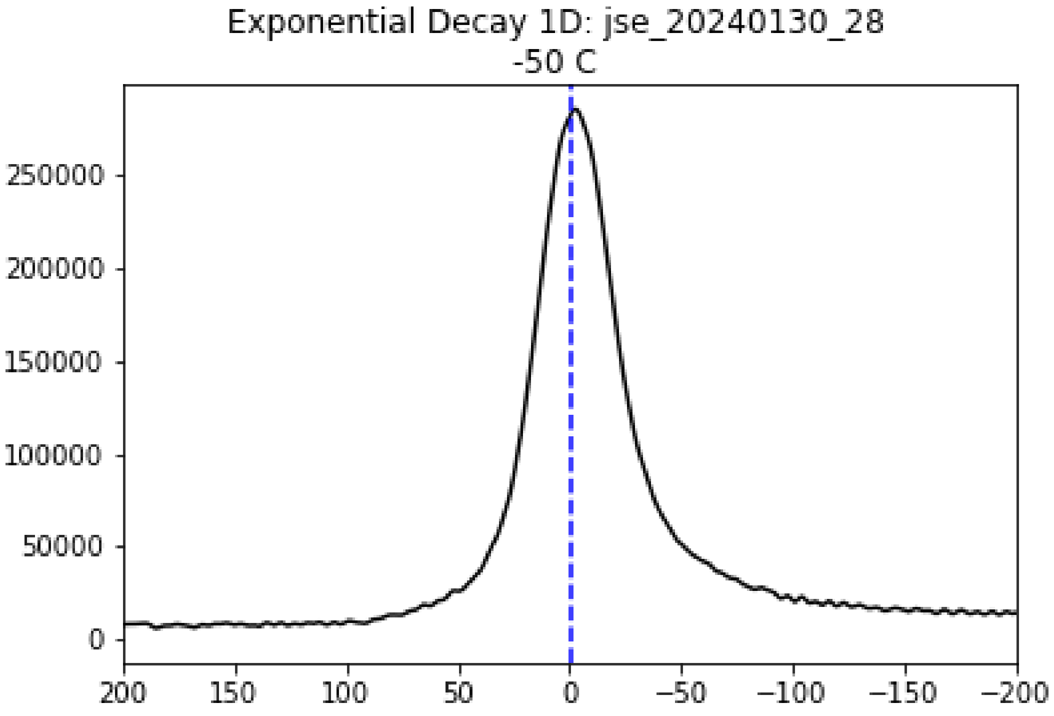

File 11: .../T1_plot/jse_20240130_28_figure.png

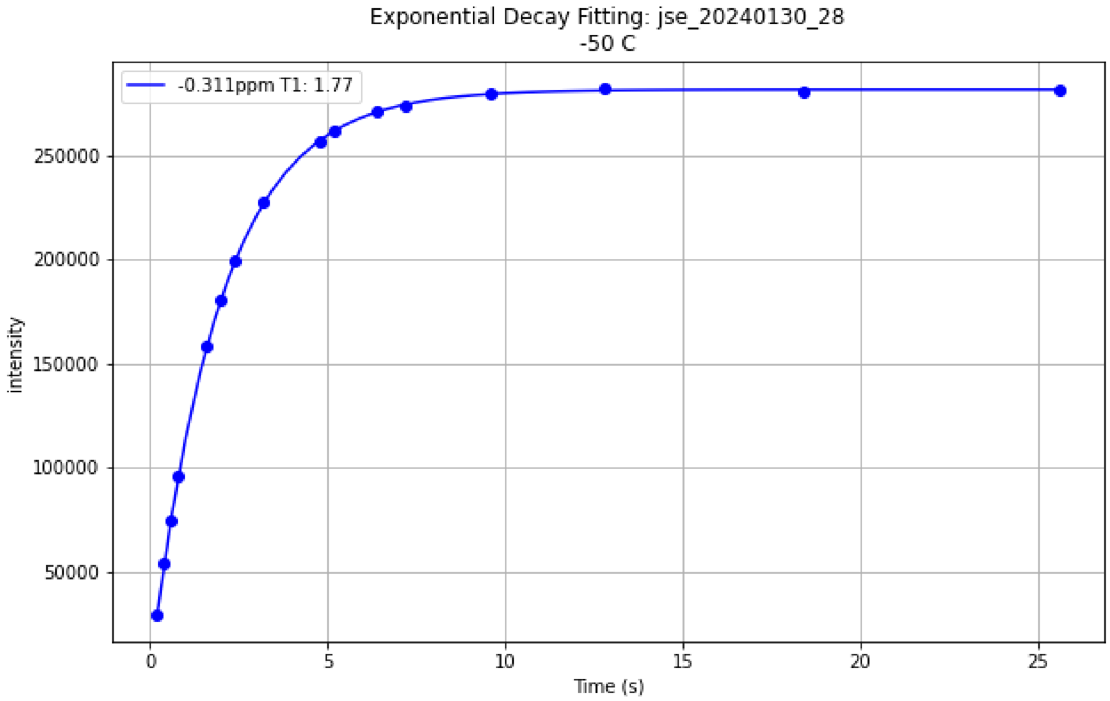

File 12: .../T1_plot/jse_T1_28.png

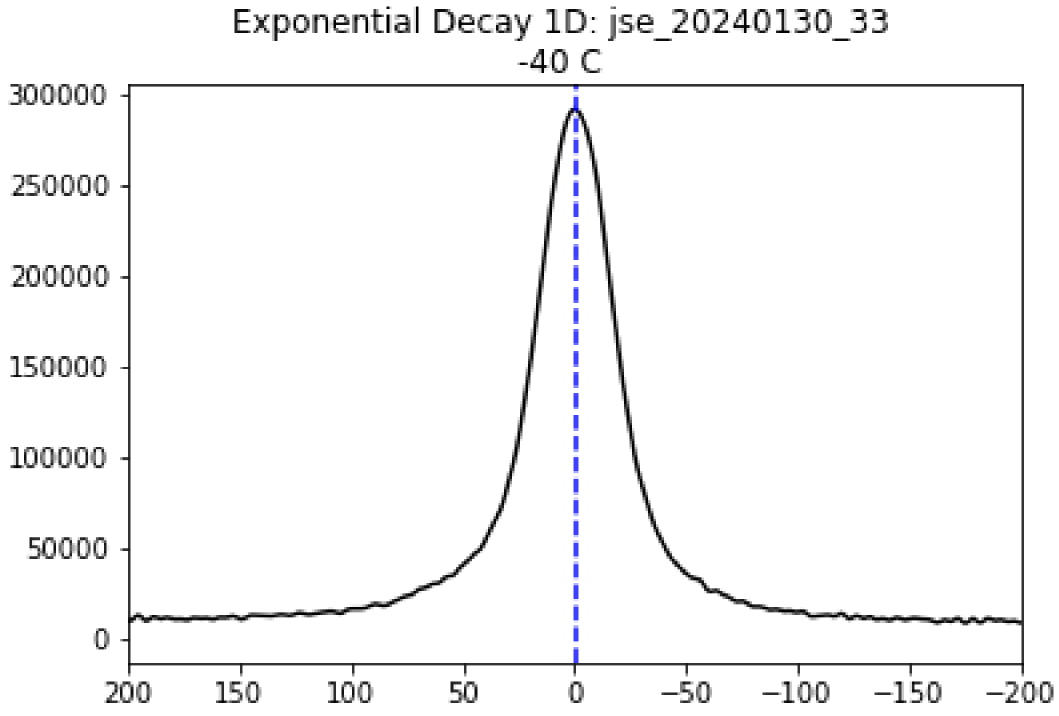

File 13: .../T1_plot/jse_20240130_33_figure.png

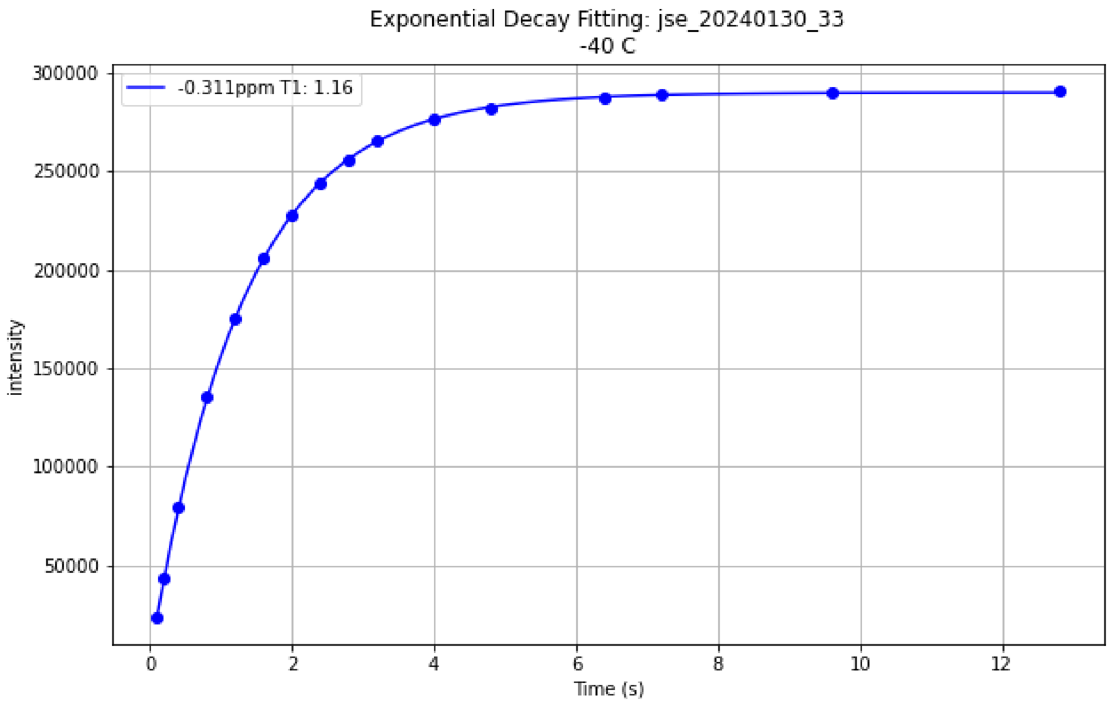

File 14: .../T1_plot/jse_T1_33.png

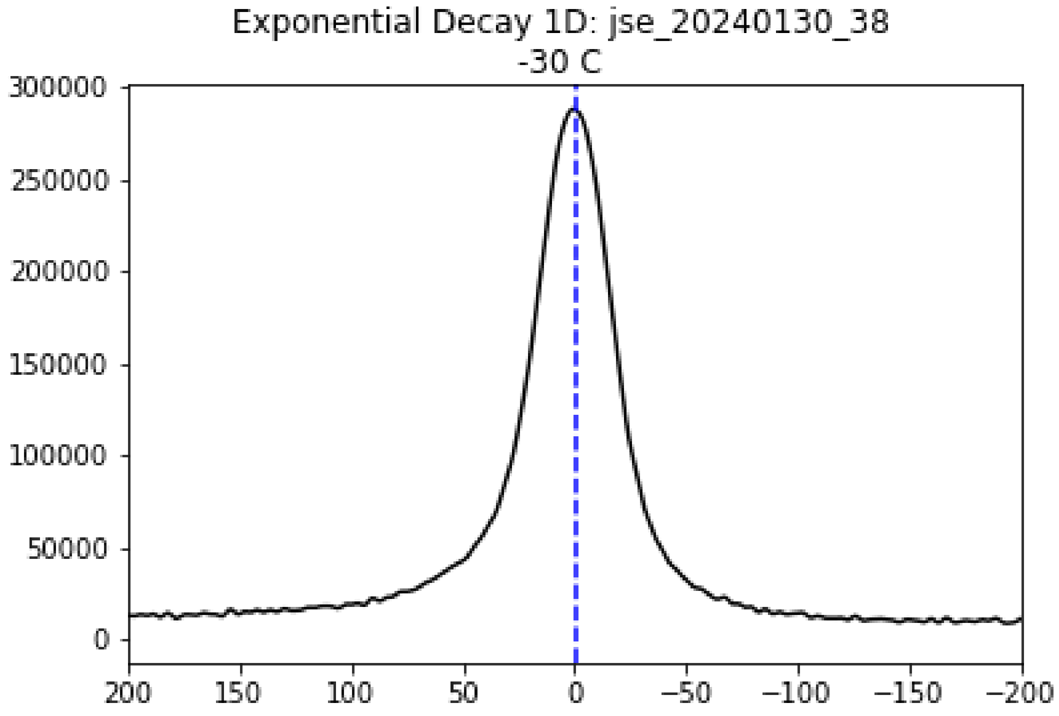

File 15: .../T1_plot/jse_20240130_38_figure.png

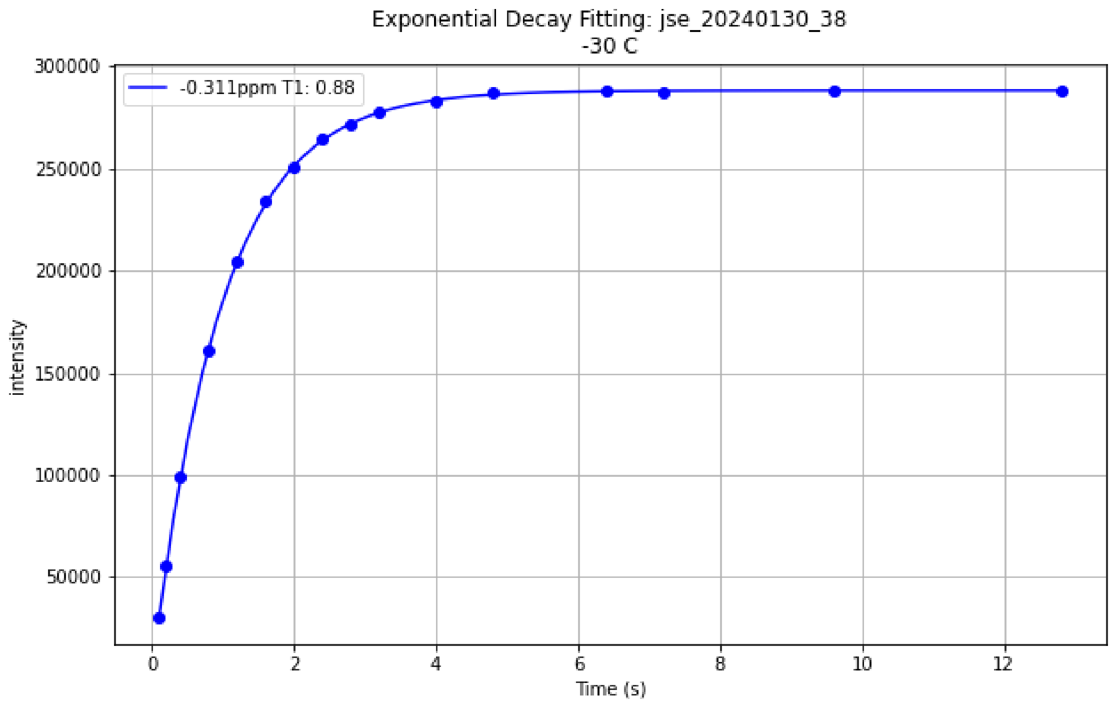

File 16: .../T1_plot/jse_T1_38.png

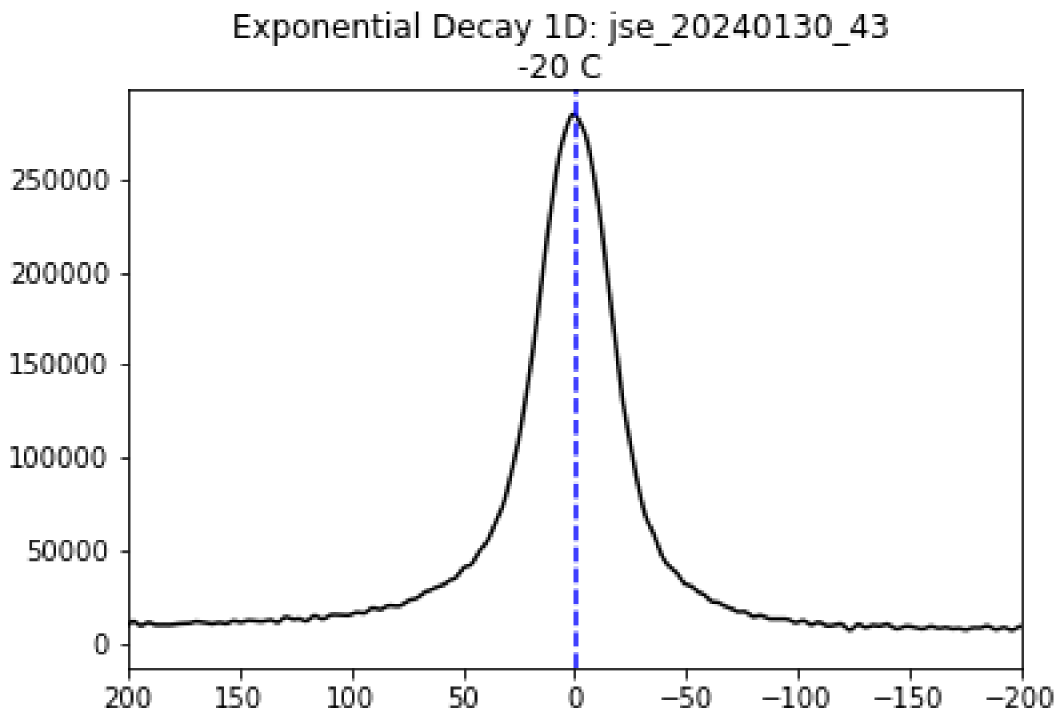

File 17: .../T1_plot/jse_20240130_43_figure.png

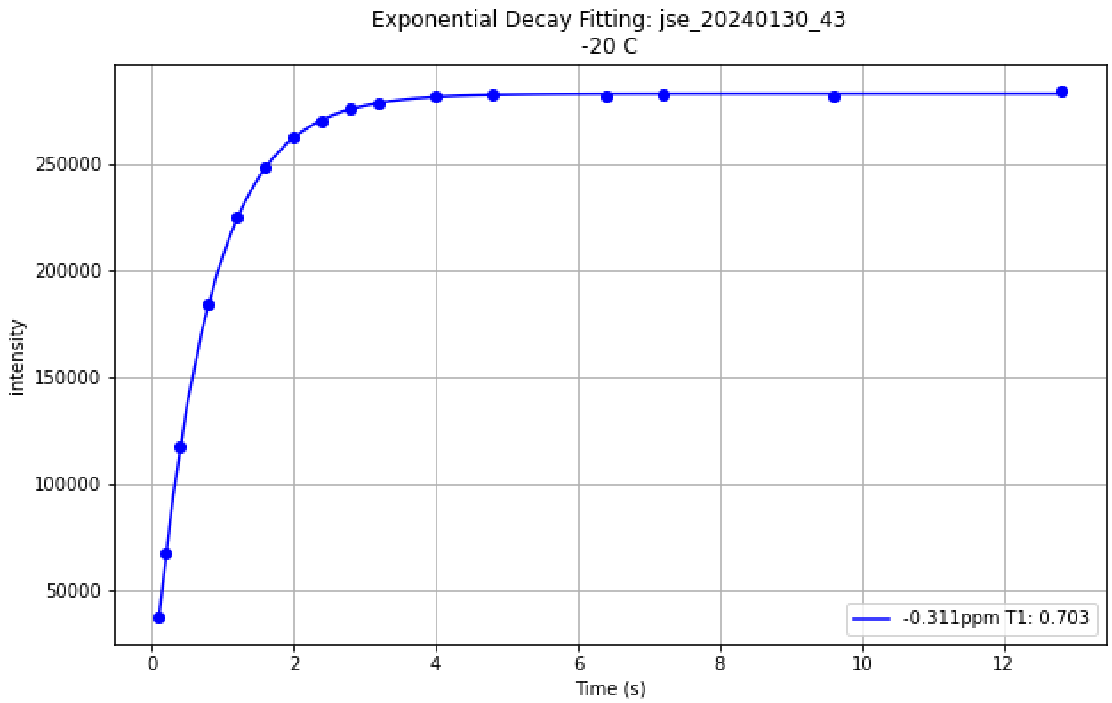

File 18: .../T1_plot/jse_T1_43.png

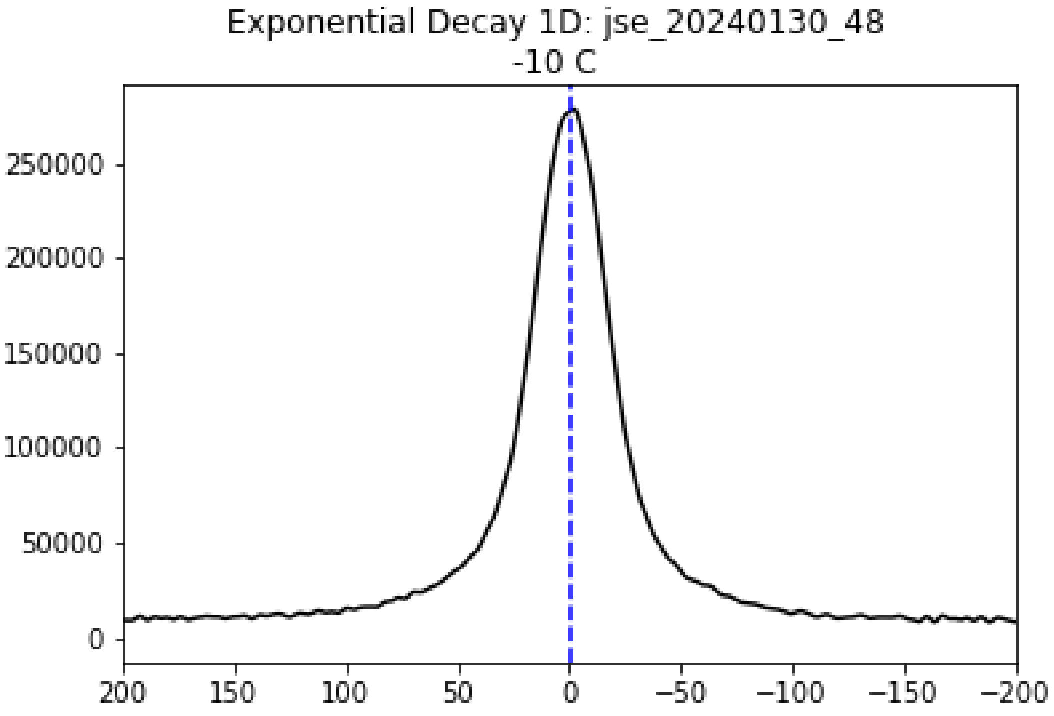

File 19: .../T1_plot/jse_20240130_48_figure.png

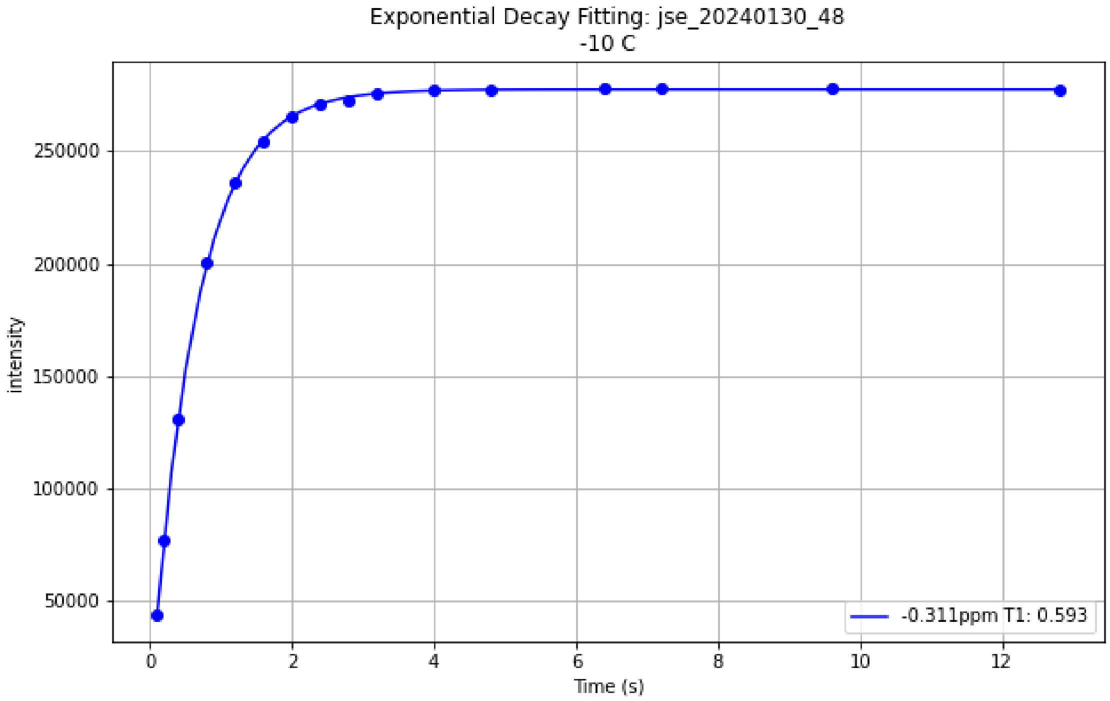

File 20: .../T1_plot/jse_T1_48.png

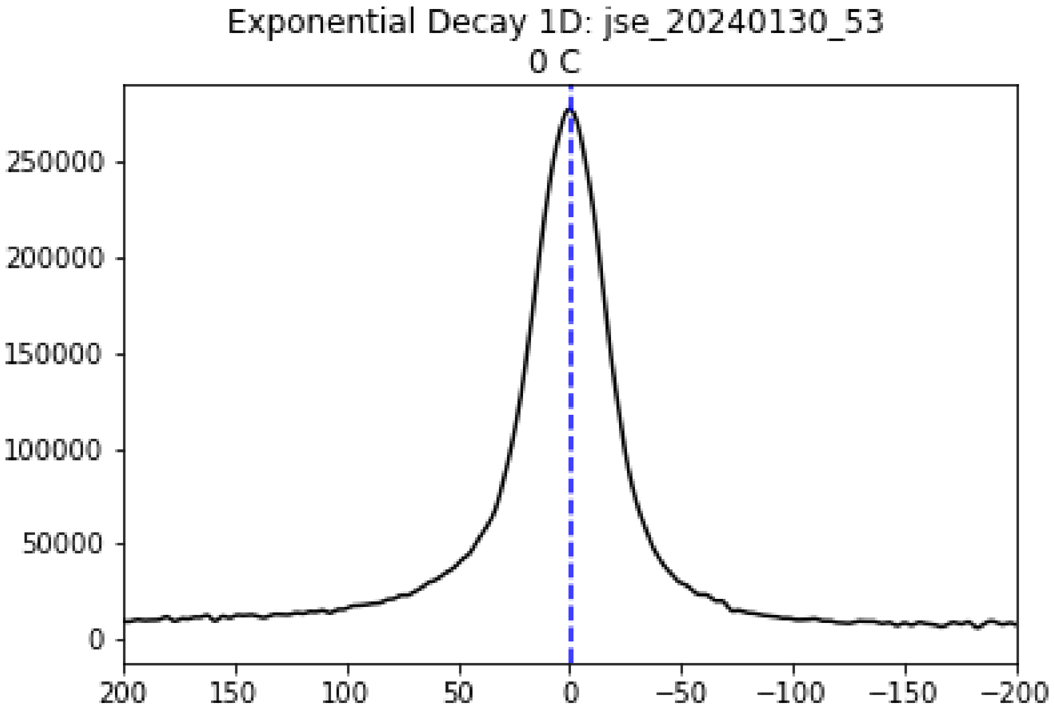

File 21: .../T1_plot/jse_20240130_53_figure.png

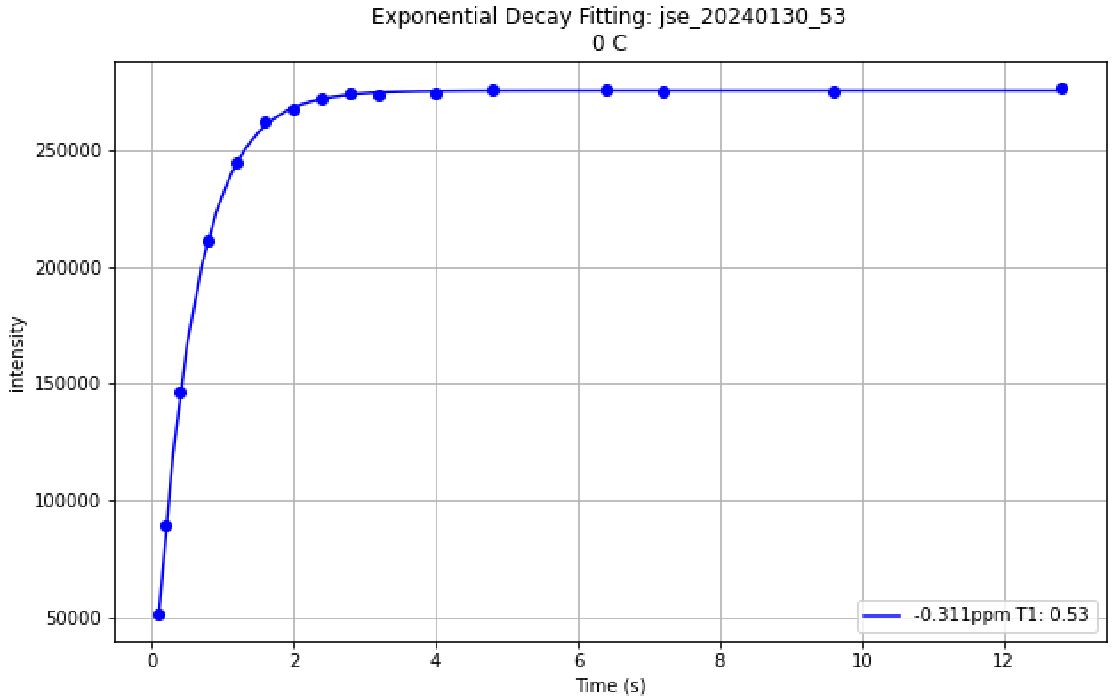

File 22: .../T1_plot/jse_T1_53.png

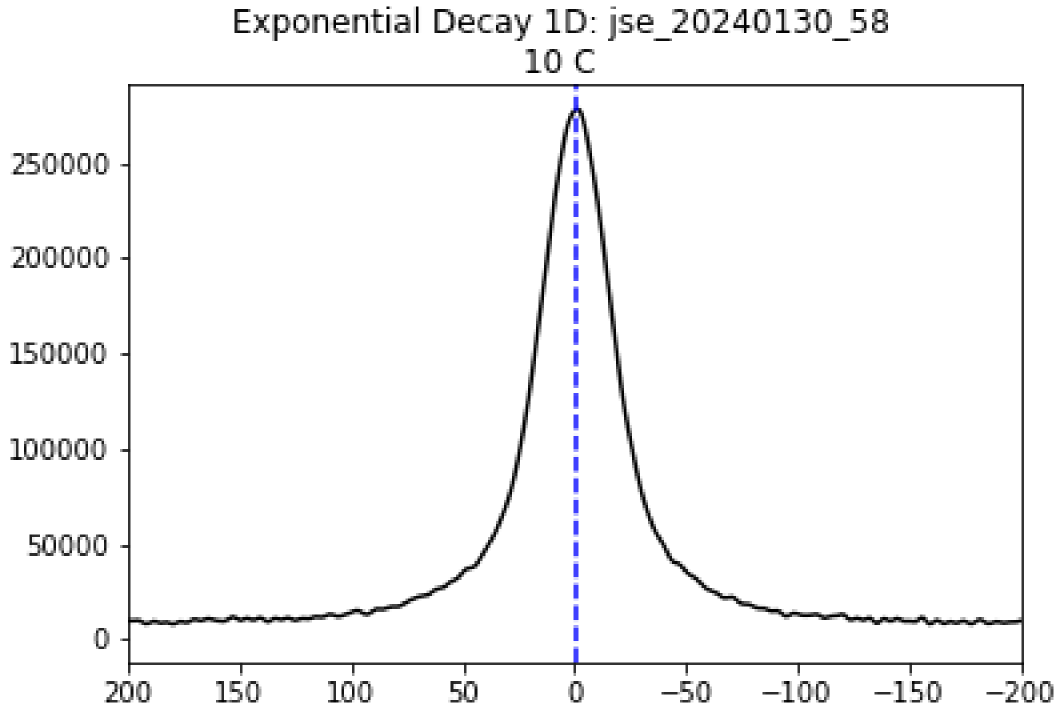

File 23: .../T1_plot/jse_20240130_58_figure.png

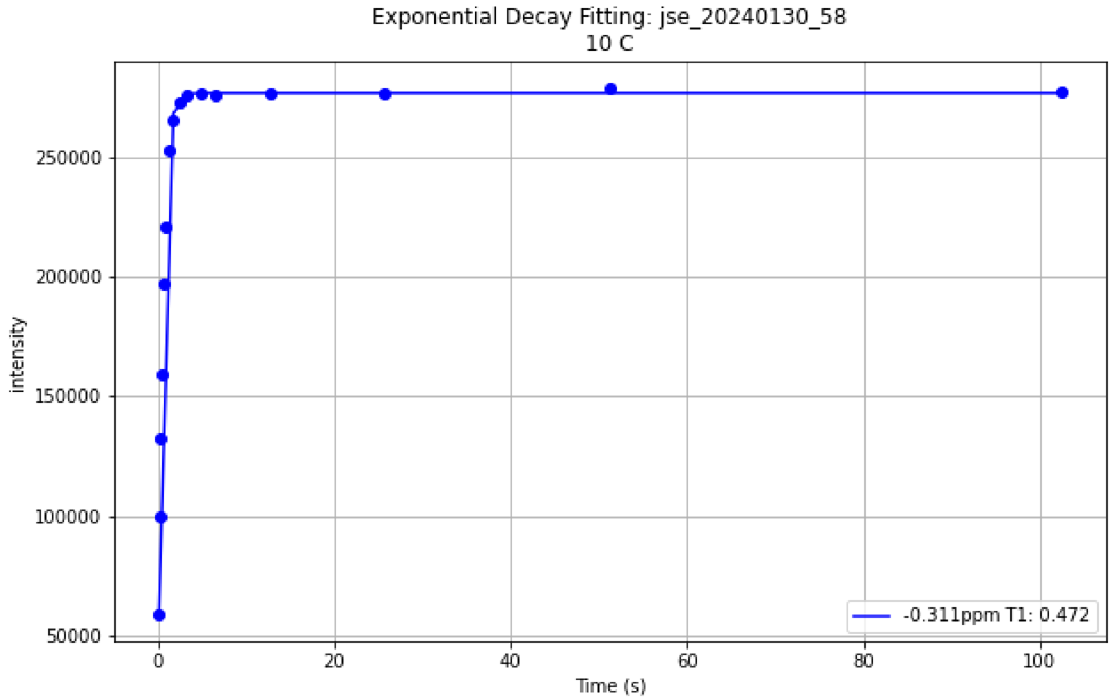

File 24: .../T1_plot/jse_T1_58.png

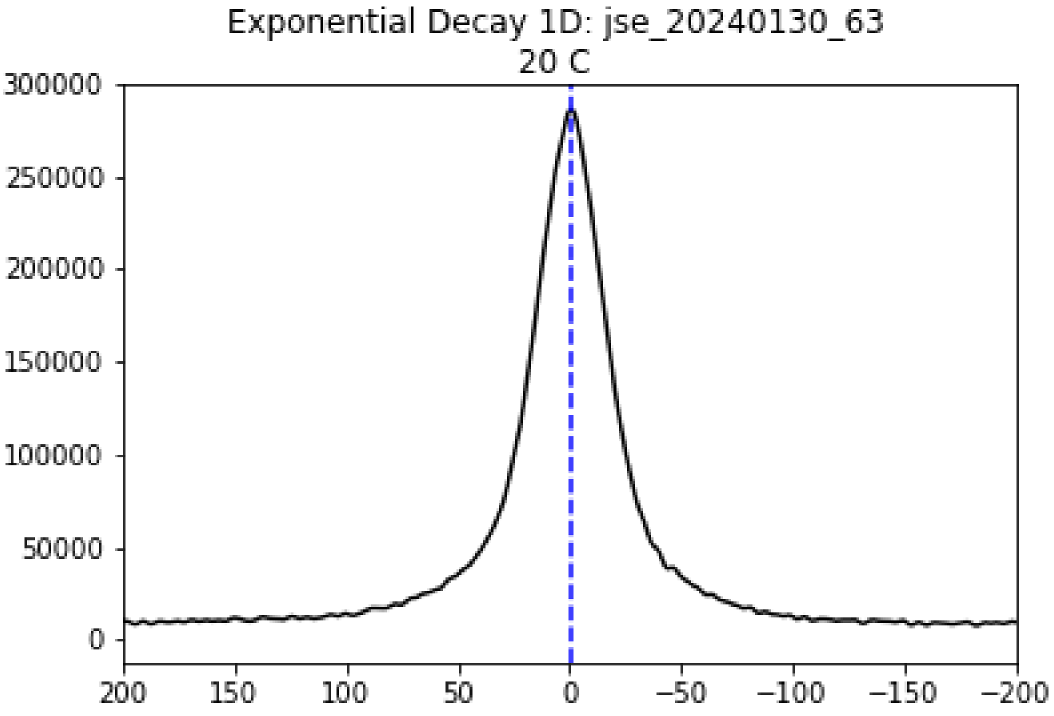

File 25: .../T1_plot/jse_20240130_63_figure.png

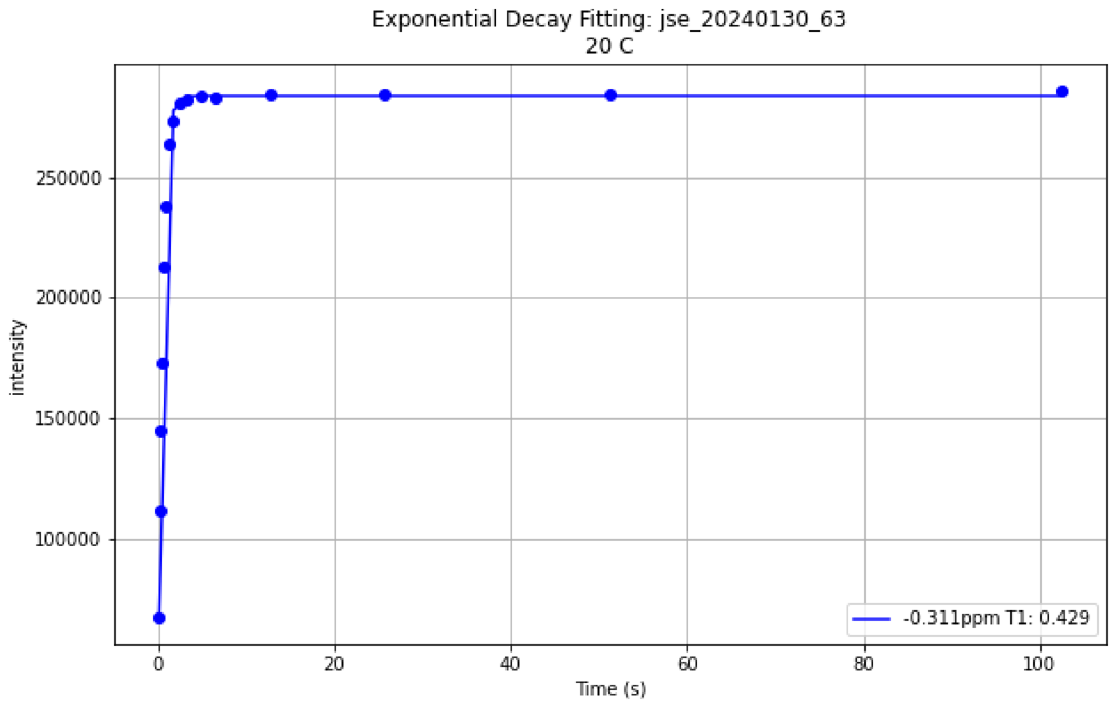

File 26: .../T1_plot/jse_T1_63.png

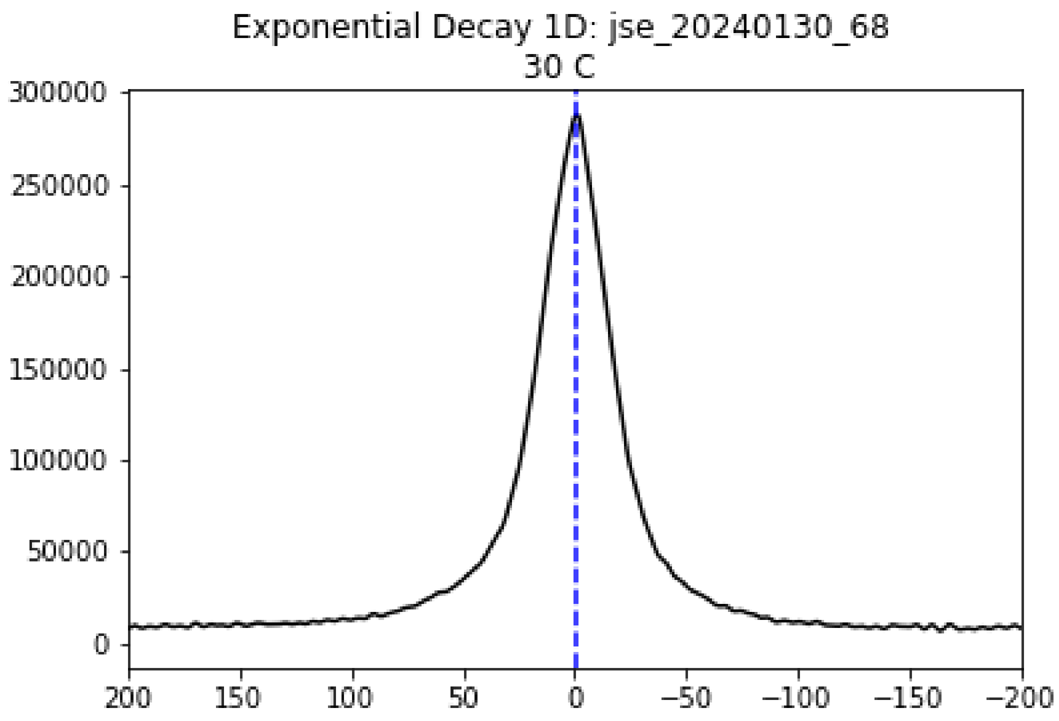

File 27: .../T1_plot/jse_20240130_68_figure.png

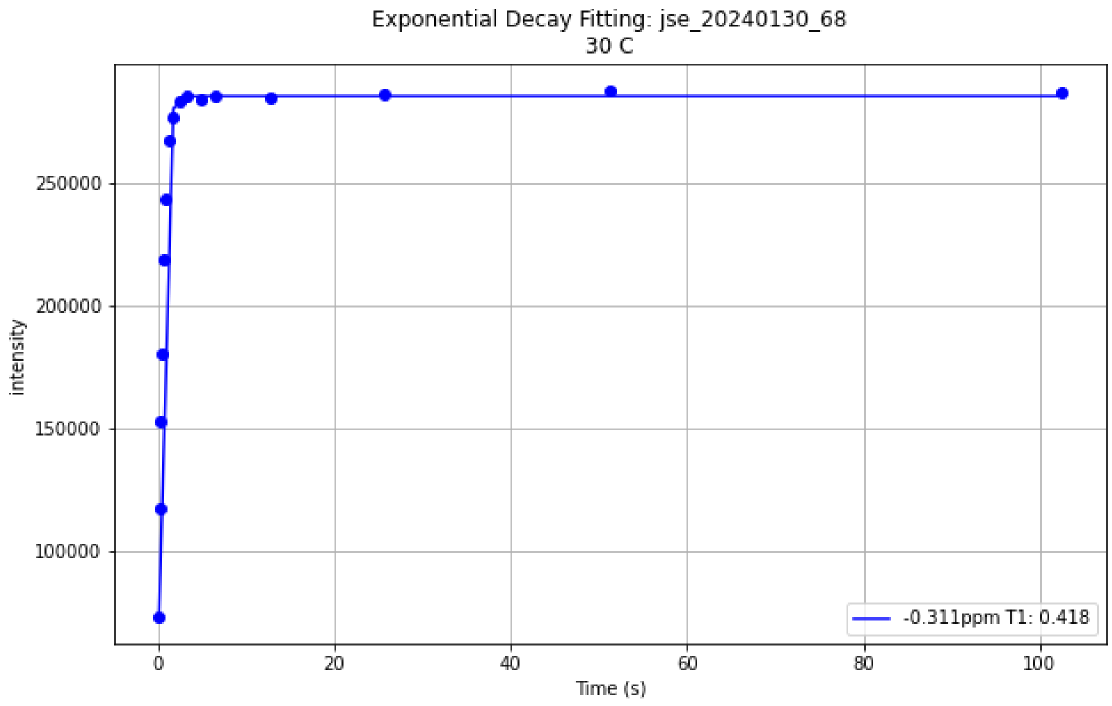

File 28: .../T1_plot/jse_T1_68.png

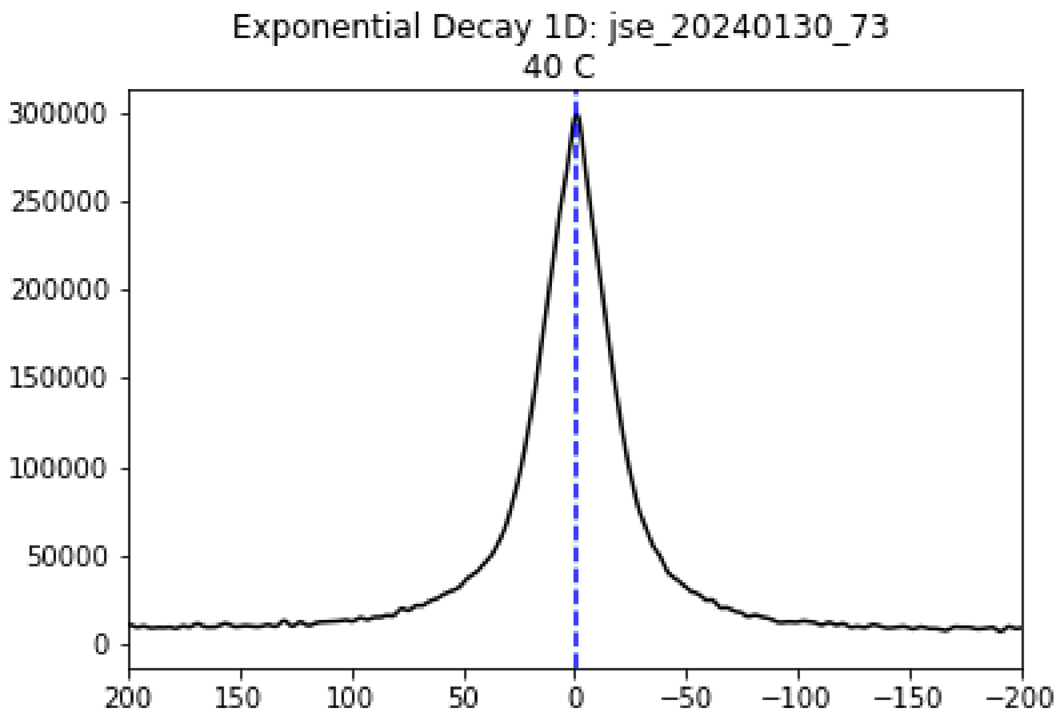

File 29: .../T1_plot/jse_20240130_73_figure.png

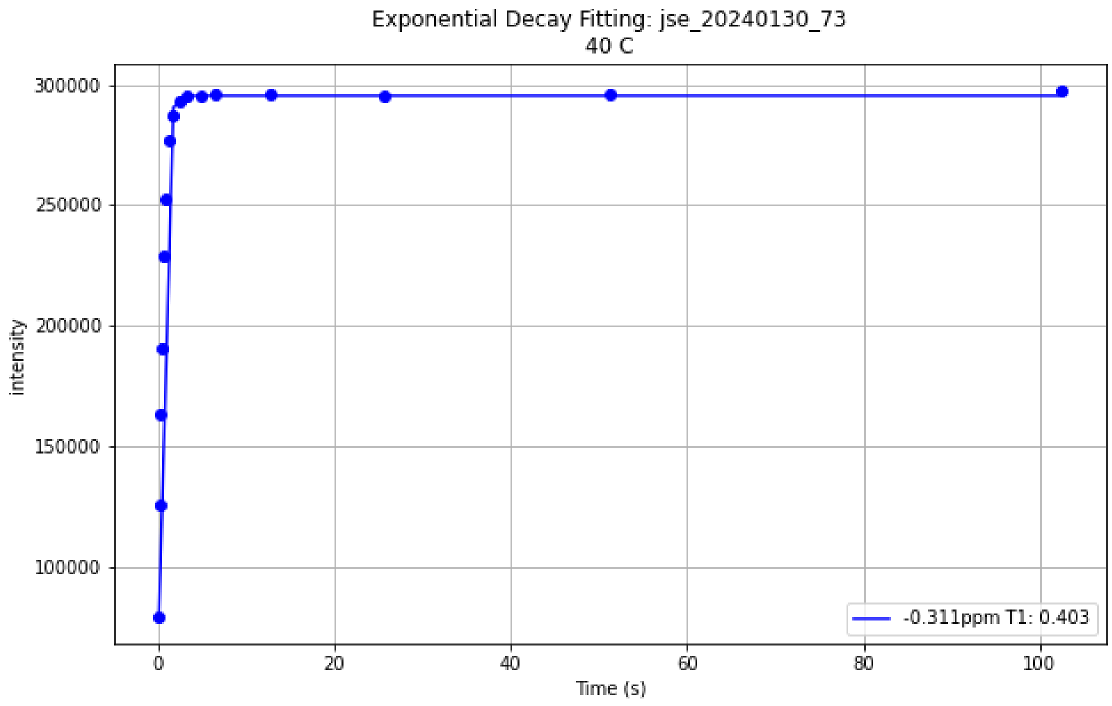

File 30: .../T1_plot/jse_T1_73.png

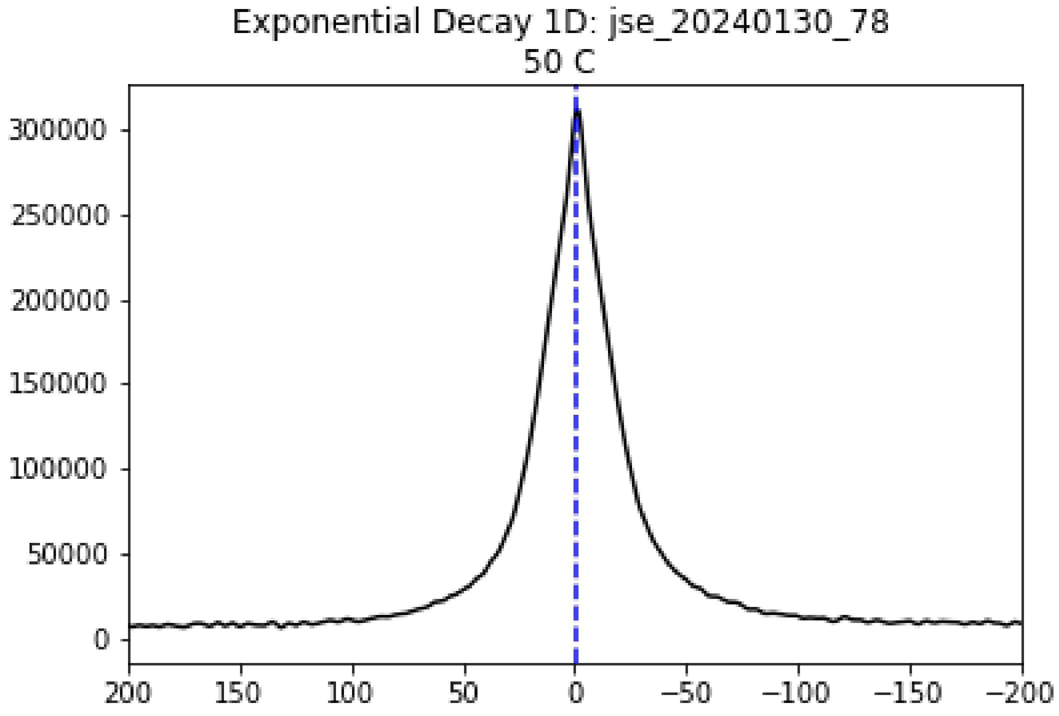

File 31: .../T1_plot/jse_20240130_78_figure.png

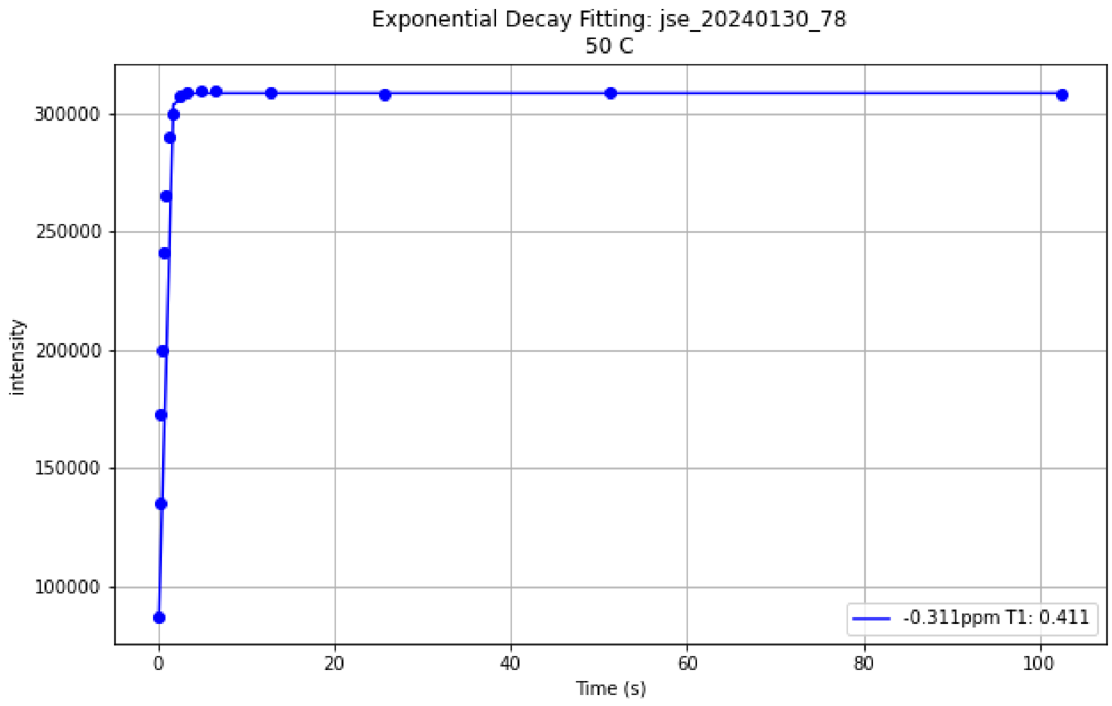

File 32: .../T1_plot/jse_T1_78.png

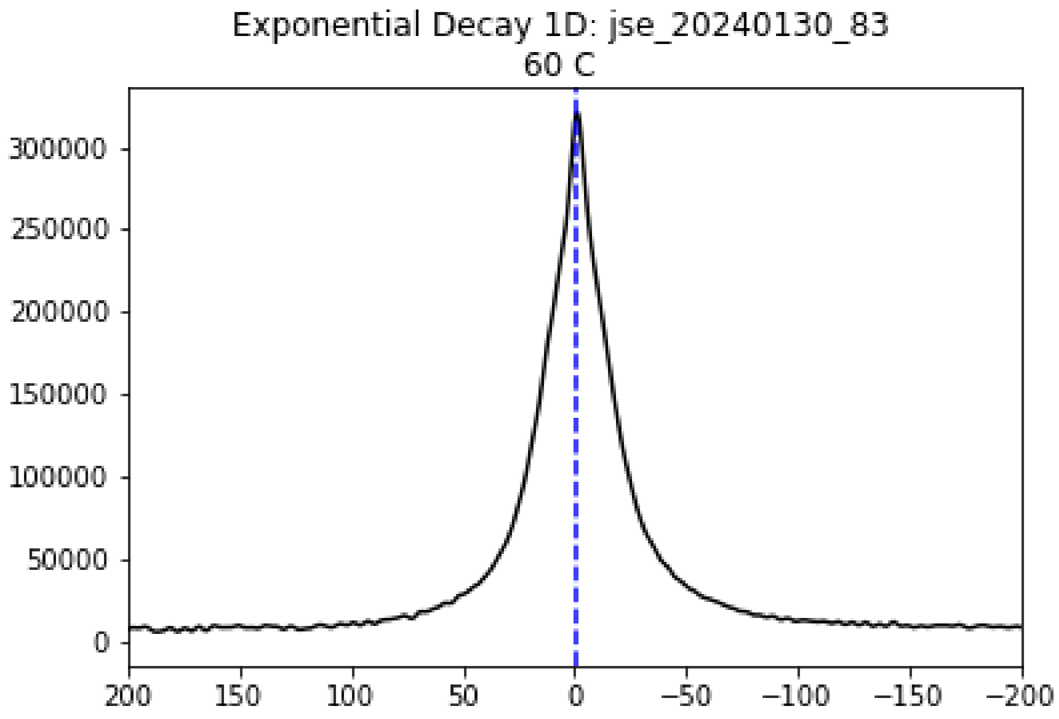

File 33: .../T1_plot/jse_20240130_83_figure.png

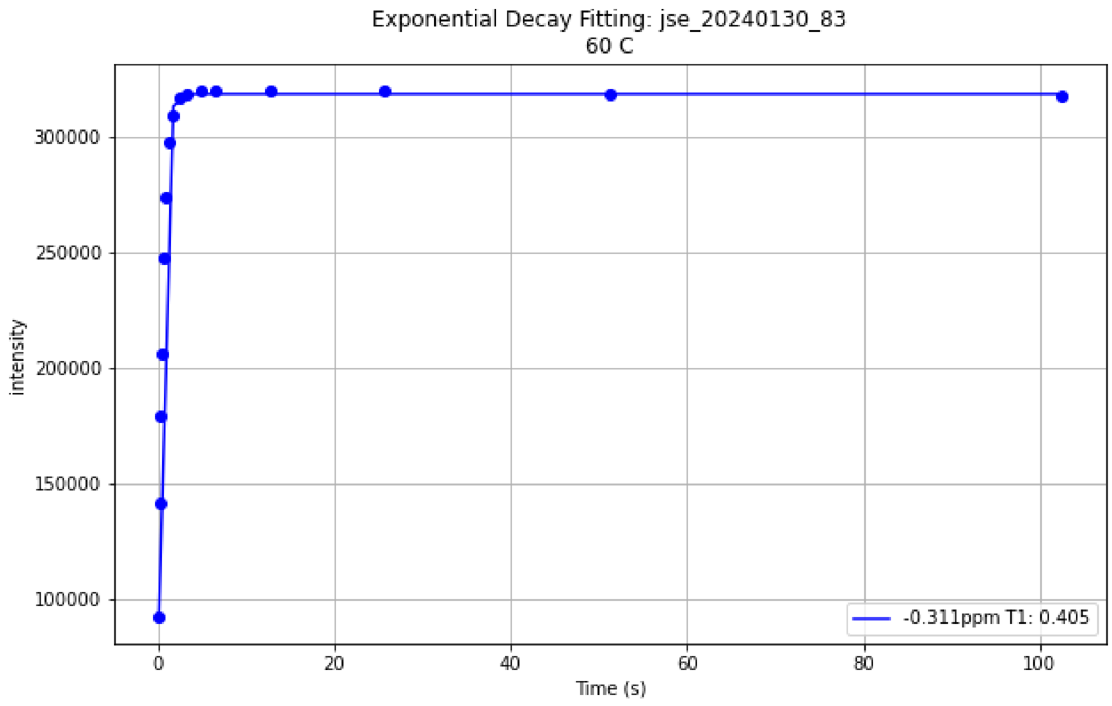

File 34: .../T1_plot/jse_T1_83.png

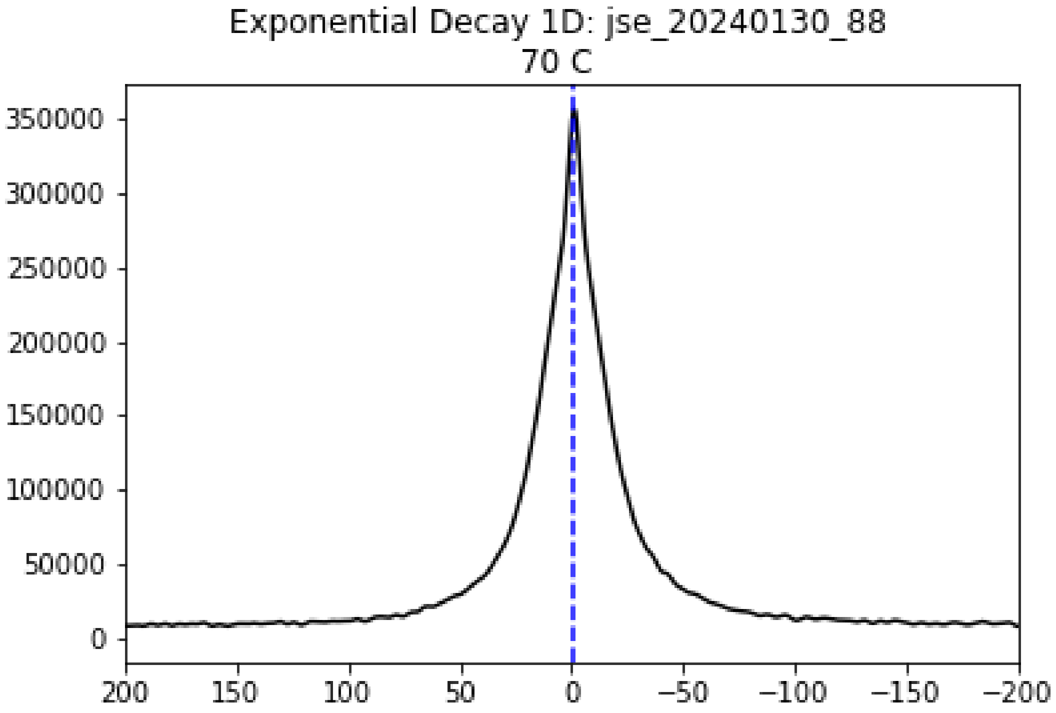

File 35: .../T1_plot/jse_20240130_88_figure.png

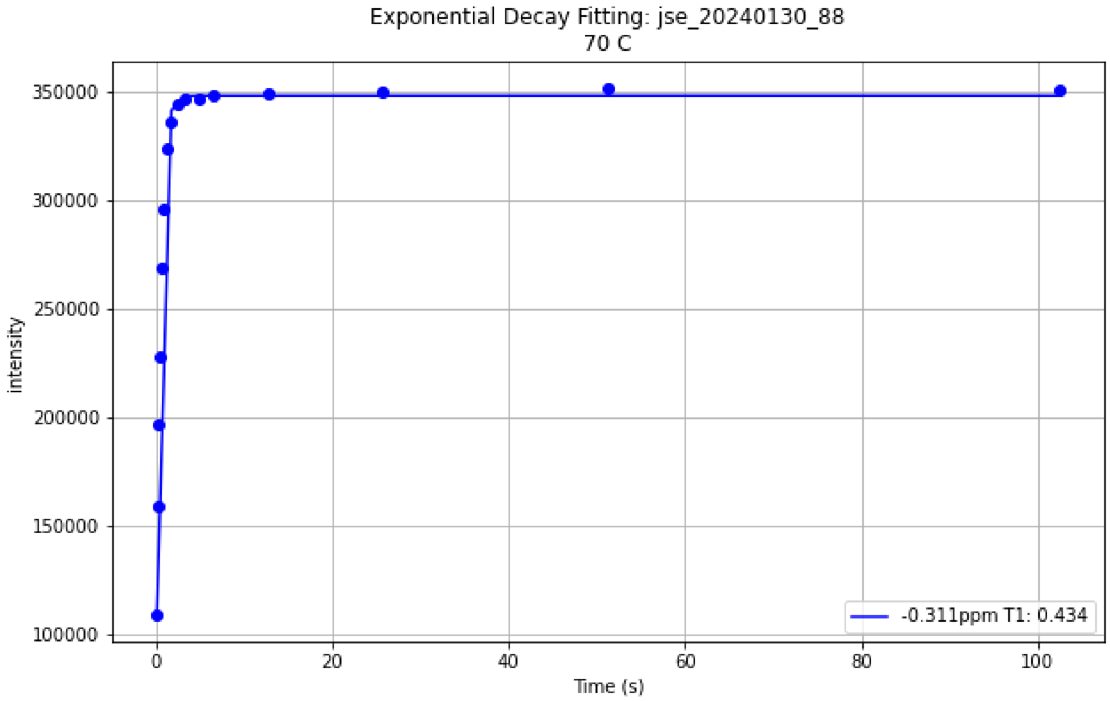

File 36: .../T1_plot/jse_T1_88.png

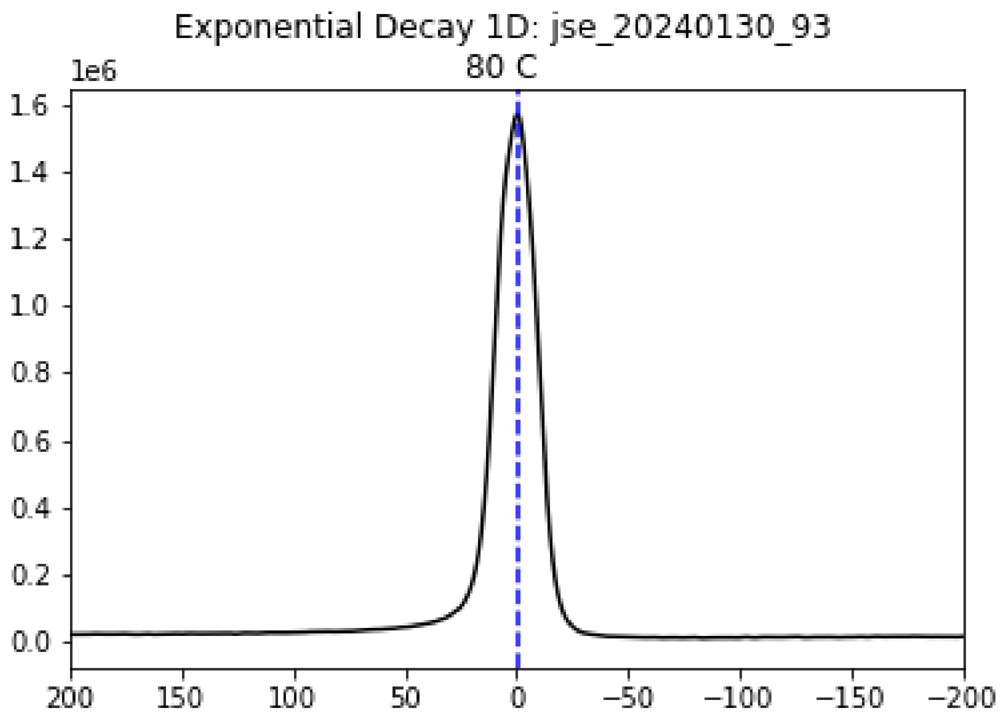

File 37: .../T1_plot/jse_20240130_93_figure.png

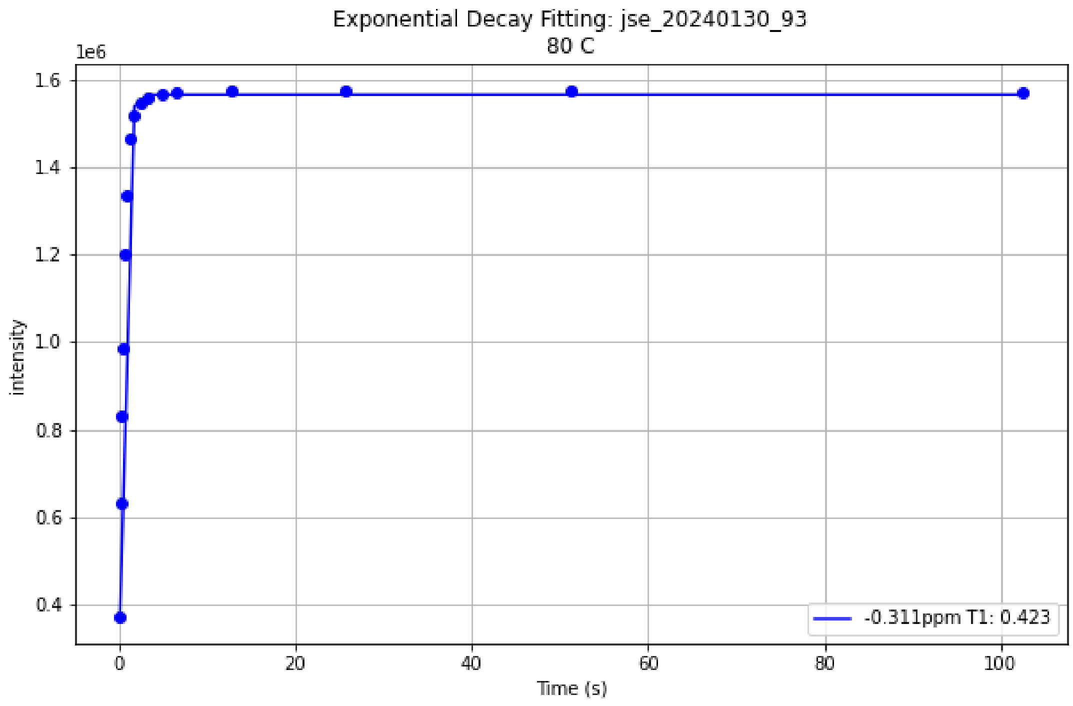

File 38: .../T1_plot/jse_T1_93.png

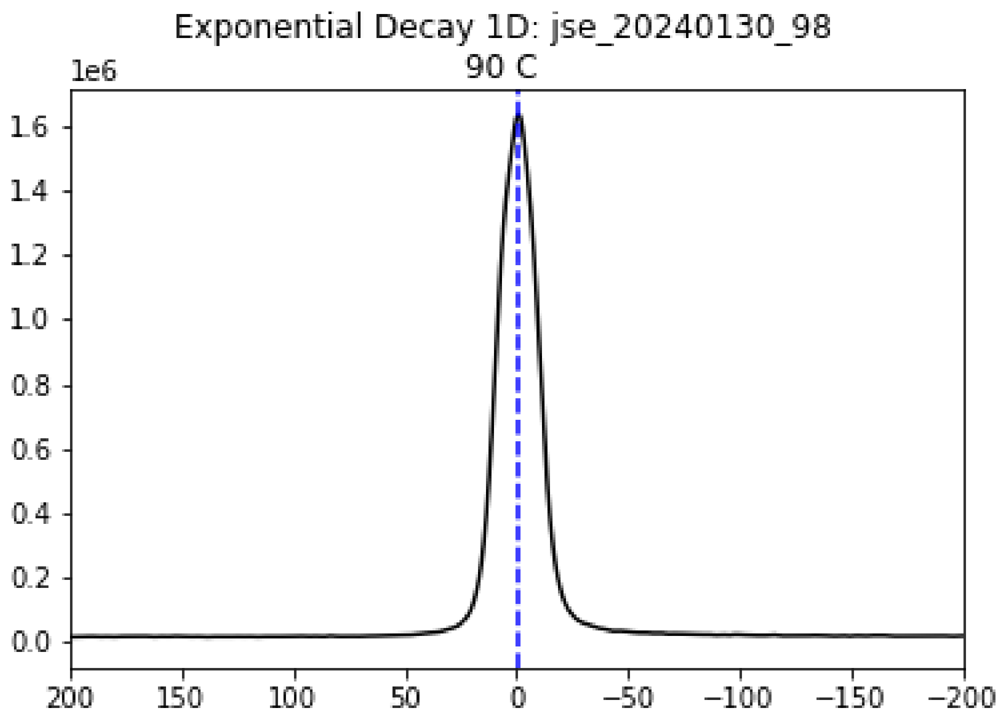

File 39: .../T1_plot/jse_20240130_98_figure.png

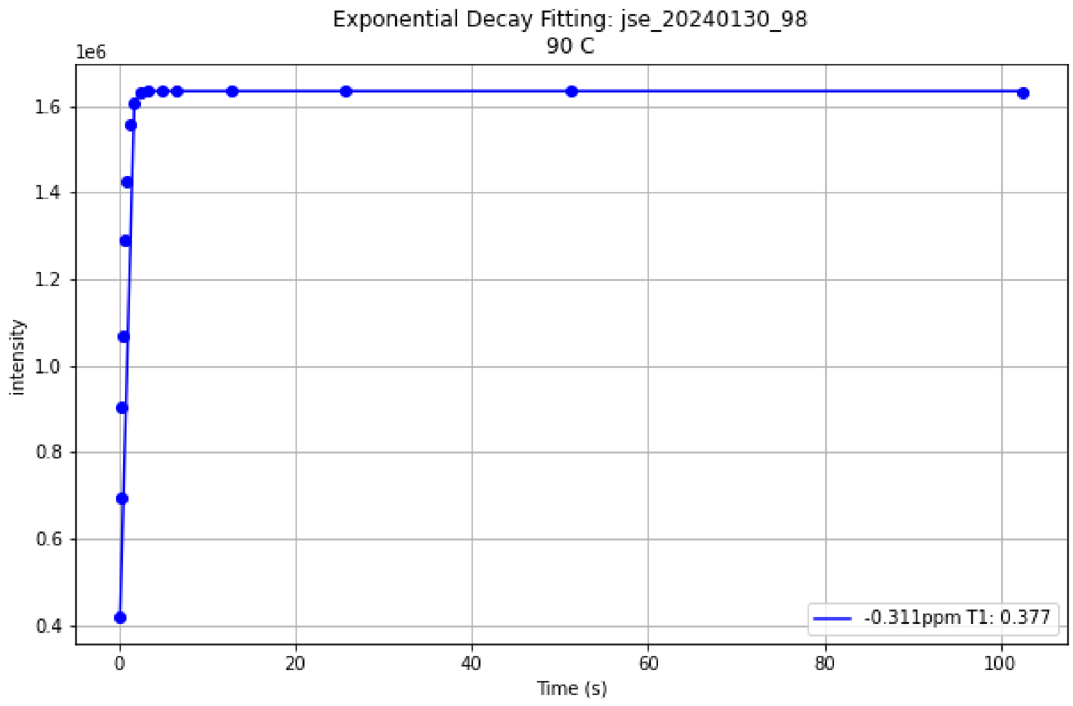

File 40: .../T1_plot/jse_T1_98.png

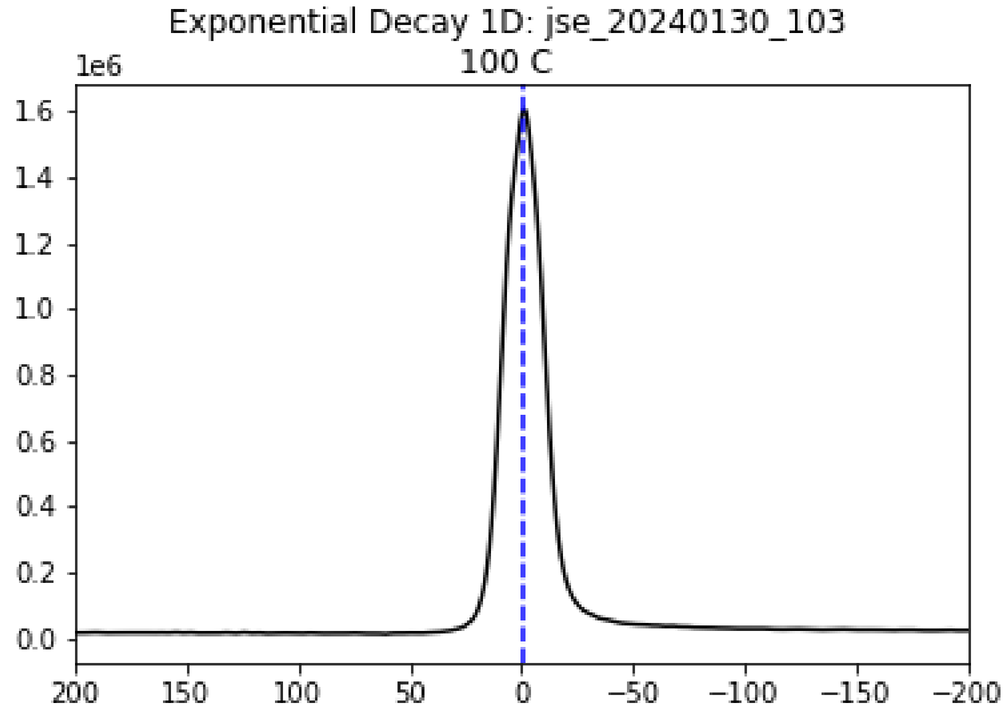

File 41: .../T1_plot/jse_20240130_103_figure.png

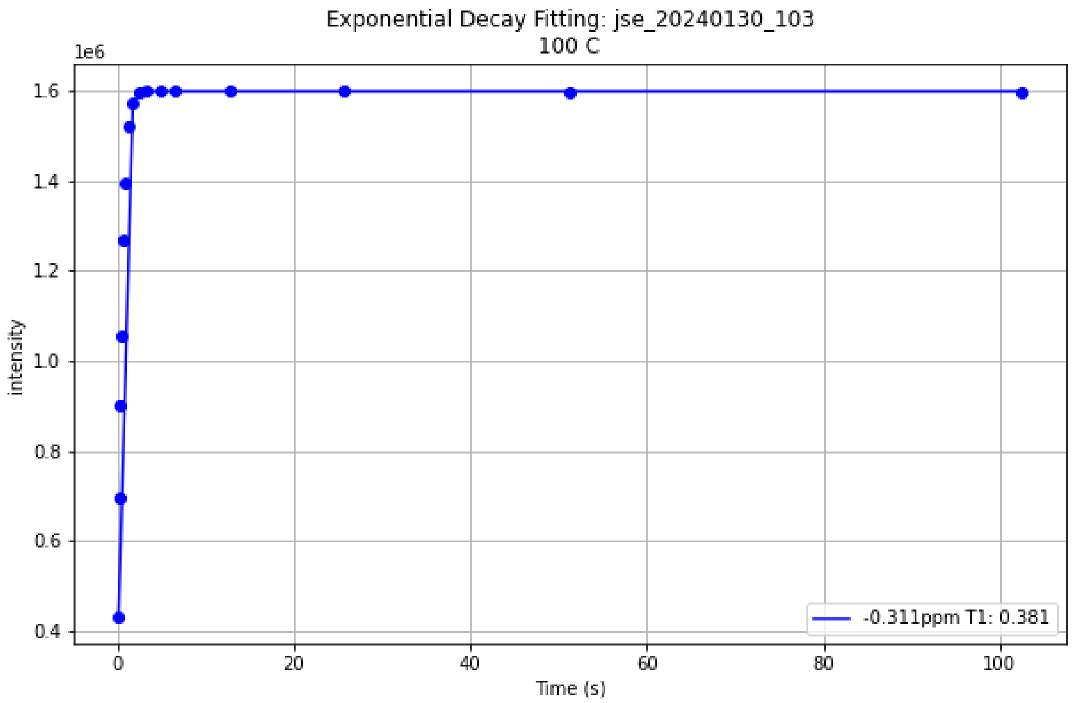

File 42: .../T1_plot/jse_T1_103.png

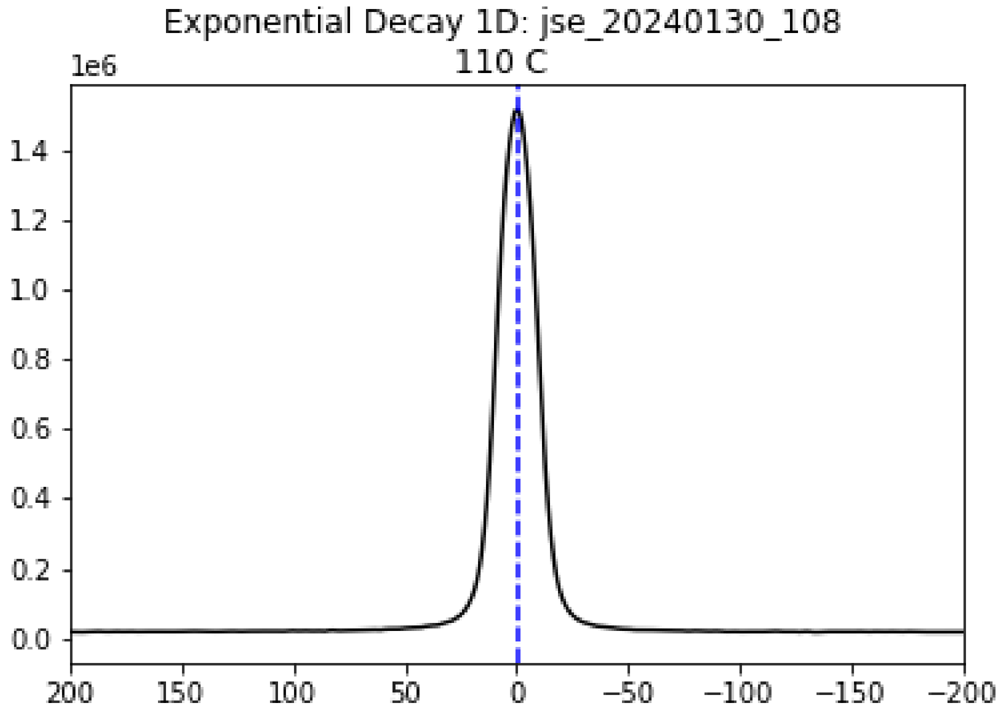

File 43: .../T1_plot/jse_20240130_108_figure.png

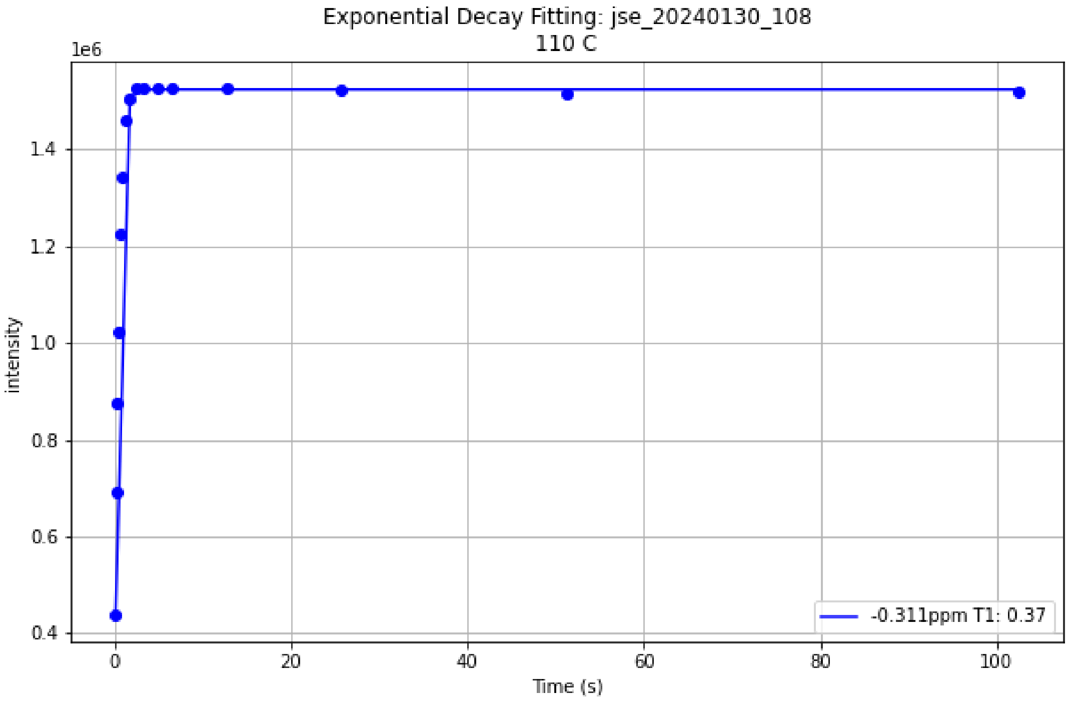

File 44: .../T1_plot/jse_T1_108.png

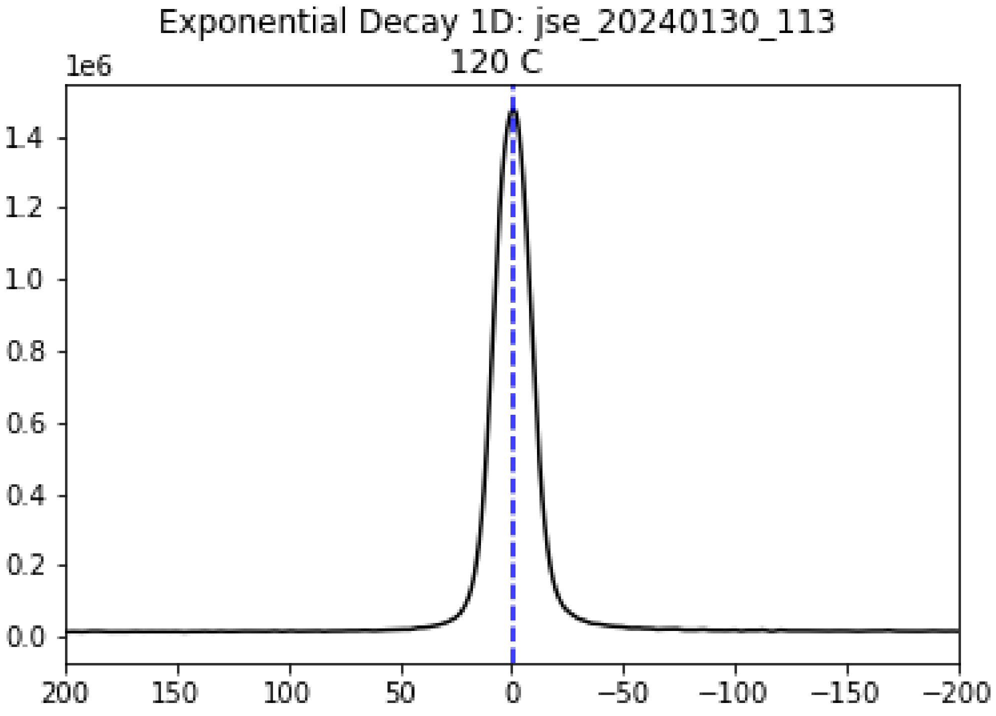

File 45: .../T1_plot/jse_20240130_113_figure.png

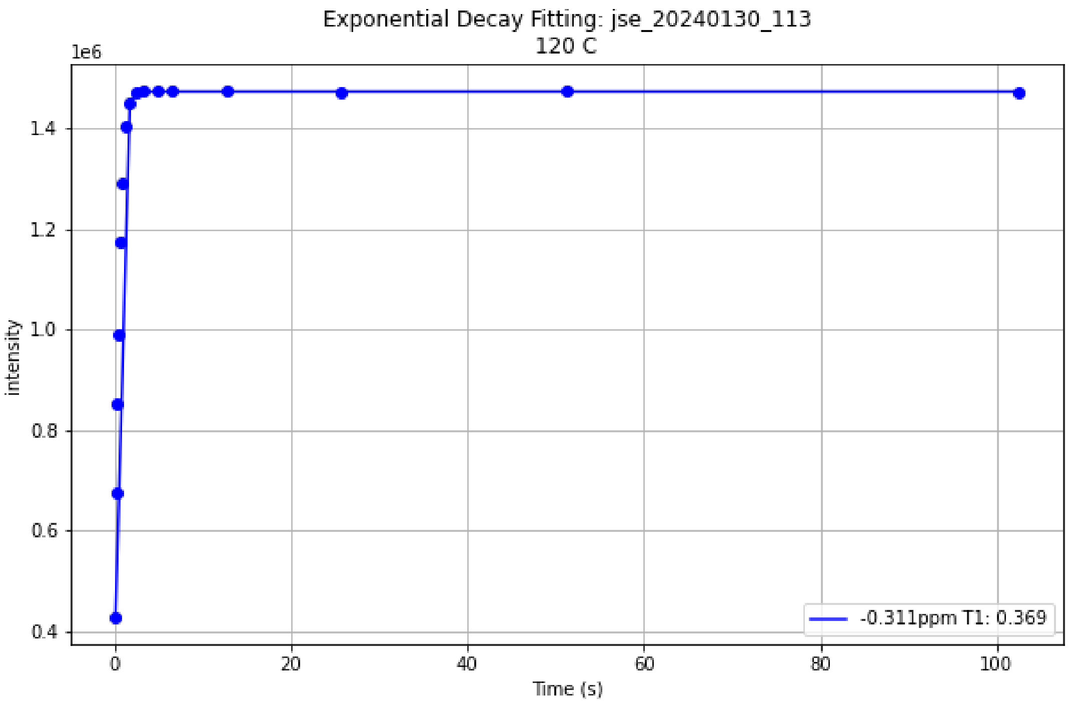

File 46: .../T1_plot/jse_T1_113.png

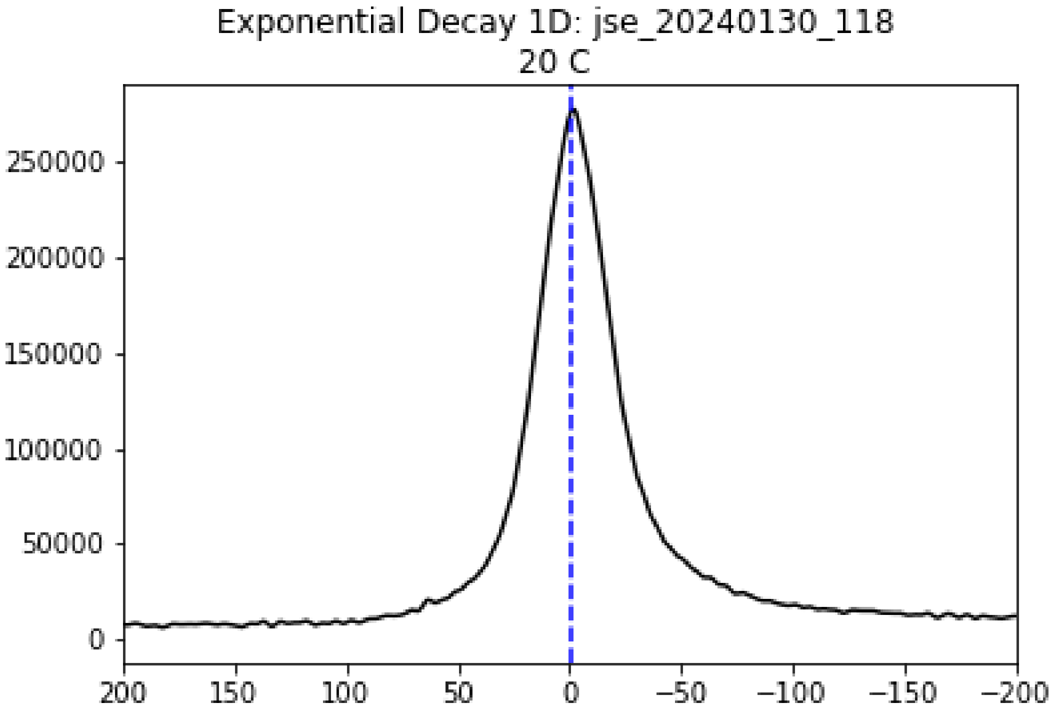

File 47: .../T1_plot/jse_20240130_118_figure.png

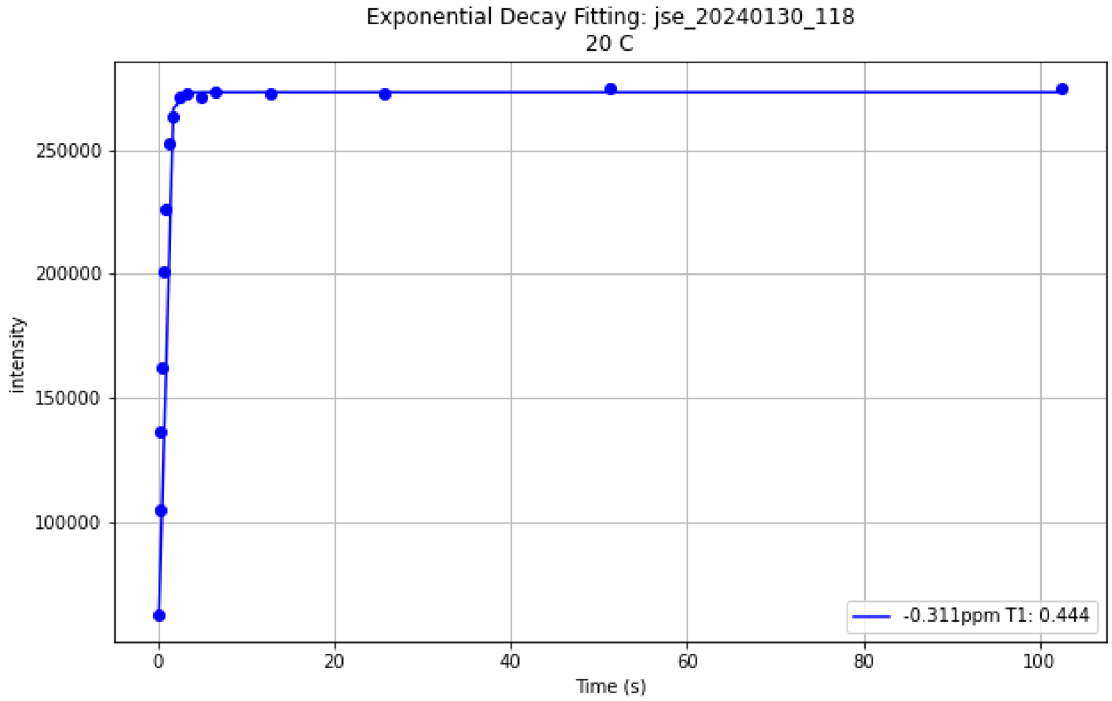

File 48: .../T1_plot/jse_T1_118.png
