# Supplementary material for: Potential Multiaxial Molecular Ferroelectricity through Chiral Cation Replacement
Source: Cryst Growth Des. 2025 Jul 21;25(15):6237–47. doi: 10.1021/acs.cgd.5c00666 (PMC12332970; doi:10.1021/acs.cgd.5c00666)
Supplement: Supplementary file 2 [file cg5c00666_si_002.zip › NMR/jse_20240130_4_to_119_T1RHO_output.pdf]

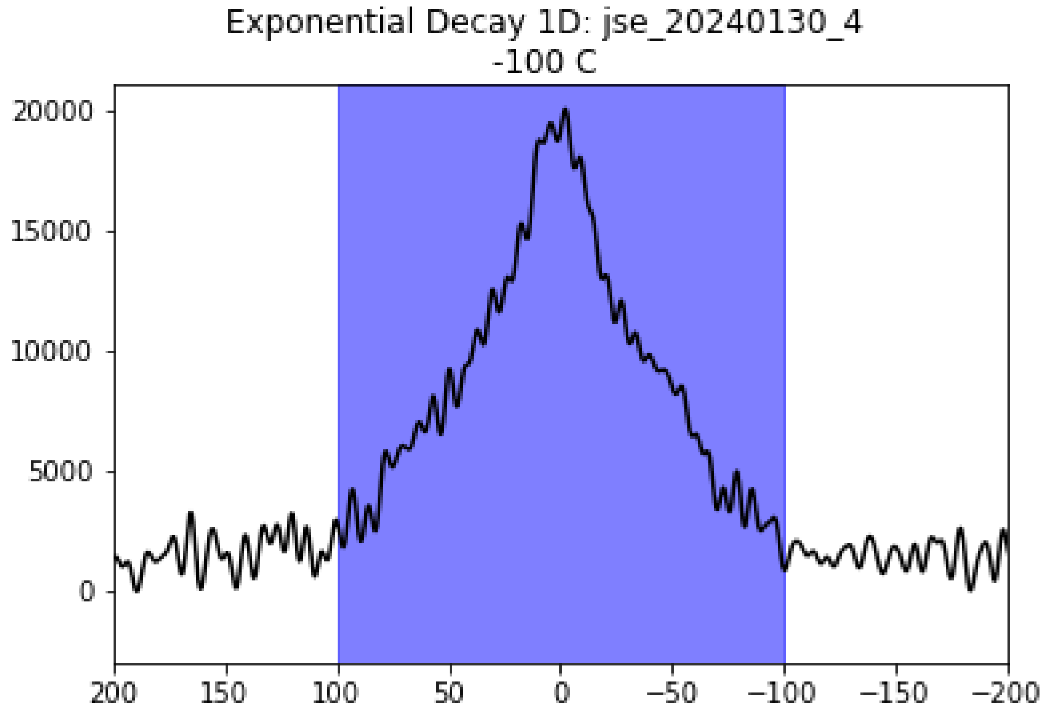

File 1: .../T1RHO_plot/jse_20240130_4_figure.png

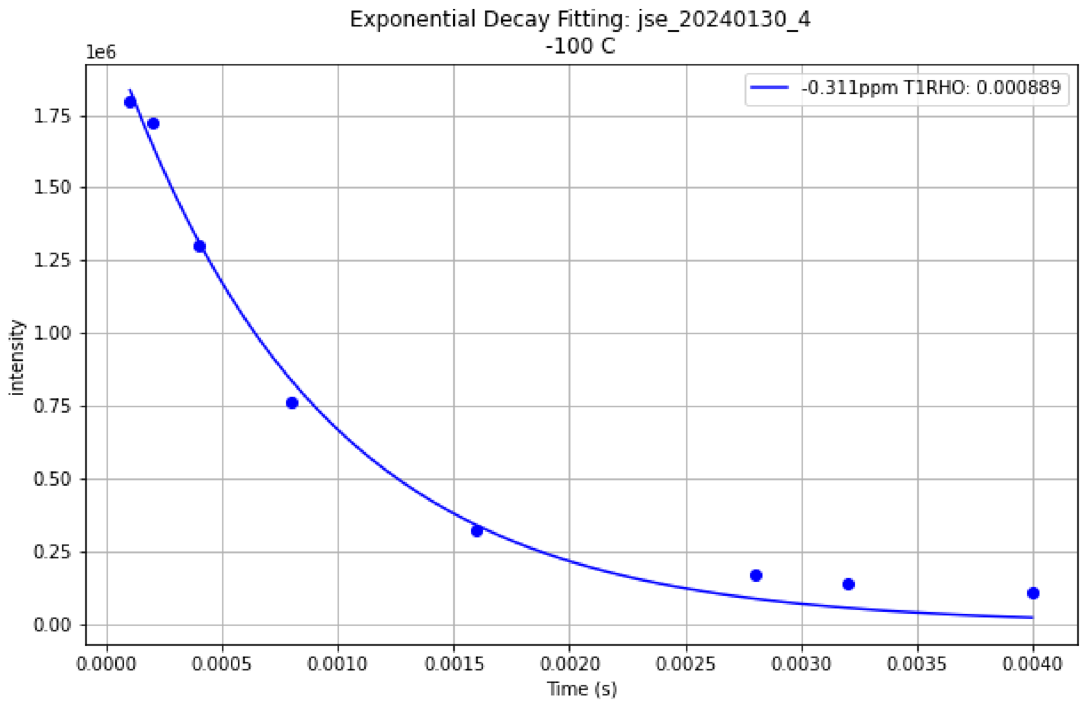

File 2: .../T1RHO_plot/jse_T1RHO_4.png

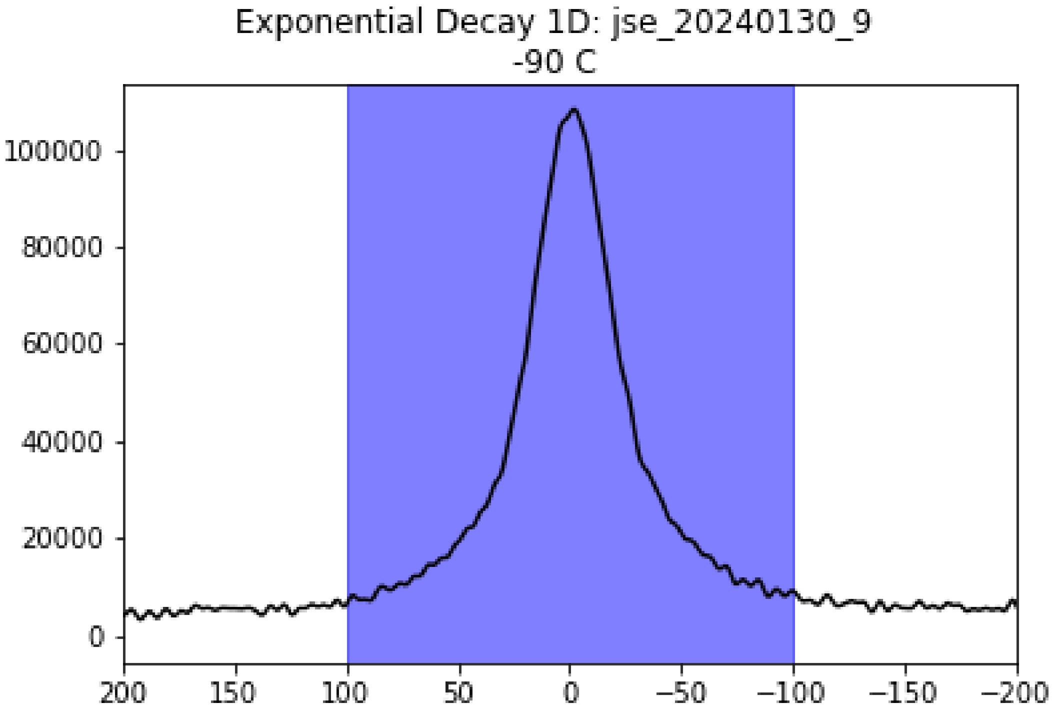

File 3: .../T1RHO_plot/jse_20240130_9_figure.png

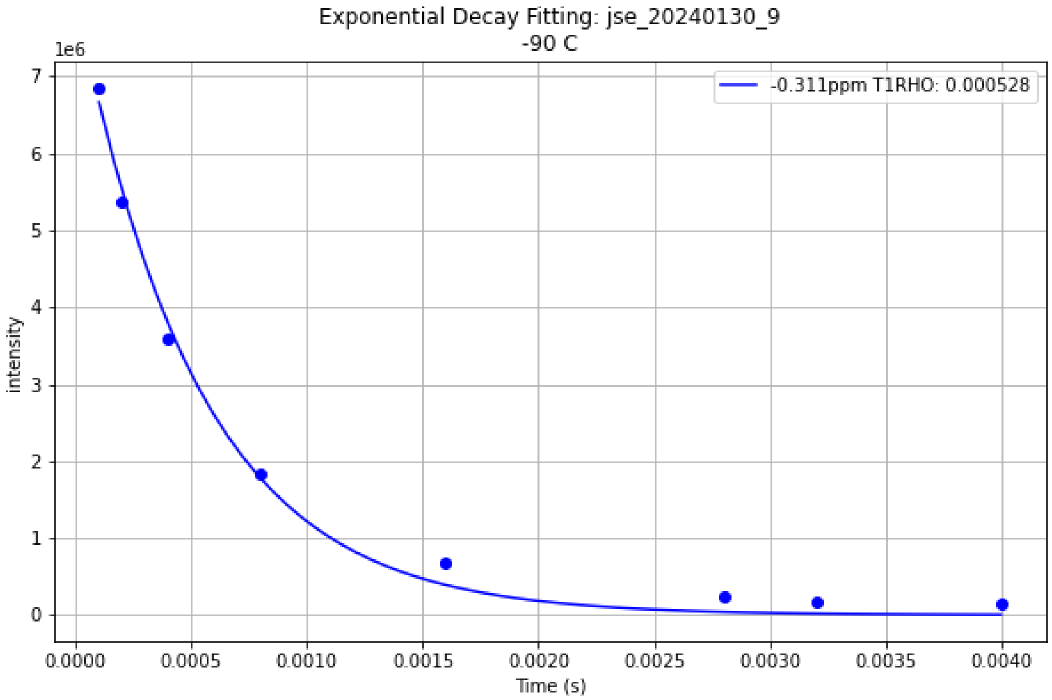

File 4: .../T1RHO_plot/jse_T1RHO_9.png

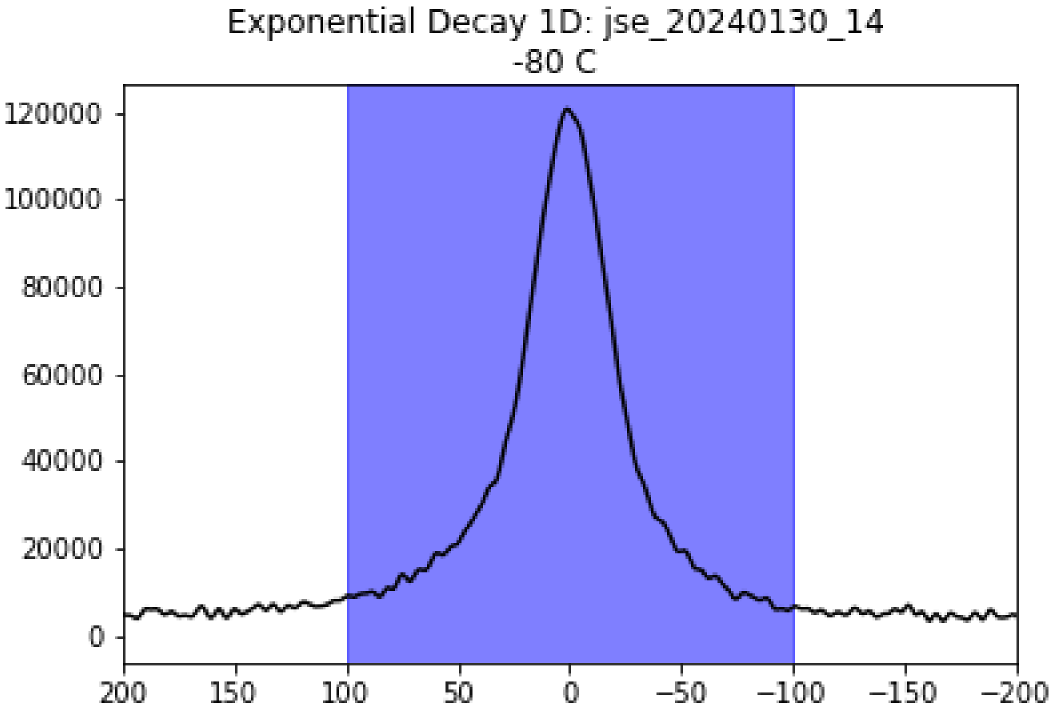

File 5: .../T1RHO_plot/jse_20240130_14_figure.png

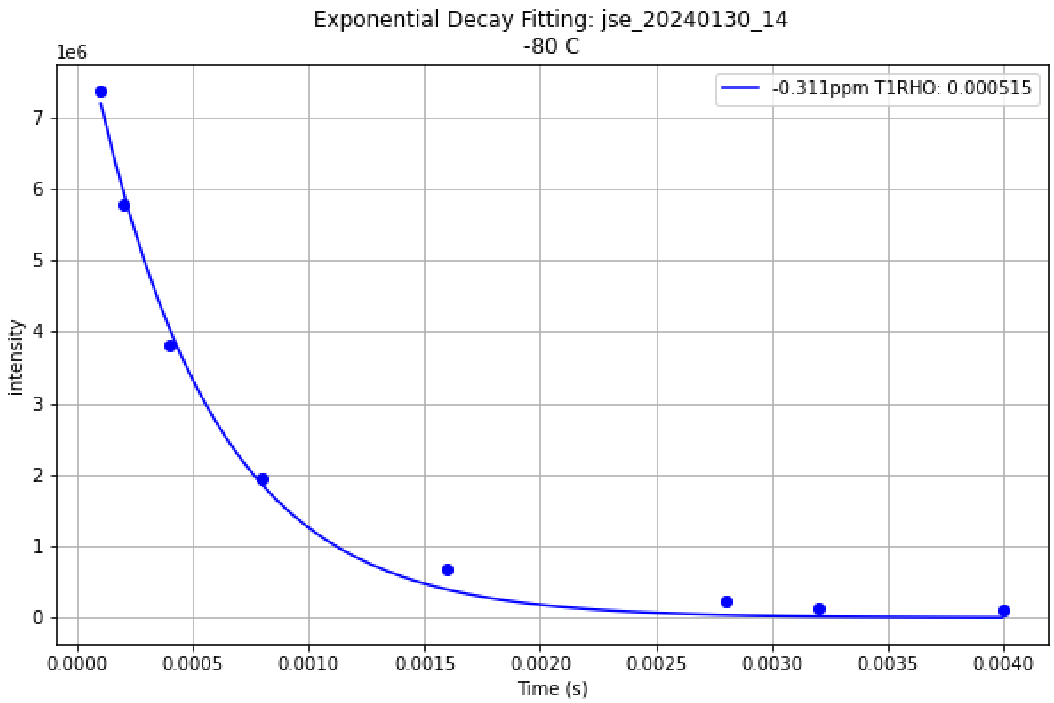

File 6: .../T1RHO_plot/jse_T1RHO_14.png

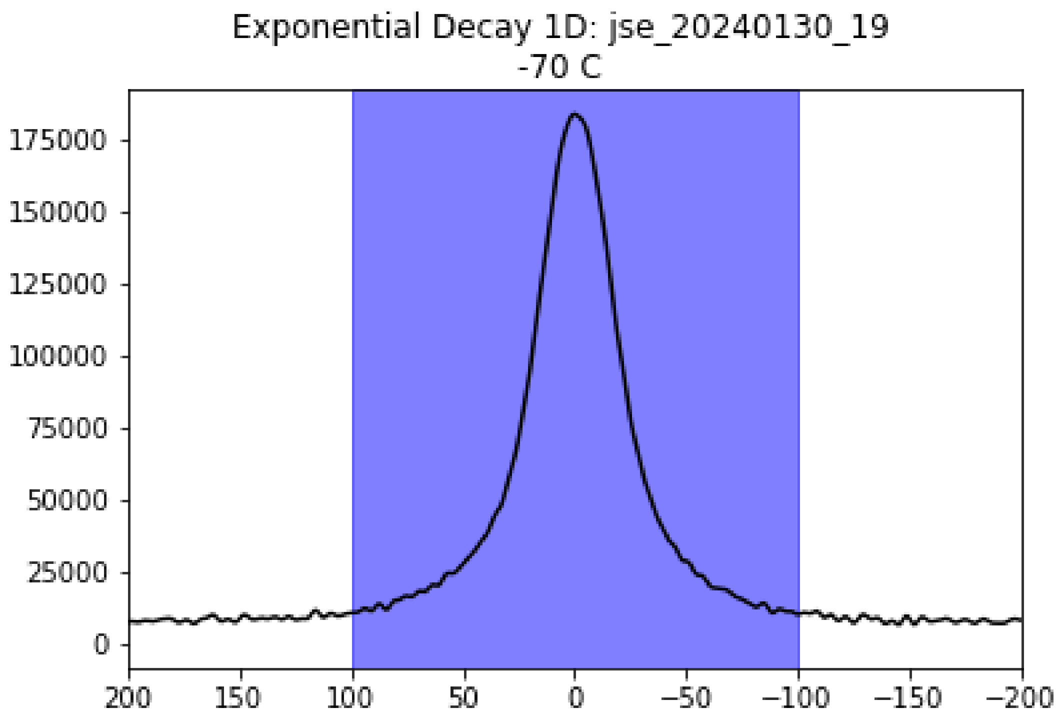

File 7: .../T1RHO_plot/jse_20240130_19_figure.png

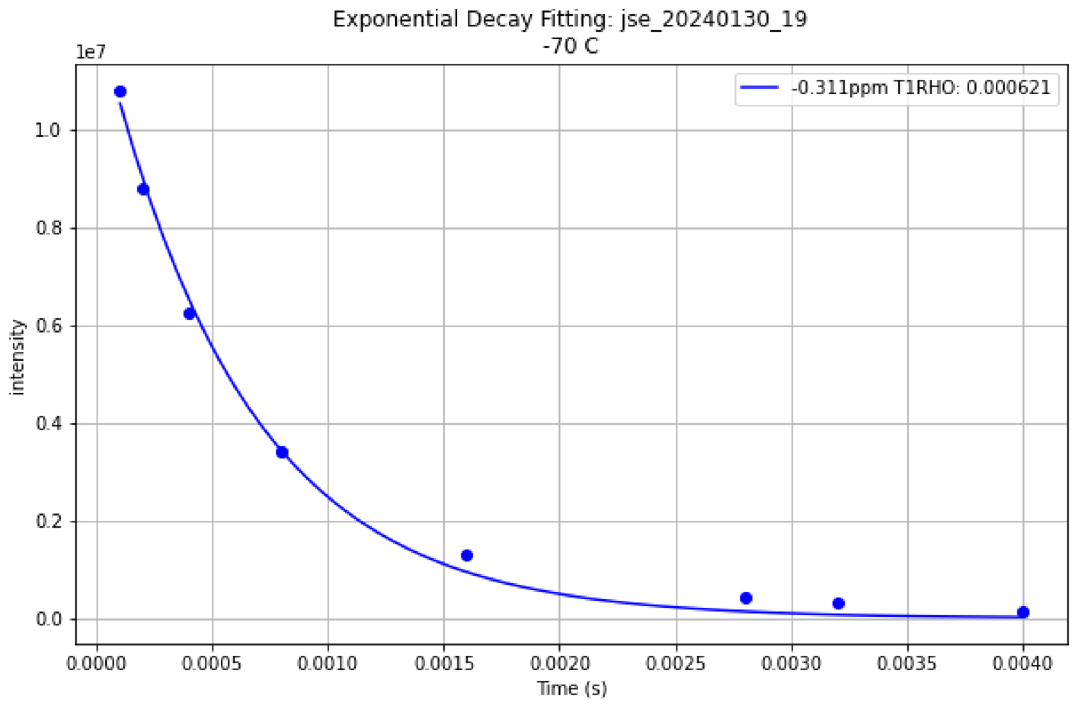

File 8: .../T1RHO_plot/jse_T1RHO_19.png

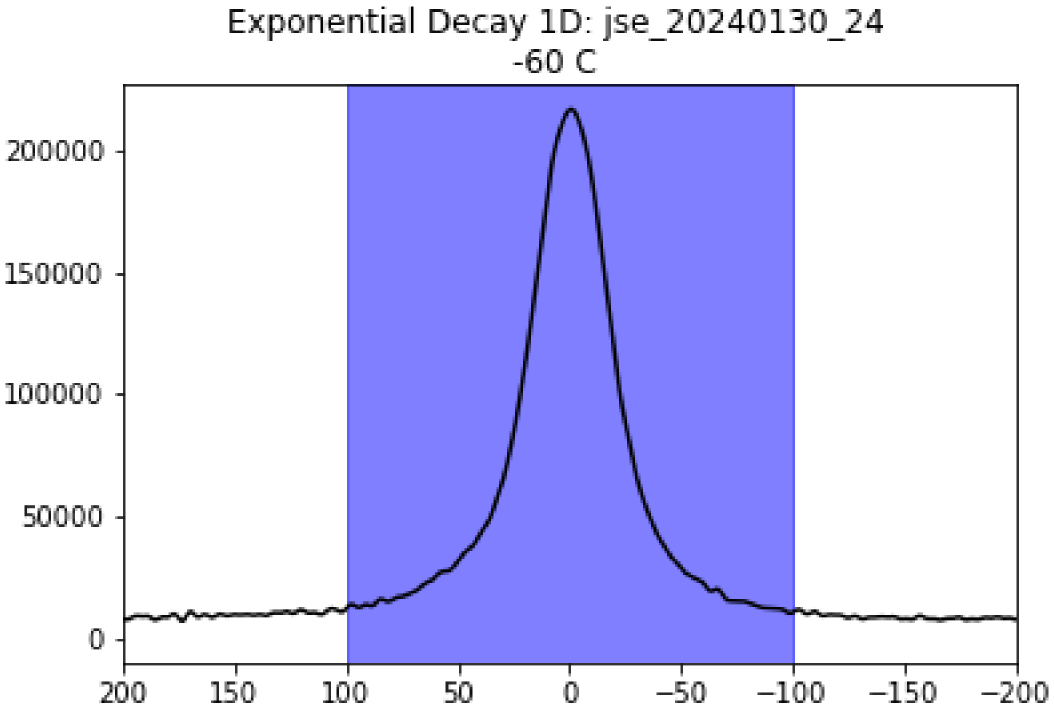

File 9: .../T1RHO_plot/jse_20240130_24_figure.png

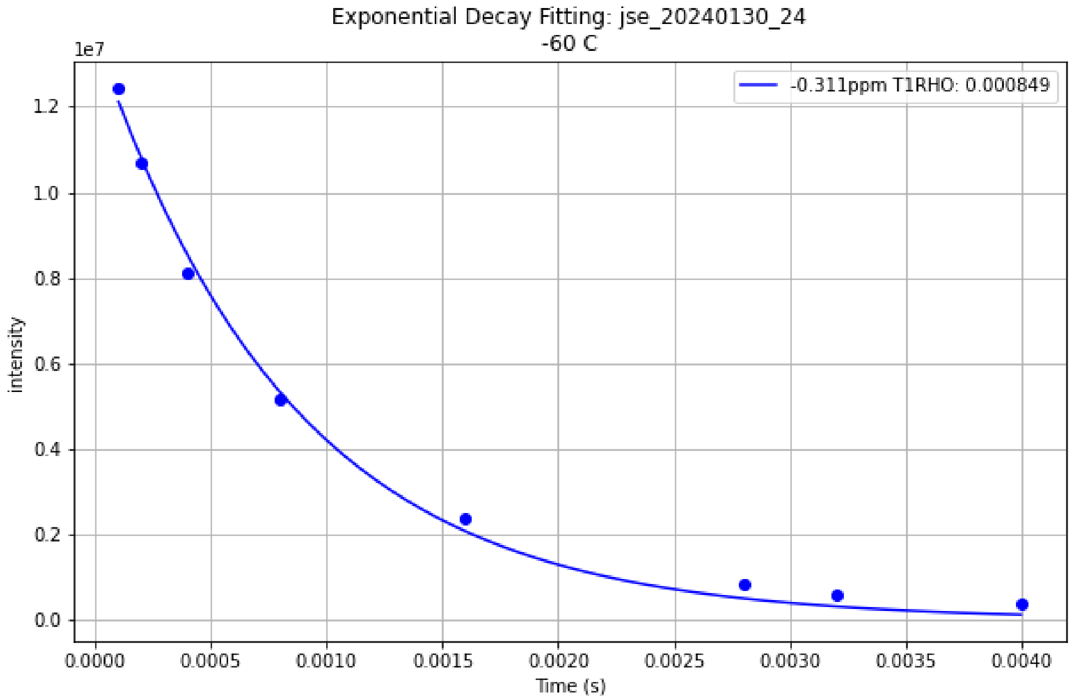

File 10: .../T1RHO_plot/jse_T1RHO_24.png

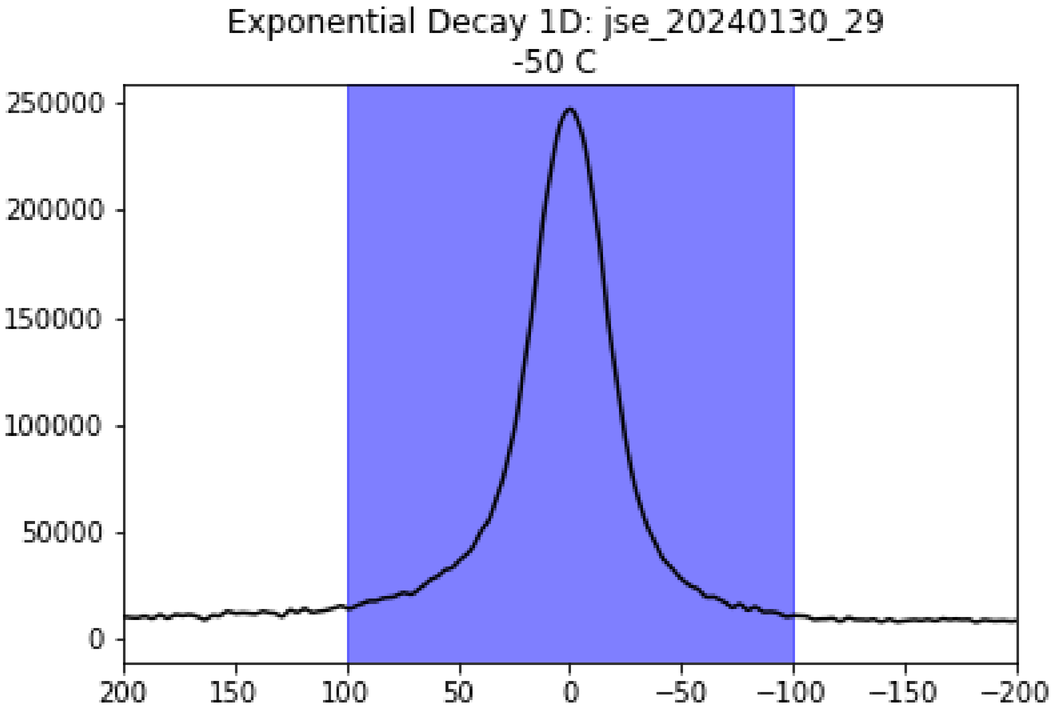

File 11: .../T1RHO_plot/jse_20240130_29_figure.png

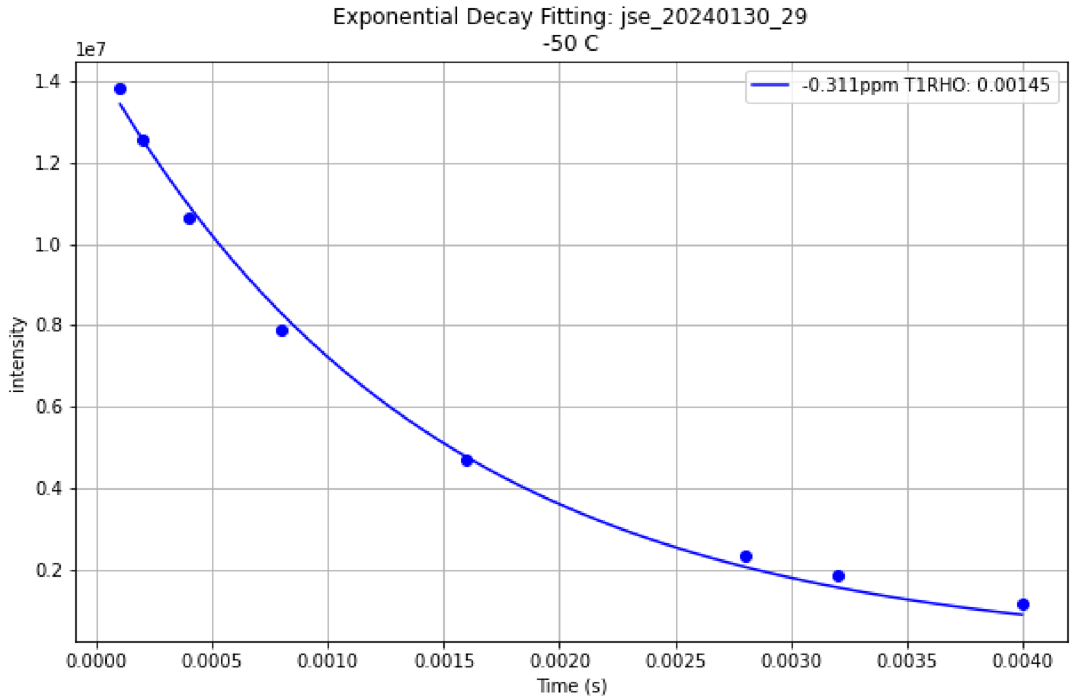

File 12: .../T1RHO_plot/jse_T1RHO_29.png

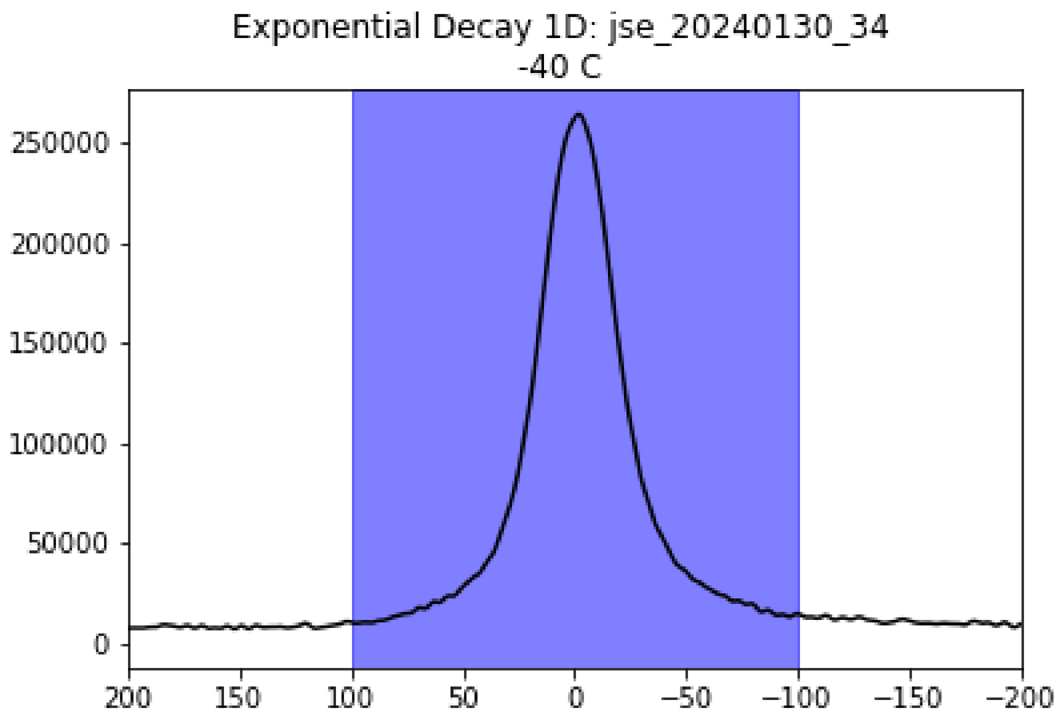

File 13: .../T1RHO_plot/jse_20240130_34_figure.png

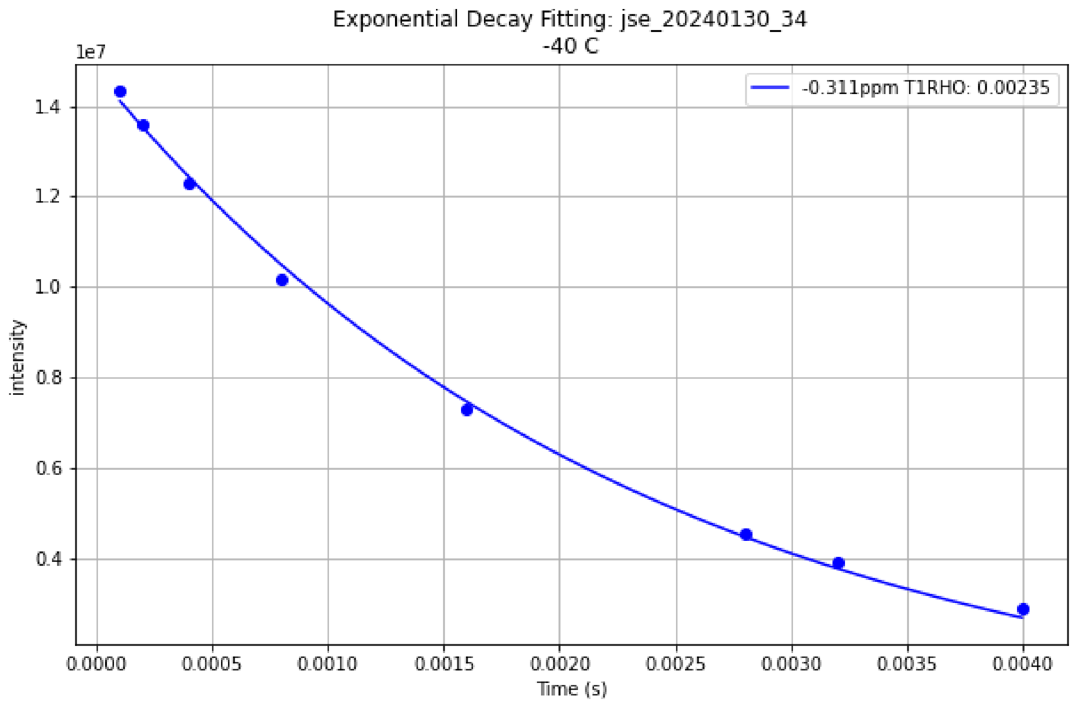

File 14: .../T1RHO_plot/jse_T1RHO_34.png

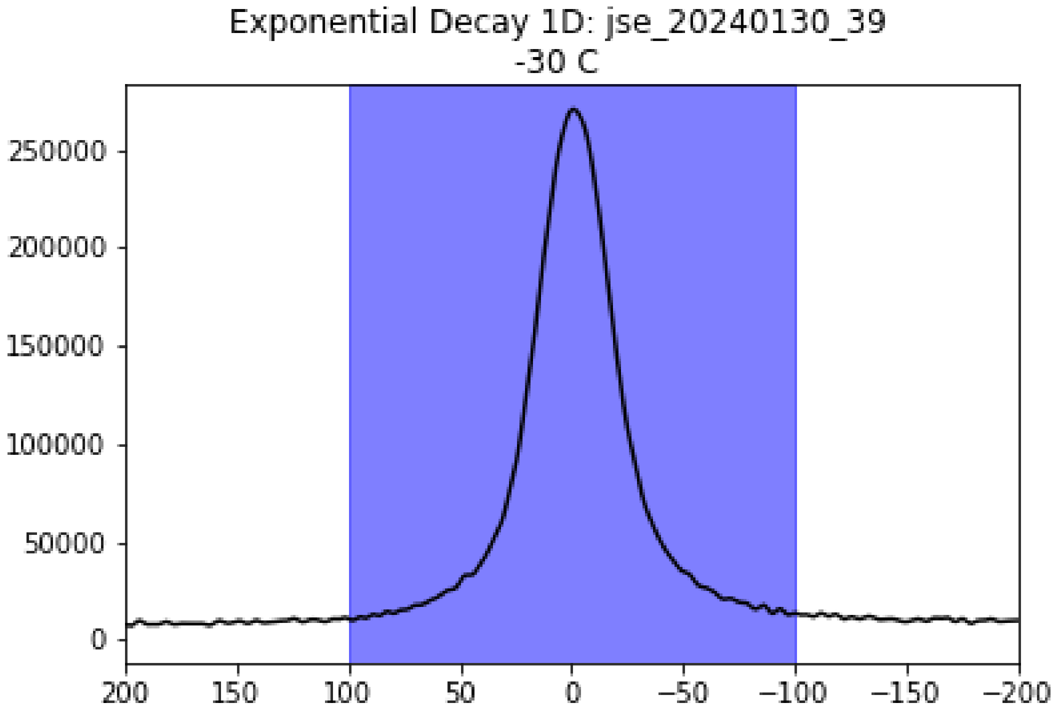

File 15: .../T1RHO_plot/jse_20240130_39_figure.png

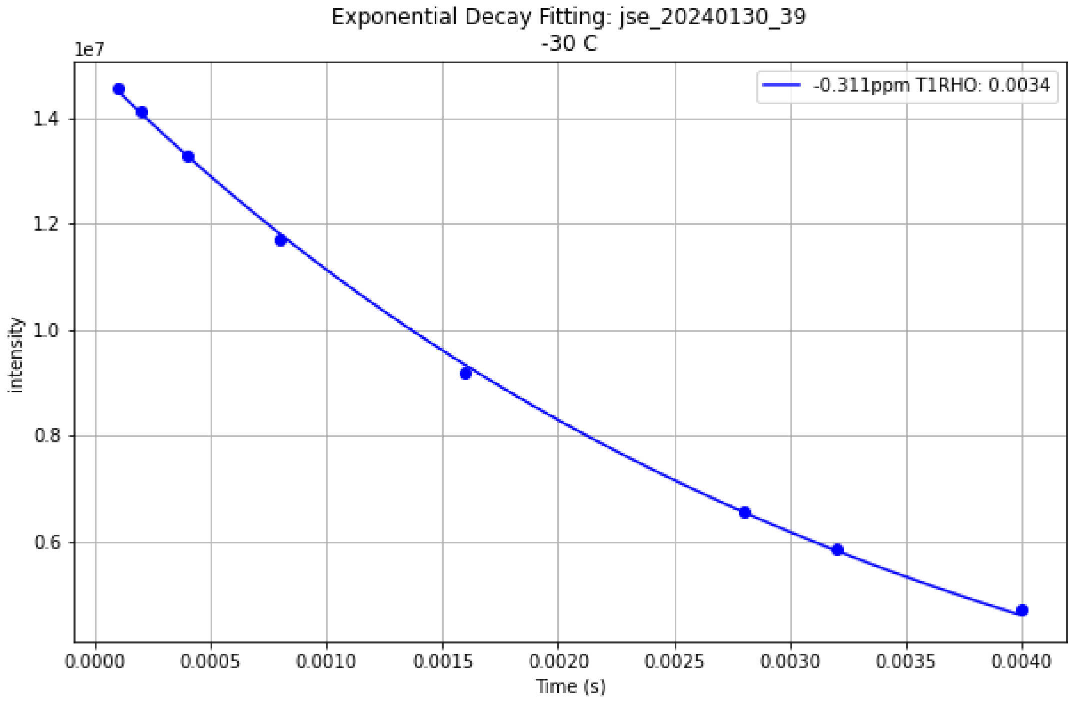

File 16: .../T1RHO_plot/jse_T1RHO_39.png

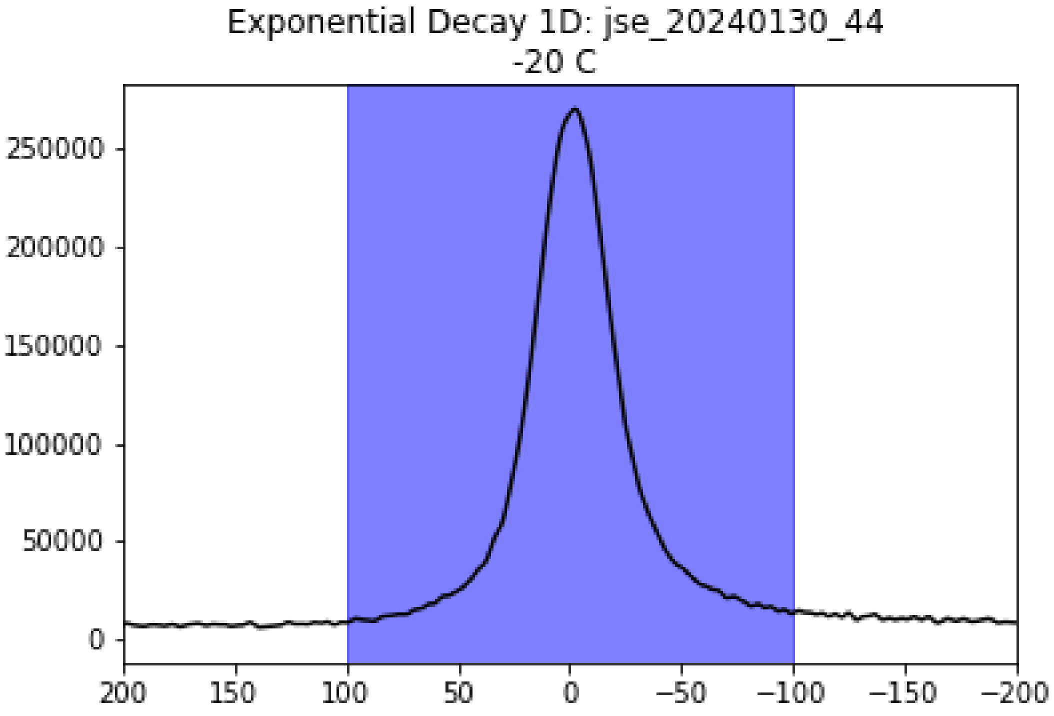

File 17: .../T1RHO_plot/jse_20240130_44_figure.png

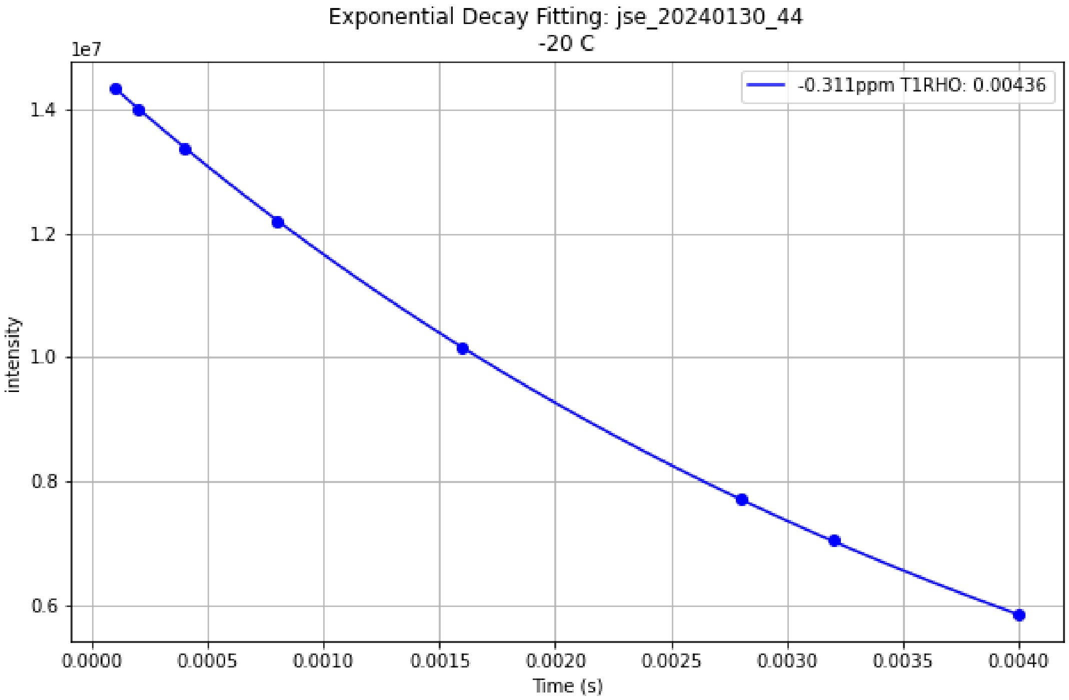

File 18: .../T1RHO_plot/jse_T1RHO_44.png

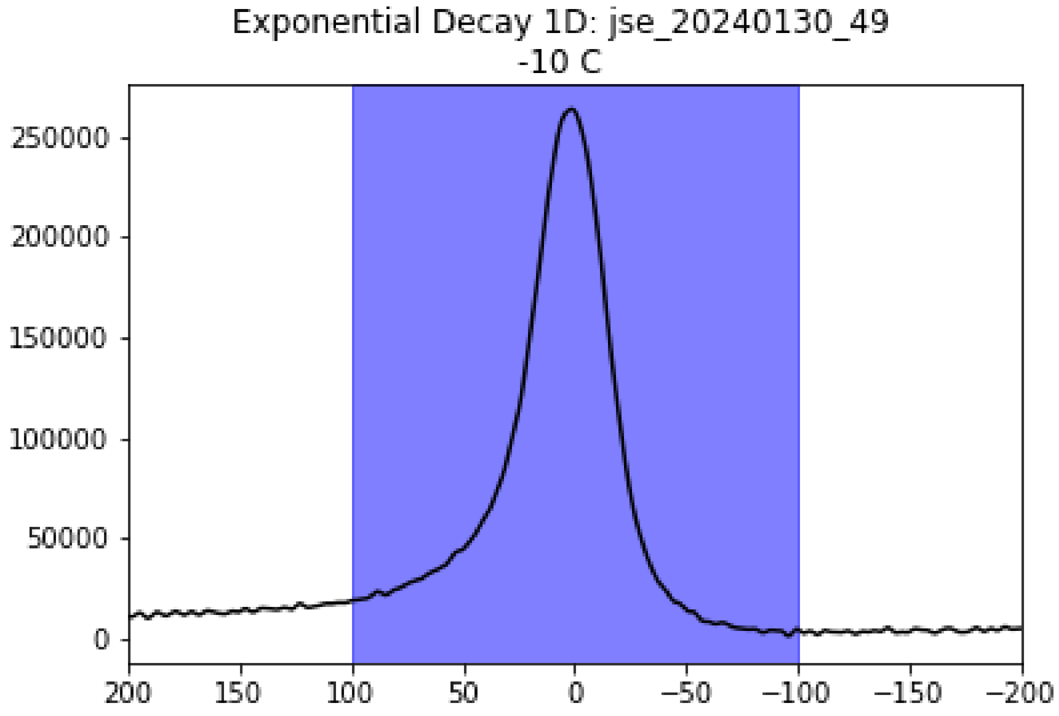

File 19: .../T1RHO_plot/jse_20240130_49_figure.png

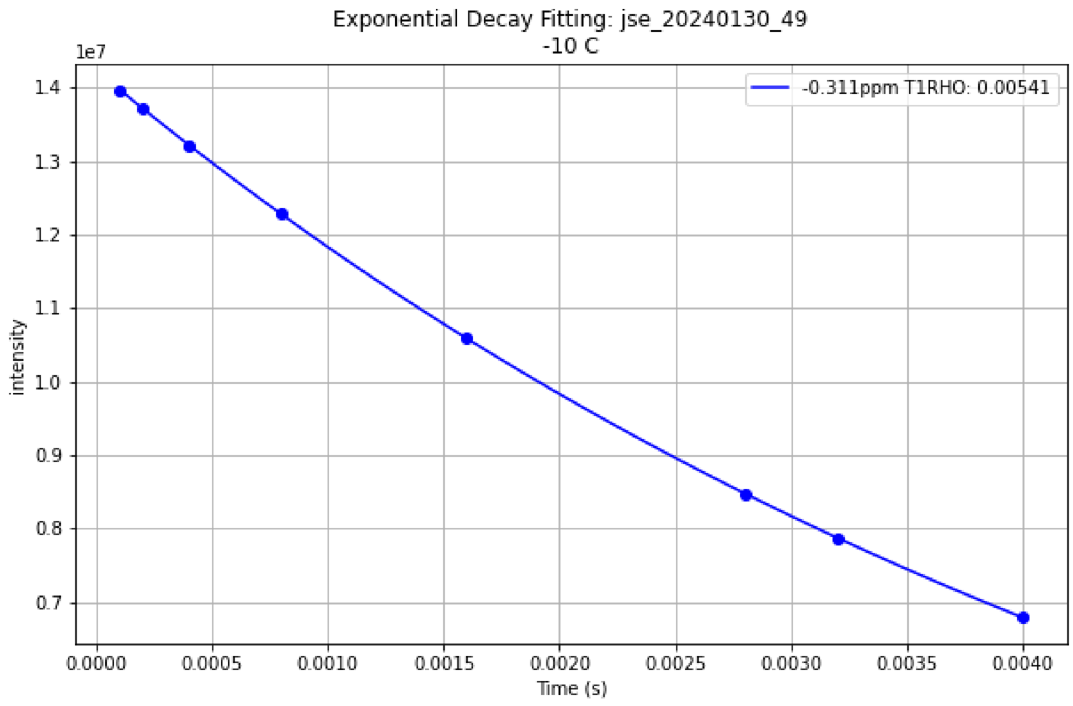

File 20: .../T1RHO_plot/jse_T1RHO_49.png

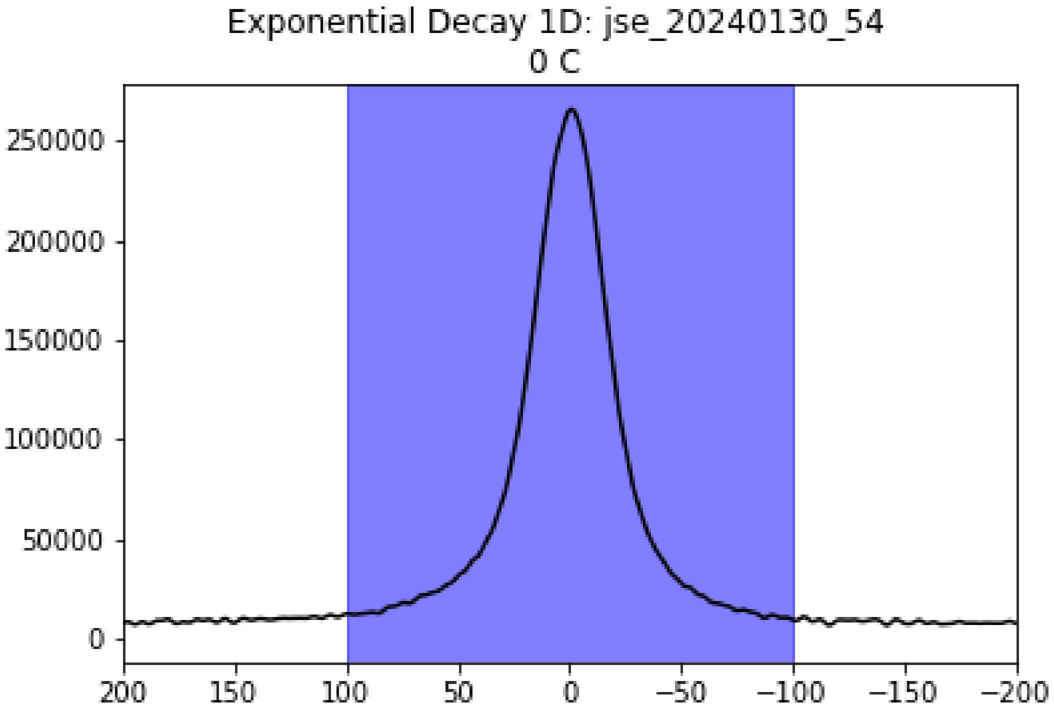

File 21: .../T1RHO_plot/jse_20240130_54_figure.png

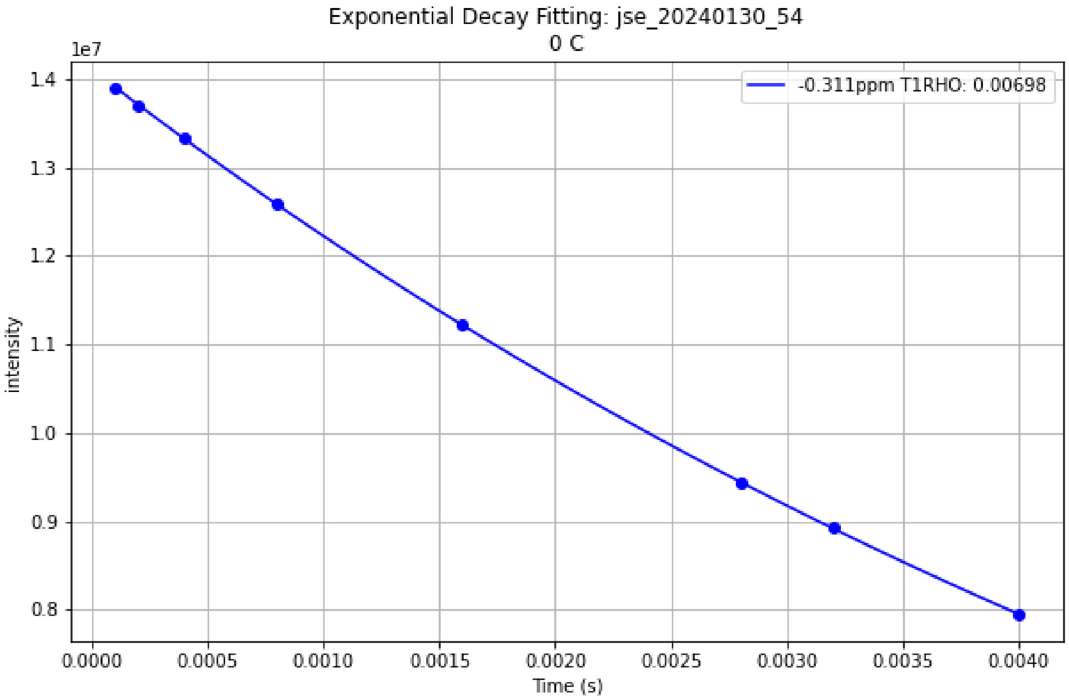

File 22: .../T1RHO_plot/jse_T1RHO_54.png

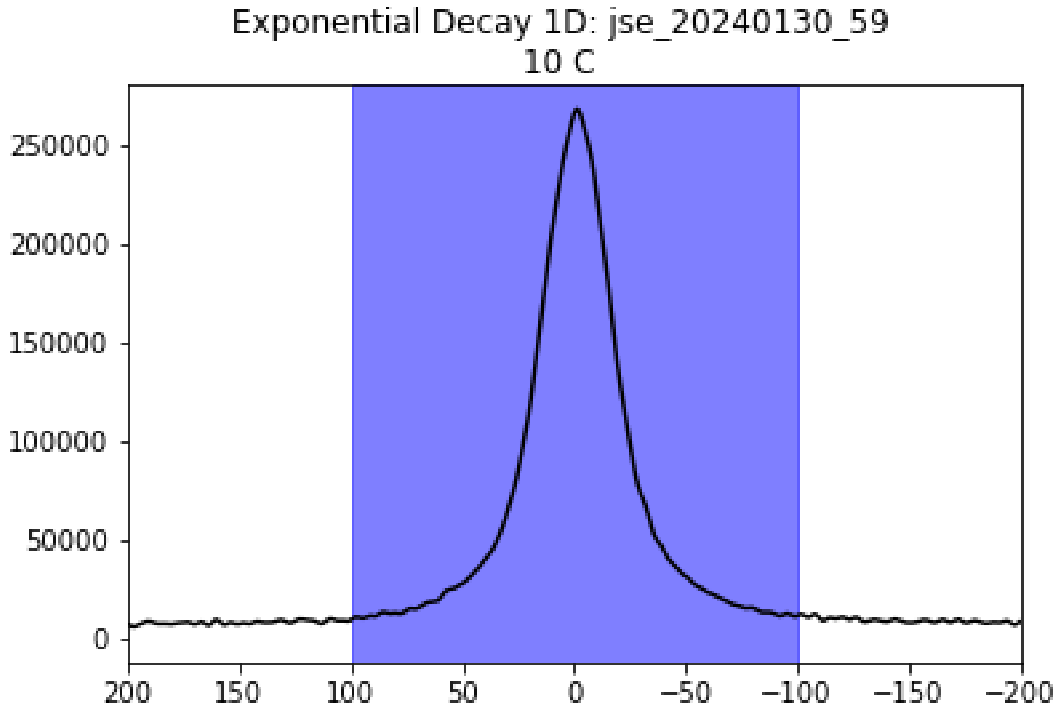

File 23: .../T1RHO_plot/jse_20240130_59_figure.png

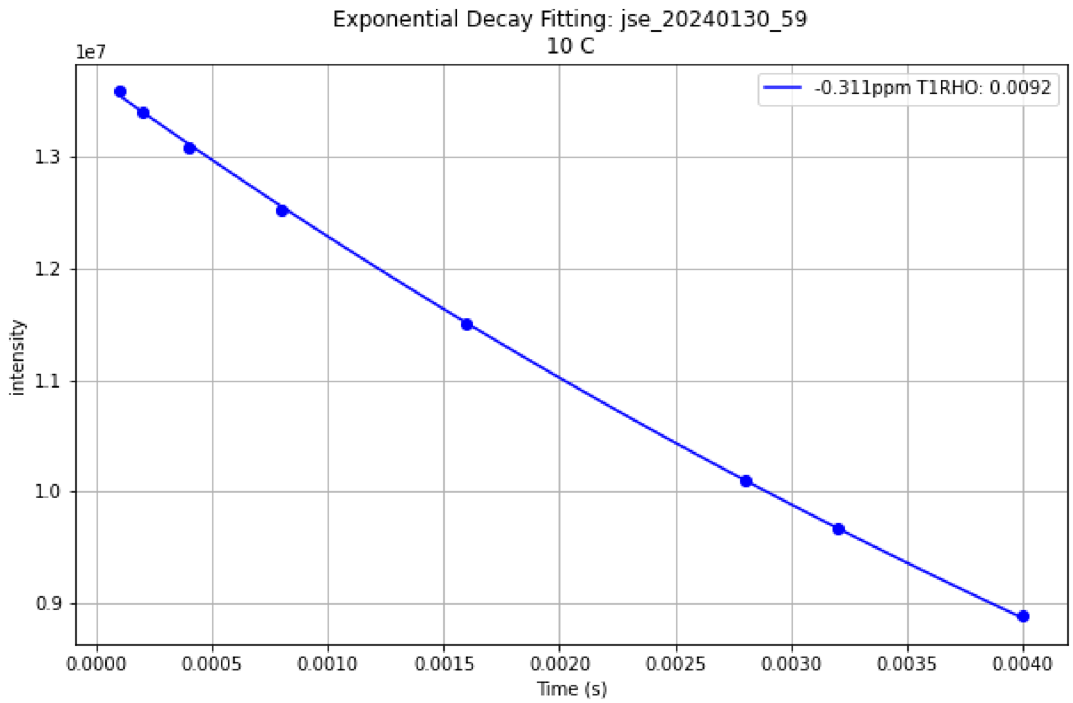

File 24: .../T1RHO_plot/jse_T1RHO_59.png

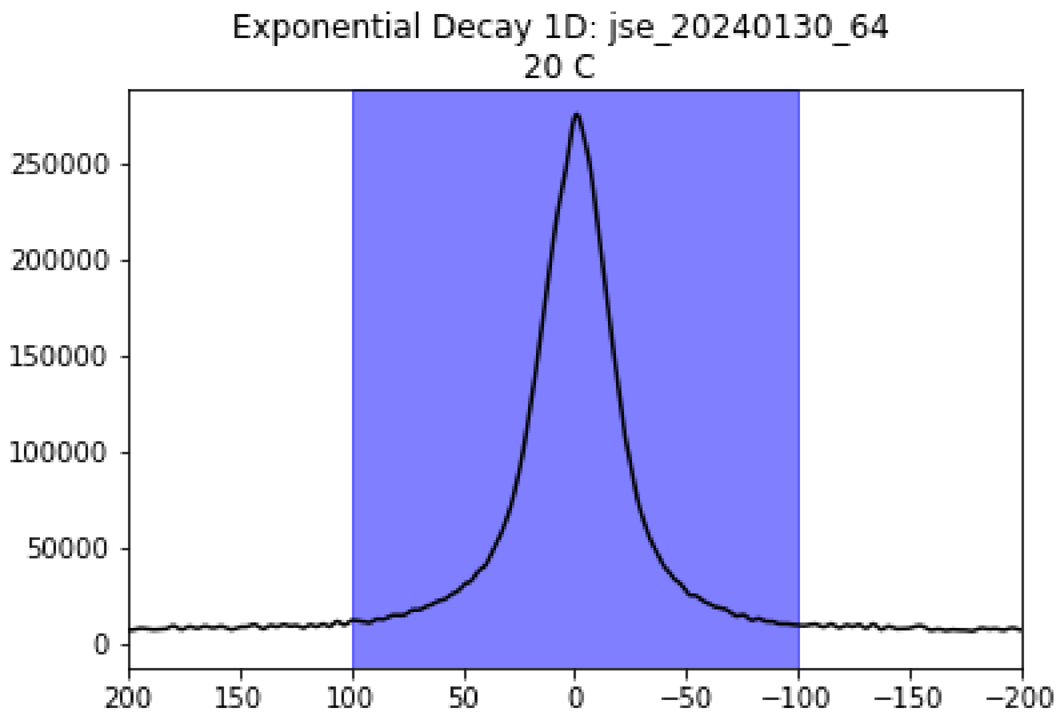

File 25: .../T1RHO_plot/jse_20240130_64_figure.png

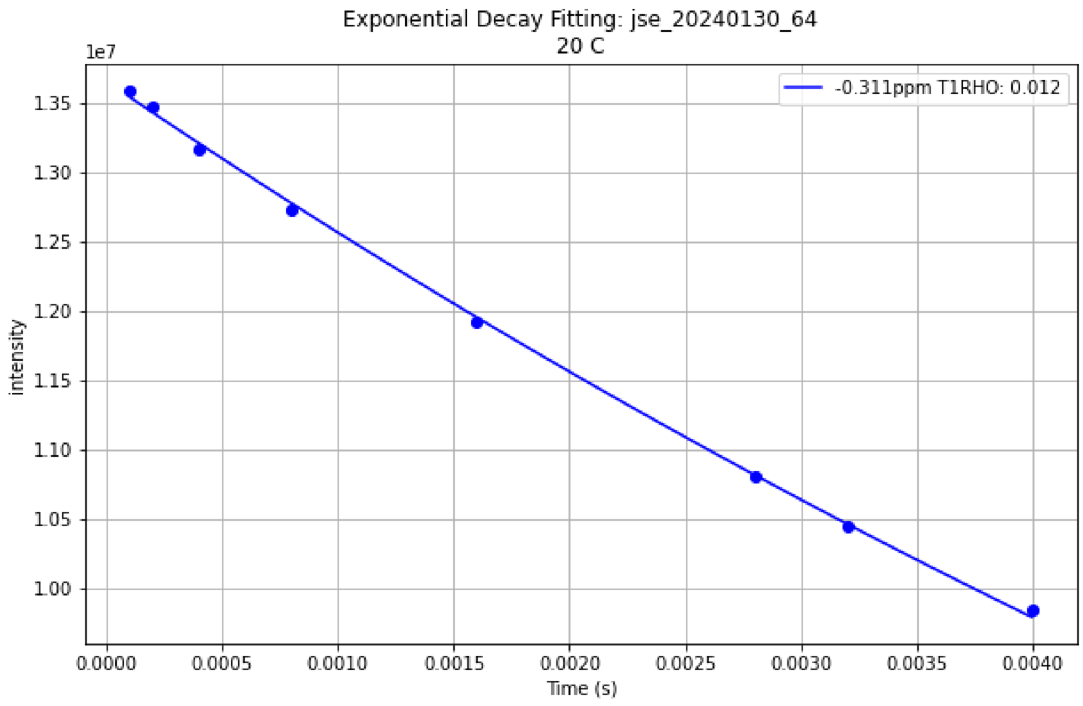

File 26: .../T1RHO_plot/jse_T1RHO_64.png

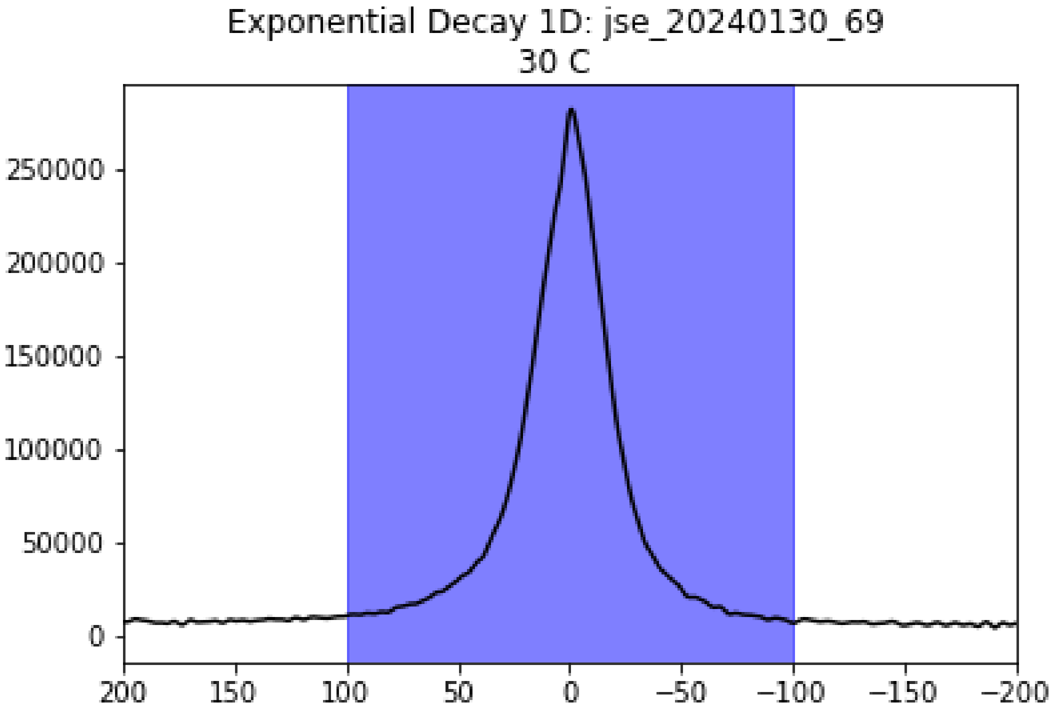

File 27: .../T1RHO_plot/jse_20240130_69_figure.png

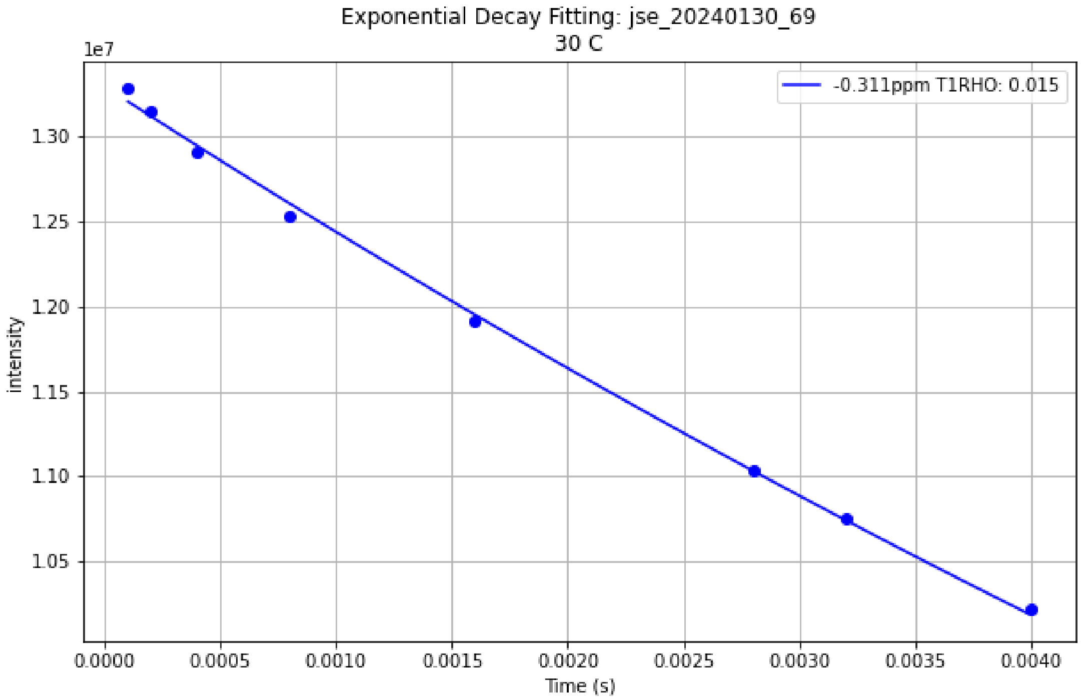

File 28: .../T1RHO_plot/jse_T1RHO_69.png

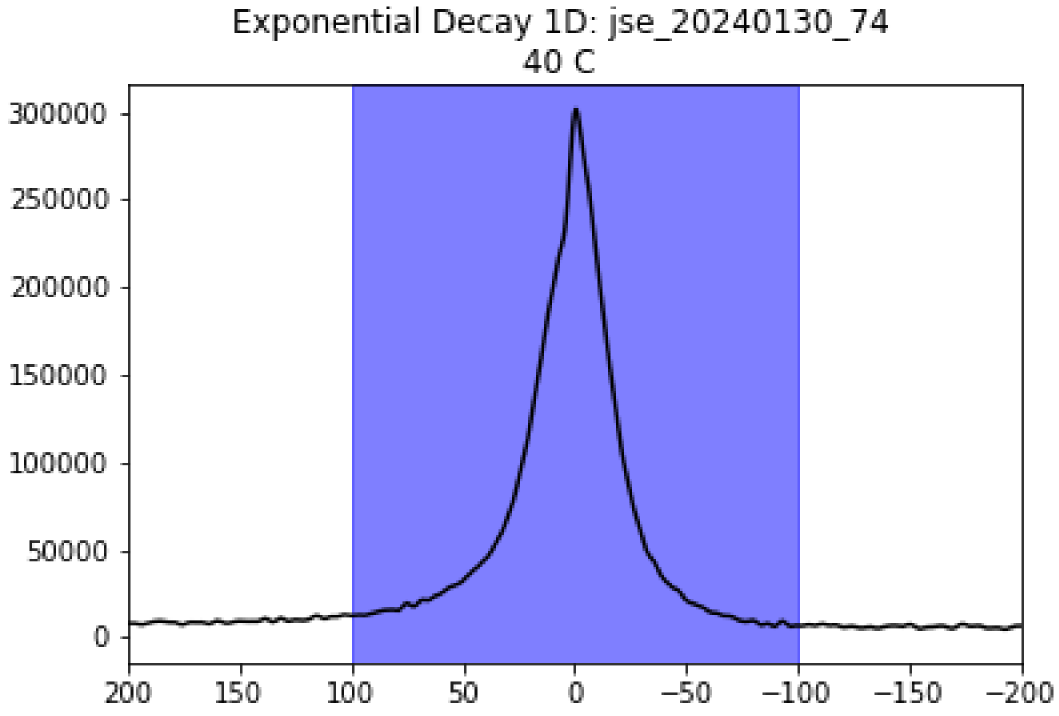

File 29: .../T1RHO_plot/jse_20240130_74_figure.png

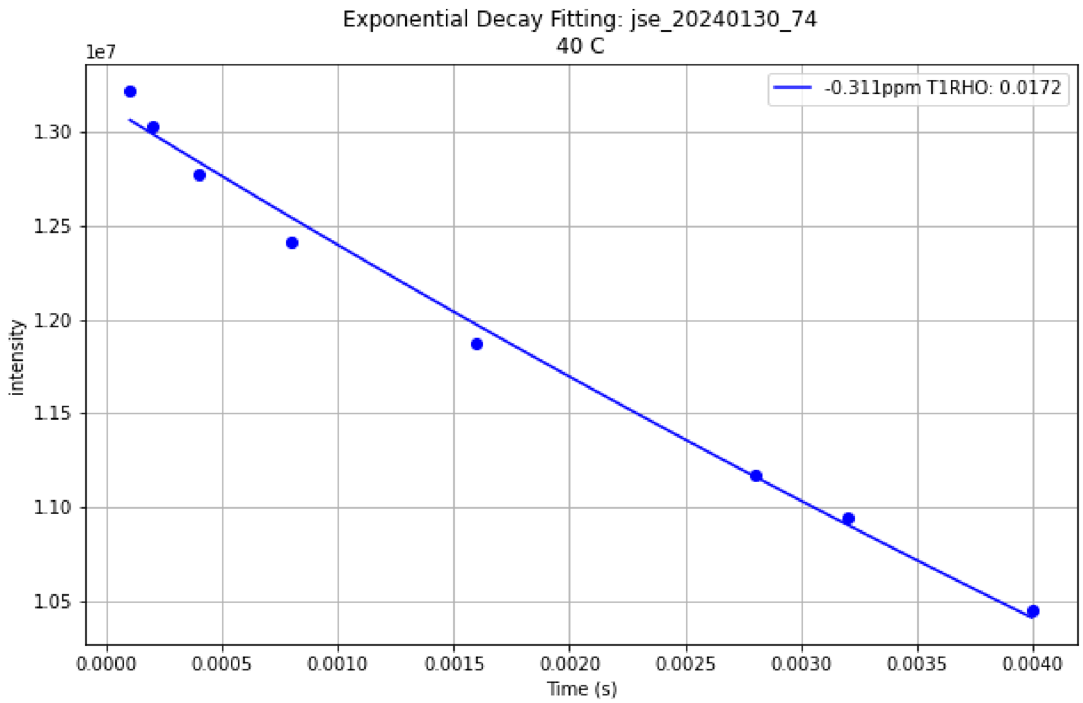

File 30: .../T1RHO_plot/jse_T1RHO_74.png

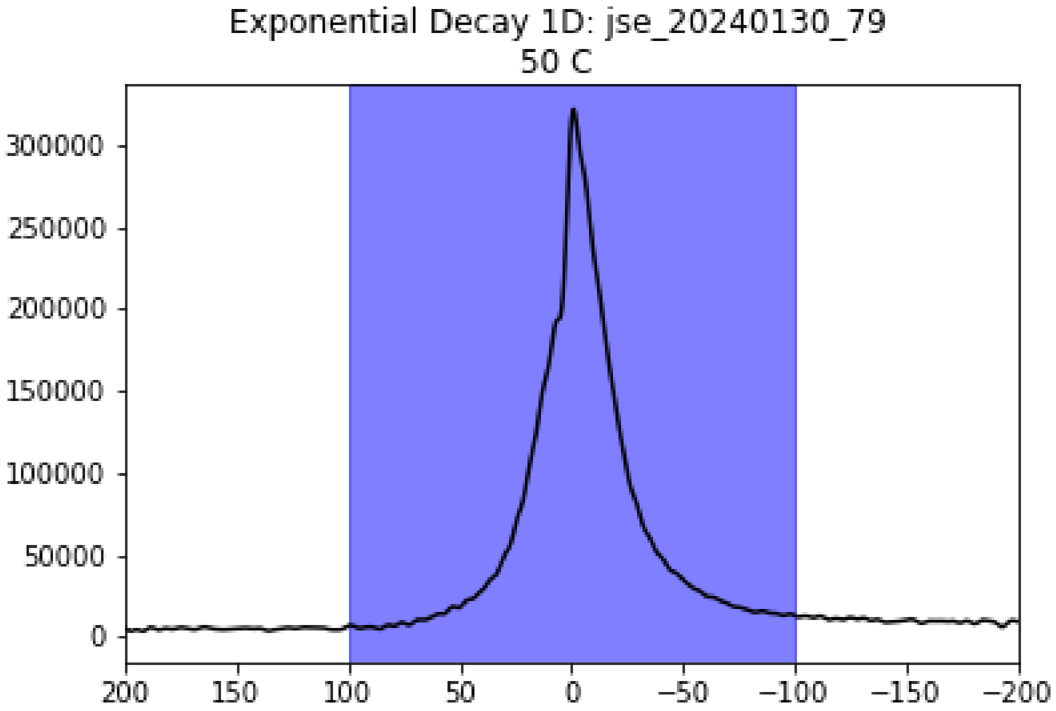

File 31: .../T1RHO_plot/jse_20240130_79_figure.png

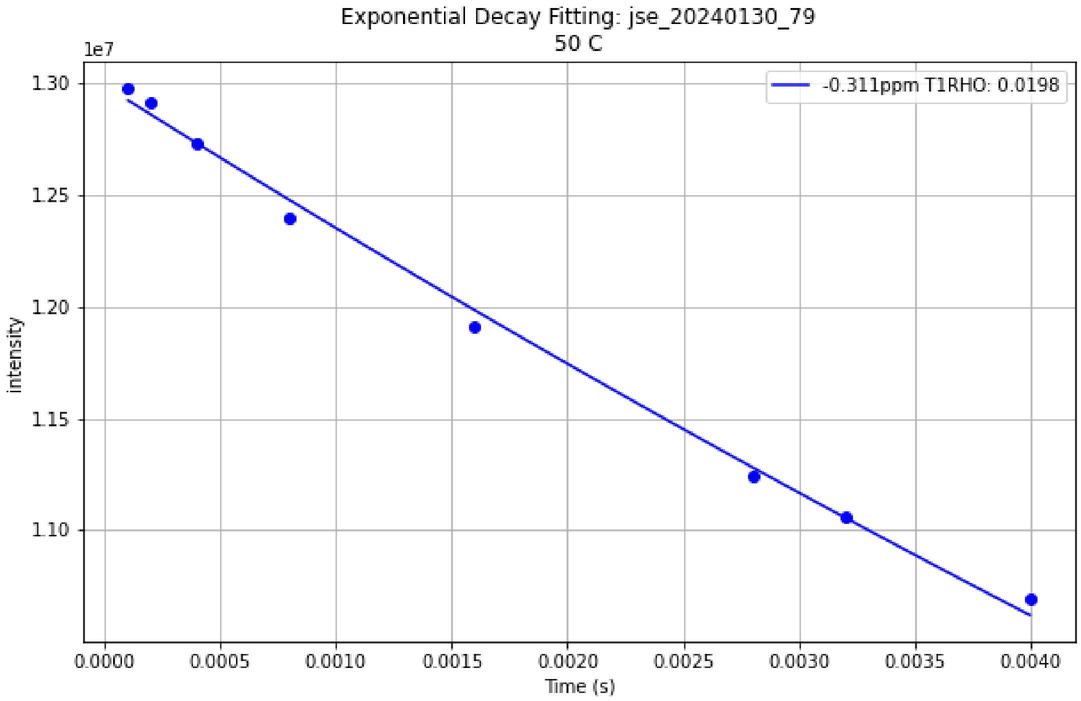

File 32: .../T1RHO_plot/jse_T1RHO_79.png

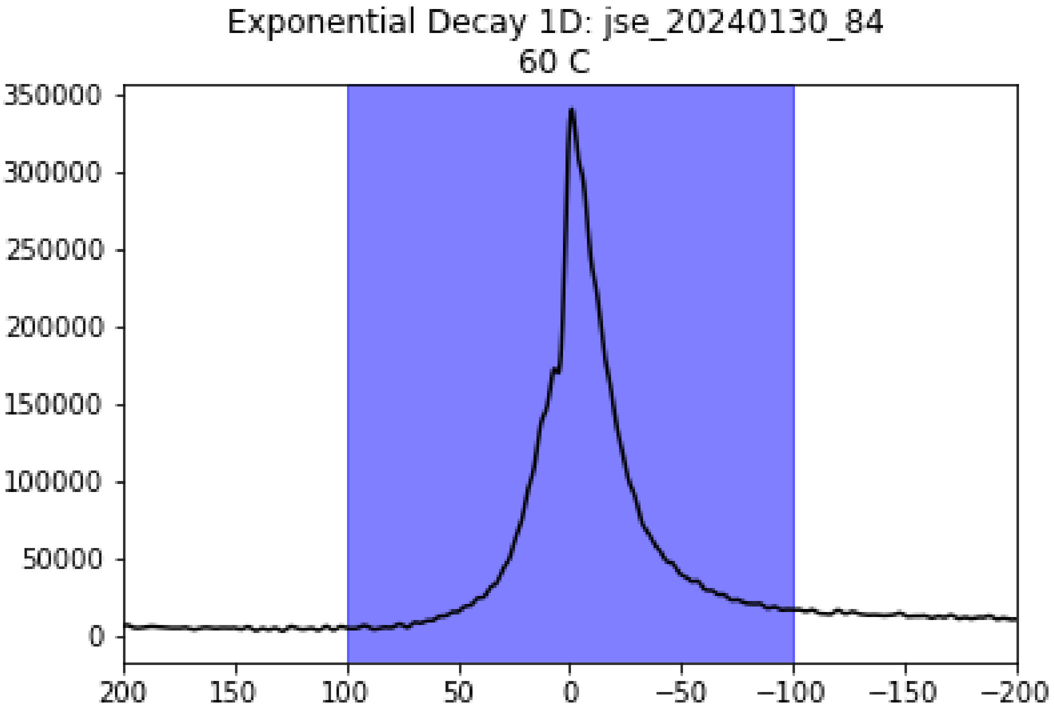

File 33: .../T1RHO_plot/jse_20240130_84_figure.png

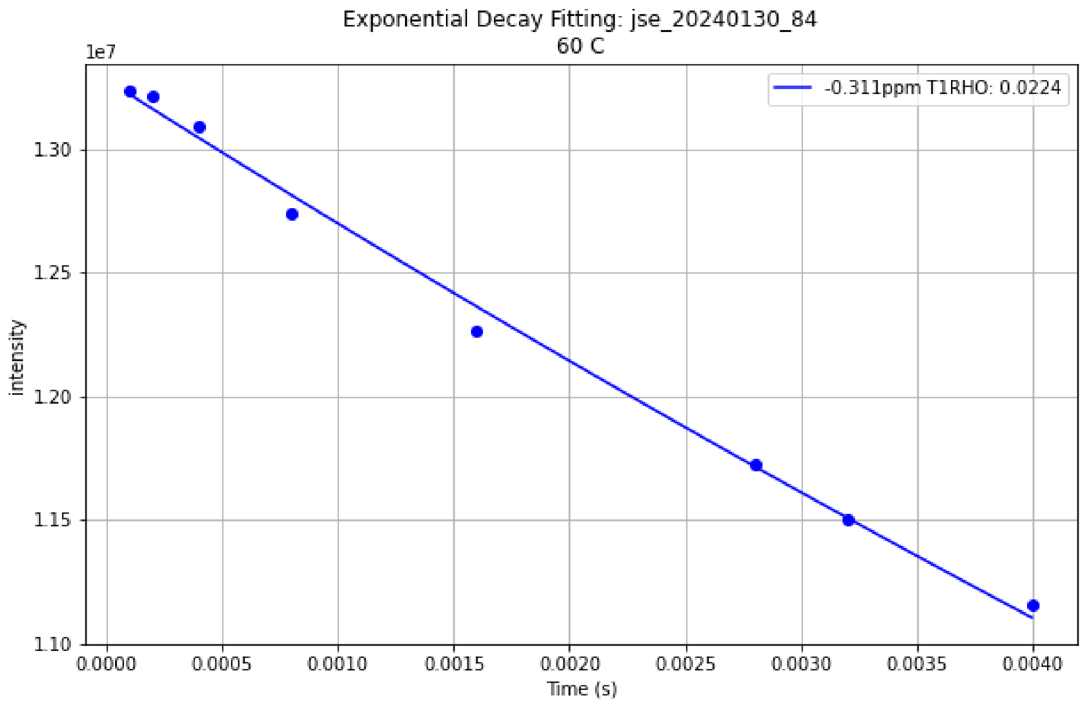

File 34: .../T1RHO_plot/jse_T1RHO_84.png

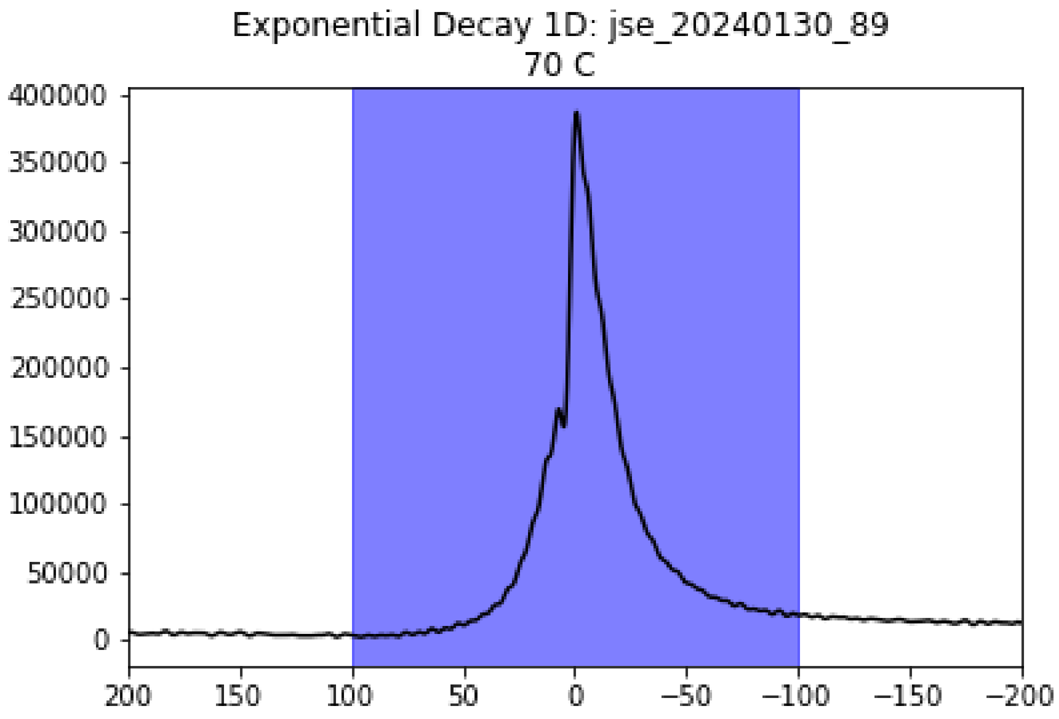

File 35: .../T1RHO_plot/jse_20240130_89_figure.png

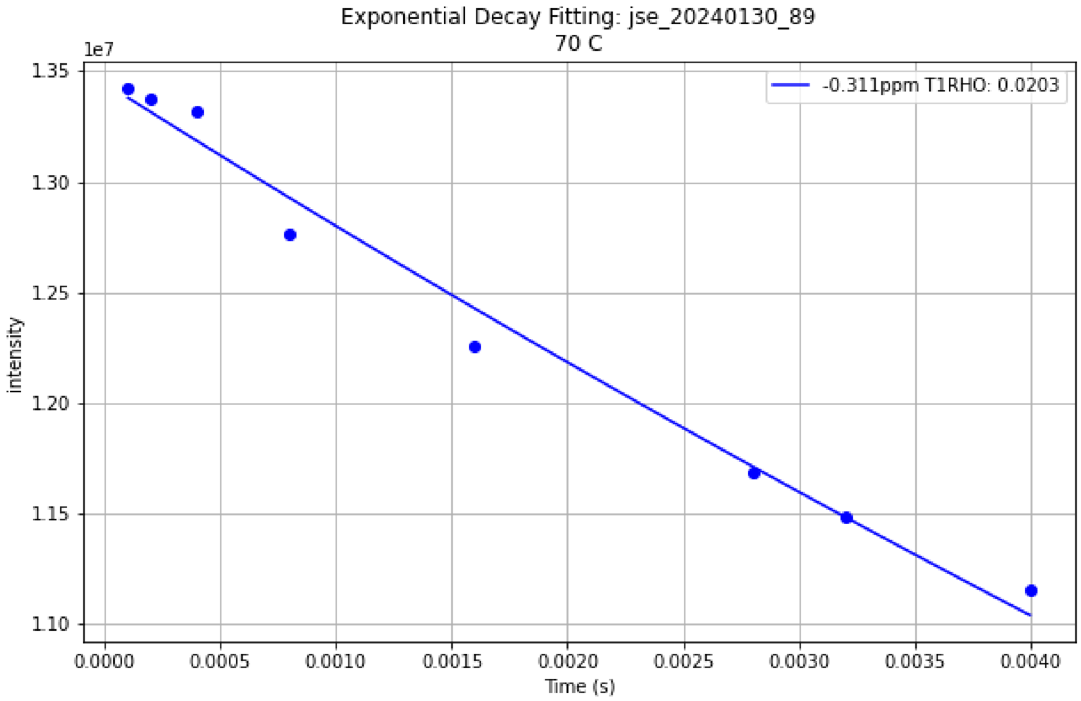

File 36: .../T1RHO_plot/jse_T1RHO_89.png

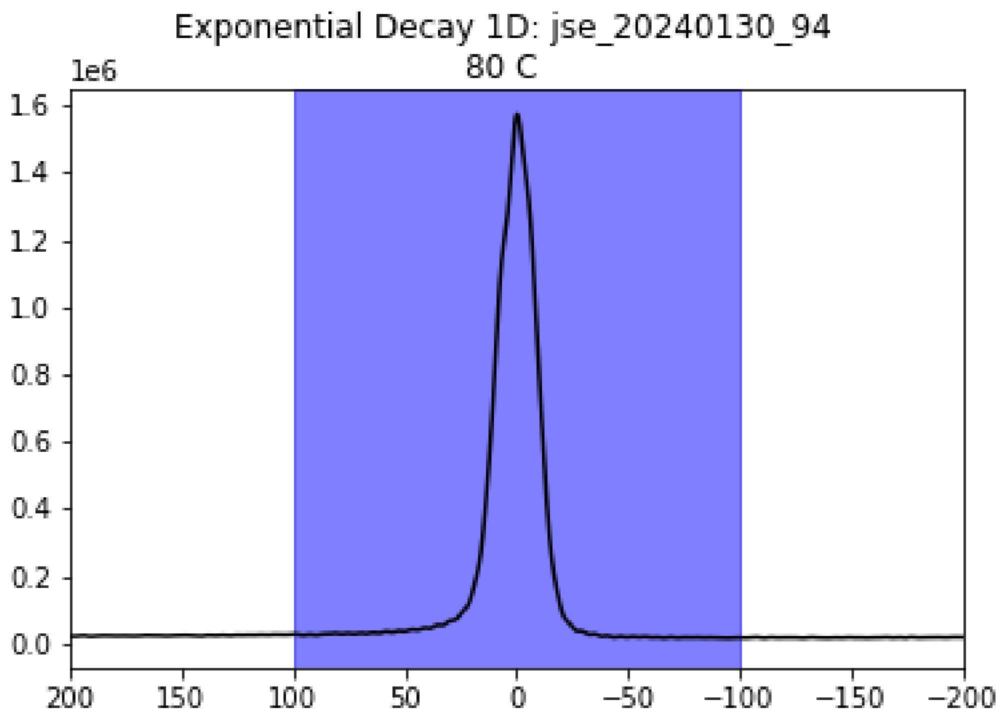

File 37: .../T1RHO_plot/jse_20240130_94_figure.png

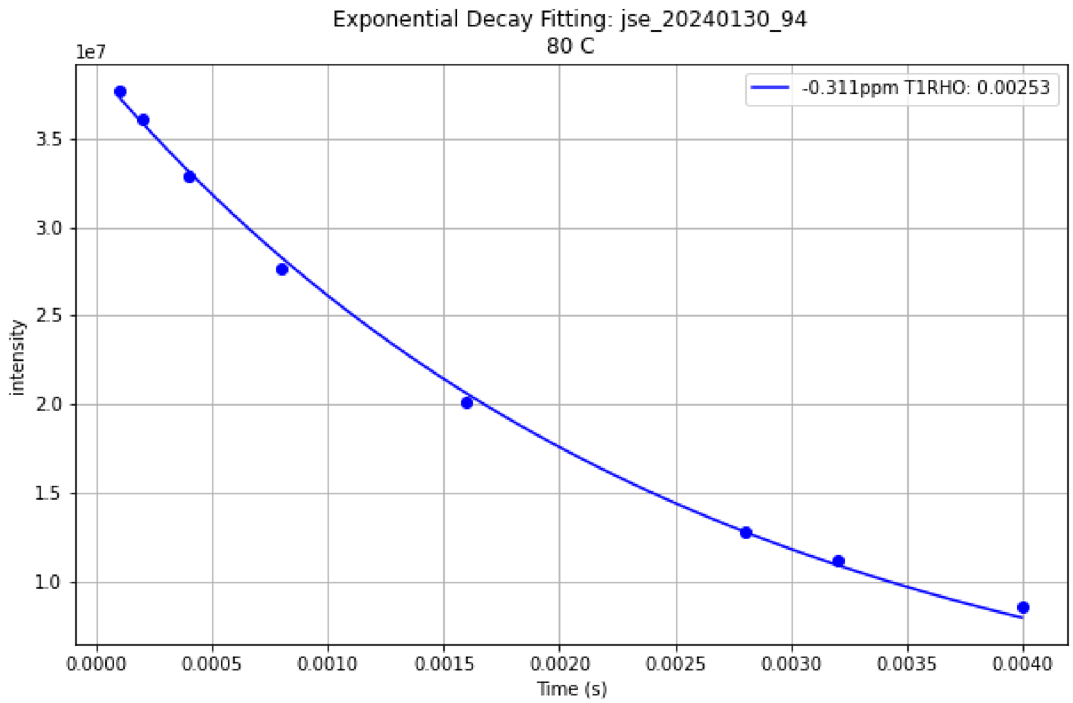

File 38: .../T1RHO_plot/jse_T1RHO_94.png

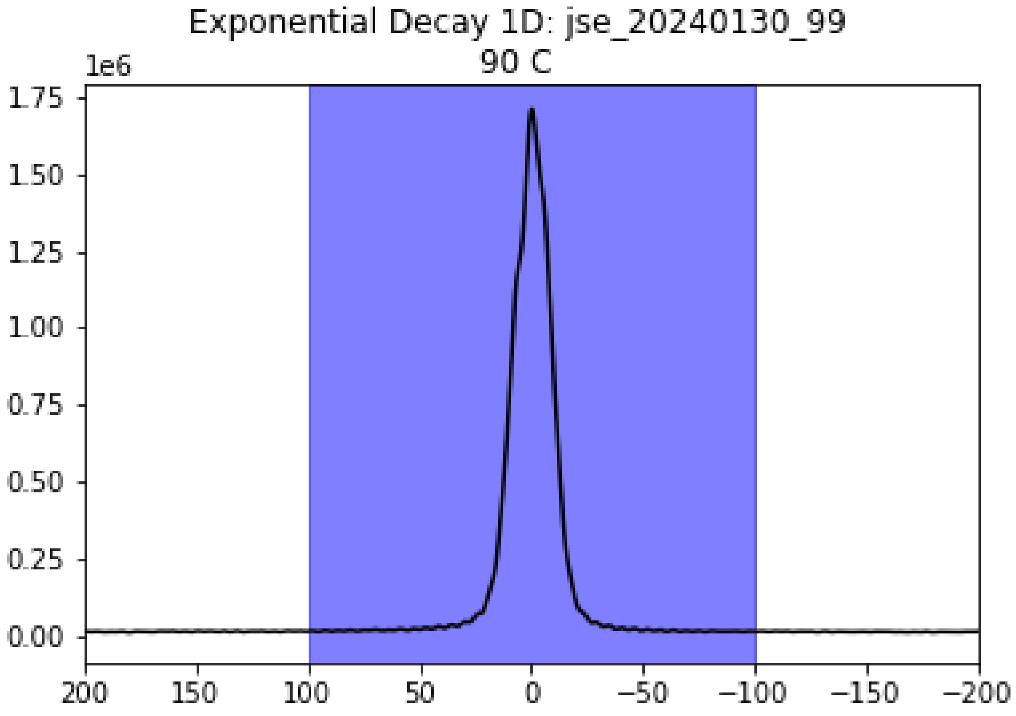

File 39: .../T1RHO_plot/jse_20240130_99_figure.png

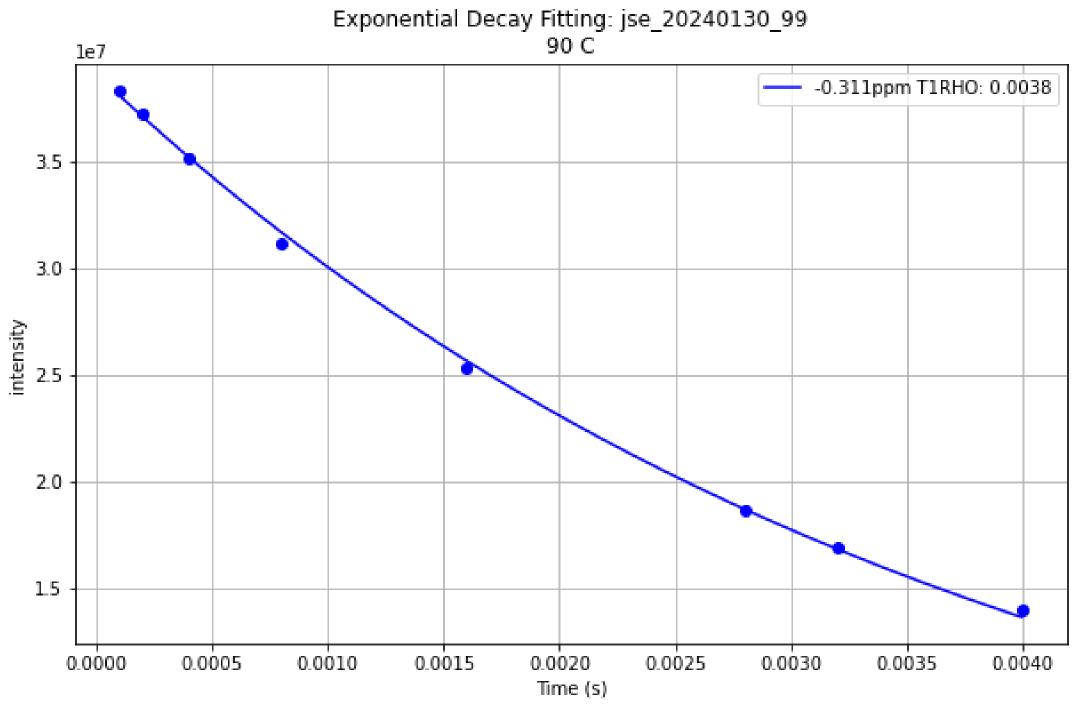

File 40: .../T1RHO_plot/jse_T1RHO_99.png

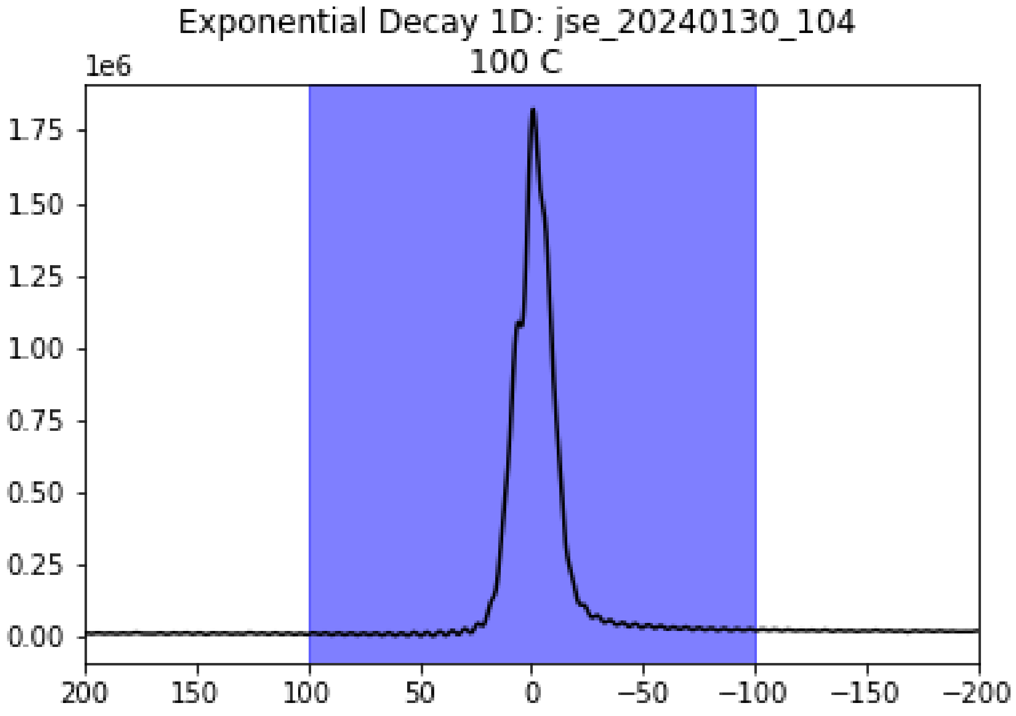

File 41: .../T1RHO_plot/jse_20240130_104_figure.png

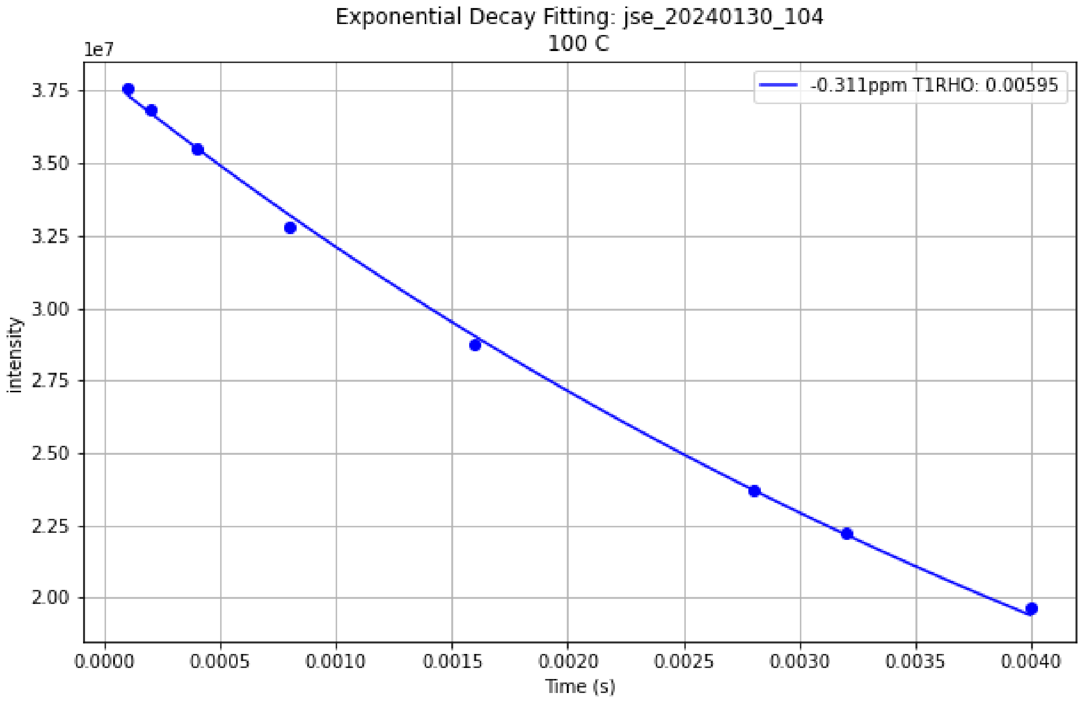

File 42: .../T1RHO_plot/jse_T1RHO_104.png

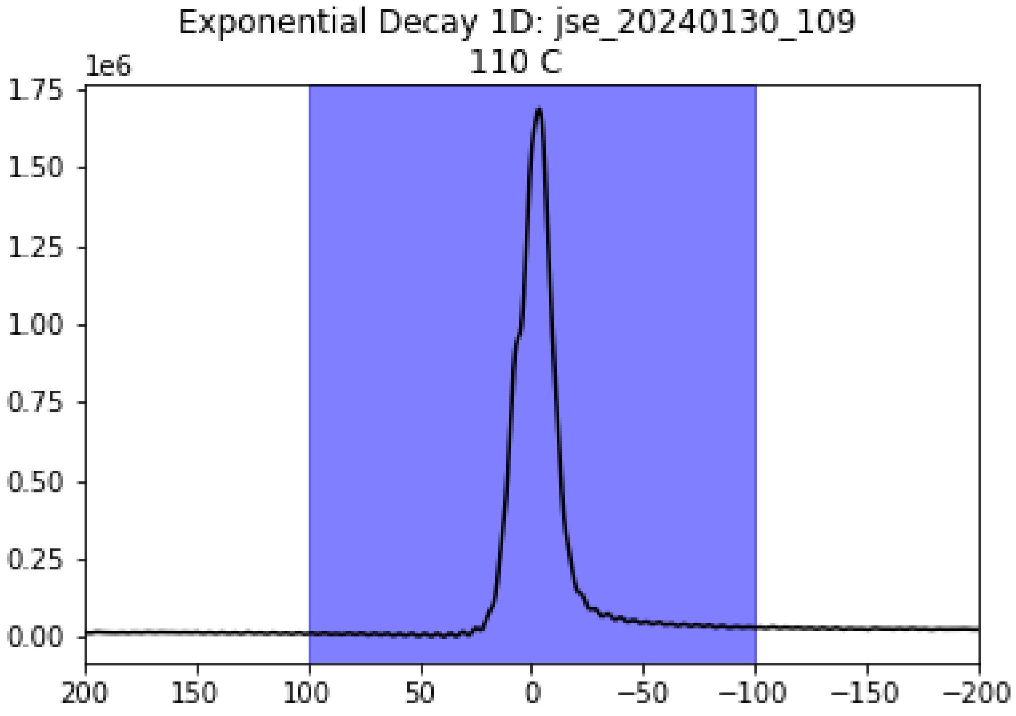

File 43: .../T1RHO_plot/jse_20240130_109_figure.png

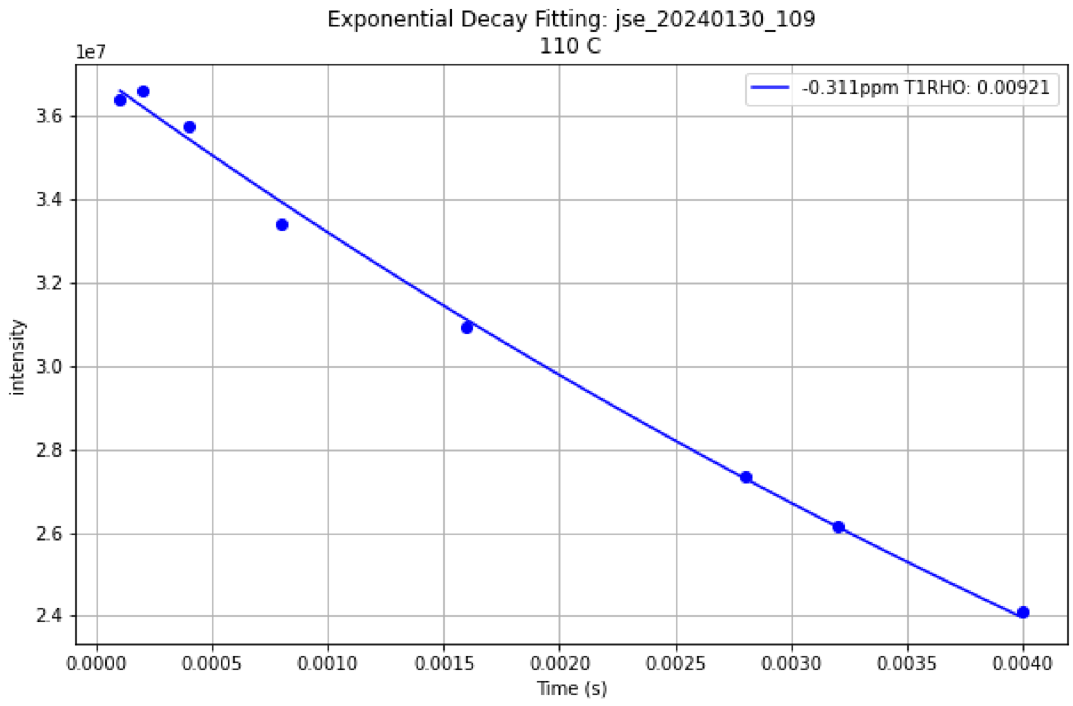

File 44: .../T1RHO_plot/jse_T1RHO_109.png

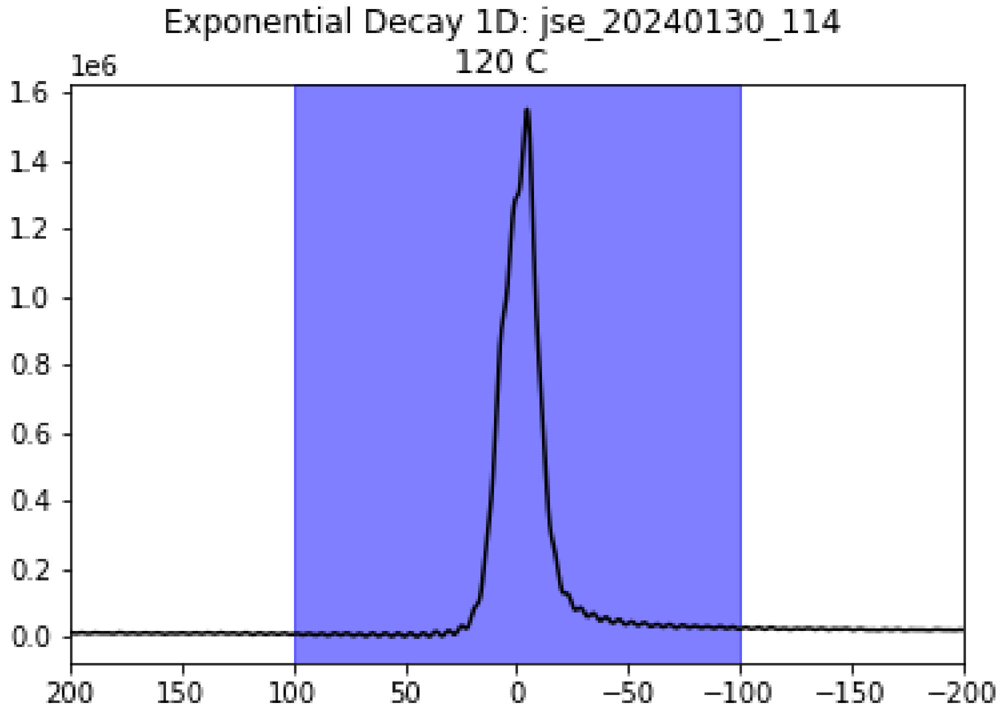

File 45: .../T1RHO_plot/jse_20240130_114_figure.png

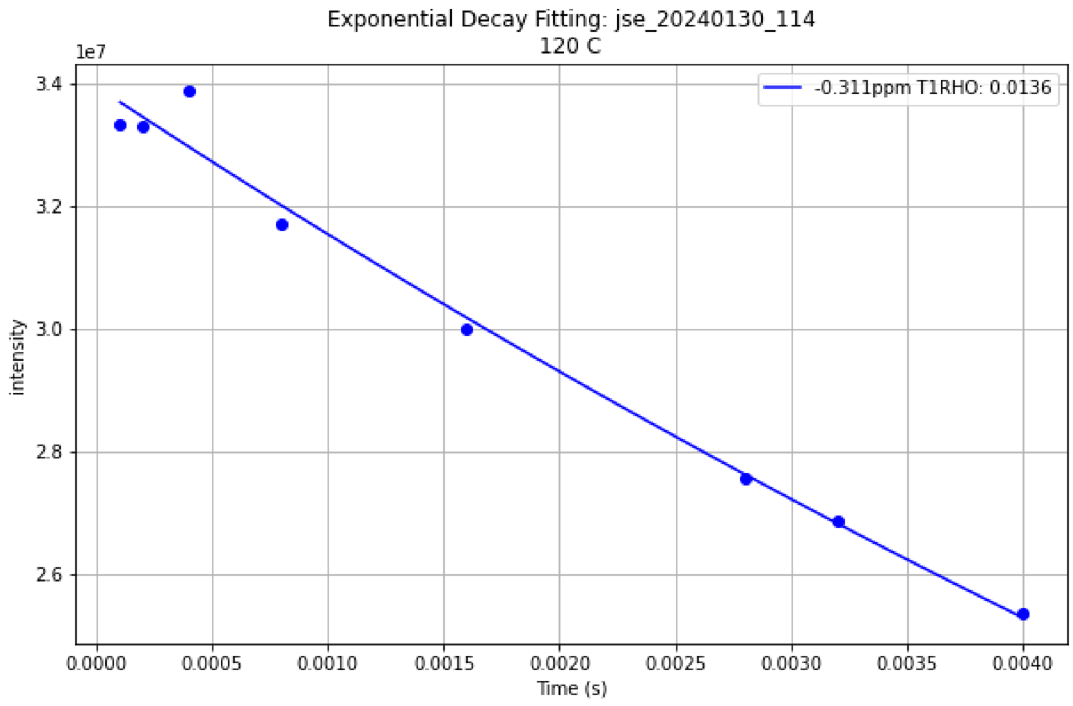

File 46: .../T1RHO_plot/jse_T1RHO_114.png

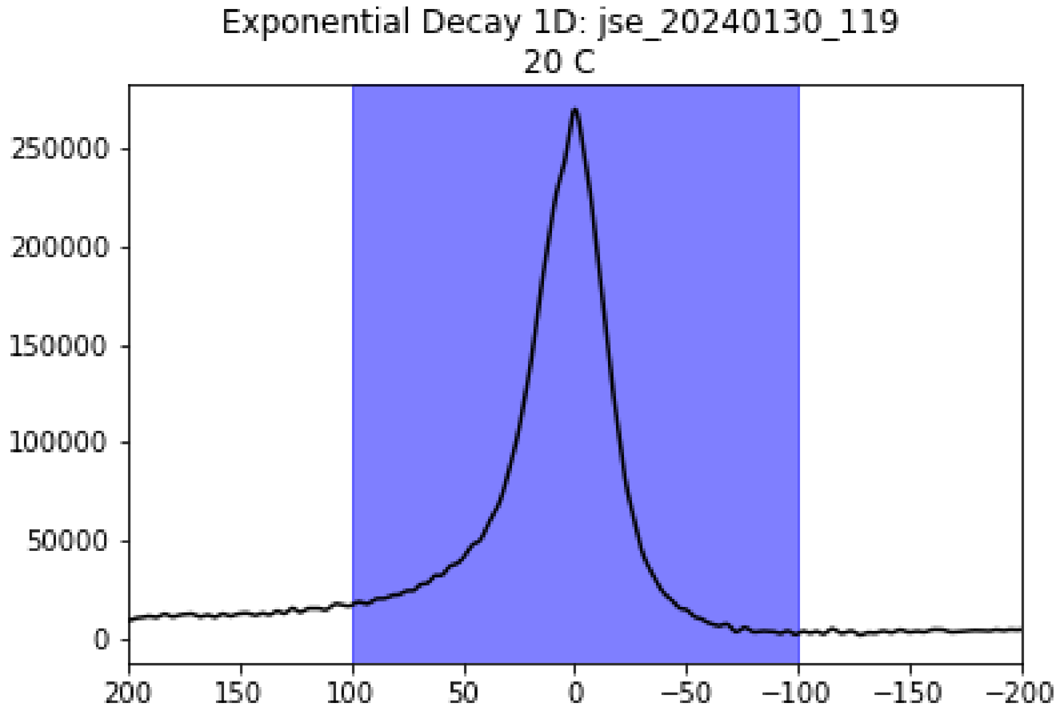

File 47: .../T1RHO_plot/jse_20240130_119_figure.png

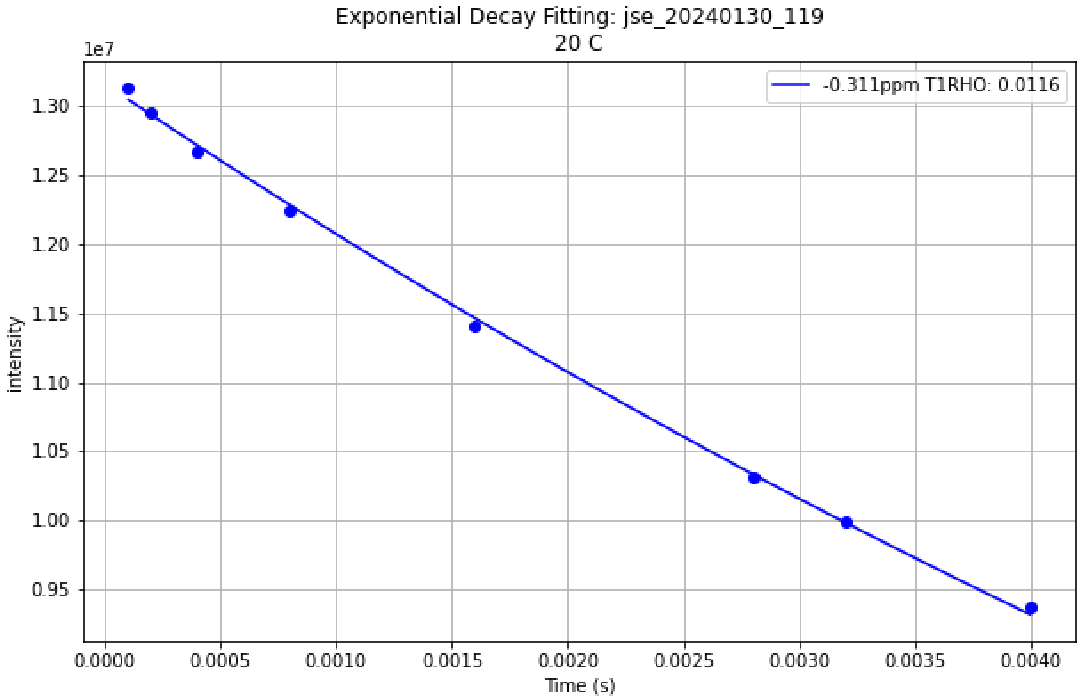

File 48: .../T1RHO_plot/jse_T1RHO_119.png
