# Supplementary material for: Potential Multiaxial Molecular Ferroelectricity through Chiral Cation Replacement
Source: Cryst Growth Des. 2025 Jul 21;25(15):6237–47. doi: 10.1021/acs.cgd.5c00666 (PMC12332970; doi:10.1021/acs.cgd.5c00666)

2624-10340 SYT0043a S-CTA CdCl4 @ static / -100 to +120 C
-100 C

```
Current Data Parameters
NAME       jse_20240130
EXPNO                 1
PROCNO                1

F2 - Acquisition Parameters
Date_          20240130
PROBHD   5 mm PE BB/1H/
PULPROG       solidecho
NS                    8
SWH         1000000.000 Hz
AQ            0.0005120 sec
TE                213.4 K
D1         104.00000000 sec
D6           0.00005000 sec
D7           0.00002750 sec

======== CHANNEL f1 ========
SFO1        400.1712930 MHz
NUC1                 1H
P1                 2.50 usec
PLW1       378.44268799 W

F2 - Processing parameters
SI                 8192
SF          400.1713306 MHz
WDW                  EM
SSB                   0
LB                10.00 Hz
GB                    0
PC                 1.00
```

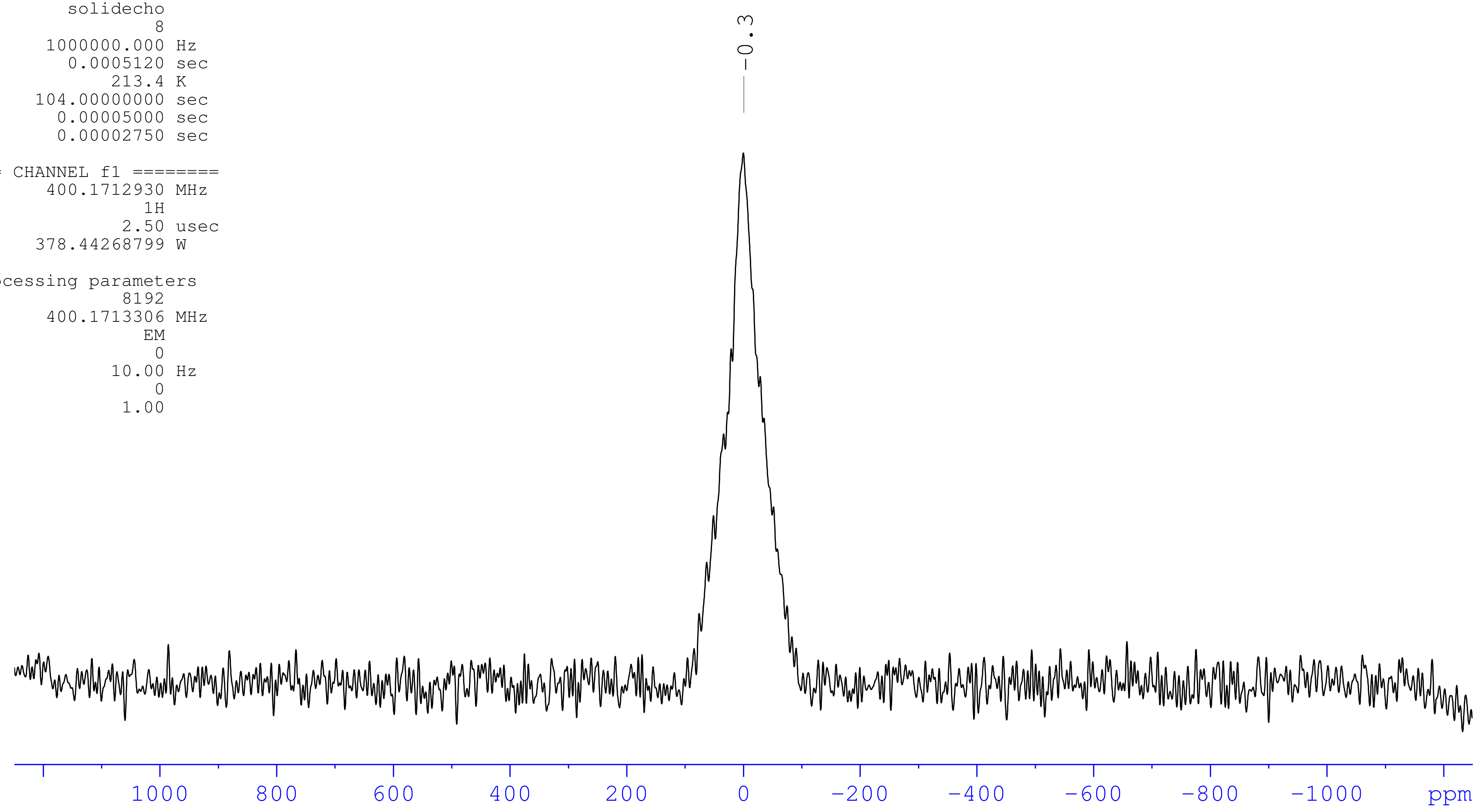

```
Current Data Parameters
NAME       jse_20240130
EXPNO                  2
PROCNO                 1

F2 - Acquisition Parameters
Date_          20240130
PROBHD    5 mm PE BB/1H/
PULPROG               zg
NS                     4
SWH         1000000.000 Hz
AQ            0.0005120 sec
TE                294.0 K
D1         104.00000000 sec
TD0                    1

======== CHANNEL f1 ========
SFO1        400.1712930 MHz
NUC1                 1H
P1                 2.50 usec
PLW1       378.44268799 W

F2 - Processing parameters
SI                 8192
SF          400.1713306 MHz
WDW                  EM
SSB                   0
LB                10.00 Hz
GB                    0
PC                 0.20
```

2624-10340 SYT0043a S-CTA CdCl4 @ static / -100 to +120 C
-100 C

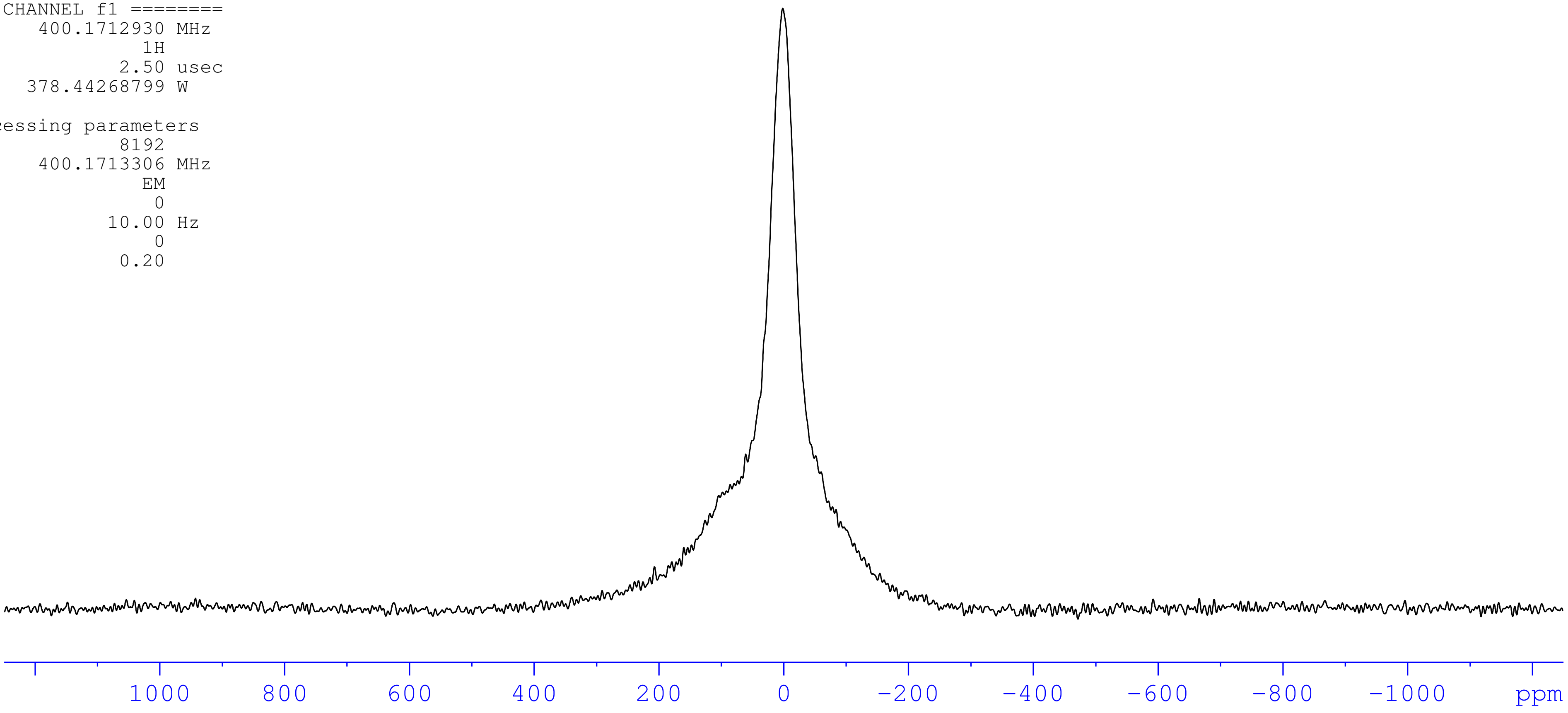

Current Data Parameters
NAME jse_20240130
EXPNO 3
PROCNO 1

F2 - Acquisition Parameters
Date_ 20240130
PROBHD 5 mm PE BB/1H/
PULPROG satrect1se
NS 8
SWH 1000000.000 Hz
AQ 0.0005120 sec
TE 298.0 K
D1 1.00000000 sec
D6 0.00004625 sec
D7 0.00002750 sec
D20 0.00040000 sec
L20 64
VDLIST Recovery_0.1_102.4_16

======== CHANNEL f1 ========
SFO1 400.1712930 MHz
NUC1 1H
P1 2.50 usec
PLW1 378.44268799 W

F1 - Acquisition parameters
TD 16
SFO1 400.1713 MHz
FIDRES 500.000000 Hz
SW 9.996 ppm
FnMODE QF

F2 - Processing parameters
SI 8192
SF 400.1713306 MHz
WDW no
SSB 0
LB 0 Hz
GB 0
PC 0.20

F1 - Processing parameters
SI 16
MC2 QF
SF 400.1700000 MHz
WDW no
SSB 0
LB 0 Hz
GB 0

2624-10340 SYT0043a S-CTA CdCl4 @ static / -100 to +120 C
-100 C

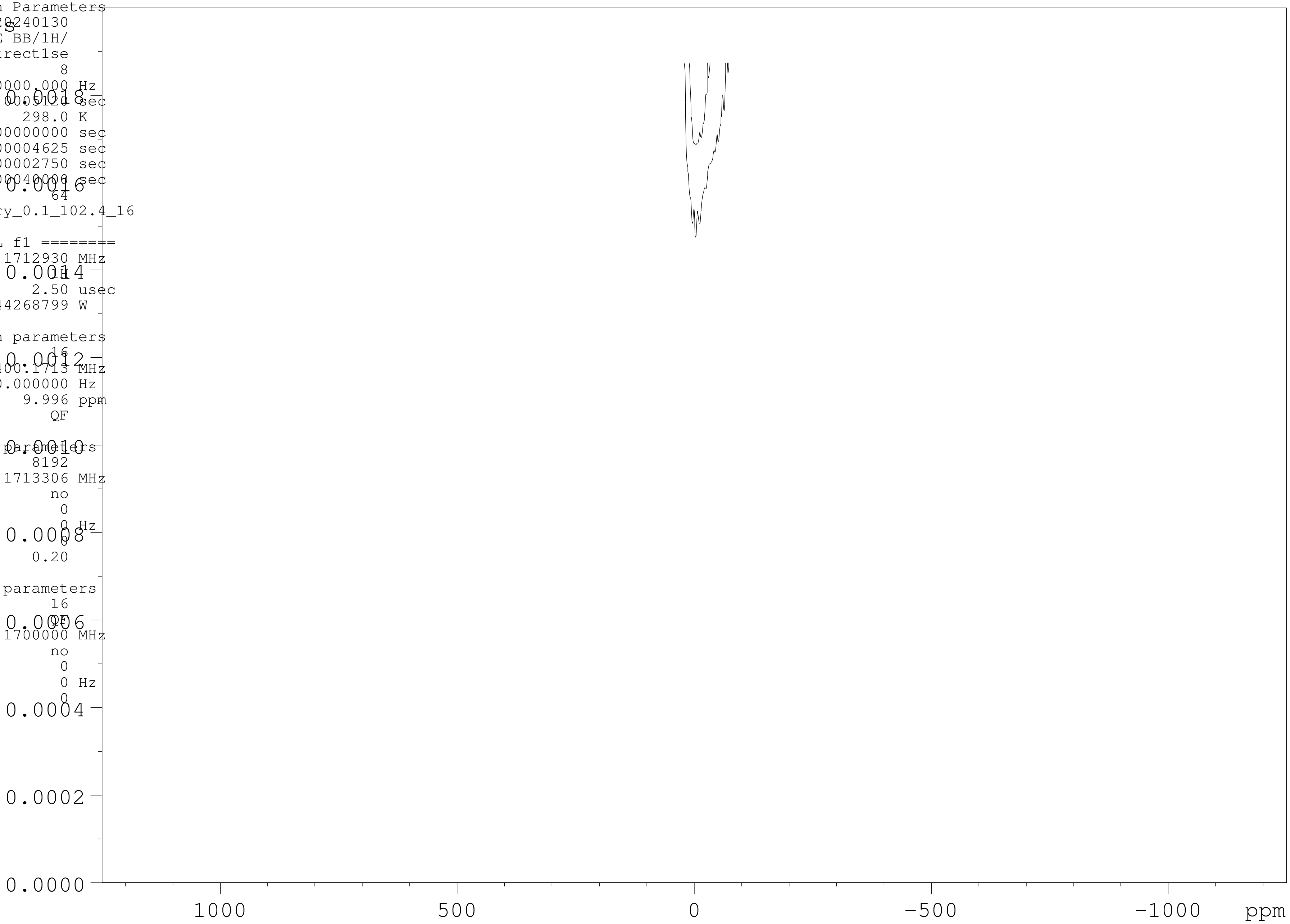

Current Data Parameters
NAME jse_20240130
EXPNO 4
PROCNO 1

2624-10340 SYT0043a S-CTA CdCl4 @ static / -100 to +120 C
-100 C

F2 - Acquisition Parameters
Date_ 20240131
PROBHD 5 mm PE BB/1H/
PULPROG t1rho_solidecho
NS 8
SWH 1000000.000 Hz
AQ 0.0005120 sec
TE 220.1 K
D1 104.00000000 sec
D6 0.00005000 sec
D7 0.00002700 sec

======== CHANNEL f1 ========
SFO1 400.1712930 MHz
NUC1 1H
P1 2.50 usec
PLW1 378.44268799 W
PLW2 94.62400055 W
VPLIST 100u_52000u_16

F1 - Acquisition parameters
TD 8
SFO1 400.1713 MHz
FIDRES 1000.000000 Hz
SW 9.996 ppm
FnMODE QF

F2 - Processing parameters
SI 8192
SF 400.1713306 MHz
WDW no
SSB 0
LB 0 Hz
GB 0
PC 0.20

F1 - Processing parameters
SI 8
MC2 QF
SF 400.1700000 MHz
WDW no
SSB 0
LB 0 Hz
GB 0

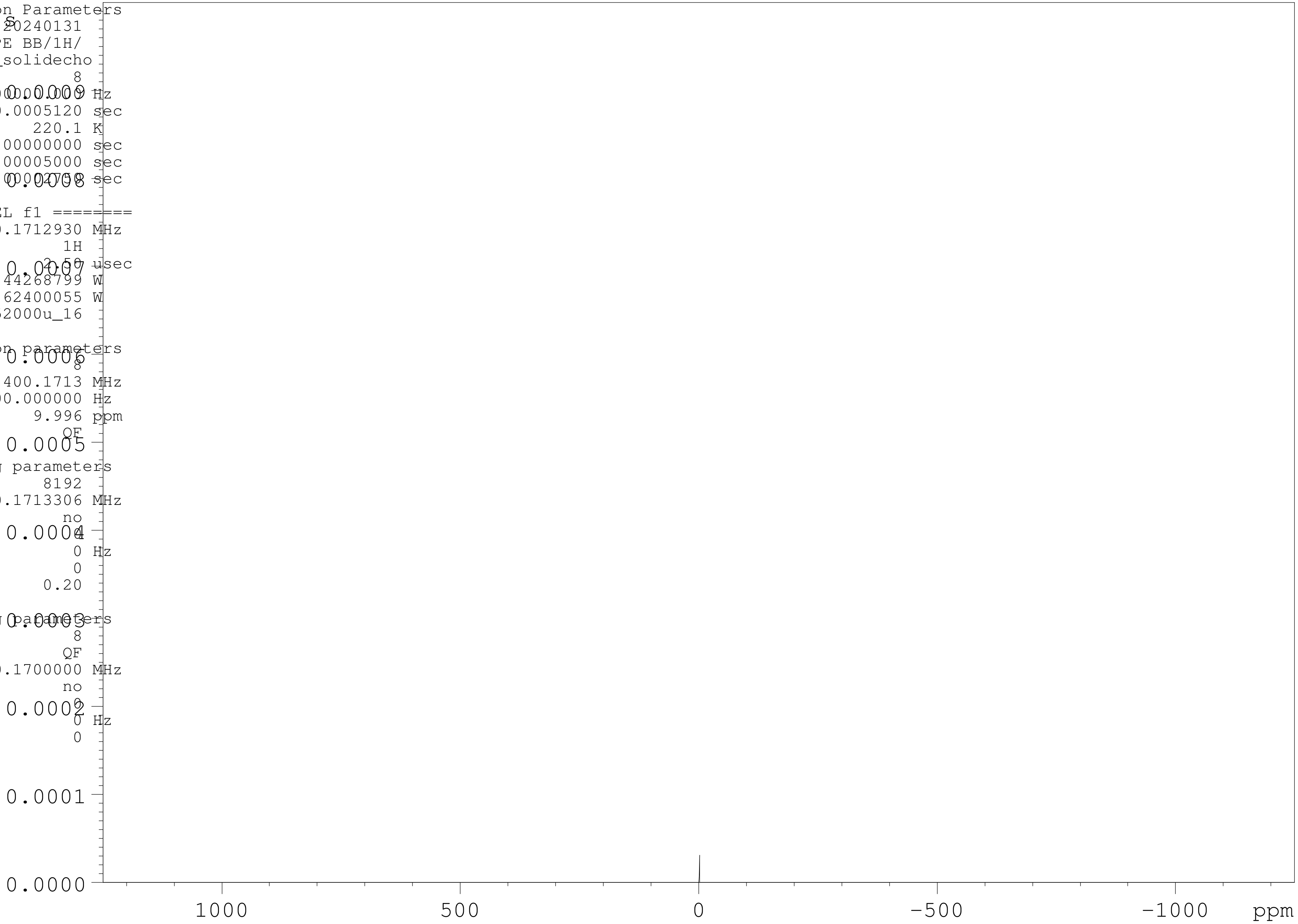

Current Data Parameters
NAME jse_20240130
EXPNO 6
PROCNO 1

F2 - Acquisition Parameters
Date_ 20240131
PROBHD 5 mm PE BB/1H/
PULPROG solidecho
NS 8
SWH 1000000.000 Hz
AQ 0.0005120 sec
TE 213.4 K
D1 58.43999863 sec
D6 0.00005000 sec
D7 0.00002750 sec

======== CHANNEL f1 ========
SFO1 400.1712930 MHz
NUC1 1H
P1 2.50 usec
PLW1 378.44268799 W

F2 - Processing parameters
SI 8192
SF 400.1713306 MHz
WDW EM
SSB 0
LB 10.00 Hz
GB 0
PC 0.20

2624-10340 SYT0043a S-CTA CdCl4 @ static / -100 to +120 C
-90 C

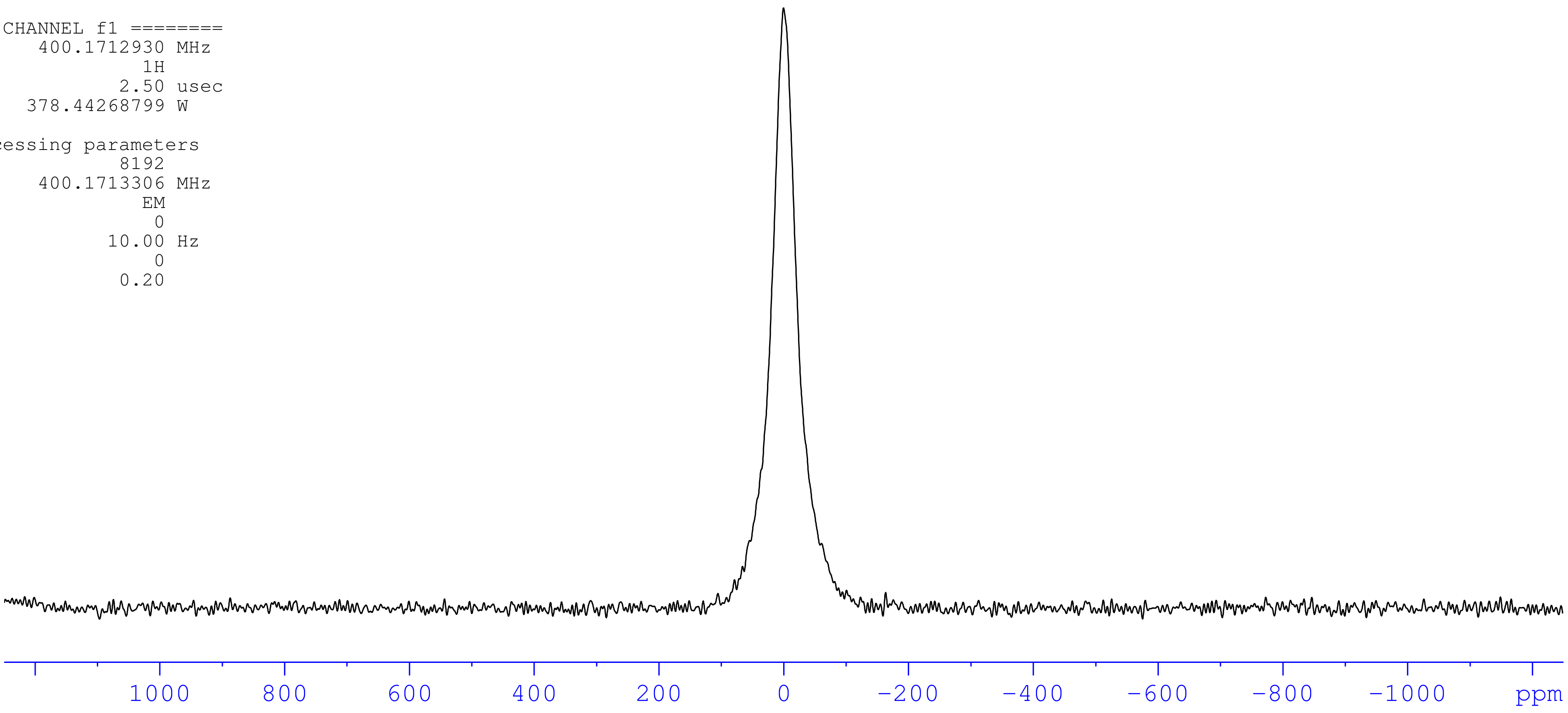

```
Current Data Parameters
NAME       jse_20240130
EXPNO                 7
PROCNO                1

F2 - Acquisition Parameters
Date_          20240131
PROBHD   5 mm PE BB/1H/
PULPROG              zg
NS                    4
SWH         1000000.000 Hz
AQ            0.0005120 sec
TE                294.0 K
D1          58.43999863 sec
TD0                   1

======== CHANNEL f1 ========
SFO1        400.1712930 MHz
NUC1                 1H
P1                 2.50 usec
PLW1       378.44268799 W

F2 - Processing parameters
SI                 8192
SF          400.1713306 MHz
WDW                  EM
SSB                   0
LB                10.00 Hz
GB                    0
PC                 0.20
```

2624-10340 SYT0043a S-CTA CdCl4 @ static / -100 to +120 C
-90 C

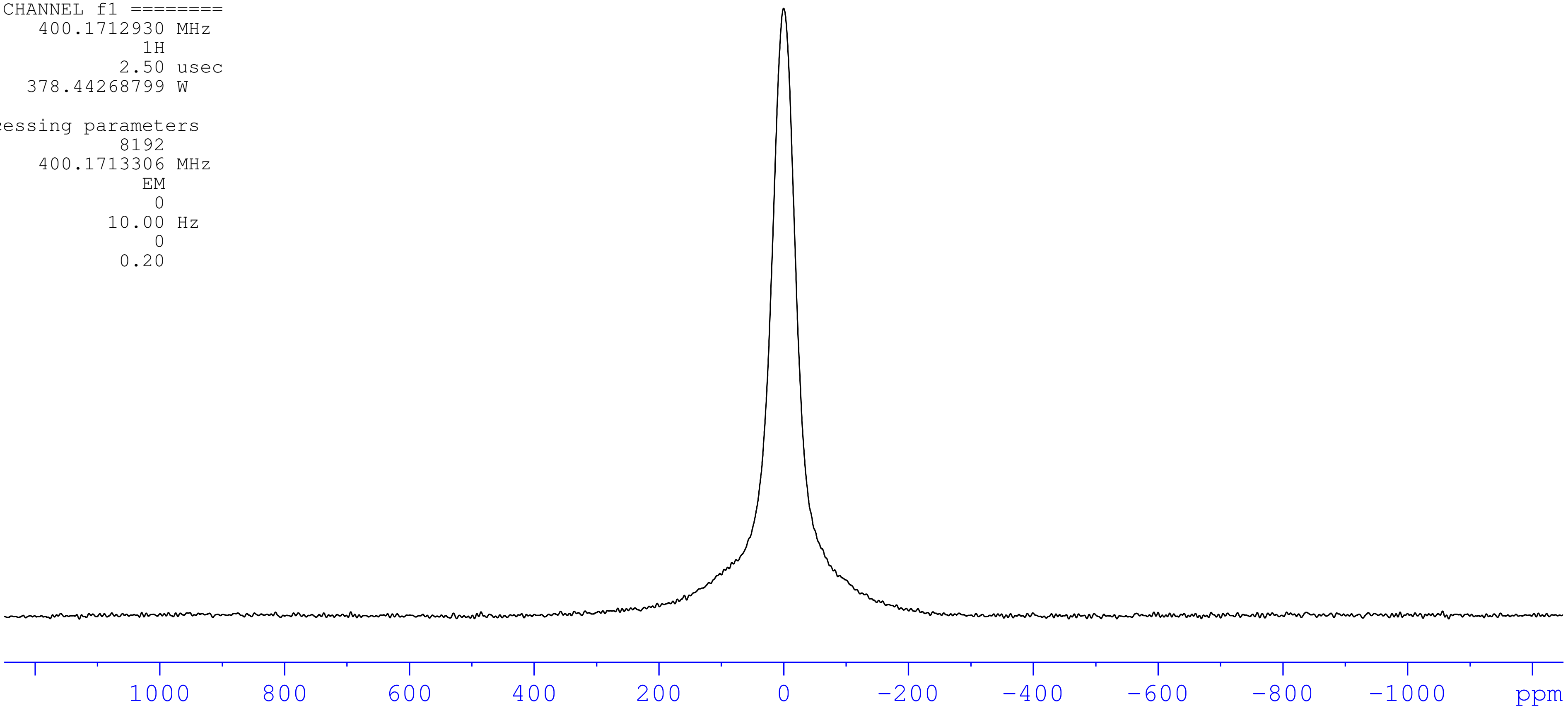

Current Data Parameters
NAME jse_20240130
EXPNO 8
PROCNO 1

F2 - Acquisition Parameters
Date_ 20240131
PROBHD 5 mm PE BB/1H/
PULPROG satrect1se
NS 8
SWH 1000000.000 Hz
AQ 0.0005120 sec
TE 298.0 K
D1 1.00000000 sec
D6 0.00004625 sec
D7 0.00002750 sec
D20 0.00040000 sec
L20 64
VDLIST Recovery_0.1_102.4_16

======== CHANNEL f1 ========
SFO1 400.1712930 MHz
NUC1 1H
P1 2.50 usec
PLW1 378.44268799 W

F1 - Acquisition parameters
TD 16
SFO1 400.1713 MHz
FIDRES 500.000000 Hz
SW 9.996 ppm
FnMODE QF

F2 - Processing parameters
SI 8192
SF 400.1713306 MHz
WDW no
SSB 0
LB 0 Hz
GB 0
PC 0.20

F1 - Processing parameters
SI 16
MC2 QF
SF 400.1700000 MHz
WDW no
SSB 0
LB 0 Hz
GB 0

2624-10340 SYT0043a S-CTA CdCl4 @ static / -100 to +120 C
-90 C

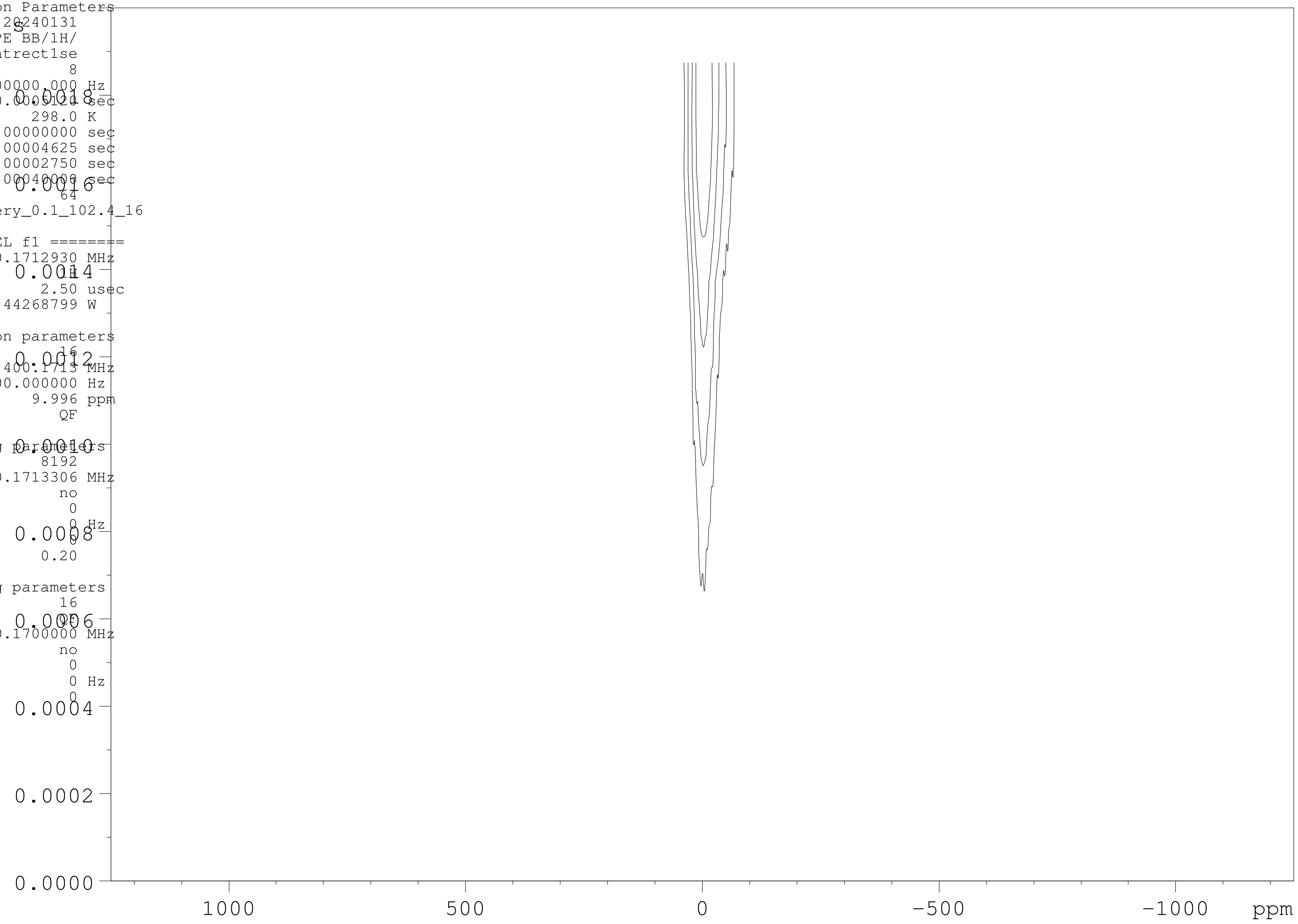

2624-10340 SYT0043a S-CTA CdCl4 @ static / -100 to +120 C
-90 C

Current Data Parameters
NAME jse_20240130
EXPNO 9
PROCNO 1

F2 - Acquisition Parameters
Date_ 20240131
PROBHD 5 mm PE BB/1H/
PULPROG t1rho_solidecho
NS 8
SWH 1000000.000 Hz
AQ 0.0005120 sec
TE 226.8 K
D1 58.43999863 sec
D6 0.00005000 sec
D7 0.0000275[illegible] sec

======== CHANNEL f1 ========
SFO1 400.1712930 MHz
NUC1 1H
P1 2.50 usec
PLW1 378.44268799 W
PLW2 94.62400055 W
VPLIST 100u_52000u_16

F1 - Acquisition parameters
TD 8
SFO1 400.1713 MHz
FIDRES 1000.000000 Hz
SW 9.996 ppm
FnMODE QF

F2 - Processing parameters
SI 8192
SF 400.1713306 MHz
WDW no
SSB [illegible]
LB 0 Hz
GB 0
PC 0.20

F1 - Processing parameters
SI 16
MC2 QF
SF 400.1700000 MHz
WDW no
SSB 0
LB 0 Hz
GB 0

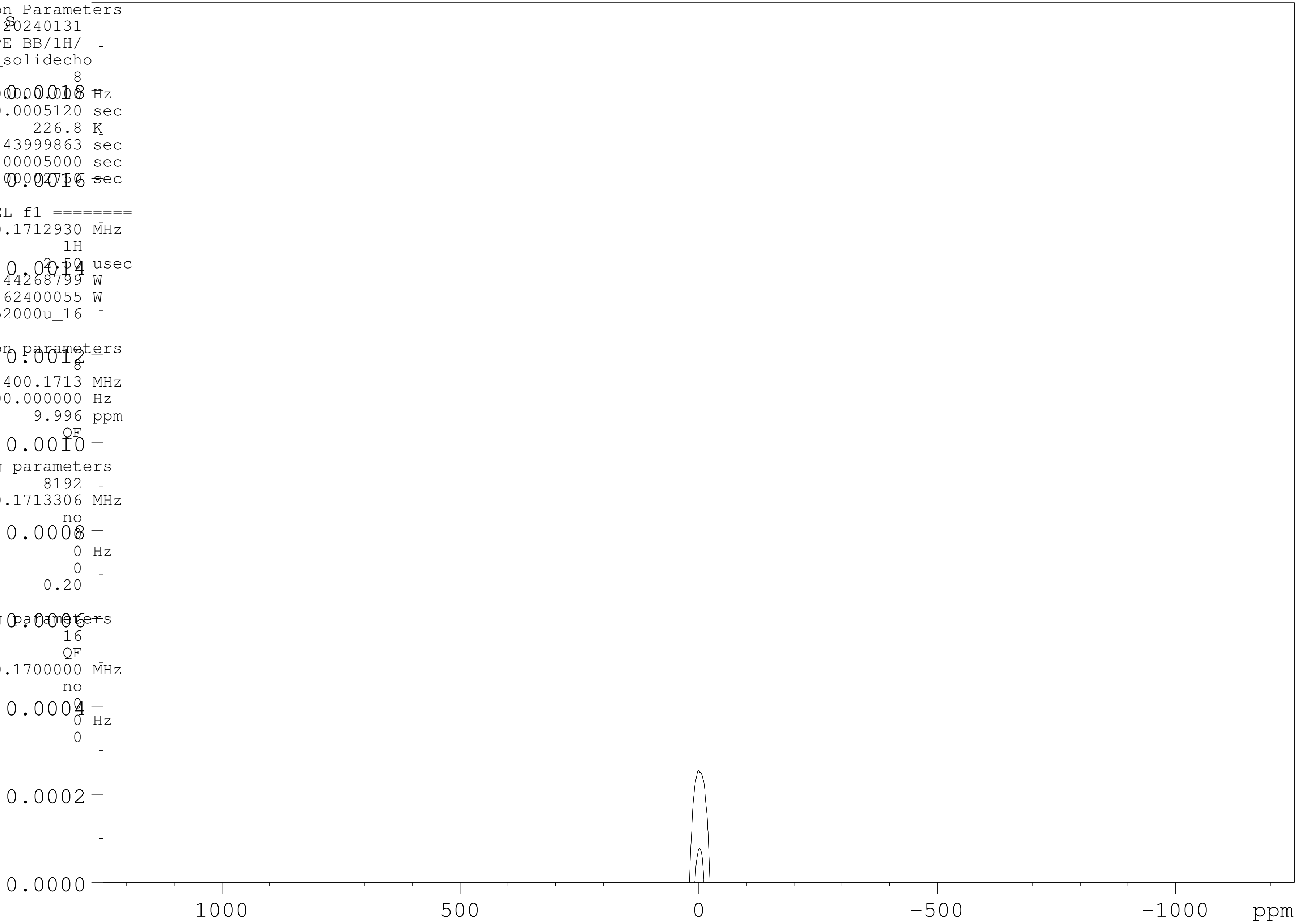

Current Data Parameters
NAME jse_20240130
EXPNO 11
PROCNO 1

F2 - Acquisition Parameters
Date_ 20240131
PROBHD 5 mm PE BB/1H/
PULPROG solidecho
NS 8
SWH 1000000.000 Hz
AQ 0.0005120 sec
TE 213.4 K
D1 24.79999924 sec
D6 0.00005000 sec
D7 0.00002750 sec

======== CHANNEL f1 ========
SFO1 400.1712930 MHz
NUC1 1H
P1 2.50 usec
PLW1 378.44268799 W

F2 - Processing parameters
SI 8192
SF 400.1713306 MHz
WDW EM
SSB 0
LB 10.00 Hz
GB 0
PC 0.20

2624-10340 SYT0043a S-CTA CdCl4 @ static / -100 to +120 C
-80 C

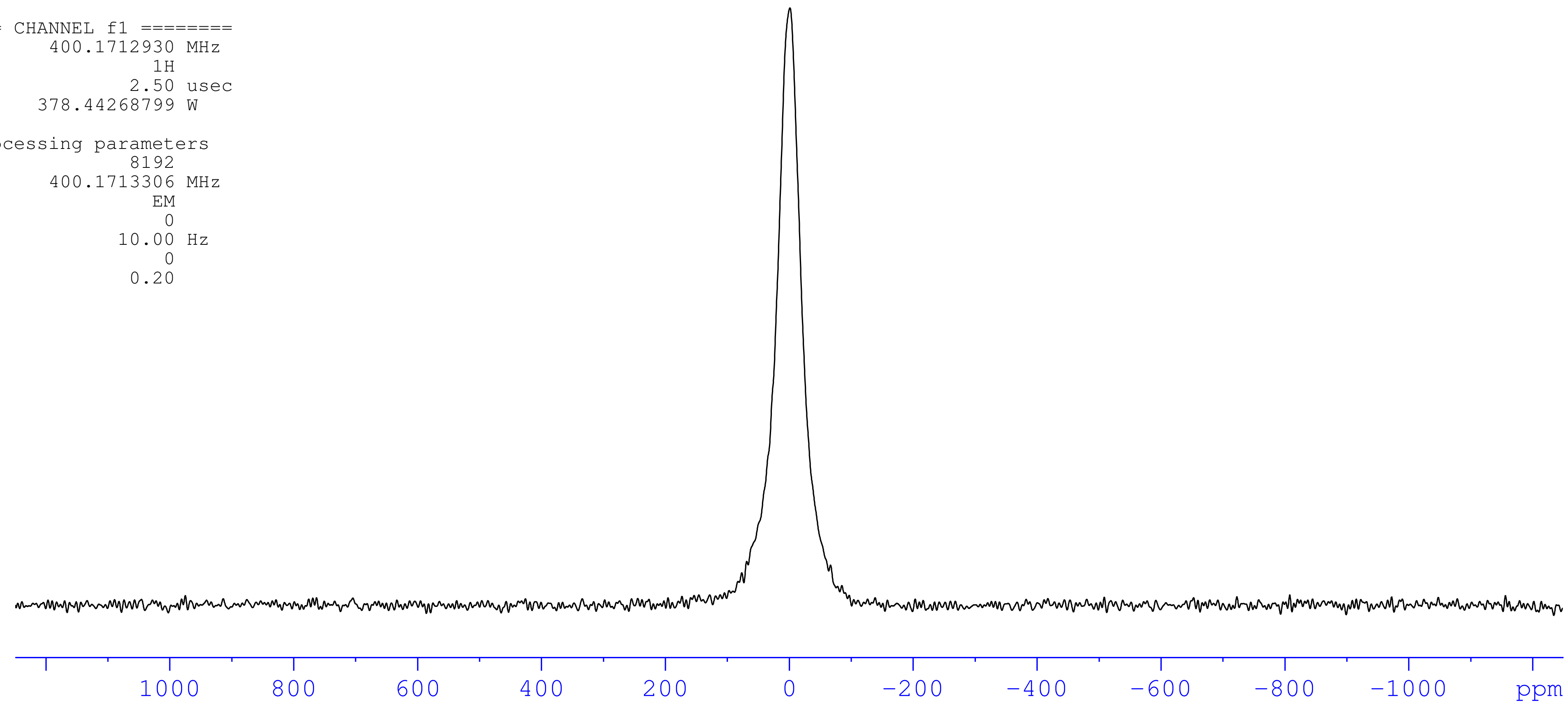

Current Data Parameters
NAME jse_20240130
EXPNO 12
PROCNO 1

F2 - Acquisition Parameters
Date_ 20240131
PROBHD 5 mm PE BB/1H/
PULPROG zg
NS 4
SWH 1000000.000 Hz
AQ 0.0005120 sec
TE 294.0 K
D1 24.79999924 sec
TD0 1

======== CHANNEL f1 ========
SFO1 400.1712930 MHz
NUC1 1H
P1 2.50 usec
PLW1 378.44268799 W

F2 - Processing parameters
SI 8192
SF 400.1713306 MHz
WDW EM
SSB 0
LB 0 Hz
GB 0
PC 0.20

2624-10340 SYT0043a S-CTA CdCl4 @ static / -100 to +120 C
-80 C

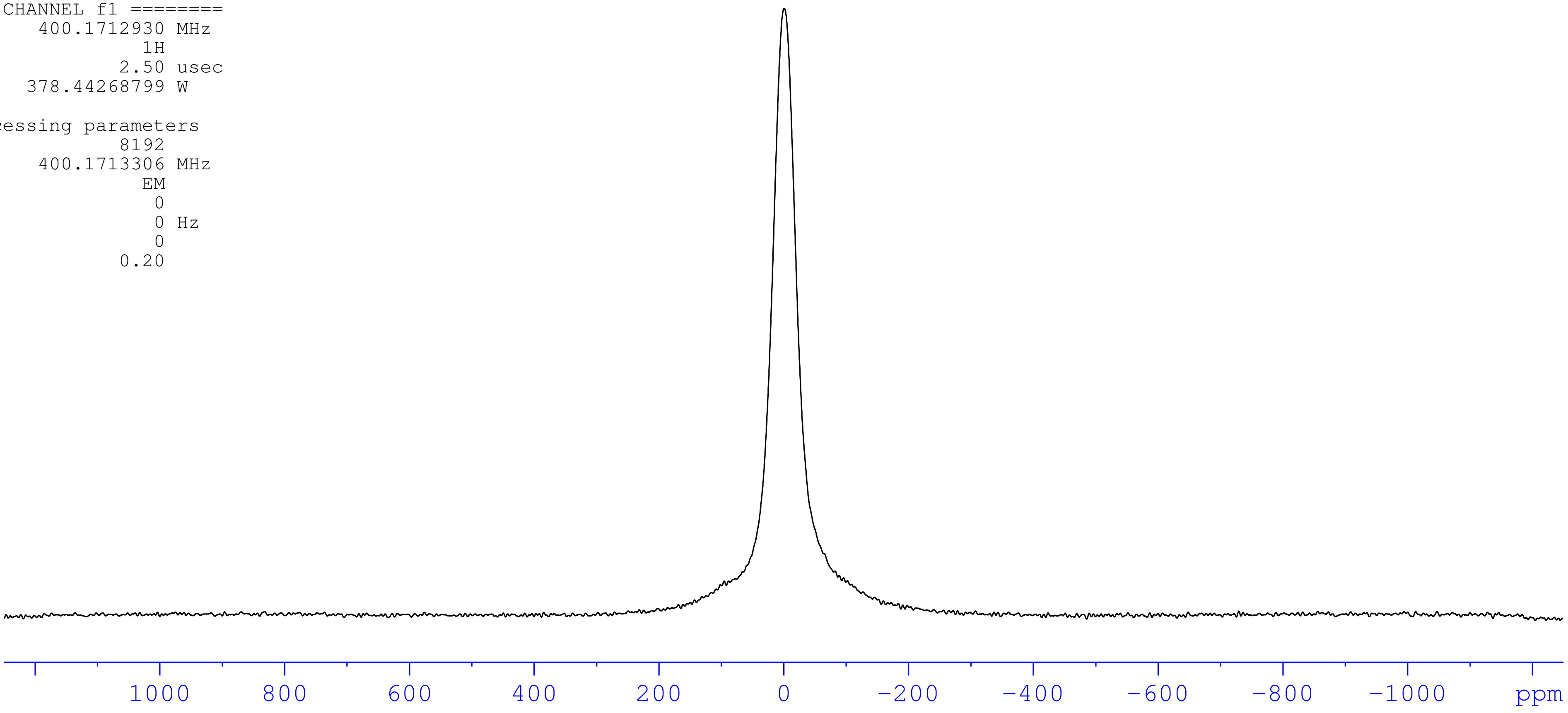

2624-10340 SYT0043a S-CTA CdCl4 @ static / -100 to +120 C
-80 C

```
Current Data Parameters
NAME        jse_20240130
EXPNO                 13
PROCNO                 1

F2 - Acquisition Parameters
Date_           20240131
PROBHD   5 mm PE BB/1H/
PULPROG       satrect1se
NS                     8
SWH        1000000.000 Hz
AQ          0.0005120 sec
TE              298.0 K
D1          1.00000000 sec
D6          0.00004625 sec
D7          0.00002750 sec
D20         0.00040000 sec
L20                   64
VDLIST   Recovery_0.1_102.4_16

======== CHANNEL f1 ========
SFO1       400.1712930 MHz
NUC1                  1H
P1                  2.50 usec
PLW1      378.44268799 W

F1 - Acquisition parameters
TD                    16
SFO1           400.1713 MHz
FIDRES       500.000000 Hz
SW                 9.996 ppm
FnMODE                QF

F2 - Processing parameters
SI                  8192
SF           400.1713306 MHz
WDW                   no
SSB                    0
LB                     0 Hz
GB                     0
PC                  0.20

F1 - Processing parameters
SI                    16
MC2                   QF
SF           400.1700000 MHz
WDW                   no
SSB                    0
LB                     0 Hz
GB                     0
```

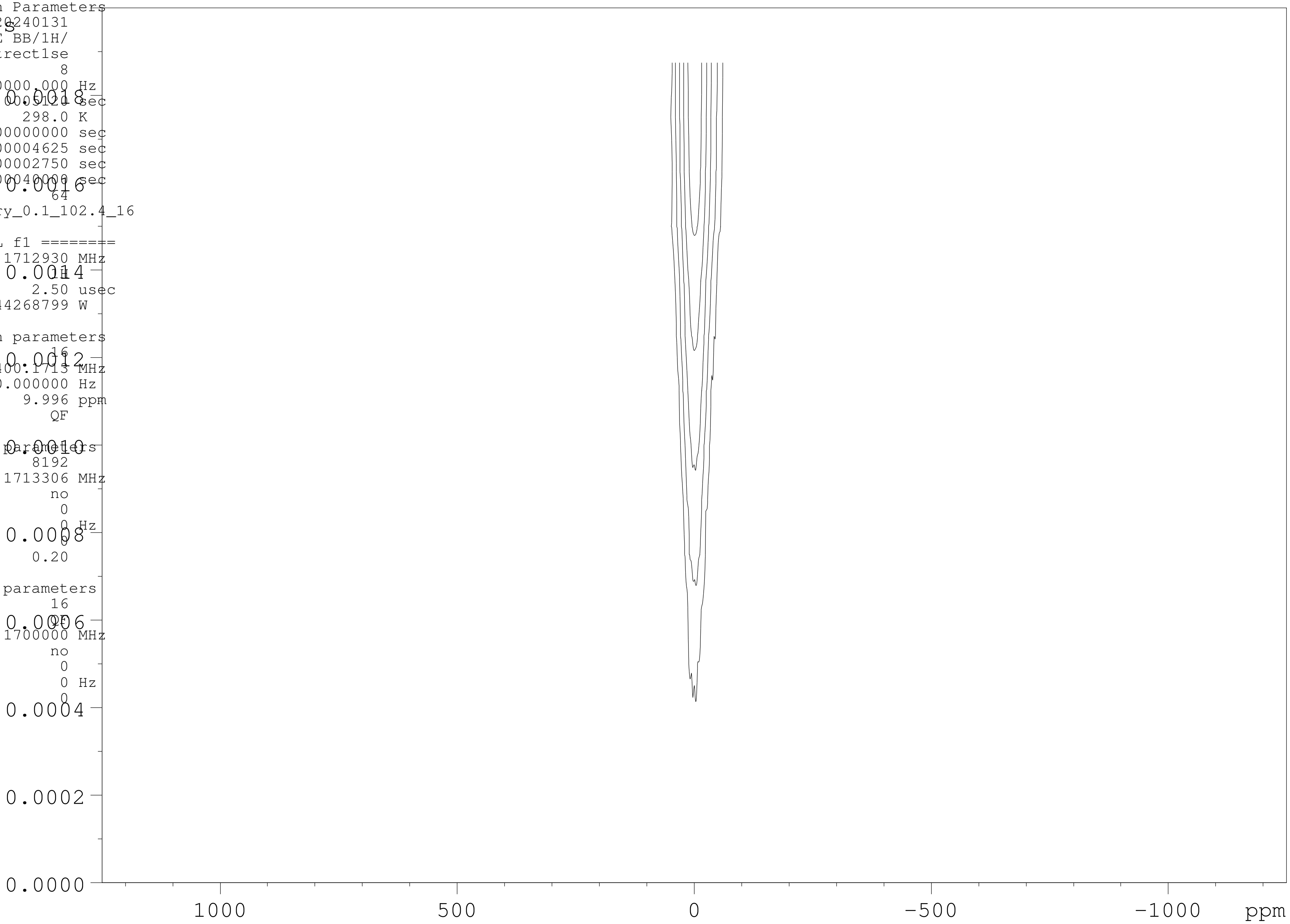

Current Data Parameters
NAME jse_20240130
EXPNO 14
PROCNO 1

2624-10340 SYT0043a S-CTA CdCl4 @ static / -100 to +120 C
-80 C

F2 - Acquisition Parameters
Date_ 20240131
PROBHD 5 mm PE BB/1H/
PULPROG t1rho_solidecho
NS 8
SWH 100000.000 Hz
AQ 0.0005120 sec
TE 233.6 K
D1 24.79999924 sec
D6 0.00005000 sec
D7 0.00002750 sec

======== CHANNEL f1 ========
SFO1 400.1712930 MHz
NUC1 1H
P1 2.50 usec
PLW1 378.44268799 W
PLW2 94.62400055 W
VPLIST 100u_52000u_16

F1 - Acquisition parameters
TD 8
SFO1 400.1713 MHz
FIDRES 1000.000000 Hz
SW 9.996 ppm
FnMODE QF

F2 - Processing parameters
SI 8192
SF 400.1713306 MHz
WDW no
SSB 0
LB 0 Hz
GB 0
PC 0.20

F1 - Processing parameters
SI 16
MC2 QF
SF 400.1700000 MHz
WDW no
SSB 0
LB 0 Hz
GB 0

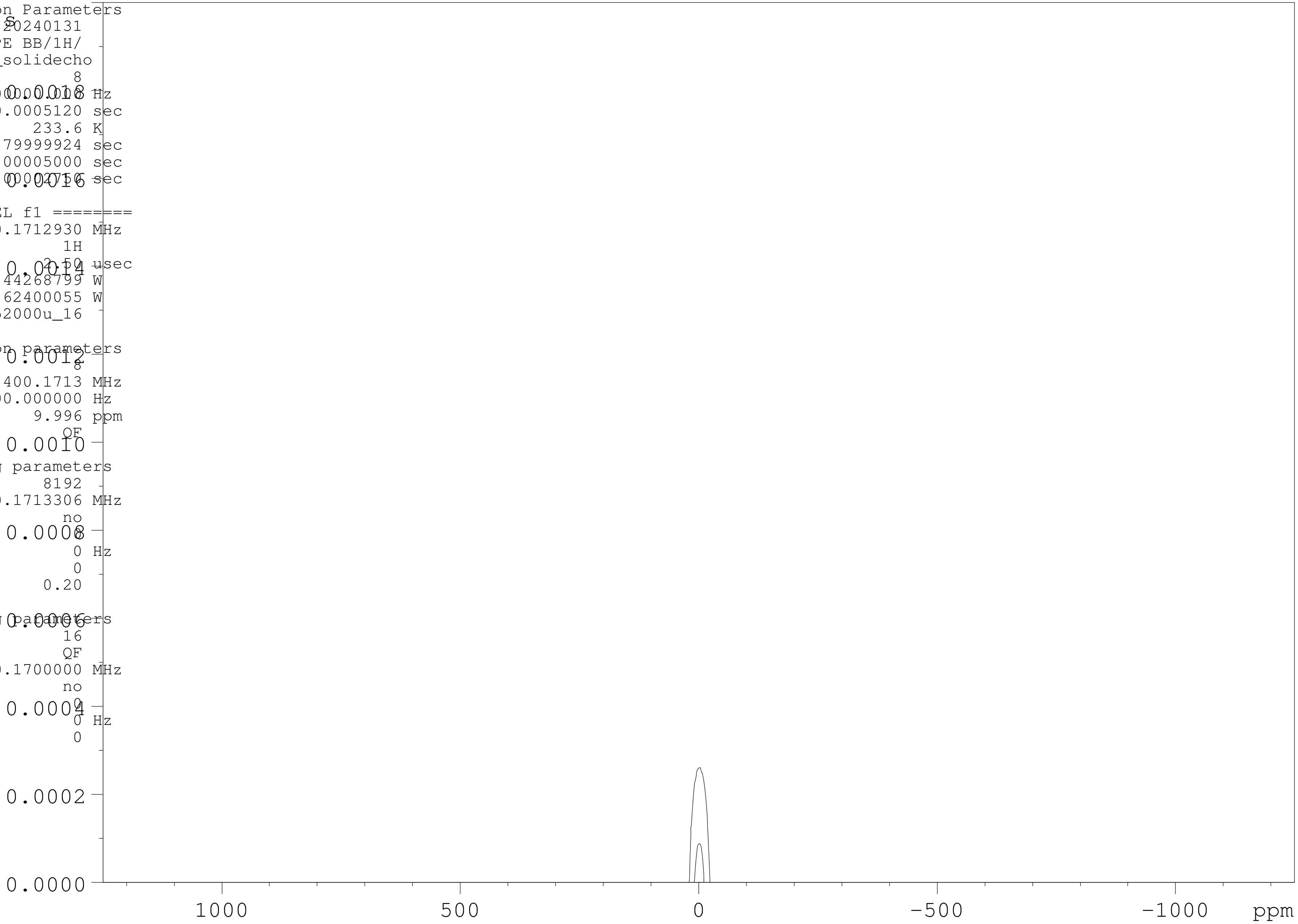

Current Data Parameters
NAME jse_20240130
EXPNO 16
PROCNO 1

F2 - Acquisition Parameters
Date_ 20240131
PROBHD 5 mm PE BB/1H/
PULPROG solidecho
NS 8
SWH 1000000.000 Hz
AQ 0.0005120 sec
TE 213.4 K
D1 24.79999924 sec
D6 0.00005000 sec
D7 0.00002750 sec

======== CHANNEL f1 ========
SFO1 400.1712930 MHz
NUC1 1H
P1 2.50 usec
PLW1 378.44268799 W

F2 - Processing parameters
SI 8192
SF 400.1713306 MHz
WDW EM
SSB 0
LB 10.00 Hz
GB 0
PC 0.20

2624-10340 SYT0043a S-CTA CdCl4 @ static / -100 to +120 C
-70 C

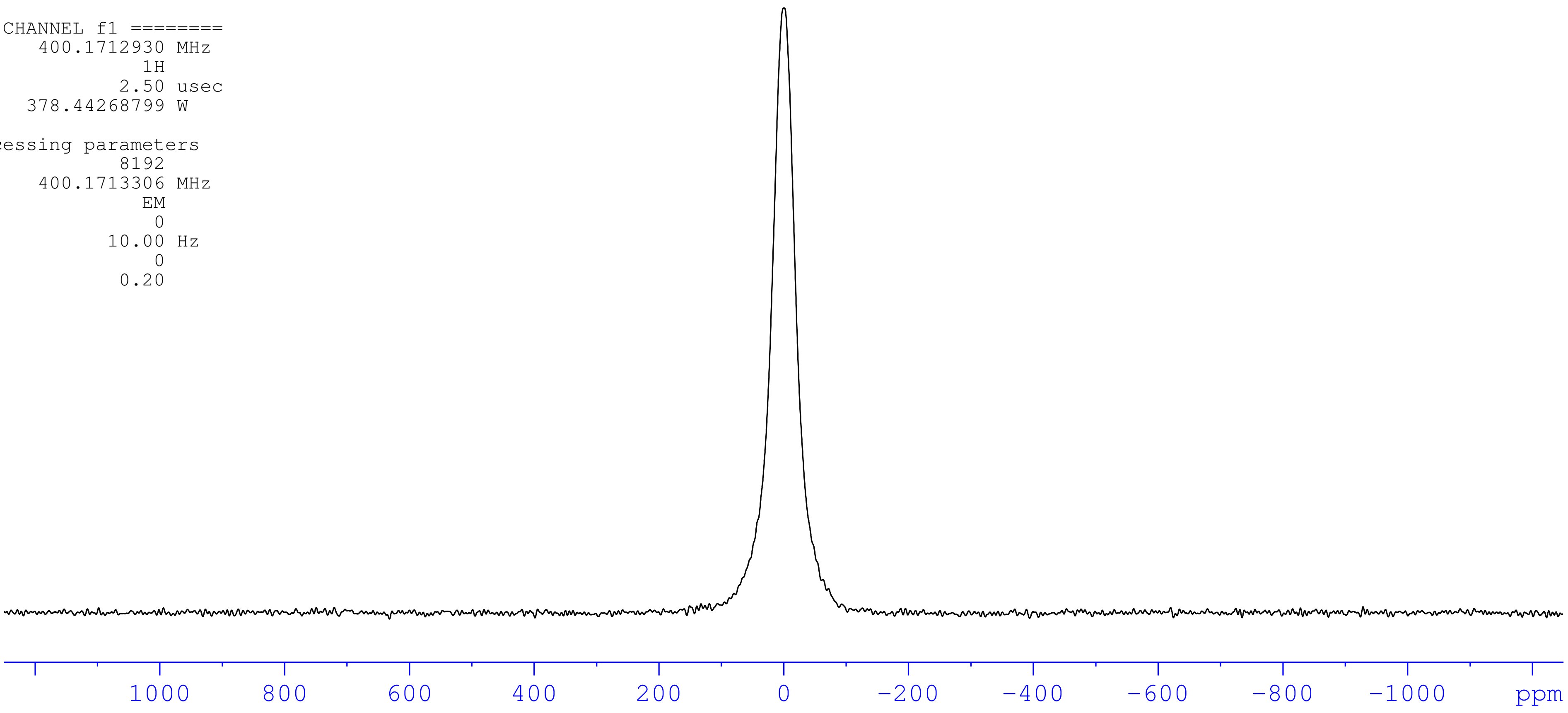

```
Current Data Parameters
NAME        jse_20240130
EXPNO                 17
PROCNO                 1

F2 - Acquisition Parameters
Date_           20240131
PROBHD   5 mm PE BB/1H/
PULPROG               zg
NS                     4
SWH         1000000.000 Hz
AQ            0.0005120 sec
TE                294.0 K
D1          24.79999924 sec
TD0                    1

======== CHANNEL f1 ========
SFO1        400.1712930 MHz
NUC1                  1H
P1                  2.50 usec
PLW1        378.44268799 W

F2 - Processing parameters
SI                  8192
SF          400.1713306 MHz
WDW                   EM
SSB                    0
LB                 10.00 Hz
GB                     0
PC                  0.20
```

2624-10340 SYT0043a S-CTA CdCl4 @ static / -100 to +120 C
-70 C

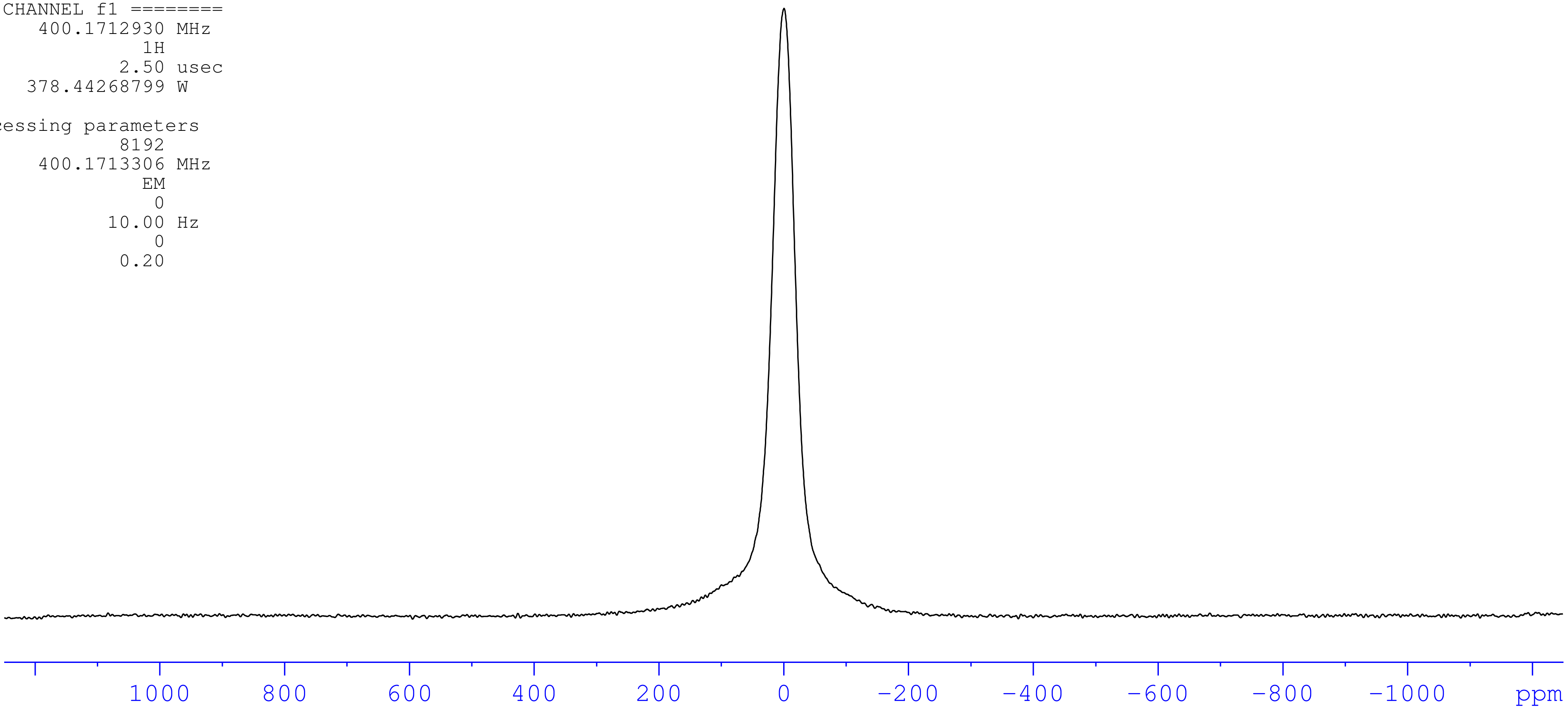

Current Data Parameters
NAME jse_20240130
EXPNO 18
PROCNO 1

F2 - Acquisition Parameters
Date_ 20240131
PROBHD 5 mm PE BB/1H/
PULPROG satrect1se
NS 8
SWH 1000000.000 Hz
AQ 0.0005120 sec
TE 298.0 K
D1 1.00000000 sec
D6 0.00004625 sec
D7 0.00002750 sec
D20 0.00040000 sec
L20 64
VDLIST Recovery_0.2_51.2_16

======== CHANNEL f1 ========
SFO1 400.1712930 MHz
NUC1 1H
P1 2.50 usec
PLW1 378.44268799 W

F1 - Acquisition parameters
TD 16
SFO1 400.1713 MHz
FIDRES 500.000000 Hz
SW 9.996 ppm
FnMODE QF

F2 - Processing parameters
SI 8192
SF 400.1713306 MHz
WDW no
SSB 0
LB 0 Hz
GB 0
PC 0.20

F1 - Processing parameters
SI 16
MC2 QF
SF 400.1700000 MHz
WDW no
SSB 0
LB 0 Hz
GB 0

2624-10340 SYT0043a S-CTA CdCl4 @ static / -100 to +120 C
-70 C

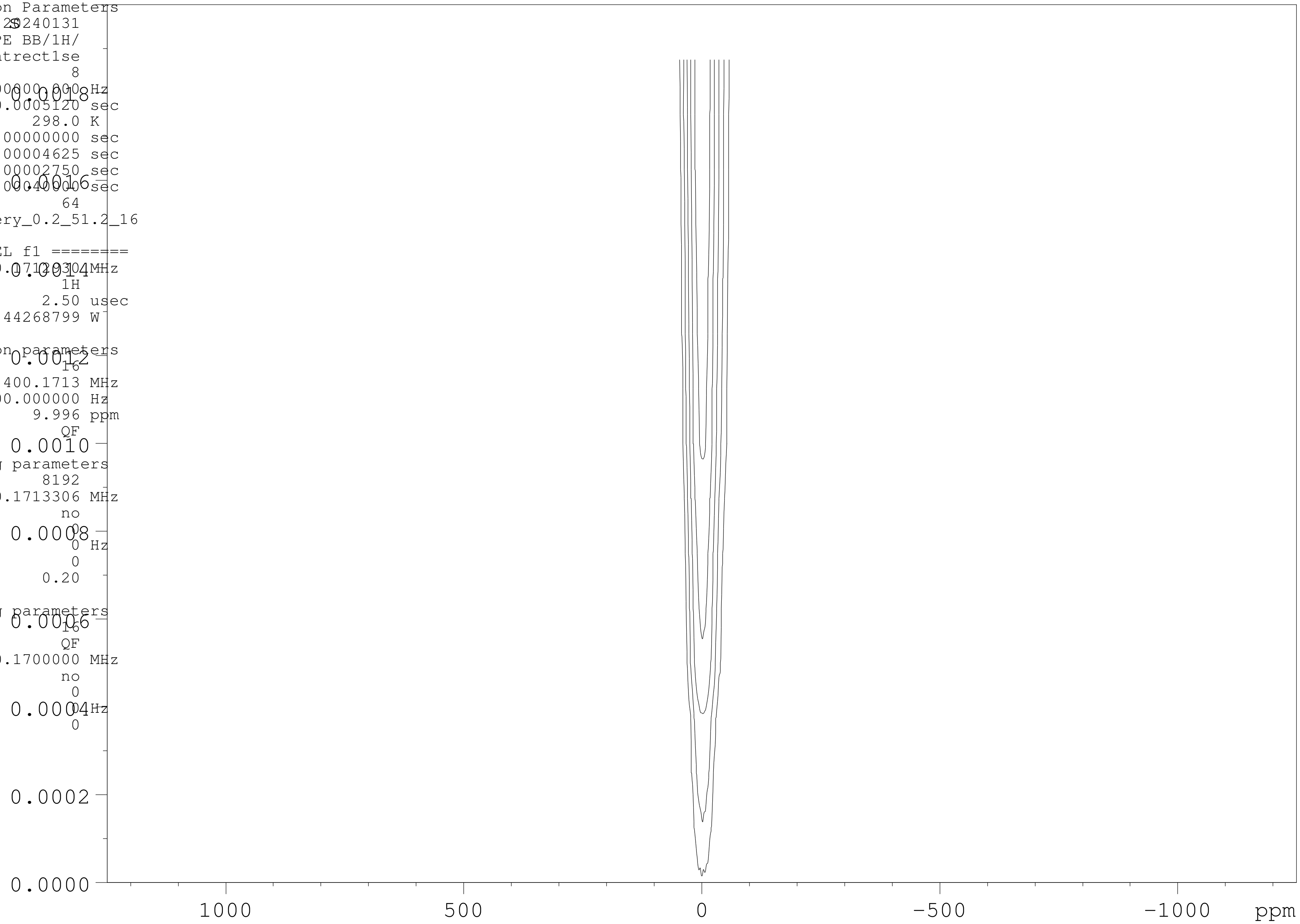

2624-10340 SYT0043a S-CTA CdCl4 @ static / -100 to +120 C
-70 C

Current Data Parameters
NAME jse_20240130
EXPNO 19
PROCNO 1

F2 - Acquisition Parameters
Date_ 20240131
PROBHD 5 mm PE BB/1H/
PULPROG t1rho_solidecho
NS 8
SWH 1000000.000 Hz
AQ 0.0005120 sec
TE 240.3 K
D1 24.79999924 sec
D6 0.00005000 sec
D7 0.00002756 sec

======== CHANNEL f1 ========
SFO1 400.1712930 MHz
NUC1 1H
P1 2.50 usec
PLW1 378.44268799 W
PLW2 94.62400055 W
VPLIST 100u_52000u_16

F1 - Acquisition parameters
TD 8
SFO1 400.1713 MHz
FIDRES 1000.000000 Hz
SW 9.996 ppm
FnMODE QF

F2 - Processing parameters
SI 8192
SF 400.1713306 MHz
WDW no
SSB 0
LB 0 Hz
GB 0
PC 0.20

F1 - Processing parameters
SI 16
MC2 QF
SF 400.1700000 MHz
WDW no
SSB 0
LB 0 Hz
GB 0

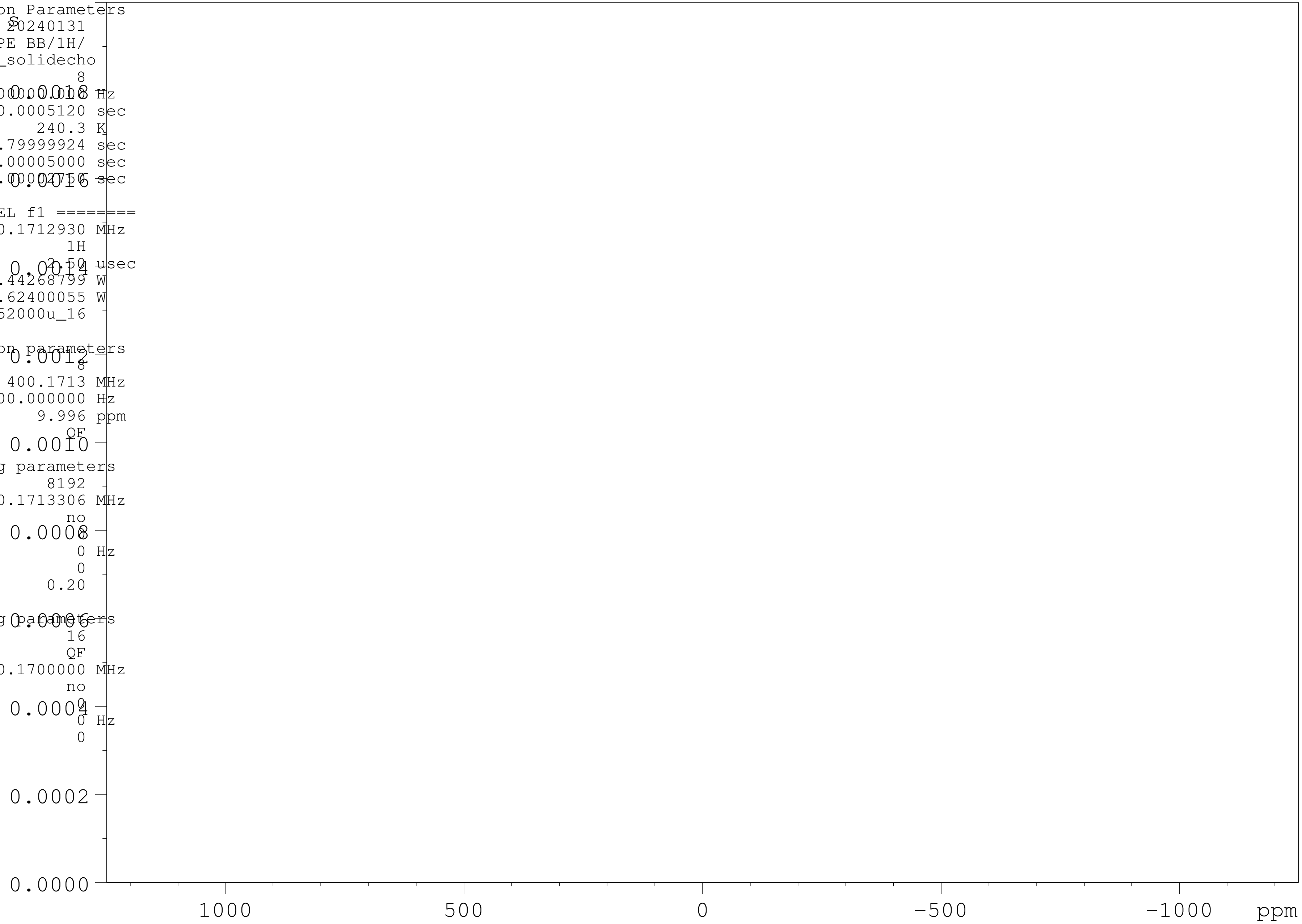

Current Data Parameters
NAME jse_20240130
EXPNO 21
PROCNO 1

F2 - Acquisition Parameters
Date_ 20240131
PROBHD 5 mm PE BB/1H/
PULPROG solidecho
NS 8
SWH 1000000.000 Hz
AQ 0.0005120 sec
TE 213.4 K
D1 11.19999981 sec
D6 0.00005000 sec
D7 0.00002750 sec

======== CHANNEL f1 ========
SFO1 400.1712930 MHz
NUC1 1H
P1 2.50 usec
PLW1 378.44268799 W

F2 - Processing parameters
SI 8192
SF 400.1713306 MHz
WDW EM
SSB 0
LB 10.00 Hz
GB 0
PC 0.20

2624-10340 SYT0043a S-CTA CdCl4 @ static / -100 to +120 C
-60 C

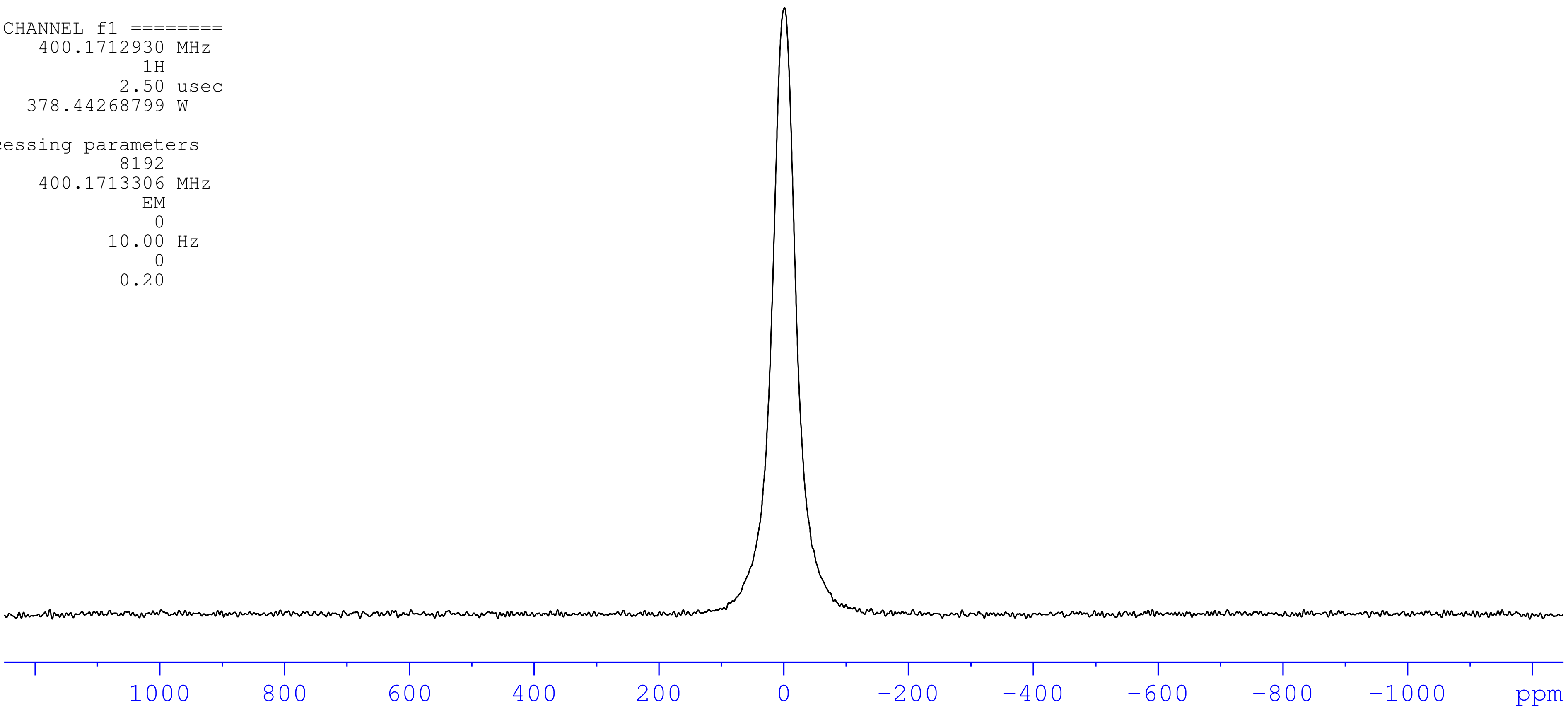

```
Current Data Parameters
NAME       jse_20240130
EXPNO                22
PROCNO                1

F2 - Acquisition Parameters
Date_          20240131
PROBHD    5 mm PE BB/1H/
PULPROG              zg
NS                    4
SWH         1000000.000 Hz
AQ            0.0005120 sec
TE                294.0 K
D1          11.19999981 sec
TD0                   1

======== CHANNEL f1 ========
SFO1        400.1712930 MHz
NUC1                 1H
P1                 2.50 usec
PLW1       378.44268799 W

F2 - Processing parameters
SI                 8192
SF          400.1713306 MHz
WDW                  EM
SSB                   0
LB                10.00 Hz
GB                    0
PC                 0.20
```

2624-10340 SYT0043a S-CTA CdCl4 @ static / -100 to +120 C
-60 C

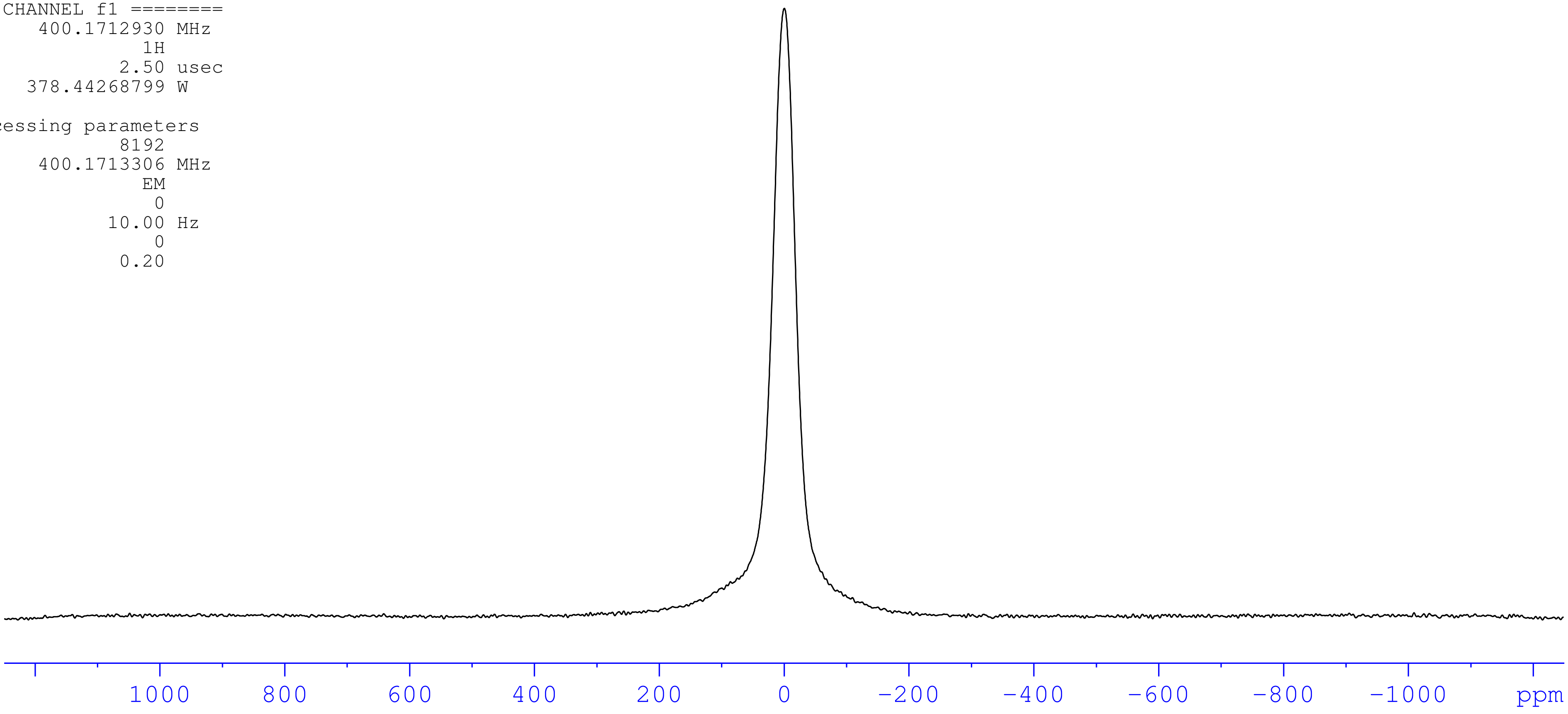

Current Data Parameters
NAME jse_20240130
EXPNO 23
PROCNO 1

F2 - Acquisition Parameters
Date_ 20240131
PROBHD 5 mm PE BB/1H/
PULPROG satrect1se
NS 8
SWH 1000000.000 Hz
AQ 0.0005120 sec
TE 298.0 K
D1 1.00000000 sec
D6 0.00004625 sec
D7 0.00002750 sec
D20 0.00040000 sec
L20 64
VDLIST Recovery_0.2_51.2_16

======== CHANNEL f1 ========
SFO1 400.1712930 MHz
NUC1 1H
P1 2.50 usec
PLW1 378.44268799 W

F1 - Acquisition parameters
TD 16
SFO1 400.1713 MHz
FIDRES 500.000000 Hz
SW 9.996 ppm
FnMODE QF

F2 - Processing parameters
SI 8192
SF 400.1713306 MHz
WDW no
SSB 0
LB 0 Hz
GB 0
PC 0.20

F1 - Processing parameters
SI 16
MC2 QF
SF 400.1700000 MHz
WDW no
SSB 0
LB 0 Hz
GB 0

2624-10340 SYT0043a S-CTA CdCl4 @ static / -100 to +120 C
-60 C

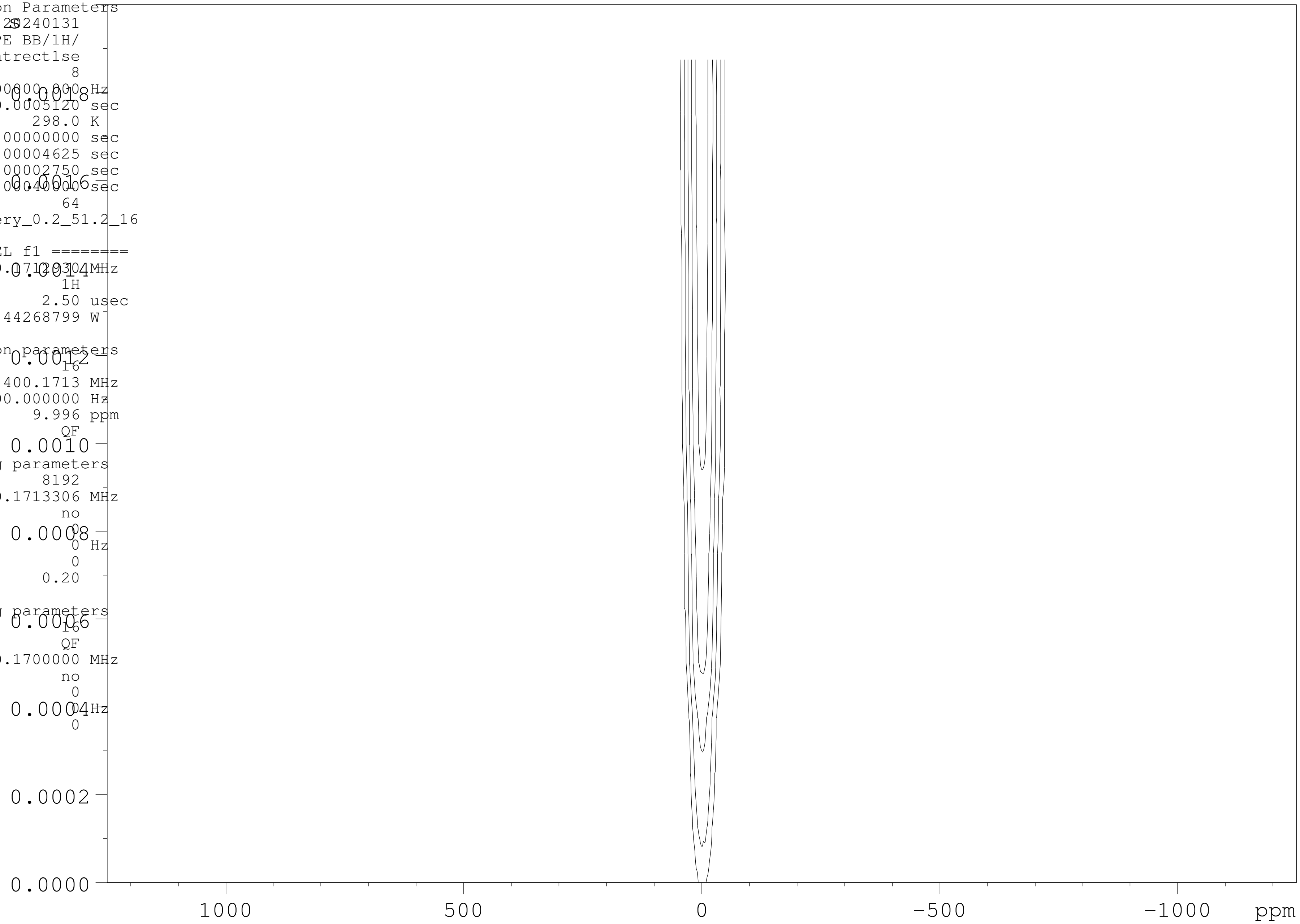

2624-10340 SYT0043a S-CTA CdCl4 @ static / -100 to +120 C
-60 C

```
Current Data Parameters
NAME       jse_20240130
EXPNO                24
PROCNO                1

F2 - Acquisition Parameters
Date_          20240131
PROBHD   5 mm PE BB/1H/
PULPROG  t1rho_solidecho
NS                    8
SWH         1000000.000 Hz
AQ            0.0005120 sec
TE                247.0 K
D1          11.19999981 sec
D6           0.00005000 sec
D7           0.00002750 sec

======== CHANNEL f1 ========
SFO1        400.1712930 MHz
NUC1                 1H
P1                 2.50 usec
PLW1       378.44268799 W
PLW2        94.62400055 W
VPLIST   100u_52000u_16

F1 - Acquisition parameters
TD                    8
SFO1           400.1713 MHz
FIDRES      1000.000000 Hz
SW                9.996 ppm
FnMODE               QF

F2 - Processing parameters
SI                 8192
SF          400.1713306 MHz
WDW                  no
SSB                   0
LB                    0 Hz
GB                    0
PC                 0.20

F1 - Processing parameters
SI                   16
MC2                  QF
SF          400.1700000 MHz
WDW                  no
SSB                   0
LB                    0 Hz
GB                    0
```

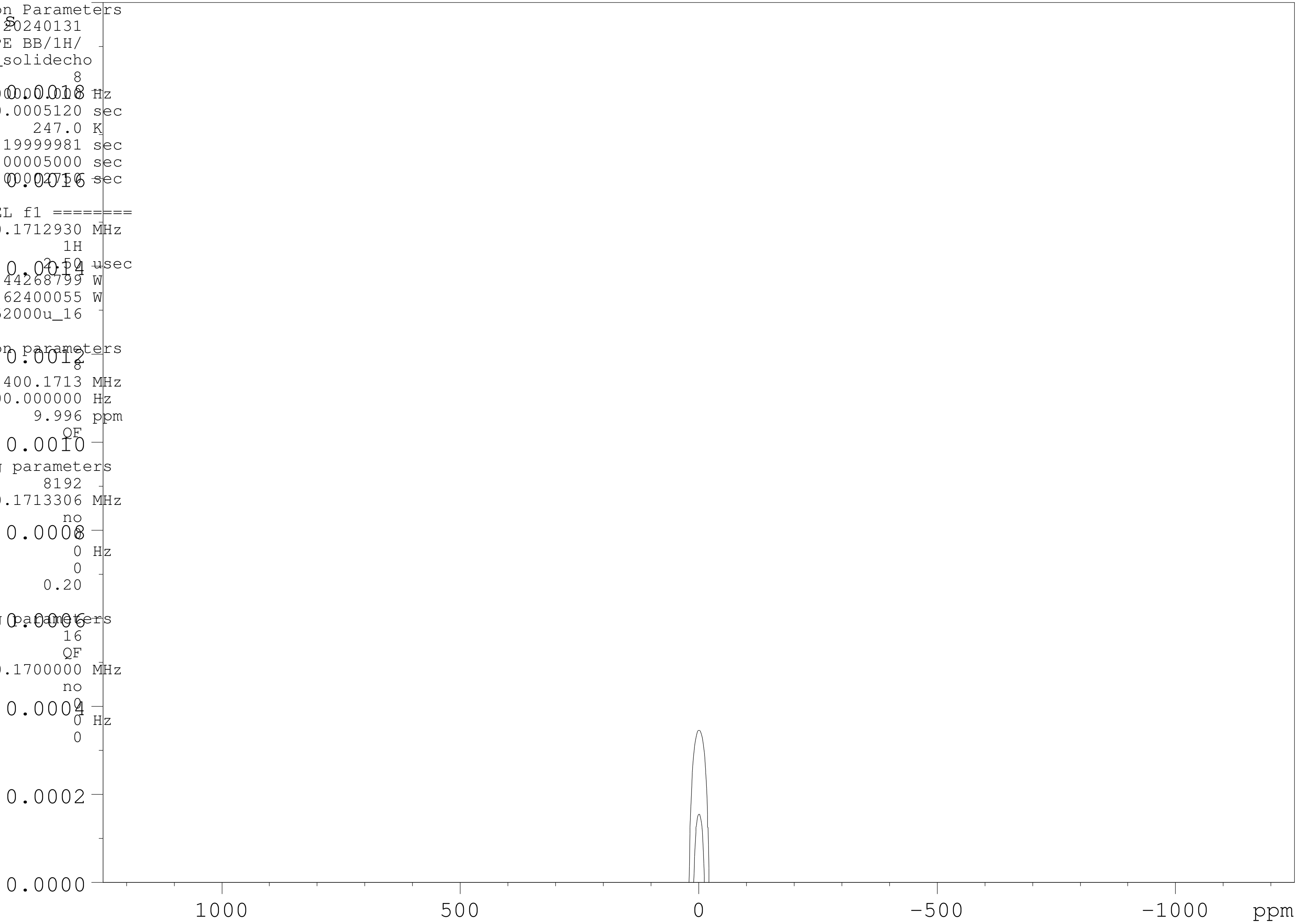

Current Data Parameters
NAME jse_20240130
EXPNO 26
PROCNO 1

F2 - Acquisition Parameters
Date_ 20240131
PROBHD 5 mm PE BB/1H/
PULPROG solidecho
NS 8
SWH 1000000.000 Hz
AQ 0.0005120 sec
TE 213.4 K
D1 11.19999981 sec
D6 0.00005000 sec
D7 0.00002750 sec

======== CHANNEL f1 ========
SFO1 400.1712930 MHz
NUC1 1H
P1 2.50 usec
PLW1 378.44268799 W

F2 - Processing parameters
SI 8192
SF 400.1713306 MHz
WDW EM
SSB 0
LB 10.00 Hz
GB 0
PC 0.20

2624-10340 SYT0043a S-CTA CdCl4 @ static / -100 to +120 C
-50 C

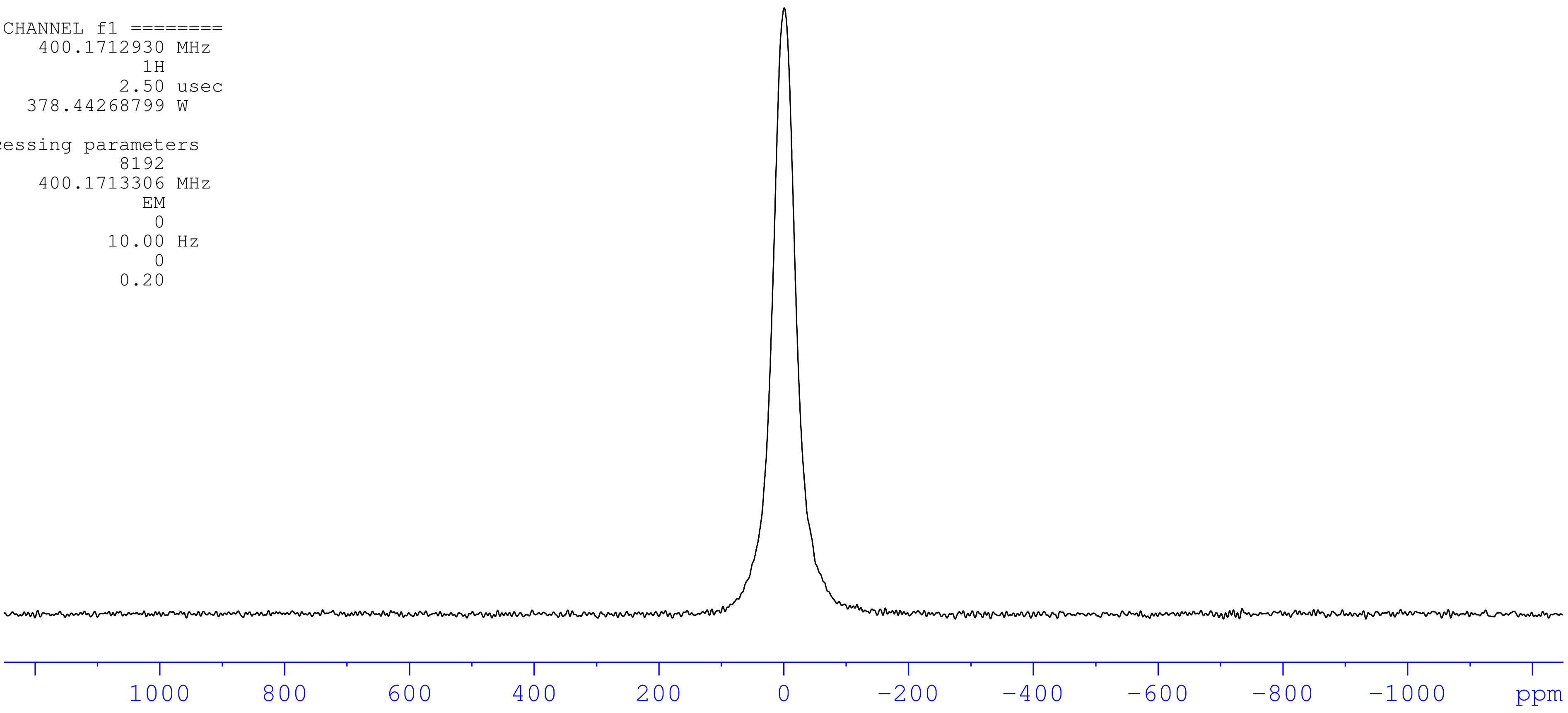

Current Data Parameters
NAME jse_20240130
EXPNO 27
PROCNO 1

F2 - Acquisition Parameters
Date_ 20240131
PROBHD 5 mm PE BB/1H/
PULPROG zg
NS 4
SWH 1000000.000 Hz
AQ 0.0005120 sec
TE 294.0 K
D1 11.19999981 sec
TD0 1

======== CHANNEL f1 ========
SFO1 400.1712930 MHz
NUC1 1H
P1 2.50 usec
PLW1 378.44268799 W

F2 - Processing parameters
SI 8192
SF 400.1713306 MHz
WDW EM
SSB 0
LB 10.00 Hz
GB 0
PC 0.20

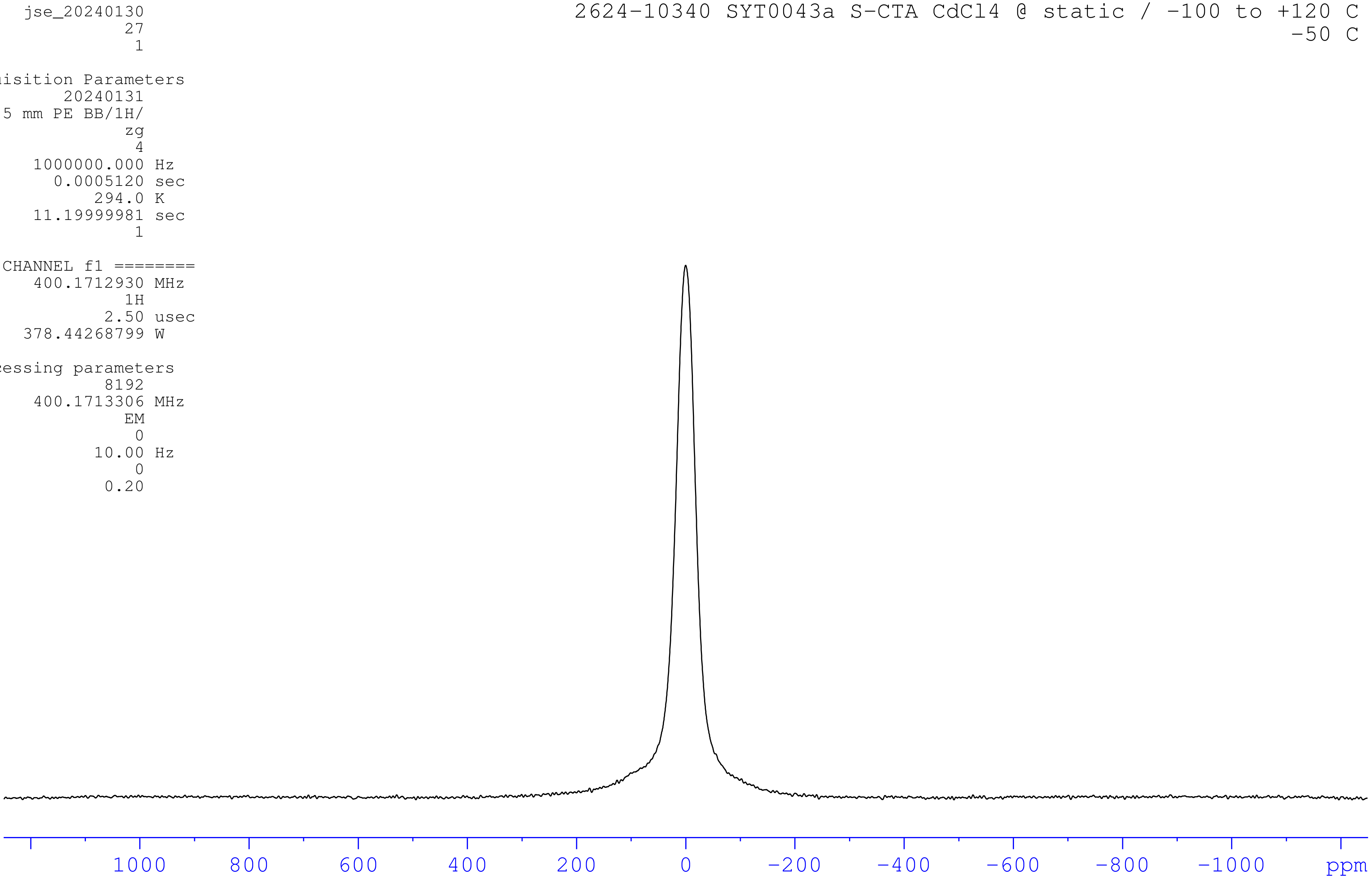

Current Data Parameters
NAME jse_20240130
EXPNO 28
PROCNO 1

F2 - Acquisition Parameters
Date_ 20240131
PROBHD 5 mm PE BB/1H/
PULPROG satrect1se
NS 8
SWH 1000000.000 Hz
AQ 0.0005120 sec
TE 298.0 K
D1 1.00000000 sec
D6 0.00004625 sec
D7 0.00002750 sec
D20 0.00040000 sec
L20 64
VDLIST Recovery_0.2_51.2_16

======== CHANNEL f1 ========
SFO1 400.1712930 MHz
NUC1 1H
P1 2.50 usec
PLW1 378.44268799 W

F1 - Acquisition parameters
TD 16
SFO1 400.1713 MHz
FIDRES 500.000000 Hz
SW 9.996 ppm
FnMODE QF

F2 - Processing parameters
SI 8192
SF 400.1713306 MHz
WDW no
SSB 0
LB 0 Hz
GB 0
PC 0.20

F1 - Processing parameters
SI 16
MC2 QF
SF 400.1700000 MHz
WDW no
SSB 0
LB 0 Hz
GB 0

2624-10340 SYT0043a S-CTA CdCl4 @ static / -100 to +120 C
-50 C

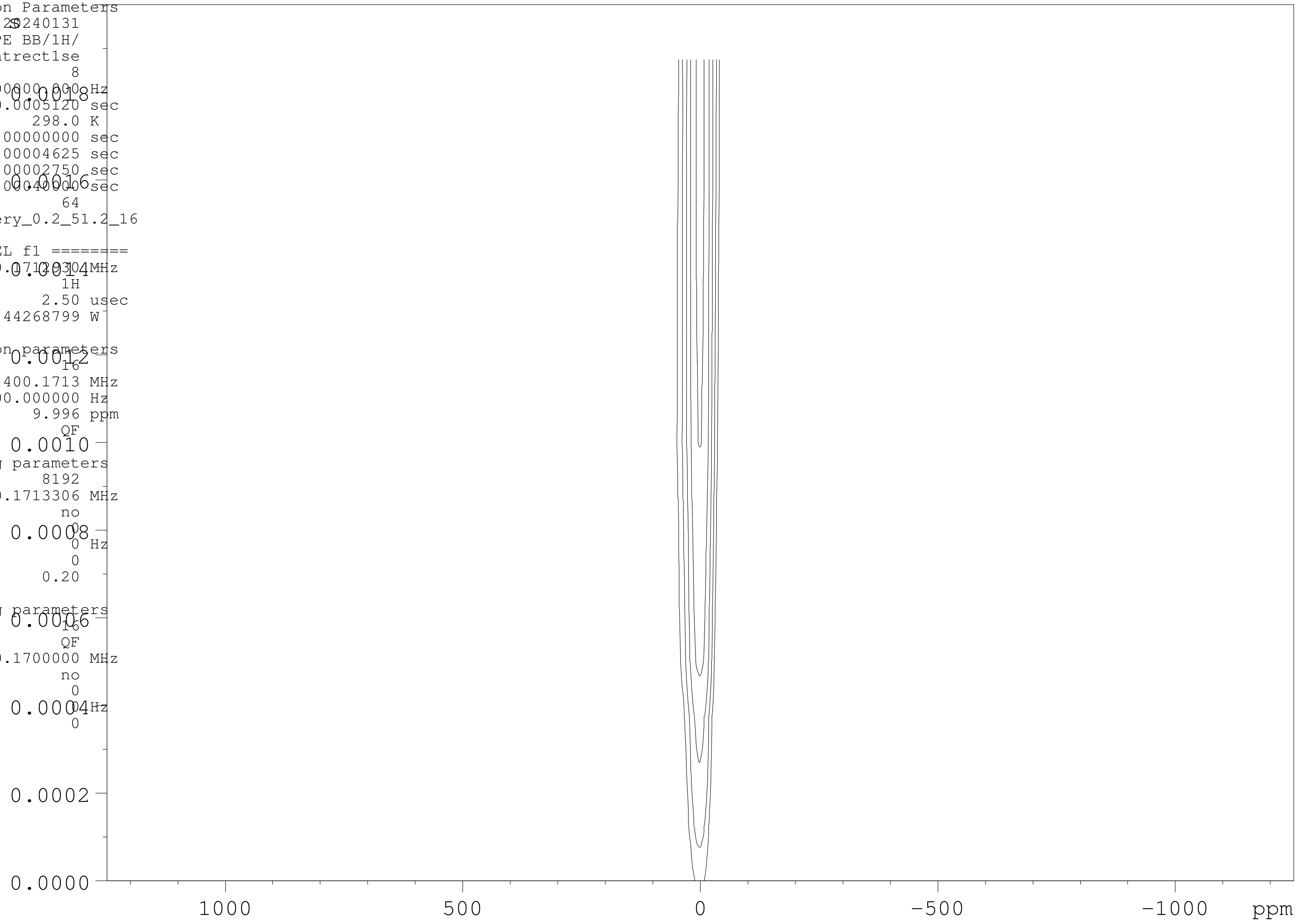

Current Data Parameters
NAME jse_20240130
EXPNO 29
PROCNO 1

2624-10340 SYT0043a S-CTA CdCl4 @ static / -100 to +120 C
-50 C

F2 - Acquisition Parameters
Date_ 20240131
PROBHD 5 mm PE BB/1H/
PULPROG t1rho_solidecho
NS 8
SWH 1000000.000 Hz
AQ 0.0005120 sec
TE 253.7 K
D1 11.19999981 sec
D6 0.00005000 sec
D7 0.0000[illegible] sec

======== CHANNEL f1 ========
SFO1 400.1712930 MHz
NUC1 1H
P1 2.50 usec
PLW1 378.44268799 W
PLW2 94.62400055 W
VPLIST 100u_52000u_16

F1 - Acquisition parameters
TD 8
SFO1 400.1713 MHz
FIDRES 1000.000000 Hz
SW 9.996 ppm
FnMODE QF

F2 - Processing parameters
SI 8192
SF 400.1713306 MHz
WDW no
SSB 0
LB 0 Hz
GB 0
PC 0.20

F1 - Processing parameters
SI 16
MC2 QF
SF 400.1700000 MHz
WDW no
SSB 0
LB 0 Hz
GB 0

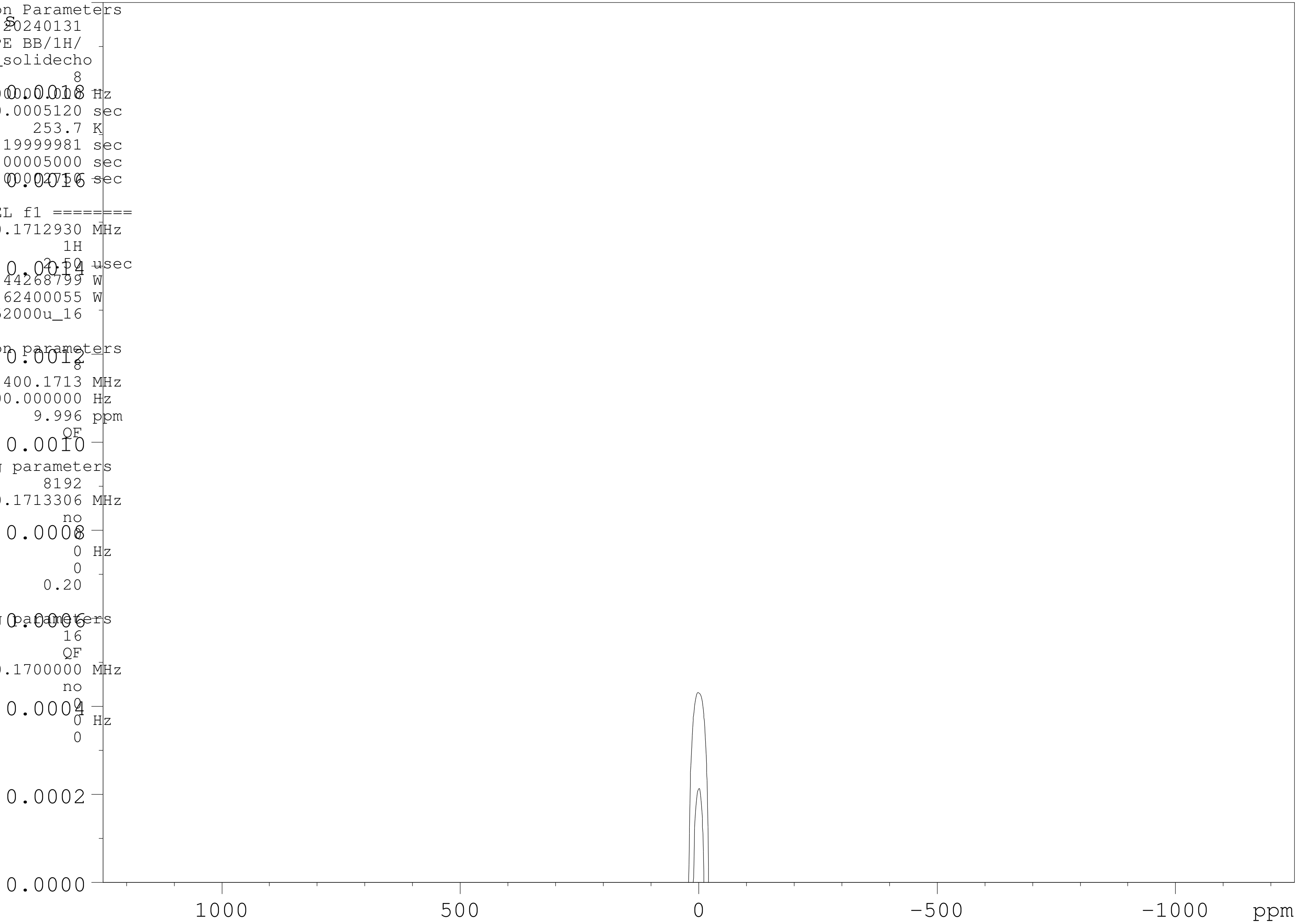

Current Data Parameters
NAME jse_20240130
EXPNO 31
PROCNO 1

F2 - Acquisition Parameters
Date_ 20240131
PROBHD 5 mm PE BB/1H/
PULPROG solidecho
NS 8
SWH 1000000.000 Hz
AQ 0.0005120 sec
TE 213.4 K
D1 7.19999981 sec
D6 0.00005000 sec
D7 0.00002750 sec

======== CHANNEL f1 ========
SFO1 400.1712930 MHz
NUC1 1H
P1 2.50 usec
PLW1 378.44268799 W

F2 - Processing parameters
SI 8192
SF 400.1713306 MHz
WDW EM
SSB 0
LB 10.00 Hz
GB 0
PC 0.20

2624-10340 SYT0043a S-CTA CdCl4 @ static / -100 to +120 C
-40 C

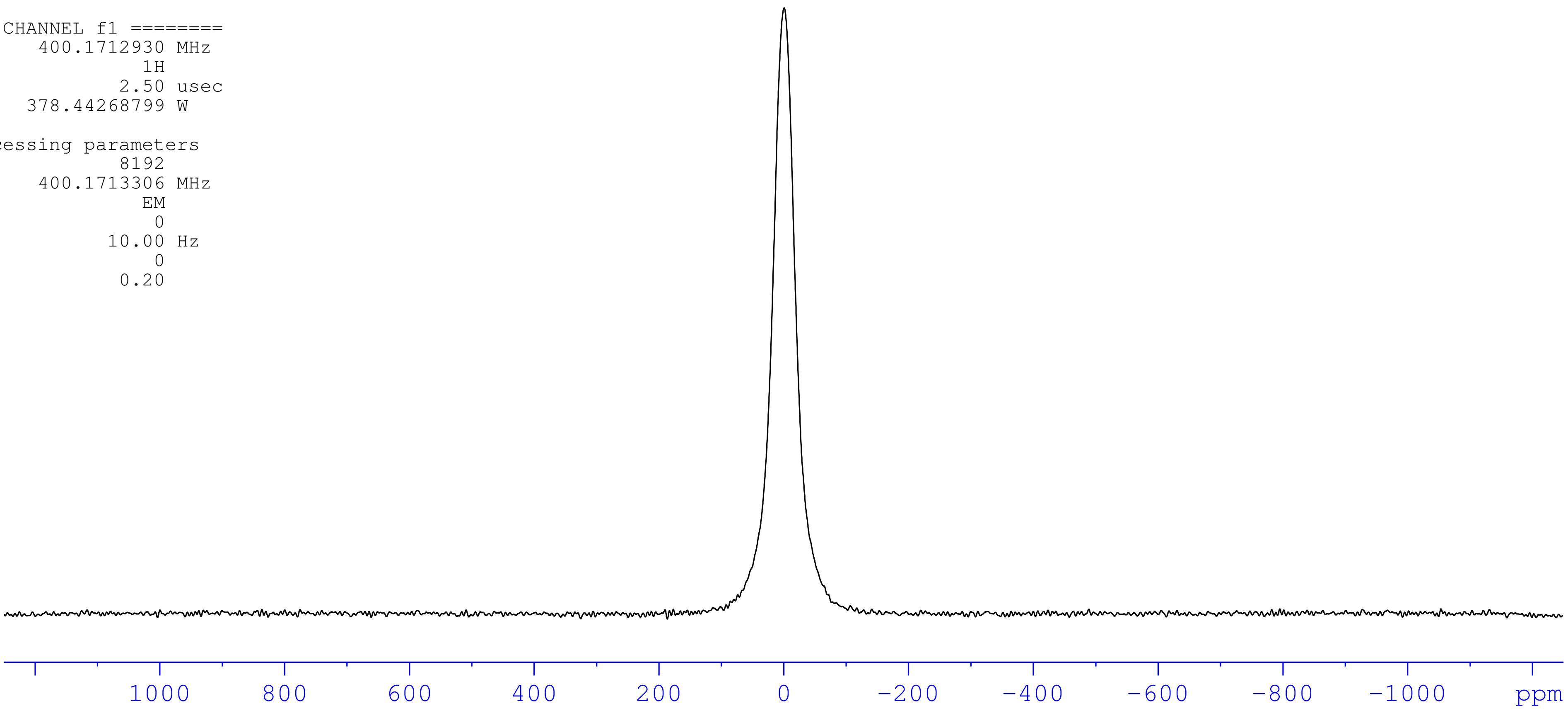

Current Data Parameters
NAME jse_20240130
EXPNO 32
PROCNO 1

F2 - Acquisition Parameters
Date_ 20240131
PROBHD 5 mm PE BB/1H/
PULPROG zg
NS 4
SWH 1000000.000 Hz
AQ 0.0005120 sec
TE 294.0 K
D1 7.19999981 sec
TD0 1

======== CHANNEL f1 ========
SFO1 400.1712930 MHz
NUC1 1H
P1 2.50 usec
PLW1 378.44268799 W

F2 - Processing parameters
SI 8192
SF 400.1713306 MHz
WDW EM
SSB 0
LB 10.00 Hz
GB 0
PC 0.20

2624-10340 SYT0043a S-CTA CdCl4 @ static / -100 to +120 C
-40 C

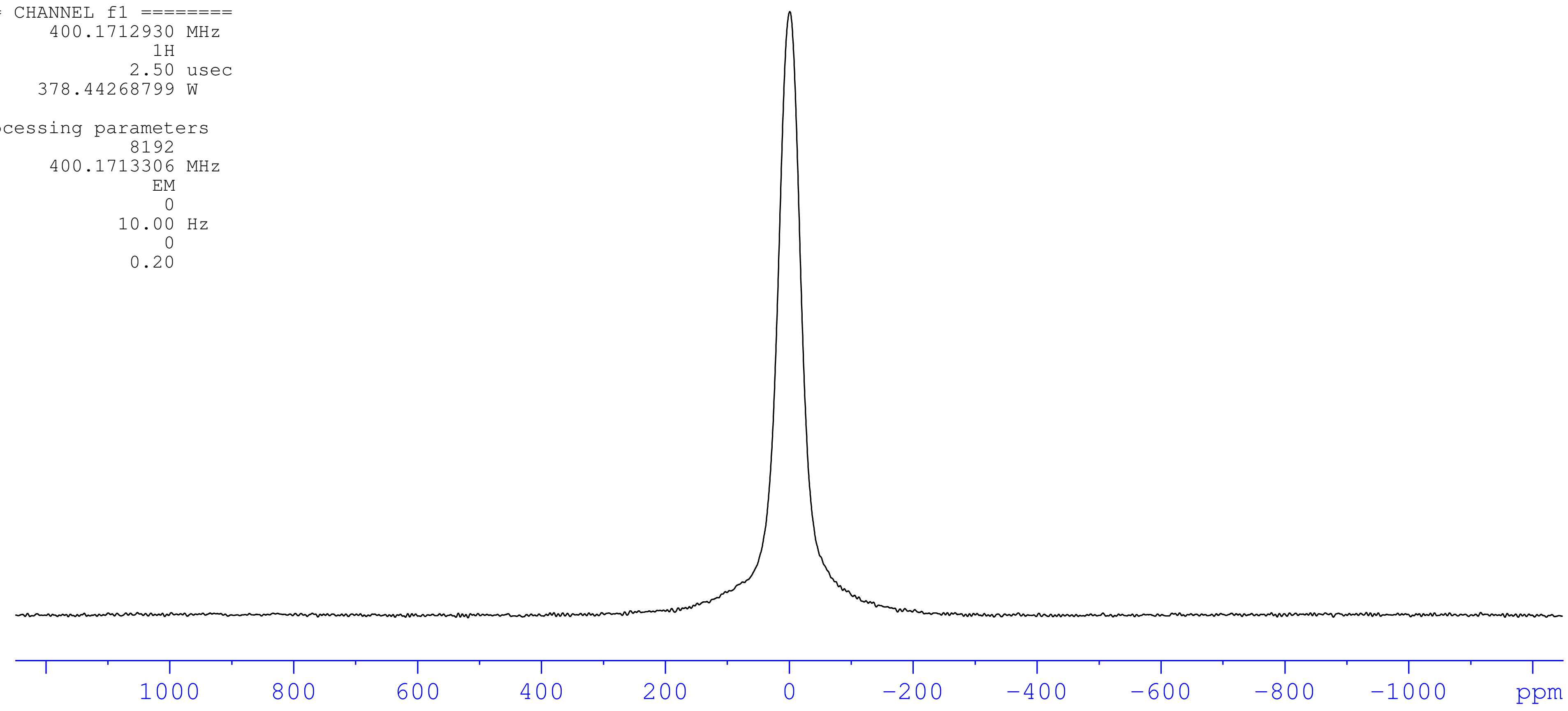

2624-10340 SYT0043a S-CTA CdCl4 @ static / -100 to +120 C
-40 C

Current Data Parameters
NAME jse_20240130
EXPNO 33
PROCNO 1

F2 - Acquisition Parameters
Date_ 20240131
PROBHD 5 mm PE BB/1H/
PULPROG satrect1se
NS 8
SWH 1000000.000 Hz
AQ 0.0005120 sec
TE 298.0 K
D1 1.00000000 sec
D6 0.00004625 sec
D7 0.00002750 sec
D20 0.00040000 sec
L20 64
VDLIST Recovery_0.1_12.8_16

======== CHANNEL f1 ========
SFO1 400.1712930 MHz
NUC1 1H
P1 2.50 usec
PLW1 378.44268799 W

F1 - Acquisition parameters
TD 16
SFO1 400.1713 MHz
FIDRES 500.000000 Hz
SW 9.996 ppm
FnMODE QF

F2 - Processing parameters
SI 8192
SF 400.1713306 MHz
WDW no
SSB 0
LB 0 Hz
GB 0
PC 0.20

F1 - Processing parameters
SI 16
MC2 QF
SF 400.1700000 MHz
WDW no
SSB 0
LB 0 Hz
GB 0

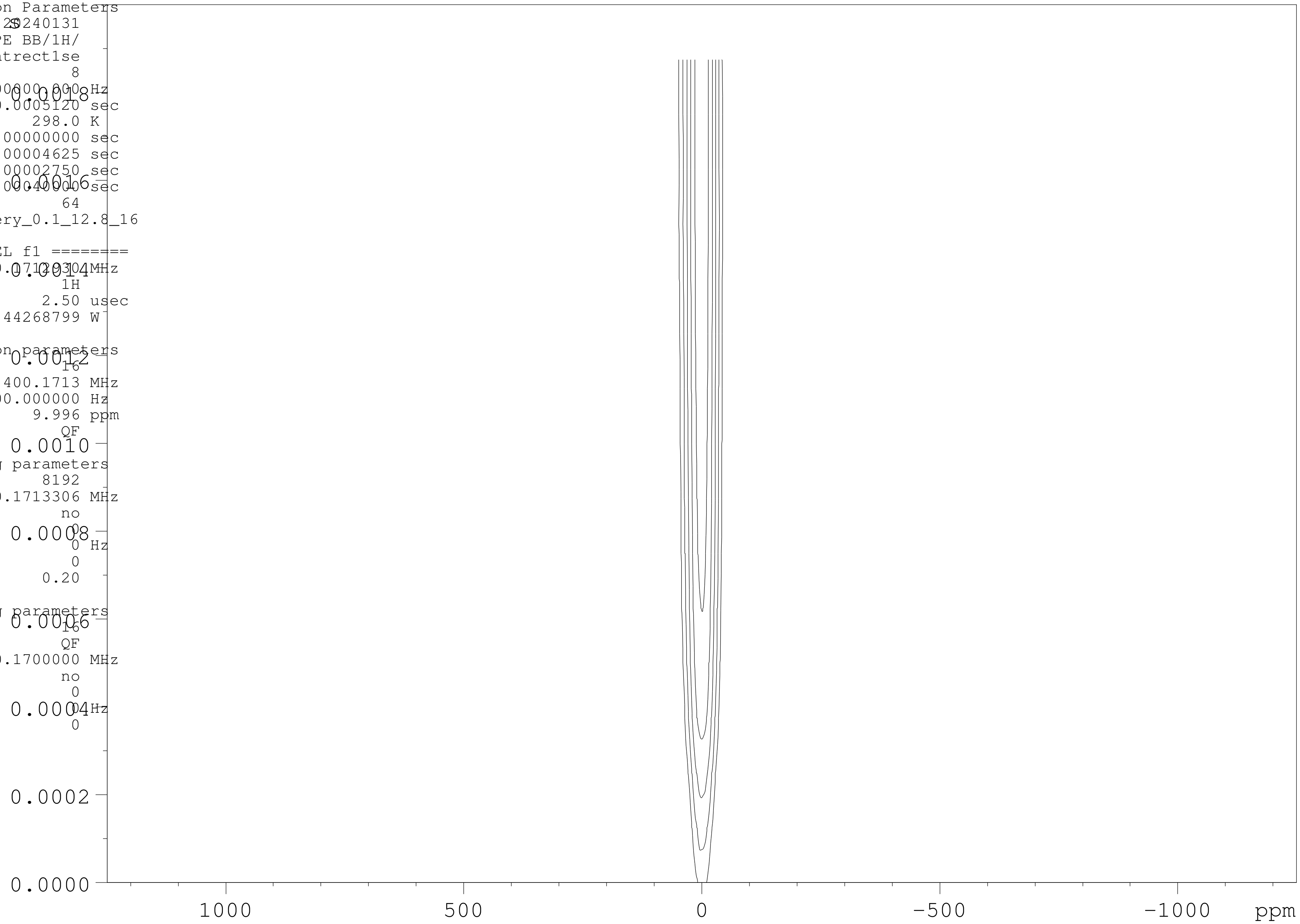

Current Data Parameters
NAME jse_20240130
EXPNO 34
PROCNO 1

F2 - Acquisition Parameters
Date_ 20240131
PROBHD 5 mm PE BB/1H/
PULPROG t1rho_solidecho
NS 8
SWH 1000000.000 Hz
AQ 0.0005120 sec
TE 260.4 K
D1 7.19999981 sec
D6 0.00005000 sec
D7 0.00002750 sec

======== CHANNEL f1 ========
SFO1 400.1712930 MHz
NUC1 1H
P1 2.50 usec
PLW1 378.44268799 W
PLW2 94.62400055 W
VPLIST 100u_52000u_16

F1 - Acquisition parameters
TD 8
SFO1 400.1713 MHz
FIDRES 1000.000000 Hz
SW 9.996 ppm
FnMODE QF

F2 - Processing parameters
SI 8192
SF 400.1713306 MHz
WDW no
SSB 0
LB 0 Hz
GB 0
PC 0.20

F1 - Processing parameters
SI 16
MC2 QF
SF 400.1700000 MHz
WDW no
SSB 0
LB 0 Hz
GB 0

2624-10340 SYT0043a S-CTA CdCl4 @ static / -100 to +120 C
-40 C

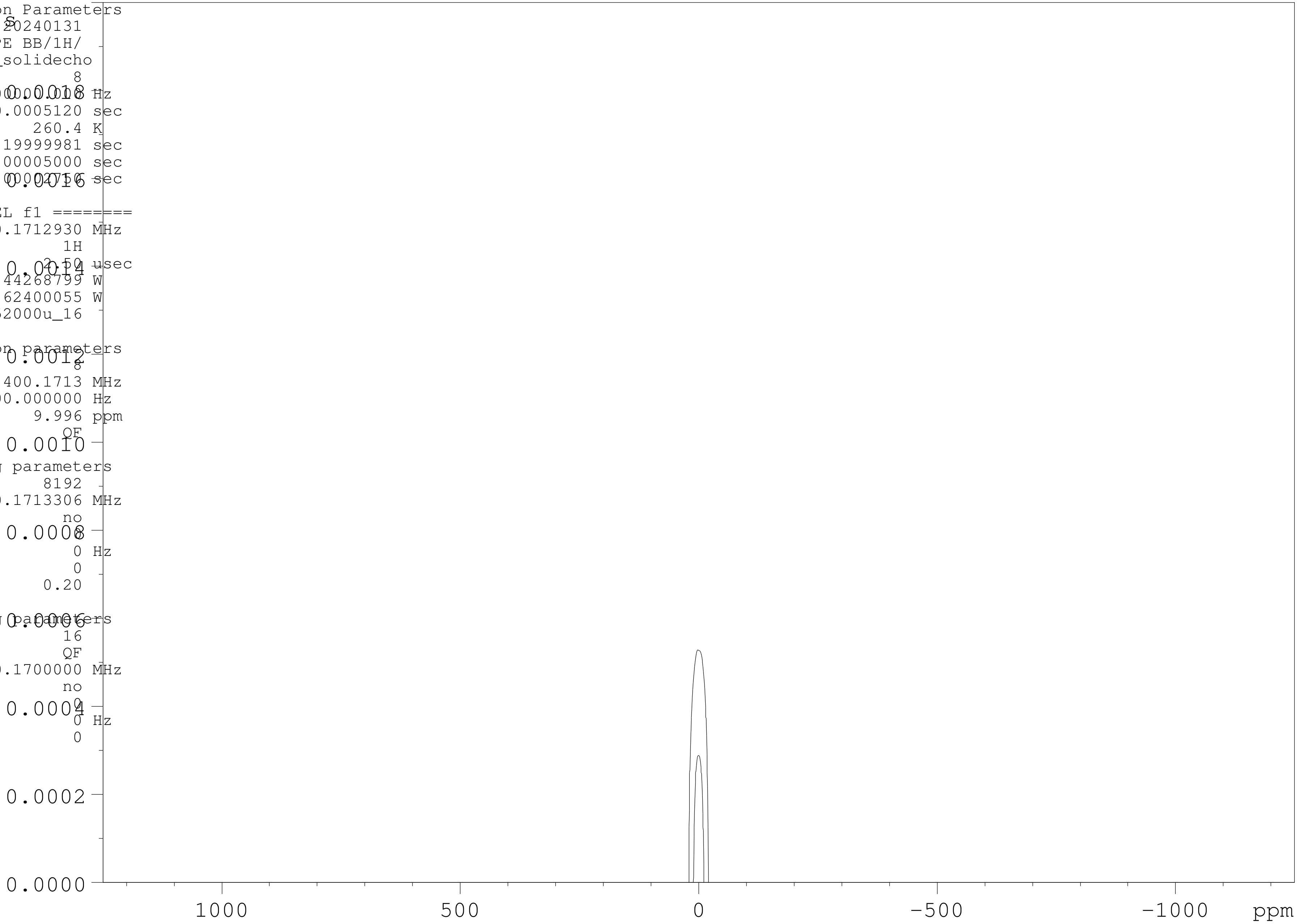

Current Data Parameters
NAME jse_20240130
EXPNO 36
PROCNO 1

F2 - Acquisition Parameters
Date_ 20240131
PROBHD 5 mm PE BB/1H/
PULPROG solidecho
NS 8
SWH 1000000.000 Hz
AQ 0.0005120 sec
TE 213.4 K
D1 7.19999981 sec
D6 0.00005000 sec
D7 0.00002750 sec

======== CHANNEL f1 ========
SFO1 400.1712930 MHz
NUC1 1H
P1 2.50 usec
PLW1 378.44268799 W

F2 - Processing parameters
SI 8192
SF 400.1713306 MHz
WDW EM
SSB 0
LB 10.00 Hz
GB 0
PC 0.20

2624-10340 SYT0043a S-CTA CdCl4 @ static / -100 to +120 C
-30 C

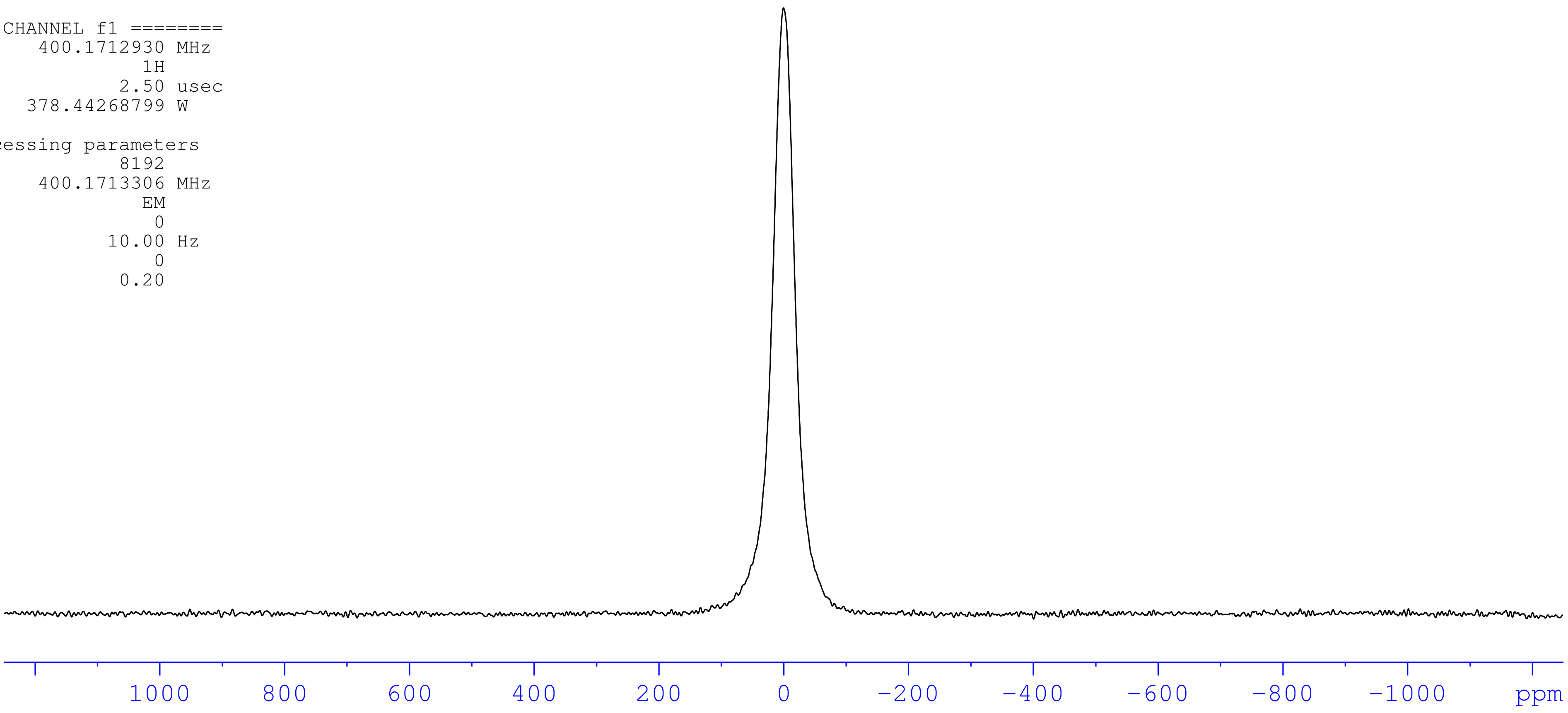

2624-10340 SYT0043a S-CTA CdCl4 @ static / -100 to +120 C
-30 C

```
Current Data Parameters
NAME       jse_20240130
EXPNO                37
PROCNO                1

F2 - Acquisition Parameters
Date_          20240131
PROBHD   5 mm PE BB/1H/
PULPROG              zg
NS                    4
SWH         1000000.000 Hz
AQ            0.0005120 sec
TE                294.0 K
D1           7.19999981 sec
TD0                   1

======== CHANNEL f1 ========
SFO1        400.1712930 MHz
NUC1                 1H
P1                 2.50 usec
PLW1       378.44268799 W

F2 - Processing parameters
SI                 8192
SF          400.1713306 MHz
WDW                  EM
SSB                   0
LB                10.00 Hz
GB                    0
PC                 0.20
```

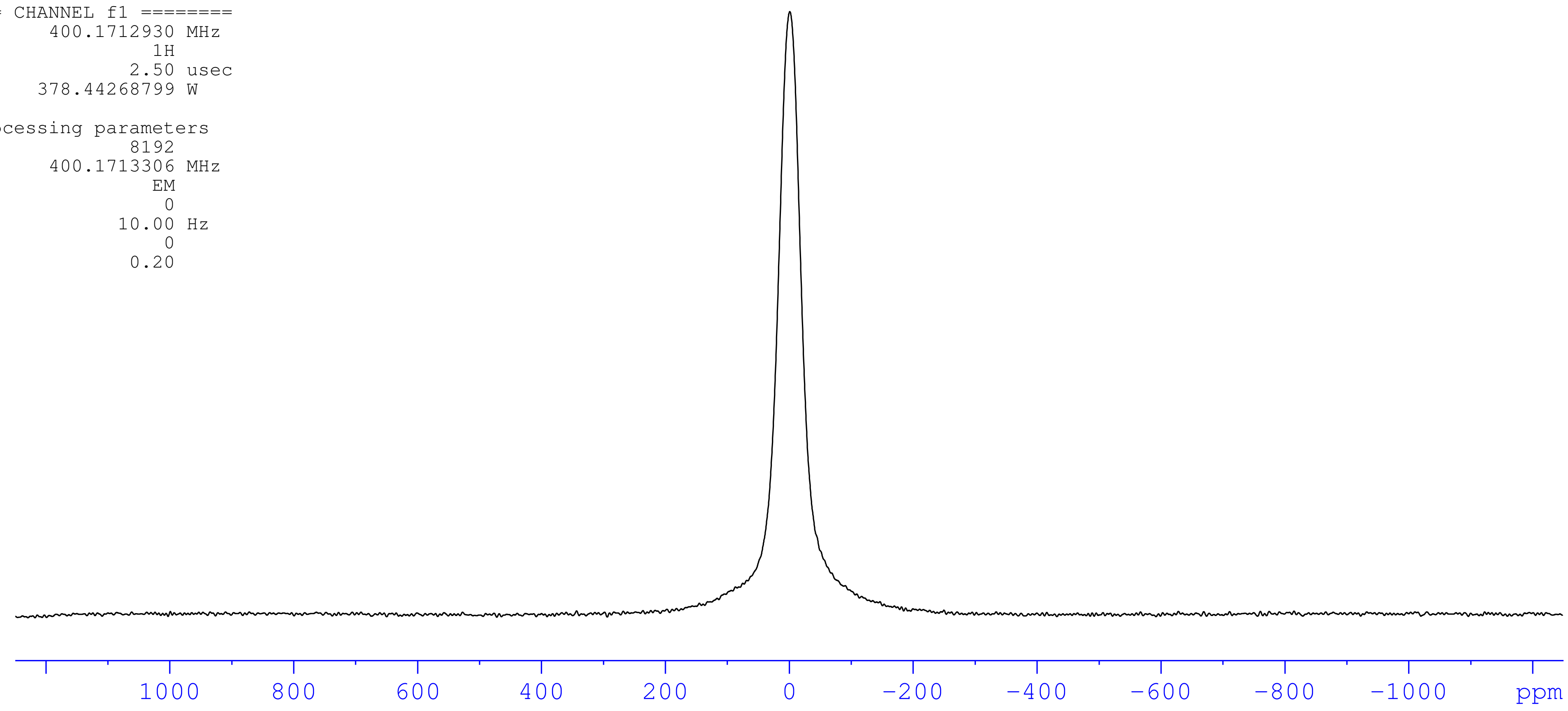

Current Data Parameters
NAME jse_20240130
EXPNO 38
PROCNO 1

F2 - Acquisition Parameters
Date_ 20240131
PROBHD 5 mm PE BB/1H/
PULPROG satrect1se
NS 8
SWH 1000000.000 Hz
AQ 0.0005120 sec
TE 298.0 K
D1 1.00000000 sec
D6 0.00004625 sec
D7 0.00002750 sec
D20 0.00040000 sec
L20 64
VDLIST Recovery_0.1_12.8_16

======== CHANNEL f1 ========
SFO1 400.1712930 MHz
NUC1 1H
P1 2.50 usec
PLW1 378.44268799 W

F1 - Acquisition parameters
TD 16
SFO1 400.1713 MHz
FIDRES 500.000000 Hz
SW 9.996 ppm
FnMODE QF

F2 - Processing parameters
SI 8192
SF 400.1713306 MHz
WDW no
SSB 0
LB 0 Hz
GB 0
PC 0.20

F1 - Processing parameters
SI 16
MC2 QF
SF 400.1700000 MHz
WDW no
SSB 0
LB 0 Hz
GB 0

2624-10340 SYT0043a S-CTA CdCl4 @ static / -100 to +120 C
-30 C

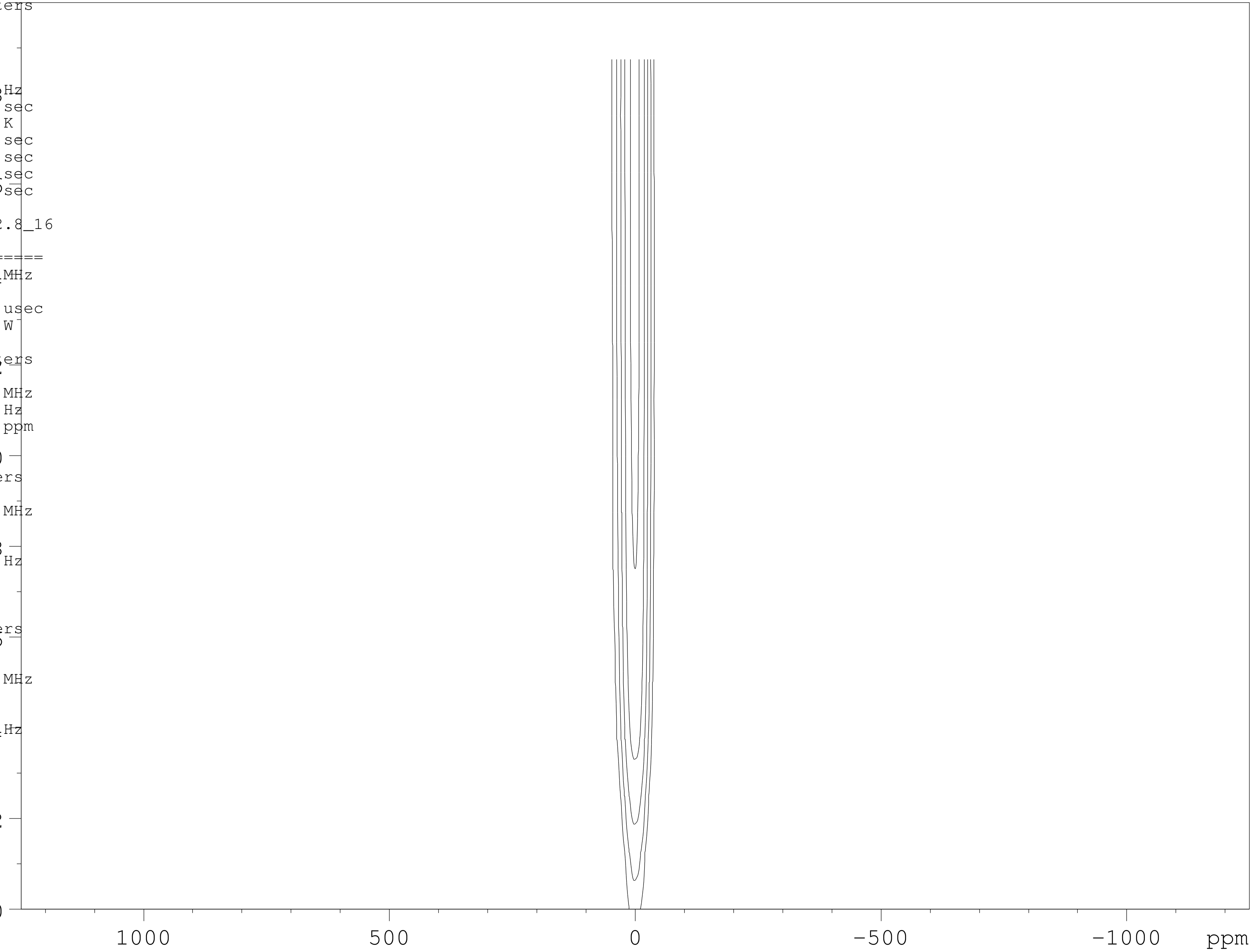

2624-10340 SYT0043a S-CTA CdCl4 @ static / -100 to +120 C
-30 C

Current Data Parameters
NAME jse_20240130
EXPNO 39
PROCNO 1

F2 - Acquisition Parameters
Date_ 20240131
PROBHD 5 mm PE BB/1H/
PULPROG t1rho_solidecho
NS 8
SWH 1000000.000 Hz
AQ 0.0005120 sec
TE 267.1 K
D1 7.19999981 sec
D6 0.00005000 sec
D7 0.00002756 sec

======== CHANNEL f1 ========
SFO1 400.1712930 MHz
NUC1 1H
P1 2.50 usec
PLW1 378.44268799 W
PLW2 94.62400055 W
VPLIST 100u_52000u_16

F1 - Acquisition parameters
TD 8
SFO1 400.1713 MHz
FIDRES 1000.000000 Hz
SW 9.996 ppm
FnMODE QF

F2 - Processing parameters
SI 8192
SF 400.1713306 MHz
WDW no
SSB 0
LB 0 Hz
GB 0
PC 0.20

F1 - Processing parameters
SI 16
MC2 QF
SF 400.1700000 MHz
WDW no
SSB 0
LB 0 Hz
GB 0

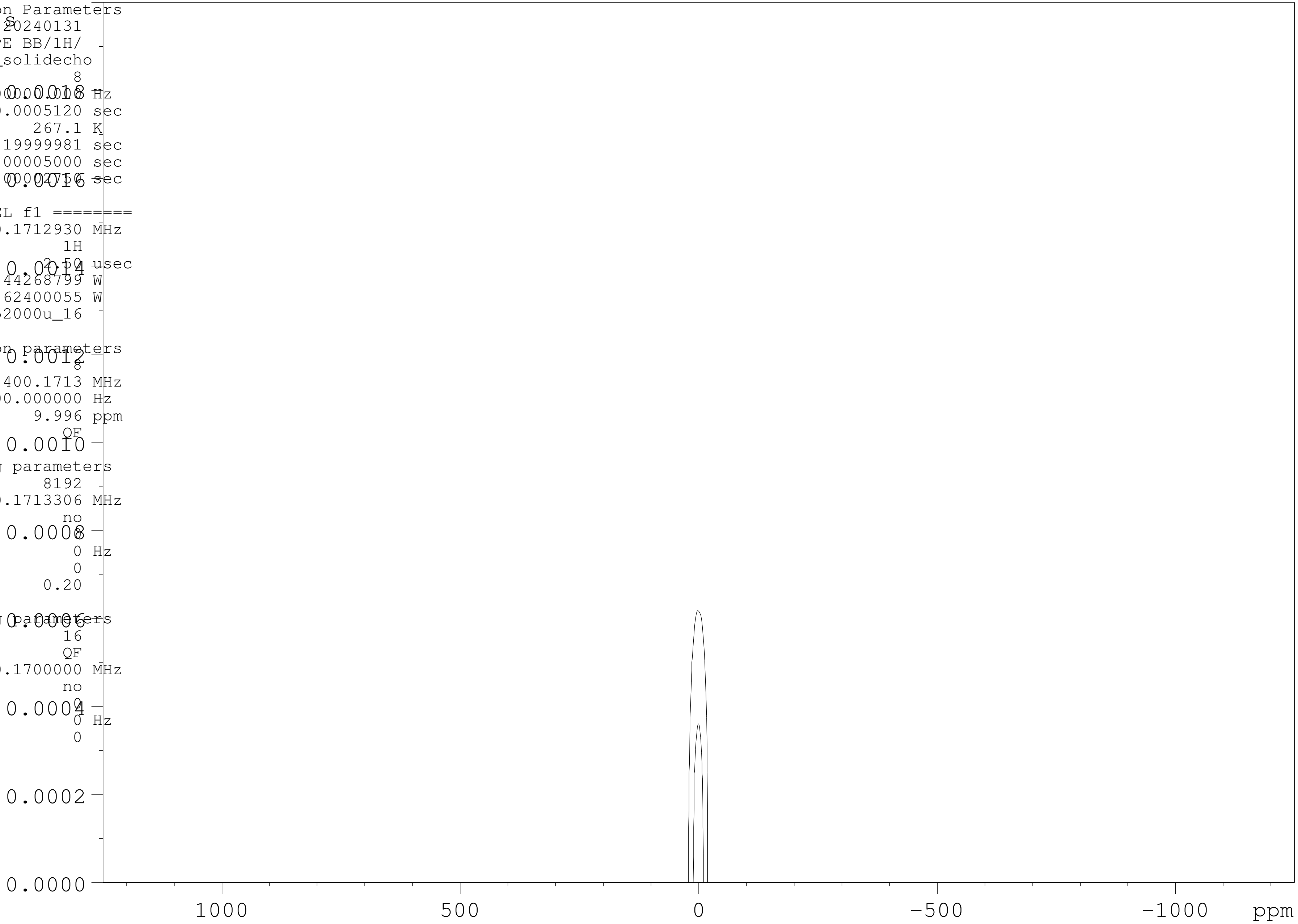

Current Data Parameters
NAME jse_20240130
EXPNO 41
PROCNO 1

F2 - Acquisition Parameters
Date_ 20240131
PROBHD 5 mm PE BB/1H/
PULPROG solidecho
NS 8
SWH 1000000.000 Hz
AQ 0.0005120 sec
TE 213.4 K
D1 7.19999981 sec
D6 0.00005000 sec
D7 0.00002750 sec

======== CHANNEL f1 ========
SFO1 400.1712930 MHz
NUC1 1H
P1 2.50 usec
PLW1 378.44268799 W

F2 - Processing parameters
SI 8192
SF 400.1713306 MHz
WDW EM
SSB 0
LB 10.00 Hz
GB 0
PC 0.20

2624-10340 SYT0043a S-CTA CdCl4 @ static / -100 to +120 C
-20 C

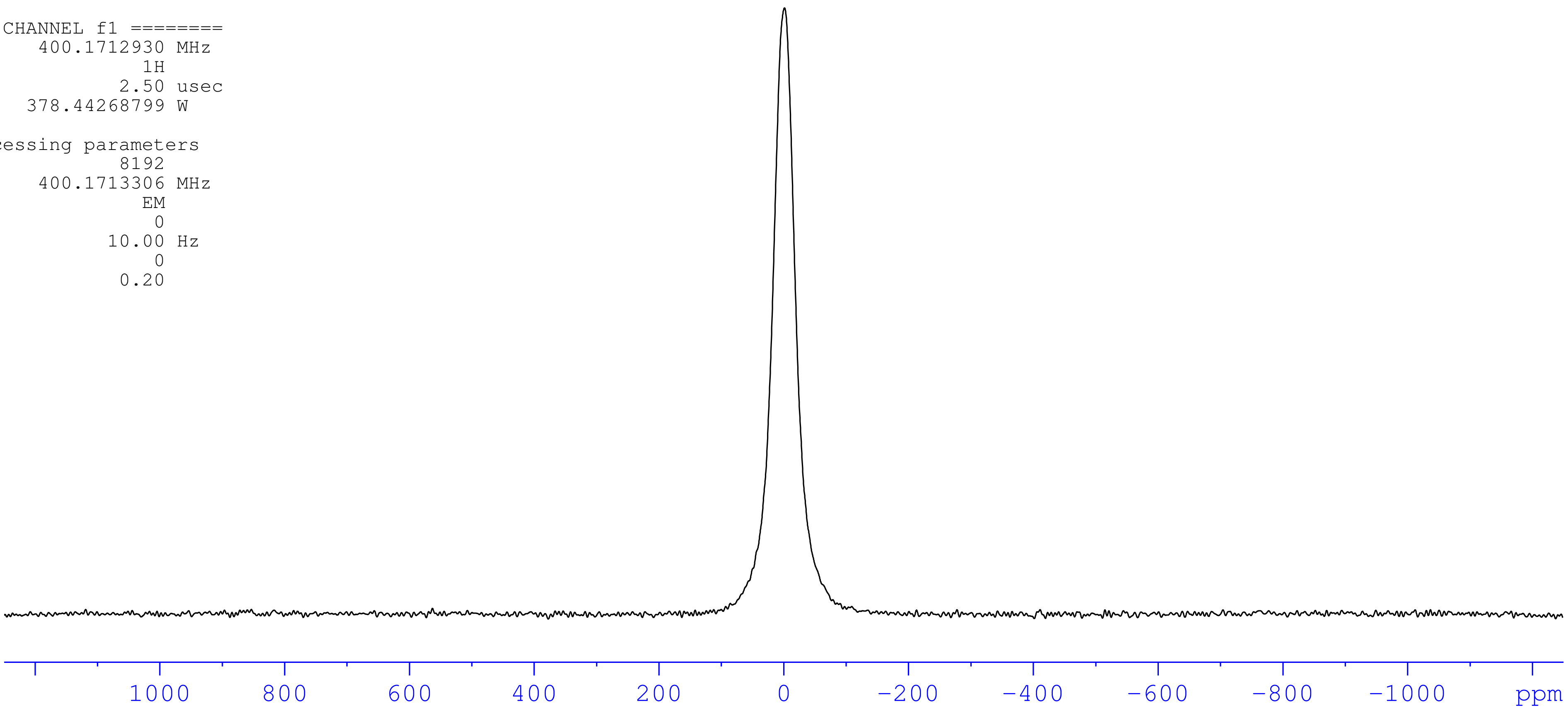

Current Data Parameters
NAME jse_20240130
EXPNO 42
PROCNO 1

F2 - Acquisition Parameters
Date_ 20240131
PROBHD 5 mm PE BB/1H/
PULPROG zg
NS 4
SWH 1000000.000 Hz
AQ 0.0005120 sec
TE 294.0 K
D1 7.19999981 sec
TD0 1

======== CHANNEL f1 ========
SFO1 400.1712930 MHz
NUC1 1H
P1 2.50 usec
PLW1 378.44268799 W

F2 - Processing parameters
SI 8192
SF 400.1713306 MHz
WDW EM
SSB 0
LB 10.00 Hz
GB 0
PC 0.20

2624-10340 SYT0043a S-CTA CdCl4 @ static / -100 to +120 C
-20 C

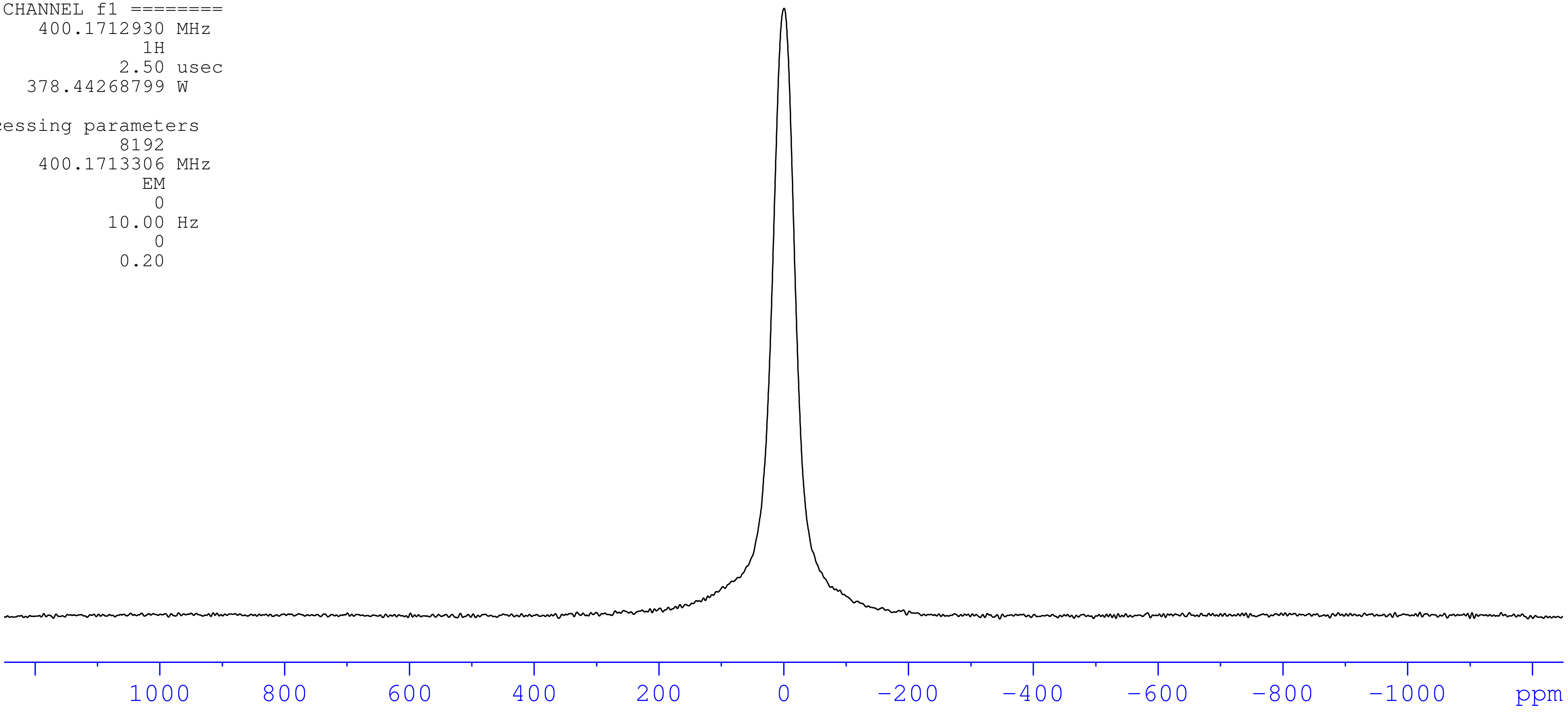

Current Data Parameters
NAME   jse_20240130
EXPNO   43
PROCNO   1

2624-10340 SYT0043a S-CTA CdCl4 @ static / -100 to +120 C
-20 C

F2 - Acquisition Parameters
Date_   20240131
PROBHD   5 mm PE BB/1H/
PULPROG   satrect1se
NS   8
SWH   1000000.000 Hz
AQ   0.0005120 sec
TE   298.0 K
D1   1.00000000 sec
D6   0.00004625 sec
D7   0.00002750 sec
D20   0.00040000 sec
L20   64
VDLIST   Recovery_0.1_12.8_16

======== CHANNEL f1 ========
SFO1   400.1712930 MHz
NUC1   1H
P1   2.50 usec
PLW1   378.44268799 W

F1 - Acquisition parameters
TD   16
SFO1   400.1713 MHz
FIDRES   500.000000 Hz
SW   9.996 ppm
FnMODE   QF

F2 - Processing parameters
SI   8192
SF   400.1713306 MHz
WDW   no
SSB   0
LB   0 Hz
GB   0
PC   0.20

F1 - Processing parameters
SI   16
MC2   QF
SF   400.1700000 MHz
WDW   no
SSB   0
LB   0 Hz
GB   0

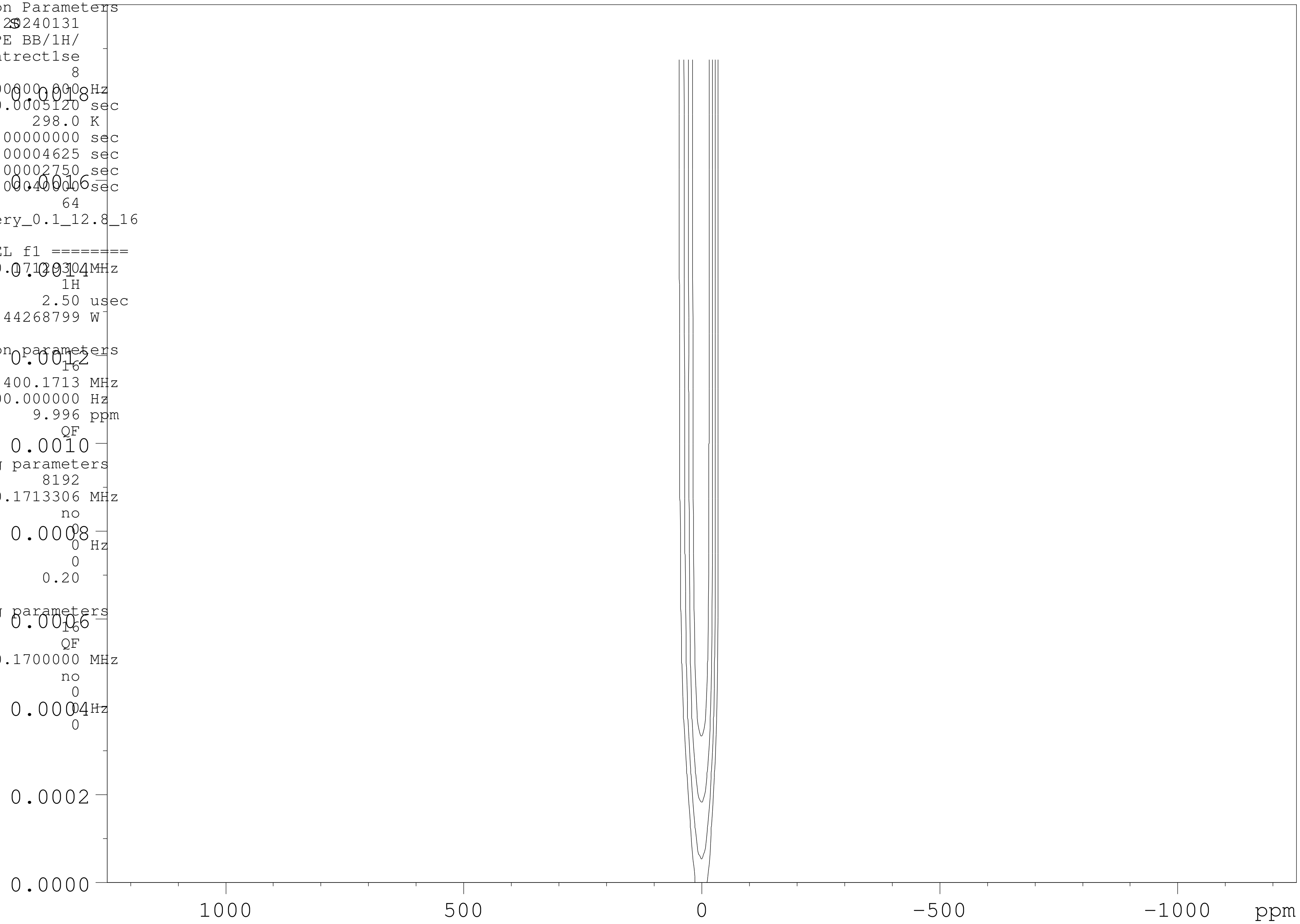

2624-10340 SYT0043a S-CTA CdCl4 @ static / -100 to +120 C
-20 C

Current Data Parameters
NAME jse_20240130
EXPNO 44
PROCNO 1

F2 - Acquisition Parameters
Date_ 20240131
PROBHD 5 mm PE BB/1H/
PULPROG t1rho_solidecho
NS 8
SWH 1000000.000 Hz
AQ 0.0005120 sec
TE 273.8 K
D1 7.19999981 sec
D6 0.00005000 sec
D7 0.00002756 sec

======== CHANNEL f1 ========
SFO1 400.1712930 MHz
NUC1 1H
P1 2.50 usec
PLW1 378.44268799 W
PLW2 94.62400055 W
VPLIST 100u_52000u_16

F1 - Acquisition parameters
TD 8
SFO1 400.1713 MHz
FIDRES 1000.000000 Hz
SW 9.996 ppm
FnMODE QF

F2 - Processing parameters
SI 8192
SF 400.1713306 MHz
WDW no
SSB 0
LB 0 Hz
GB 0
PC 0.20

F1 - Processing parameters
SI 16
MC2 QF
SF 400.1700000 MHz
WDW no
SSB 0
LB 0 Hz
GB 0

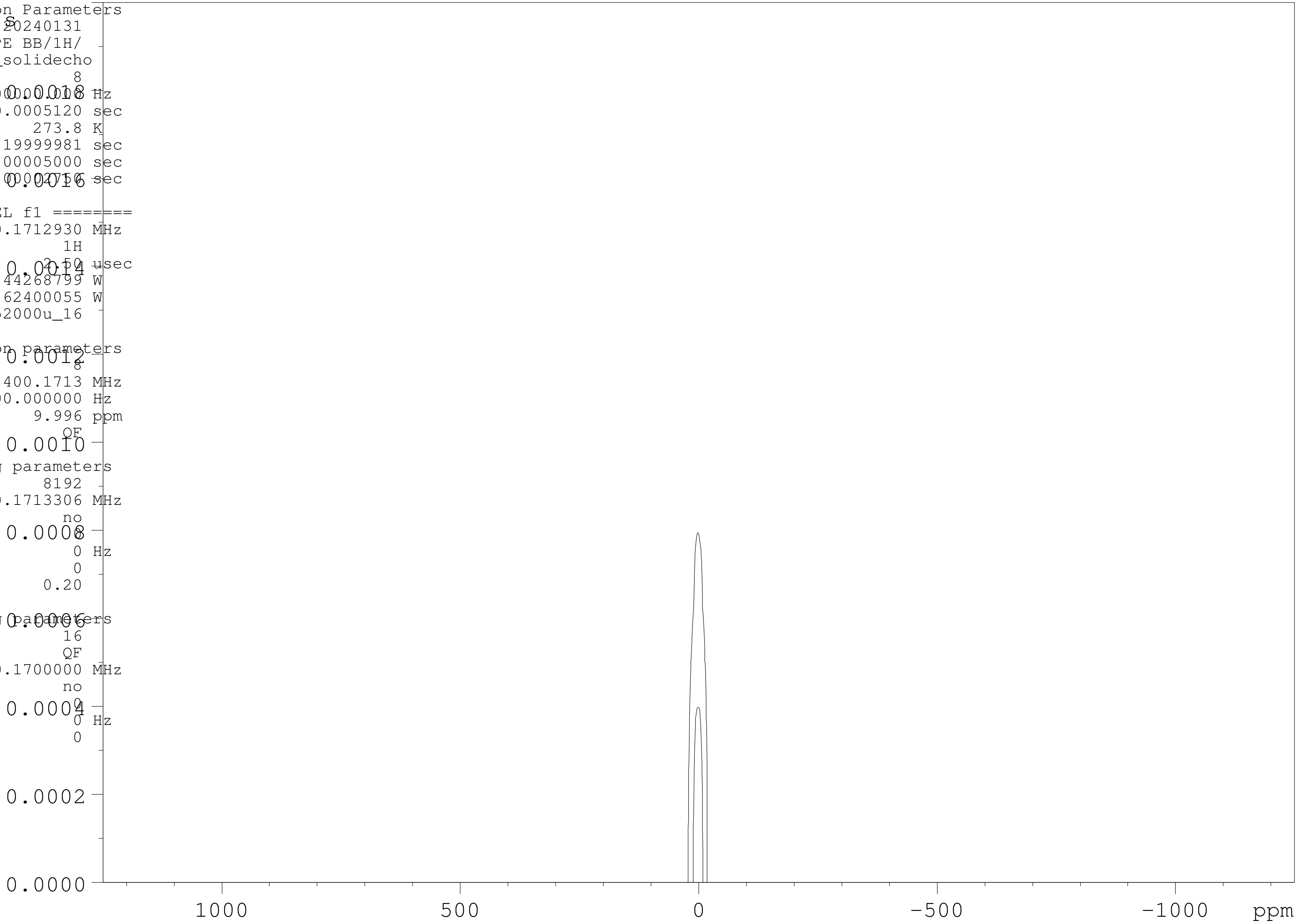

Current Data Parameters
NAME jse_20240130
EXPNO 46
PROCNO 1

F2 - Acquisition Parameters
Date_ 20240131
PROBHD 5 mm PE BB/1H/
PULPROG solidecho
NS 8
SWH 1000000.000 Hz
AQ 0.0005120 sec
TE 213.4 K
D1 7.19999981 sec
D6 0.00005000 sec
D7 0.00002750 sec

======== CHANNEL f1 ========
SFO1 400.1712930 MHz
NUC1 1H
P1 2.50 usec
PLW1 378.44268799 W

F2 - Processing parameters
SI 8192
SF 400.1713306 MHz
WDW EM
SSB 0
LB 10.00 Hz
GB 0
PC 0.20

2624-10340 SYT0043a S-CTA CdCl4 @ static / -100 to +120 C
-10 C

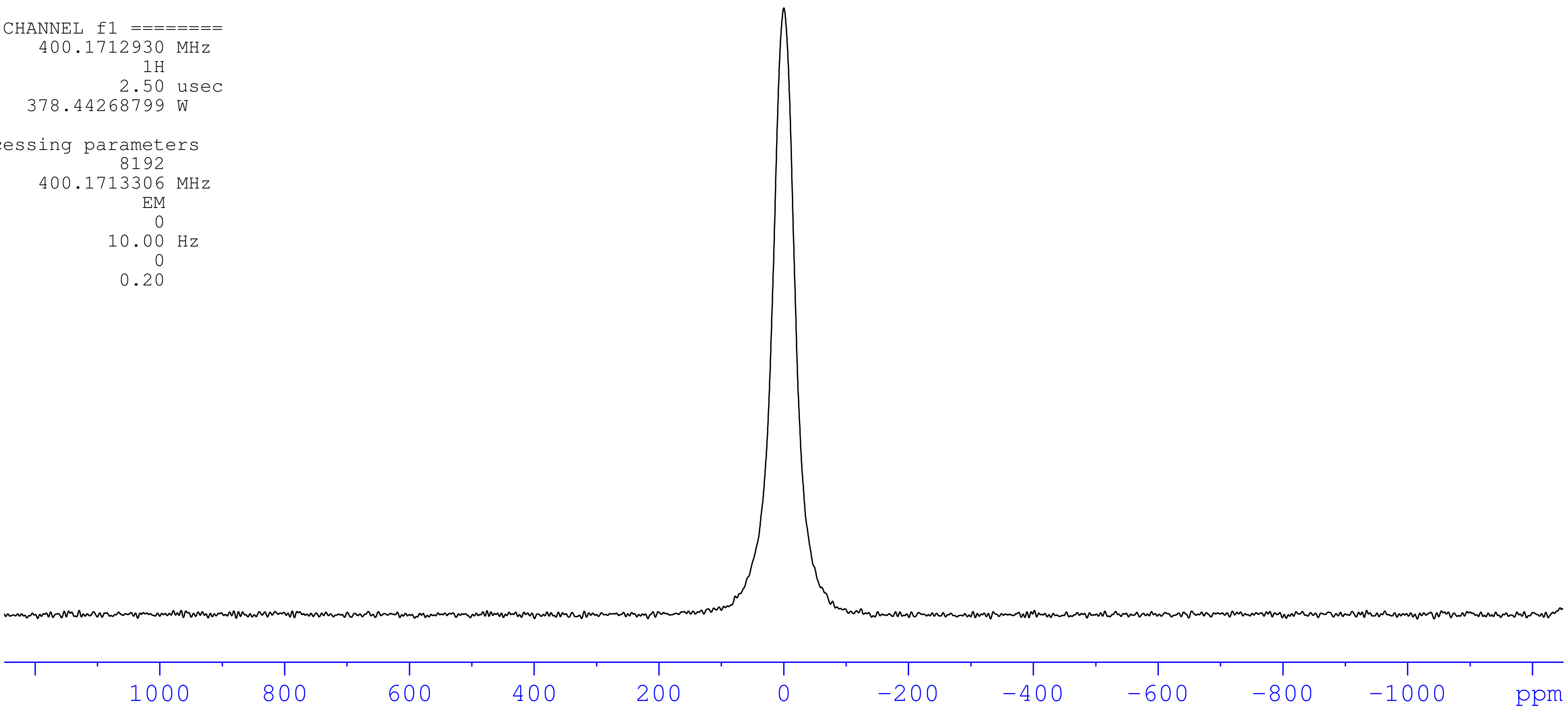

```
Current Data Parameters
NAME        jse_20240130
EXPNO                 47
PROCNO                 1

F2 - Acquisition Parameters
Date_           20240131
PROBHD     5 mm PE BB/1H/
PULPROG               zg
NS                     4
SWH          1000000.000 Hz
AQ             0.0005120 sec
TE                 294.0 K
D1            7.19999981 sec
TD0                    1

======== CHANNEL f1 ========
SFO1         400.1712930 MHz
NUC1                  1H
P1                  2.50 usec
PLW1        378.44268799 W

F2 - Processing parameters
SI                  8192
SF           400.1713306 MHz
WDW                   EM
SSB                    0
LB                 10.00 Hz
GB                     0
PC                  0.20
```

2624-10340 SYT0043a S-CTA CdCl4 @ static / -100 to +120 C
-10 C

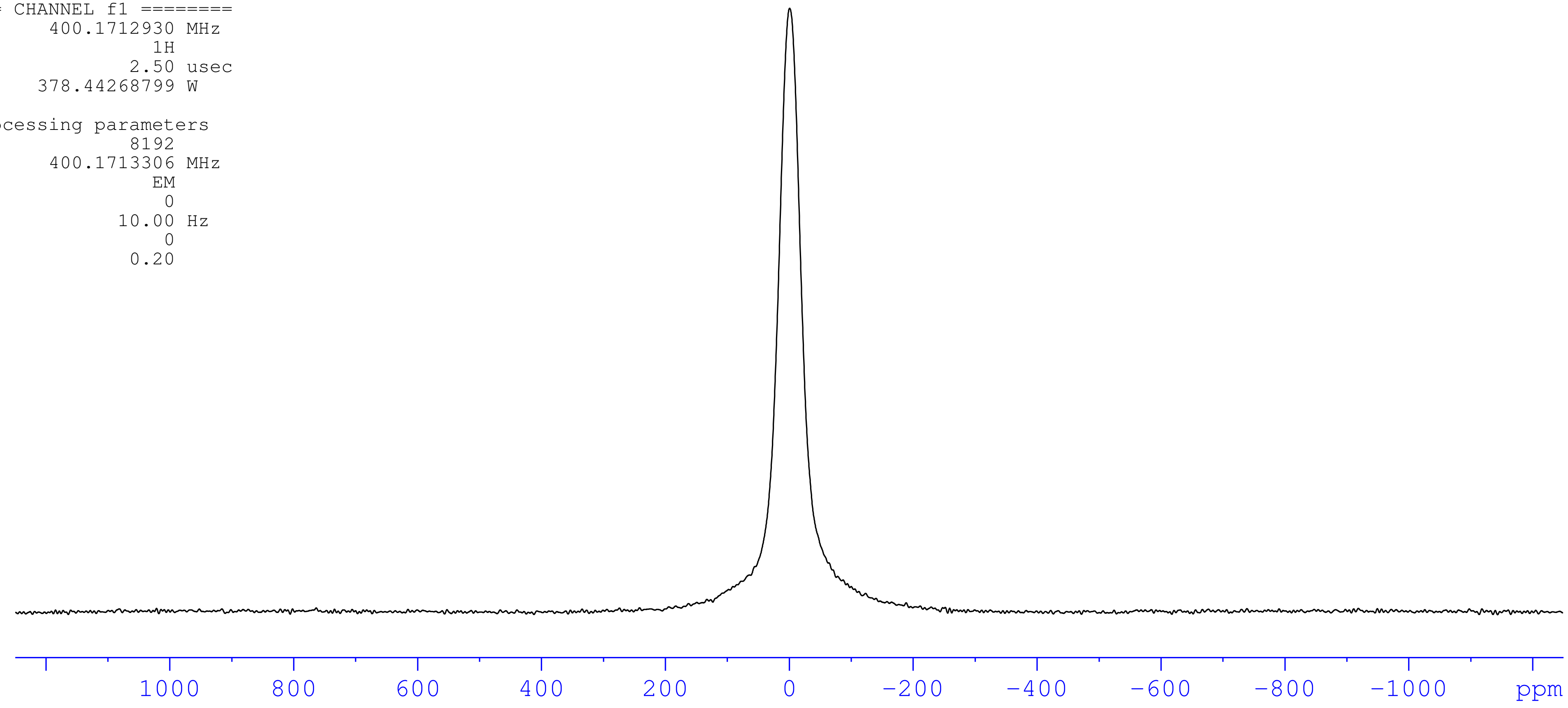

Current Data Parameters
NAME jse_20240130
EXPNO 48
PROCNO 1

2624-10340 SYT0043a S-CTA CdCl4 @ static / -100 to +120 C
-10 C

F2 - Acquisition Parameters
Date_ 20240131
PROBHD 5 mm PE BB/1H/
PULPROG satrect1se
NS 8
SWH 1000000.000 Hz
AQ 0.0005120 sec
TE 298.0 K
D1 1.00000000 sec
D6 0.00004625 sec
D7 0.00002750 sec
D20 0.00040000 sec
L20 64
VDLIST Recovery_0.1_12.8_16

======== CHANNEL f1 ========
SFO1 400.1712930 MHz
NUC1 1H
P1 2.50 usec
PLW1 378.44268799 W

F1 - Acquisition parameters
TD 16
SFO1 400.1713 MHz
FIDRES 500.000000 Hz
SW 9.996 ppm
FnMODE QF

F2 - Processing parameters
SI 8192
SF 400.1713306 MHz
WDW no
SSB 0
LB 0 Hz
GB 0
PC 0.20

F1 - Processing parameters
SI 16
MC2 QF
SF 400.1700000 MHz
WDW no
SSB 0
LB 0 Hz
GB 0

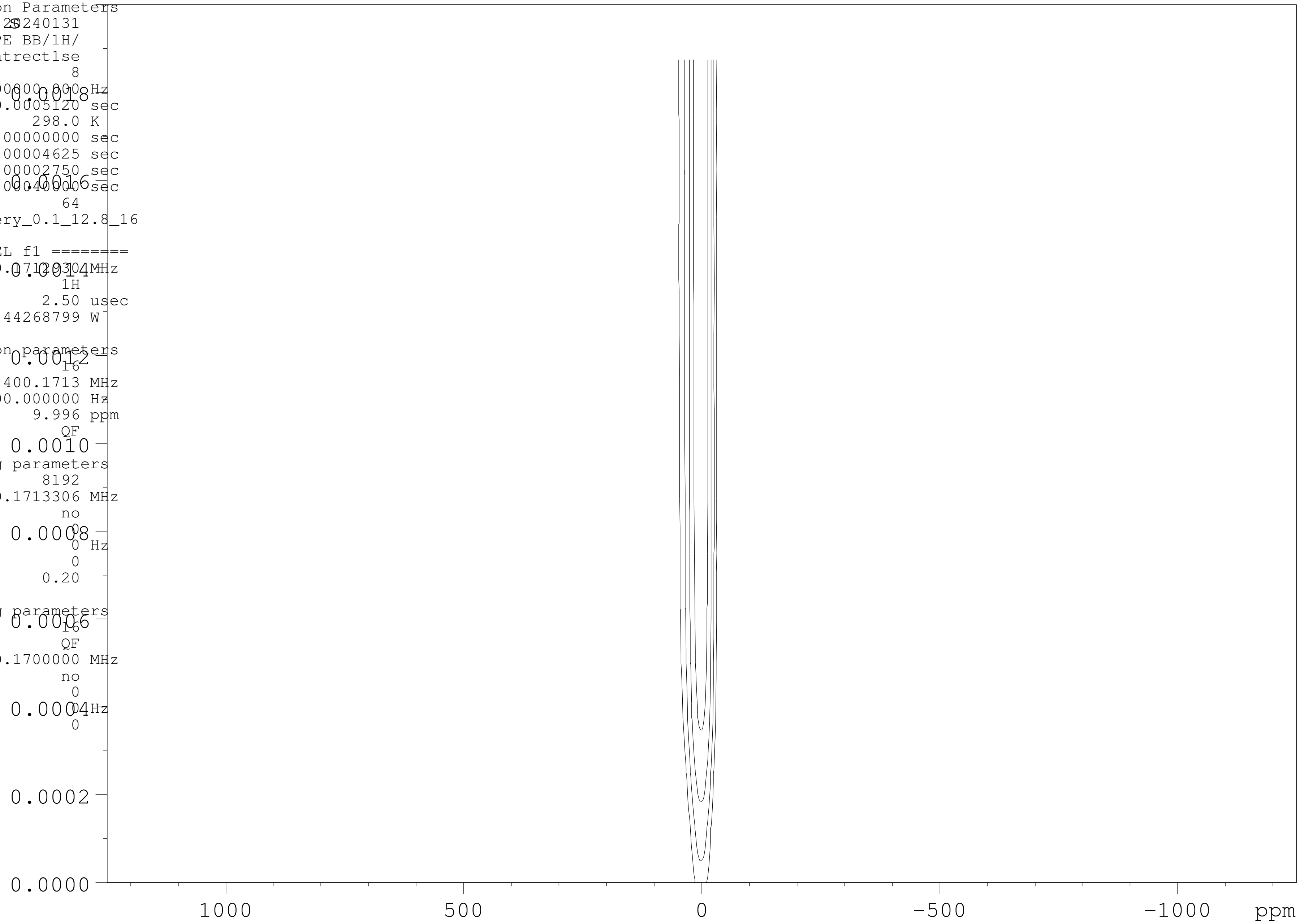

Current Data Parameters
NAME jse_20240130
EXPNO 49
PROCNO 1

2624-10340 SYT0043a S-CTA CdCl4 @ static / -100 to +120 C
-10 C

F2 - Acquisition Parameters
Date_ 20240131
PROBHD 5 mm PE BB/1H/
PULPROG t1rho_solidecho
NS 8
SWH 1000000.000 Hz
AQ 0.0005120 sec
TE 280.5 K
D1 7.19999981 sec
D6 0.00005000 sec
D7 0.00002756 sec

======== CHANNEL f1 ========
SFO1 400.1712930 MHz
NUC1 1H
P1 2.50 usec
PLW1 378.44268799 W
PLW2 94.62400055 W
VPLIST 100u_52000u_16

F1 - Acquisition parameters
TD 8
SFO1 400.1713 MHz
FIDRES 1000.000000 Hz
SW 9.996 ppm
FnMODE QF

F2 - Processing parameters
SI 8192
SF 400.1713306 MHz
WDW no
SSB 0
LB 0 Hz
GB 0
PC 0.20

F1 - Processing parameters
SI 16
MC2 QF
SF 400.1700000 MHz
WDW no
SSB 0
LB 0 Hz
GB 0

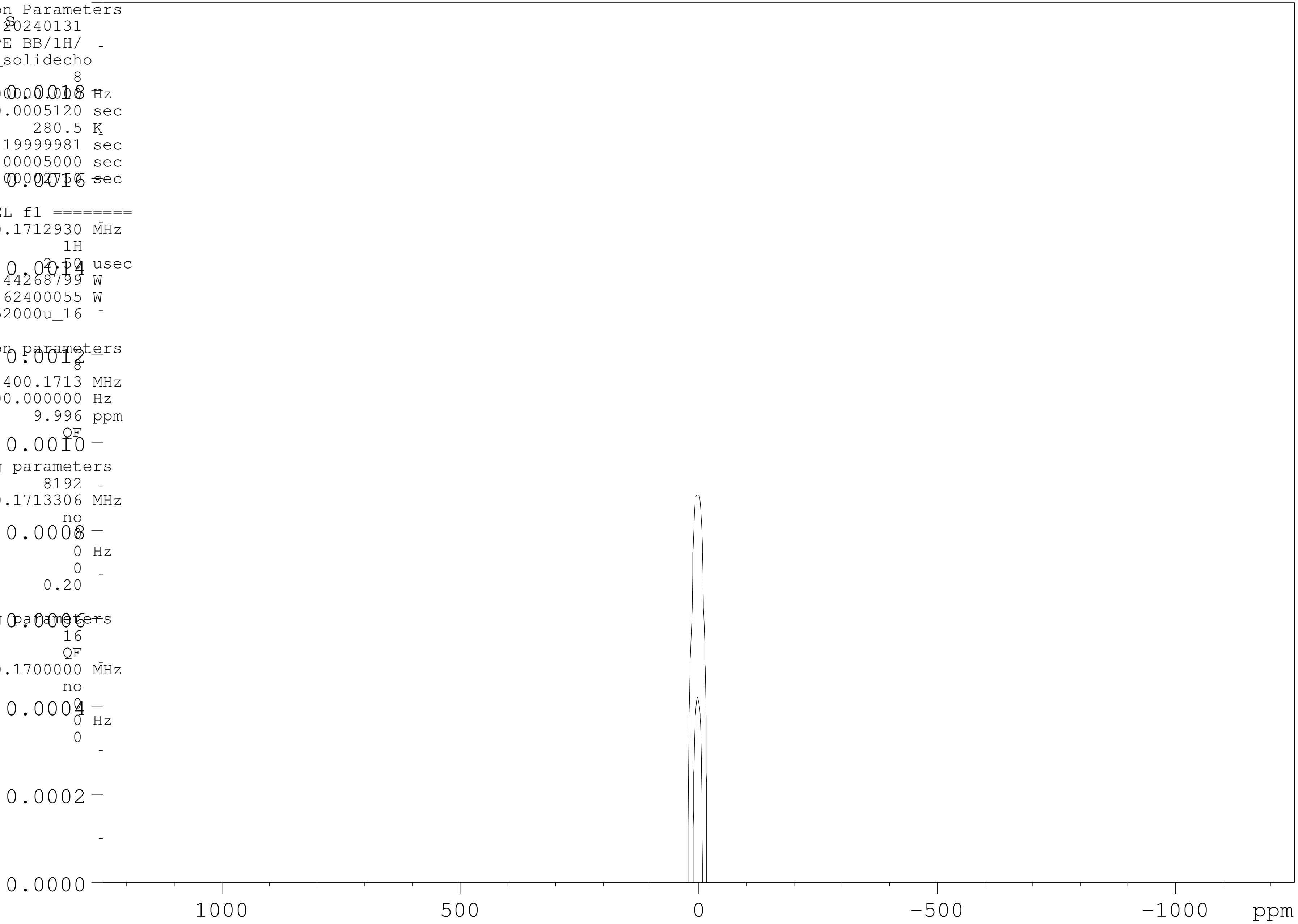

Current Data Parameters
NAME jse_20240130
EXPNO 51
PROCNO 1

F2 - Acquisition Parameters
Date_ 20240131
PROBHD 5 mm PE BB/1H/
PULPROG solidecho
NS 8
SWH 1000000.000 Hz
AQ 0.0005120 sec
TE 213.4 K
D1 7.19999981 sec
D6 0.00005000 sec
D7 0.00002750 sec

======== CHANNEL f1 ========
SFO1 400.1712930 MHz
NUC1 1H
P1 2.50 usec
PLW1 378.44268799 W

F2 - Processing parameters
SI 8192
SF 400.1713306 MHz
WDW EM
SSB 0
LB 10.00 Hz
GB 0
PC 0.20

2624-10340 SYT0043a S-CTA CdCl4 @ static / -100 to +120 C
0 C

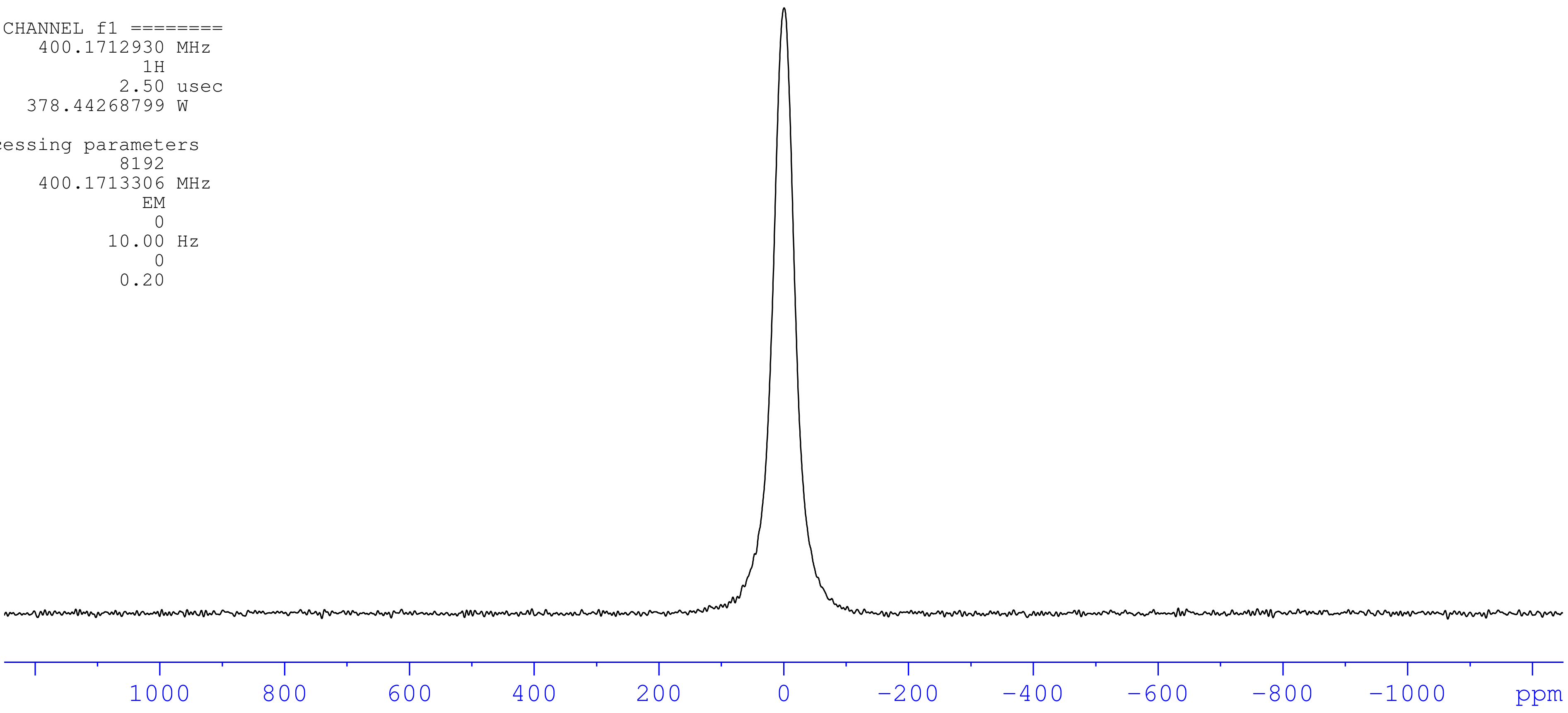

Current Data Parameters
NAME jse_20240130
EXPNO 52
PROCNO 1

F2 - Acquisition Parameters
Date_ 20240131
PROBHD 5 mm PE BB/1H/
PULPROG zg
NS 4
SWH 1000000.000 Hz
AQ 0.0005120 sec
TE 294.0 K
D1 7.19999981 sec
TD0 1

======== CHANNEL f1 ========
SFO1 400.1712930 MHz
NUC1 1H
P1 2.50 usec
PLW1 378.44268799 W

F2 - Processing parameters
SI 8192
SF 400.1713306 MHz
WDW EM
SSB 0
LB 10.00 Hz
GB 0
PC 0.20

2624-10340 SYT0043a S-CTA CdCl4 @ static / -100 to +120 C
0 C

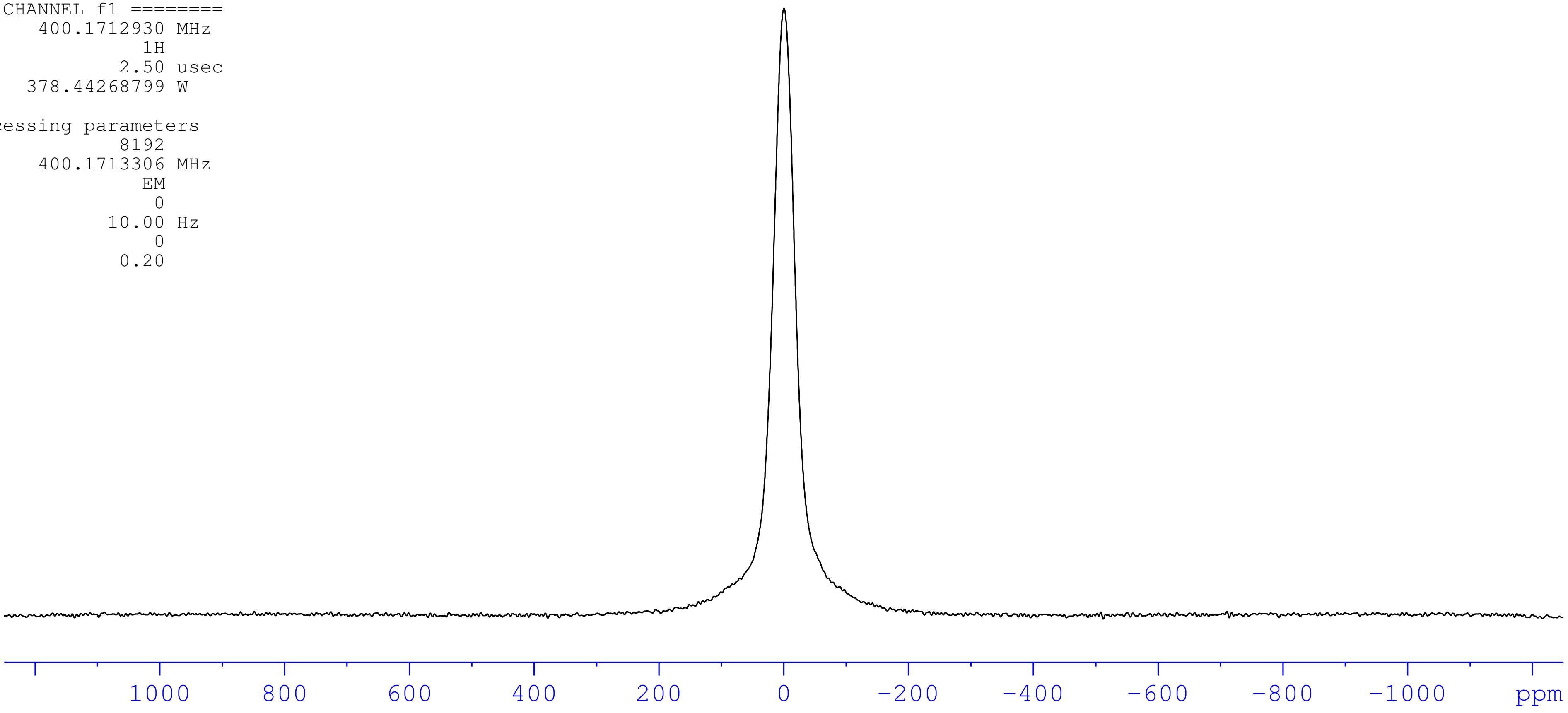

Current Data Parameters
NAME jse_20240130
EXPNO 53
PROCNO 1

F2 - Acquisition Parameters
Date_ 20240131
PROBHD 5 mm PE BB/1H/
PULPROG satrect1se
NS 8
SWH 1000000.000 Hz
AQ 0.0005120 sec
TE 298.0 K
D1 1.00000000 sec
D6 0.00004625 sec
D7 0.00002750 sec
D20 0.00040000 sec
L20 64
VDLIST Recovery_0.1_12.8_16

======== CHANNEL f1 ========
SFO1 400.1712930 MHz
NUC1 1H
P1 2.50 usec
PLW1 378.44268799 W

F1 - Acquisition parameters
TD 16
SFO1 400.1713 MHz
FIDRES 500.000000 Hz
SW 9.996 ppm
FnMODE QF

F2 - Processing parameters
SI 8192
SF 400.1713306 MHz
WDW no
SSB 0
LB 0 Hz
GB 0
PC 0.20

F1 - Processing parameters
SI 16
MC2 QF
SF 400.1700000 MHz
WDW no
SSB 0
LB 0 Hz
GB 0

2624-10340 SYT0043a S-CTA CdCl4 @ static / -100 to +120 C
0 C

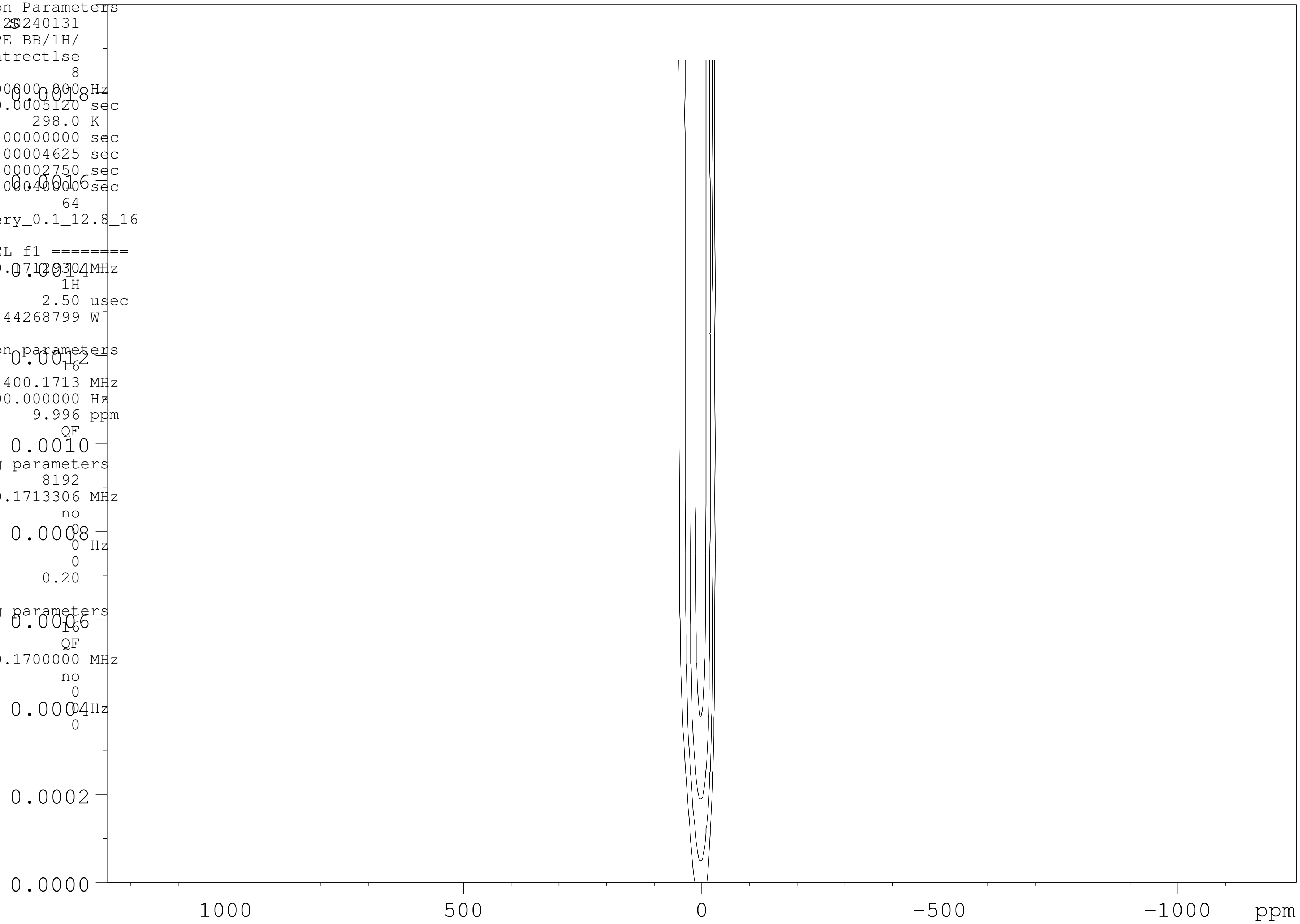

Current Data Parameters
NAME jse_20240130
EXPNO 54
PROCNO 1

F2 - Acquisition Parameters
Date_ 20240131
PROBHD 5 mm PE BB/1H/
PULPROG t1rho_solidecho
NS 8
SWH 1000000.000 Hz
AQ 0.0005120 sec
TE 287.2 K
D1 7.19999981 sec
D6 0.00005000 sec
D7 0.00002750 sec

======== CHANNEL f1 ========
SFO1 400.1712930 MHz
NUC1 1H
P1 2.50 usec
PLW1 378.44268799 W
PLW2 94.62400055 W
VPLIST 100u_52000u_16

F1 - Acquisition parameters
TD 8
SFO1 400.1713 MHz
FIDRES 1000.000000 Hz
SW 9.996 ppm
FnMODE QF

F2 - Processing parameters
SI 8192
SF 400.1713306 MHz
WDW no
SSB 0
LB 0 Hz
GB 0
PC 0.20

F1 - Processing parameters
SI 16
MC2 QF
SF 400.1700000 MHz
WDW no
SSB 0
LB 0 Hz
GB 0

2624-10340 SYT0043a S-CTA CdCl4 @ static / -100 to +120 C
0 C

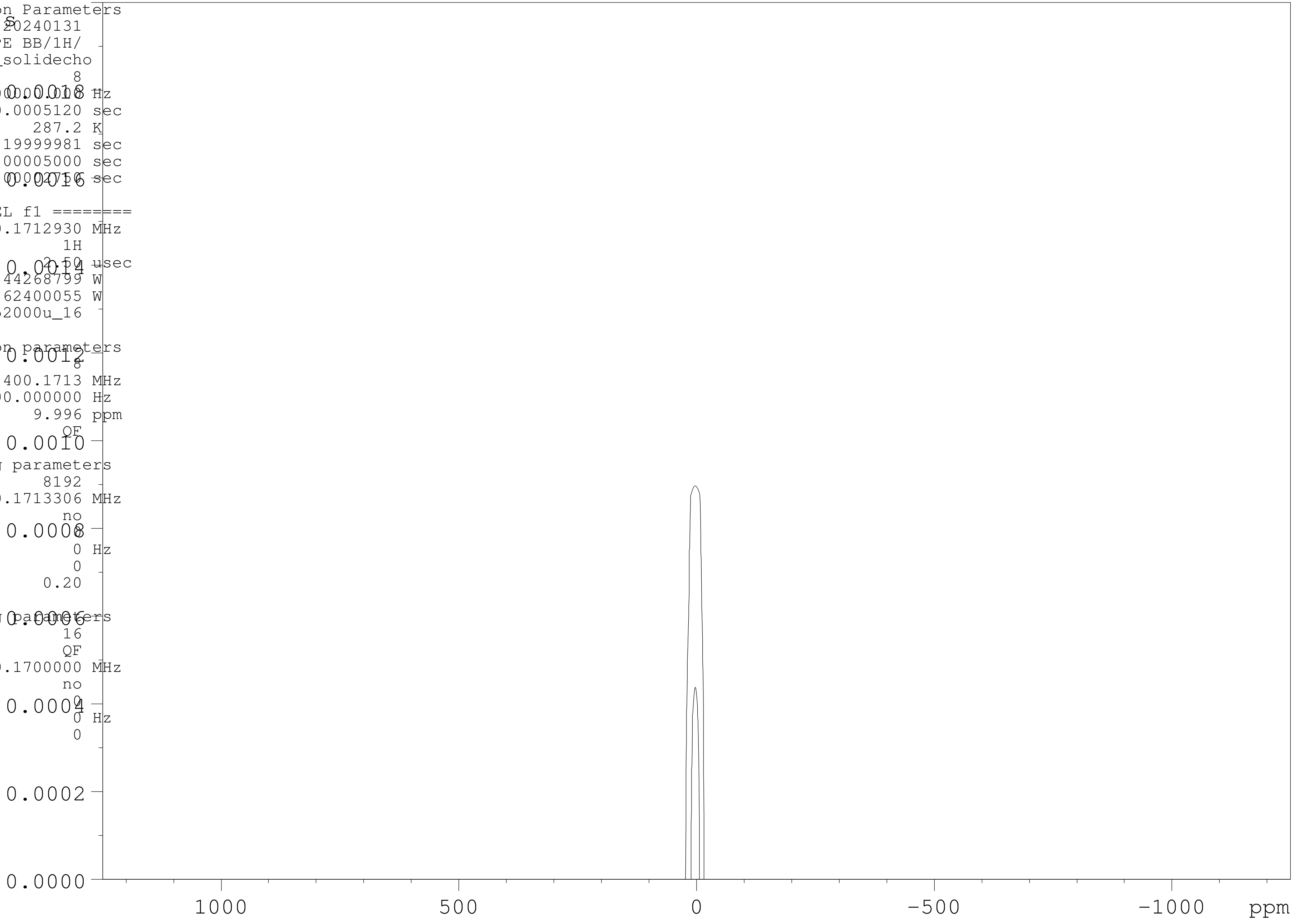

Current Data Parameters
NAME jse_20240130
EXPNO 56
PROCNO 1

F2 - Acquisition Parameters
Date_ 20240131
PROBHD 5 mm PE BB/1H/
PULPROG solidecho
NS 8
SWH 1000000.000 Hz
AQ 0.0005120 sec
TE 213.4 K
D1 7.19999981 sec
D6 0.00005000 sec
D7 0.00002750 sec

======== CHANNEL f1 ========
SFO1 400.1712930 MHz
NUC1 1H
P1 2.50 usec
PLW1 378.44268799 W

F2 - Processing parameters
SI 8192
SF 400.1713306 MHz
WDW EM
SSB 0
LB 10.00 Hz
GB 0
PC 0.20

2624-10340 SYT0043a S-CTA CdCl4 @ static / -100 to +120 C
10 C

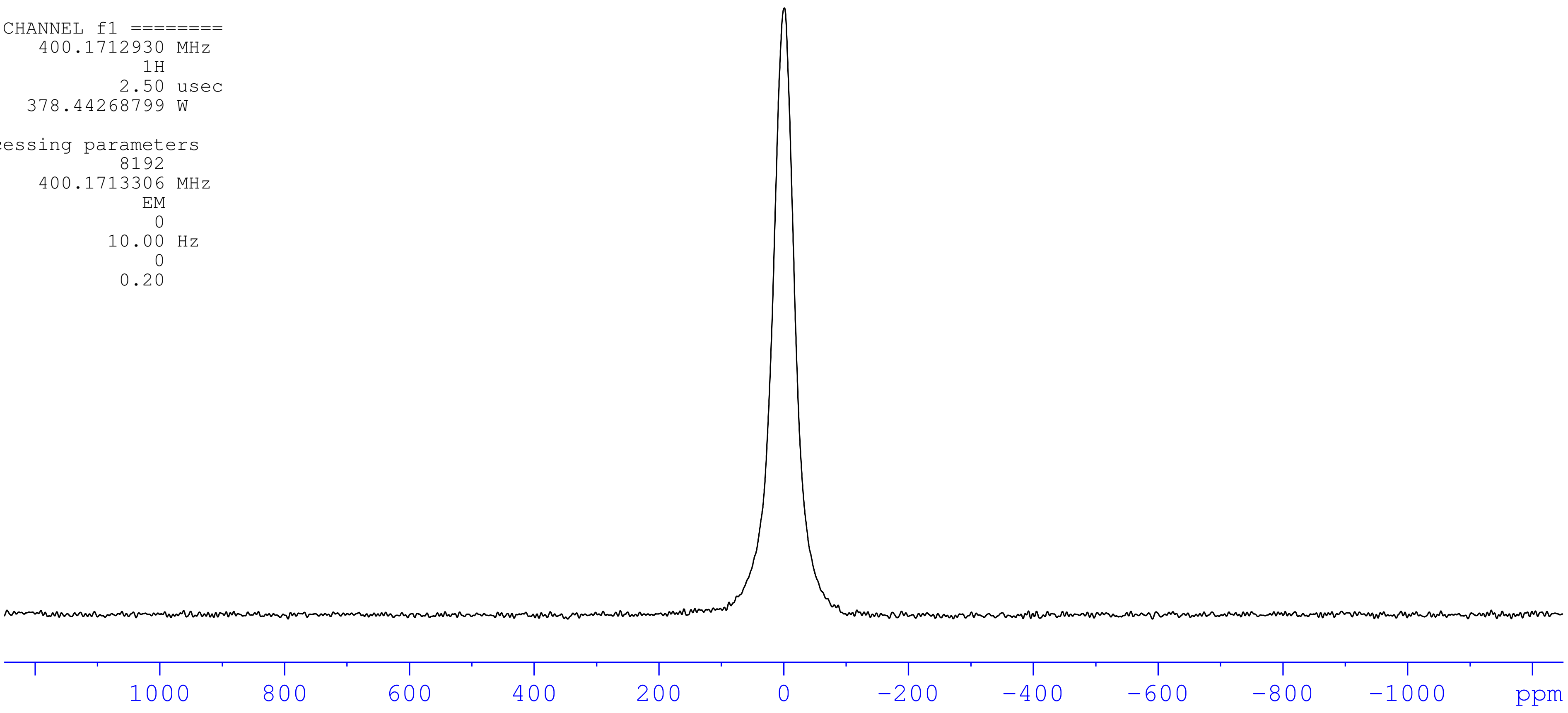

2624-10340 SYT0043a S-CTA CdCl4 @ static / -100 to +120 C
10 C

```
Current Data Parameters
NAME       jse_20240130
EXPNO                57
PROCNO                1

F2 - Acquisition Parameters
Date_          20240131
PROBHD    5 mm PE BB/1H/
PULPROG              zg
NS                    4
SWH         1000000.000 Hz
AQ            0.0005120 sec
TE                294.0 K
D1           7.19999981 sec
TD0                   1

======== CHANNEL f1 ========
SFO1        400.1712930 MHz
NUC1                 1H
P1                 2.50 usec
PLW1       378.44268799 W

F2 - Processing parameters
SI                 8192
SF          400.1713306 MHz
WDW                  EM
SSB                   0
LB                10.00 Hz
GB                    0
PC                 0.20
```

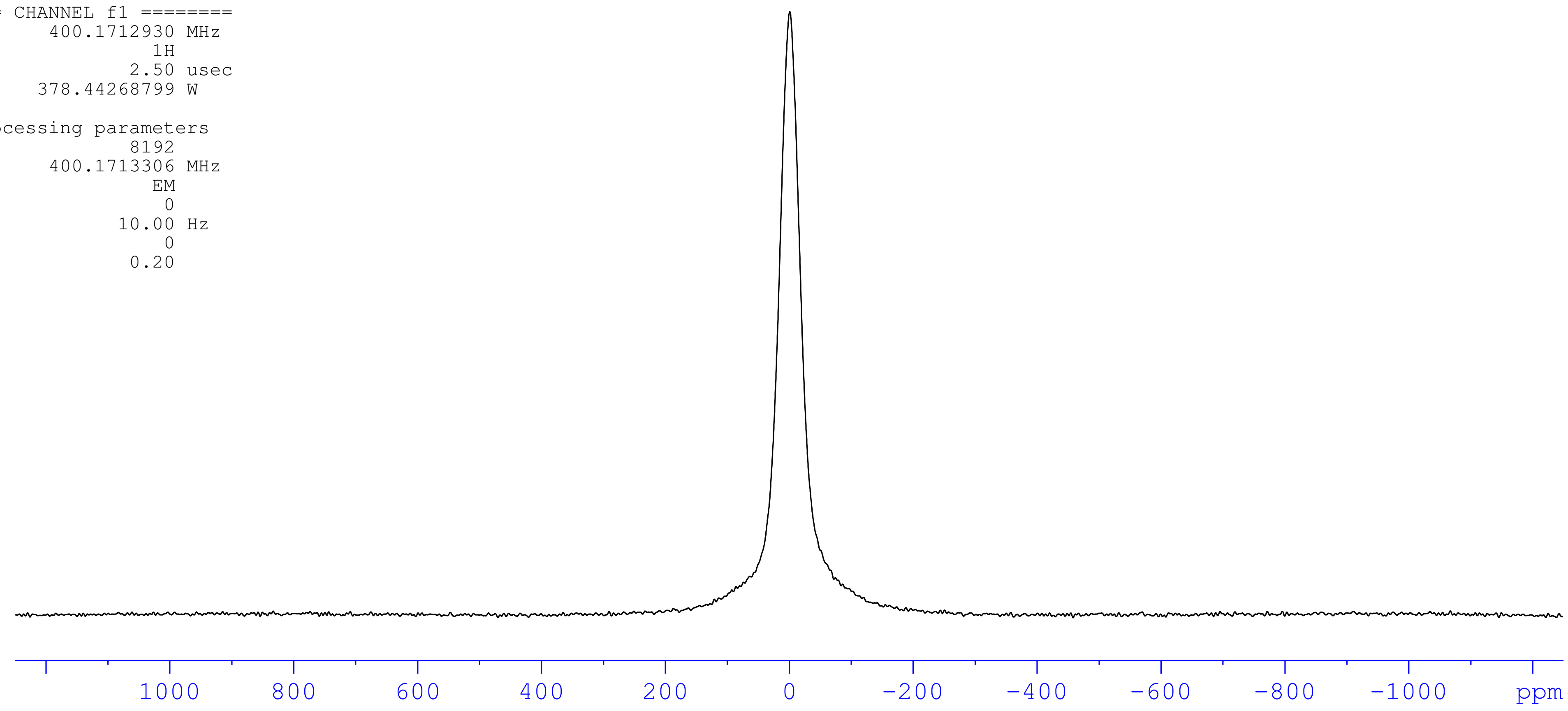

Current Data Parameters
NAME jse_20240130
EXPNO 58
PROCNO 1

F2 - Acquisition Parameters
Date_ 20240131
PROBHD 5 mm PE BB/1H/
PULPROG satrect1se
NS 8
SWH 1000000.000 Hz
AQ 0.0005120 sec
TE 298.0 K
D1 1.00000000 sec
D6 0.00004625 sec
D7 0.00002750 sec
D20 0.00040000 sec
L20 64
VDLIST Recovery_0.1_102.4_16

======== CHANNEL f1 ========
SFO1 400.1712930 MHz
NUC1 1H
P1 2.50 usec
PLW1 378.44268799 W

F1 - Acquisition parameters
TD 16
SFO1 400.1713 MHz
FIDRES 500.000000 Hz
SW 9.996 ppm
FnMODE QF

F2 - Processing parameters
SI 8192
SF 400.1713306 MHz
WDW no
SSB 0
LB 0 Hz
GB 0
PC 0.20

F1 - Processing parameters
SI 16
MC2 QF
SF 400.1700000 MHz
WDW no
SSB 0
LB 0 Hz
GB 0

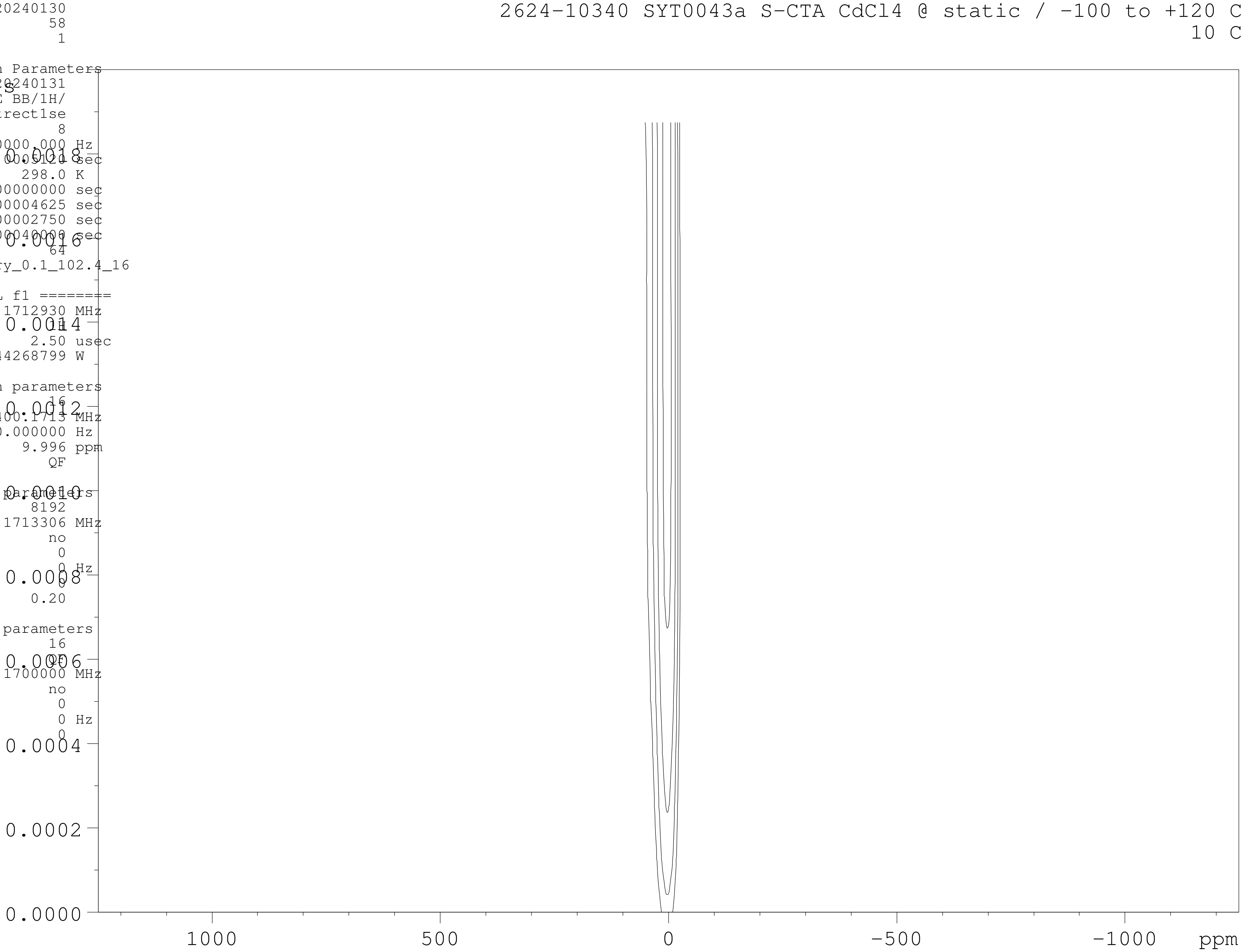

Current Data Parameters
NAME jse_20240130
EXPNO 59
PROCNO 1

F2 - Acquisition Parameters
Date_ 20240131
PROBHD 5 mm PE BB/1H/
PULPROG t1rho_solidecho
NS 8
SWH 1000000.000 Hz
AQ 0.0005120 sec
TE 220.1 K
D1 7.19999981 sec
D6 0.00005000 sec
D7 0.00002750 sec

======== CHANNEL f1 ========
SFO1 400.1712930 MHz
NUC1 1H
P1 2.50 usec
PLW1 378.44268799 W
PLW2 94.62400055 W
VPLIST 100u_52000u_16

F1 - Acquisition parameters
TD 8
SFO1 400.1713 MHz
FIDRES 1000.000000 Hz
SW 9.996 ppm
FnMODE QF

F2 - Processing parameters
SI 8192
SF 400.1713306 MHz
WDW no
SSB 0
LB 0 Hz
GB 0
PC 0.20

F1 - Processing parameters
SI 16
MC2 QF
SF 400.1700000 MHz
WDW no
SSB 0
LB 0 Hz
GB 0

2624-10340 SYT0043a S-CTA CdCl4 @ static / -100 to +120 C
10 C

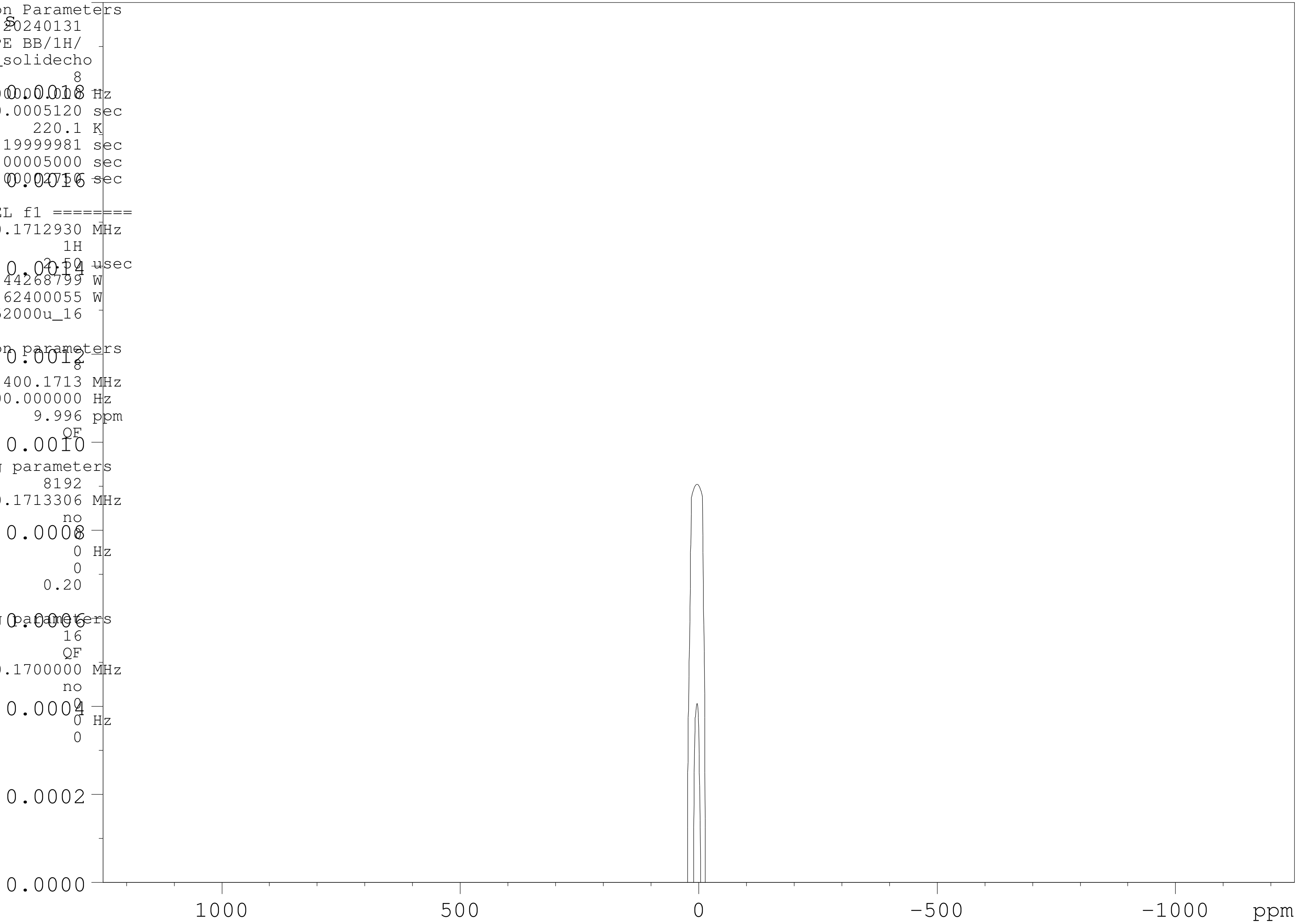

Current Data Parameters
NAME jse_20240130
EXPNO 61
PROCNO 1

F2 - Acquisition Parameters
Date_ 20240201
PROBHD 5 mm PE BB/1H/
PULPROG solidecho
NS 8
SWH 1000000.000 Hz
AQ 0.0005120 sec
TE 294.0 K
D1 7.19999981 sec
D6 0.00005000 sec
D7 0.00002750 sec

======== CHANNEL f1 ========
SFO1 400.1712930 MHz
NUC1 1H
P1 2.50 usec
PLW1 378.44268799 W

F2 - Processing parameters
SI 8192
SF 400.1713306 MHz
WDW EM
SSB 0
LB 10.00 Hz
GB 0
PC 0.20

2624-10340 SYT0043a S-CTA CdCl4 @ static / -100 to +120 C
20 C

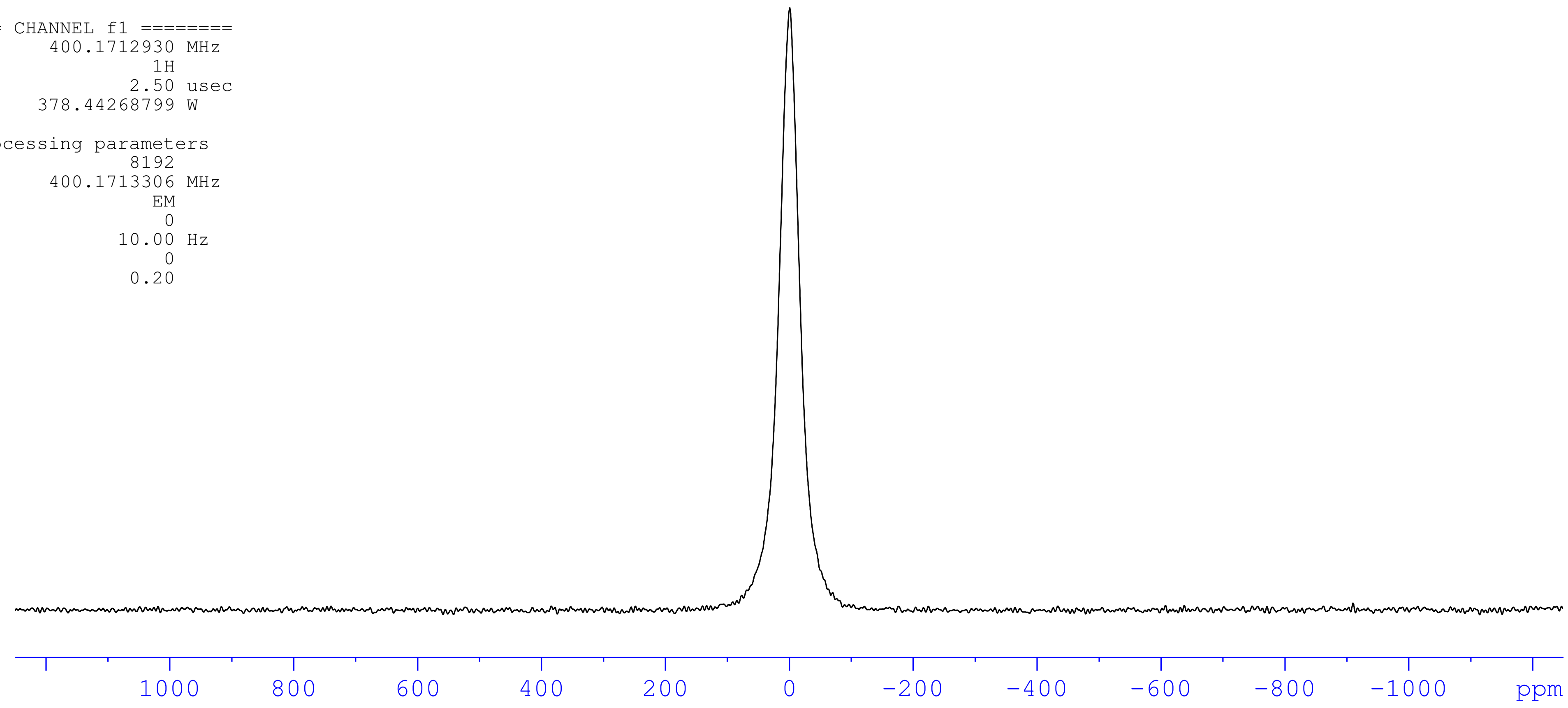

```
Current Data Parameters
NAME        jse_20240130
EXPNO                 62
PROCNO                 1

F2 - Acquisition Parameters
Date_           20240201
PROBHD    5 mm PE BB/1H/
PULPROG               zg
NS                     4
SWH          1000000.000 Hz
AQ             0.0005120 sec
TE                 294.0 K
D1            7.19999981 sec
TD0                    1

======== CHANNEL f1 ========
SFO1         400.1712930 MHz
NUC1                  1H
P1                  2.50 usec
PLW1        378.44268799 W

F2 - Processing parameters
SI                  8192
SF           400.1713306 MHz
WDW                   EM
SSB                    0
LB                 10.00 Hz
GB                     0
PC                  0.20
```

2624-10340 SYT0043a S-CTA CdCl4 @ static / -100 to +120 C
20 C

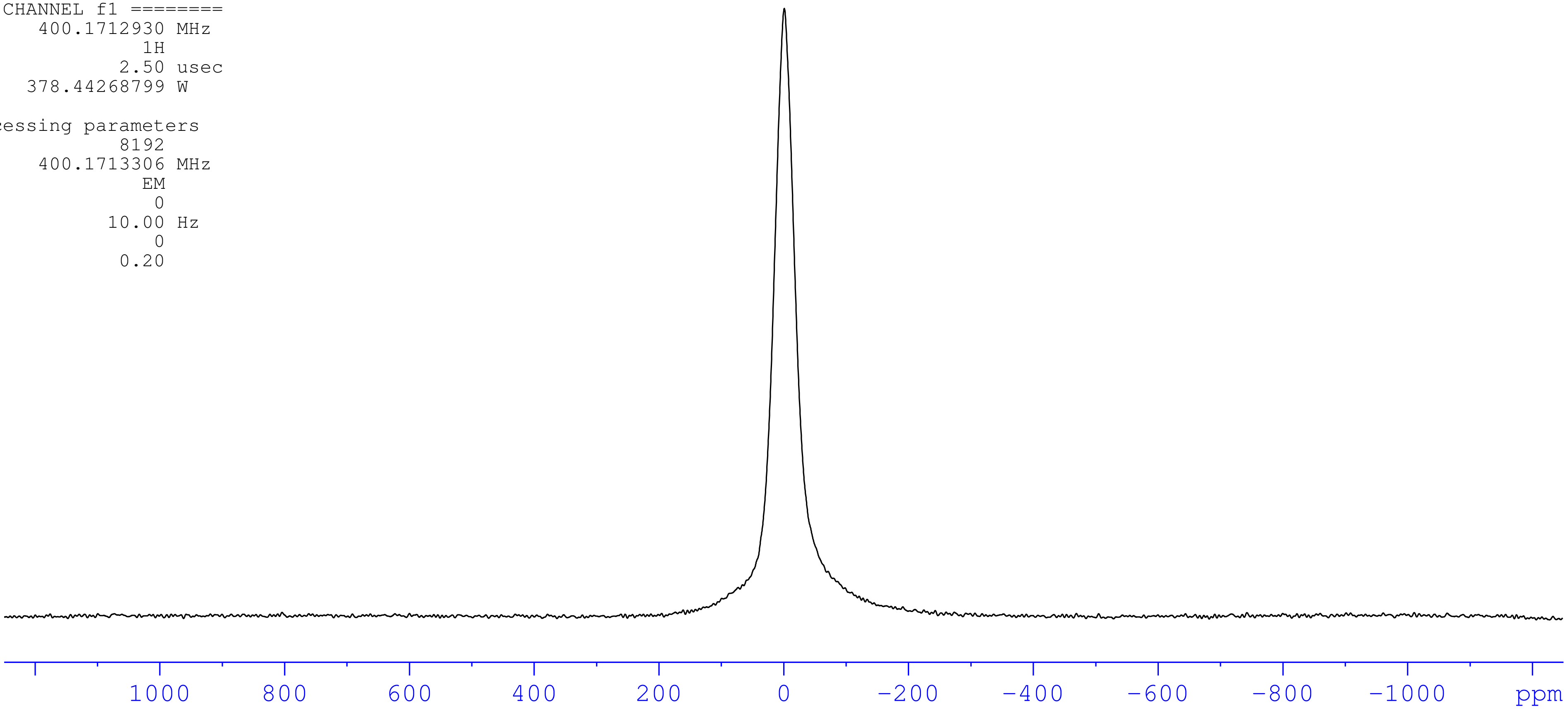

Current Data Parameters
NAME jse_20240130
EXPNO 63
PROCNO 1

F2 - Acquisition Parameters
Date_ 20240201
PROBHD 5 mm PE BB/1H/
PULPROG satrect1se
NS 8
SWH 1000000.000 Hz
AQ 0.0005120 sec
TE 298.0 K
D1 1.00000000 sec
D6 0.00004625 sec
D7 0.00002750 sec
D20 0.00040000 sec
L20 64
VDLIST Recovery_0.1_102.4_16

======== CHANNEL f1 ========
SFO1 400.1712930 MHz
NUC1 1H
P1 2.50 usec
PLW1 378.44268799 W

F1 - Acquisition parameters
TD 16
SFO1 400.1713 MHz
FIDRES 500.000000 Hz
SW 9.996 ppm
FnMODE QF

F2 - Processing parameters
SI 8192
SF 400.1713306 MHz
WDW no
SSB 0
LB 0 Hz
GB 0
PC 0.20

F1 - Processing parameters
SI 16
MC2 QF
SF 400.1700000 MHz
WDW no
SSB 0
LB 0 Hz
GB 0

2624-10340 SYT0043a S-CTA CdCl4 @ static / -100 to +120 C
20 C

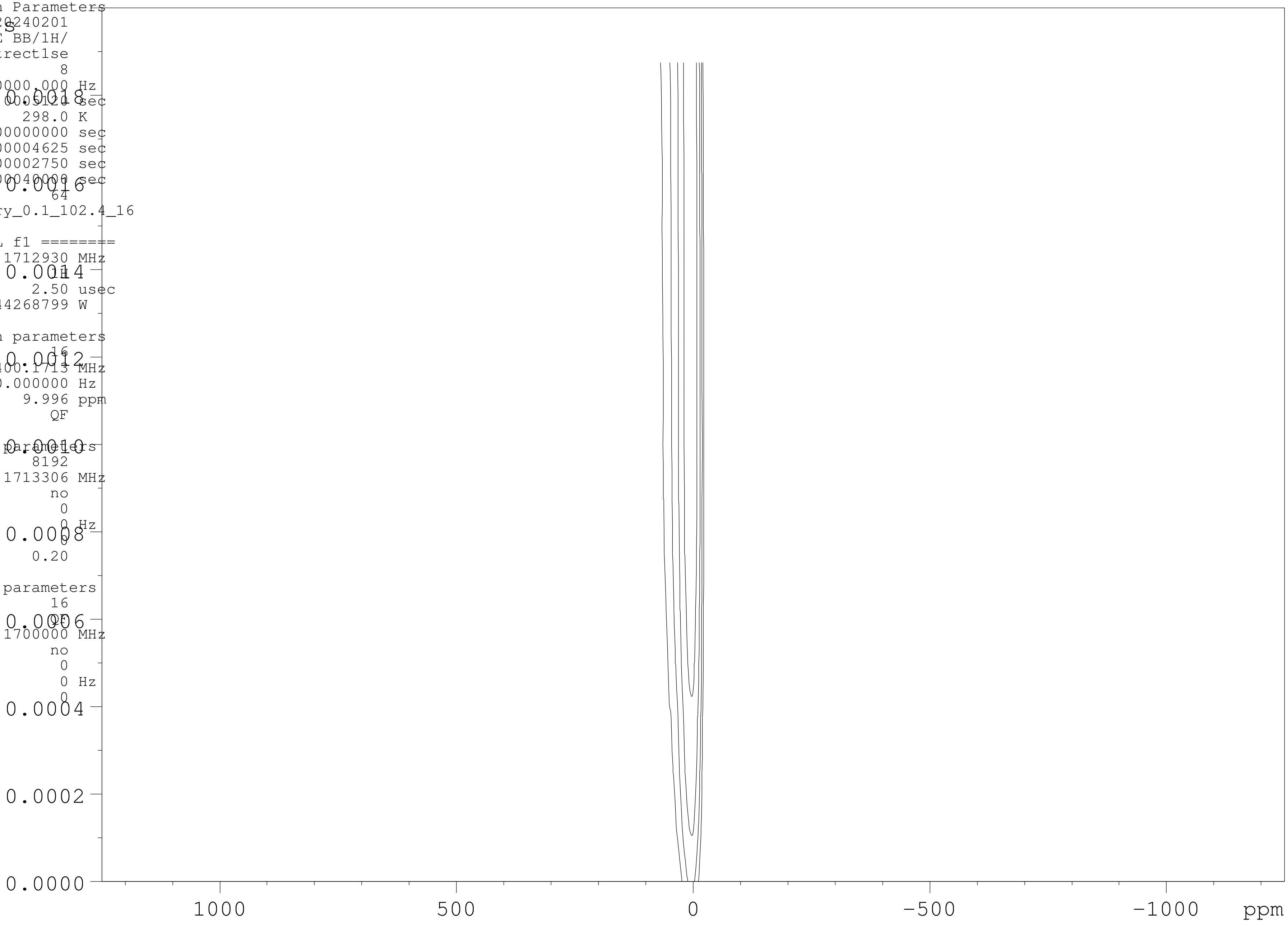

2624-10340 SYT0043a S-CTA CdCl4 @ static / -100 to +120 C
20 C

Current Data Parameters
NAME jse_20240130
EXPNO 64
PROCNO 1

F2 - Acquisition Parameters
Date_ 20240201
PROBHD 5 mm PE BB/1H/
PULPROG t1rho_solidecho
NS 8
SWH 1000000.000 Hz
AQ 0.0005120 sec
TE 300.7 K
D1 7.19999981 sec
D6 0.00005000 sec
D7 0.00002750 sec

======== CHANNEL f1 ========
SFO1 400.1712930 MHz
NUC1 1H
P1 2.50 usec
PLW1 378.44268799 W
PLW2 94.62400055 W
VPLIST 100u_52000u_16

F1 - Acquisition parameters
TD 8
SFO1 400.1713 MHz
FIDRES 1000.000000 Hz
SW 9.996 ppm
FnMODE QF

F2 - Processing parameters
SI 8192
SF 400.1713306 MHz
WDW no
SSB 0
LB 0 Hz
GB 0
PC 0.20

F1 - Processing parameters
SI 16
MC2 QF
SF 400.1700000 MHz
WDW no
SSB 0
LB 0 Hz
GB 0

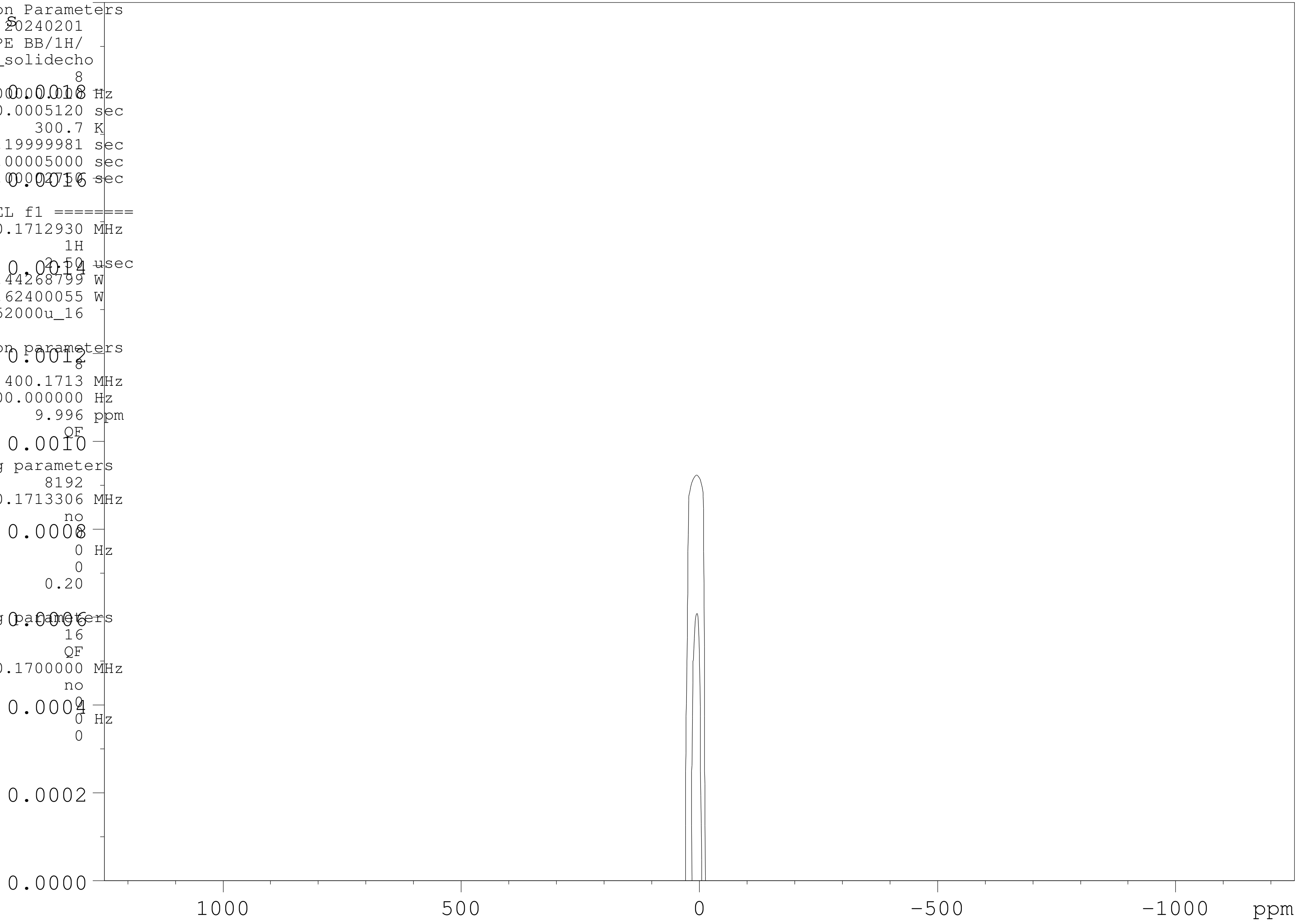

Current Data Parameters
NAME jse_20240130
EXPNO 66
PROCNO 1

F2 - Acquisition Parameters
Date_ 20240201
PROBHD 5 mm PE BB/1H/
PULPROG solidecho
NS 8
SWH 1000000.000 Hz
AQ 0.0005120 sec
TE 294.0 K
D1 7.19999981 sec
D6 0.00005000 sec
D7 0.00002750 sec

======== CHANNEL f1 ========
SFO1 400.1712930 MHz
NUC1 1H
P1 2.50 usec
PLW1 378.44268799 W

F2 - Processing parameters
SI 8192
SF 400.1713306 MHz
WDW EM
SSB 0
LB 10.00 Hz
GB 0
PC 0.20

2624-10340 SYT0043a S-CTA CdCl4 @ static / -100 to +120 C
30 C

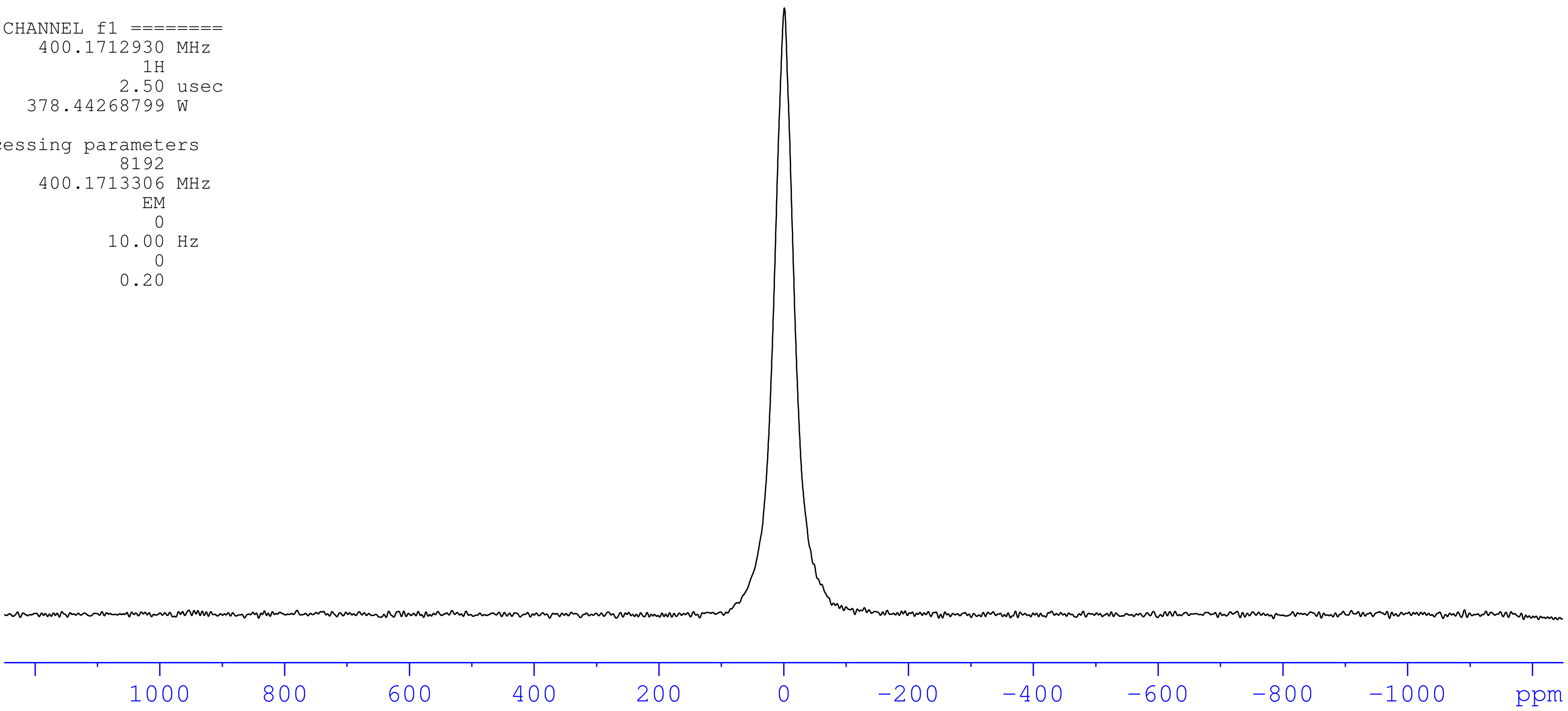

Current Data Parameters
NAME jse_20240130
EXPNO 67
PROCNO 1

F2 - Acquisition Parameters
Date_ 20240201
PROBHD 5 mm PE BB/1H/
PULPROG zg
NS 4
SWH 1000000.000 Hz
AQ 0.0005120 sec
TE 294.0 K
D1 7.19999981 sec
TD0 1

======== CHANNEL f1 ========
SFO1 400.1712930 MHz
NUC1 1H
P1 2.50 usec
PLW1 378.44268799 W

F2 - Processing parameters
SI 8192
SF 400.1713306 MHz
WDW EM
SSB 0
LB 10.00 Hz
GB 0
PC 0.20

2624-10340 SYT0043a S-CTA CdCl4 @ static / -100 to +120 C
30 C

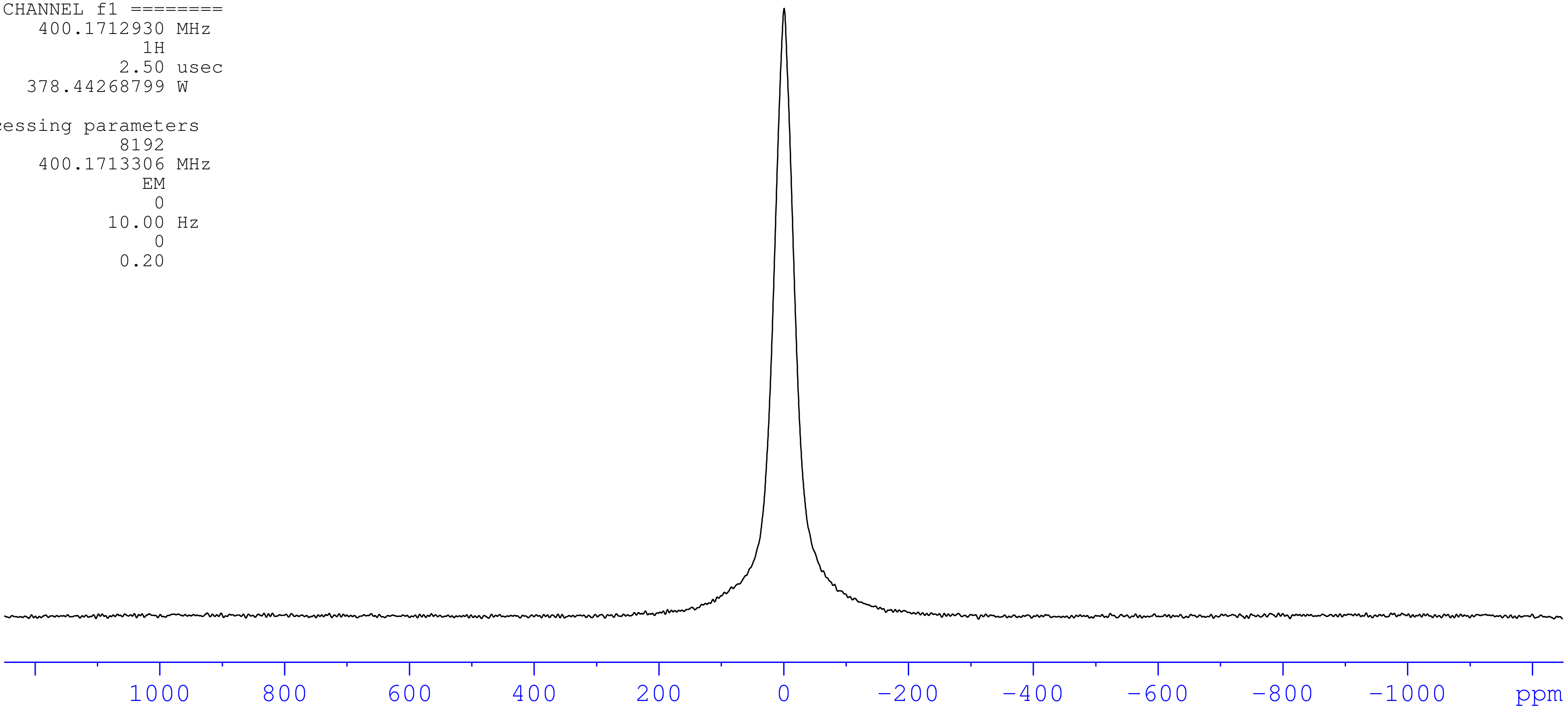

2624-10340 SYT0043a S-CTA CdCl4 @ static / -100 to +120 C
30 C

```
Current Data Parameters
NAME        jse_20240130
EXPNO                 68
PROCNO                 1

F2 - Acquisition Parameters
Date_           20240201
PROBHD   5 mm PE BB/1H/
PULPROG       satrect1se
NS                     8
SWH       1000000.000 Hz
AQ          0.0005120 sec
TE              298.0 K
D1         1.00000000 sec
D6         0.00004625 sec
D7         0.00002750 sec
D20        0.00040000 sec
L20                   64
VDLIST   Recovery_0.1_102.4_16

======== CHANNEL f1 ========
SFO1      400.1712930 MHz
NUC1                  1H
P1               2.50 usec
PLW1      378.44268799 W

F1 - Acquisition parameters
TD                    16
SFO1         400.1713 MHz
FIDRES     500.000000 Hz
SW              9.996 ppm
FnMODE                QF

F2 - Processing parameters
SI                  8192
SF        400.1713306 MHz
WDW                   no
SSB                    0
LB                   0 Hz
GB                     0
PC                  0.20

F1 - Processing parameters
SI                    16
MC2                   QF
SF        400.1700000 MHz
WDW                   no
SSB                    0
LB                   0 Hz
GB                     0
```

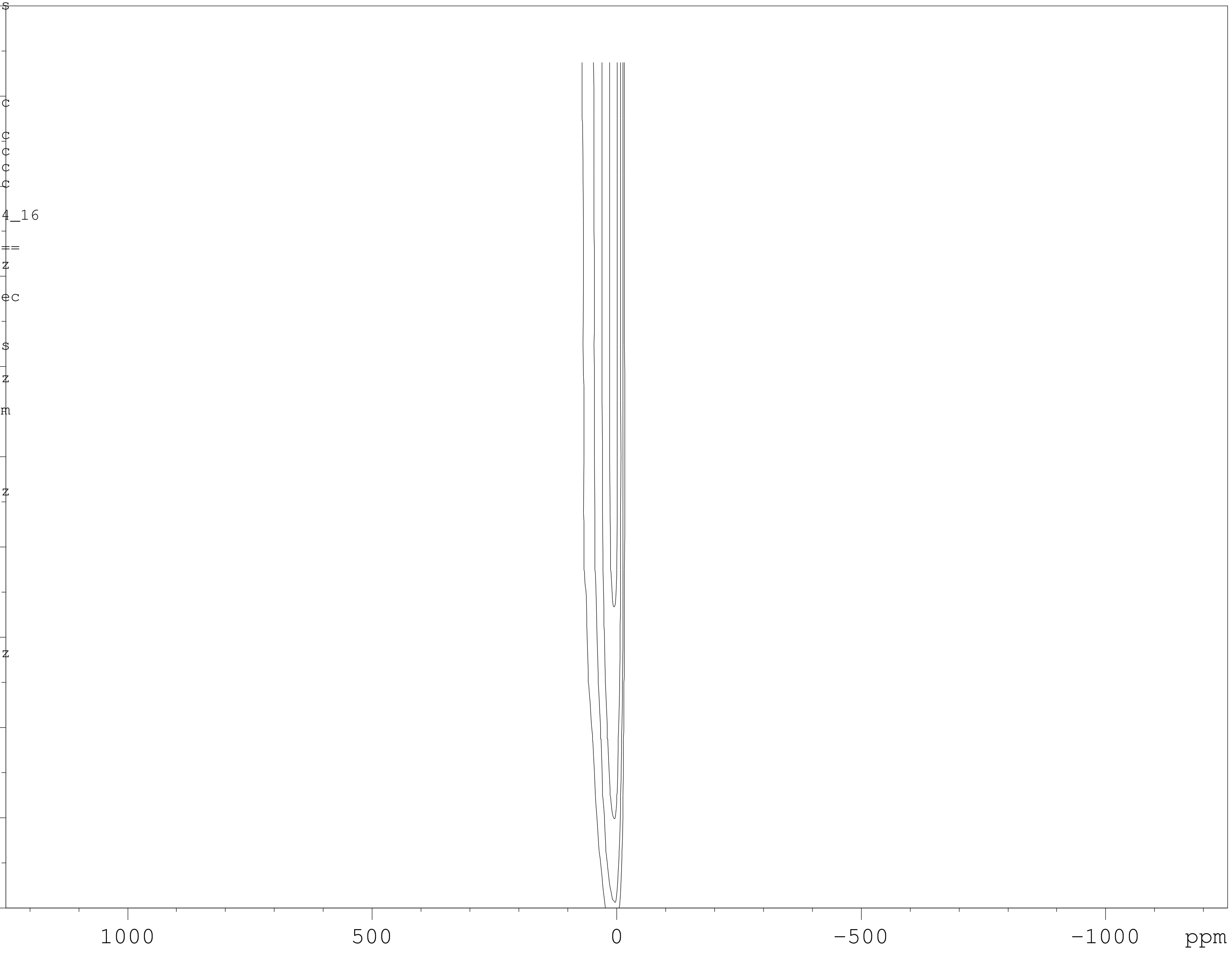

Current Data Parameters
NAME jse_20240130
EXPNO 69
PROCNO 1

F2 - Acquisition Parameters
Date_ 20240201
PROBHD 5 mm PE BB/1H/
PULPROG t1rho_solidecho
NS 8
SWH 1000000.000 Hz
AQ 0.0005120 sec
TE 307.4 K
D1 7.19999981 sec
D6 0.00005000 sec
D7 0.0000275[illegible] sec

======== CHANNEL f1 ========
SFO1 400.1712930 MHz
NUC1 1H
P1 2.50 usec
PLW1 378.44268799 W
PLW2 94.62400055 W
VPLIST 100u_52000u_16

F1 - Acquisition parameters
TD 8
SFO1 400.1713 MHz
FIDRES 1000.000000 Hz
SW 9.996 ppm
FnMODE QF

F2 - Processing parameters
SI 8192
SF 400.1713306 MHz
WDW no
SSB 0
LB 0 Hz
GB 0
PC 0.20

F1 - Processing parameters
SI 16
MC2 QF
SF 400.1700000 MHz
WDW no
SSB 0
LB 0 Hz
GB 0

2624-10340 SYT0043a S-CTA CdCl4 @ static / -100 to +120 C
30 C

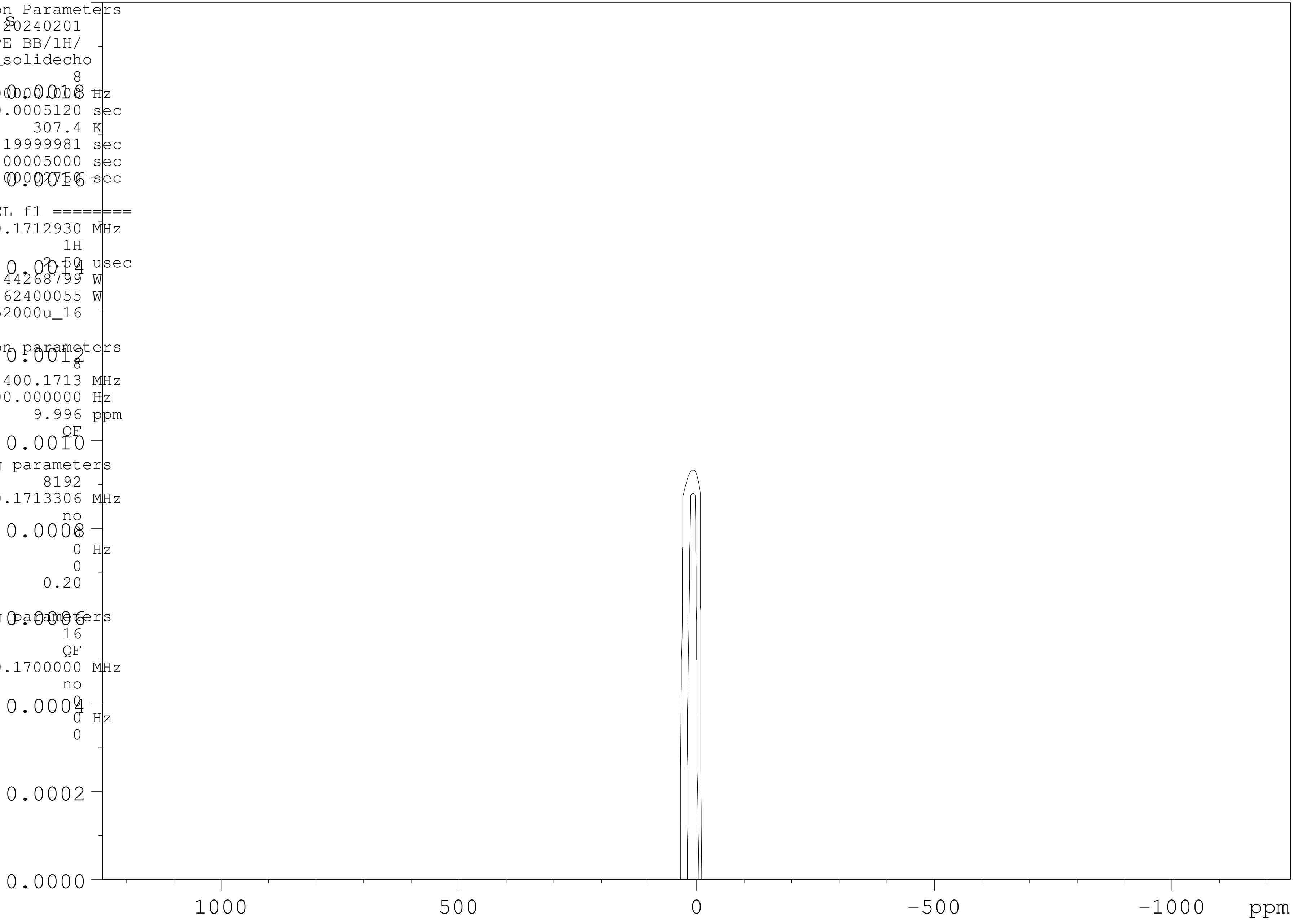

Current Data Parameters
NAME jse_20240130
EXPNO 71
PROCNO 1

F2 - Acquisition Parameters
Date_ 20240201
PROBHD 5 mm PE BB/1H/
PULPROG solidecho
NS 8
SWH 1000000.000 Hz
AQ 0.0005120 sec
TE 294.0 K
D1 7.19999981 sec
D6 0.00005000 sec
D7 0.00002750 sec

======== CHANNEL f1 ========
SFO1 400.1712930 MHz
NUC1 1H
P1 2.50 usec
PLW1 378.44268799 W

F2 - Processing parameters
SI 8192
SF 400.1713306 MHz
WDW EM
SSB 0
LB 10.00 Hz
GB 0
PC 0.20

2624-10340 SYT0043a S-CTA CdCl4 @ static / -100 to +120 C
40 C

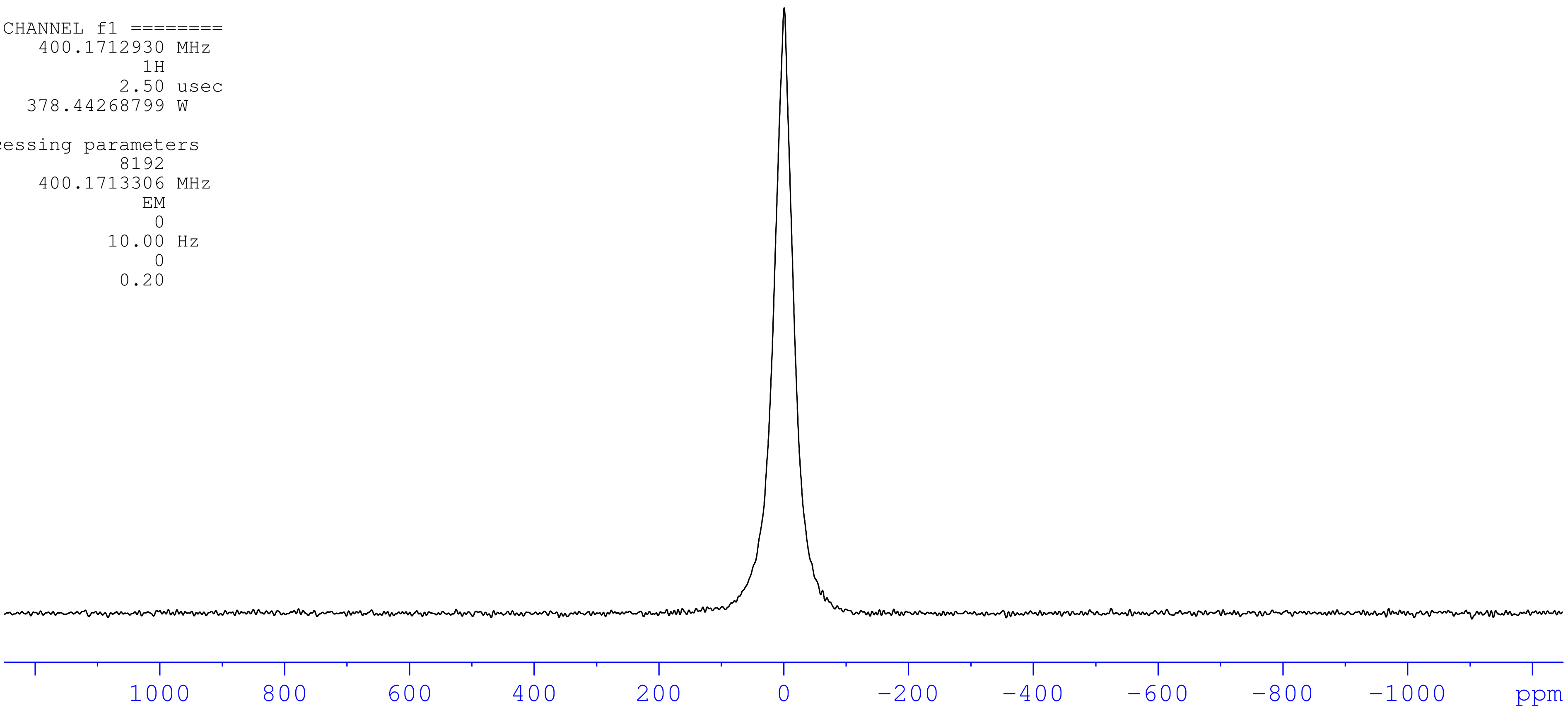

Current Data Parameters
NAME jse_20240130
EXPNO 72
PROCNO 1

F2 - Acquisition Parameters
Date_ 20240201
PROBHD 5 mm PE BB/1H/
PULPROG zg
NS 4
SWH 1000000.000 Hz
AQ 0.0005120 sec
TE 294.0 K
D1 7.19999981 sec
TD0 1

======== CHANNEL f1 ========
SFO1 400.1712930 MHz
NUC1 1H
P1 2.50 usec
PLW1 378.44268799 W

F2 - Processing parameters
SI 8192
SF 400.1713306 MHz
WDW EM
SSB 0
LB 10.00 Hz
GB 0
PC 0.20

2624-10340 SYT0043a S-CTA CdCl4 @ static / -100 to +120 C
40 C

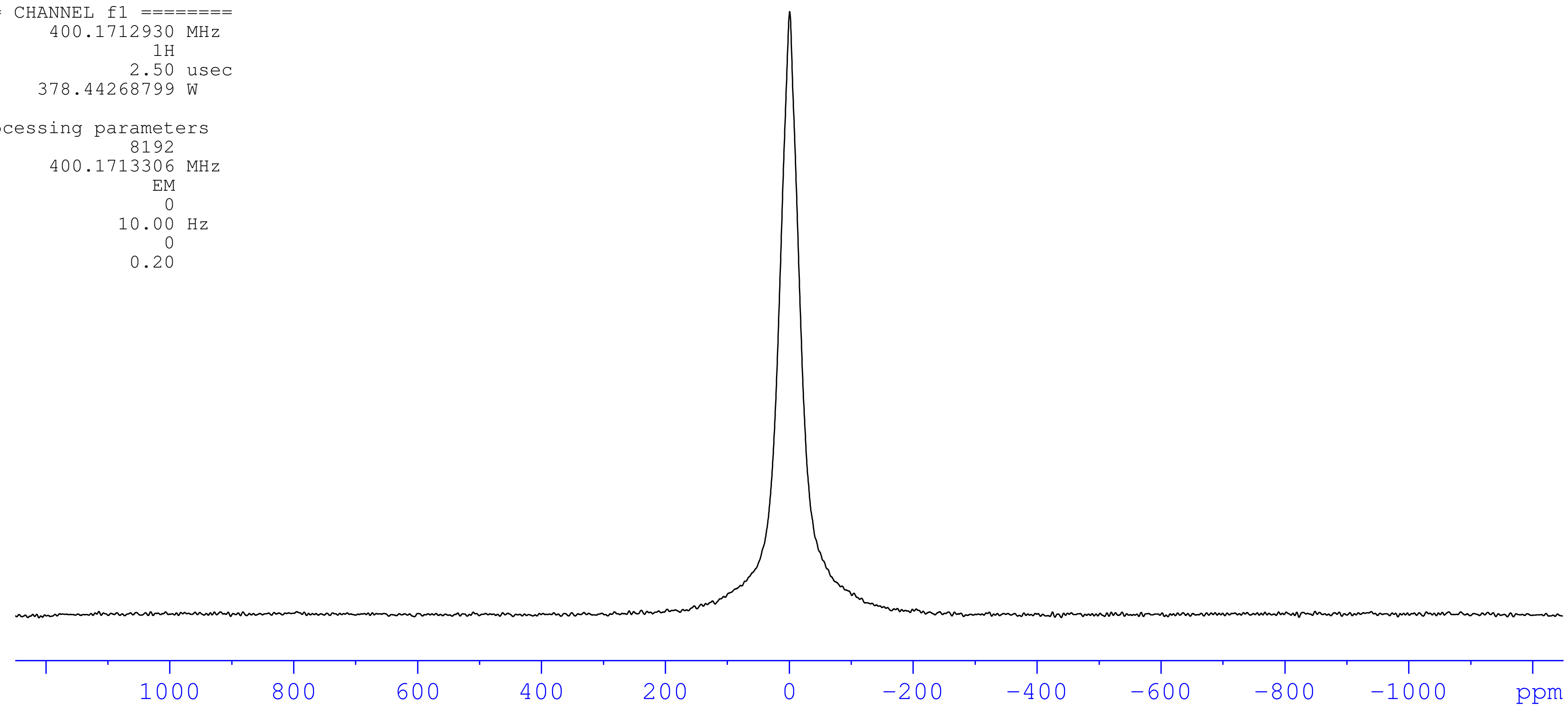

Current Data Parameters
NAME jse_20240130
EXPNO 73
PROCNO 1

F2 - Acquisition Parameters
Date_ 20240201
PROBHD 5 mm PE BB/1H/
PULPROG satrect1se
NS 8
SWH 1000000.000 Hz
AQ 0.0005120 sec
TE 298.0 K
D1 1.00000000 sec
D6 0.00004625 sec
D7 0.00002750 sec
D20 0.00040000 sec
L20 64
VDLIST Recovery_0.1_102.4_16

======== CHANNEL f1 ========
SFO1 400.1712930 MHz
NUC1 1H
P1 2.50 usec
PLW1 378.44268799 W

F1 - Acquisition parameters
TD 16
SFO1 400.1713 MHz
FIDRES 500.000000 Hz
SW 9.996 ppm
FnMODE QF

F2 - Processing parameters
SI 8192
SF 400.1713306 MHz
WDW no
SSB 0
LB 0 Hz
GB 0
PC 0.20

F1 - Processing parameters
SI 16
MC2 QF
SF 400.1700000 MHz
WDW no
SSB 0
LB 0 Hz
GB 0

2624-10340 SYT0043a S-CTA CdCl4 @ static / -100 to +120 C
40 C

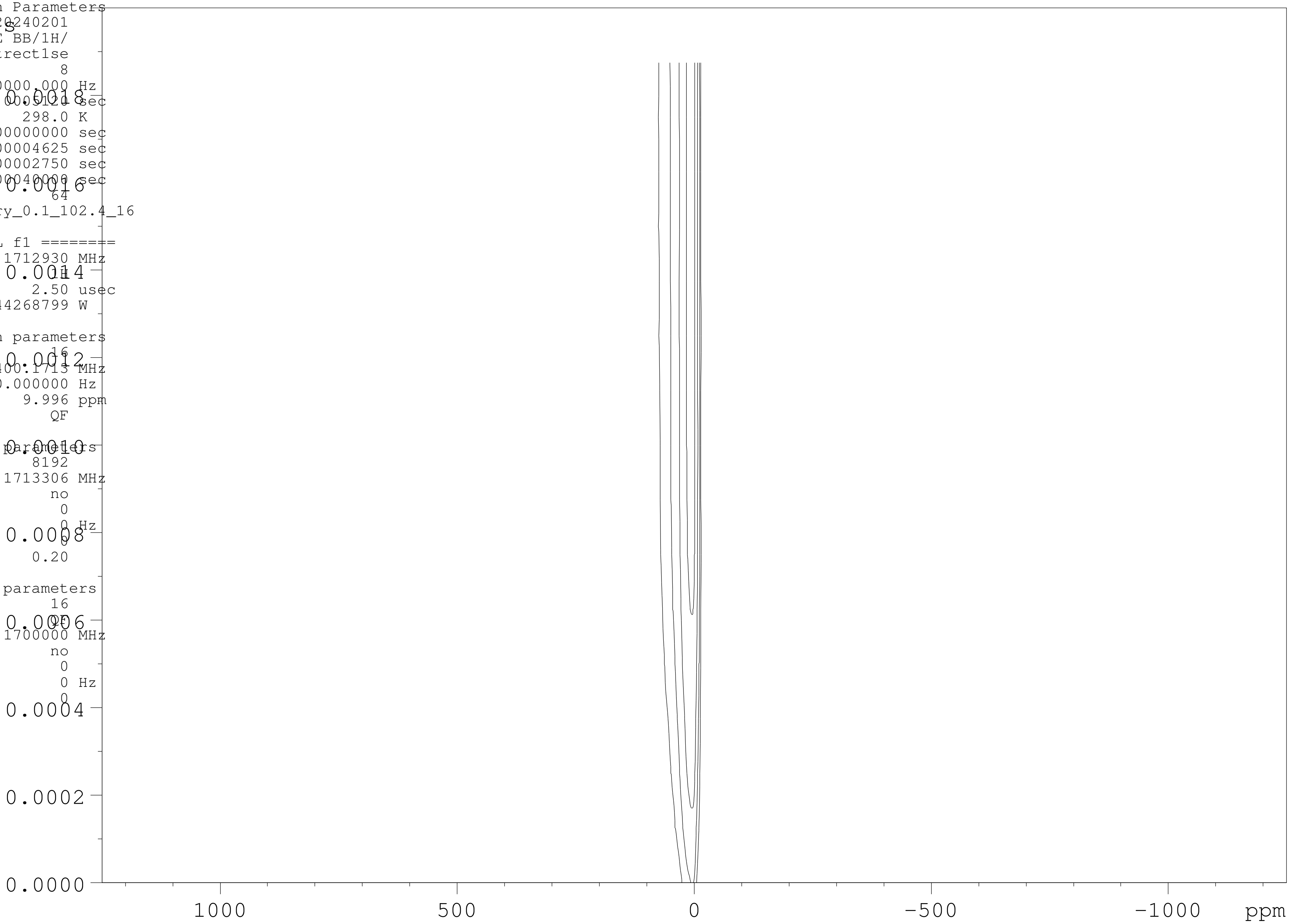

2624-10340 SYT0043a S-CTA CdCl4 @ static / -100 to +120 C
40 C

Current Data Parameters
NAME jse_20240130
EXPNO 74
PROCNO 1

F2 - Acquisition Parameters
Date_ 20240201
PROBHD 5 mm PE BB/1H/
PULPROG t1rho_solidecho
NS 8
SWH 1000000.000 Hz
AQ 0.0005120 sec
TE 314.1 K
D1 7.19999981 sec
D6 0.00005000 sec
D7 0.00002750 sec

======== CHANNEL f1 ========
SFO1 400.1712930 MHz
NUC1 1H
P1 2.50 usec
PLW1 378.44268799 W
PLW2 94.62400055 W
VPLIST 100u_52000u_16

F1 - Acquisition parameters
TD 8
SFO1 400.1713 MHz
FIDRES 1000.000000 Hz
SW 9.996 ppm
FnMODE QF

F2 - Processing parameters
SI 8192
SF 400.1713306 MHz
WDW no
SSB 0
LB 0 Hz
GB 0
PC 0.20

F1 - Processing parameters
SI 16
MC2 QF
SF 400.1700000 MHz
WDW no
SSB 0
LB 0 Hz
GB 0

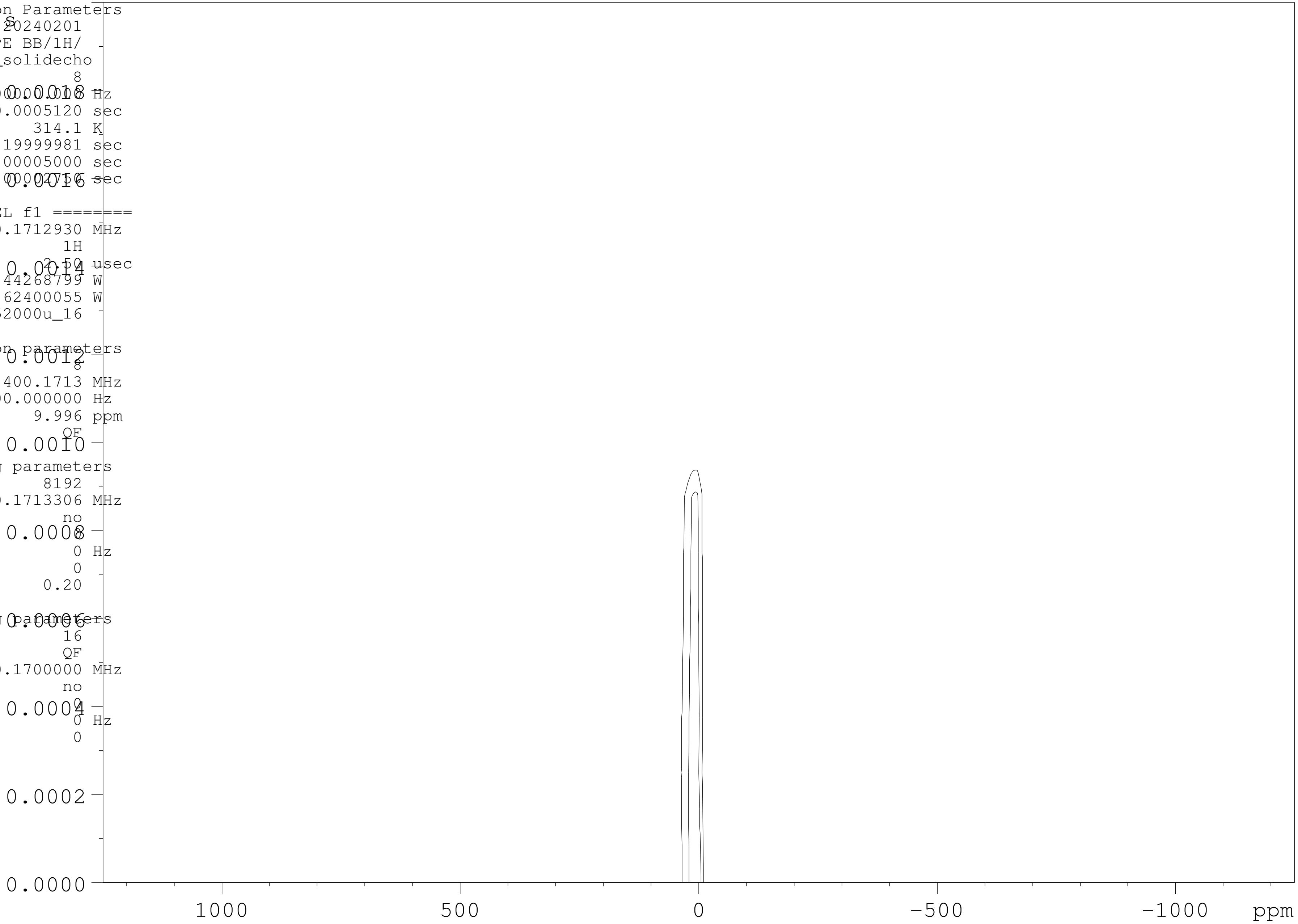

2624-10340 SYT0043a S-CTA CdCl4 @ static / -100 to +120 C
50 C

```
Current Data Parameters
NAME       jse_20240130
EXPNO                76
PROCNO                1

F2 - Acquisition Parameters
Date_          20240201
PROBHD    5 mm PE BB/1H/
PULPROG       solidecho
NS                    8
SWH         1000000.000 Hz
AQ            0.0005120 sec
TE                294.0 K
D1           7.19999981 sec
D6           0.00005000 sec
D7           0.00002750 sec

======== CHANNEL f1 ========
SFO1        400.1712930 MHz
NUC1                 1H
P1                 2.50 usec
PLW1       378.44268799 W

F2 - Processing parameters
SI                 8192
SF          400.1713306 MHz
WDW                  EM
SSB                   0
LB                10.00 Hz
GB                    0
PC                 0.20
```

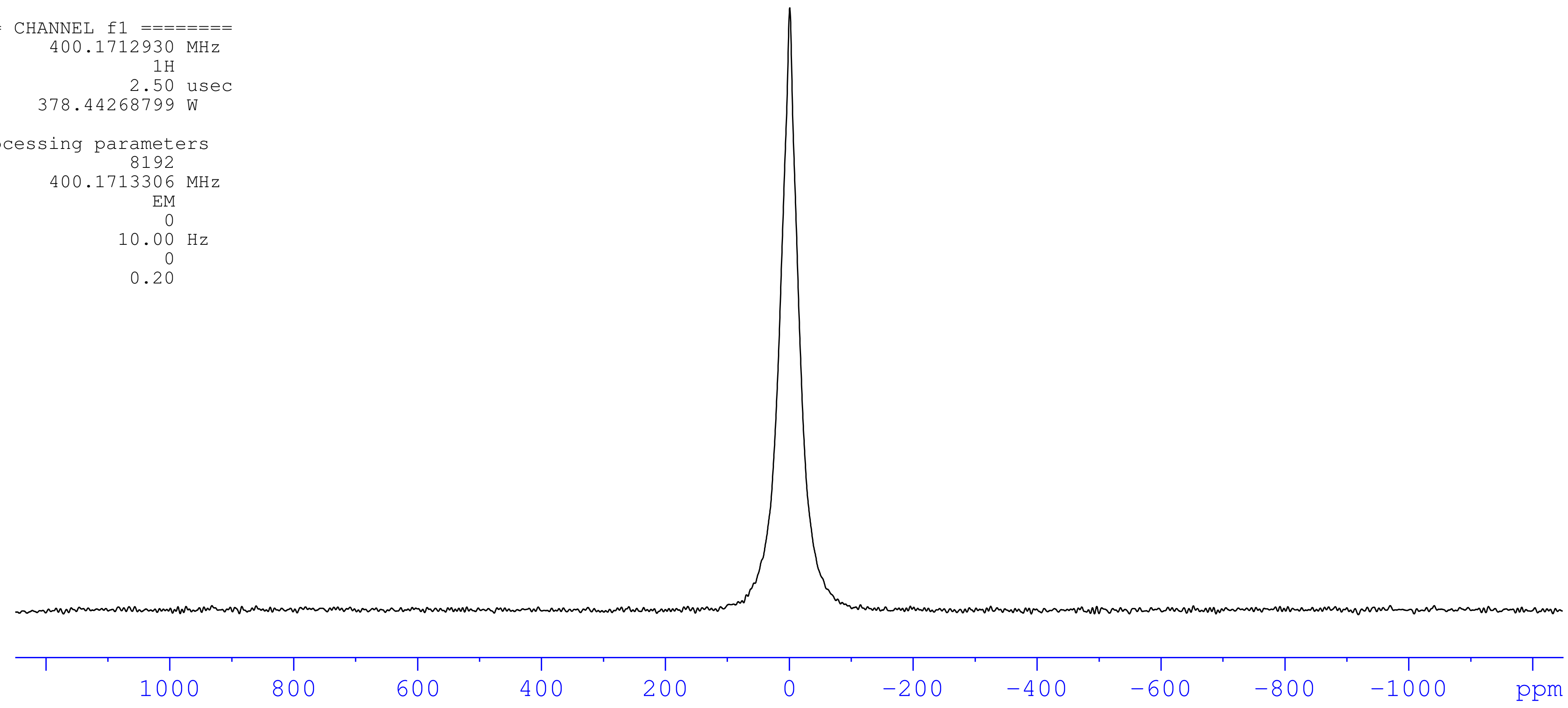

Current Data Parameters
NAME jse_20240130
EXPNO 77
PROCNO 1

F2 - Acquisition Parameters
Date_ 20240201
PROBHD 5 mm PE BB/1H/
PULPROG zg
NS 4
SWH 1000000.000 Hz
AQ 0.0005120 sec
TE 294.0 K
D1 7.19999981 sec
TD0 1

======== CHANNEL f1 ========
SFO1 400.1712930 MHz
NUC1 1H
P1 2.50 usec
PLW1 378.44268799 W

F2 - Processing parameters
SI 8192
SF 400.1713306 MHz
WDW EM
SSB 0
LB 10.00 Hz
GB 0
PC 0.20

2624-10340 SYT0043a S-CTA CdCl4 @ static / -100 to +120 C
50 C

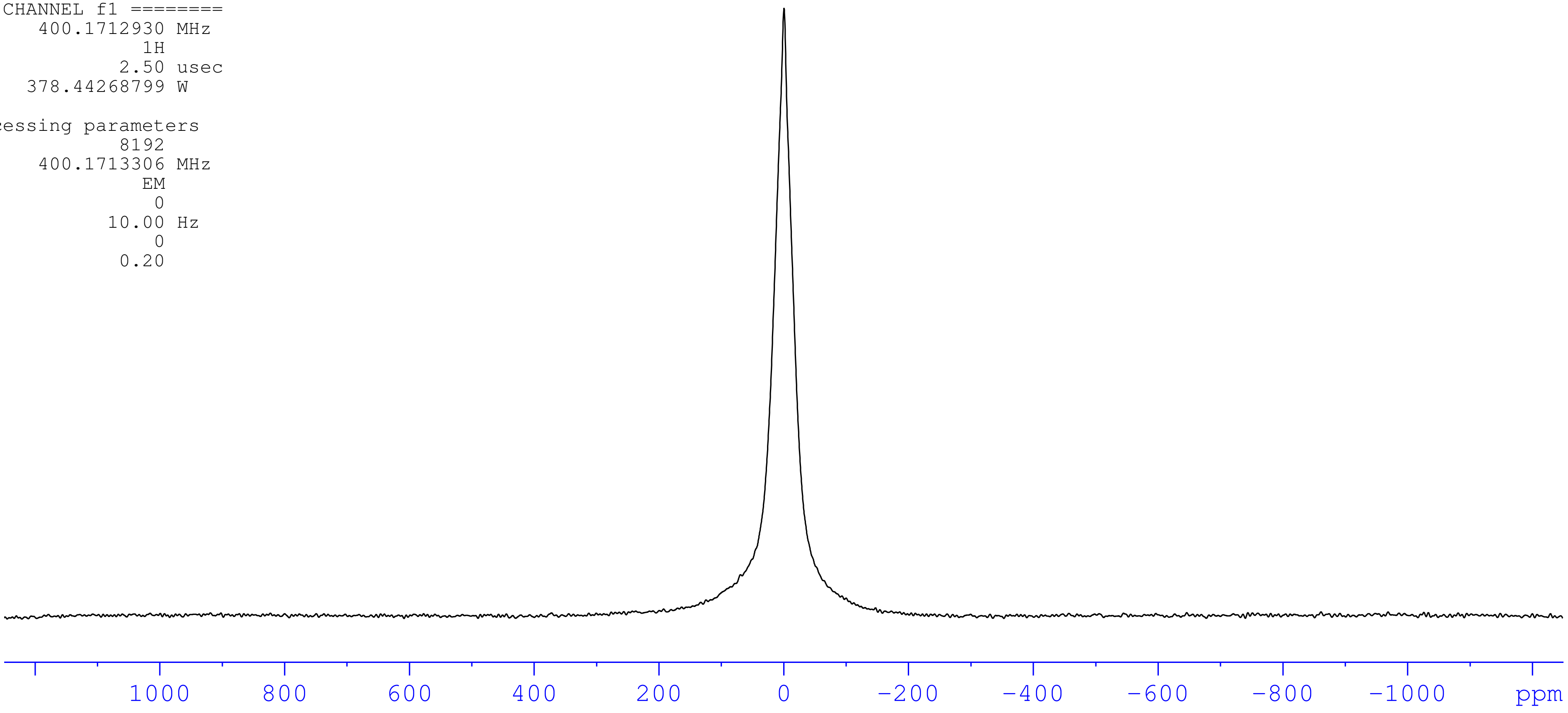

2624-10340 SYT0043a S-CTA CdCl4 @ static / -100 to +120 C
50 C

```
Current Data Parameters
NAME        jse_20240130
EXPNO                 78
PROCNO                 1

F2 - Acquisition Parameters
Date_           20240201
PROBHD   5 mm PE BB/1H/
PULPROG      satrect1se
NS                     8
SWH       1000000.000 Hz
AQ          0.0005120 sec
TE              298.0 K
D1         1.00000000 sec
D6         0.00004625 sec
D7         0.00002750 sec
D20        0.00040000 sec
L20                   64
VDLIST   Recovery_0.1_102.4_16

======== CHANNEL f1 ========
SFO1       400.1712930 MHz
NUC1                  1H
P1                  2.50 usec
PLW1        378.44268799 W

F1 - Acquisition parameters
TD                    16
SFO1           400.1713 MHz
FIDRES       500.000000 Hz
SW                 9.996 ppm
FnMODE                QF

F2 - Processing parameters
SI                  8192
SF         400.1713306 MHz
WDW                   no
SSB                    0
LB                     0 Hz
GB                     0
PC                  0.20

F1 - Processing parameters
SI                    16
MC2                   QF
SF         400.1700000 MHz
WDW                   no
SSB                    0
LB                     0 Hz
GB                     0
```

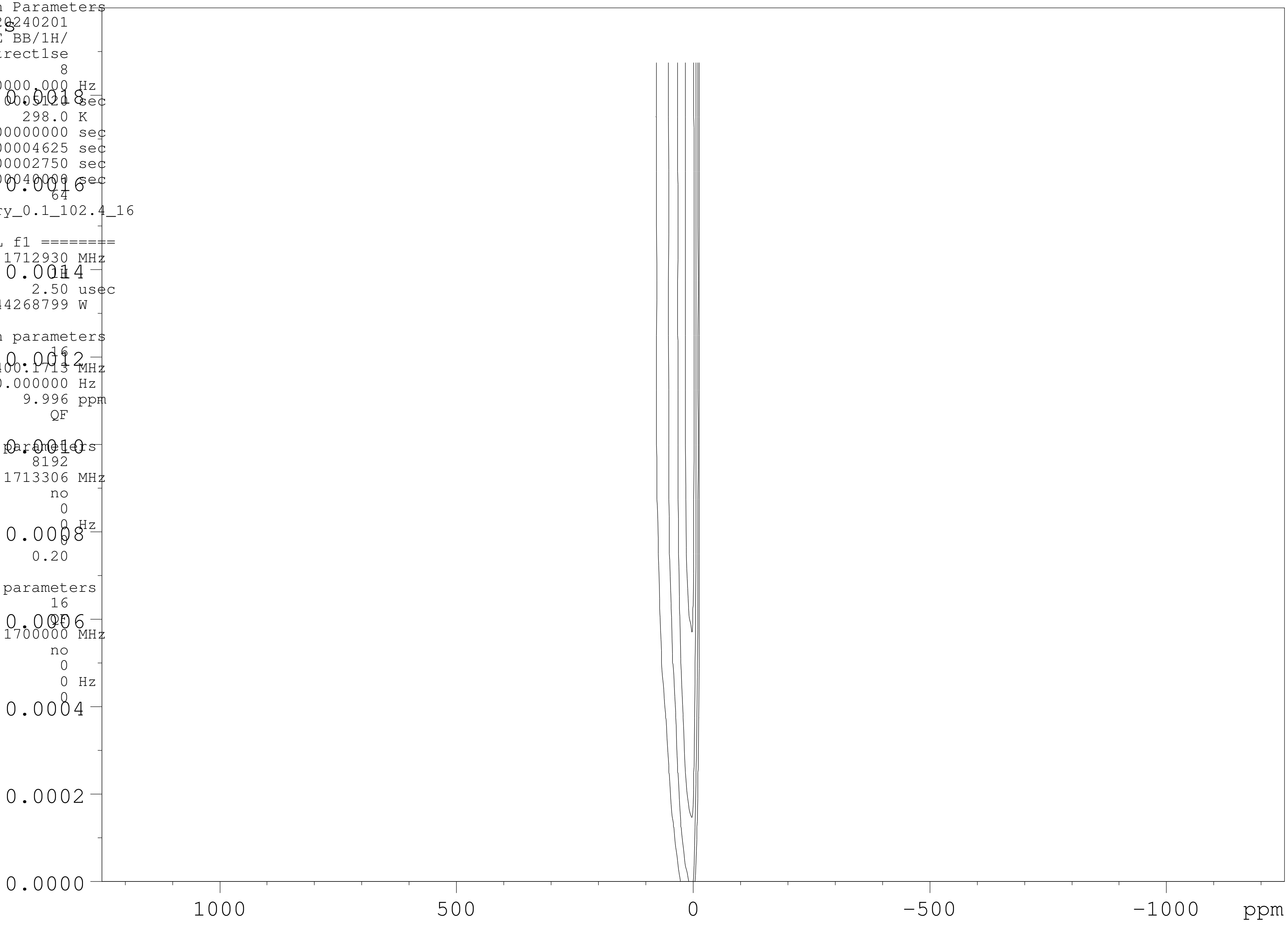

Current Data Parameters
NAME jse_20240130
EXPNO 79
PROCNO 1

F2 - Acquisition Parameters
Date_ 20240201
PROBHD 5 mm PE BB/1H/
PULPROG t1rho_solidecho
NS 8
SWH 1000000.000 Hz
AQ 0.0005120 sec
TE 320.8 K
D1 7.19999981 sec
D6 0.00005000 sec
D7 0.00002750 sec

======== CHANNEL f1 ========
SFO1 400.1712930 MHz
NUC1 1H
P1 2.50 usec
PLW1 378.44268799 W
PLW2 94.62400055 W
VPLIST 100u_52000u_16

F1 - Acquisition parameters
TD 8
SFO1 400.1713 MHz
FIDRES 1000.000000 Hz
SW 9.996 ppm
FnMODE QF

F2 - Processing parameters
SI 8192
SF 400.1713306 MHz
WDW no
SSB 0
LB 0 Hz
GB 0
PC 0.20

F1 - Processing parameters
SI 16
MC2 QF
SF 400.1700000 MHz
WDW no
SSB 0
LB 0 Hz
GB 0

2624-10340 SYT0043a S-CTA CdCl4 @ static / -100 to +120 C
50 C

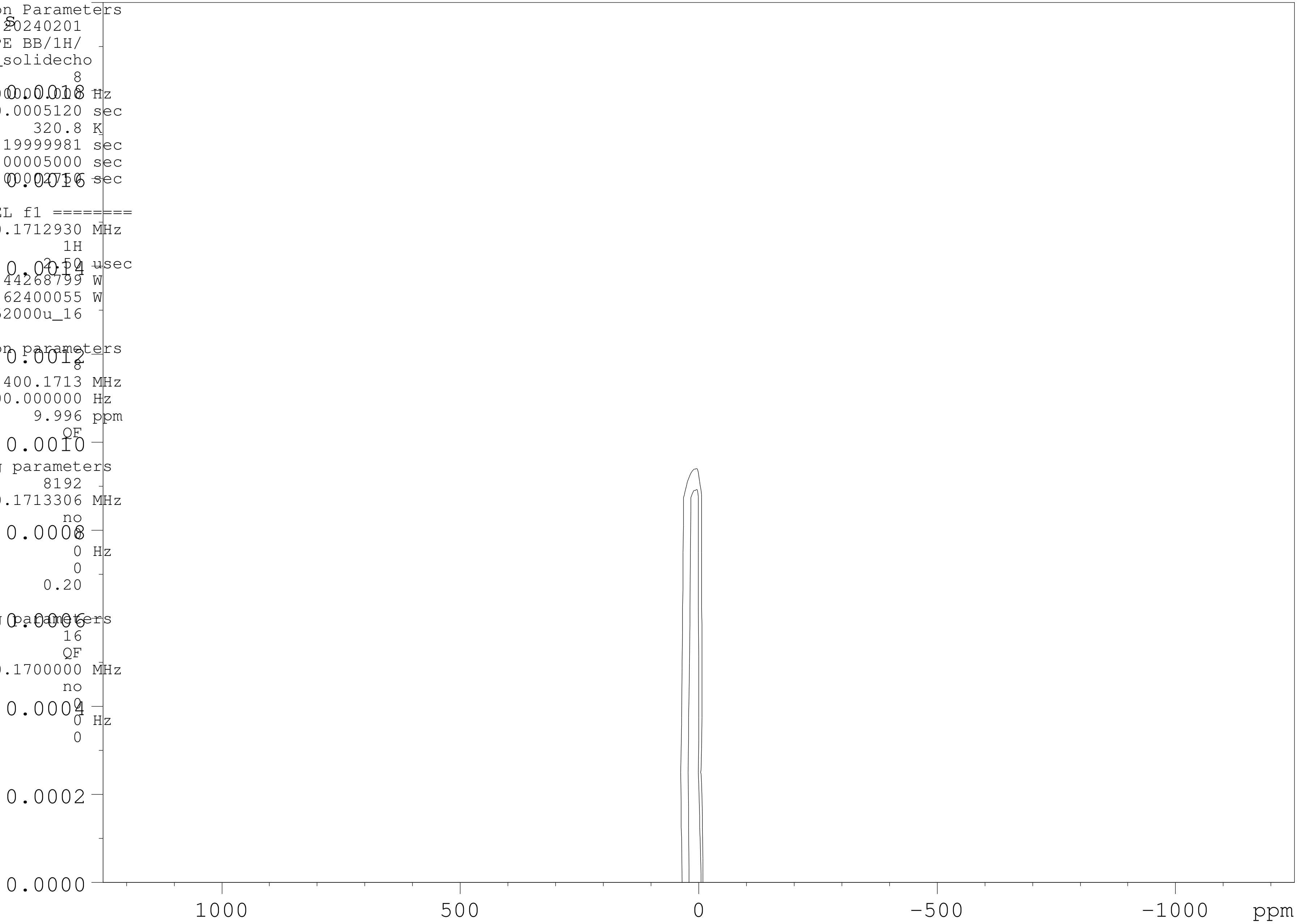

Current Data Parameters
NAME jse_20240130
EXPNO 81
PROCNO 1

F2 - Acquisition Parameters
Date_ 20240201
PROBHD 5 mm PE BB/1H/
PULPROG solidecho
NS 8
SWH 1000000.000 Hz
AQ 0.0005120 sec
TE 294.0 K
D1 7.19999981 sec
D6 0.00005000 sec
D7 0.00002750 sec

======== CHANNEL f1 ========
SFO1 400.1712930 MHz
NUC1 1H
P1 2.50 usec
PLW1 378.44268799 W

F2 - Processing parameters
SI 8192
SF 400.1713306 MHz
WDW EM
SSB 0
LB 10.00 Hz
GB 0
PC 0.20

2624-10340 SYT0043a S-CTA CdCl4 @ static / -100 to +120 C
60 C

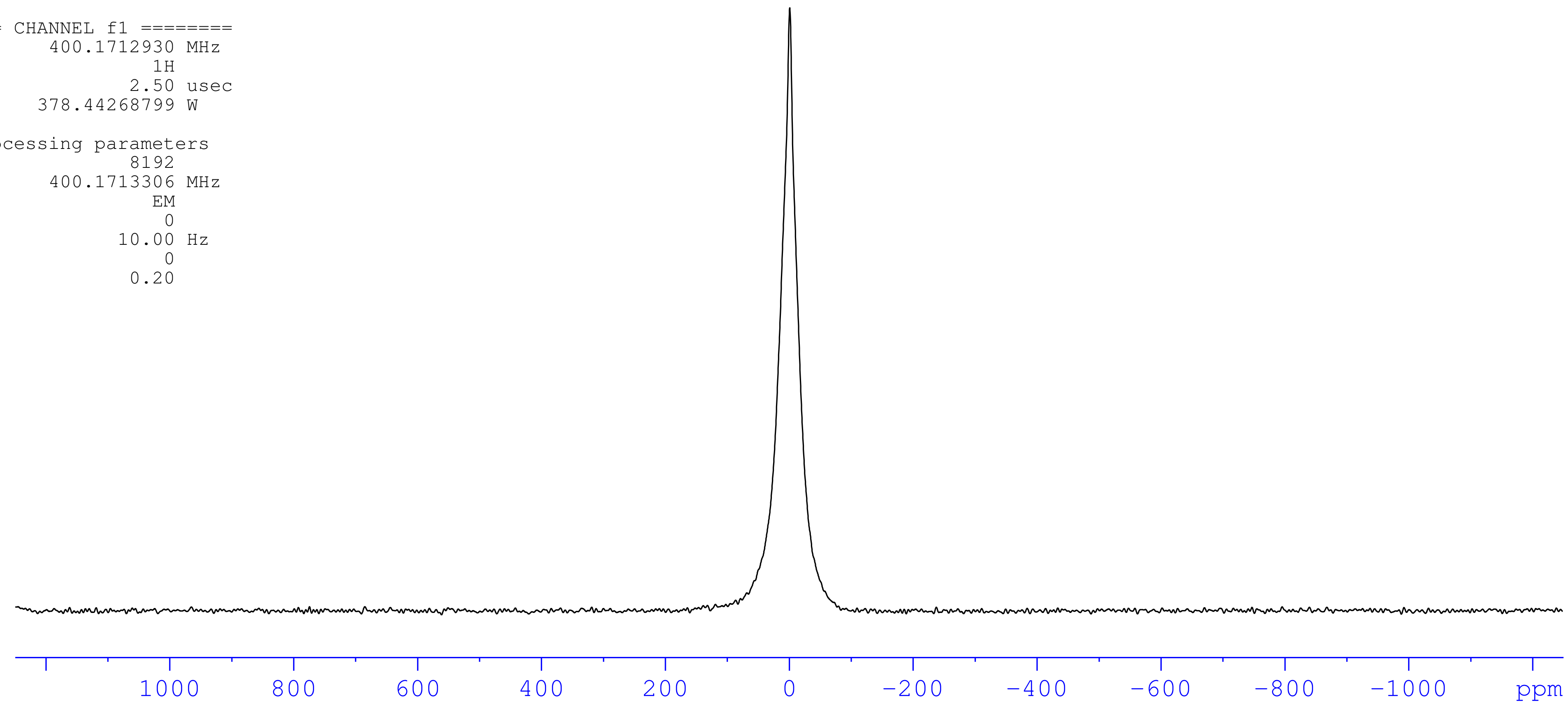

```
Current Data Parameters
NAME         jse_20240130
EXPNO                  82
PROCNO                  1

F2 - Acquisition Parameters
Date_           20240201
PROBHD    5 mm PE BB/1H/
PULPROG               zg
NS                     4
SWH          1000000.000 Hz
AQ             0.0005120 sec
TE                 294.0 K
D1            7.19999981 sec
TD0                    1

======== CHANNEL f1 ========
SFO1         400.1712930 MHz
NUC1                  1H
P1                  2.50 usec
PLW1        378.44268799 W

F2 - Processing parameters
SI                  8192
SF           400.1713306 MHz
WDW                   EM
SSB                    0
LB                 10.00 Hz
GB                     0
PC                  0.20
```

2624-10340 SYT0043a S-CTA CdCl4 @ static / -100 to +120 C
60 C

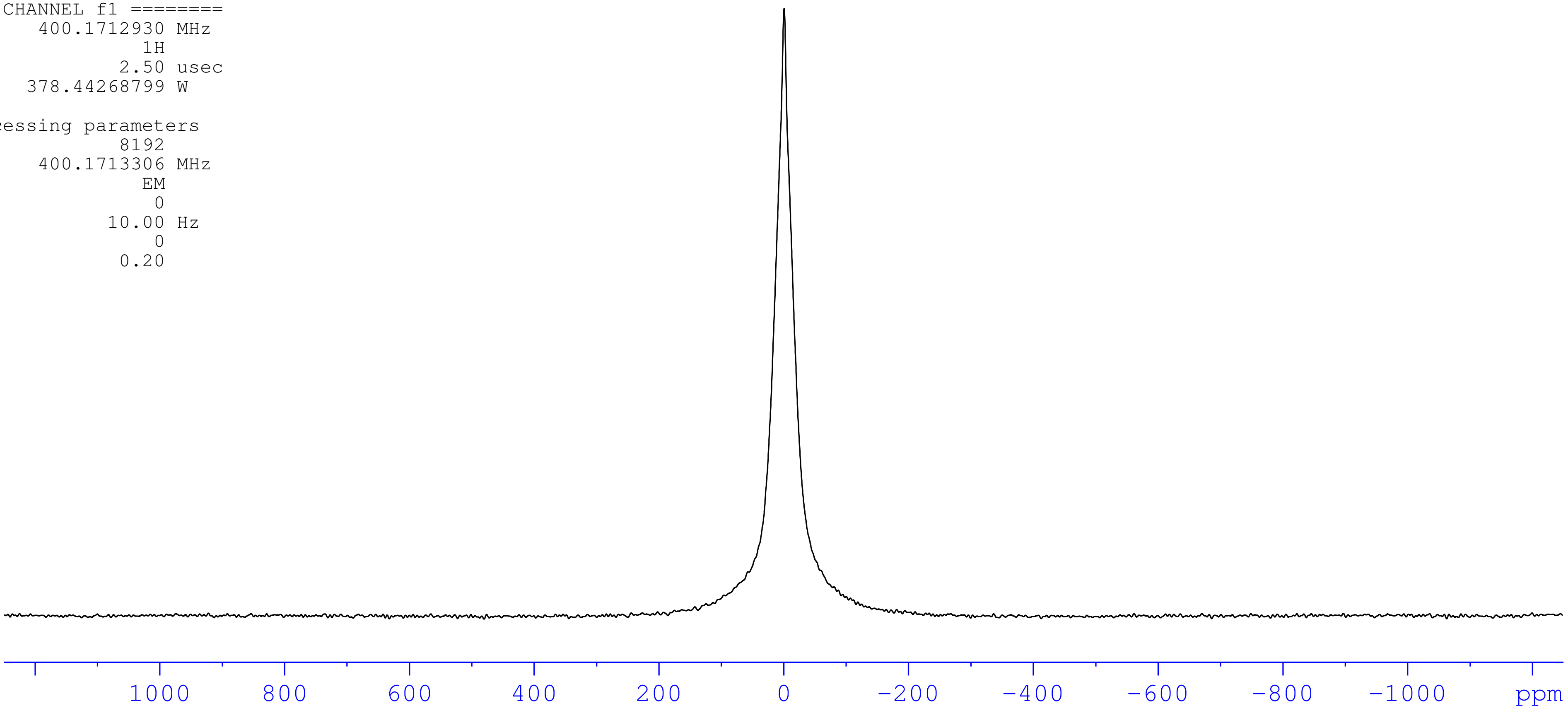

Current Data Parameters
NAME jse_20240130
EXPNO 83
PROCNO 1

F2 - Acquisition Parameters
Date_ 20240201
PROBHD 5 mm PE BB/1H/
PULPROG satrect1se
NS 8
SWH 1000000.000 Hz
AQ 0.0005120 sec
TE 298.0 K
D1 1.00000000 sec
D6 0.00004625 sec
D7 0.00002750 sec
D20 0.00040000 sec
L20 64
VDLIST Recovery_0.1_102.4_16

======== CHANNEL f1 ========
SFO1 400.1712930 MHz
NUC1 [illegible]
P1 2.50 usec
PLW1 378.44268799 W

F1 - Acquisition parameters
TD 16
SFO1 400.1713 MHz
FIDRES 500.000000 Hz
SW 9.996 ppm
FnMODE QF

F2 - Processing parameters
SI 8192
SF 400.1713306 MHz
WDW no
SSB 0
LB 0 Hz
GB 0
PC 0.20

F1 - Processing parameters
SI 16
MC2 [illegible]
SF 400.1700000 MHz
WDW no
SSB 0
LB 0 Hz
GB 0

2624-10340 SYT0043a S-CTA CdCl4 @ static / -100 to +120 C
60 C

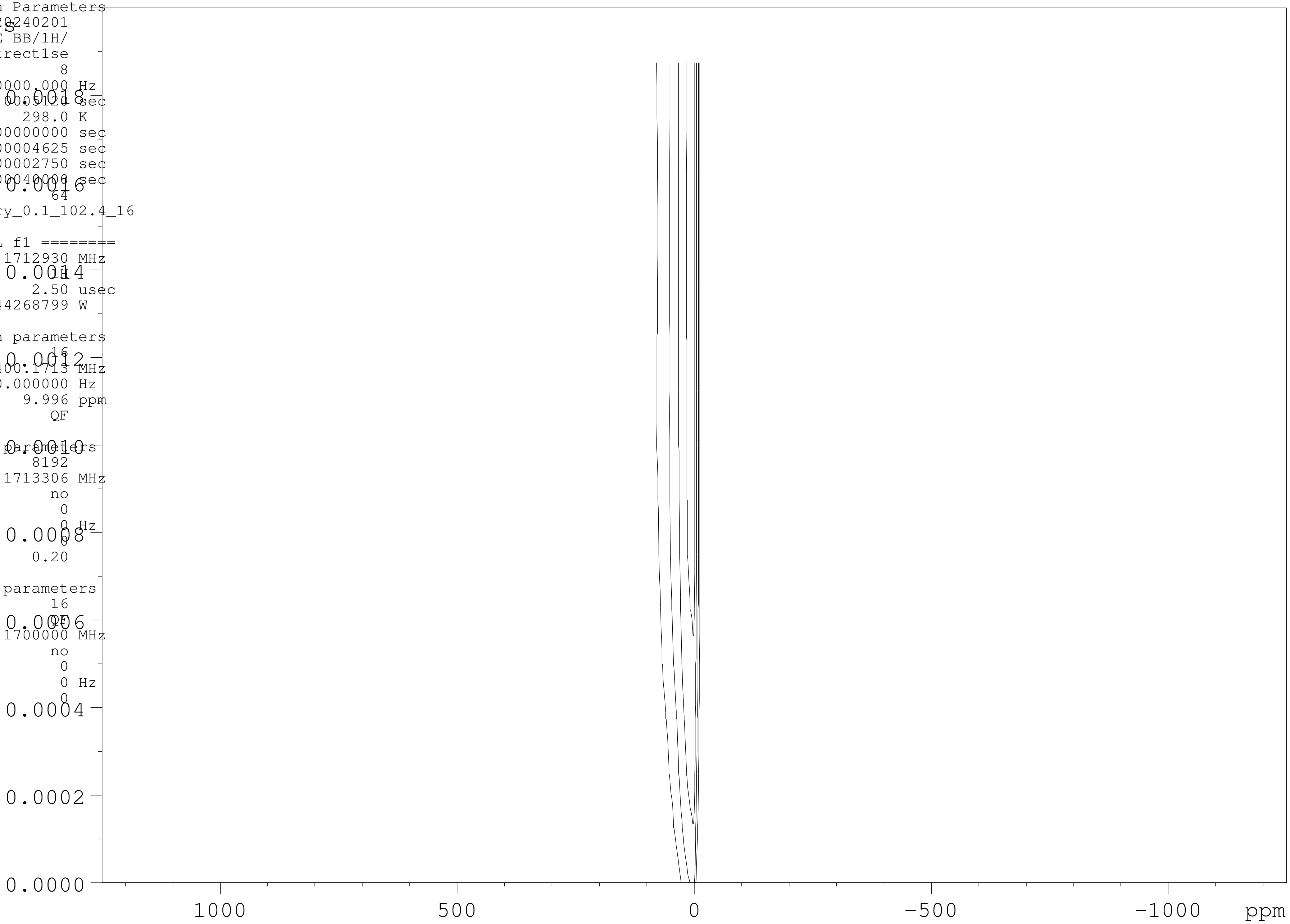

2624-10340 SYT0043a S-CTA CdCl4 @ static / -100 to +120 C
60 C

```
Current Data Parameters
NAME       jse_20240130
EXPNO                84
PROCNO                1

F2 - Acquisition Parameters
Date_          20240201
PROBHD    5 mm PE BB/1H/
PULPROG  t1rho_solidecho
NS                    8
SWH         1000000.000 Hz
AQ            0.0005120 sec
TE                327.5 K
D1           7.19999981 sec
D6           0.00005000 sec
D7           0.00002750 sec

======== CHANNEL f1 ========
SFO1        400.1712930 MHz
NUC1                 1H
P1                 2.50 usec
PLW1       378.44268799 W
PLW2        94.62400055 W
VPLIST    100u_52000u_16

F1 - Acquisition parameters
TD                    8
SFO1           400.1713 MHz
FIDRES      1000.000000 Hz
SW                9.996 ppm
FnMODE               QF

F2 - Processing parameters
SI                 8192
SF          400.1713306 MHz
WDW                  no
SSB                   0
LB                    0 Hz
GB                    0
PC                 0.20

F1 - Processing parameters
SI                   16
MC2                  QF
SF          400.1700000 MHz
WDW                  no
SSB                   0
LB                    0 Hz
GB                    0
```

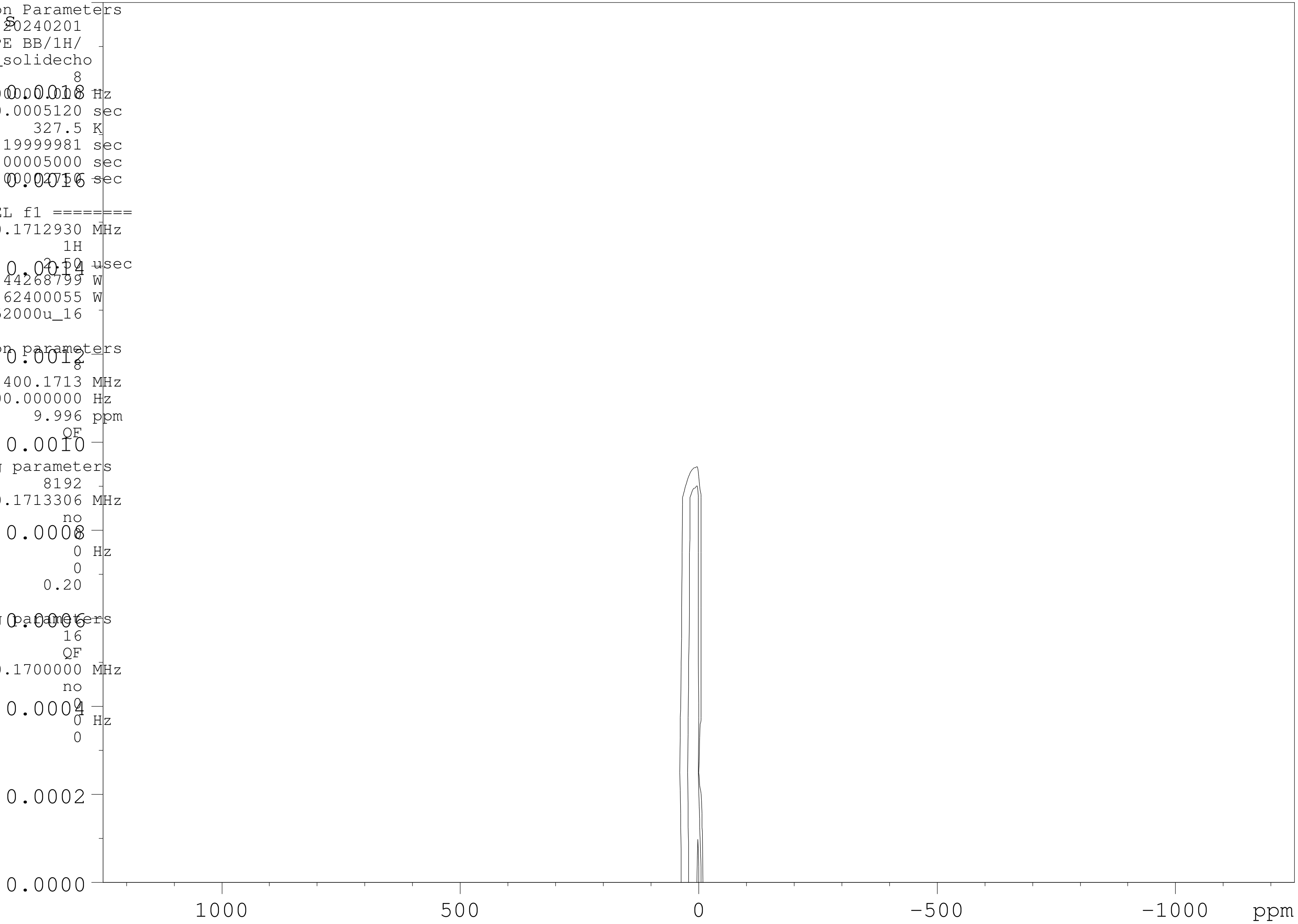

```
Current Data Parameters
NAME       jse_20240130
EXPNO                86
PROCNO                1

F2 - Acquisition Parameters
Date_          20240201
PROBHD   5 mm PE BB/1H/
PULPROG       solidecho
NS                    8
SWH         1000000.000 Hz
AQ            0.0005120 sec
TE                294.0 K
D1           7.19999981 sec
D6           0.00005000 sec
D7           0.00002750 sec

======== CHANNEL f1 ========
SFO1        400.1712930 MHz
NUC1                 1H
P1                 2.50 usec
PLW1       378.44268799 W

F2 - Processing parameters
SI                 8192
SF          400.1713306 MHz
WDW                  EM
SSB                   0
LB                10.00 Hz
GB                    0
PC                 0.20
```

2624-10340 SYT0043a S-CTA CdCl4 @ static / -100 to +120 C
70 C

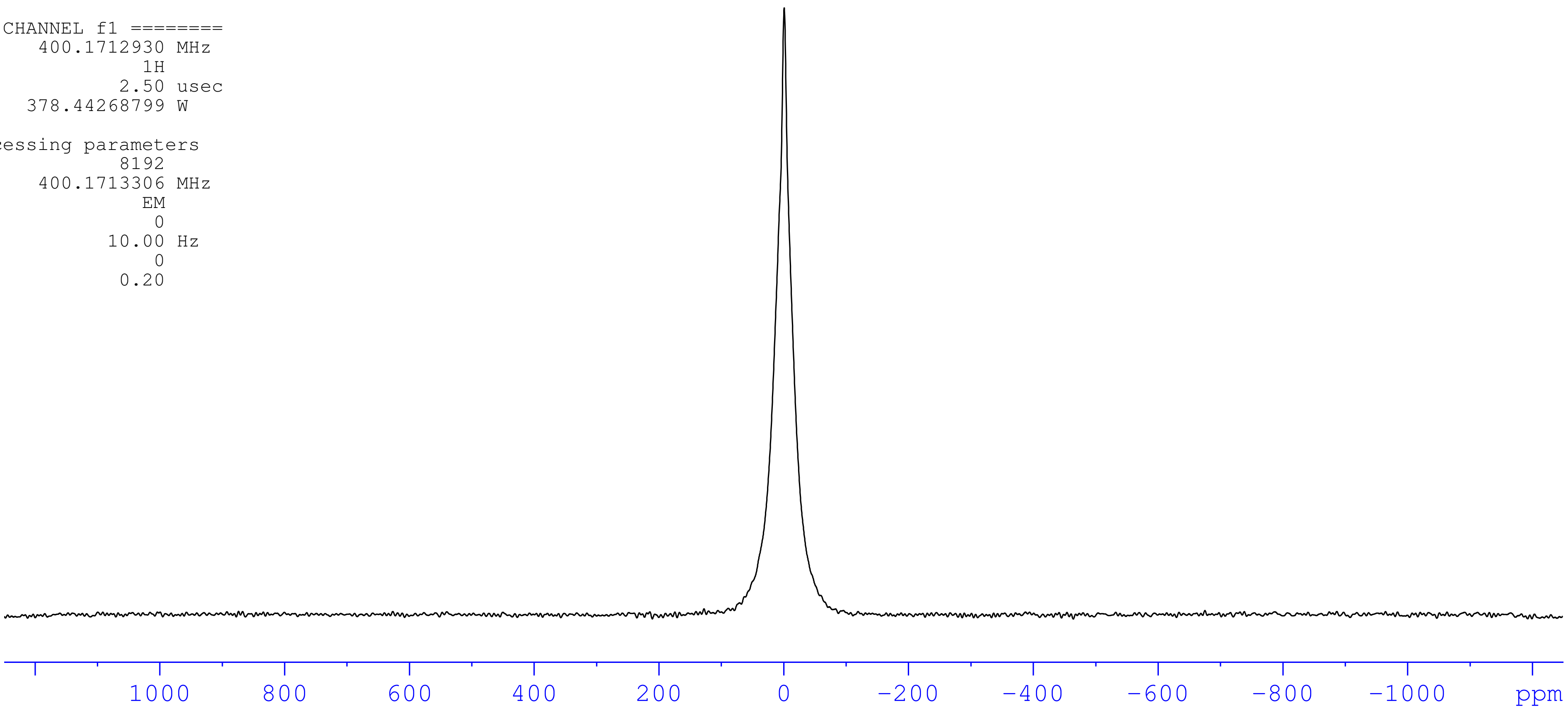

```
Current Data Parameters
NAME       jse_20240130
EXPNO                87
PROCNO                1

F2 - Acquisition Parameters
Date_          20240201
PROBHD    5 mm PE BB/1H/
PULPROG              zg
NS                    4
SWH         1000000.000 Hz
AQ            0.0005120 sec
TE                294.0 K
D1           7.19999981 sec
TD0                   1

======== CHANNEL f1 ========
SFO1        400.1712930 MHz
NUC1                 1H
P1                 2.50 usec
PLW1       378.44268799 W

F2 - Processing parameters
SI                 8192
SF          400.1713306 MHz
WDW                  EM
SSB                   0
LB                10.00 Hz
GB                    0
PC                 0.20
```

2624-10340 SYT0043a S-CTA CdCl4 @ static / -100 to +120 C
70 C

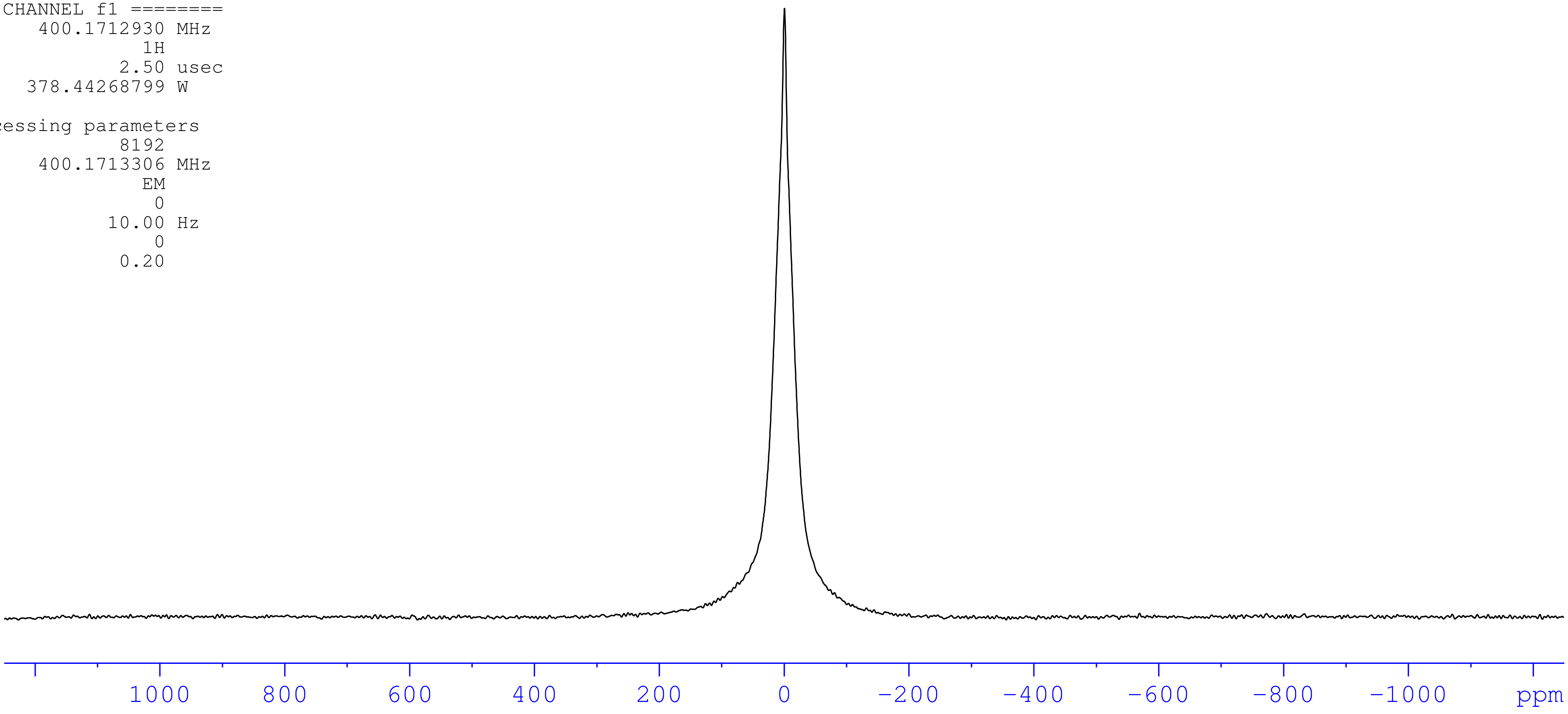

```
Current Data Parameters
NAME       jse_20240130
EXPNO                88
PROCNO                1

F2 - Acquisition Parameters
Date_          20240201
PROBHD   5 mm PE BB/1H/
PULPROG      satrect1se
NS                    8
SWH      1000000.000 Hz
AQ        0.0005120 sec
TE              298.0 K
D1       1.00000000 sec
D6       0.00004625 sec
D7       0.00002750 sec
D20      0.00040000 sec
L20                  64
VDLIST   Recovery_0.1_102.4_16

======== CHANNEL f1 ========
SFO1     400.1712930 MHz
NUC1                 1H
P1             2.50 usec
PLW1     378.44268799 W

F1 - Acquisition parameters
TD                   16
SFO1        400.1713 MHz
FIDRES   500.000000 Hz
SW            9.996 ppm
FnMODE               QF

F2 - Processing parameters
SI                 8192
SF       400.1713306 MHz
WDW                  no
SSB                   0
LB                 0 Hz
GB                    0
PC                 0.20

F1 - Processing parameters
SI                   16
MC2                  QF
SF       400.1700000 MHz
WDW                  no
SSB                   0
LB                 0 Hz
GB                    0
```

2624-10340 SYT0043a S-CTA CdCl4 @ static / -100 to +120 C
70 C

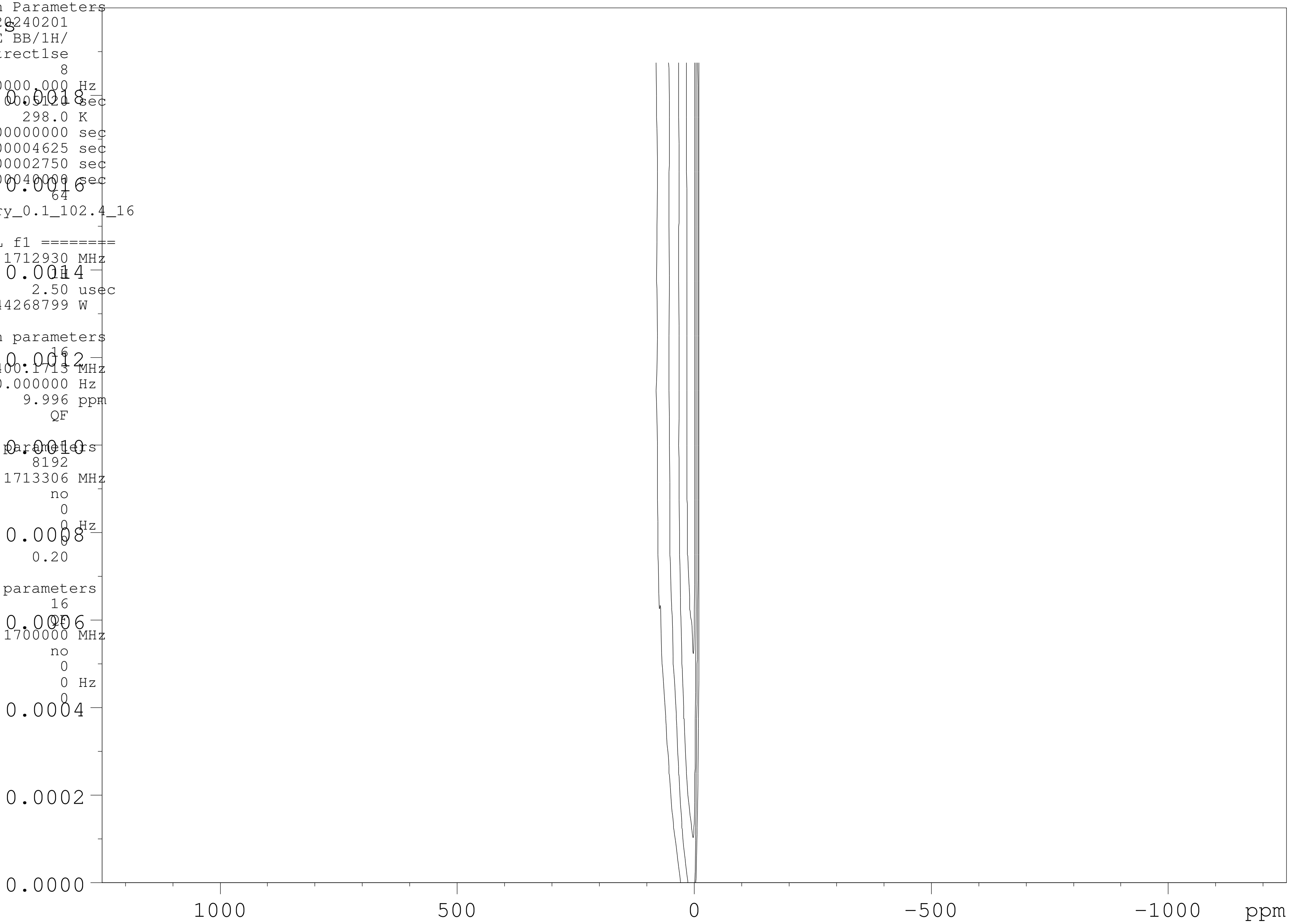

Current Data Parameters
NAME jse_20240130
EXPNO 89
PROCNO 1

2624-10340 SYT0043a S-CTA CdCl4 @ static / -100 to +120 C
70 C

F2 - Acquisition Parameters
Date_ 20240201
PROBHD 5 mm PE BB/1H/
PULPROG t1rho_solidecho
NS 8
SWH 1000000.000 Hz
AQ 0.0005120 sec
TE 300.7 K
D1 7.19999981 sec
D6 0.00005000 sec
D7 0.0000275[illegible] sec

======== CHANNEL f1 ========
SFO1 400.1712930 MHz
NUC1 1H
P1 2.50 usec
PLW1 378.44268799 W
PLW2 94.62400055 W
VPLIST 100u_52000u_16

F1 - Acquisition parameters
TD 8
SFO1 400.1713 MHz
FIDRES 1000.000000 Hz
SW 9.996 ppm
FnMODE QF

F2 - Processing parameters
SI 8192
SF 400.1713306 MHz
WDW no
SSB 0
LB 0 Hz
GB 0
PC 0.20

F1 - Processing parameters
SI 16
MC2 QF
SF 400.1700000 MHz
WDW no
SSB 0
LB 0 Hz
GB 0

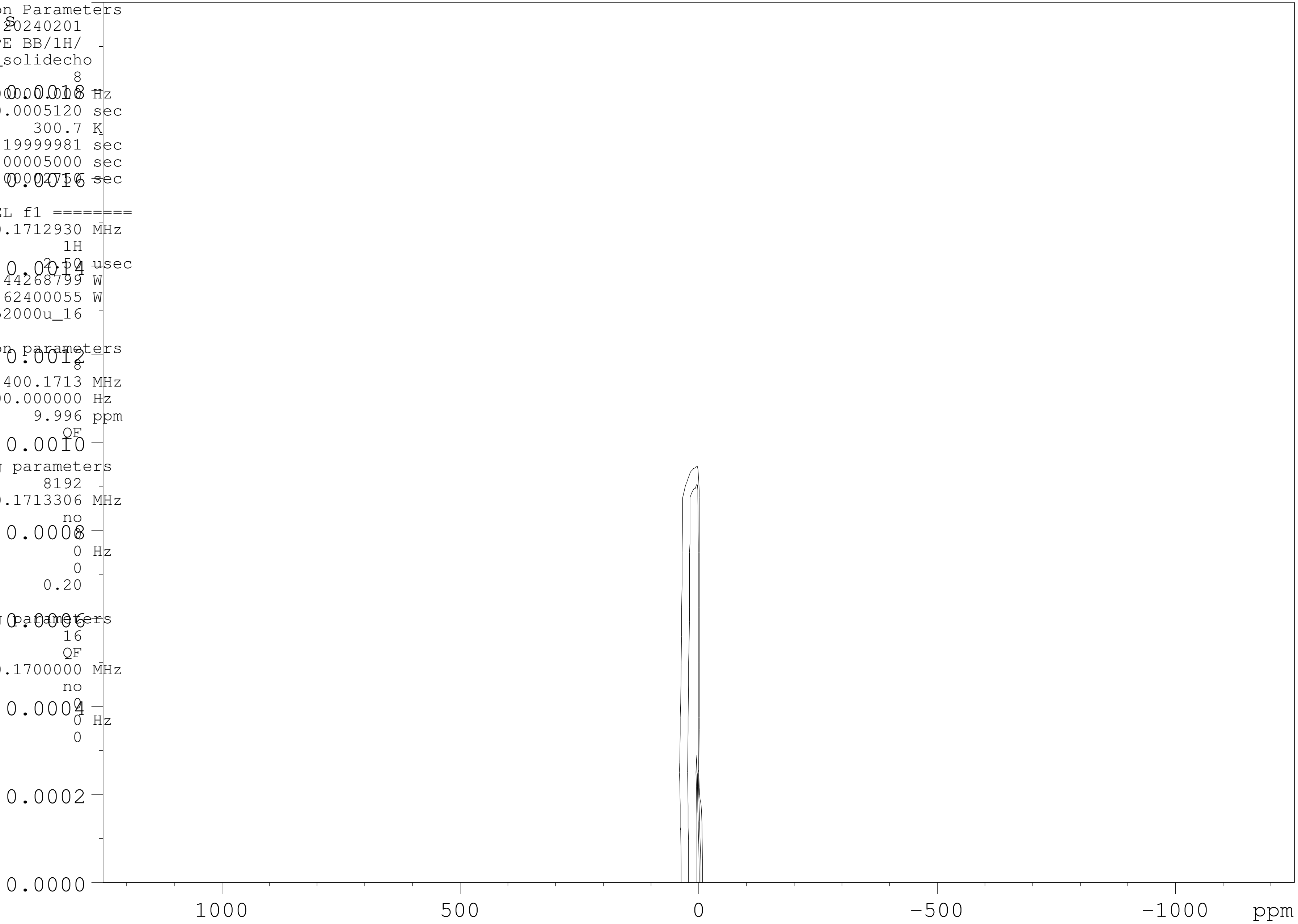

Current Data Parameters
NAME jse_20240130
EXPNO 91
PROCNO 1

F2 - Acquisition Parameters
Date_ 20240202
PROBHD 5 mm PE BB/1H/
PULPROG solidecho
NS 8
SWH 1000000.000 Hz
AQ 0.0005120 sec
TE 334.2 K
D1 7.19999981 sec
D6 0.00005000 sec
D7 0.00002750 sec

======== CHANNEL f1 ========
SFO1 400.1712930 MHz
NUC1 1H
P1 2.50 usec
PLW1 378.44268799 W

F2 - Processing parameters
SI 8192
SF 400.1713306 MHz
WDW EM
SSB 0
LB 10.00 Hz
GB 0
PC 0.20

2624-10340 SYT0043a S-CTA CdCl4 @ static / -100 to +120 C
80 C

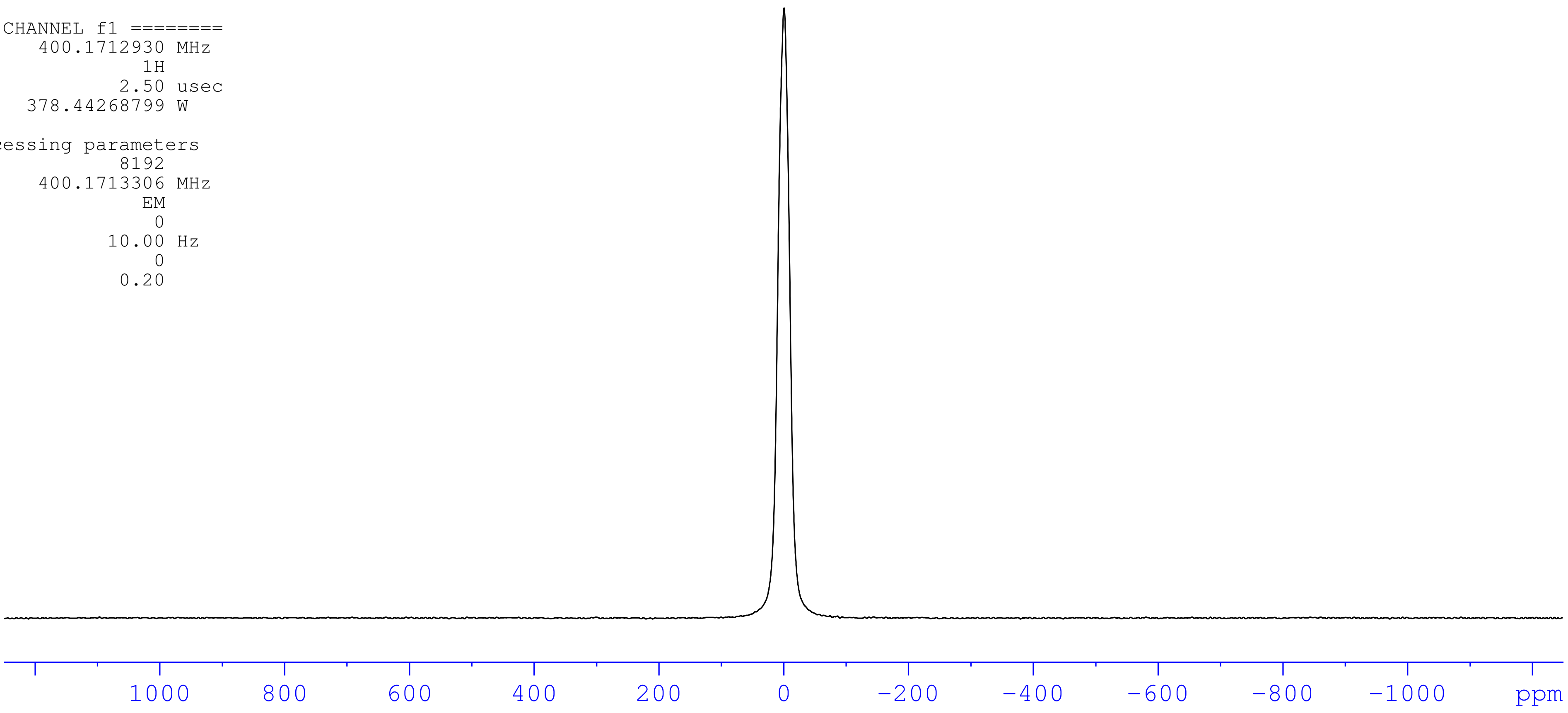

Current Data Parameters
NAME jse_20240130
EXPNO 92
PROCNO 1

F2 - Acquisition Parameters
Date_ 20240202
PROBHD 5 mm PE BB/1H/
PULPROG zg
NS 4
SWH 1000000.000 Hz
AQ 0.0005120 sec
TE 294.0 K
D1 7.19999981 sec
TD0 1

======== CHANNEL f1 ========
SFO1 400.1712930 MHz
NUC1 1H
P1 2.50 usec
PLW1 378.44268799 W

F2 - Processing parameters
SI 8192
SF 400.1713306 MHz
WDW EM
SSB 0
LB 10.00 Hz
GB 0
PC 0.20

2624-10340 SYT0043a S-CTA CdCl4 @ static / -100 to +120 C
80 C

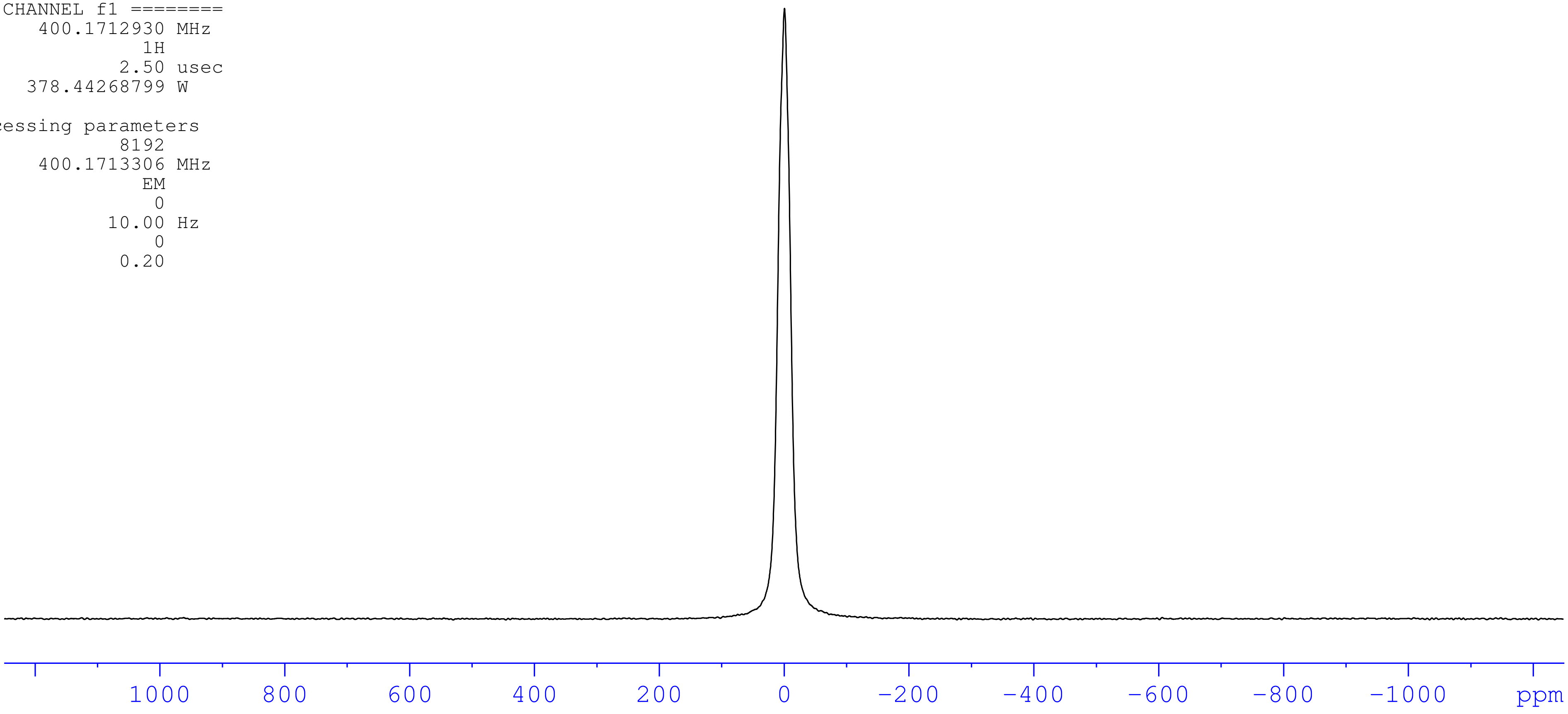

Current Data Parameters
NAME jse_20240130
EXPNO 93
PROCNO 1

F2 - Acquisition Parameters
Date_ 20240202
PROBHD 5 mm PE BB/1H/
PULPROG satrect1se
NS 8
SWH 1000000.000 Hz
AQ 0.0005120 sec
TE 298.0 K
D1 1.00000000 sec
D6 0.00004625 sec
D7 0.00002750 sec
D20 0.00040000 sec
L20 64
VDLIST Recovery_0.1_102.4_16

======== CHANNEL f1 ========
SFO1 400.1712930 MHz
NUC1 1H
P1 2.50 usec
PLW1 378.44268799 W

F1 - Acquisition parameters
TD 16
SFO1 400.1713 MHz
FIDRES 500.000000 Hz
SW 9.996 ppm
FnMODE QF

F2 - Processing parameters
SI 8192
SF 400.1713306 MHz
WDW no
SSB 0
LB 0 Hz
GB 0
PC 0.20

F1 - Processing parameters
SI 16
MC2 QF
SF 400.1700000 MHz
WDW no
SSB 0
LB 0 Hz
GB 0

2624-10340 SYT0043a S-CTA CdCl4 @ static / -100 to +120 C
80 C

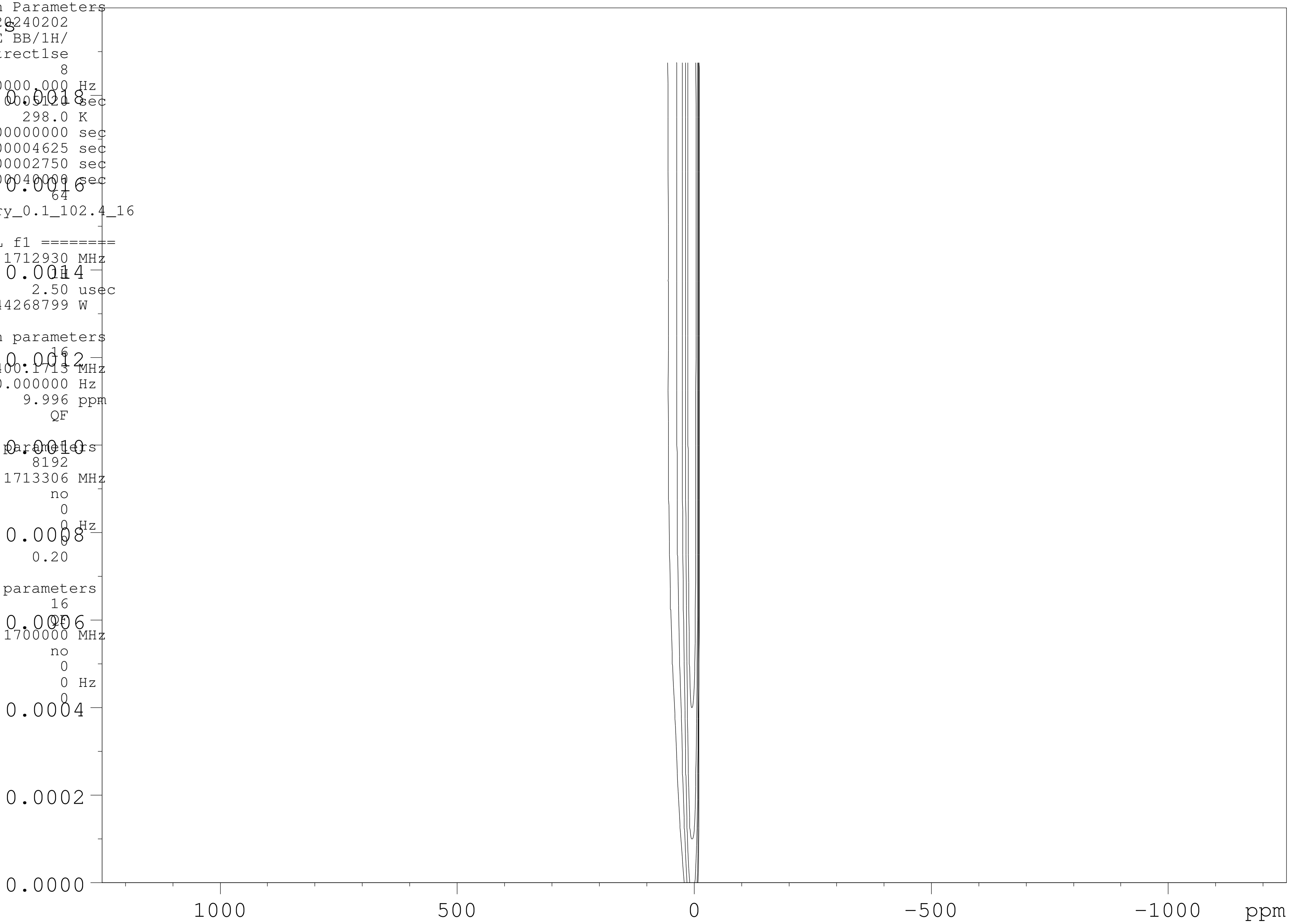

No parameters

2624-10340 SYT0043a S-CTA CdCl4 @ static / -100 to +120 C
80 C

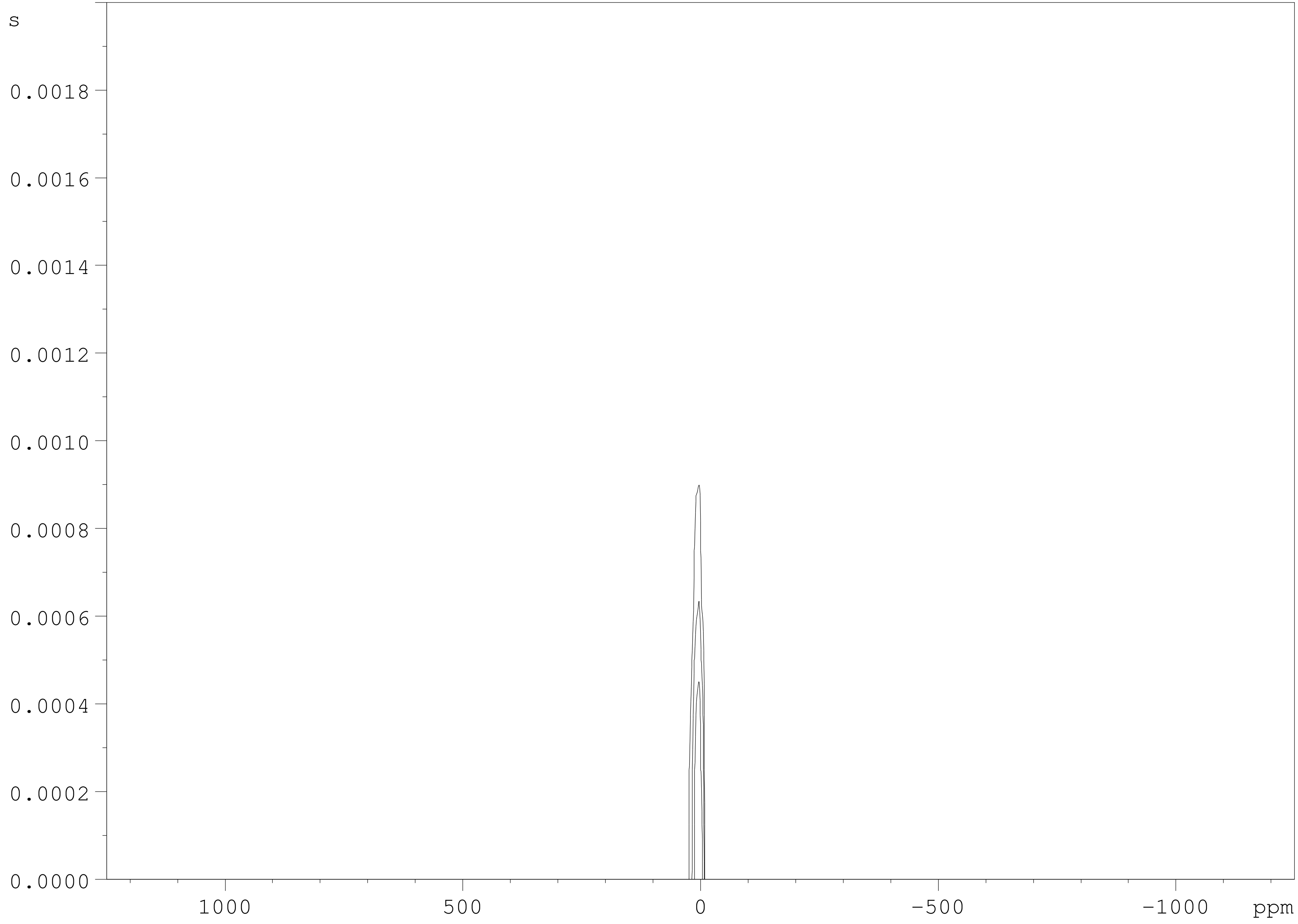

Current Data Parameters
NAME jse_20240130
EXPNO 96
PROCNO 1

F2 - Acquisition Parameters
Date_ 20240202
PROBHD 5 mm PE BB/1H/
PULPROG solidecho
NS 8
SWH 1000000.000 Hz
AQ 0.0005120 sec
TE 334.2 K
D1 7.19999981 sec
D6 0.00005000 sec
D7 0.00002750 sec

======== CHANNEL f1 ========
SFO1 400.1712930 MHz
NUC1 1H
P1 2.50 usec
PLW1 378.44268799 W

F2 - Processing parameters
SI 8192
SF 400.1713306 MHz
WDW EM
SSB 0
LB 10.00 Hz
GB 0
PC 0.20

2624-10340 SYT0043a S-CTA CdCl4 @ static / -100 to +120 C
90 C

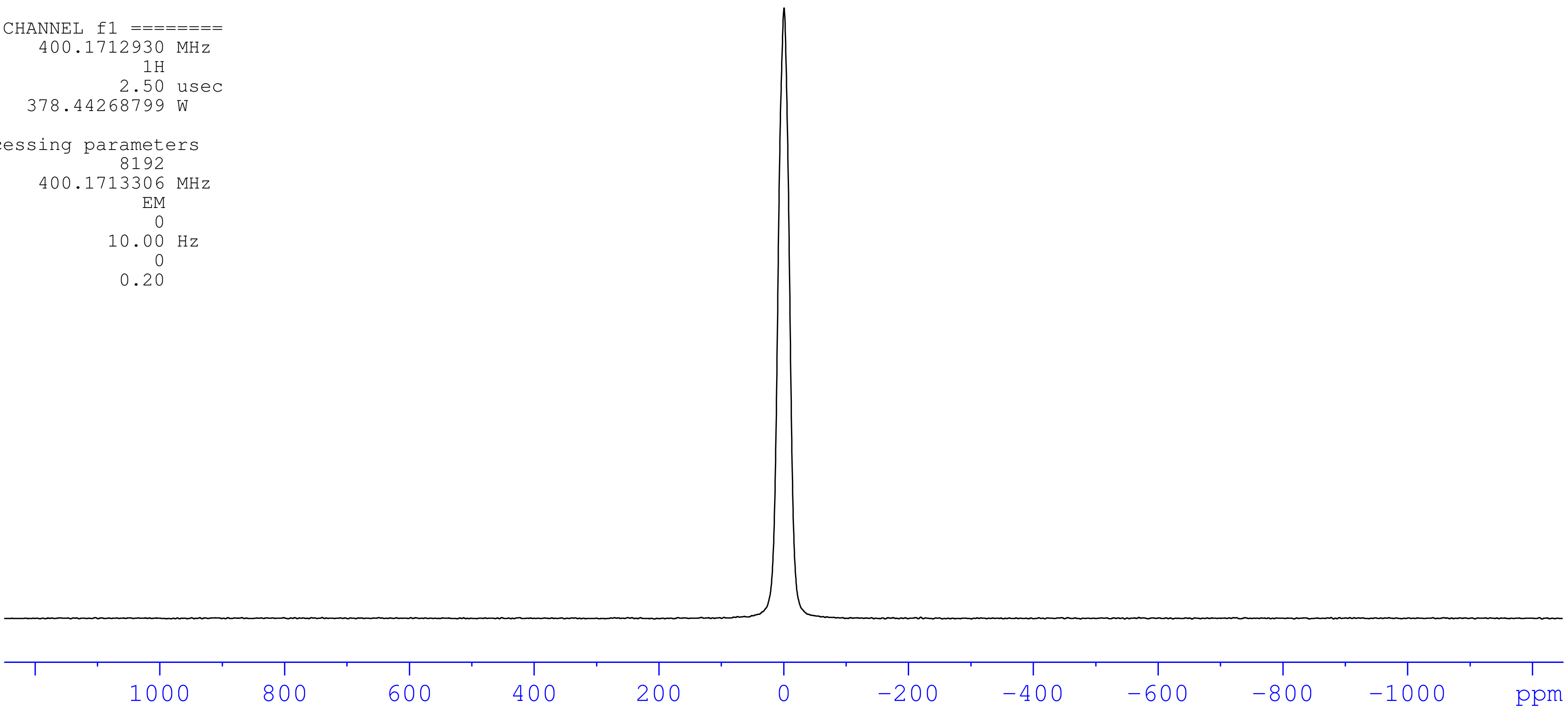

Current Data Parameters
NAME jse_20240130
EXPNO 97
PROCNO 1

F2 - Acquisition Parameters
Date_ 20240202
PROBHD 5 mm PE BB/1H/
PULPROG zg
NS 4
SWH 1000000.000 Hz
AQ 0.0005120 sec
TE 294.0 K
D1 7.19999981 sec
TD0 1

======== CHANNEL f1 ========
SFO1 400.1712930 MHz
NUC1 1H
P1 2.50 usec
PLW1 378.44268799 W

F2 - Processing parameters
SI 8192
SF 400.1713306 MHz
WDW EM
SSB 0
LB 10.00 Hz
GB 0
PC 0.20

2624-10340 SYT0043a S-CTA CdCl4 @ static / -100 to +120 C
90 C

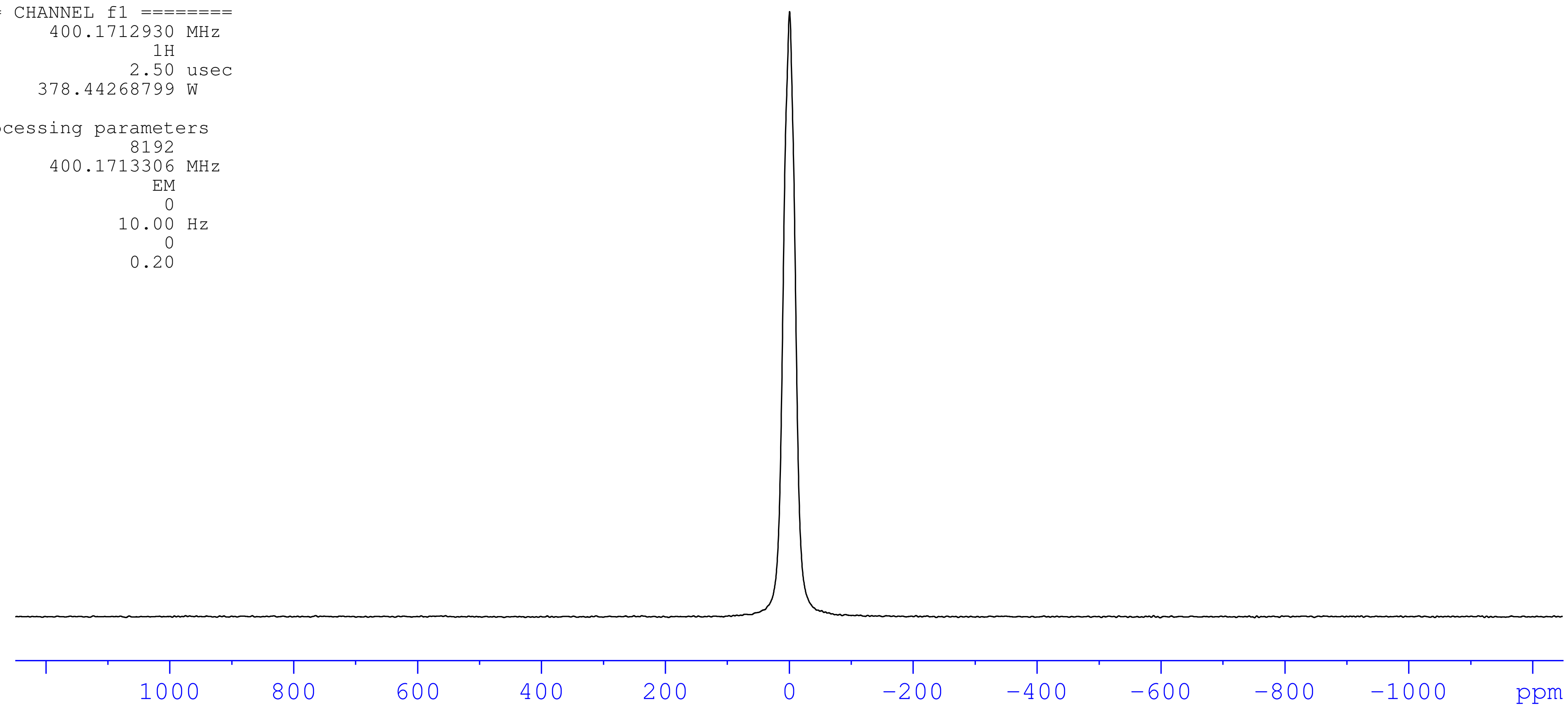

Current Data Parameters
NAME jse_20240130
EXPNO 98
PROCNO 1

F2 - Acquisition Parameters
Date_ 20240202
PROBHD 5 mm PE BB/1H/
PULPROG satrect1se
NS 8
SWH 1000000.000 Hz
AQ 0.0005120 sec
TE 298.0 K
D1 1.00000000 sec
D6 0.00004625 sec
D7 0.00002750 sec
D20 0.00040000 sec
L20 64
VDLIST Recovery_0.1_102.4_16

======== CHANNEL f1 ========
SFO1 400.1712930 MHz
NUC1 1H
P1 2.50 usec
PLW1 378.44268799 W

F1 - Acquisition parameters
TD 16
SFO1 400.1713 MHz
FIDRES 500.000000 Hz
SW 9.996 ppm
FnMODE QF

F2 - Processing parameters
SI 8192
SF 400.1713306 MHz
WDW no
SSB 0
LB 0 Hz
GB 0
PC 0.20

F1 - Processing parameters
SI 16
MC2 QF
SF 400.1700000 MHz
WDW no
SSB 0
LB 0 Hz
GB 0

2624-10340 SYT0043a S-CTA CdCl4 @ static / -100 to +120 C
90 C

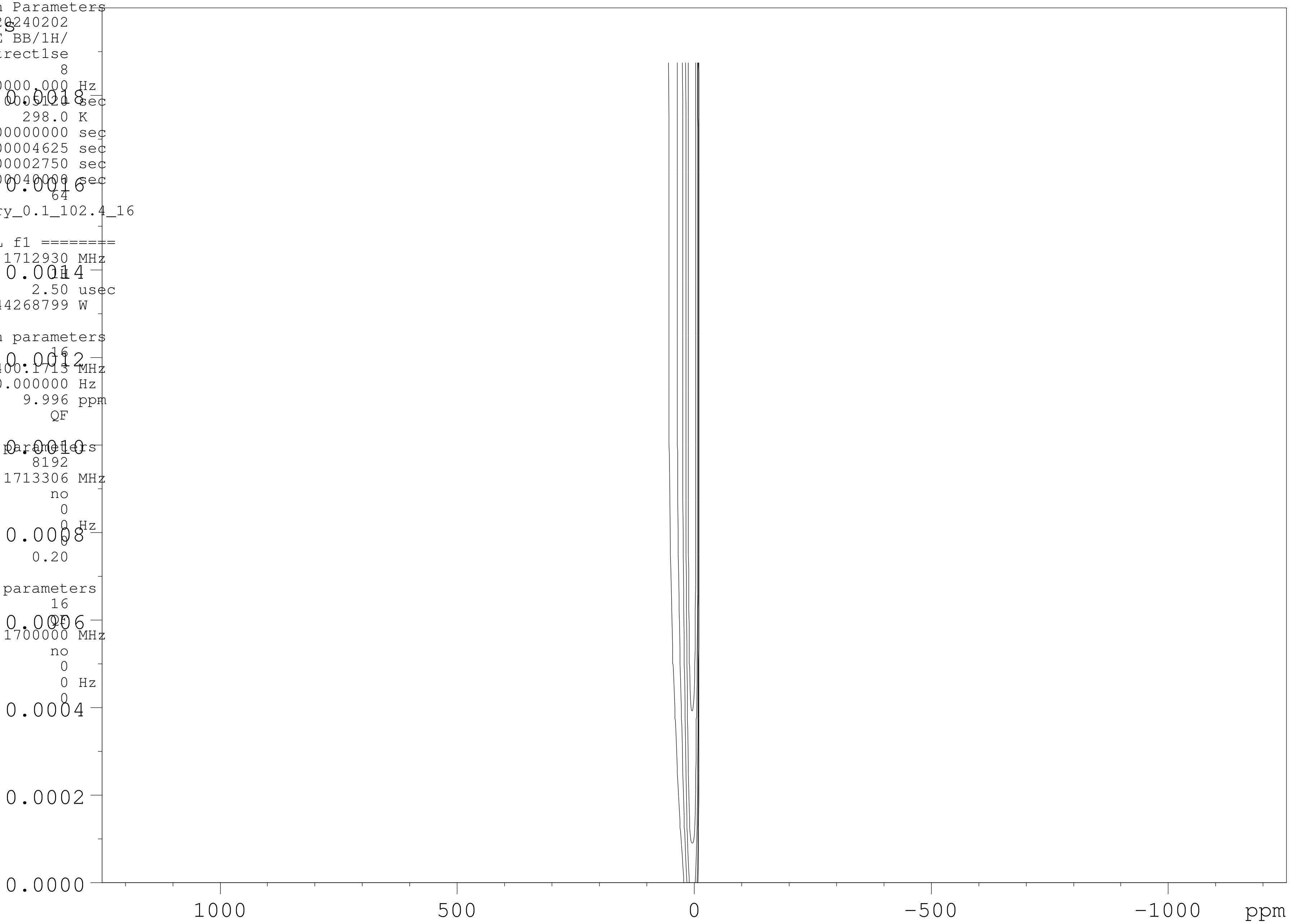

2624-10340 SYT0043a S-CTA CdCl4 @ static / -100 to +120 C
90 C

```
Current Data Parameters
NAME       jse_20240130
EXPNO                99
PROCNO                1

F2 - Acquisition Parameters
Date_          20240202
PROBHD   5 mm PE BB/1H/
PULPROG  t1rho_solidecho
NS                    8
SWH         1000000.000 Hz
AQ            0.0005120 sec
TE                347.6 K
D1           7.19999981 sec
D6           0.00005000 sec
D7           0.00002750 sec

======== CHANNEL f1 ========
SFO1        400.1712930 MHz
NUC1                 1H
P1                 2.50 usec
PLW1       378.44268799 W
PLW2        94.62400055 W
VPLIST   100u_52000u_16

F1 - Acquisition parameters
TD                    8
SFO1           400.1713 MHz
FIDRES      1000.000000 Hz
SW                9.996 ppm
FnMODE               QF

F2 - Processing parameters
SI                 8192
SF          400.1713306 MHz
WDW                  no
SSB                   0
LB                    0 Hz
GB                    0
PC                 0.20

F1 - Processing parameters
SI                   16
MC2                  QF
SF          400.1700000 MHz
WDW                  no
SSB                   0
LB                    0 Hz
GB                    0
```

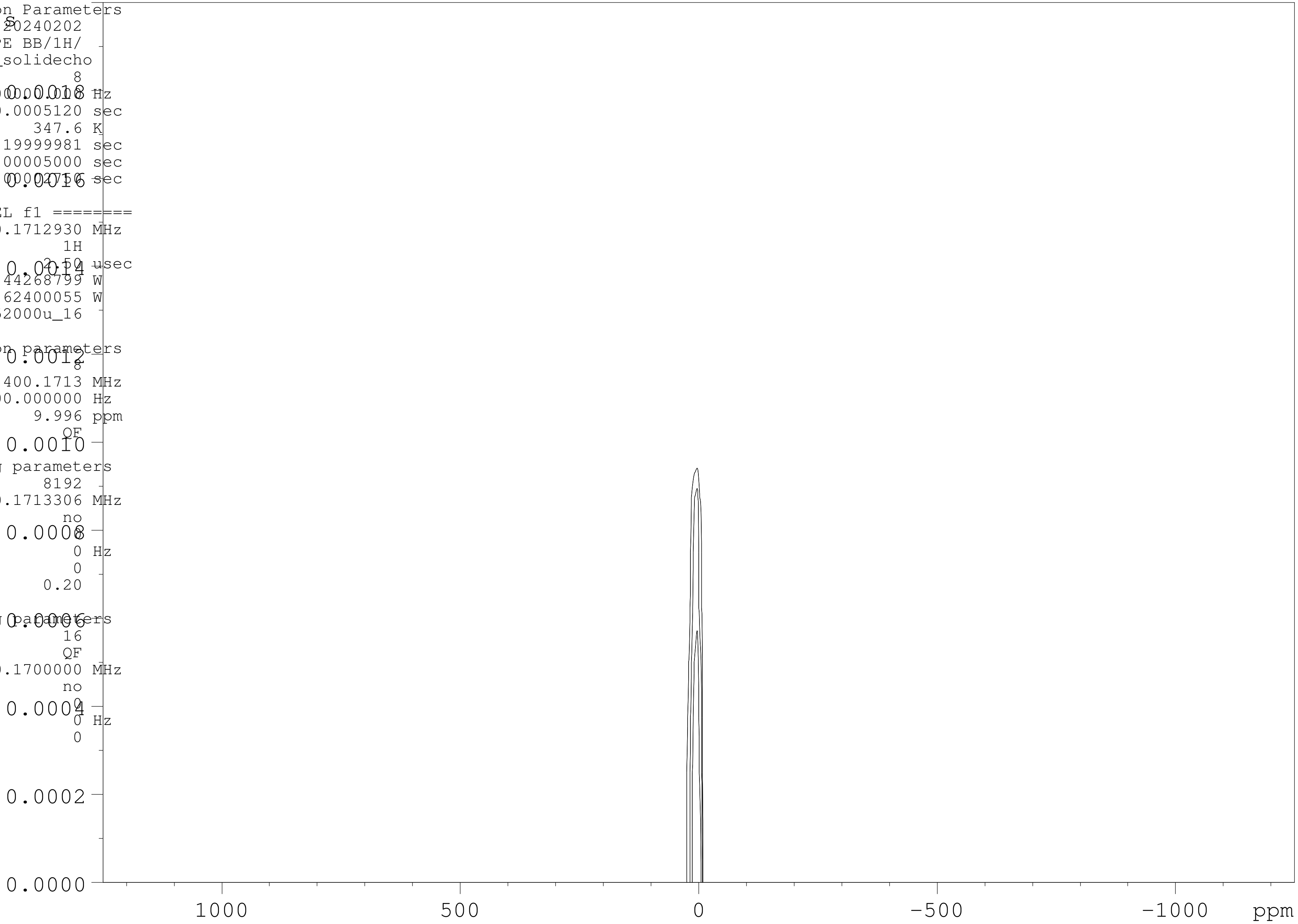

Current Data Parameters
NAME jse_20240130
EXPNO 101
PROCNO 1

F2 - Acquisition Parameters
Date_ 20240202
PROBHD 5 mm PE BB/1H/
PULPROG solidecho
NS 8
SWH 1000000.000 Hz
AQ 0.0005120 sec
TE 334.2 K
D1 7.19999981 sec
D6 0.00005000 sec
D7 0.00002750 sec

======== CHANNEL f1 ========
SFO1 400.1712930 MHz
NUC1 1H
P1 2.50 usec
PLW1 378.44268799 W

F2 - Processing parameters
SI 8192
SF 400.1713306 MHz
WDW EM
SSB 0
LB 10.00 Hz
GB 0
PC 0.20

2624-10340 SYT0043a S-CTA $CdCl_4$ @ static / -100 to +120 C
100 C

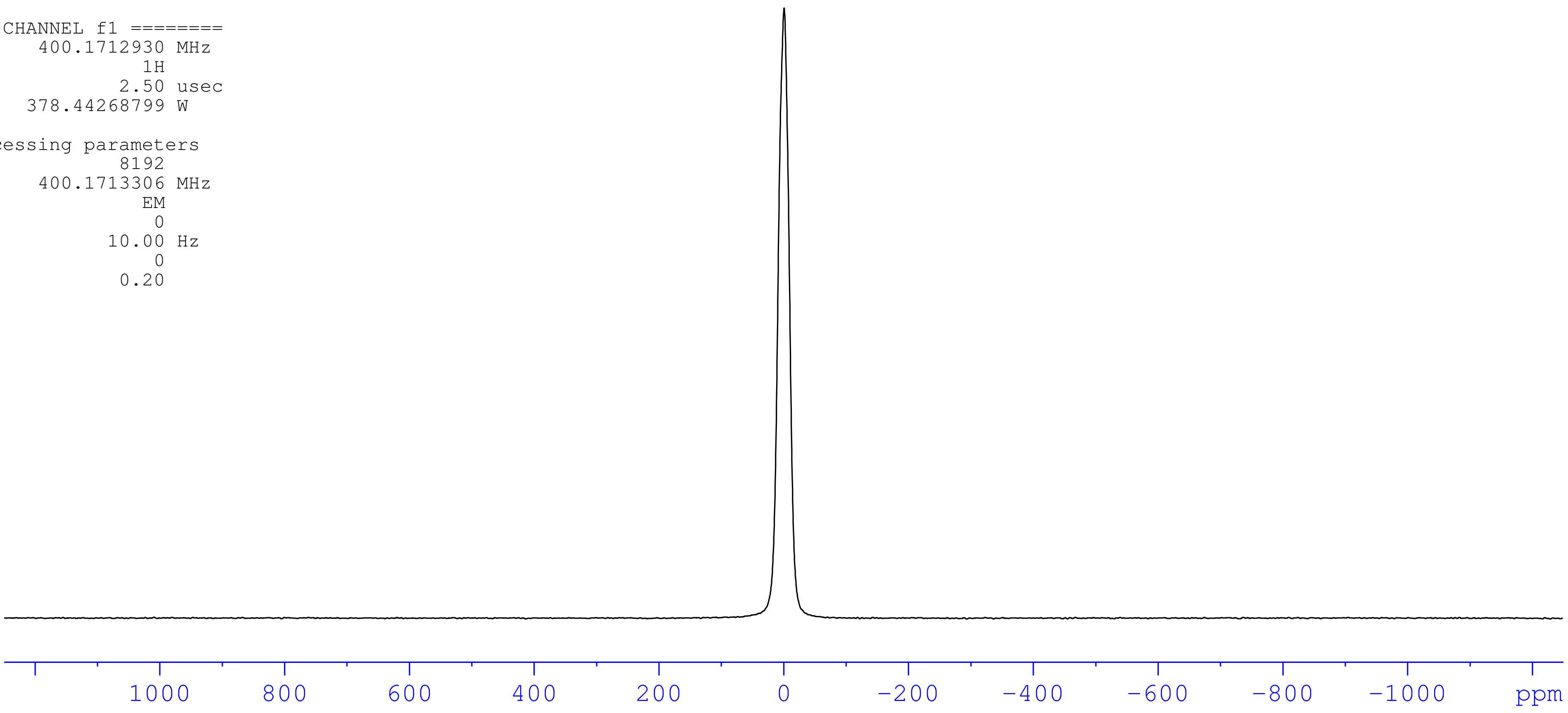

Current Data Parameters
NAME jse_20240130
EXPNO 102
PROCNO 1

F2 - Acquisition Parameters
Date_ 20240202
PROBHD 5 mm PE BB/1H/
PULPROG zg
NS 4
SWH 1000000.000 Hz
AQ 0.0005120 sec
TE 294.0 K
D1 7.19999981 sec
TD0 1

======== CHANNEL f1 ========
SFO1 400.1712930 MHz
NUC1 1H
P1 2.50 usec
PLW1 378.44268799 W

F2 - Processing parameters
SI 8192
SF 400.1713306 MHz
WDW EM
SSB 0
LB 10.00 Hz
GB 0
PC 0.20

2624-10340 SYT0043a S-CTA CdCl4 @ static / -100 to +120 C
100 C

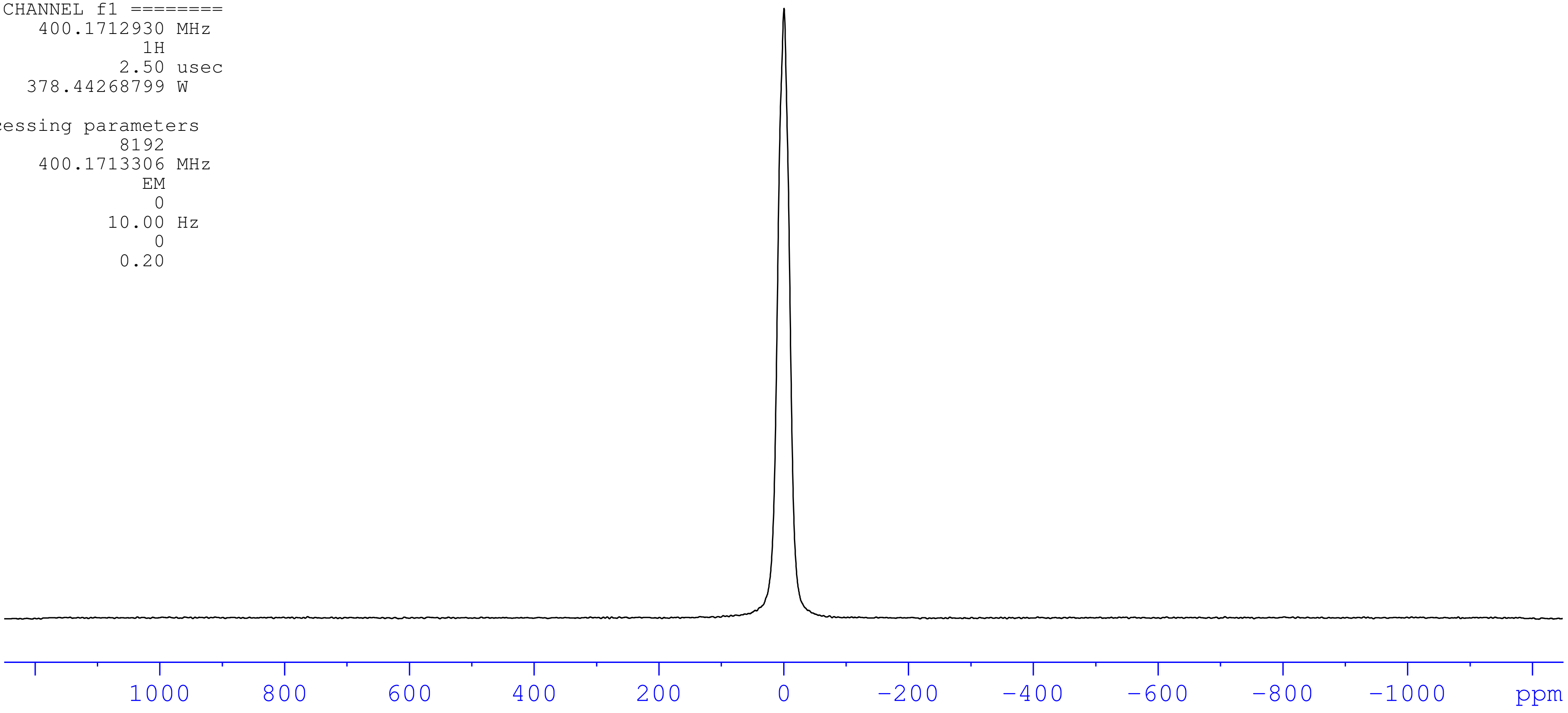

2624-10340 SYT0043a S-CTA CdCl4 @ static / -100 to +120 C
100 C

Current Data Parameters

NAME	jse_20240130
EXPNO	103
PROCNO	1

F2 - Acquisition Parameters

Date_	20240202
PROBHD	5 mm PE BB/1H/
PULPROG	satrect1se
NS	8
SWH	1000000.000 Hz
AQ	0.0005120 sec
TE	298.0 K
D1	1.00000000 sec
D6	0.00004625 sec
D7	0.00002750 sec
D20	0.00040000 sec
L20	64
VDLIST	Recovery_0.1_102.4_16

======== CHANNEL f1 ========

SFO1	400.1712930 MHz
NUC1	1H
P1	2.50 usec
PLW1	378.44268799 W

F1 - Acquisition parameters

TD	16
SFO1	400.1713 MHz
FIDRES	500.000000 Hz
SW	9.996 ppm
FnMODE	QF

F2 - Processing parameters

SI	8192
SF	400.1713306 MHz
WDW	no
SSB	0
LB	0 Hz
GB	0
PC	0.20

F1 - Processing parameters

SI	16
MC2	QF
SF	400.1700000 MHz
WDW	no
SSB	0
LB	0 Hz
GB	0

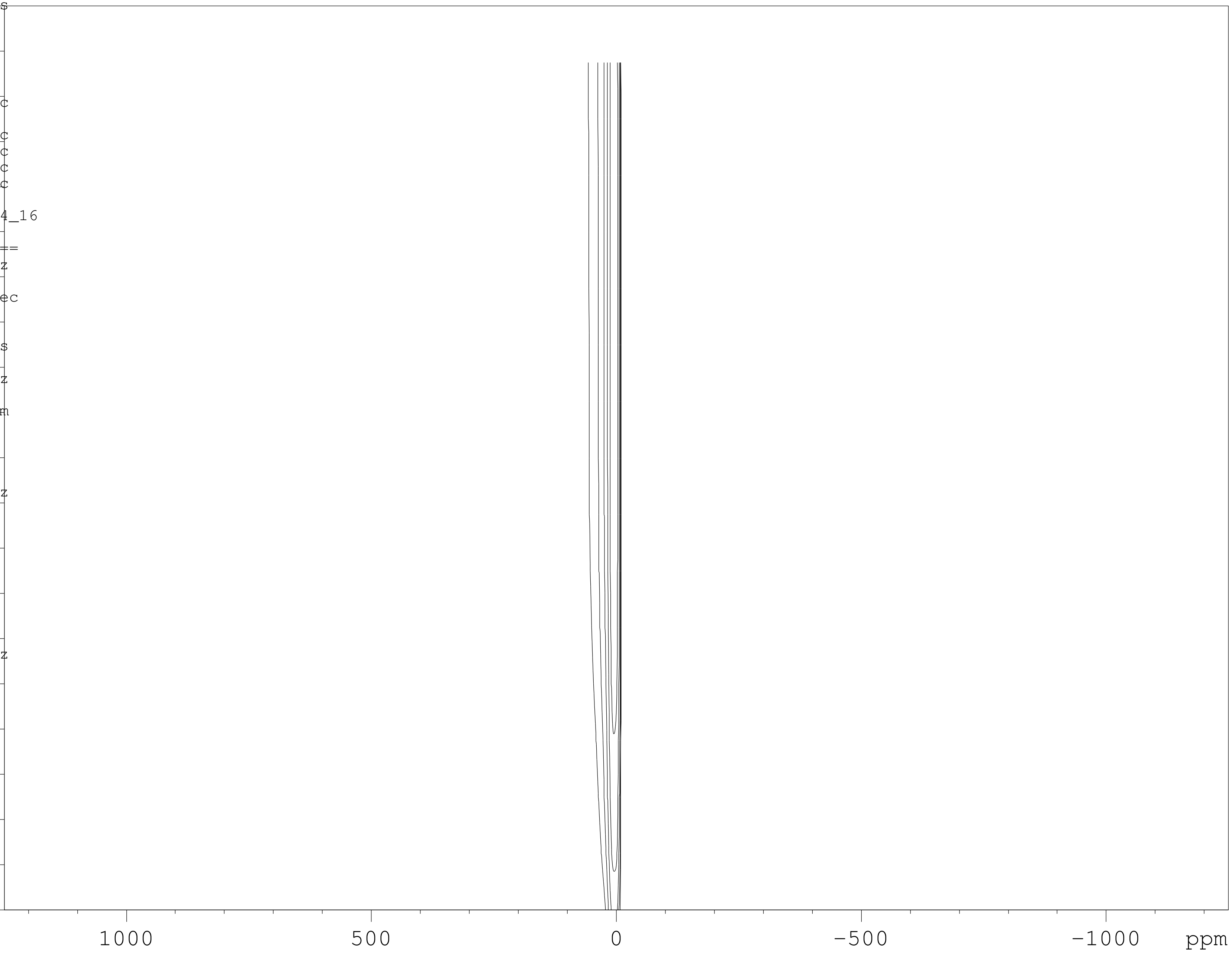

2624-10340 SYT0043a S-CTA CdCl4 @ static / -100 to +120 C
100 C

Current Data Parameters
NAME jse_20240130
EXPNO 104
PROCNO 1

F2 - Acquisition Parameters
Date_ 20240202
PROBHD 5 mm PE BB/1H/
PULPROG t1rho_solidecho
NS 8
SWH 1000000.000 Hz
AQ 0.0005120 sec
TE 354.4 K
D1 7.19999981 sec
D6 0.00005000 sec
D7 0.0000275[illegible] sec

======== CHANNEL f1 ========
SFO1 400.1712930 MHz
NUC1 1H
P1 2.50 usec
PLW1 378.44268799 W
PLW2 94.62400055 W
VPLIST 100u_52000u_16

F1 - Acquisition parameters
TD 8
SFO1 400.1713 MHz
FIDRES 1000.000000 Hz
SW 9.996 ppm
FnMODE QF

F2 - Processing parameters
SI 8192
SF 400.1713306 MHz
WDW no
SSB 0
LB 0 Hz
GB 0
PC 0.20

F1 - Processing parameters
SI 16
MC2 QF
SF 400.1700000 MHz
WDW no
SSB 0
LB 0 Hz
GB 0

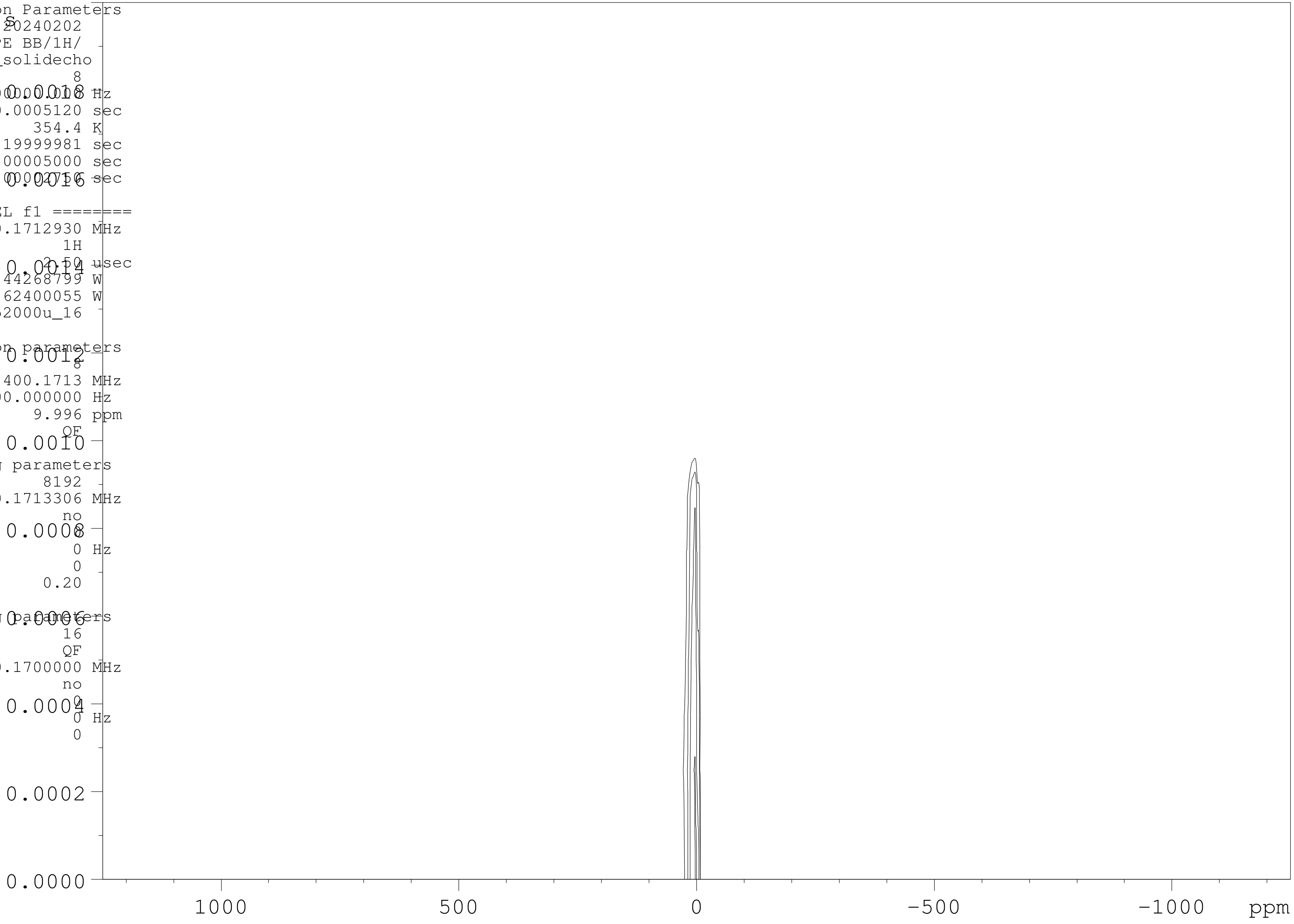

Current Data Parameters
NAME jse_20240130
EXPNO 106
PROCNO 1

F2 - Acquisition Parameters
Date_ 20240202
PROBHD 5 mm PE BB/1H/
PULPROG solidecho
NS 8
SWH 1000000.000 Hz
AQ 0.0005120 sec
TE 334.2 K
D1 7.19999981 sec
D6 0.00005000 sec
D7 0.00002750 sec

======== CHANNEL f1 ========
SFO1 400.1712930 MHz
NUC1 1H
P1 2.50 usec
PLW1 378.44268799 W

F2 - Processing parameters
SI 8192
SF 400.1713306 MHz
WDW EM
SSB 0
LB 10.00 Hz
GB 0
PC 0.20

2624-10340 SYT0043a S-CTA CdCl4 @ static / -100 to +120 C
110 C

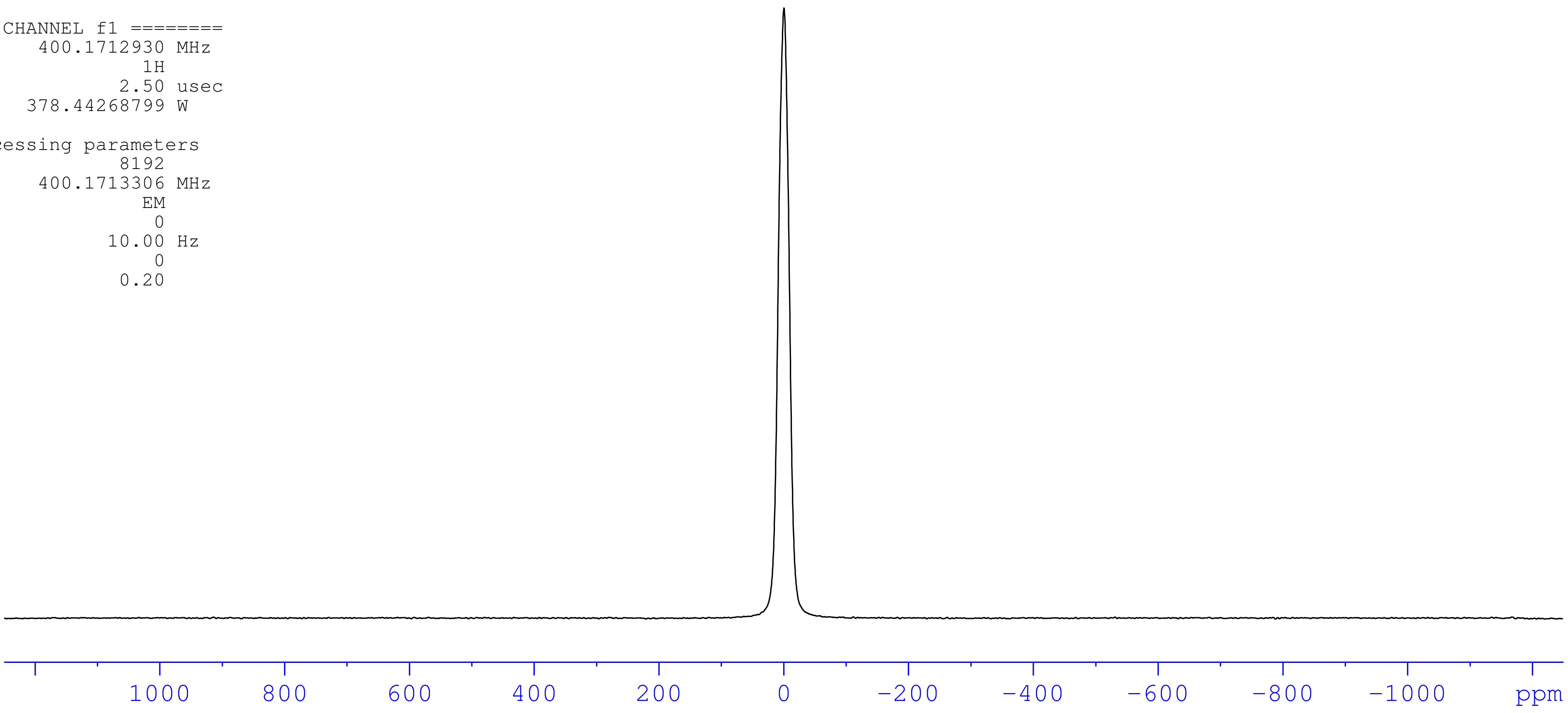

2624-10340 SYT0043a S-CTA CdCl4 @ static / -100 to +120 C
110 C

```
Current Data Parameters
NAME       jse_20240130
EXPNO               107
PROCNO                1

F2 - Acquisition Parameters
Date_          20240202
PROBHD   5 mm PE BB/1H/
PULPROG              zg
NS                    4
SWH         1000000.000 Hz
AQ            0.0005120 sec
TE                294.0 K
D1           7.19999981 sec
TD0                   1

======== CHANNEL f1 ========
SFO1        400.1712930 MHz
NUC1                 1H
P1                 2.50 usec
PLW1       378.44268799 W

F2 - Processing parameters
SI                 8192
SF          400.1713306 MHz
WDW                  EM
SSB                   0
LB                10.00 Hz
GB                    0
PC                 0.20
```

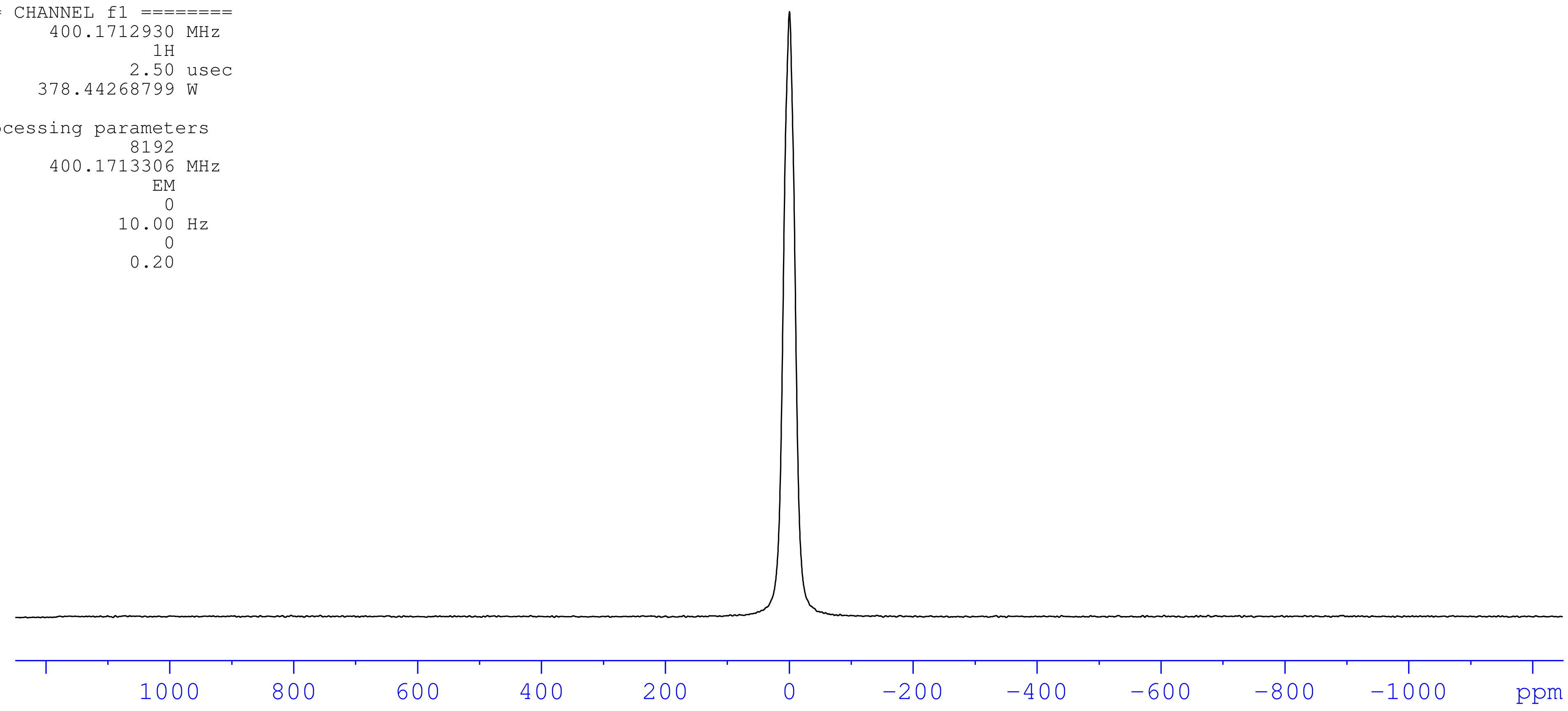

2624-10340 SYT0043a S-CTA CdCl4 @ static / -100 to +120 C
110 C

Current Data Parameters
NAME jse_20240130
EXPNO 108
PROCNO 1

F2 - Acquisition Parameters
Date_ 20240202
PROBHD 5 mm PE BB/1H/
PULPROG satrect1se
NS 8
SWH 1000000.000 Hz
AQ 0.0005120 sec
TE 298.0 K
D1 1.00000000 sec
D6 0.00004625 sec
D7 0.00002750 sec
D20 0.00040000 sec
L20 64
VDLIST Recovery_0.1_102.4_16

======== CHANNEL f1 ========
SFO1 400.1712930 MHz
NUC1 1H
P1 2.50 usec
PLW1 378.44268799 W

F1 - Acquisition parameters
TD 16
SFO1 400.1713 MHz
FIDRES 500.000000 Hz
SW 9.996 ppm
FnMODE QF

F2 - Processing parameters
SI 8192
SF 400.1713306 MHz
WDW no
SSB 0
LB 0 Hz
GB 0
PC 0.20

F1 - Processing parameters
SI 16
MC2 QF
SF 400.1700000 MHz
WDW no
SSB 0
LB 0 Hz
GB 0

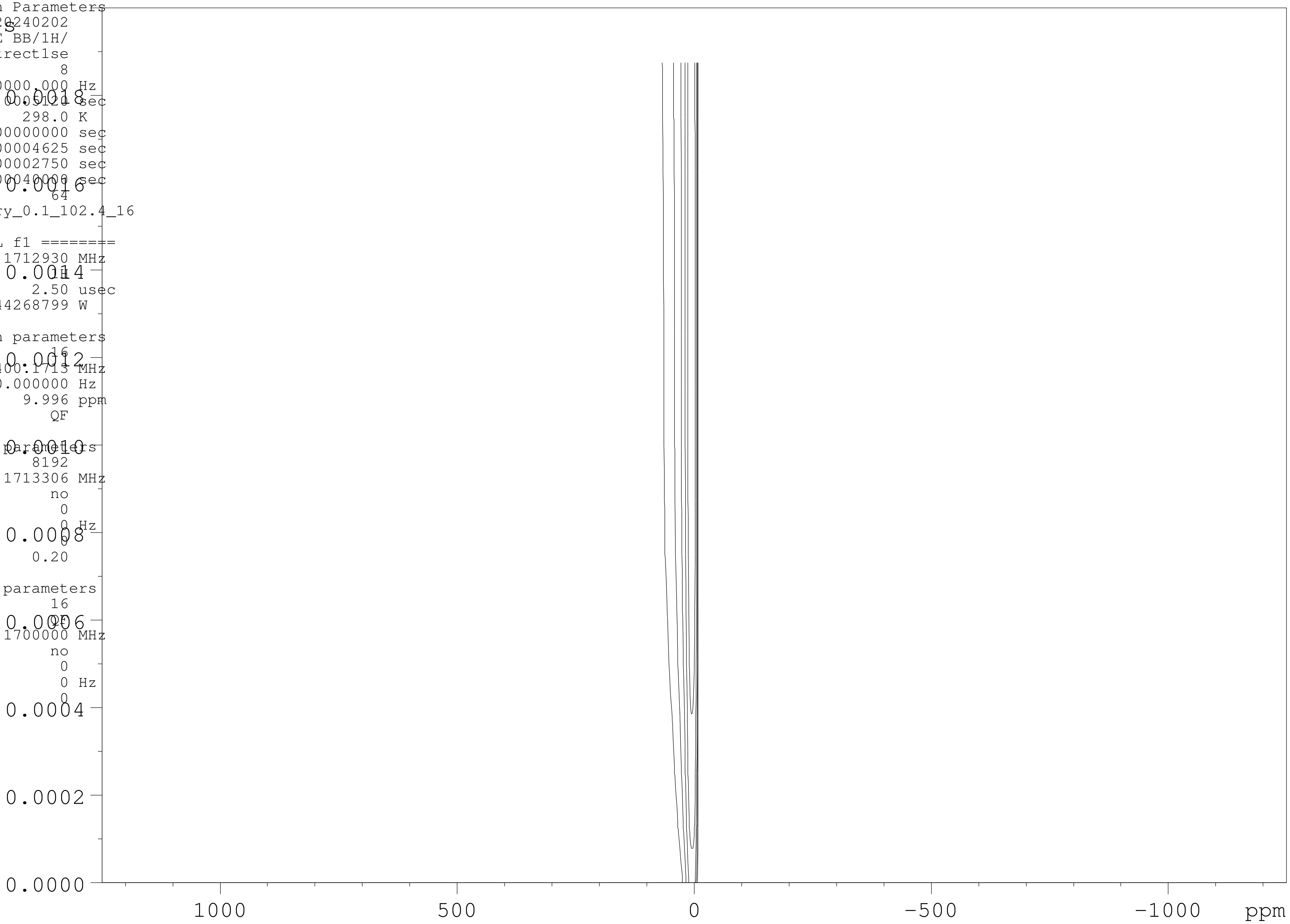

Current Data Parameters
NAME jse_20240130
EXPNO 109
PROCNO 1

F2 - Acquisition Parameters
Date_ 20240202
PROBHD 5 mm PE BB/1H/
PULPROG t1rho_solidecho
NS 8
SWH 1000000.000 Hz
AQ 0.0005120 sec
TE 361.1 K
D1 7.19999981 sec
D6 0.00005000 sec
D7 0.0000275[illegible] sec

======== CHANNEL f1 ========
SFO1 400.1712930 MHz
NUC1 1H
P1 2.50 usec
PLW1 378.44268799 W
PLW2 94.62400055 W
VPLIST 100u_52000u_16

F1 - Acquisition parameters
TD 8
SFO1 400.1713 MHz
FIDRES 1000.000000 Hz
SW 9.996 ppm
FnMODE QF

F2 - Processing parameters
SI 8192
SF 400.1713306 MHz
WDW no
SSB 0
LB 0 Hz
GB 0
PC 0.20

F1 - Processing parameters
SI 16
MC2 QF
SF 400.1700000 MHz
WDW no
SSB 0
LB 0 Hz
GB 0

2624-10340 SYT0043a S-CTA CdCl4 @ static / -100 to +120 C
110 C

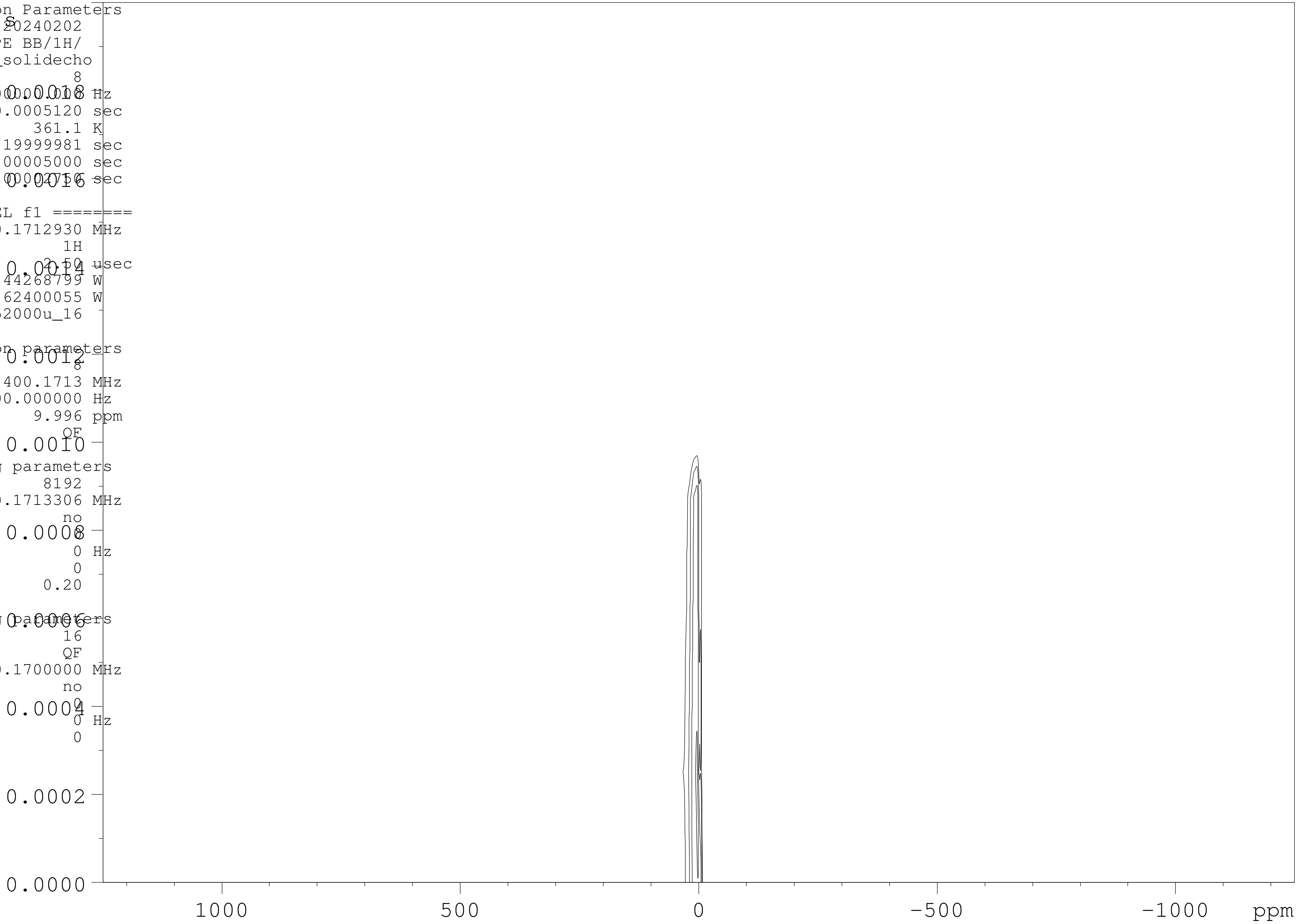

Current Data Parameters
NAME jse_20240130
EXPNO 111
PROCNO 1

F2 - Acquisition Parameters
Date_ 20240202
PROBHD 5 mm PE BB/1H/
PULPROG solidecho
NS 8
SWH 1000000.000 Hz
AQ 0.0005120 sec
TE 334.2 K
D1 7.19999981 sec
D6 0.00005000 sec
D7 0.00002750 sec

======== CHANNEL f1 ========
SFO1 400.1712930 MHz
NUC1 1H
P1 2.50 usec
PLW1 378.44268799 W

F2 - Processing parameters
SI 8192
SF 400.1713306 MHz
WDW EM
SSB 0
LB 10.00 Hz
GB 0
PC 0.20

2624-10340 SYT0043a S-CTA CdCl4 @ static / -100 to +120 C
120 C

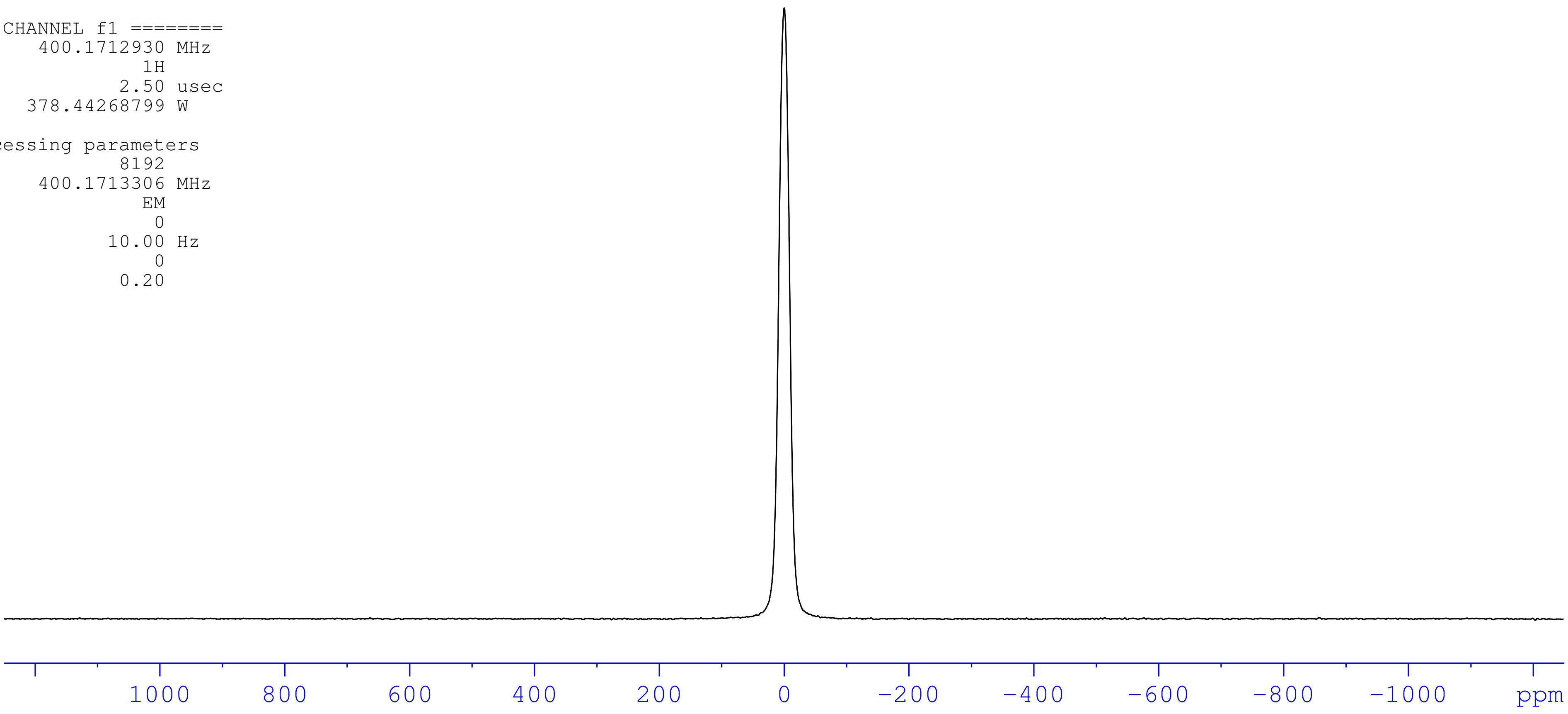

Current Data Parameters
NAME jse_20240130
EXPNO 112
PROCNO 1

F2 - Acquisition Parameters
Date_ 20240202
PROBHD 5 mm PE BB/1H/
PULPROG zg
NS 4
SWH 1000000.000 Hz
AQ 0.0005120 sec
TE 294.0 K
D1 7.19999981 sec
TD0 1

======== CHANNEL f1 ========
SFO1 400.1712930 MHz
NUC1 1H
P1 2.50 usec
PLW1 378.44268799 W

F2 - Processing parameters
SI 8192
SF 400.1713306 MHz
WDW EM
SSB 0
LB 10.00 Hz
GB 0
PC 0.20

2624-10340 SYT0043a S-CTA CdCl4 @ static / -100 to +120 C
120 C

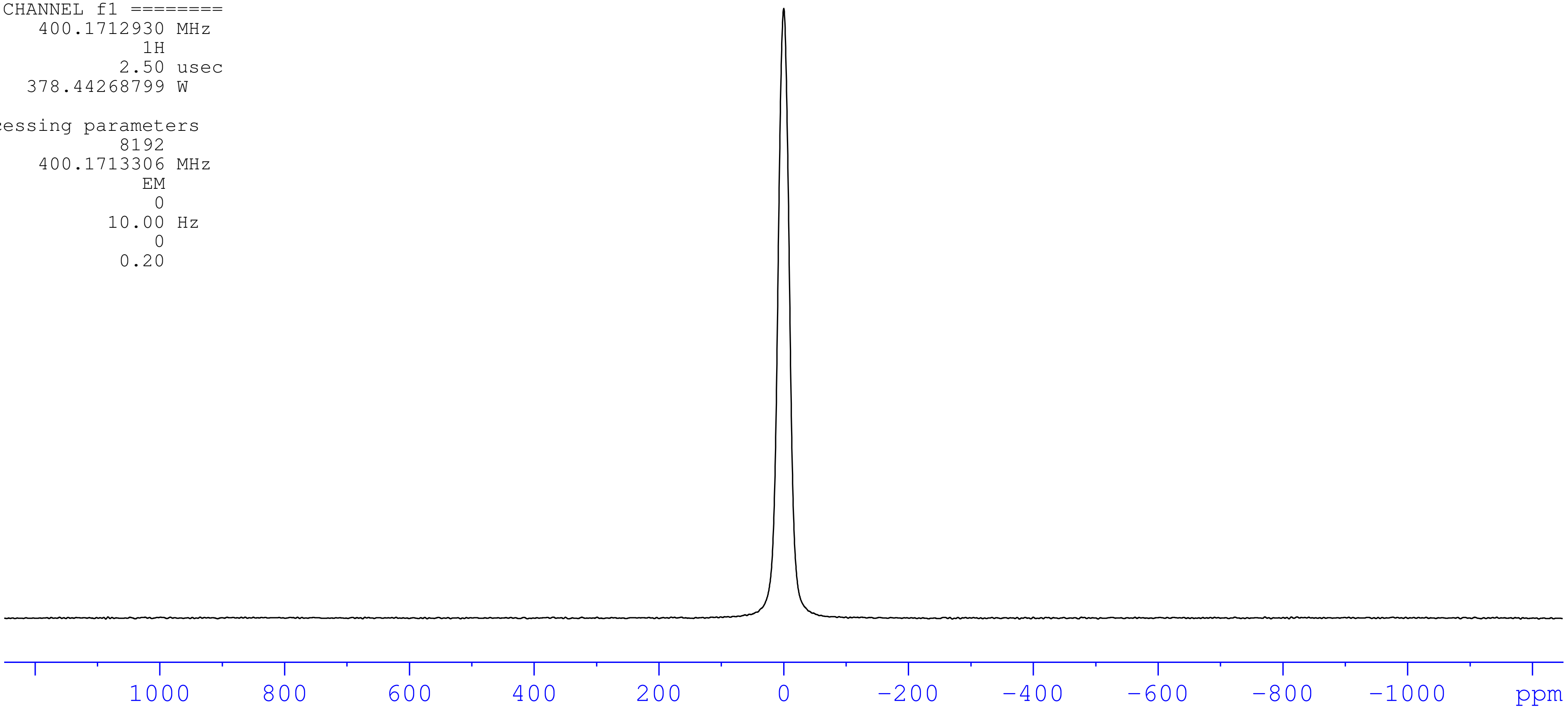

Current Data Parameters
NAME jse_20240130
EXPNO 113
PROCNO 1

F2 - Acquisition Parameters
Date_ 20240202
PROBHD 5 mm PE BB/1H/
PULPROG satrect1se
NS 8
SWH 1000000.000 Hz
AQ 0.0005120 sec
TE 298.0 K
D1 1.00000000 sec
D6 0.00004625 sec
D7 0.00002750 sec
D20 0.00040000 sec
L20 64
VDLIST Recovery_0.1_102.4_16

======== CHANNEL f1 ========
SFO1 400.1712930 MHz
NUC1 1H
P1 2.50 usec
PLW1 378.44268799 W

F1 - Acquisition parameters
TD 16
SFO1 400.1713 MHz
FIDRES 500.000000 Hz
SW 9.996 ppm
FnMODE QF

F2 - Processing parameters
SI 8192
SF 400.1713306 MHz
WDW no
SSB 0
LB 0 Hz
GB 0
PC 0.20

F1 - Processing parameters
SI 16
MC2 QF
SF 400.1700000 MHz
WDW no
SSB 0
LB 0 Hz
GB 0

2624-10340 SYT0043a S-CTA CdCl4 @ static / -100 to +120 C
120 C

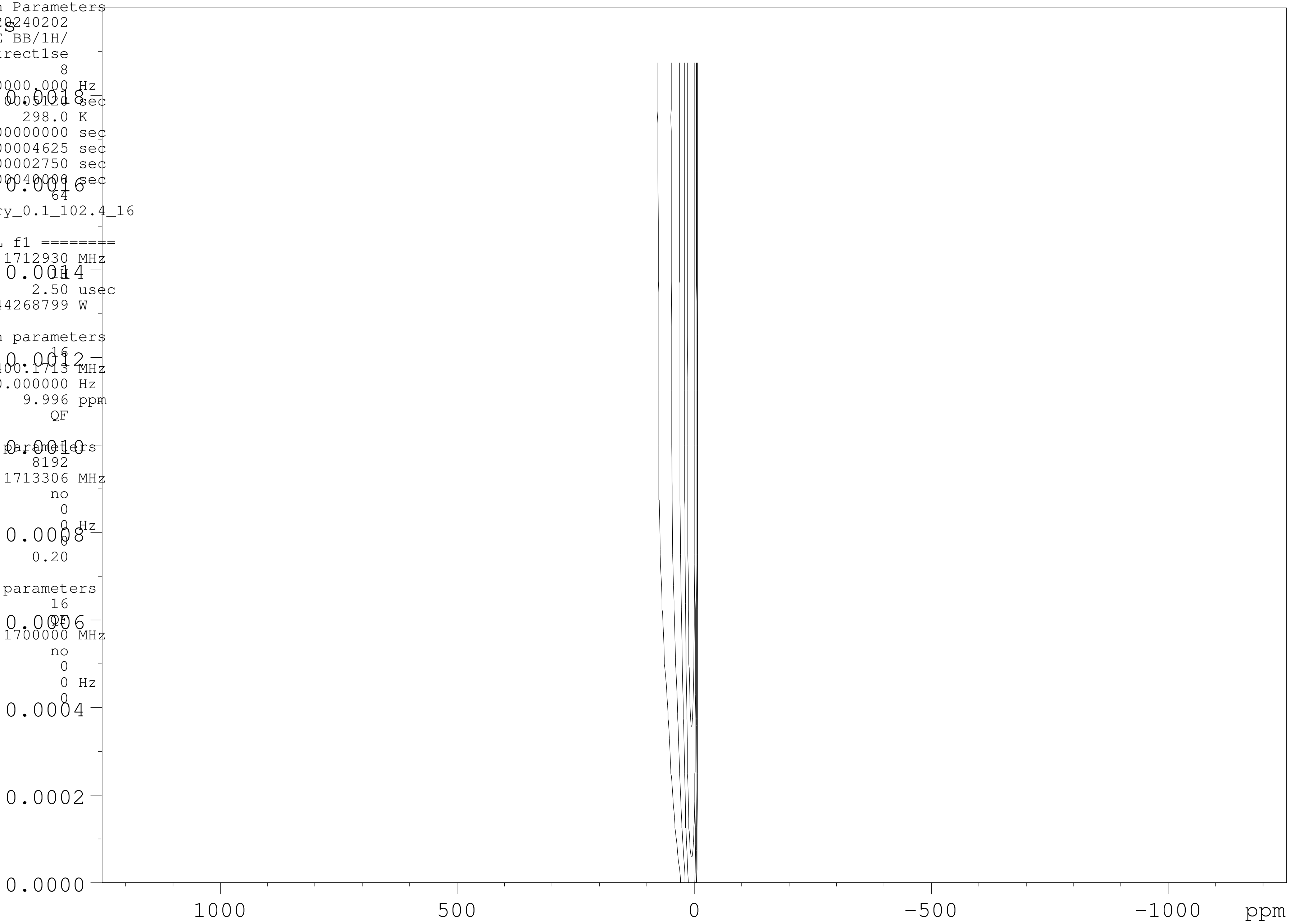

Current Data Parameters
NAME jse_20240130
EXPNO 114
PROCNO 1

2624-10340 SYT0043a S-CTA CdCl4 @ static / -100 to +120 C
120 C

F2 - Acquisition Parameters
Date_ 20240202
PROBHD 5 mm PE BB/1H/
PULPROG t1rho_solidecho
NS 8
SWH 1000000.000 Hz
AQ 0.0005120 sec
TE 294.0 K
D1 7.19999981 sec
D6 0.00005000 sec
D7 0.00002756 sec

======== CHANNEL f1 ========
SFO1 400.1712930 MHz
NUC1 1H
P1 2.50 usec
PLW1 378.44268799 W
PLW2 94.62400055 W
VPLIST 100u_52000u_16

F1 - Acquisition parameters
TD 8
SFO1 400.1713 MHz
FIDRES 1000.000000 Hz
SW 9.996 ppm
FnMODE QF

F2 - Processing parameters
SI 8192
SF 400.1713306 MHz
WDW no
SSB 0
LB 0 Hz
GB 0
PC 0.20

F1 - Processing parameters
SI 16
MC2 QF
SF 400.1700000 MHz
WDW no
SSB 0
LB 0 Hz
GB 0

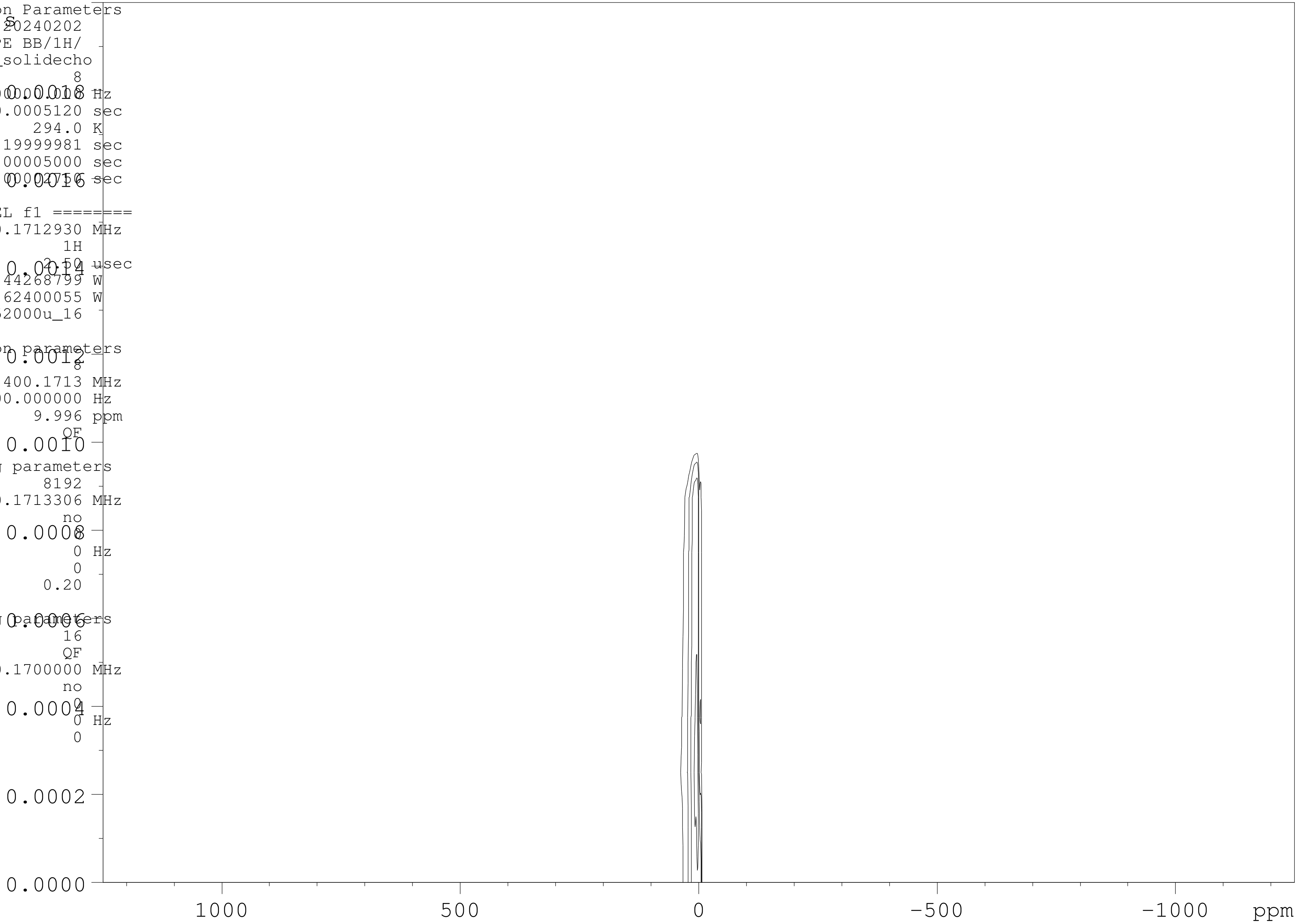

Current Data Parameters
NAME jse_20240130
EXPNO 116
PROCNO 1

F2 - Acquisition Parameters
Date_ 20240201
PROBHD 5 mm PE BB/1H/
PULPROG solidecho
NS 8
SWH 1000000.000 Hz
AQ 0.0005120 sec
TE 294.0 K
D1 7.19999981 sec
D6 0.00005000 sec
D7 0.00002750 sec

======== CHANNEL f1 ========
SFO1 400.1712930 MHz
NUC1 1H
P1 2.50 usec
PLW1 378.44268799 W

F2 - Processing parameters
SI 8192
SF 400.1713306 MHz
WDW EM
SSB 0
LB 10.00 Hz
GB 0
PC 0.20

2624-10340 SYT0043a S-CTA CdCl4 @ static / -100 to +120 C
20 C

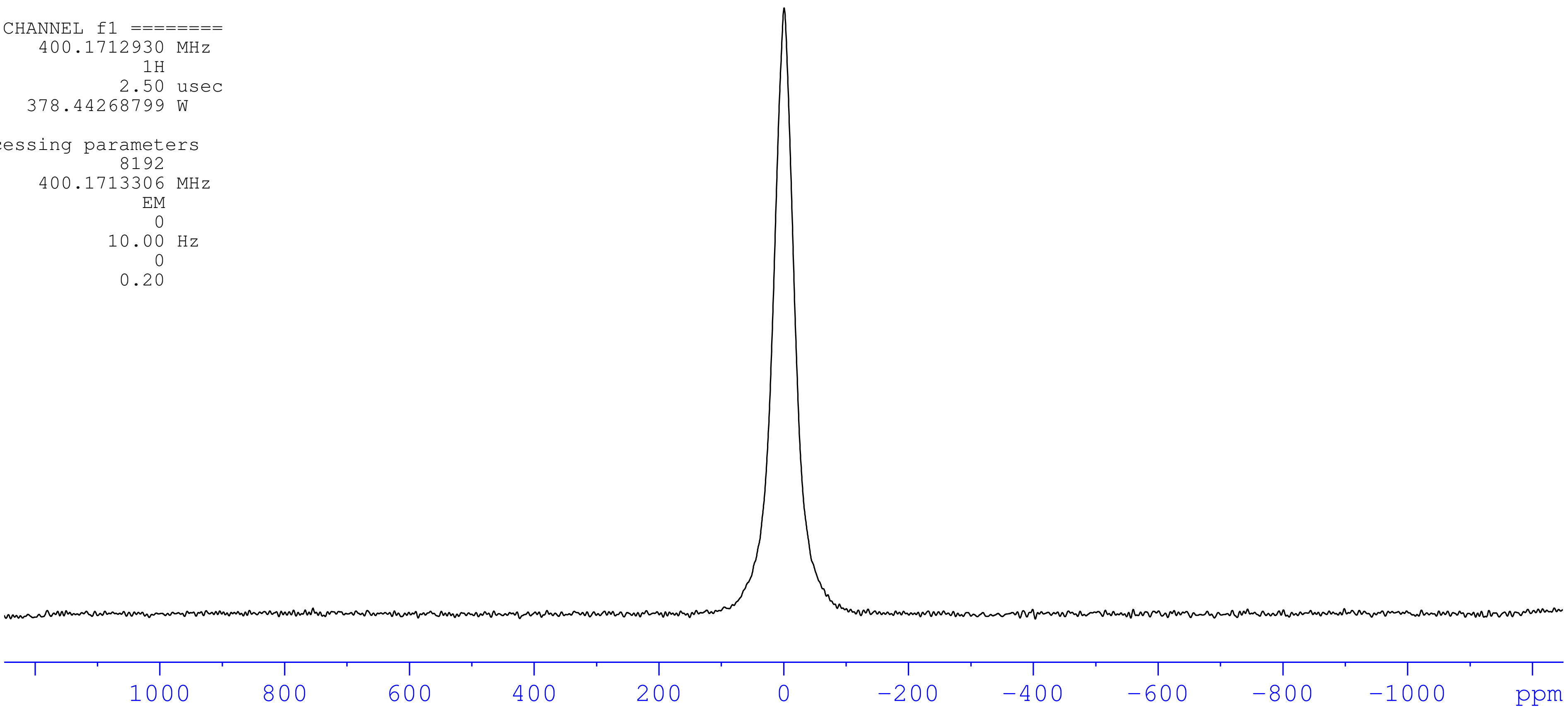

Current Data Parameters
NAME jse_20240130
EXPNO 117
PROCNO 1

F2 - Acquisition Parameters
Date_ 20240201
PROBHD 5 mm PE BB/1H/
PULPROG zg
NS 4
SWH 1000000.000 Hz
AQ 0.0005120 sec
TE 294.0 K
D1 7.19999981 sec
TD0 1

======== CHANNEL f1 ========
SFO1 400.1712930 MHz
NUC1 1H
P1 2.50 usec
PLW1 378.44268799 W

F2 - Processing parameters
SI 8192
SF 400.1713306 MHz
WDW EM
SSB 0
LB 10.00 Hz
GB 0
PC 0.20

2624-10340 SYT0043a S-CTA CdCl4 @ static / -100 to +120 C
20 C

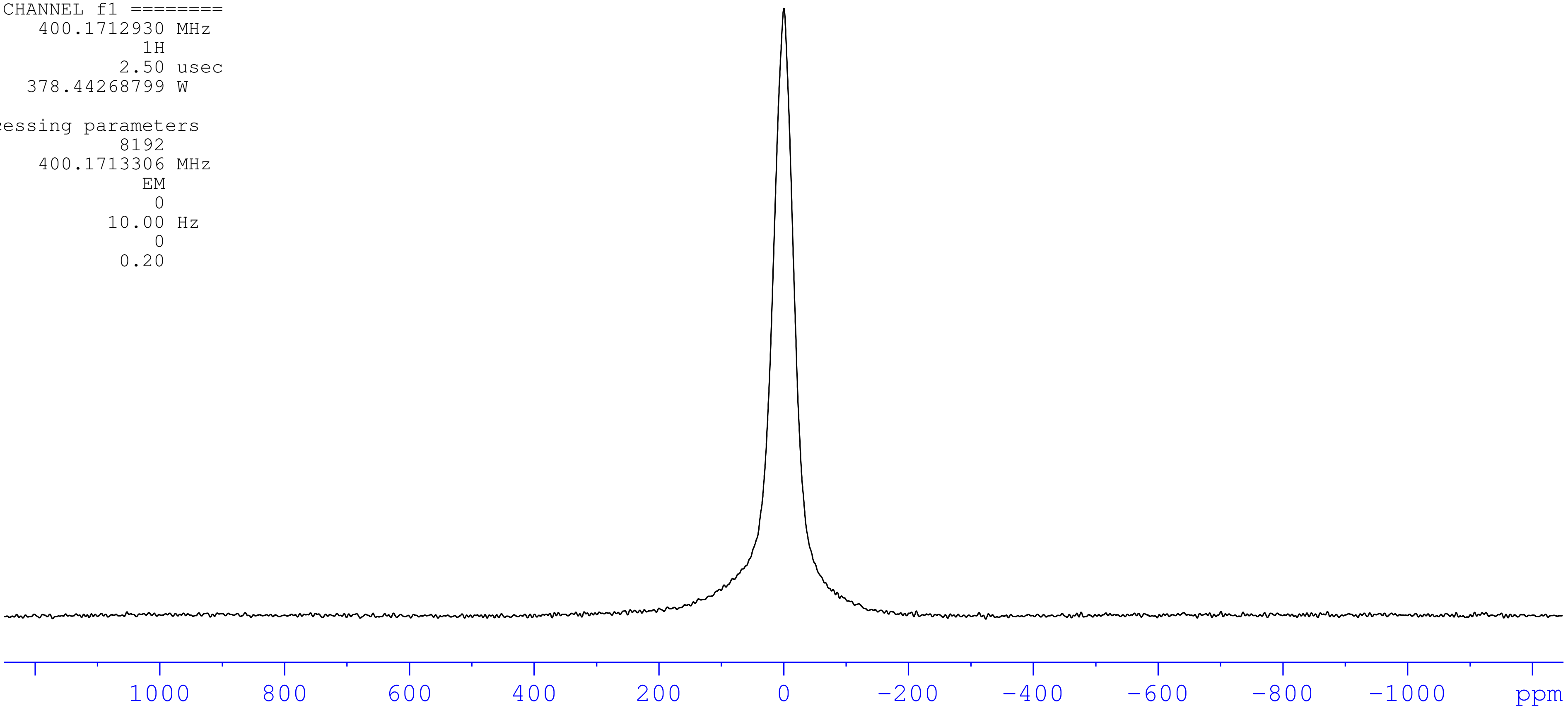

2624-10340 SYT0043a S-CTA CdCl4 @ static / -100 to +120 C
20 C

Current Data Parameters
NAME jse_20240130
EXPNO 118
PROCNO 1

F2 - Acquisition Parameters
Date_ 20240201
PROBHD 5 mm PE BB/1H/
PULPROG satrect1se
NS 8
SWH 1000000.000 Hz
AQ 0.0005120 sec
TE 298.0 K
D1 1.00000000 sec
D6 0.00004625 sec
D7 0.00002750 sec
D20 0.00040000 sec
L20 64
VDLIST Recovery_0.1_102.4_16

======== CHANNEL f1 ========
SFO1 400.1712930 MHz
NUC1 1H
P1 2.50 usec
PLW1 378.44268799 W

F1 - Acquisition parameters
TD 16
SFO1 400.1713 MHz
FIDRES 500.000000 Hz
SW 9.996 ppm
FnMODE QF

F2 - Processing parameters
SI 8192
SF 400.1713306 MHz
WDW no
SSB 0
LB 0 Hz
GB 0
PC 0.20

F1 - Processing parameters
SI 16
MC2 QF
SF 400.1700000 MHz
WDW no
SSB 0
LB 0 Hz
GB 0

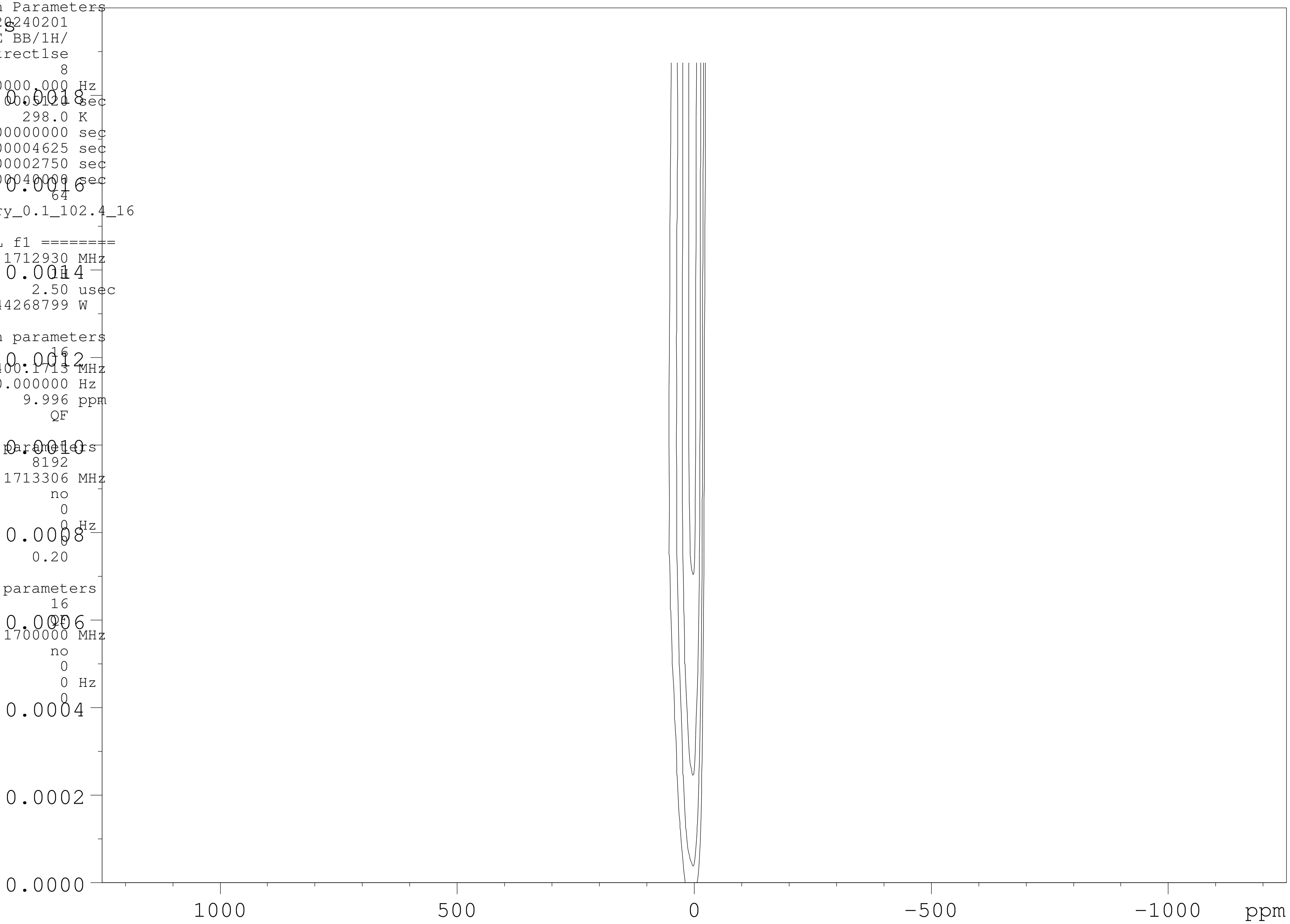

Current Data Parameters
NAME jse_20240130
EXPNO 119
PROCNO 1

F2 - Acquisition Parameters
Date_ 20240201
PROBHD 5 mm PE BB/1H/
PULPROG t1rho_solidecho
NS 8
SWH 1000000.000 Hz
AQ 0.0005120 sec
TE 300.7 K
D1 7.19999981 sec
D6 0.00005000 sec
D7 0.00002750 sec

======== CHANNEL f1 ========
SFO1 400.1712930 MHz
NUC1 1H
P1 2.50 usec
PLW1 378.44268799 W
PLW2 94.62400055 W
VPLIST 100u_52000u_16

F1 - Acquisition parameters
TD 8
SFO1 400.1713 MHz
FIDRES 1000.000000 Hz
SW 9.996 ppm
FnMODE QF

F2 - Processing parameters
SI 8192
SF 400.1713306 MHz
WDW no
SSB 0
LB 0 Hz
GB 0
PC 0.20

F1 - Processing parameters
SI 16
MC2 QF
SF 400.1700000 MHz
WDW no
SSB 0
LB 0 Hz
GB 0

2624-10340 SYT0043a S-CTA CdCl4 @ static / -100 to +120 C
20 C

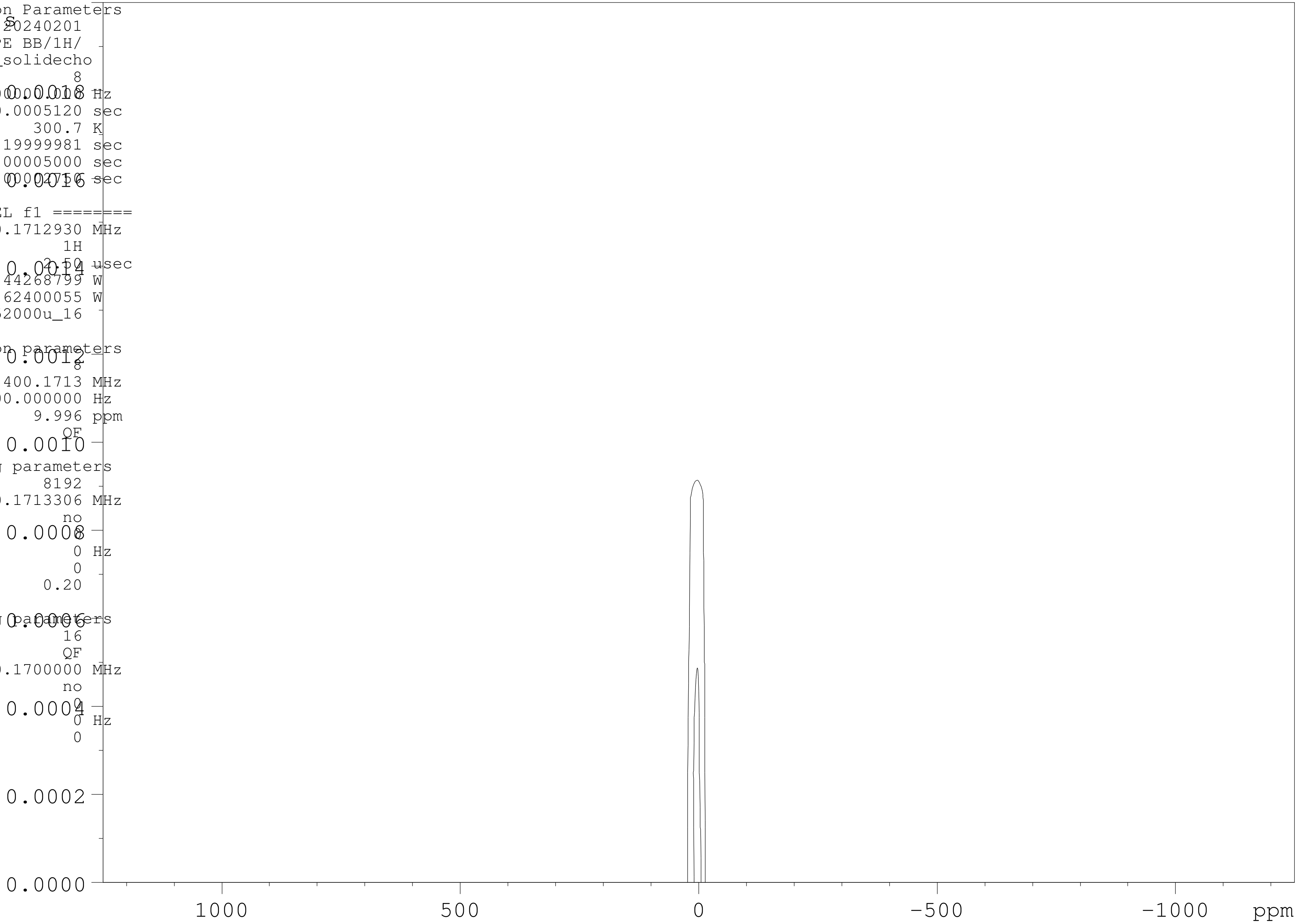

Supplement: Supplementary file 2 [file cg5c00666_si_002.zip › NMR/ssNMR data 2624c.pdf]
