# Supplementary figures and images for: Potential Multiaxial Molecular Ferroelectricity through Chiral Cation Replacement (part 2 of 2)
Source: Cryst Growth Des. 2025 Jul 21;25(15):6237–47. doi: 10.1021/acs.cgd.5c00666 (PMC12332970; doi:10.1021/acs.cgd.5c00666)

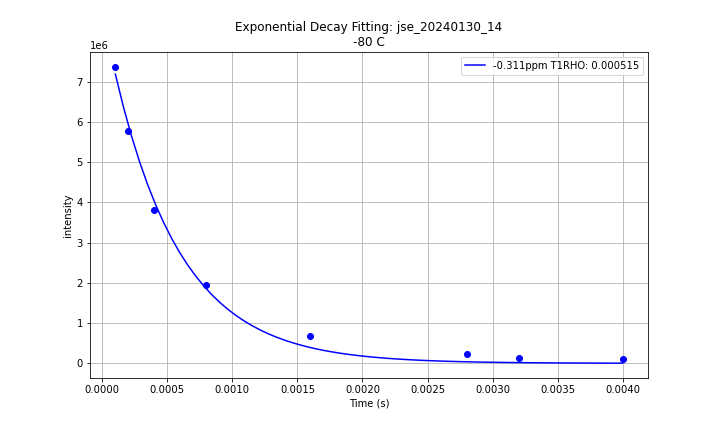

Supplement: Supplementary file 2 [file cg5c00666_si_002.zip › NMR/T1RHO/jse_T1RHO_14.png]

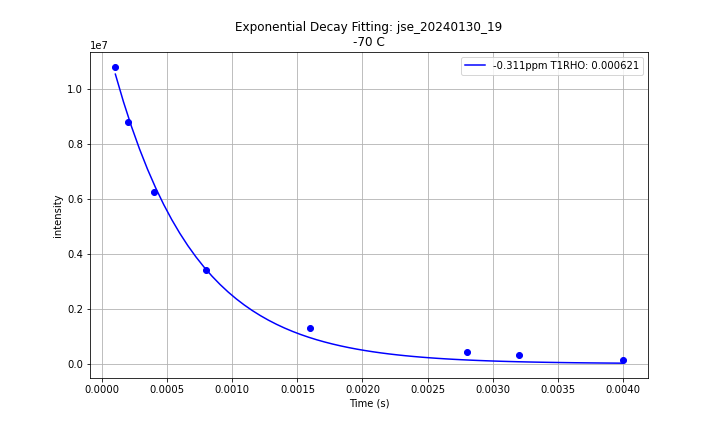

Supplement: Supplementary file 2 [file cg5c00666_si_002.zip › NMR/T1RHO/jse_T1RHO_19.png]

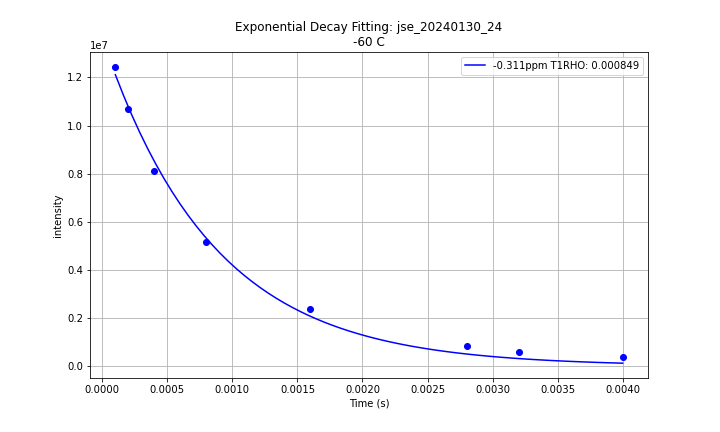

Supplement: Supplementary file 2 [file cg5c00666_si_002.zip › NMR/T1RHO/jse_T1RHO_24.png]

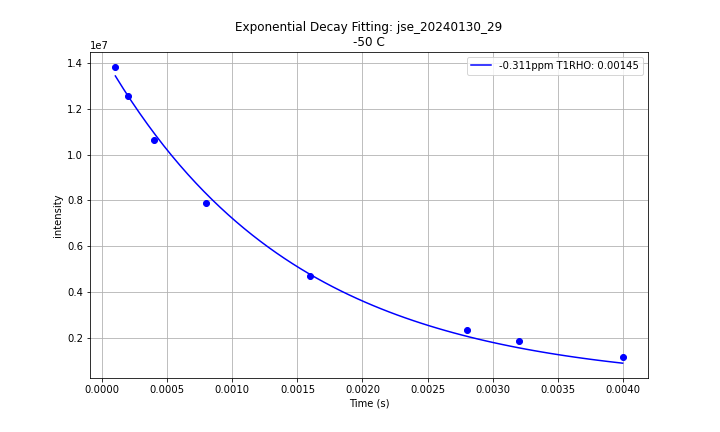

Supplement: Supplementary file 2 [file cg5c00666_si_002.zip › NMR/T1RHO/jse_T1RHO_29.png]

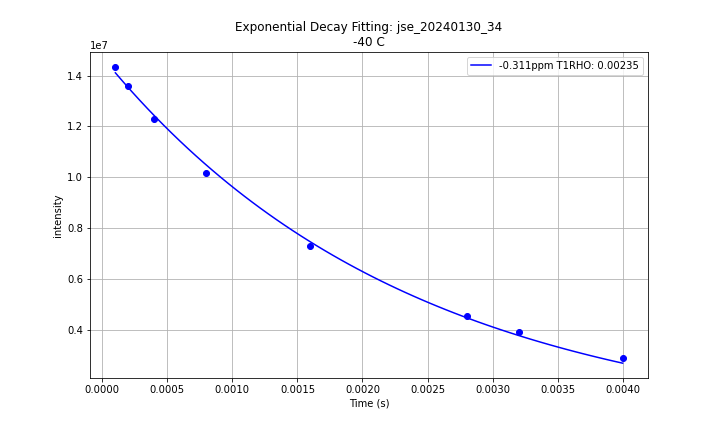

Supplement: Supplementary file 2 [file cg5c00666_si_002.zip › NMR/T1RHO/jse_T1RHO_34.png]

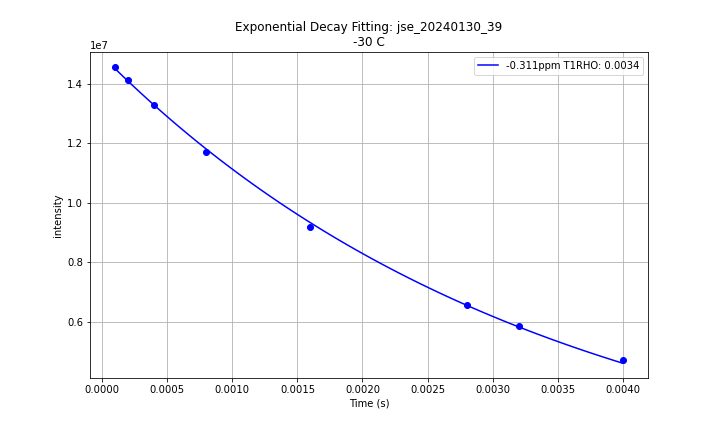

Supplement: Supplementary file 2 [file cg5c00666_si_002.zip › NMR/T1RHO/jse_T1RHO_39.png]

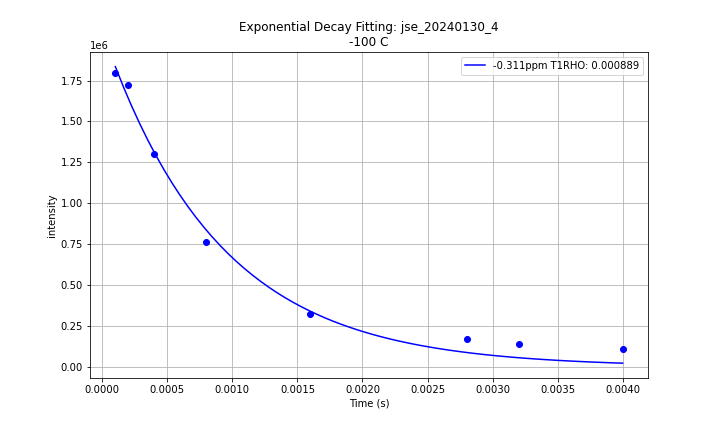

Supplement: Supplementary file 2 [file cg5c00666_si_002.zip › NMR/T1RHO/jse_T1RHO_4.png]

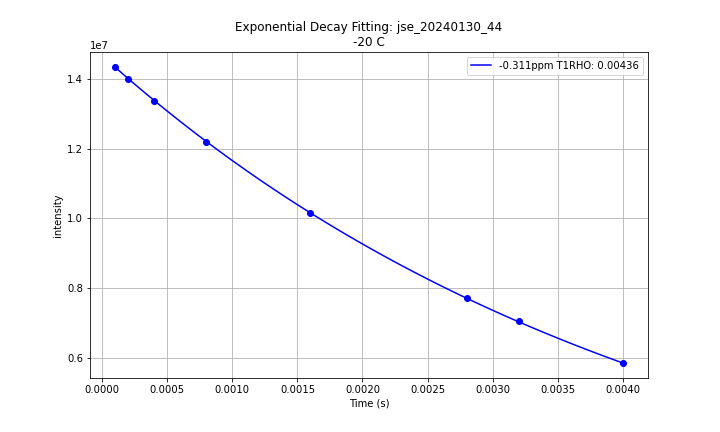

Supplement: Supplementary file 2 [file cg5c00666_si_002.zip › NMR/T1RHO/jse_T1RHO_44.png]

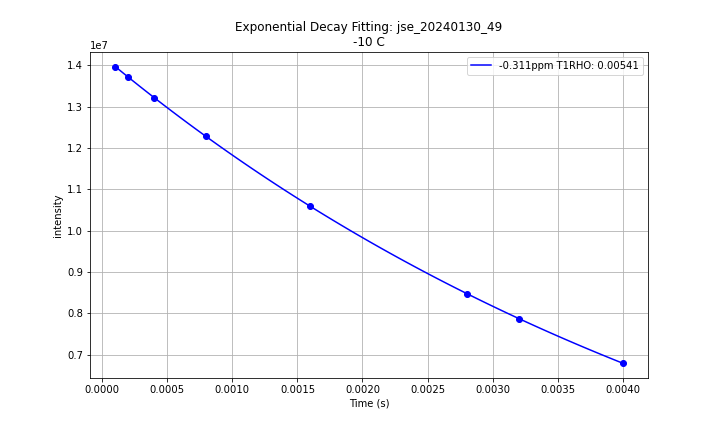

Supplement: Supplementary file 2 [file cg5c00666_si_002.zip › NMR/T1RHO/jse_T1RHO_49.png]

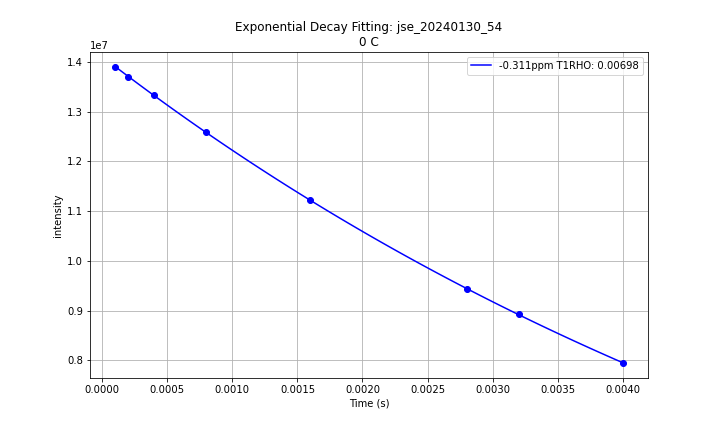

Supplement: Supplementary file 2 [file cg5c00666_si_002.zip › NMR/T1RHO/jse_T1RHO_54.png]

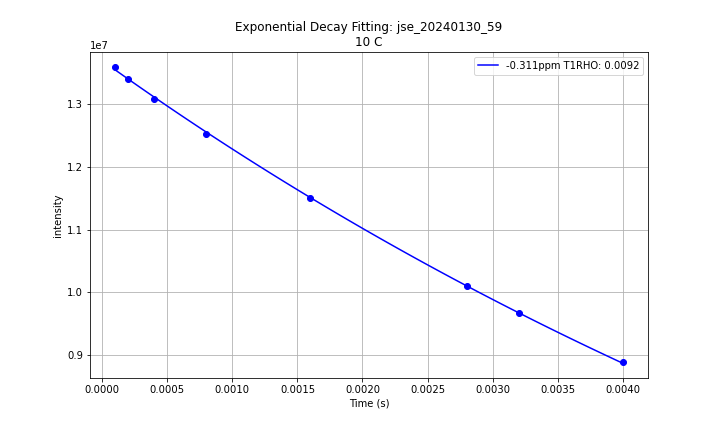

Supplement: Supplementary file 2 [file cg5c00666_si_002.zip › NMR/T1RHO/jse_T1RHO_59.png]

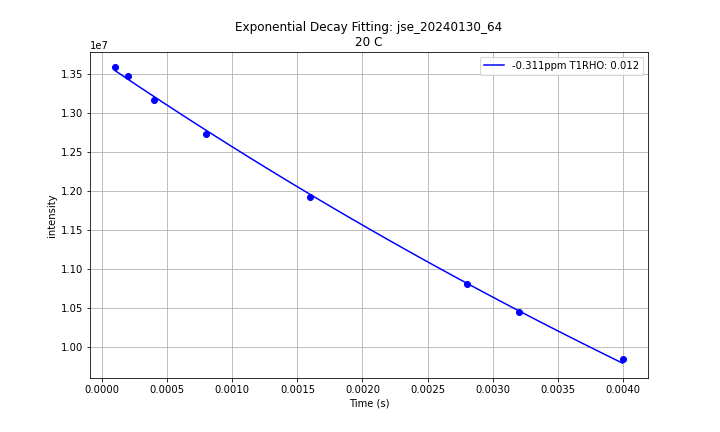

Supplement: Supplementary file 2 [file cg5c00666_si_002.zip › NMR/T1RHO/jse_T1RHO_64.png]

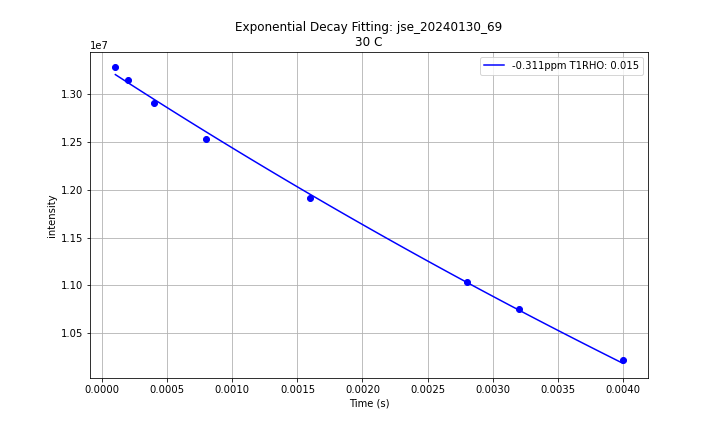

Supplement: Supplementary file 2 [file cg5c00666_si_002.zip › NMR/T1RHO/jse_T1RHO_69.png]

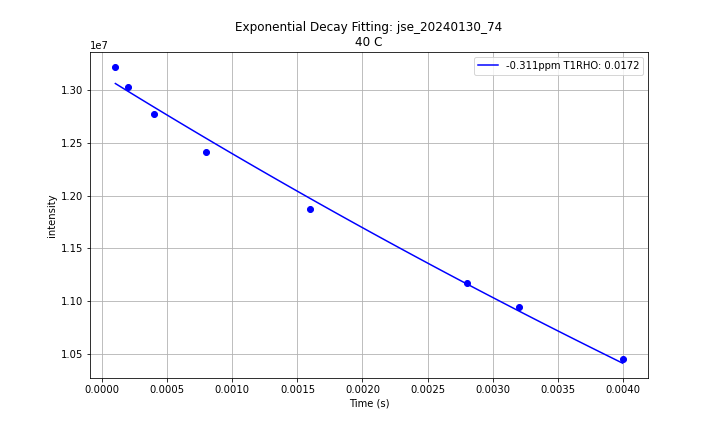

Supplement: Supplementary file 2 [file cg5c00666_si_002.zip › NMR/T1RHO/jse_T1RHO_74.png]

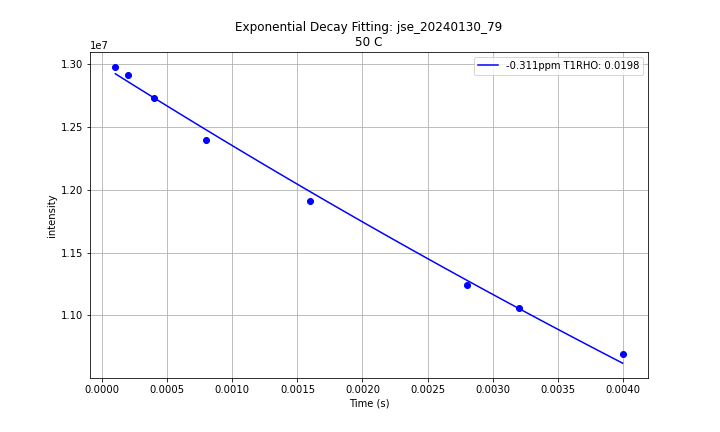

Supplement: Supplementary file 2 [file cg5c00666_si_002.zip › NMR/T1RHO/jse_T1RHO_79.png]

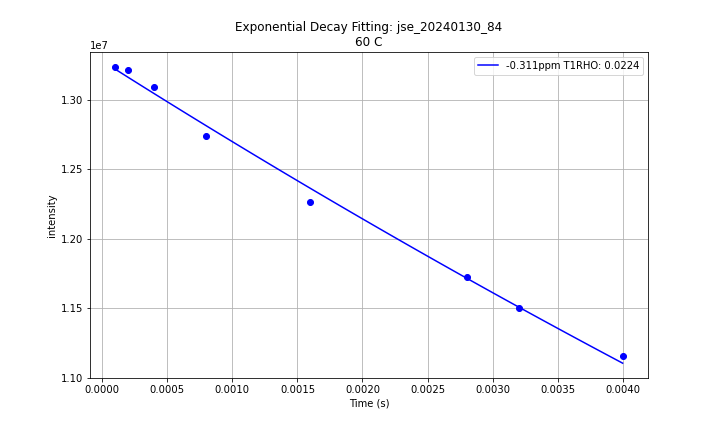

Supplement: Supplementary file 2 [file cg5c00666_si_002.zip › NMR/T1RHO/jse_T1RHO_84.png]

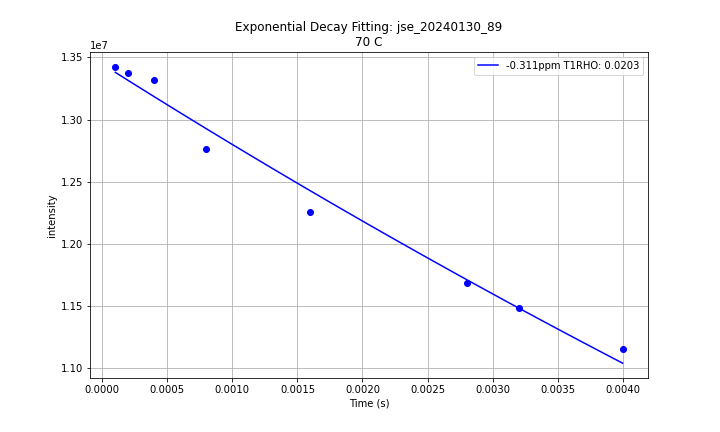

Supplement: Supplementary file 2 [file cg5c00666_si_002.zip › NMR/T1RHO/jse_T1RHO_89.png]

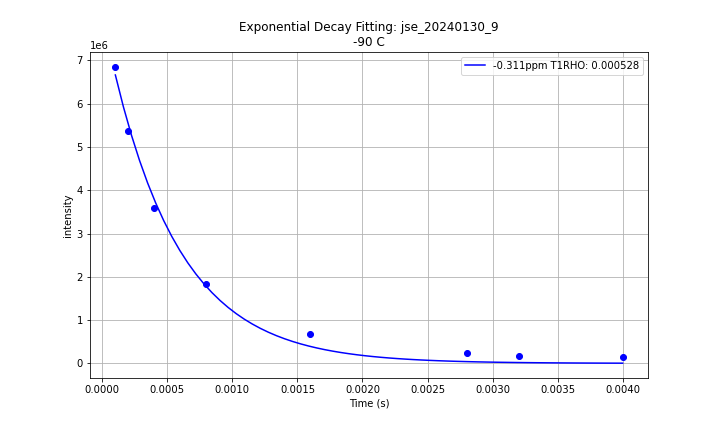

Supplement: Supplementary file 2 [file cg5c00666_si_002.zip › NMR/T1RHO/jse_T1RHO_9.png]

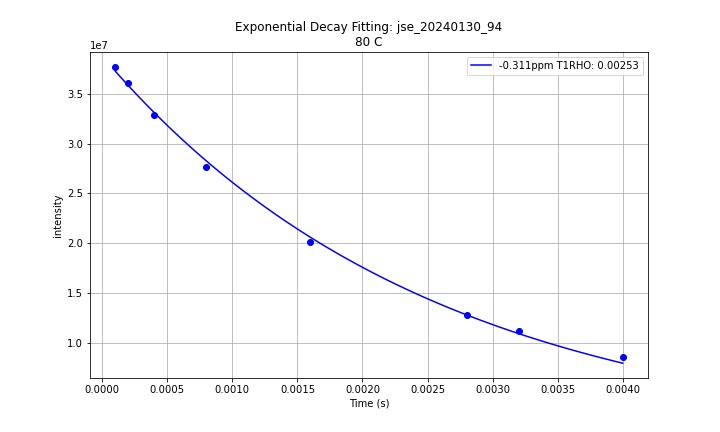

Supplement: Supplementary file 2 [file cg5c00666_si_002.zip › NMR/T1RHO/jse_T1RHO_94.png]

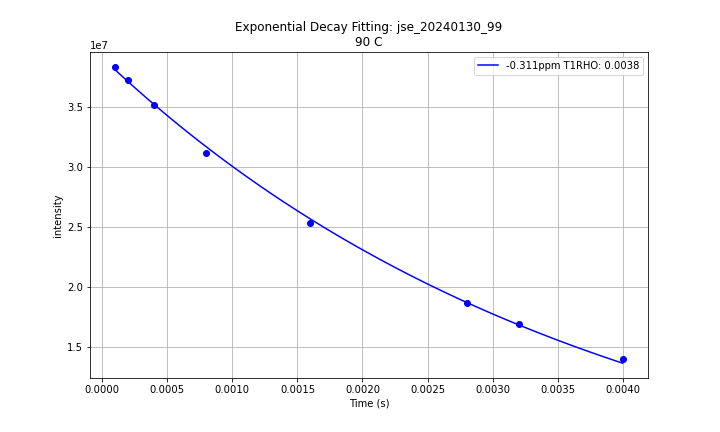

Supplement: Supplementary file 2 [file cg5c00666_si_002.zip › NMR/T1RHO/jse_T1RHO_99.png]
