# Supplementary figures and images for: Potential Multiaxial Molecular Ferroelectricity through Chiral Cation Replacement (part 1 of 2)
Source: Cryst Growth Des. 2025 Jul 21;25(15):6237–47. doi: 10.1021/acs.cgd.5c00666 (PMC12332970; doi:10.1021/acs.cgd.5c00666)

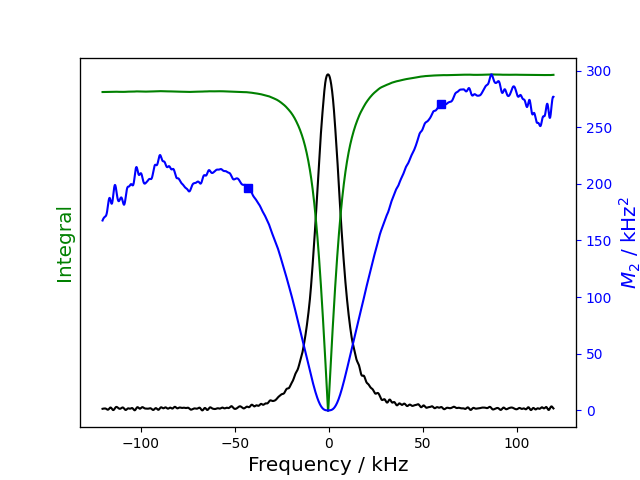

Supplement: Supplementary file 2 [file cg5c00666_si_002.zip › NMR/M2/0.txt.png]

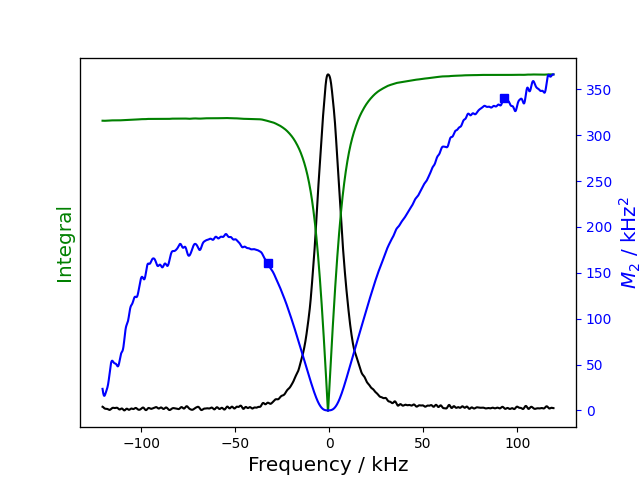

Supplement: Supplementary file 2 [file cg5c00666_si_002.zip › NMR/M2/10.txt.png]

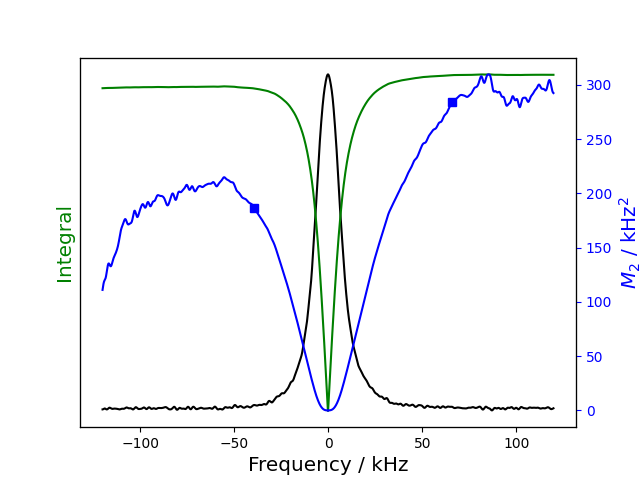

Supplement: Supplementary file 2 [file cg5c00666_si_002.zip › NMR/M2/-10.txt.png]

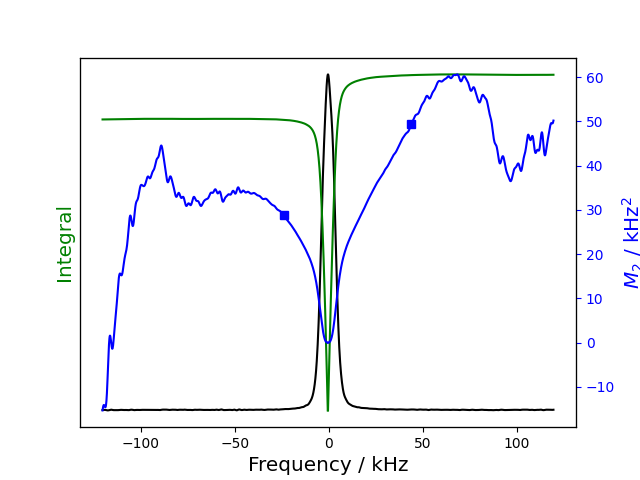

Supplement: Supplementary file 2 [file cg5c00666_si_002.zip › NMR/M2/100.txt.png]

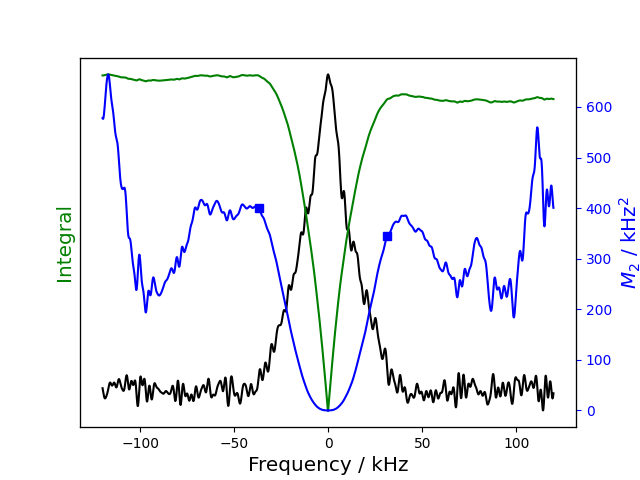

Supplement: Supplementary file 2 [file cg5c00666_si_002.zip › NMR/M2/-100.txt.png]

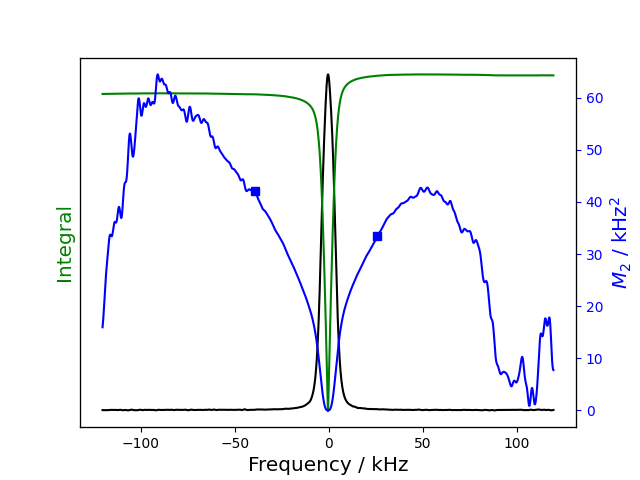

Supplement: Supplementary file 2 [file cg5c00666_si_002.zip › NMR/M2/110.txt.png]

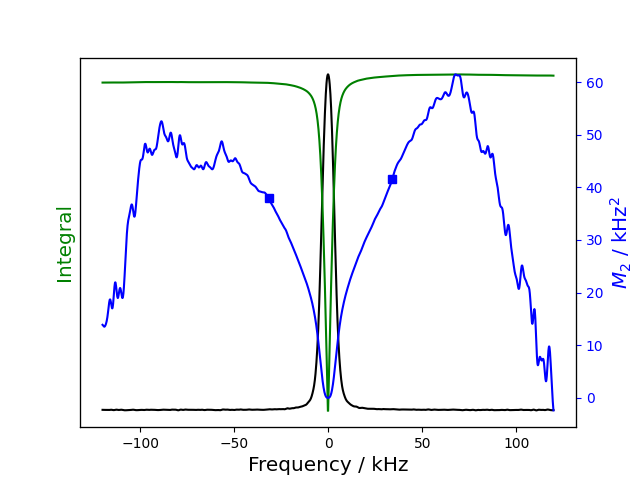

Supplement: Supplementary file 2 [file cg5c00666_si_002.zip › NMR/M2/120.txt.png]

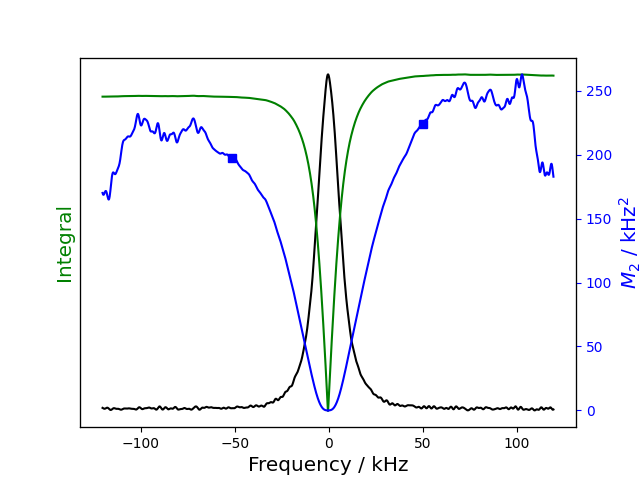

Supplement: Supplementary file 2 [file cg5c00666_si_002.zip › NMR/M2/20.txt.png]

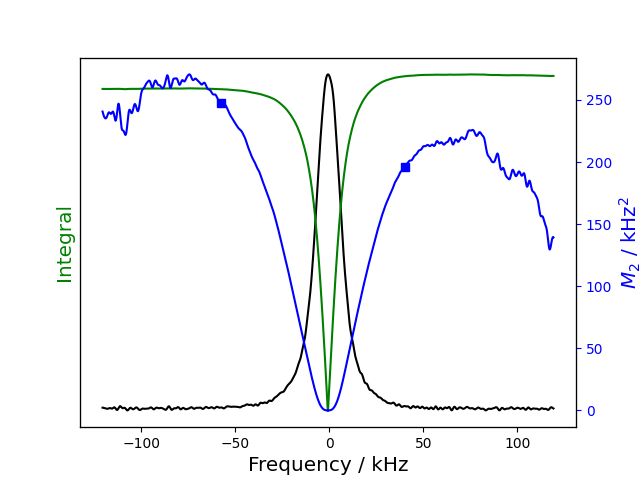

Supplement: Supplementary file 2 [file cg5c00666_si_002.zip › NMR/M2/-20.txt.png]

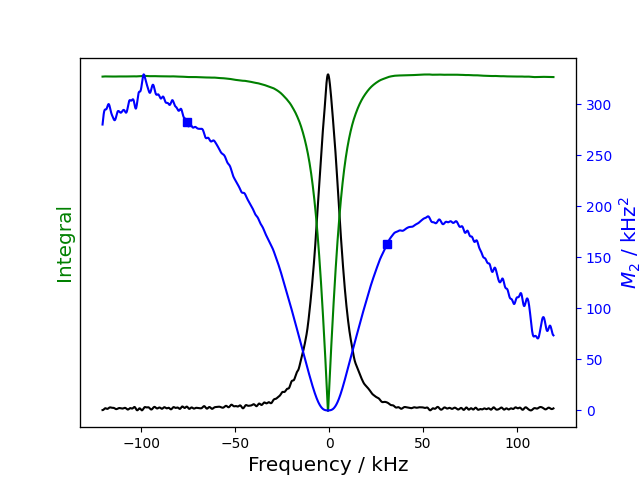

Supplement: Supplementary file 2 [file cg5c00666_si_002.zip › NMR/M2/30.txt.png]

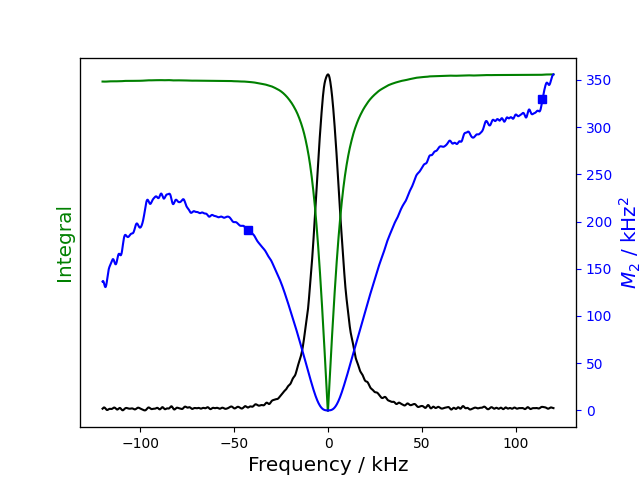

Supplement: Supplementary file 2 [file cg5c00666_si_002.zip › NMR/M2/-30.txt.png]

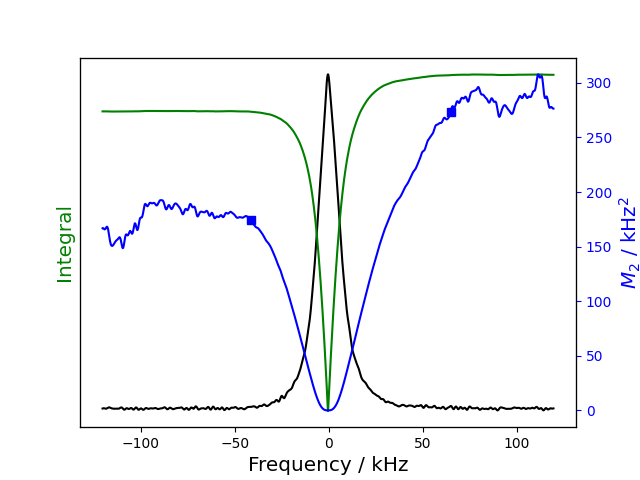

Supplement: Supplementary file 2 [file cg5c00666_si_002.zip › NMR/M2/40.txt.png]

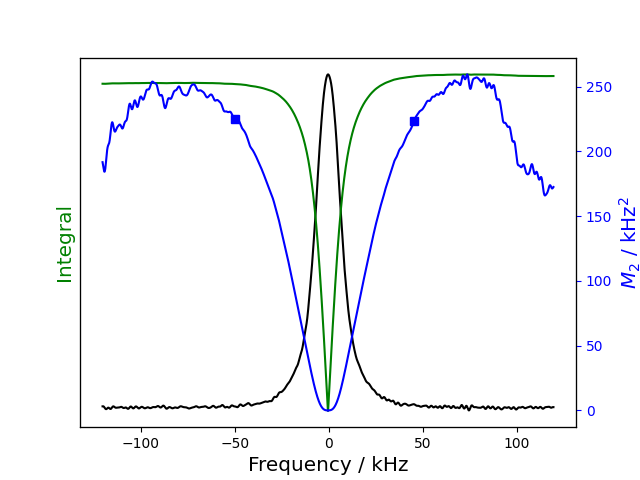

Supplement: Supplementary file 2 [file cg5c00666_si_002.zip › NMR/M2/-40.txt.png]

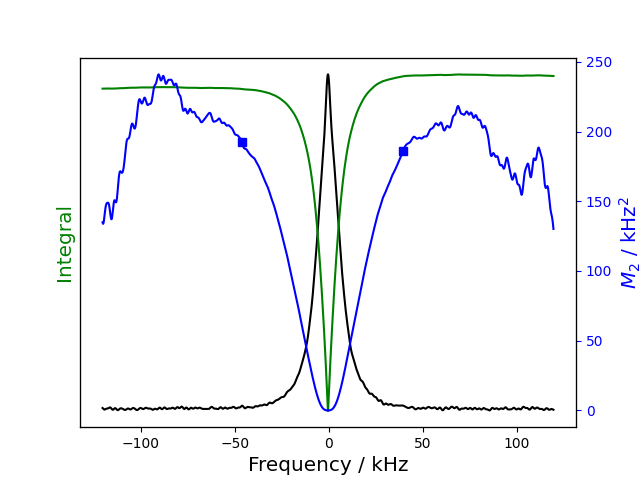

Supplement: Supplementary file 2 [file cg5c00666_si_002.zip › NMR/M2/50.txt.png]

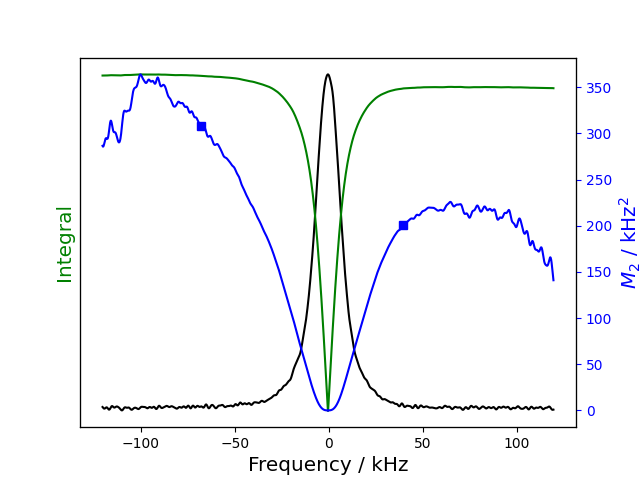

Supplement: Supplementary file 2 [file cg5c00666_si_002.zip › NMR/M2/-50.txt.png]

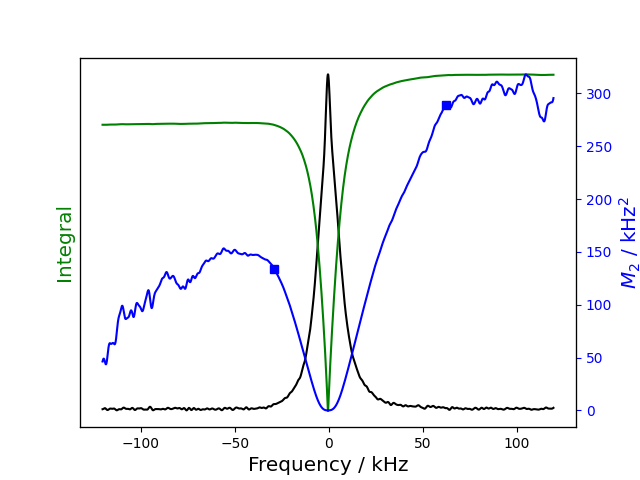

Supplement: Supplementary file 2 [file cg5c00666_si_002.zip › NMR/M2/60.txt.png]

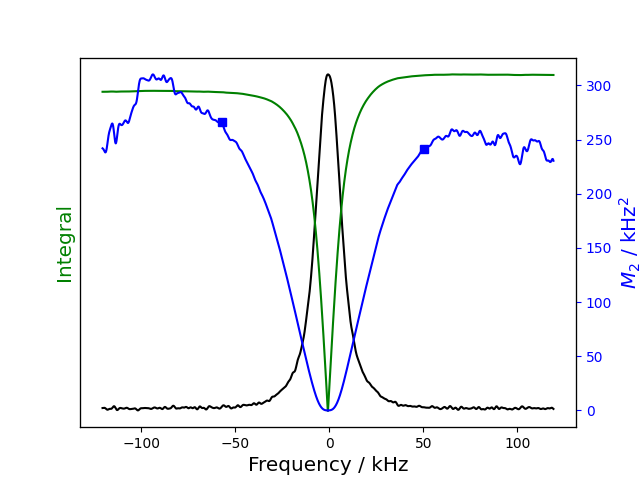

Supplement: Supplementary file 2 [file cg5c00666_si_002.zip › NMR/M2/-60.txt.png]

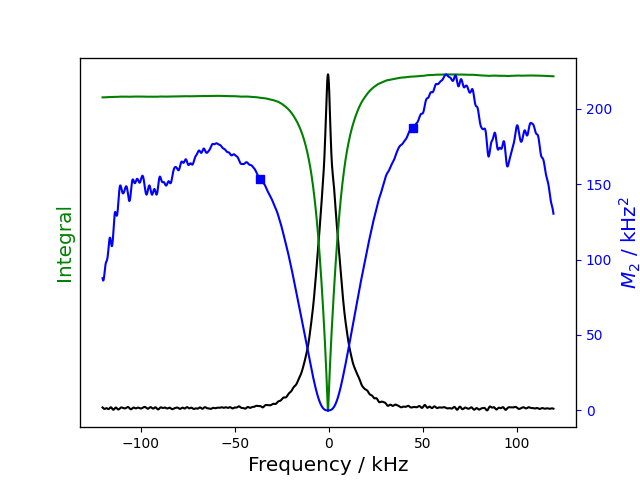

Supplement: Supplementary file 2 [file cg5c00666_si_002.zip › NMR/M2/70.txt.png]

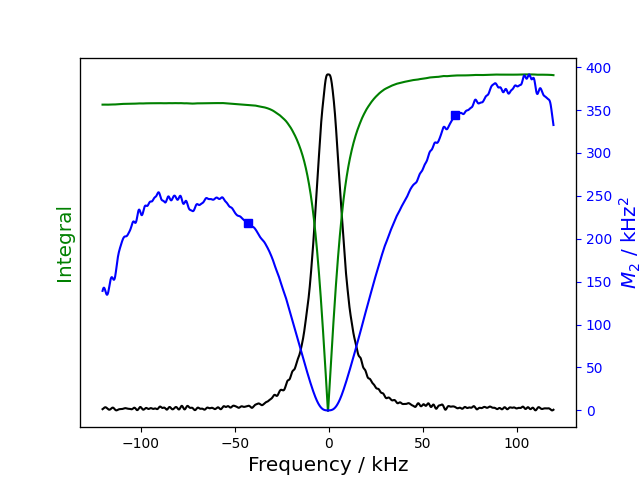

Supplement: Supplementary file 2 [file cg5c00666_si_002.zip › NMR/M2/-70.txt.png]

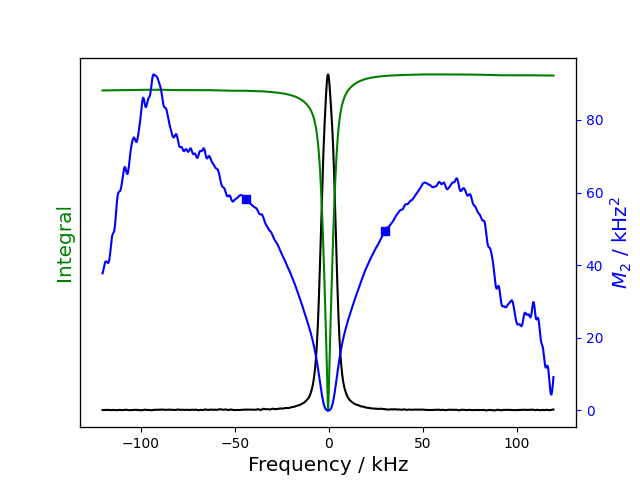

Supplement: Supplementary file 2 [file cg5c00666_si_002.zip › NMR/M2/80.txt.png]

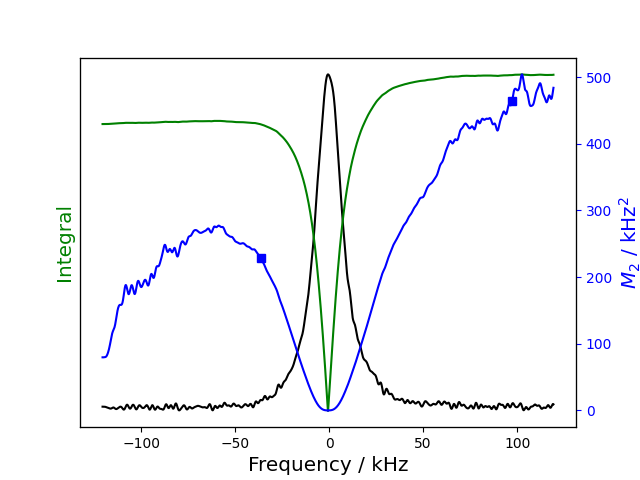

Supplement: Supplementary file 2 [file cg5c00666_si_002.zip › NMR/M2/-80.txt.png]

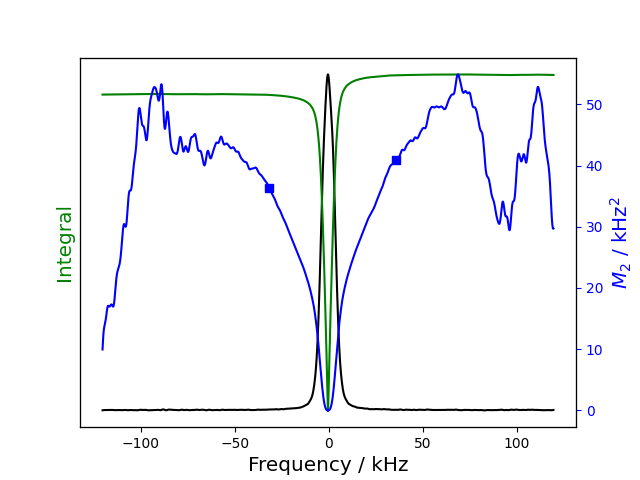

Supplement: Supplementary file 2 [file cg5c00666_si_002.zip › NMR/M2/90.txt.png]

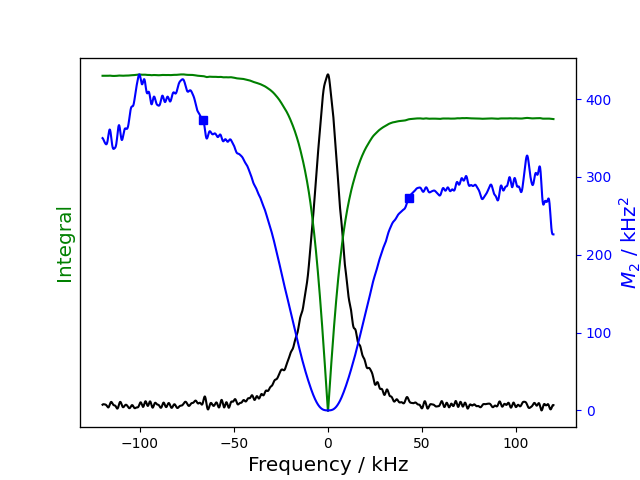

Supplement: Supplementary file 2 [file cg5c00666_si_002.zip › NMR/M2/-90.txt.png]

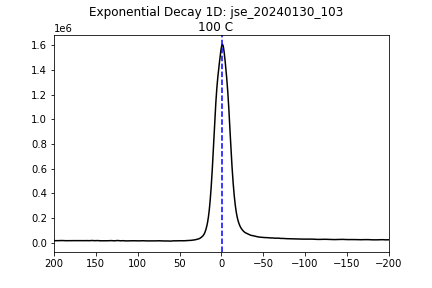

Supplement: Supplementary file 2 [file cg5c00666_si_002.zip › NMR/T1/jse_20240130_103_figure.png]

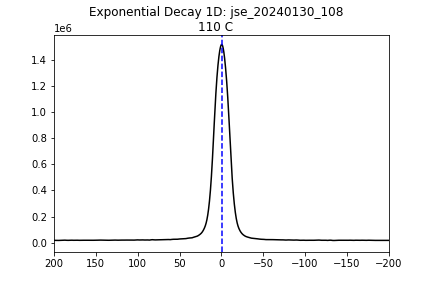

Supplement: Supplementary file 2 [file cg5c00666_si_002.zip › NMR/T1/jse_20240130_108_figure.png]

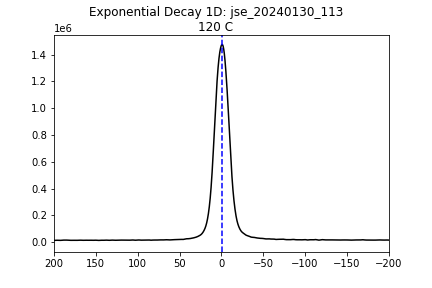

Supplement: Supplementary file 2 [file cg5c00666_si_002.zip › NMR/T1/jse_20240130_113_figure.png]

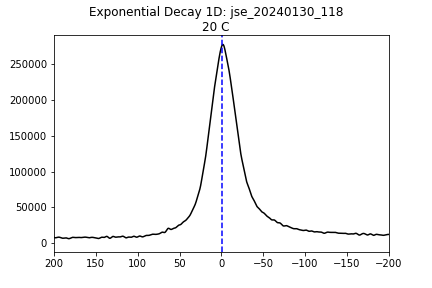

Supplement: Supplementary file 2 [file cg5c00666_si_002.zip › NMR/T1/jse_20240130_118_figure.png]

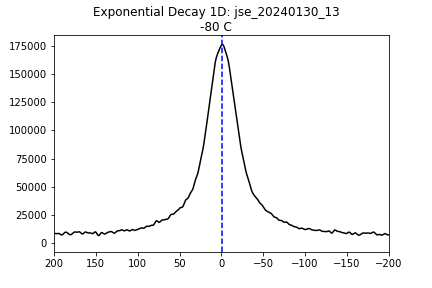

Supplement: Supplementary file 2 [file cg5c00666_si_002.zip › NMR/T1/jse_20240130_13_figure.png]

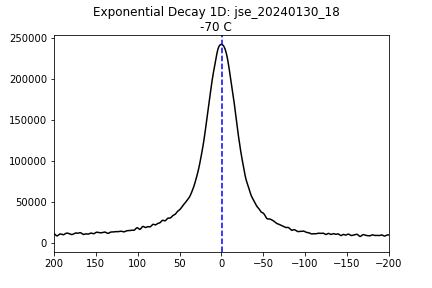

Supplement: Supplementary file 2 [file cg5c00666_si_002.zip › NMR/T1/jse_20240130_18_figure.png]

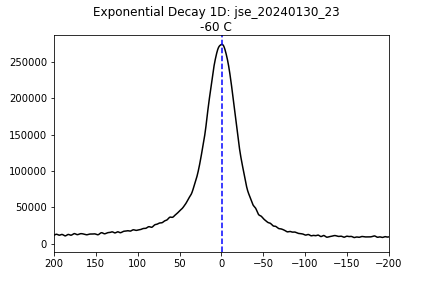

Supplement: Supplementary file 2 [file cg5c00666_si_002.zip › NMR/T1/jse_20240130_23_figure.png]

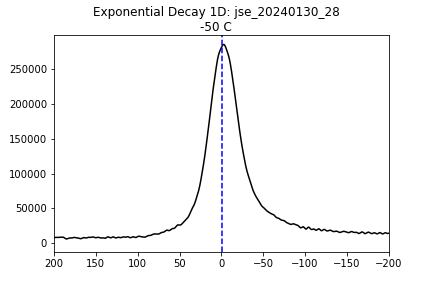

Supplement: Supplementary file 2 [file cg5c00666_si_002.zip › NMR/T1/jse_20240130_28_figure.png]

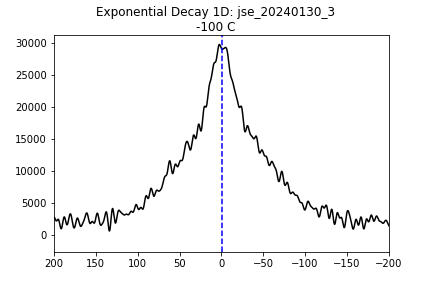

Supplement: Supplementary file 2 [file cg5c00666_si_002.zip › NMR/T1/jse_20240130_3_figure.png]

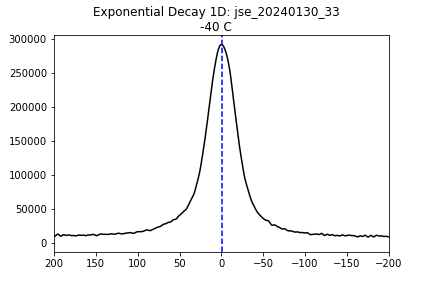

Supplement: Supplementary file 2 [file cg5c00666_si_002.zip › NMR/T1/jse_20240130_33_figure.png]

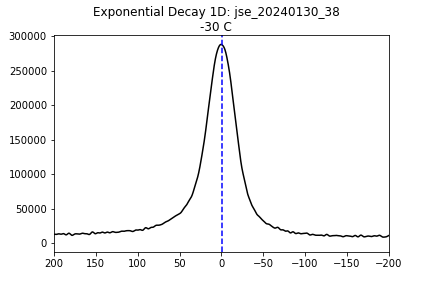

Supplement: Supplementary file 2 [file cg5c00666_si_002.zip › NMR/T1/jse_20240130_38_figure.png]

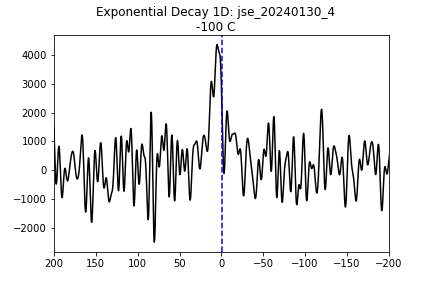

Supplement: Supplementary file 2 [file cg5c00666_si_002.zip › NMR/T1/jse_20240130_4_figure.png]

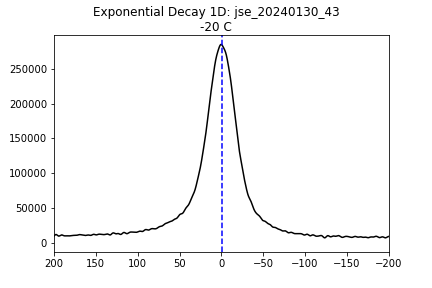

Supplement: Supplementary file 2 [file cg5c00666_si_002.zip › NMR/T1/jse_20240130_43_figure.png]

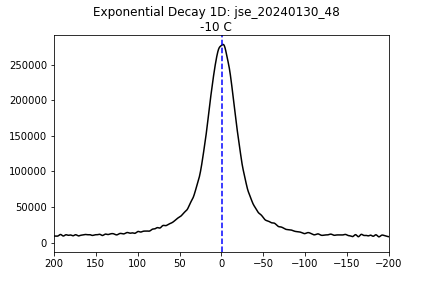

Supplement: Supplementary file 2 [file cg5c00666_si_002.zip › NMR/T1/jse_20240130_48_figure.png]

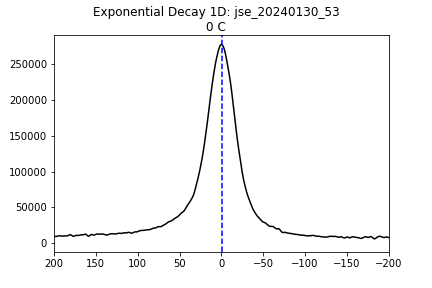

Supplement: Supplementary file 2 [file cg5c00666_si_002.zip › NMR/T1/jse_20240130_53_figure.png]

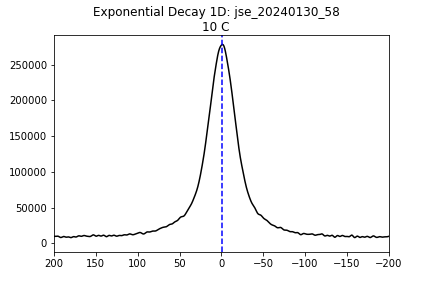

Supplement: Supplementary file 2 [file cg5c00666_si_002.zip › NMR/T1/jse_20240130_58_figure.png]

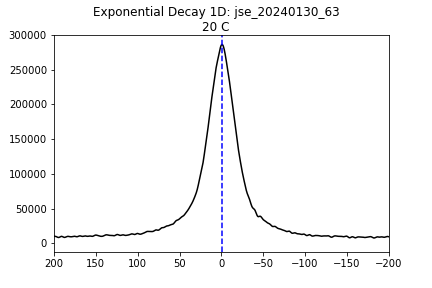

Supplement: Supplementary file 2 [file cg5c00666_si_002.zip › NMR/T1/jse_20240130_63_figure.png]

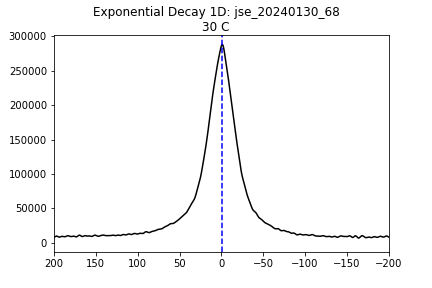

Supplement: Supplementary file 2 [file cg5c00666_si_002.zip › NMR/T1/jse_20240130_68_figure.png]

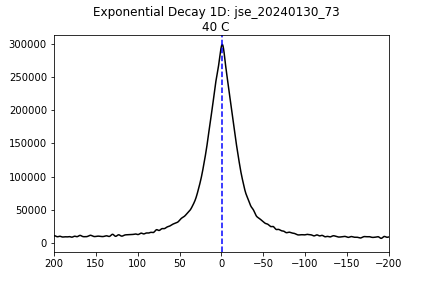

Supplement: Supplementary file 2 [file cg5c00666_si_002.zip › NMR/T1/jse_20240130_73_figure.png]

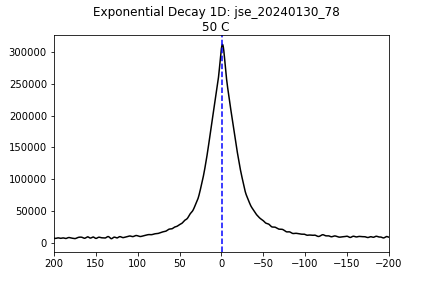

Supplement: Supplementary file 2 [file cg5c00666_si_002.zip › NMR/T1/jse_20240130_78_figure.png]

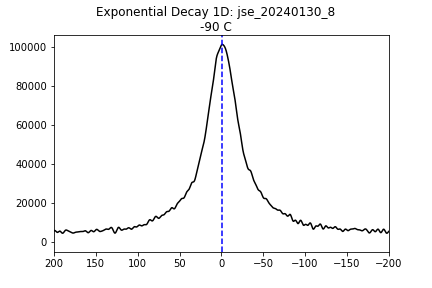

Supplement: Supplementary file 2 [file cg5c00666_si_002.zip › NMR/T1/jse_20240130_8_figure.png]

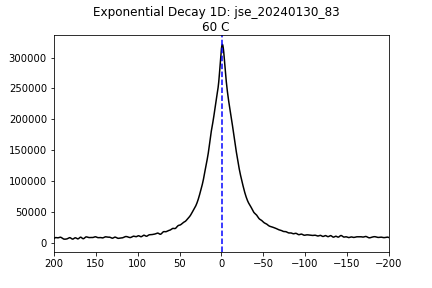

Supplement: Supplementary file 2 [file cg5c00666_si_002.zip › NMR/T1/jse_20240130_83_figure.png]

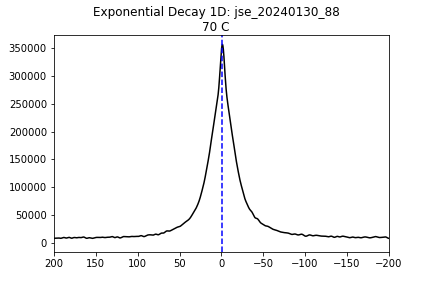

Supplement: Supplementary file 2 [file cg5c00666_si_002.zip › NMR/T1/jse_20240130_88_figure.png]

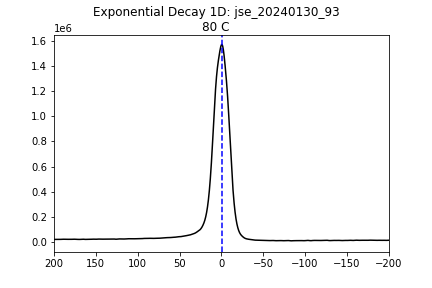

Supplement: Supplementary file 2 [file cg5c00666_si_002.zip › NMR/T1/jse_20240130_93_figure.png]

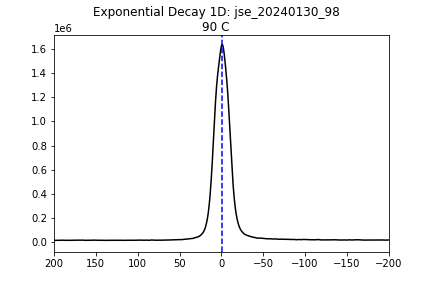

Supplement: Supplementary file 2 [file cg5c00666_si_002.zip › NMR/T1/jse_20240130_98_figure.png]

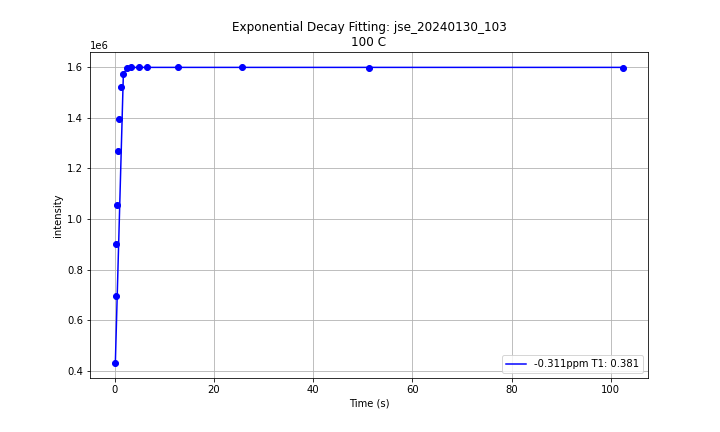

Supplement: Supplementary file 2 [file cg5c00666_si_002.zip › NMR/T1/jse_T1_103.png]

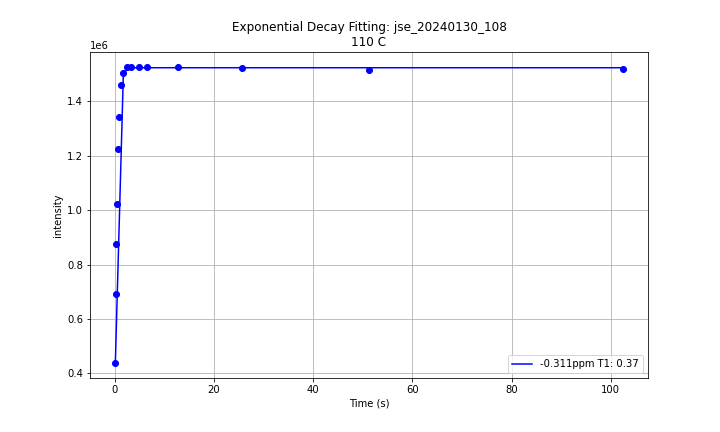

Supplement: Supplementary file 2 [file cg5c00666_si_002.zip › NMR/T1/jse_T1_108.png]

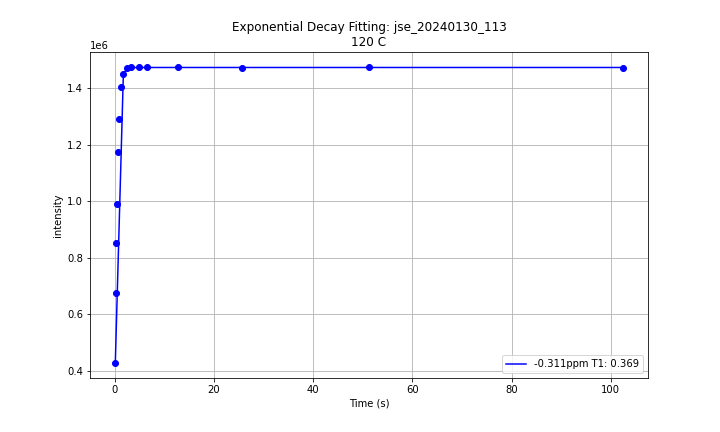

Supplement: Supplementary file 2 [file cg5c00666_si_002.zip › NMR/T1/jse_T1_113.png]

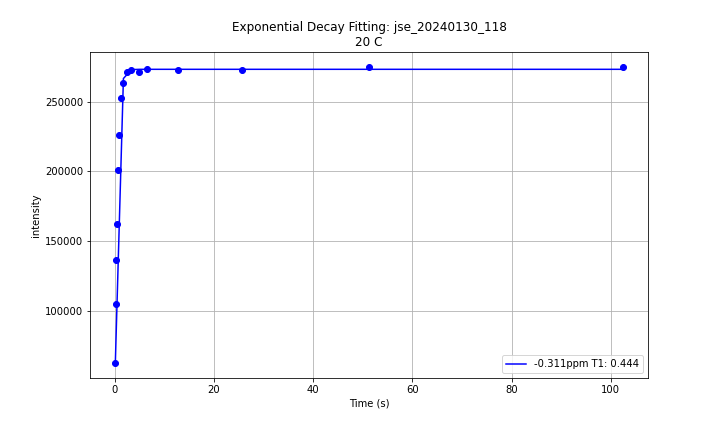

Supplement: Supplementary file 2 [file cg5c00666_si_002.zip › NMR/T1/jse_T1_118.png]

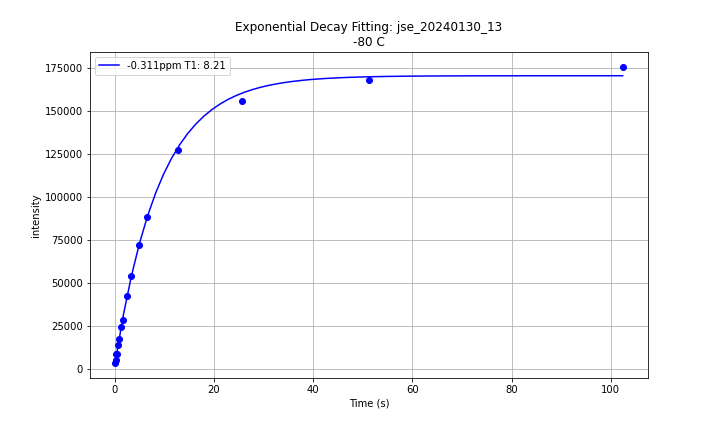

Supplement: Supplementary file 2 [file cg5c00666_si_002.zip › NMR/T1/jse_T1_13.png]

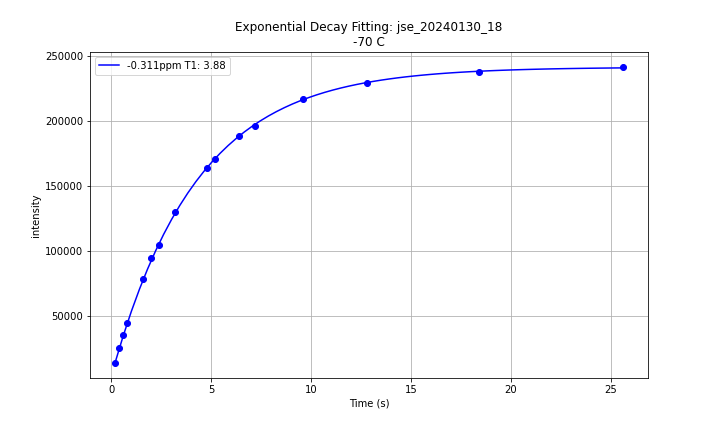

Supplement: Supplementary file 2 [file cg5c00666_si_002.zip › NMR/T1/jse_T1_18.png]

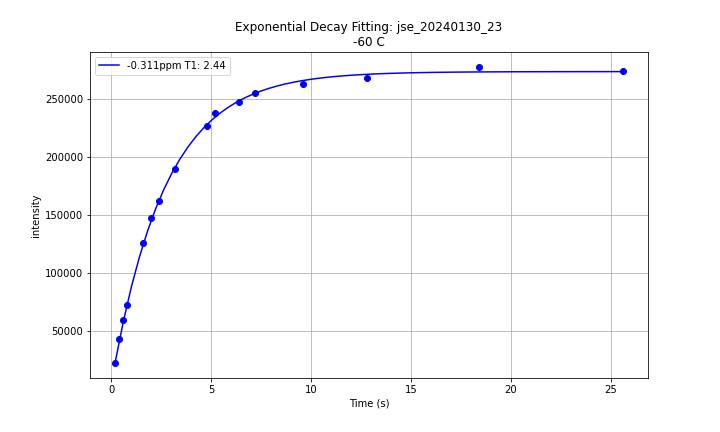

Supplement: Supplementary file 2 [file cg5c00666_si_002.zip › NMR/T1/jse_T1_23.png]

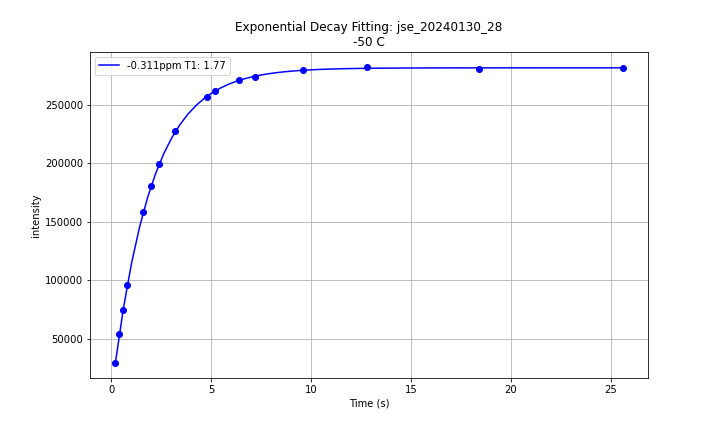

Supplement: Supplementary file 2 [file cg5c00666_si_002.zip › NMR/T1/jse_T1_28.png]

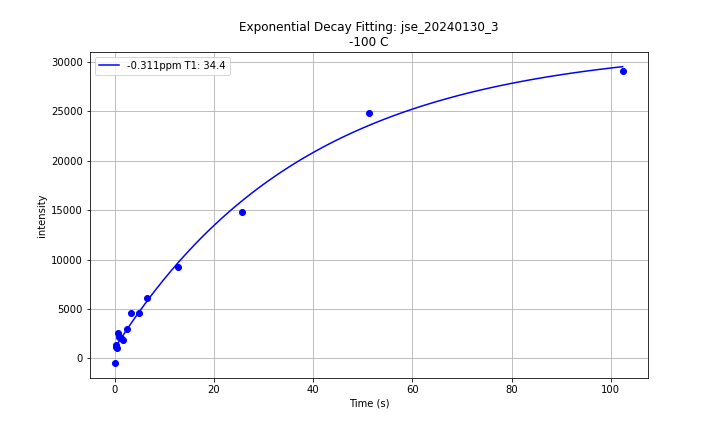

Supplement: Supplementary file 2 [file cg5c00666_si_002.zip › NMR/T1/jse_T1_3.png]

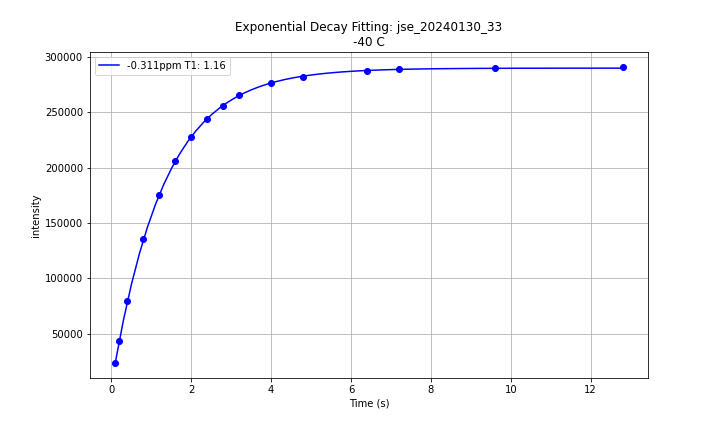

Supplement: Supplementary file 2 [file cg5c00666_si_002.zip › NMR/T1/jse_T1_33.png]

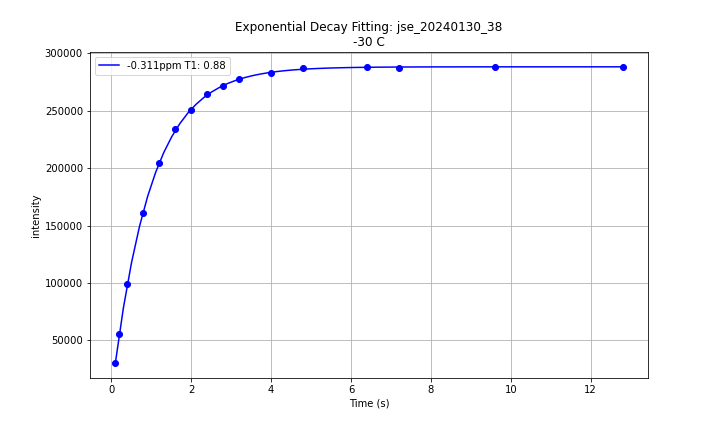

Supplement: Supplementary file 2 [file cg5c00666_si_002.zip › NMR/T1/jse_T1_38.png]

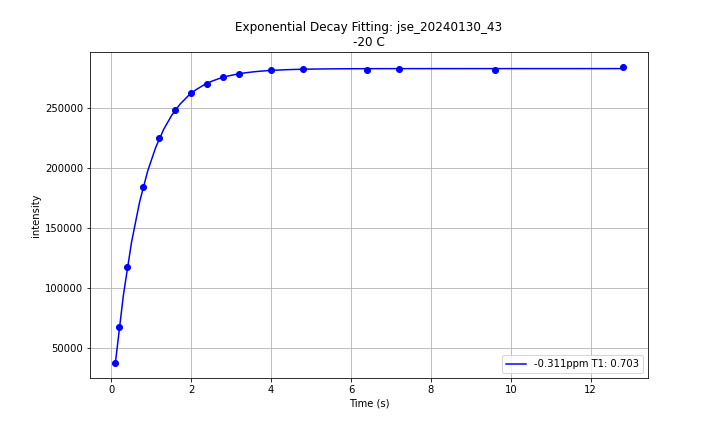

Supplement: Supplementary file 2 [file cg5c00666_si_002.zip › NMR/T1/jse_T1_43.png]

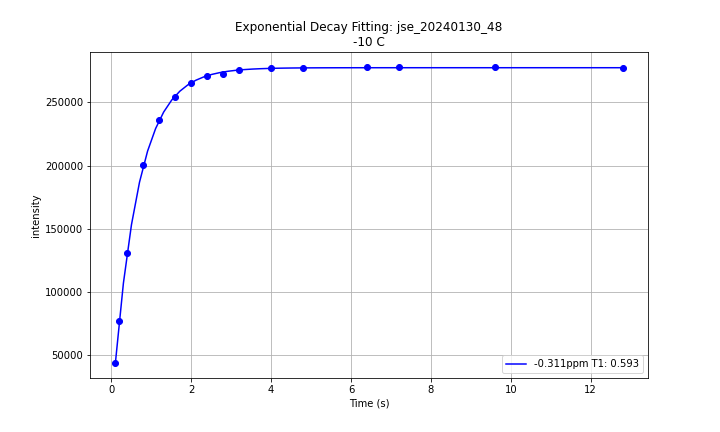

Supplement: Supplementary file 2 [file cg5c00666_si_002.zip › NMR/T1/jse_T1_48.png]

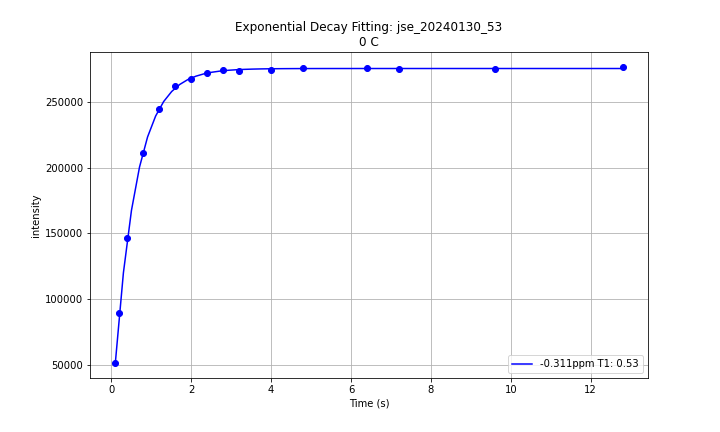

Supplement: Supplementary file 2 [file cg5c00666_si_002.zip › NMR/T1/jse_T1_53.png]

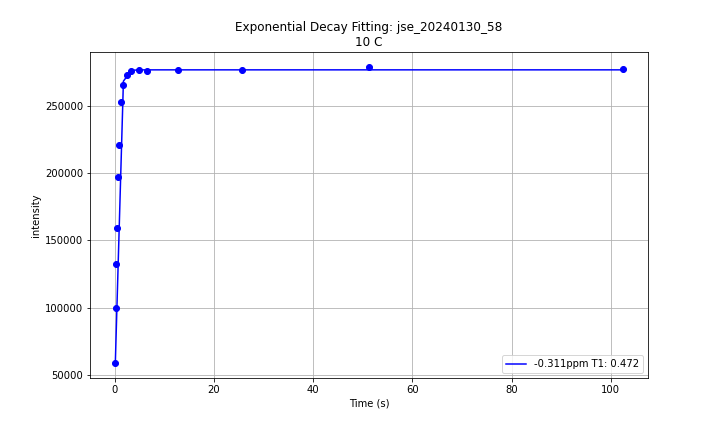

Supplement: Supplementary file 2 [file cg5c00666_si_002.zip › NMR/T1/jse_T1_58.png]

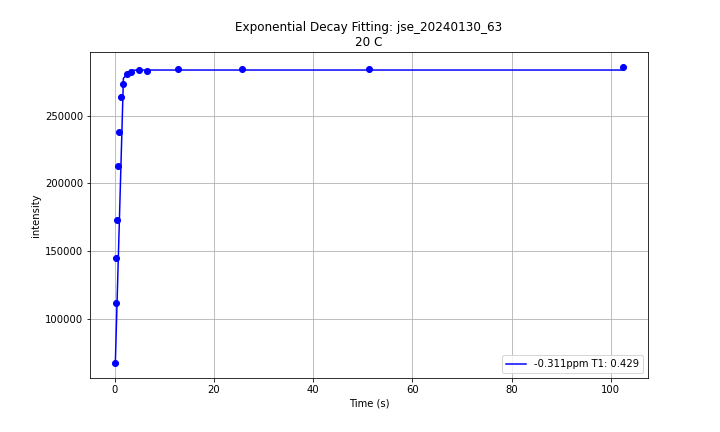

Supplement: Supplementary file 2 [file cg5c00666_si_002.zip › NMR/T1/jse_T1_63.png]

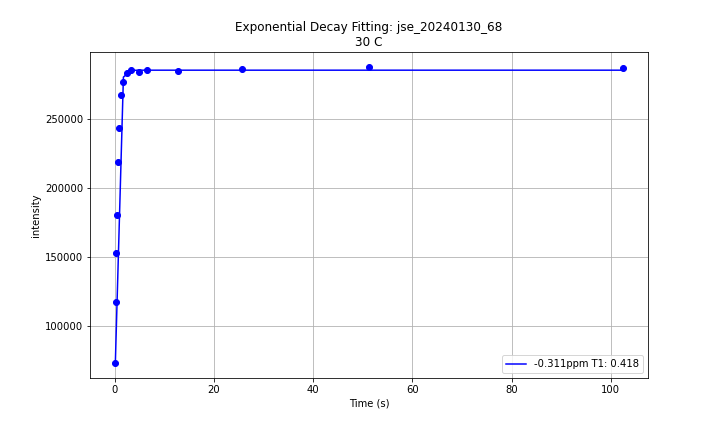

Supplement: Supplementary file 2 [file cg5c00666_si_002.zip › NMR/T1/jse_T1_68.png]

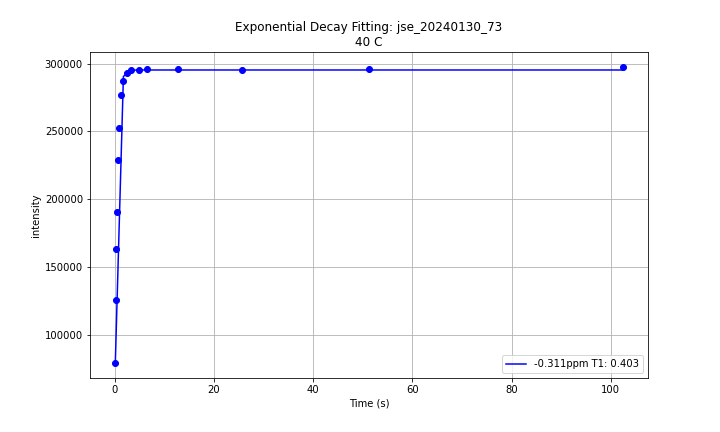

Supplement: Supplementary file 2 [file cg5c00666_si_002.zip › NMR/T1/jse_T1_73.png]

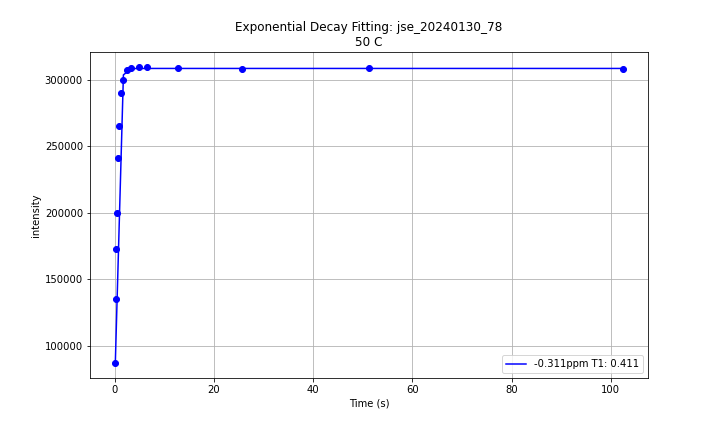

Supplement: Supplementary file 2 [file cg5c00666_si_002.zip › NMR/T1/jse_T1_78.png]

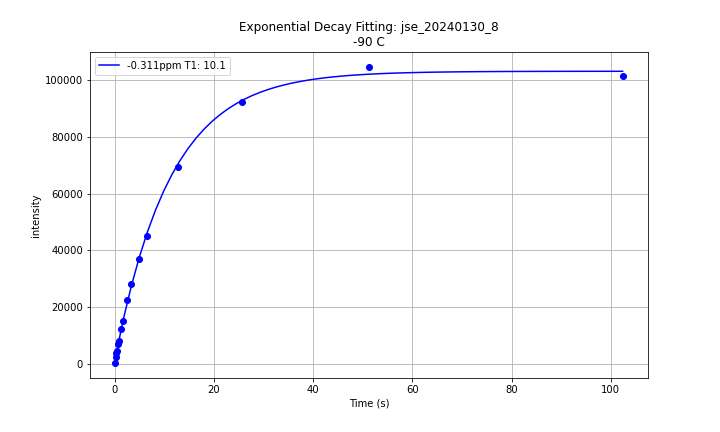

Supplement: Supplementary file 2 [file cg5c00666_si_002.zip › NMR/T1/jse_T1_8.png]

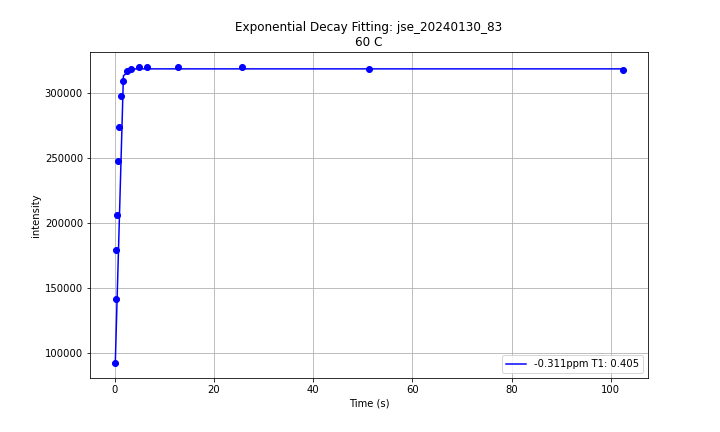

Supplement: Supplementary file 2 [file cg5c00666_si_002.zip › NMR/T1/jse_T1_83.png]

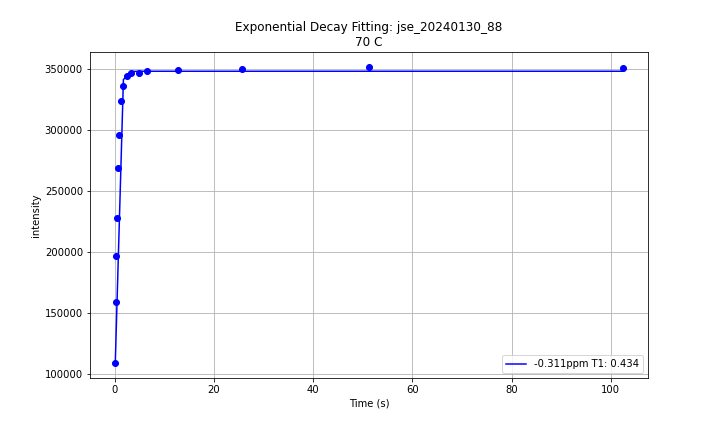

Supplement: Supplementary file 2 [file cg5c00666_si_002.zip › NMR/T1/jse_T1_88.png]

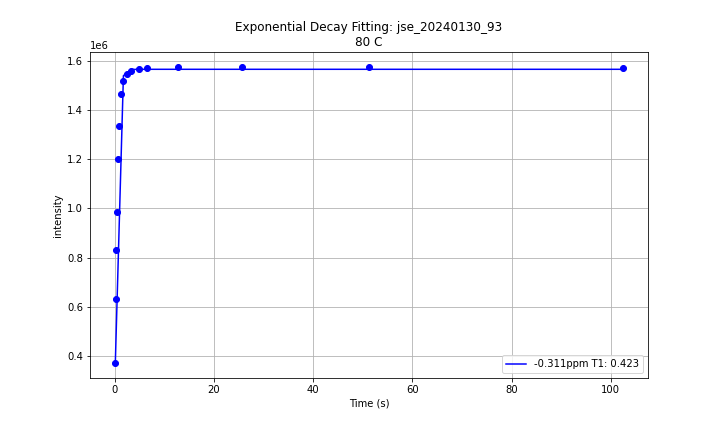

Supplement: Supplementary file 2 [file cg5c00666_si_002.zip › NMR/T1/jse_T1_93.png]

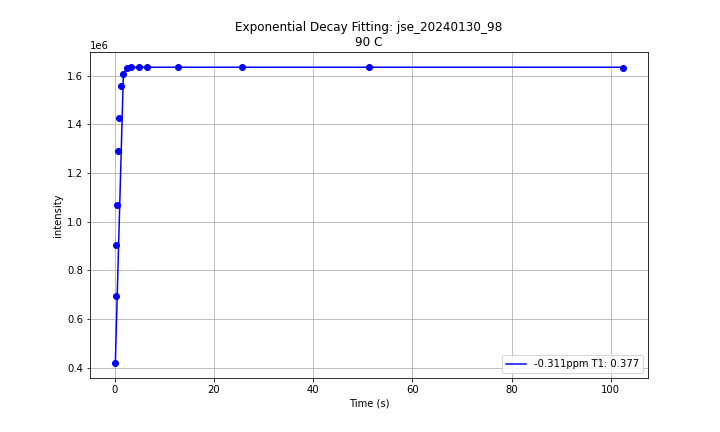

Supplement: Supplementary file 2 [file cg5c00666_si_002.zip › NMR/T1/jse_T1_98.png]

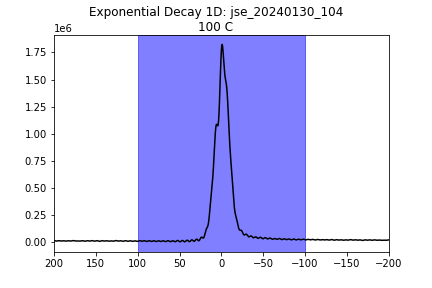

Supplement: Supplementary file 2 [file cg5c00666_si_002.zip › NMR/T1RHO/jse_20240130_104_figure.png]

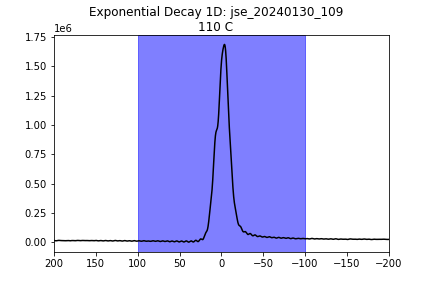

Supplement: Supplementary file 2 [file cg5c00666_si_002.zip › NMR/T1RHO/jse_20240130_109_figure.png]

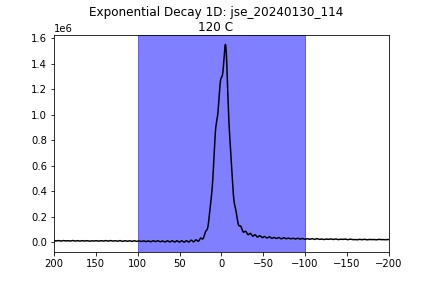

Supplement: Supplementary file 2 [file cg5c00666_si_002.zip › NMR/T1RHO/jse_20240130_114_figure.png]

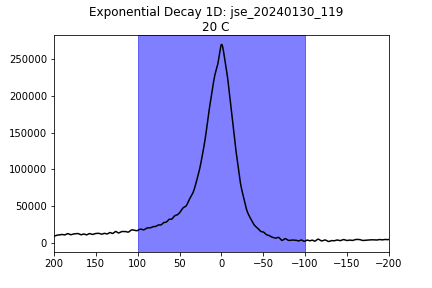

Supplement: Supplementary file 2 [file cg5c00666_si_002.zip › NMR/T1RHO/jse_20240130_119_figure.png]

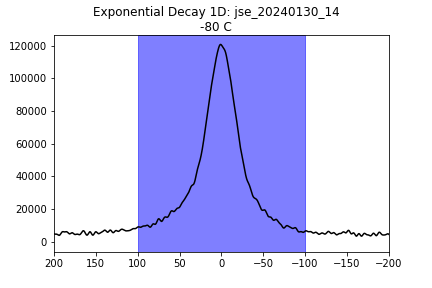

Supplement: Supplementary file 2 [file cg5c00666_si_002.zip › NMR/T1RHO/jse_20240130_14_figure.png]

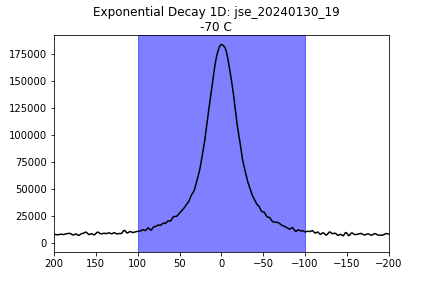

Supplement: Supplementary file 2 [file cg5c00666_si_002.zip › NMR/T1RHO/jse_20240130_19_figure.png]

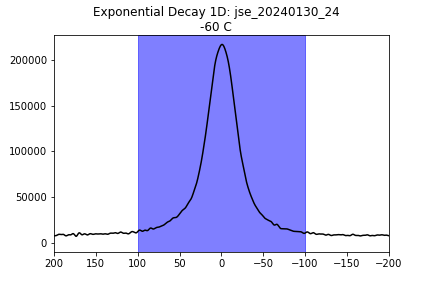

Supplement: Supplementary file 2 [file cg5c00666_si_002.zip › NMR/T1RHO/jse_20240130_24_figure.png]

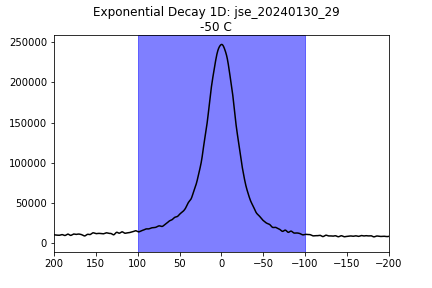

Supplement: Supplementary file 2 [file cg5c00666_si_002.zip › NMR/T1RHO/jse_20240130_29_figure.png]

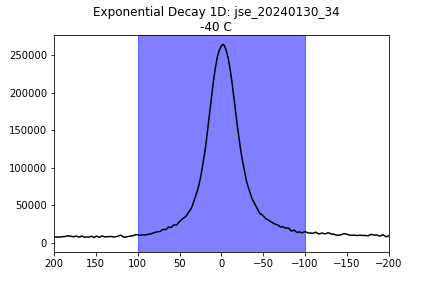

Supplement: Supplementary file 2 [file cg5c00666_si_002.zip › NMR/T1RHO/jse_20240130_34_figure.png]

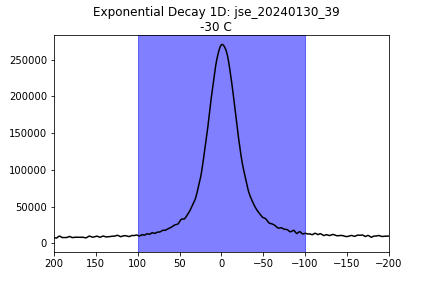

Supplement: Supplementary file 2 [file cg5c00666_si_002.zip › NMR/T1RHO/jse_20240130_39_figure.png]

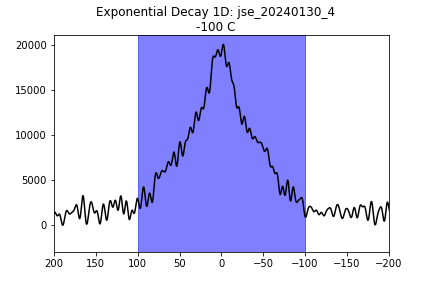

Supplement: Supplementary file 2 [file cg5c00666_si_002.zip › NMR/T1RHO/jse_20240130_4_figure.png]

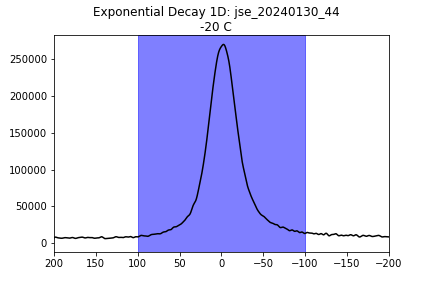

Supplement: Supplementary file 2 [file cg5c00666_si_002.zip › NMR/T1RHO/jse_20240130_44_figure.png]

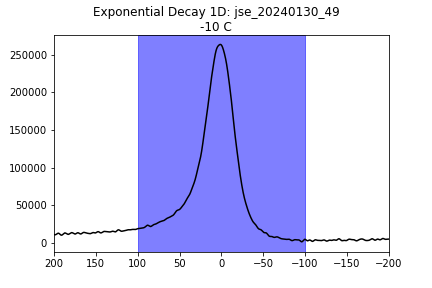

Supplement: Supplementary file 2 [file cg5c00666_si_002.zip › NMR/T1RHO/jse_20240130_49_figure.png]

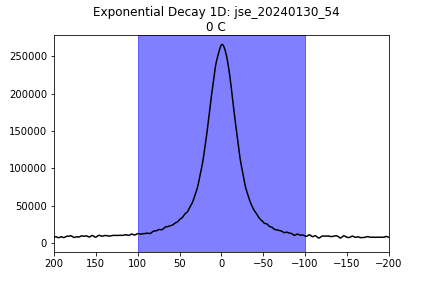

Supplement: Supplementary file 2 [file cg5c00666_si_002.zip › NMR/T1RHO/jse_20240130_54_figure.png]

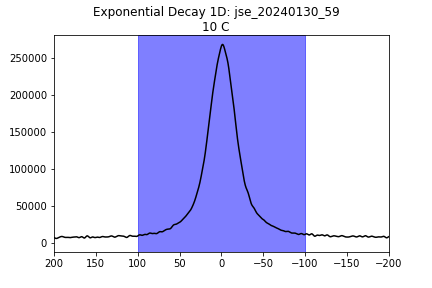

Supplement: Supplementary file 2 [file cg5c00666_si_002.zip › NMR/T1RHO/jse_20240130_59_figure.png]

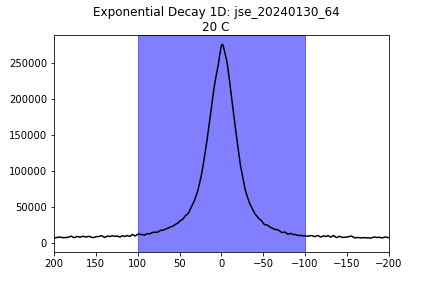

Supplement: Supplementary file 2 [file cg5c00666_si_002.zip › NMR/T1RHO/jse_20240130_64_figure.png]

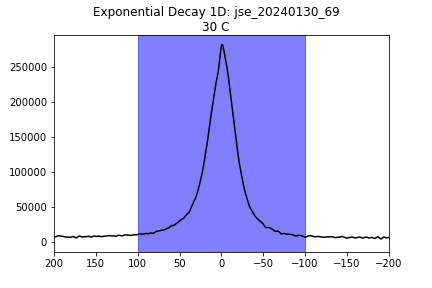

Supplement: Supplementary file 2 [file cg5c00666_si_002.zip › NMR/T1RHO/jse_20240130_69_figure.png]

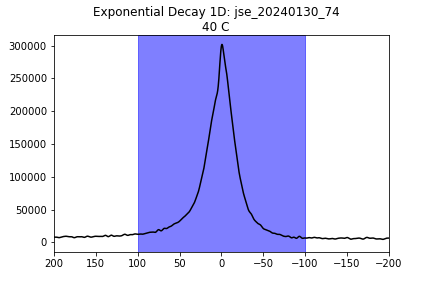

Supplement: Supplementary file 2 [file cg5c00666_si_002.zip › NMR/T1RHO/jse_20240130_74_figure.png]

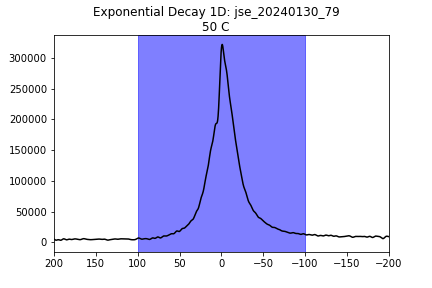

Supplement: Supplementary file 2 [file cg5c00666_si_002.zip › NMR/T1RHO/jse_20240130_79_figure.png]

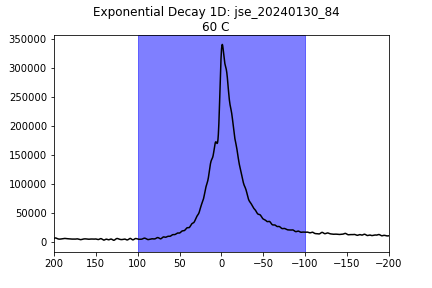

Supplement: Supplementary file 2 [file cg5c00666_si_002.zip › NMR/T1RHO/jse_20240130_84_figure.png]

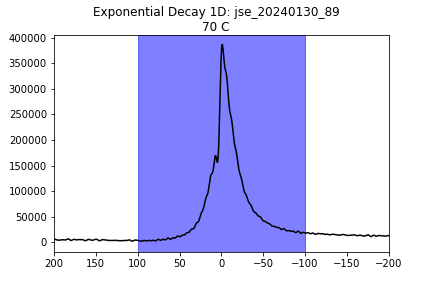

Supplement: Supplementary file 2 [file cg5c00666_si_002.zip › NMR/T1RHO/jse_20240130_89_figure.png]

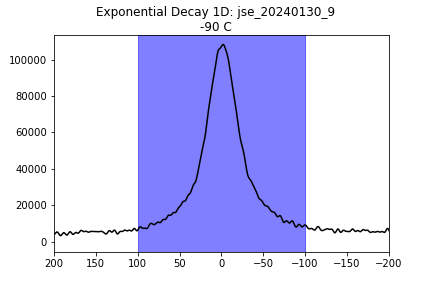

Supplement: Supplementary file 2 [file cg5c00666_si_002.zip › NMR/T1RHO/jse_20240130_9_figure.png]

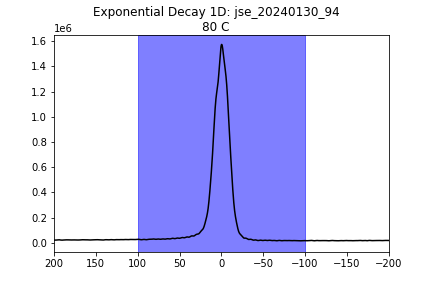

Supplement: Supplementary file 2 [file cg5c00666_si_002.zip › NMR/T1RHO/jse_20240130_94_figure.png]

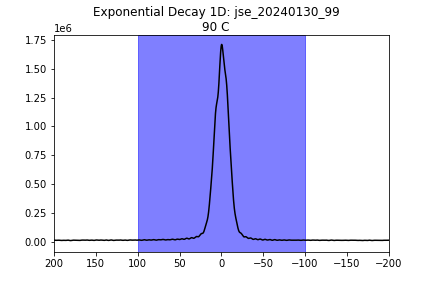

Supplement: Supplementary file 2 [file cg5c00666_si_002.zip › NMR/T1RHO/jse_20240130_99_figure.png]

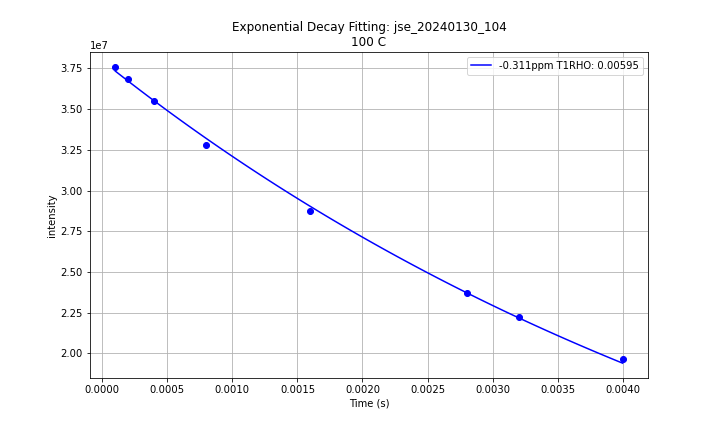

Supplement: Supplementary file 2 [file cg5c00666_si_002.zip › NMR/T1RHO/jse_T1RHO_104.png]

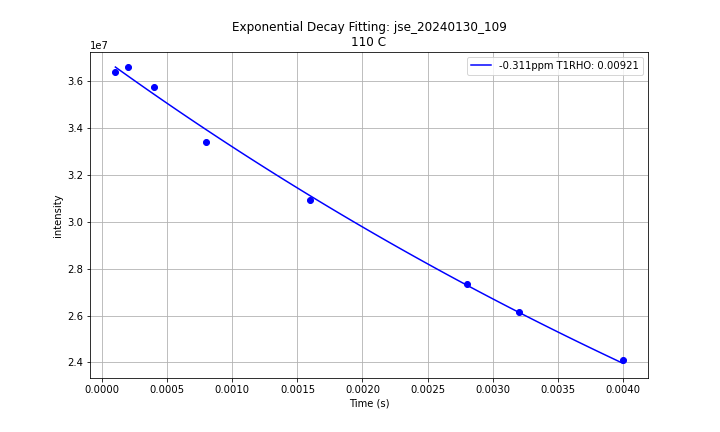

Supplement: Supplementary file 2 [file cg5c00666_si_002.zip › NMR/T1RHO/jse_T1RHO_109.png]

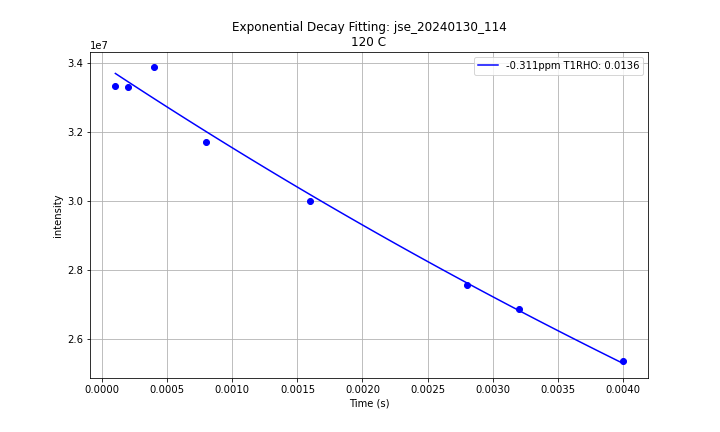

Supplement: Supplementary file 2 [file cg5c00666_si_002.zip › NMR/T1RHO/jse_T1RHO_114.png]

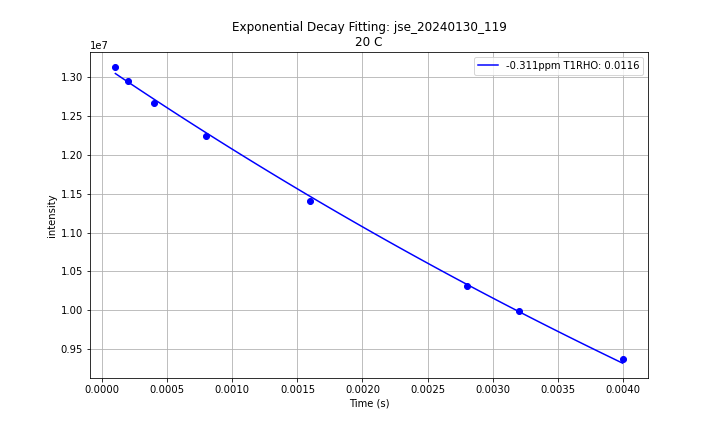

Supplement: Supplementary file 2 [file cg5c00666_si_002.zip › NMR/T1RHO/jse_T1RHO_119.png]
