# Supplementary material for: Potential Multiaxial Molecular Ferroelectricity through Chiral Cation Replacement
Source: Cryst Growth Des. 2025 Jul 21;25(15):6237–47. doi: 10.1021/acs.cgd.5c00666 (PMC12332970; doi:10.1021/acs.cgd.5c00666)

checkCIF/PLATON report

Structure factors have been supplied for datablock(s) S-Cd

THIS REPORT IS FOR GUIDANCE ONLY. IF USED AS PART OF A REVIEW PROCEDURE FOR PUBLICATION, IT SHOULD NOT REPLACE THE EXPERTISE OF AN EXPERIENCED CRYSTALLOGRAPHIC REFEREE.

No syntax errors found. CIF dictionary Interpreting this report

Datablock: S-Cd

```
Bond precision:   C-C = 0.0023 A           Wavelength=0.71073

Cell:             a=9.8943(6)     b=9.3092(6)        c=12.4610(7)
                  alpha=90        beta=96.188(2)     gamma=90
Temperature:      120 K

                Calculated                   Reported
Volume          1141.07(12)                  1141.07(12)
Space group     P 2                          P 1 2 1
Hall group      P 2y                         P 2y
Moiety formula  2(C6 H15 Cl N O), Cd Cl4     0.5(Cd Cl4), C6 H15 Cl N O
Sum formula     C12 H30 Cd Cl6 N2 O2         C6 H15 Cd0.5 Cl3 N O
Mr              559.49                       279.76
Dx,g cm-3       1.628                        1.628
Z               2                            4
Mu (mm-1)       1.667                        1.667
F000            564.0                        564.4
F000'           564.29
h,k,lmax        14,13,18                     14,13,18
Nref            7605[ 4011]                  7510
Tmin,Tmax       0.713,0.761                  0.671,0.746
Tmin'           0.692

Correction method= # Reported T Limits: Tmin=0.671 Tmax=0.746
AbsCorr = MULTI-SCAN

Data completeness= 1.87/0.99       Theta(max)= 31.520

                                                     wR2(reflections)=
R(reflections)= 0.0181( 7245)                        0.0404( 7510)

S = 1.038                  Npar= 229
```

The following ALERTS were generated. Each ALERT has the format
test-name_ALERT_alert-type_alert-level.
Click on the hyperlinks for more details of the test.

Alert level C

```
PLAT041_ALERT_1_C Calc. and Reported SumFormula    Strings  Differ     Please Check
              Calc: C12 H30 Cd Cl6 N2 O2
              Rep.: C6 H15 Cd0.5 Cl3 N O
PLAT042_ALERT_1_C Calc. and Reported MoietyFormula Strings  Differ     Please Check
              Calc: 2(C6 H15 Cl N O), Cd Cl4
              Rep.: 0.5(Cd Cl4), C6 H15 Cl N O
PLAT911_ALERT_3_C Missing FCF Refl Between Thmin & STh/L=    0.600         38 Report
                0  2  0,   1  1  0,   2  2  0,   4  0  0,  -4  1  1,  -3  0  1,
               -3  1  1,  -3  2  1,  -2  1  1,  -2  2  1,  -1  0  1,   0  1  1,
                0  2  1,   1  0  1,   1  2  1,   2  1  1,  -5  1  2,  -3  3  2,
               -2  2  2,  -1  1  2,  -1  3  2,   0  0  2,   1  1  2,   1  2  2,
                2  2  2,   3  3  2,  -2  4  3,   0  2  3,   0  3  3,   1  1  3,
                3  0  3,  -3  3  4,  -2  2  4,   0  0  4,   4  0  4,   1  0  5,
                2  1  5,   0  0  6,
PLAT913_ALERT_3_C Missing # of Very Strong Reflections in FCF ....         22 Note
                0  2  0,   2  2  0,   4  0  0,  -4  1  1,  -3  0  1,  -2  1  1,
                1  0  1,   2  1  1,  -5  1  2,  -2  2  2,  -1  1  2,   2  2  2,
                3  3  2,  -2  4  3,   0  2  3,   0  3  3,  -3  3  4,   0  0  4,
                4  0  4,   1  0  5,   2  1  5,   0  0  6,
PLAT927_ALERT_1_C Reported and Calculated  wR2 Differ by .........    -0.0018 Check
```

Alert level G

```
PLAT019_ALERT_1_G _diffrn_measured_fraction_theta_full/*_max < 1.0      0.992 Report
PLAT045_ALERT_1_G Calculated and Reported Z Differ by a Factor ...      0.500 Check
PLAT111_ALERT_2_G ADDSYM Detects New (Pseudo) Centre of Symmetry .         91 %Fit
PLAT112_ALERT_2_G ADDSYM Detects New (Pseudo) Symm. Elem         n         83 %Fit
PLAT113_ALERT_2_G ADDSYM Suggests Possible Pseudo/New  Space Group       P2/n Check
              Check Model Parameter Symmetry for Reflection Data Support
PLAT164_ALERT_4_G Nr. of Refined C-H H-Atoms in Heavy-Atom Struct.          2 Note
PLAT769_ALERT_4_G CIF Embedded Explicitly Supplied Scattering Data     Please Note
PLAT791_ALERT_4_G Model has Chirality at C2         (Sohncke SpGr)          S Verify
PLAT791_ALERT_4_G Model has Chirality at C8         (Sohncke SpGr)          S Verify
PLAT794_ALERT_5_G Tentative Bond Valency for Cd1       (II)      .       2.03 Info
PLAT794_ALERT_5_G Tentative Bond Valency for Cd2       (II)      .       2.05 Info
PLAT910_ALERT_3_G Missing # of FCF Reflection(s) Below Theta(Min).          1 Note
                0  0  1,
PLAT912_ALERT_4_G Missing # of FCF Reflections Above STh/L=  0.600          1 Note
PLAT969_ALERT_5_G The 'Henn et al.' R-Factor-gap value ..........      1.498 Note
              Predicted wR2: Based on SigI**2  2.70 or SHELX Weight  3.89
PLAT978_ALERT_2_G Number C-C Bonds with Positive Residual Density.          3 Info
PLAT982_ALERT_1_G The Cd-f'=   -0.8053 Deviates from  IT-Value =      -0.8075 Check
PLAT983_ALERT_1_G The Cd-f"=    1.2285 Deviates from  IT-Value =       1.2024 Check
PLAT983_ALERT_1_G The Cl-f"=    0.1603 Deviates from  IT-Value =       0.1585 Check
```

0 **ALERT level A** = Most likely a serious problem - resolve or explain
0 **ALERT level B** = A potentially serious problem, consider carefully
5 **ALERT level C** = Check. Ensure it is not caused by an omission or oversight
18 **ALERT level G** = General information/check it is not something unexpected

```
8 ALERT type 1 CIF construction/syntax error, inconsistent or missing data
4 ALERT type 2 Indicator that the structure model may be wrong or deficient
3 ALERT type 3 Indicator that the structure quality may be low
5 ALERT type 4 Improvement, methodology, query or suggestion
3 ALERT type 5 Informative message, check
```

It is advisable to attempt to resolve as many as possible of the alerts in all categories. Often the minor alerts point to easily fixed oversights, errors and omissions in your CIF or refinement strategy, so attention to these fine details can be worthwhile. In order to resolve some of the more serious problems it may be necessary to carry out additional measurements or structure refinements. However, the purpose of your study may justify the reported deviations and the more serious of these should normally be commented upon in the discussion or experimental section of a paper or in the "special_details" fields of the CIF. checkCIF was carefully designed to identify outliers and unusual parameters, but every test has its limitations and alerts that are not important in a particular case may appear. Conversely, the absence of alerts does not guarantee there are no aspects of the results needing attention. It is up to the individual to critically assess their own results and, if necessary, seek expert advice.

Publication of your CIF in IUCr journals

A basic structural check has been run on your CIF. These basic checks will be run on all CIFs submitted for publication in IUCr journals (*Acta Crystallographica*, *Journal of Applied Crystallography*, *Journal of Synchrotron Radiation*); however, if you intend to submit to *Acta Crystallographica Section C* or *E* or *IUCrData*, you should make sure that full publication checks are run on the final version of your CIF prior to submission.

Publication of your CIF in other journals

Please refer to the *Notes for Authors* of the relevant journal for any special instructions relating to CIF submission.

PLATON version of 02/02/2025; check.def file version of 02/02/2025

Datablock S-Cd - ellipsoid plot

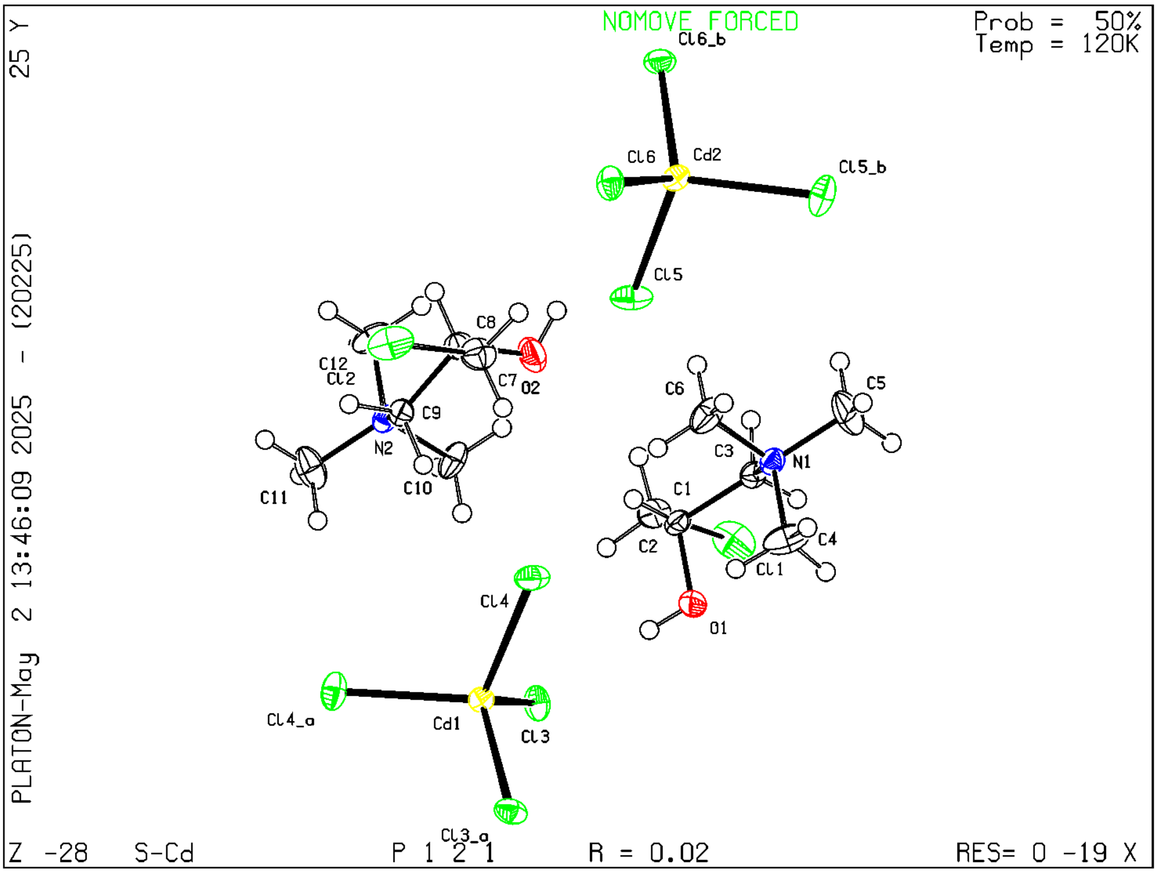

Supplement: Supplementary file 2 [file cg5c00666_si_002.zip › SXRD/S-Cd_checkcif.pdf]
