# Supplementary material for: Potential Multiaxial Molecular Ferroelectricity through Chiral Cation Replacement
Source: Cryst Growth Des. 2025 Jul 21;25(15):6237–47. doi: 10.1021/acs.cgd.5c00666 (PMC12332970; doi:10.1021/acs.cgd.5c00666)

checkCIF/PLATON report

Structure factors have been supplied for datablock(s) S-Co

THIS REPORT IS FOR GUIDANCE ONLY. IF USED AS PART OF A REVIEW PROCEDURE FOR PUBLICATION, IT SHOULD NOT REPLACE THE EXPERTISE OF AN EXPERIENCED CRYSTALLOGRAPHIC REFEREE.

No syntax errors found. CIF dictionary Interpreting this report

Datablock: S-Co

Bond precision: C-C = 0.0103 A Wavelength=0.71073

Cell:	a=9.3727(3)	b=9.3727(3)	c=66.173(3)
	alpha=90	beta=90	gamma=120
Temperature:	120 K		

	Calculated	Reported
Volume	5034.3(4)	5034.3(3)
Space group	P 32 2 1	P 32 2 1
Hall group	P 32 2"	P 32 2"
Moiety formula	2(C6 H15 Cl N O), Cl4 Co	0.5(Cl4 Co), C6 H15 Cl N O
Sum formula	C12 H30 Cl6 Co N2 O2	C6 H15 Cl3 Co0.5 N O
Mr	506.01	253.02
Dx,g cm-3	1.502	1.502
Z	9	18
Mu (mm-1)	1.491	1.491
F000	2349.0	2361.1
F000’	2360.60	
h,k,lmax	13,13,91	11,6,91
Nref	9383[5478]	9188
Tmin,Tmax	0.759,0.783	0.599,0.746
Tmin’	0.744	

Correction method= # Reported T Limits: Tmin=0.599 Tmax=0.746
AbsCorr = MULTI-SCAN

Data completeness= 1.68/0.98 Theta(max)= 29.570

R(reflections)= 0.0864(6744) wR2(reflections)= 0.1731(9188)

S = 1.052 Npar= 366

The following ALERTS were generated. Each ALERT has the format
test-name_ALERT_alert-type_alert-level.
Click on the hyperlinks for more details of the test.

Alert level C

```
PLAT041_ALERT_1_C Calc. and Reported SumFormula    Strings  Differ     Please Check
              Calc: C12 H30 Cl6 Co N2 O2
              Rep.: C6 H15 Cl3 Co0.5 N O
PLAT042_ALERT_1_C Calc. and Reported MoietyFormula Strings  Differ     Please Check
              Calc: 2(C6 H15 Cl N O), Cl4 Co
              Rep.: 0.5(Cl4 Co), C6 H15 Cl N O
PLAT213_ALERT_2_C Atom C00Y            has ADP max/min Ratio .....        3.9 prolat
PLAT220_ALERT_2_C NonSolvent   Resd 3  C   Ueq(max)/Ueq(min) Range        3.9 Ratio
PLAT222_ALERT_3_C NonSolvent Resd 3  H   Uiso(max)/Uiso(min) Range        5.0 Ratio
PLAT234_ALERT_4_C Large Hirshfeld Difference N1       --C3        .      0.18 Ang.
PLAT242_ALERT_2_C Low    'MainMol' Ueq as Compared to Neighbors of       N00F Check
PLAT242_ALERT_2_C Low    'MainMol' Ueq as Compared to Neighbors of        Co2 Check
PLAT250_ALERT_2_C Large U3/U1 Ratio for <U(i,j)> Tensor(Resd    1)        2.1 Note
PLAT250_ALERT_2_C Large U3/U1 Ratio for <U(i,j)> Tensor(Resd    3)        2.5 Note
PLAT341_ALERT_3_C Low Bond Precision on  C-C Bonds ..............    0.01025 Ang.
PLAT906_ALERT_3_C Large K Value in the Analysis of Variance ......      4.253 Check
PLAT918_ALERT_3_C Reflection(s) with I(obs) much Smaller I(calc) .          1 Check
```

Alert level G

```
PLAT002_ALERT_2_G Number of Distance or Angle Restraints on AtSite         13 Note
PLAT003_ALERT_2_G Number of Uiso or U(i,j)  Restrained non-H-Atoms         13 Report
PLAT045_ALERT_1_G Calculated and Reported Z Differ by a Factor ...      0.500 Check
PLAT068_ALERT_1_G Reported F000 Differs from Calcd (or Missing)...     Please Check
PLAT073_ALERT_1_G H-atoms ref., but hydrogen treatment Reported as     constr Check
PLAT083_ALERT_2_G SHELXL Second Parameter in WGHT  Unusually Large      48.46 Why ?
PLAT172_ALERT_4_G The CIF-Embedded .res File Contains DFIX Records          2 Report
PLAT176_ALERT_4_G The CIF-Embedded .res File Contains SADI Records          5 Report
PLAT178_ALERT_4_G The CIF-Embedded .res File Contains SIMU Records          1 Report
PLAT187_ALERT_4_G The CIF-Embedded .res File Contains RIGU Records          1 Report
PLAT190_ALERT_3_G A Non-default RIGU Restraint Value for First Par     0.0010 Report
PLAT190_ALERT_3_G A Non-default RIGU Restraint Value for SecondPar     0.0010 Report
PLAT301_ALERT_3_G Main Residue  Disorder ..............(Resd    1)        56% Note
PLAT412_ALERT_2_G Short Intra XH3 .. XHn     H3A       ..H14A     .      1.58 Ang.
                                                       x,y,z  =     1_555 Check
PLAT412_ALERT_2_G Short Intra XH3 .. XHn     H3C       ..H14B     .      1.80 Ang.
                                                       x,y,z  =     1_555 Check
PLAT414_ALERT_2_G Short Intra D-H..H-X       H5B       ..H1AC     .      2.10 Ang.
                                                       x,y,z  =     1_555 Check
PLAT720_ALERT_4_G Number of Unusual/Non-Standard Labels ..........         28 Note
              O00C    H00C    N00F    C00G    H00G    C00J    H00A    H00B
              H00D    C00K    H00E    H00F    C00N    H00H    H00I    C00U
              H00J    H00K    H00L    C00Y    H00M    H00N    H00O    H3AA
              H3AB    H1AA    H1AB    H1AC
PLAT769_ALERT_4_G CIF Embedded Explicitly Supplied Scattering Data     Please Note
PLAT779_ALERT_4_G Suspect or Irrelevant (Bond) Angle(s) in CIF ...      41.40 Deg.
              C14  -N1   -C3A     1_555   1_555   1_555 ......     #   38 Check
PLAT790_ALERT_4_G Centre of Gravity not Within Unit Cell: Resd.  #          2 Note
              C6 H15 Cl N O
PLAT791_ALERT_4_G Model has Chirality at C00G       (Sohncke SpGr)          S Verify
```

```
PLAT791_ALERT_4_G Model has Chirality at C2         (Sohncke SpGr)          S Verify
PLAT791_ALERT_4_G Model has Chirality at C8         (Sohncke SpGr)          S Verify
PLAT794_ALERT_5_G Tentative Bond Valency for Co2       (II)      .       2.11 Info
PLAT860_ALERT_3_G Number of Least-Squares Restraints ............         382 Note
PLAT910_ALERT_3_G Missing # of FCF Reflection(s) Below Theta(Min).          1 Note
                0  0  3,
PLAT912_ALERT_4_G Missing # of FCF Reflections Above STh/L=  0.600         50 Note
PLAT960_ALERT_3_G Number of Intensities with I < - 2*Sigma(I) ....          4 Check
PLAT969_ALERT_5_G The 'Henn et al.' R-Factor-gap value ..........      3.542 Note
             Predicted wR2: Based on SigI**2  4.89 or SHELX Weight 16.46
PLAT978_ALERT_2_G Number C-C Bonds with Positive Residual Density.          0 Info
PLAT982_ALERT_1_G The Co-f'=    0.3566 Deviates from  IT-Value =       0.3494 Check
PLAT983_ALERT_1_G The Cl-f"=    0.1603 Deviates from  IT-Value =       0.1585 Check
PLAT983_ALERT_1_G The Co-f"=    0.9658 Deviates from  IT-Value =       0.9721 Check
```

---

  0 **ALERT level A** = Most likely a serious problem - resolve or explain
  0 **ALERT level B** = A potentially serious problem, consider carefully
 13 **ALERT level C** = Check. Ensure it is not caused by an omission or oversight
 33 **ALERT level G** = General information/check it is not something unexpected

  8 ALERT type 1 CIF construction/syntax error, inconsistent or missing data
 13 ALERT type 2 Indicator that the structure model may be wrong or deficient
 10 ALERT type 3 Indicator that the structure quality may be low
 13 ALERT type 4 Improvement, methodology, query or suggestion
  2 ALERT type 5 Informative message, check

It is advisable to attempt to resolve as many as possible of the alerts in all categories. Often the minor alerts point to easily fixed oversights, errors and omissions in your CIF or refinement strategy, so attention to these fine details can be worthwhile. In order to resolve some of the more serious problems it may be necessary to carry out additional measurements or structure refinements. However, the purpose of your study may justify the reported deviations and the more serious of these should normally be commented upon in the discussion or experimental section of a paper or in the "special_details" fields of the CIF. checkCIF was carefully designed to identify outliers and unusual parameters, but every test has its limitations and alerts that are not important in a particular case may appear. Conversely, the absence of alerts does not guarantee there are no aspects of the results needing attention. It is up to the individual to critically assess their own results and, if necessary, seek expert advice.

Publication of your CIF in IUCr journals

A basic structural check has been run on your CIF. These basic checks will be run on all CIFs submitted for publication in IUCr journals (*Acta Crystallographica*, *Journal of Applied Crystallography*, *Journal of Synchrotron Radiation*); however, if you intend to submit to *Acta Crystallographica Section C* or *E* or *IUCrData*, you should make sure that full publication checks are run on the final version of your CIF prior to submission.

Publication of your CIF in other journals

Please refer to the *Notes for Authors* of the relevant journal for any special instructions relating to CIF submission.

PLATON version of 02/02/2025; check.def file version of 02/02/2025

Datablock S-Co - ellipsoid plot

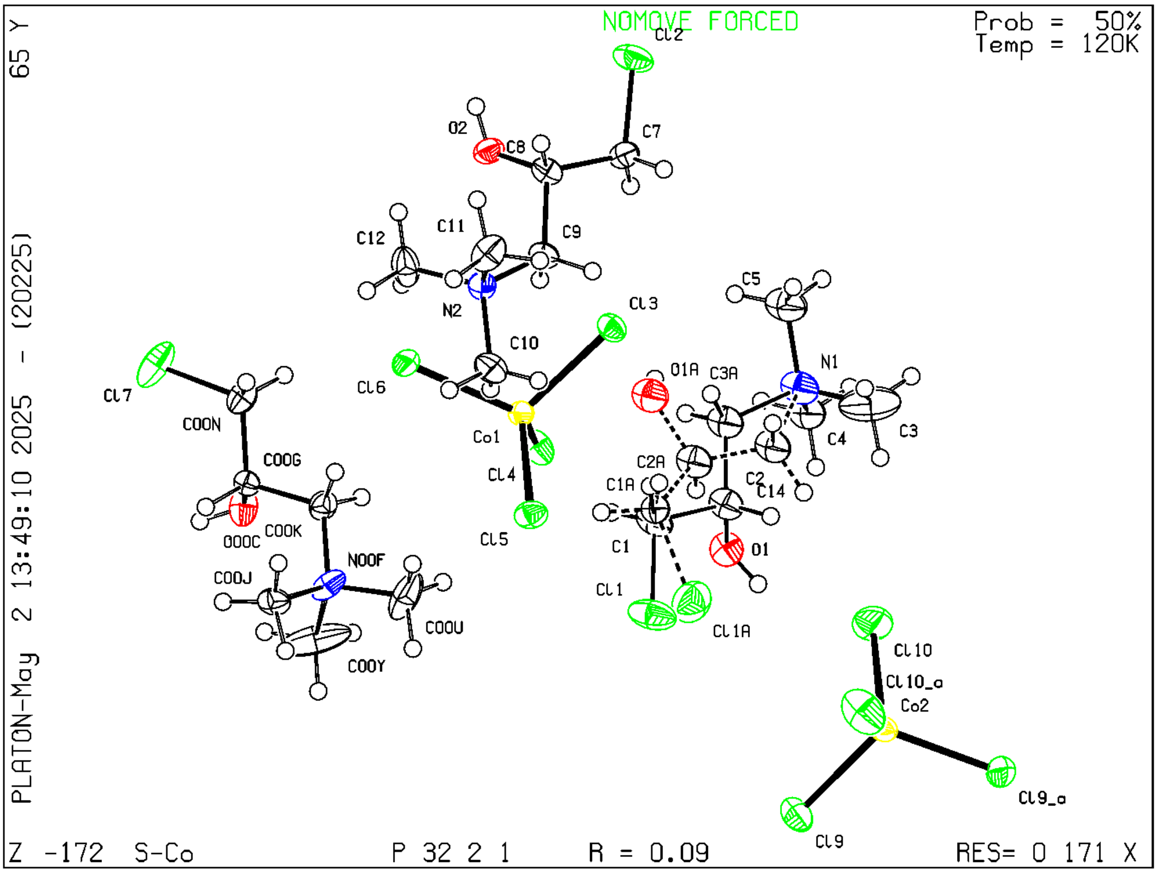

Supplement: Supplementary file 2 [file cg5c00666_si_002.zip › SXRD/S-Co_checkcif.pdf]
