# Supplementary material for: Potential Multiaxial Molecular Ferroelectricity through Chiral Cation Replacement
Source: Cryst Growth Des. 2025 Jul 21;25(15):6237–47. doi: 10.1021/acs.cgd.5c00666 (PMC12332970; doi:10.1021/acs.cgd.5c00666)

checkCIF/PLATON report

Structure factors have been supplied for datablock(s) S-Mn

THIS REPORT IS FOR GUIDANCE ONLY. IF USED AS PART OF A REVIEW PROCEDURE FOR PUBLICATION, IT SHOULD NOT REPLACE THE EXPERTISE OF AN EXPERIENCED CRYSTALLOGRAPHIC REFEREE.

No syntax errors found. CIF dictionary Interpreting this report

Datablock: S-Mn

```
Bond precision:   C-C = 0.0057 A          Wavelength=0.71073

Cell:               a=9.4127(3)      b=9.4127(3)      c=66.730(3)
                    alpha=90         beta=90          gamma=120
Temperature:        120 K

                 Calculated                   Reported
Volume           5120.1(4)                    5120.1(3)
Space group      P 32 2 1                     P 32 2 1
Hall group       P 32 2"                      P 32 2"
Moiety formula   2(C6 H15 Cl N O), Cl4 Mn     0.5(Cl4 Mn), C6 H15 Cl N O
Sum formula      C12 H30 Cl6 Mn N2 O2         C6 H15 Cl3 Mn0.5 N O
Mr               502.02                       251.02
Dx,g cm-3        1.465                        1.465
Z                9                            18
Mu (mm-1)        1.292                        1.292
F000             2331.0                       2343.0
F000'            2342.50
h,k,lmax         13,13,93                     11,6,92
Nref             9888[ 5757]                  9596
Tmin,Tmax        0.724,0.830                  0.620,0.746
Tmin'            0.679

Correction method= # Reported T Limits: Tmin=0.620 Tmax=0.746
AbsCorr = MULTI-SCAN

Data completeness= 1.67/0.97        Theta(max)= 29.910

                                                        wR2(reflections)=
R(reflections)= 0.0565( 8980)                           0.0999( 9596)

S = 1.046                  Npar= 366
```

The following ALERTS were generated. Each ALERT has the format
test-name_ALERT_alert-type_alert-level.
Click on the hyperlinks for more details of the test.

Alert level C

```
PLAT041_ALERT_1_C Calc. and Reported SumFormula    Strings  Differ     Please Check
              Calc: C12 H30 Cl6 Mn N2 O2
              Rep.: C6 H15 Cl3 Mn0.5 N O
PLAT042_ALERT_1_C Calc. and Reported MoietyFormula Strings  Differ     Please Check
              Calc: 2(C6 H15 Cl N O), Cl4 Mn
              Rep.: 0.5(Cl4 Mn), C6 H15 Cl N O
PLAT213_ALERT_2_C Atom C16            has ADP max/min Ratio .....        3.5 prolat
PLAT220_ALERT_2_C NonSolvent   Resd 1  C   Ueq(max)/Ueq(min) Range       3.3 Ratio
PLAT220_ALERT_2_C NonSolvent   Resd 3  C   Ueq(max)/Ueq(min) Range       3.8 Ratio
PLAT222_ALERT_3_C NonSolvent Resd 3  H   Uiso(max)/Uiso(min) Range       4.8 Ratio
PLAT250_ALERT_2_C Large U3/U1 Ratio for <U(i,j)> Tensor(Resd    3)       2.8 Note
PLAT336_ALERT_2_C Long Bond Distance for ..... C1       -Cl1           1.853 Ang.
PLAT790_ALERT_4_C Centre of Gravity not Within Unit Cell: Resd.  #         1 Note
              C6 H15 Cl N O
PLAT906_ALERT_3_C Large K Value in the Analysis of Variance ......     2.871 Check
PLAT911_ALERT_3_C Missing FCF Refl Between Thmin & STh/L=    0.600        13 Report
               -1  2  0,   0  2  1,   0  1  3,  -2  2  8,  -5  8  8,  -5 10 11,
                0  1 12,  -2  2 12,  -1  2 14,  -1  1 16,  -7  7 19,  -7  7 24,
               -8  8 26,
PLAT913_ALERT_3_C Missing # of Very Strong Reflections in FCF ....         4 Note
               -1  2  0,   0  1 12,  -1  2 14,  -1  1 16,
```

Alert level G

```
PLAT003_ALERT_2_G Number of Uiso or U(i,j)  Restrained non-H-Atoms         2 Report
PLAT045_ALERT_1_G Calculated and Reported Z Differ by a Factor ...     0.500 Check
PLAT068_ALERT_1_G Reported F000 Differs from Calcd (or Missing)...    Please Check
PLAT073_ALERT_1_G H-atoms ref., but hydrogen treatment Reported as    constr Check
PLAT083_ALERT_2_G SHELXL Second Parameter in WGHT  Unusually Large     15.32 Why ?
PLAT186_ALERT_4_G The CIF-Embedded .res File Contains ISOR Records         1 Report
PLAT301_ALERT_3_G Main Residue  Disorder .............(Resd    1)        56% Note
PLAT412_ALERT_2_G Short Intra XH3 .. XHn     H5A      ..H3AA     .       1.58 Ang.
                                                  x,y,z  =      1_555 Check
PLAT412_ALERT_2_G Short Intra XH3 .. XHn     H5C      ..H3AB     .       1.80 Ang.
                                                  x,y,z  =      1_555 Check
PLAT720_ALERT_4_G Number of Unusual/Non-Standard Labels ..........         4 Note
              H1AA    H1AB    H3AA    H3AB
PLAT769_ALERT_4_G CIF Embedded Explicitly Supplied Scattering Data     Please Note
PLAT779_ALERT_4_G Suspect or Irrelevant (Bond) Angle(s) in CIF ...     41.00 Deg.
              C3A  -N1   -C3      1_555   1_555   1_555 ......     #   15 Check
PLAT790_ALERT_4_G Centre of Gravity not Within Unit Cell: Resd.  #         2 Note
              C6 H15 Cl N O
PLAT790_ALERT_4_G Centre of Gravity not Within Unit Cell: Resd.  #         3 Note
              C6 H15 Cl N O
PLAT790_ALERT_4_G Centre of Gravity not Within Unit Cell: Resd.  #         4 Note
              Cl4 Mn
PLAT790_ALERT_4_G Centre of Gravity not Within Unit Cell: Resd.  #         5 Note
              Cl4 Mn
PLAT791_ALERT_4_G Model has Chirality at C2         (Sohncke SpGr)         S Verify
PLAT791_ALERT_4_G Model has Chirality at C8         (Sohncke SpGr)         S Verify
```

```
PLAT791_ALERT_4_G Model has Chirality at C14        (Sohncke SpGr)          S Verify
PLAT794_ALERT_5_G Tentative Bond Valency for Mn2       (II)      .       2.15 Info
PLAT860_ALERT_3_G Number of Least-Squares Restraints ............           6 Note
PLAT910_ALERT_3_G Missing # of FCF Reflection(s) Below Theta(Min).          2 Note
                0  0  3,   0  0  6,
PLAT912_ALERT_4_G Missing # of FCF Reflections Above STh/L=  0.600         93 Note
PLAT933_ALERT_2_G Number of HKL-OMIT Records in Embedded .res File          1 Note
                0  0  6,
PLAT969_ALERT_5_G The 'Henn et al.' R-Factor-gap value ..........      3.113 Note
              Predicted wR2: Based on SigI**2  3.21 or SHELX Weight  9.55
PLAT978_ALERT_2_G Number C-C Bonds with Positive Residual Density.          0 Info
PLAT982_ALERT_1_G The Mn-f'=    0.3478 Deviates from  IT-Value =       0.3368 Check
PLAT983_ALERT_1_G The Cl-f"=    0.1603 Deviates from  IT-Value =       0.1585 Check
PLAT983_ALERT_1_G The Mn-f"=    0.7326 Deviates from  IT-Value =       0.7283 Check
```

---

   0 **ALERT level A** = Most likely a serious problem - resolve or explain
   0 **ALERT level B** = A potentially serious problem, consider carefully
  12 **ALERT level C** = Check. Ensure it is not caused by an omission or oversight
  29 **ALERT level G** = General information/check it is not something unexpected

   8 ALERT type 1 CIF construction/syntax error, inconsistent or missing data
  11 ALERT type 2 Indicator that the structure model may be wrong or deficient
   7 ALERT type 3 Indicator that the structure quality may be low
  13 ALERT type 4 Improvement, methodology, query or suggestion
   2 ALERT type 5 Informative message, check

It is advisable to attempt to resolve as many as possible of the alerts in all categories. Often the minor alerts point to easily fixed oversights, errors and omissions in your CIF or refinement strategy, so attention to these fine details can be worthwhile. In order to resolve some of the more serious problems it may be necessary to carry out additional measurements or structure refinements. However, the purpose of your study may justify the reported deviations and the more serious of these should normally be commented upon in the discussion or experimental section of a paper or in the "special_details" fields of the CIF. checkCIF was carefully designed to identify outliers and unusual parameters, but every test has its limitations and alerts that are not important in a particular case may appear. Conversely, the absence of alerts does not guarantee there are no aspects of the results needing attention. It is up to the individual to critically assess their own results and, if necessary, seek expert advice.

Publication of your CIF in IUCr journals

A basic structural check has been run on your CIF. These basic checks will be run on all CIFs submitted for publication in IUCr journals (*Acta Crystallographica*, *Journal of Applied Crystallography*, *Journal of Synchrotron Radiation*); however, if you intend to submit to *Acta Crystallographica Section C* or *E* or *IUCrData*, you should make sure that full publication checks are run on the final version of your CIF prior to submission.

Publication of your CIF in other journals

Please refer to the *Notes for Authors* of the relevant journal for any special instructions relating to CIF submission.

PLATON version of 02/02/2025; check.def file version of 02/02/2025

Datablock S-Mn - ellipsoid plot

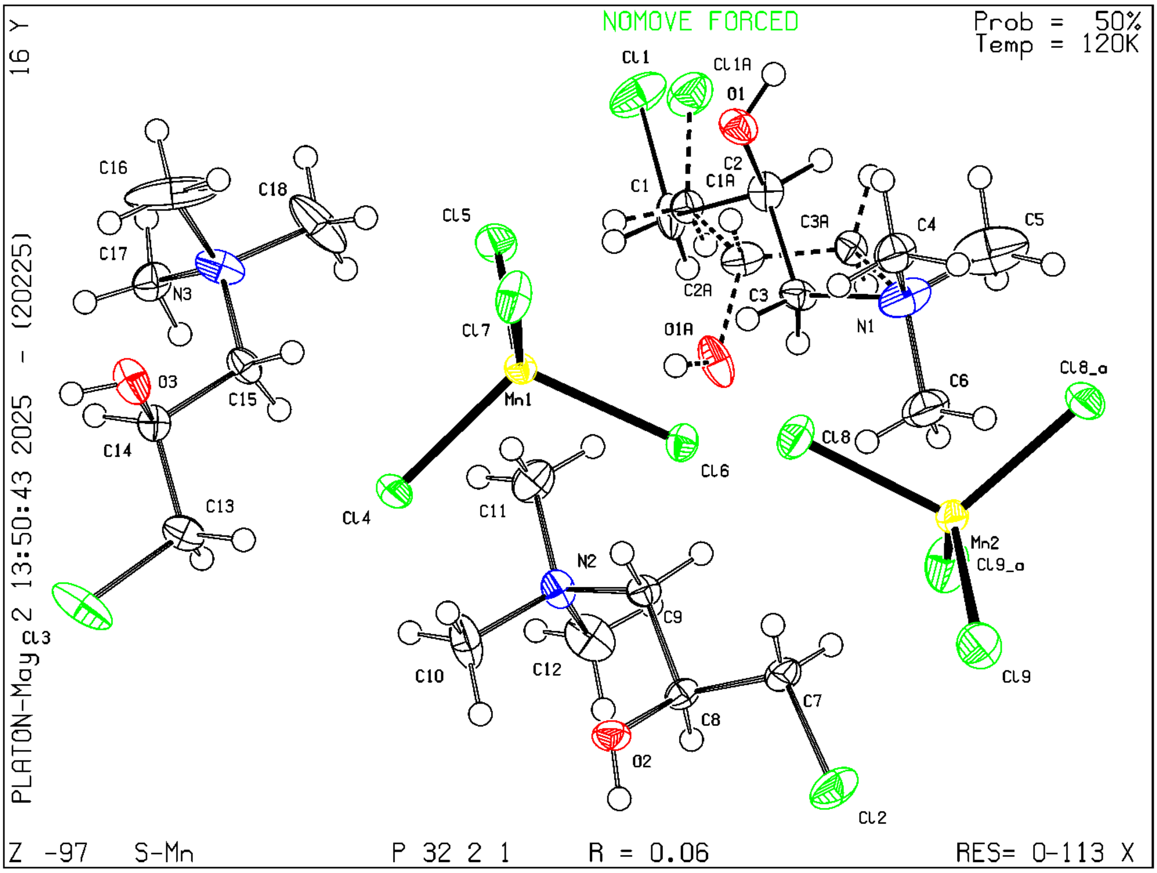

Supplement: Supplementary file 2 [file cg5c00666_si_002.zip › SXRD/S-Mn_checkcif.pdf]
