# Supplementary material for: Potential Multiaxial Molecular Ferroelectricity through Chiral Cation Replacement
Source: Cryst Growth Des. 2025 Jul 21;25(15):6237–47. doi: 10.1021/acs.cgd.5c00666 (PMC12332970; doi:10.1021/acs.cgd.5c00666)

checkCIF/PLATON report

Structure factors have been supplied for datablock(s) S-Ni

THIS REPORT IS FOR GUIDANCE ONLY. IF USED AS PART OF A REVIEW PROCEDURE FOR PUBLICATION, IT SHOULD NOT REPLACE THE EXPERTISE OF AN EXPERIENCED CRYSTALLOGRAPHIC REFEREE.

No syntax errors found.   CIF dictionary   Interpreting this report

Datablock: S-Ni

Bond precision: C-C = 0.0040 A   Wavelength=0.71073

Cell:	a=16.9483(6)	b=9.0380(3)	c=14.9863(5)
	alpha=90	beta=101.8272(11)	gamma=90
Temperature:	120 K		

	Calculated	Reported
Volume	2246.85(13)	2246.85(13)
Space group	C 2	C 1 2 1
Hall group	C 2y	C 2y
Moiety formula	2(C6 H15 Cl N O), Cl4 Ni	0.5(Cl4 Ni), C6 H15 Cl N O
Sum formula	C12 H30 Cl6 N2 Ni O2	C6 H15 Cl3 N Ni0.5 O
Mr	505.77	252.90
Dx,g cm-3	1.495	1.495
Z	4	8
Mu (mm-1)	1.584	1.584
F000	1048.0	1053.4
F000'	1053.11	
h,k,lmax	23,12,21	23,12,21
Nref	6558[3470]	6541
Tmin,Tmax	0.840,0.895	0.292,0.338
Tmin'	0.840	

Correction method= # Reported T Limits: Tmin=0.292 Tmax=0.338
AbsCorr = MULTI-SCAN

Data completeness= 1.89/1.00   Theta(max)= 29.990

R(reflections)= 0.0315(6088)   wR2(reflections)= 0.0769(6541)

S = 1.025   Npar= 223

The following ALERTS were generated. Each ALERT has the format
test-name_ALERT_alert-type_alert-level.
Click on the hyperlinks for more details of the test.

Alert level C

```
PLAT041_ALERT_1_C Calc. and Reported SumFormula    Strings  Differ     Please Check
              Calc: C12 H30 Cl6 N2 Ni O2
              Rep.: C6 H15 Cl3 N Ni0.5 O
PLAT042_ALERT_1_C Calc. and Reported MoietyFormula Strings  Differ     Please Check
              Calc: 2(C6 H15 Cl N O), Cl4 Ni
              Rep.: 0.5(Cl4 Ni), C6 H15 Cl N O
PLAT911_ALERT_3_C Missing FCF Refl Between Thmin & STh/L=    0.600          9 Report
                0  2  0,  -3  1  1,   2  2  1,  -2  0  2,   0  0  2,   4  0  2,
               -2  2  3,   1  1  3,  -4  0  4,
```

Alert level G

```
PLAT045_ALERT_1_G Calculated and Reported Z Differ by a Factor ...      0.500 Check
PLAT068_ALERT_1_G Reported F000 Differs from Calcd (or Missing)...     Please Check
PLAT111_ALERT_2_G ADDSYM Detects New (Pseudo) Centre of Symmetry .         91 %Fit
PLAT112_ALERT_2_G ADDSYM Detects New (Pseudo) Symm. Elem         c         91 %Fit
PLAT113_ALERT_2_G ADDSYM Suggests Possible Pseudo/New  Space Group       C2/c Check
              Check Model Parameter Symmetry for Reflection Data Support
PLAT720_ALERT_4_G Number of Unusual/Non-Standard Labels .........          14 Note
              H1AB    H1AC    H3AA    H3AB    H4AA    H4AB    H4AC    H5AA
              H5AB    H5AC    H6AA    H6AB    H6AC    H1AA
PLAT769_ALERT_4_G CIF Embedded Explicitly Supplied Scattering Data     Please Note
PLAT791_ALERT_4_G Model has Chirality at C2         (Sohncke SpGr)          S Verify
PLAT791_ALERT_4_G Model has Chirality at C2A        (Sohncke SpGr)          S Verify
PLAT794_ALERT_5_G Tentative Bond Valency for Ni1       (II)      .       2.06 Info
PLAT794_ALERT_5_G Tentative Bond Valency for Ni1A      (II)      .       2.04 Info
PLAT910_ALERT_3_G Missing # of FCF Reflection(s) Below Theta(Min).          1 Note
                0  0  1,
PLAT912_ALERT_4_G Missing # of FCF Reflections Above STh/L=  0.600          1 Note
PLAT913_ALERT_3_G Missing # of Very Strong Reflections in FCF ....          3 Note
               -3  1  1,   4  0  2,  -2  2  3,
PLAT933_ALERT_2_G Number of HKL-OMIT Records in Embedded .res File          1 Note
               -2  0  2,
PLAT960_ALERT_3_G Number of Intensities with I < - 2*Sigma(I) ....          1 Check
PLAT967_ALERT_5_G Note: Two-Theta Cutoff Value in Embedded .res ..       60.0 Degree
PLAT969_ALERT_5_G The 'Henn et al.' R-Factor-gap value ..........       3.112 Note
              Predicted wR2: Based on SigI**2  2.47 or SHELX Weight  7.50
PLAT978_ALERT_2_G Number C-C Bonds with Positive Residual Density.          3 Info
PLAT982_ALERT_1_G The Ni-f'=    0.3566 Deviates from  IT-Value =       0.3393 Check
PLAT983_ALERT_1_G The Cl-f"=    0.1603 Deviates from  IT-Value =       0.1585 Check
PLAT983_ALERT_1_G The Ni-f"=    1.1249 Deviates from  IT-Value =       1.1124 Check
```

0 **ALERT level A** = Most likely a serious problem - resolve or explain
0 **ALERT level B** = A potentially serious problem, consider carefully
3 **ALERT level C** = Check. Ensure it is not caused by an omission or oversight
22 **ALERT level G** = General information/check it is not something unexpected

7 ALERT type 1 CIF construction/syntax error, inconsistent or missing data

```
5 ALERT type 2 Indicator that the structure model may be wrong or deficient
4 ALERT type 3 Indicator that the structure quality may be low
5 ALERT type 4 Improvement, methodology, query or suggestion
4 ALERT type 5 Informative message, check
```

It is advisable to attempt to resolve as many as possible of the alerts in all categories. Often the minor alerts point to easily fixed oversights, errors and omissions in your CIF or refinement strategy, so attention to these fine details can be worthwhile. In order to resolve some of the more serious problems it may be necessary to carry out additional measurements or structure refinements. However, the purpose of your study may justify the reported deviations and the more serious of these should normally be commented upon in the discussion or experimental section of a paper or in the "special_details" fields of the CIF. checkCIF was carefully designed to identify outliers and unusual parameters, but every test has its limitations and alerts that are not important in a particular case may appear. Conversely, the absence of alerts does not guarantee there are no aspects of the results needing attention. It is up to the individual to critically assess their own results and, if necessary, seek expert advice.

Publication of your CIF in IUCr journals

A basic structural check has been run on your CIF. These basic checks will be run on all CIFs submitted for publication in IUCr journals (*Acta Crystallographica*, *Journal of Applied Crystallography*, *Journal of Synchrotron Radiation*); however, if you intend to submit to *Acta Crystallographica Section C* or *E* or *IUCrData*, you should make sure that full publication checks are run on the final version of your CIF prior to submission.

Publication of your CIF in other journals

Please refer to the *Notes for Authors* of the relevant journal for any special instructions relating to CIF submission.

PLATON version of 02/02/2025; check.def file version of 02/02/2025

Datablock S-Ni - ellipsoid plot

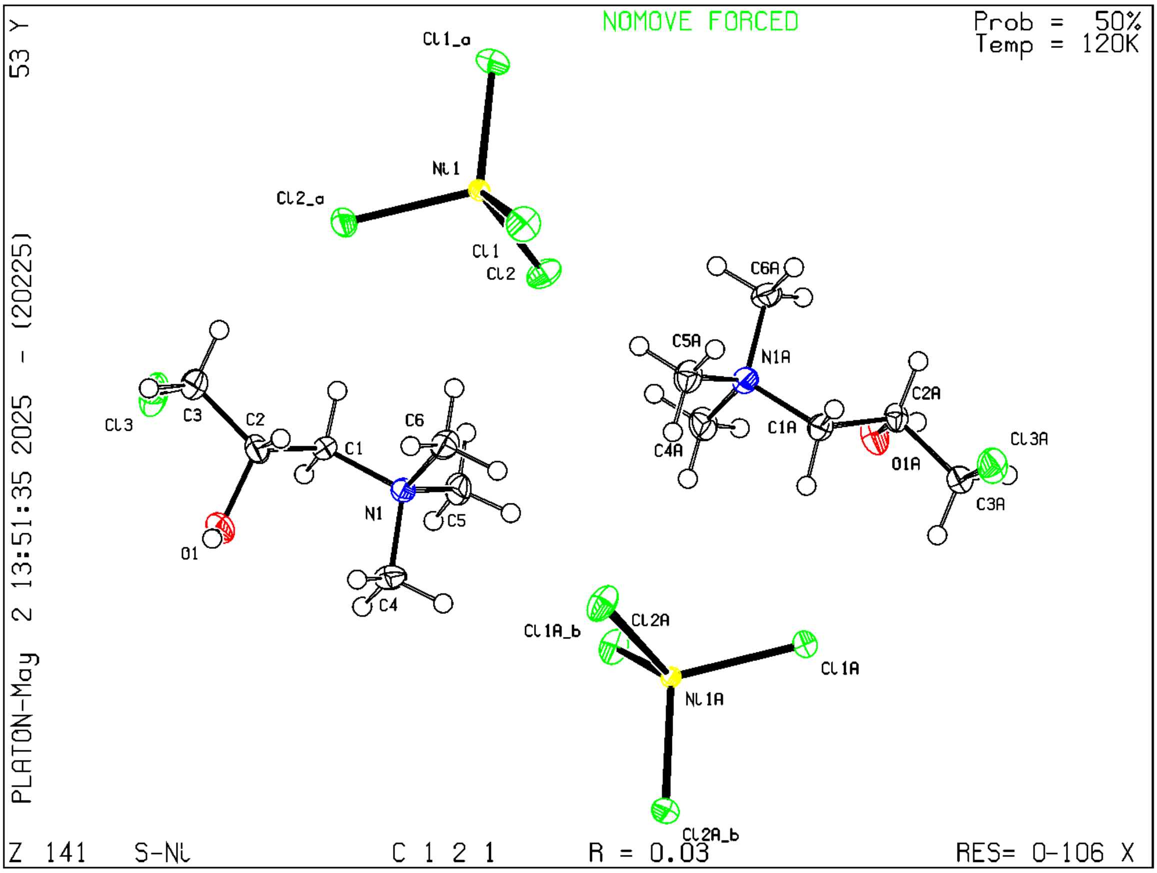

Supplement: Supplementary file 2 [file cg5c00666_si_002.zip › SXRD/S-Ni_checkcif.pdf]
