# Supplementary material for: Potential Multiaxial Molecular Ferroelectricity through Chiral Cation Replacement
Source: Cryst Growth Des. 2025 Jul 21;25(15):6237–47. doi: 10.1021/acs.cgd.5c00666 (PMC12332970; doi:10.1021/acs.cgd.5c00666)

checkCIF/PLATON report

Structure factors have been supplied for datablock(s) S-Zn

THIS REPORT IS FOR GUIDANCE ONLY. IF USED AS PART OF A REVIEW PROCEDURE FOR PUBLICATION, IT SHOULD NOT REPLACE THE EXPERTISE OF AN EXPERIENCED CRYSTALLOGRAPHIC REFEREE.

No syntax errors found.   CIF dictionary   Interpreting this report

Datablock: S-Zn

```
Bond precision:   C-C = 0.0025 A          Wavelength=0.71073

Cell:             a=16.9521(4)    b=9.0391(2)          c=14.9881(4)
                  alpha=90        beta=101.8307(8)     gamma=90
Temperature:      120 K

                 Calculated                  Reported
Volume           2247.87(9)                  2247.87(9)
Space group      C 2                         C 1 2 1
Hall group       C 2y                        C 2y
Moiety formula   2(C6 H15 Cl N O), Cl4 Zn    0.5(Cl4 Zn), C6 H15 Cl N O
Sum formula      C12 H30 Cl6 N2 O2 Zn        C6 H15 Cl3 N O Zn0.5
Mr               512.47                      256.25
Dx,g cm-3        1.514                       1.514
Z                4                           8
Mu (mm-1)        1.813                       1.813
F000             1056.0                      1061.3
F000'            1060.90
h,k,lmax         24,13,22                    24,13,22
Nref             7556[ 3991]                 7503
Tmin,Tmax        0.807,0.870                 0.696,0.746
Tmin'            0.807

Correction method= # Reported T Limits: Tmin=0.696 Tmax=0.746
AbsCorr = MULTI-SCAN

Data completeness= 1.88/0.99       Theta(max)= 31.620

R(reflections)= 0.0266( 6847)                        wR2(reflections)=
                                                     0.0544( 7503)
S = 1.021                  Npar= 217
```

The following ALERTS were generated. Each ALERT has the format
test-name_ALERT_alert-type_alert-level.
Click on the hyperlinks for more details of the test.

Alert level C

```
PLAT041_ALERT_1_C Calc. and Reported SumFormula    Strings  Differ     Please Check
              Calc: C12 H30 Cl6 N2 O2 Zn
              Rep.: C6 H15 Cl3 N O Zn0.5
PLAT042_ALERT_1_C Calc. and Reported MoietyFormula Strings  Differ     Please Check
              Calc: 2(C6 H15 Cl N O), Cl4 Zn
              Rep.: 0.5(Cl4 Zn), C6 H15 Cl N O
PLAT230_ALERT_2_C Hirshfeld Test Diff for    Cl1      --C1       .        5.1 s.u.
PLAT790_ALERT_4_C Centre of Gravity not Within Unit Cell: Resd.  #          1 Note
              C6 H15 Cl N O
PLAT911_ALERT_3_C Missing FCF Refl Between Thmin & STh/L=    0.600          8 Report
                0  2  0,  -3  1  1,   2  2  1,   0  0  2,   4  0  2,  -2  2  3,
                1  1  3,  -4  0  4,
```

Alert level G

```
PLAT045_ALERT_1_G Calculated and Reported Z Differ by a Factor ...      0.500 Check
PLAT068_ALERT_1_G Reported F000 Differs from Calcd (or Missing)...     Please Check
PLAT073_ALERT_1_G H-atoms ref., but hydrogen treatment Reported as     constr Check
PLAT111_ALERT_2_G ADDSYM Detects New (Pseudo) Centre of Symmetry .         91 %Fit
PLAT112_ALERT_2_G ADDSYM Detects New (Pseudo) Symm. Elem         c         91 %Fit
PLAT113_ALERT_2_G ADDSYM Suggests Possible Pseudo/New  Space Group       C2/c Check
              Check Model Parameter Symmetry for Reflection Data Support
PLAT769_ALERT_4_G CIF Embedded Explicitly Supplied Scattering Data     Please Note
PLAT790_ALERT_4_G Centre of Gravity not Within Unit Cell: Resd.  #          2 Note
              C6 H15 Cl N O
PLAT790_ALERT_4_G Centre of Gravity not Within Unit Cell: Resd.  #          3 Note
              Cl4 Zn
PLAT790_ALERT_4_G Centre of Gravity not Within Unit Cell: Resd.  #          4 Note
              Cl4 Zn
PLAT791_ALERT_4_G Model has Chirality at C2         (Sohncke SpGr)          S Verify
PLAT791_ALERT_4_G Model has Chirality at C8         (Sohncke SpGr)          S Verify
PLAT794_ALERT_5_G Tentative Bond Valency for Zn1       (II)      .       2.00 Info
PLAT794_ALERT_5_G Tentative Bond Valency for Zn2       (II)      .       1.98 Info
PLAT910_ALERT_3_G Missing # of FCF Reflection(s) Below Theta(Min).          1 Note
                0  0  1,
PLAT912_ALERT_4_G Missing # of FCF Reflections Above STh/L=  0.600         16 Note
PLAT913_ALERT_3_G Missing # of Very Strong Reflections in FCF ....          2 Note
               -3  1  1,   4  0  2,
PLAT969_ALERT_5_G The 'Henn et al.' R-Factor-gap value ..........       2.056 Note
              Predicted wR2: Based on SigI**2  2.64 or SHELX Weight  5.32
PLAT978_ALERT_2_G Number C-C Bonds with Positive Residual Density.          4 Info
PLAT982_ALERT_1_G The Zn-f'=    0.2915 Deviates from  IT-Value =       0.2839 Check
PLAT983_ALERT_1_G The Cl-f"=    0.1597 Deviates from  IT-Value =       0.1585 Check
PLAT983_ALERT_1_G The Zn-f"=    1.4382 Deviates from  IT-Value =       1.4301 Check
```

0 **ALERT level A** = Most likely a serious problem - resolve or explain
0 **ALERT level B** = A potentially serious problem, consider carefully
5 **ALERT level C** = Check. Ensure it is not caused by an omission or oversight
22 **ALERT level G** = General information/check it is not something unexpected

```
8 ALERT type 1 CIF construction/syntax error, inconsistent or missing data
5 ALERT type 2 Indicator that the structure model may be wrong or deficient
3 ALERT type 3 Indicator that the structure quality may be low
8 ALERT type 4 Improvement, methodology, query or suggestion
3 ALERT type 5 Informative message, check
```

It is advisable to attempt to resolve as many as possible of the alerts in all categories. Often the minor alerts point to easily fixed oversights, errors and omissions in your CIF or refinement strategy, so attention to these fine details can be worthwhile. In order to resolve some of the more serious problems it may be necessary to carry out additional measurements or structure refinements. However, the purpose of your study may justify the reported deviations and the more serious of these should normally be commented upon in the discussion or experimental section of a paper or in the "special_details" fields of the CIF. checkCIF was carefully designed to identify outliers and unusual parameters, but every test has its limitations and alerts that are not important in a particular case may appear. Conversely, the absence of alerts does not guarantee there are no aspects of the results needing attention. It is up to the individual to critically assess their own results and, if necessary, seek expert advice.

Publication of your CIF in IUCr journals

A basic structural check has been run on your CIF. These basic checks will be run on all CIFs submitted for publication in IUCr journals (*Acta Crystallographica*, *Journal of Applied Crystallography*, *Journal of Synchrotron Radiation*); however, if you intend to submit to *Acta Crystallographica Section C* or *E* or *IUCrData*, you should make sure that full publication checks are run on the final version of your CIF prior to submission.

Publication of your CIF in other journals

Please refer to the *Notes for Authors* of the relevant journal for any special instructions relating to CIF submission.

PLATON version of 02/02/2025; check.def file version of 02/02/2025

Datablock S-Zn - ellipsoid plot

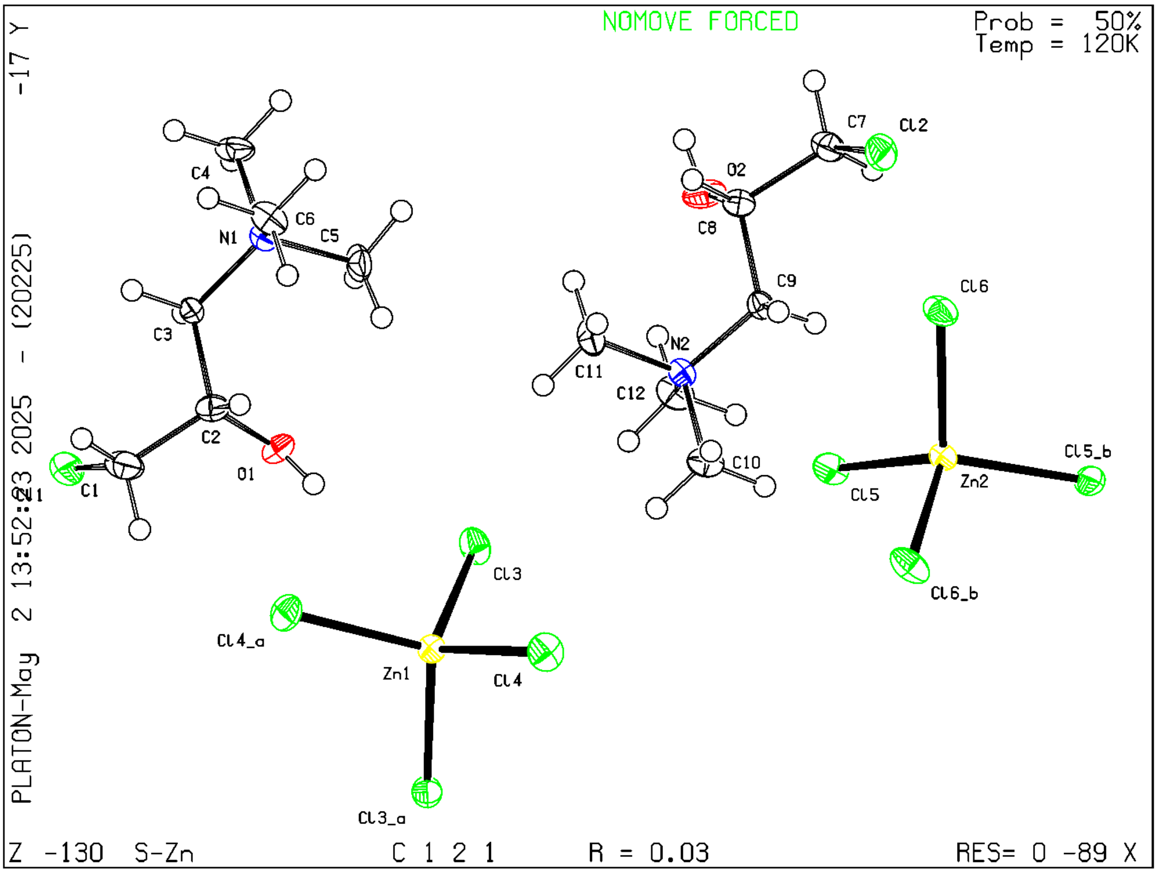

Supplement: Supplementary file 2 [file cg5c00666_si_002.zip › SXRD/S-Zn_checkcif.pdf]
