# Supplementary material for: Potential Multiaxial Molecular Ferroelectricity through Chiral Cation Replacement
Source: Cryst Growth Des. 2025 Jul 21;25(15):6237–47. doi: 10.1021/acs.cgd.5c00666 (PMC12332970; doi:10.1021/acs.cgd.5c00666)
Supplement: Supplementary file 2 [file cg5c00666_si_002.zip › SXRD/S-Zn_tables.html]

S-Zn


# S-Zn

b"\n \n \n "

Table 1 Crystal data and structure refinement for S-Zn.

| Identification code | S-Zn |
| Empirical formula | C_6_H_15_Cl_3_NOZn_0.5_ |
| Formula weight | 256.248 |
| Temperature/K | 120.00 |
| Crystal system | monoclinic |
| Space group | C2 |
| a/Å | 16.9521(4) |
| b/Å | 9.0391(2) |
| c/Å | 14.9881(4) |
| α/° | 90 |
| β/° | 101.8307(8) |
| γ/° | 90 |
| Volume/Å^3^ | 2247.87(9) |
| Z | 8 |
| ρ_calc_g/cm^3^ | 1.514 |
| μ/mm^‑1^ | 1.813 |
| F(000) | 1061.3 |
| Crystal size/mm^3^ | 0.118 × 0.117 × 0.077 |
| Radiation | Mo Kα (λ = 0.71073) |
| 2Θ range for data collection/° | 4.92 to 63.24 |
| Index ranges | -24 ≤ h ≤ 24, -13 ≤ k ≤ 13, -22 ≤ l ≤ 22 |
| Reflections collected | 32092 |
| Independent reflections | 7503 [R_int_ = 0.0329, R_sigma_ = 0.0321] |
| Data/restraints/parameters | 7503/1/217 |
| Goodness-of-fit on F^2^ | 1.021 |
| Final R indexes [I>=2σ (I)] | R_1_ = 0.0266, wR_2_ = 0.0521 |
| Final R indexes [all data] | R_1_ = 0.0320, wR_2_ = 0.0544 |
| Largest diff. peak/hole / e Å^-3^ | 0.33/-0.31 || Flack parameter | -0.006(4) |

Table 2 Fractional Atomic Coordinates (×10^4^) and Equivalent Isotropic Displacement Parameters (Å^2^×10^3^) for S-Zn. U_eq_ is defined as 1/3 of the trace of the orthogonalised U_IJ_ tensor.

| Atom | *x* | *y* | *z* | U(eq) |
| --- | --- | --- | --- | --- |
| Zn1 | -10000 | 188.90(19) | 5000 | 16.11(7) |
| Cl3 | -9136.2(3) | -1329.1(5) | 5962.2(3) | 23.6(1) |
| Cl4 | -10730.4(3) | 1426.5(5) | 5845.9(4) | 26.04(11) |
| Zn2 | -10000 | -4482.84(19) | 10000 | 15.37(7) |
| Cl5 | -9378.3(3) | -5836.5(5) | 9068.5(3) | 22.6(1) |
| Cl6 | -9063.5(3) | -3079.2(5) | 10925.1(3) | 25.87(11) |
| Cl1 | -6778.6(3) | -933.1(6) | 3402.5(4) | 37.91(15) |
| O1 | -7575.4(7) | -829.3(14) | 5112.5(9) | 24.9(3) |
| N1 | -6082.5(9) | 651.6(17) | 6369.1(10) | 15.7(3) |
| C1 | -7251.4(12) | 580(2) | 3870.7(14) | 27.6(5) |
| C2 | -7168.7(10) | 444(2) | 4893.6(14) | 19.0(4) |
| C3 | -6284.1(10) | 318(2) | 5356.7(11) | 15.9(4) |
| C4 | -5206.5(11) | 315(2) | 6704.6(14) | 24.7(5) |
| C5 | -6555.7(13) | -281(2) | 6903.2(14) | 25.0(5) |
| C6 | -6216.8(13) | 2265(2) | 6543.8(14) | 22.8(4) |
| Cl2 | -8344.1(3) | 1867.9(5) | 11541.8(4) | 28.95(11) |
| O2 | -7442.0(7) | -901.3(13) | 9924.1(10) | 25.3(3) |
| N2 | -8891.9(9) | -63.8(17) | 8626.0(11) | 16.4(3) |
| C7 | -7819.2(12) | 345(2) | 11165.5(13) | 23.2(4) |
| C8 | -7870.9(10) | 348(2) | 10137.1(13) | 17.6(4) |
| C9 | -8751.4(10) | 223(2) | 9649.5(12) | 16.2(4) |
| C10 | -9761.0(11) | 298(2) | 8238.3(14) | 23.7(4) |
| C11 | -8366.3(13) | 900(2) | 8180.4(14) | 23.3(4) |
| C12 | -8762.9(13) | -1662(2) | 8432.2(15) | 23.9(5) |

Table 3 Anisotropic Displacement Parameters (Å^2^×10^3^) for S-Zn. The Anisotropic displacement factor exponent takes the form: -2π^2^[h^2^a\*^2^U_11_+2hka\*b\*U_12_+…].

| Atom | U_11_ | U_22_ | U_33_ | U_12_ | U_13_ | U_23_ |
| --- | --- | --- | --- | --- | --- | --- |
| Zn1 | 17.62(14) | 13.78(16) | 16.88(15) | -0 | 3.41(11) | 0 |
| Cl3 | 26.6(2) | 25.2(3) | 19.7(2) | 10.05(19) | 6.44(18) | 5.3(2) |
| Cl4 | 28.0(2) | 22.5(2) | 28.9(3) | 7.83(19) | 9.02(19) | -3.6(2) |
| Zn2 | 17.85(14) | 13.63(15) | 14.08(14) | -0 | 1.99(11) | 0 |
| Cl5 | 22.0(2) | 24.1(2) | 20.7(2) | 7.74(19) | 2.06(16) | -3.99(19) |
| Cl6 | 33.5(3) | 23.4(3) | 18.1(2) | -13.3(2) | -0.79(18) | 0.7(2) |
| Cl1 | 27.9(2) | 66.4(4) | 20.1(2) | -7.5(3) | 6.34(19) | -12.3(3) |
| O1 | 16.6(6) | 31.0(7) | 27.2(7) | -7.9(5) | 5.1(5) | -1.9(6) |
| N1 | 18.8(7) | 12.7(7) | 14.8(8) | -0.4(6) | 1.6(6) | 0.9(6) |
| C1 | 27.7(10) | 28.0(11) | 22.8(10) | -5.6(9) | -5.1(8) | 3.1(9) |
| C2 | 17.6(8) | 16.4(9) | 21.6(9) | -2.1(8) | 0.3(7) | -1.1(8) |
| C3 | 17.1(8) | 18.9(9) | 11.5(8) | -2.9(7) | 2.4(6) | 0.8(7) |
| C4 | 18.5(9) | 27.1(12) | 23.8(10) | -0.1(8) | -7.0(7) | 4.0(9) |
| C5 | 33.8(11) | 25.3(11) | 18.3(9) | -8.8(9) | 10.7(8) | 2.8(8) |
| C6 | 30.9(11) | 16.0(10) | 19.7(10) | 4.2(8) | 1.3(8) | -2.0(8) |
| Cl2 | 31.9(2) | 34.7(3) | 22.7(2) | -6.6(2) | 11.22(19) | -10.4(2) |
| O2 | 16.6(6) | 20.6(6) | 35.8(7) | 2.8(5) | -1.5(5) | -7.4(6) |
| N2 | 19.4(7) | 15.6(8) | 13.9(8) | 0.8(6) | 2.9(6) | 0.6(6) |
| C7 | 28.3(10) | 21.1(10) | 18.0(9) | -6.1(8) | -0.3(7) | 1.5(7) |
| C8 | 18.9(8) | 13.7(9) | 18.8(9) | 0.4(7) | 1.0(7) | -2.6(7) |
| C9 | 17.7(8) | 16.8(9) | 14.3(8) | 1.3(7) | 4.3(6) | -0.6(7) |
| C10 | 21.3(9) | 25.1(11) | 22.5(10) | 2.0(8) | -1.0(7) | -1.3(9) |
| C11 | 31.8(10) | 22.4(11) | 17.2(9) | -3.0(9) | 8.8(8) | 0.6(8) |
| C12 | 32.5(11) | 14.6(10) | 23.1(11) | -0.2(9) | 2.2(9) | -3.5(8) |

Table 4 Bond Lengths for S-Zn.

| Atom | Atom | Length/Å |  | Atom | Atom | Length/Å |
| --- | --- | --- | --- | --- | --- | --- |
| Zn1 | Cl3^1^ | 2.2893(4) |  | N1 | C5 | 1.503(2) |
| Zn1 | Cl3 | 2.2893(4) |  | N1 | C6 | 1.507(3) |
| Zn1 | Cl4^1^ | 2.2441(5) |  | C1 | C2 | 1.516(3) |
| Zn1 | Cl4 | 2.2441(5) |  | C2 | C3 | 1.522(2) |
| Zn2 | Cl5^2^ | 2.2717(5) |  | Cl2 | C7 | 1.792(2) |
| Zn2 | Cl5 | 2.2717(5) |  | O2 | C8 | 1.415(2) |
| Zn2 | Cl6 | 2.2694(4) |  | N2 | C9 | 1.526(2) |
| Zn2 | Cl6^2^ | 2.2694(4) |  | N2 | C10 | 1.505(2) |
| Cl1 | C1 | 1.799(2) |  | N2 | C11 | 1.498(2) |
| O1 | C2 | 1.415(2) |  | N2 | C12 | 1.499(3) |
| N1 | C3 | 1.516(2) |  | C7 | C8 | 1.526(3) |
| N1 | C4 | 1.498(2) |  | C8 | C9 | 1.526(2) |

^1^-2-X,+Y,1-Z; ^2^-2-X,+Y,2-Z

Table 5 Bond Angles for S-Zn.

| Atom | Atom | Atom | Angle/˚ |  | Atom | Atom | Atom | Angle/˚ |
| --- | --- | --- | --- | --- | --- | --- | --- | --- |
| Cl3^1^ | Zn1 | Cl3 | 106.35(2) |  | C6 | N1 | C5 | 109.52(16) |
| Cl4^1^ | Zn1 | Cl3 | 107.392(18) |  | C2 | C1 | Cl1 | 112.13(15) |
| Cl4^1^ | Zn1 | Cl3^1^ | 107.376(17) |  | C1 | C2 | O1 | 110.62(16) |
| Cl4 | Zn1 | Cl3 | 107.376(17) |  | C3 | C2 | O1 | 108.32(15) |
| Cl4 | Zn1 | Cl3^1^ | 107.392(18) |  | C3 | C2 | C1 | 110.22(16) |
| Cl4 | Zn1 | Cl4^1^ | 120.20(2) |  | C2 | C3 | N1 | 116.12(15) |
| Cl5^2^ | Zn2 | Cl5 | 114.82(2) |  | C10 | N2 | C9 | 106.86(14) |
| Cl6 | Zn2 | Cl5 | 108.788(18) |  | C11 | N2 | C9 | 111.40(15) |
| Cl6 | Zn2 | Cl5^2^ | 106.273(16) |  | C11 | N2 | C10 | 109.04(15) |
| Cl6^2^ | Zn2 | Cl5 | 106.273(16) |  | C12 | N2 | C9 | 111.09(15) |
| Cl6^2^ | Zn2 | Cl5^2^ | 108.788(18) |  | C12 | N2 | C10 | 107.76(16) |
| Cl6^2^ | Zn2 | Cl6 | 112.01(2) |  | C12 | N2 | C11 | 110.53(16) |
| C4 | N1 | C3 | 107.46(14) |  | C8 | C7 | Cl2 | 112.78(14) |
| C5 | N1 | C3 | 112.71(15) |  | C7 | C8 | O2 | 107.50(15) |
| C5 | N1 | C4 | 107.93(15) |  | C9 | C8 | O2 | 109.27(14) |
| C6 | N1 | C3 | 110.83(15) |  | C9 | C8 | C7 | 109.37(16) |
| C6 | N1 | C4 | 108.24(16) |  | C8 | C9 | N2 | 115.47(15) |

^1^-2-X,+Y,1-Z; ^2^-2-X,+Y,2-Z

Table 6 Torsion Angles for S-Zn.

| A | B | C | D | Angle/˚ |  | A | B | C | D | Angle/˚ |
| --- | --- | --- | --- | --- | --- | --- | --- | --- | --- | --- |
| Cl1 | C1 | C2 | O1 | -63.73(14) |  | Cl2 | C7 | C8 | O2 | -179.61(12) |
| Cl1 | C1 | C2 | C3 | 56.04(15) |  | Cl2 | C7 | C8 | C9 | -61.07(14) |
| O1 | C2 | C3 | N1 | -77.26(16) |  | O2 | C8 | C9 | N2 | -52.14(17) |
| N1 | C3 | C2 | C1 | 161.58(17) |  | N2 | C9 | C8 | C7 | -169.56(16) |
| C2 | C3 | N1 | C4 | 174.34(16) |  | C8 | C9 | N2 | C10 | -163.49(16) |
| C2 | C3 | N1 | C5 | 55.56(19) |  | C8 | C9 | N2 | C11 | -44.49(18) |
| C2 | C3 | N1 | C6 | -67.58(19) |  | C8 | C9 | N2 | C12 | 79.21(19) |

Table 7 Hydrogen Atom Coordinates (Å×10^4^) and Isotropic Displacement Parameters (Å^2^×10^3^) for S-Zn.

| Atom | *x* | *y* | *z* | U(eq) |
| --- | --- | --- | --- | --- |
| H1 | -8046(4) | -600(5) | 5156(15) | 37.3(4) |
| H1a | -7003.7(12) | 1522(2) | 3730.9(14) | 33.2(5) |
| H1b | -7830.4(12) | 611(2) | 3577.8(14) | 33.2(5) |
| H2 | -7405.1(10) | 1342(2) | 5128.2(14) | 22.9(5) |
| H3a | -5970.1(10) | 999(2) | 5046.7(11) | 19.1(4) |
| H3b | -6101.4(10) | -701(2) | 5263.7(11) | 19.1(4) |
| H4a | -5106.9(19) | -732(4) | 6598(9) | 37.1(7) |
| H4b | -4883.7(11) | 930(12) | 6377(7) | 37.1(7) |
| H4c | -5056(2) | 526(16) | 7359(3) | 37.1(7) |
| H5a | -7128.1(18) | -22(12) | 6731(7) | 37.6(7) |
| H5b | -6485(7) | -1329(2) | 6771(8) | 37.6(7) |
| H5c | -6362(6) | -101(12) | 7556.3(14) | 37.6(7) |
| H6a | -5883(6) | 2871(2) | 6224(8) | 34.2(7) |
| H6b | -6786(2) | 2511(4) | 6321(8) | 34.2(7) |
| H6c | -6068(8) | 2462(4) | 7199.8(17) | 34.2(7) |
| H2a | -6946.1(12) | -771(11) | 10120(14) | 38.0(4) |
| H7a | -7245.6(12) | 384(2) | 11478.6(13) | 27.8(5) |
| H7b | -8048.6(12) | -592(2) | 11341.3(13) | 27.8(5) |
| H8 | -7628.2(10) | 1276(2) | 9948.4(13) | 21.1(5) |
| H9a | -9030.8(10) | 1151(2) | 9749.8(12) | 19.4(4) |
| H9b | -9004.1(10) | -589(2) | 9934.6(12) | 19.4(4) |
| H10a | -10108.6(11) | -335(11) | 8523(7) | 35.6(7) |
| H10b | -9863(2) | 1338(5) | 8361(9) | 35.6(7) |
| H10c | -9877(3) | 127(16) | 7579(2) | 35.6(7) |
| H11a | -8449(6) | 1939(3) | 8325(8) | 34.9(6) |
| H11b | -7800.0(14) | 635(11) | 8406(7) | 34.9(6) |
| H11c | -8505(6) | 758(12) | 7518.4(16) | 34.9(6) |
| H12a | -8195(2) | -1919(4) | 8658(8) | 35.9(7) |
| H12b | -9102(6) | -2278(2) | 8739(8) | 35.9(7) |
| H12c | -8906(8) | -1836(4) | 7773.3(17) | 35.9(7) |

Experimental

Single crystals of C_6_H_15_Cl_3_NOZn_0.5_
[S-Zn]
were
[].
A suitable crystal was selected and
[]
on a
Bruker APEX-II CCD
diffractometer. The crystal was kept at 120.00 K during data collection.
Using Olex2 [1], the structure was solved with the
olex2.solve
[2] structure solution program using
Charge Flipping
and refined with the
olex2.refine
[3] refinement package using
Gauss-Newton
minimisation.

1. Dolomanov, O.V., Bourhis, L.J., Gildea, R.J, Howard, J.A.K. & Puschmann, H.
   (2009), J. Appl. Cryst. 42, 339-341.
2. Bourhis, L.J., Dolomanov, O.V., Gildea, R.J., Howard, J.A.K., Puschmann, H.
   (2015). Acta Cryst. A71, 59-75.
3. Bourhis, L.J., Dolomanov, O.V., Gildea, R.J., Howard, J.A.K., Puschmann, H.
   (2015). Acta Cryst. A71, 59-75.

Crystal structure determination of
[S-Zn]

**Crystal Data**
for C_6_H_15_Cl_3_NOZn_0.5_ (*M* =256.248 g/mol):
monoclinic, space group C2 (no. 5),
*a* = 16.9521(4) Å, *b* = 9.0391(2) Å, *c* = 14.9881(4) Å, *β* = 101.8307(8)°,
*V* = 2247.87(9) Å^3^,
*Z* = 8,
*T* = 120.00 K,
μ(Mo Kα) = 1.813 mm^-1^,
*Dcalc* = 1.514 g/cm^3^,
32092 reflections measured (4.92° ≤ 2Θ ≤ 63.24°),
7503 unique (*R*_int_ = 0.0329, R_sigma_ = 0.0321) which were used in all calculations.
The final *R*_1_ was 0.0266
(I>=2u(I)) and *wR*_2_ was 0.0544 (all data).

Refinement model description

Number of restraints - 1,
number of constraints - 44.

Details:

```
1. Fixed Uiso
```

This report has been created with Olex2, compiled on
2021.12.09 svn.r5202d8cf for OlexSys. Please
let us know
if there are any errors or if you would like to have additional features.
