# Supplementary material for: Correction to “Potential Multiaxial Molecular Ferroelectricity through Chiral Cation Replacement”
Source: Cryst Growth Des. 2026 Jun 1;26(12):4778. doi: 10.1021/acs.cgd.6c00647 (PMC13281391; doi:10.1021/acs.cgd.6c00647)
Supplement: Supplementary file 1 [file cg6c00647_si_001.pdf]

Potential multiaxial molecular ferroelectricity through chiral cation replacement

Sam Y. Thompson[a], Rebecca H. Abeyasekere[a], Samuel J. Page[a], Paul Hodgkinson[a], Cameron A. M. Scott[b], Nicholas C. Bristowe[b], Oliver J. Wagstaff[a] and John S. O. Evans[a]*

[a]Department of Chemistry, Lower Mount Joy, South Road, Durham University, Durham DH1 3LE, United Kingdom

[b]Centre for Materials Physics, Durham University, Durham DH1 3LE, United Kingdom

Supplementary Information

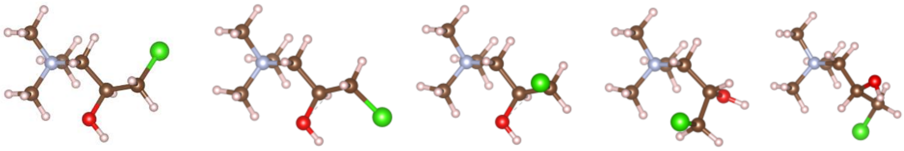

Conformer	1	2	3	4	5
MM3 (ΔE / kJ mol^{-1})	0.000	+5.827	+6.655	+10.630	+25.506
DFT (ΔG / kJ mol^{-1})	0.000	+0.171	+1.132	+10.001	+25.449

Figure S1 – Five lowest energy conformers of CTA^+. Conformers 1-3 are those found in the room temperature structures of ***Rac*-M** and ***S*-M**. DFT calculations are at 298.15 K.

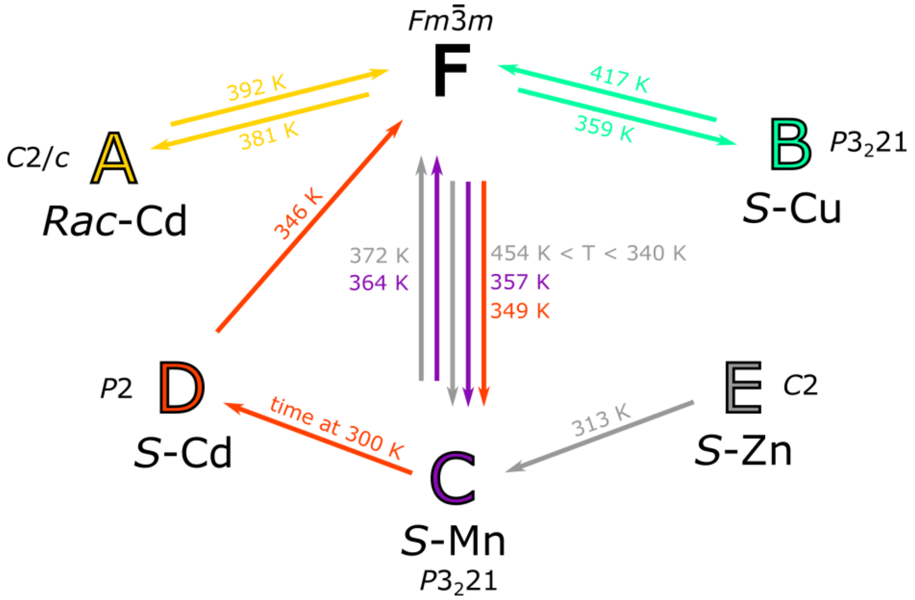

Figure S2 - Phase sequences observed in variable temperature powder X-ray diffraction studies of ***Rac*-M** and ***S*-M**.

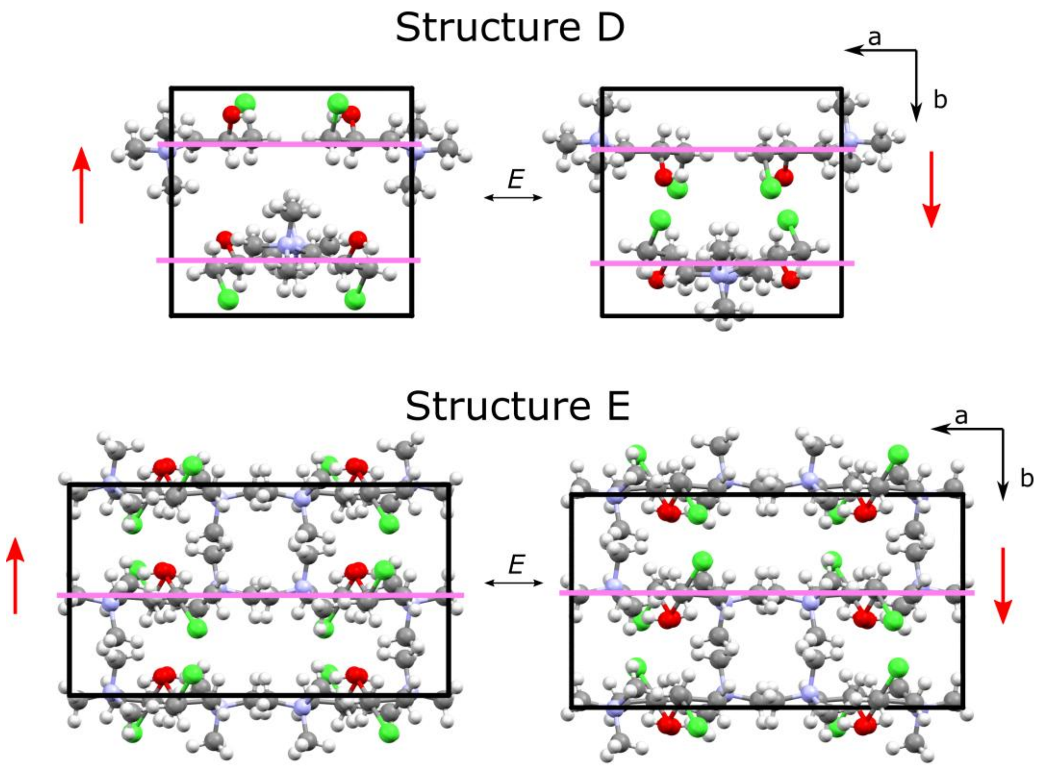

Figure S3 - Suggested ferroelectric switching mechanism for structures D and E. The polarization is primarily due to the C-O bond vector components parallel to the *b*-axis; this is shown by red oxygen atoms above and below the pink planes. The potential switching mechanism occurs via CTA^+ rotation about an axis in the pink plane.

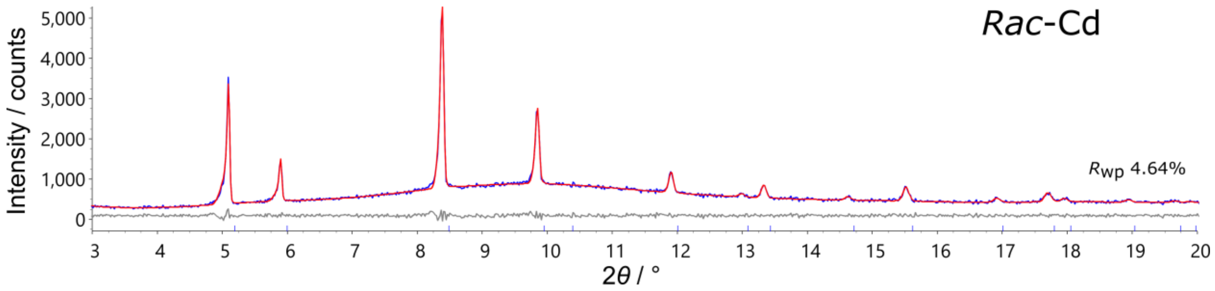

Figure S4 - Powder X-ray diffraction data (blue) of *Rac*-Cd at 437 K with a Pawley refinement fit (red).

Table S1- Crystal data and Rietveld refinement information for *S*-Mn (structure type C).

**Empirical formula**	$C_6H_{15}Cl_3Mn_{0.5}NO$	**Radiation**	Cu Kα
**Formula weight**	251.019	$\mathbf{2\theta}$ **/ °**	3–60
**Space group**	$P3_221$	***T / K***	298
**Unit cell dimensions**		$\mathbf{B_{eq}}$ **/ Å²**	
a / Å	9.4780(2)	$MnCl_4$	6.6(4)
c / Å	67.807(2)	CTA^+	5.8(5)
***V* / Å³**	5277.1(3)	$\mathbf{R_{wp}}$ **(%)**	5.36
Z	18	$\mathbf{R_{Bragg}}$ **(%)**	4.28
$\mathbf{D_x}$ **/ g cm⁻³**	1.42122(8)	$\mathbf{R_p}$ **(%)**	3.95
		**GOF**	1.47

Table S2 - Crystal data and Rietveld refinement information for *S*-Co (structure type C).

**Empirical formula**	$C_6H_{15}Cl_3Co_{0.5}NO$	**Radiation**	Cu Kα
**Formula weight**	253.016	**2θ / °**	3−60
**Space group**	$P3_221$	***T* / *K***	298
**Unit cell dimensions**		**B_{eq} / Å^2**	
a / Å	9.4317(6)	$CoCl_4$	12(1)
c / Å	67.271(4)	CTA^+	11(9)
***V* / Å^3**	5182.5(8)	**R_{wp} (%)**	3.87
Z	18	**R_{Bragg} (%)**	2.11
**D_x / g cm^{-3}**	1.4592(2)	**R_p (%)**	3.04
		**GOF**	1.14

Table S3 - Crystal data and Rietveld refinement information for *S*-Cd (structure type D).

**Empirical formula**	$C_6H_{15}Cd_{0.5}Cl_3NO$	**Radiation**	Cu Kα
**Formula weight**	279.756	**2θ / °**	3−60
**Space group**	*P*2	***T* / *K***	298
**Unit cell dimensions**		**B_{eq} / Å^2**	
a / Å	9.9987(3)	$CdCl_4$	5.9(3)
b / Å	9.3912(3)	CTA^+	8.0(6)
c / Å	12.5992(5)	**R_{wp} (%)**	7.84%
β / °	96.238(2)	**R_{Bragg} (%)**	2.94%
***V* / Å^3**	1176.06(7)	**R_p (%)**	5.99
Z	4	**GOF**	1.48
**D_x / g cm^{-3}**	1.5800(10)		

Table S4 - Crystal data and Rietveld refinement information for *S*-Zn (structure type E).

**Empirical formula**	$C_6H_{15}Cl_3NOZn_{0.5}$	**Radiation**	Cu Kα
**Formula weight**	256.248	**2θ / °**	3−60
**Space group**	*C*2	***T* / *K***	298
**Unit cell dimensions**		**B_{eq} / Å^2**	7.4(2)
a / Å	17.1354(6)	**R_{wp} (%)**	6.80
b / Å	9.1316(3)	**R_{Bragg} (%)**	3.38
c / Å	15.0984(6)	**R_p (%)**	5.33
β / °	102.234(2)	**GOF**	1.26
***V* / Å^3**	2308.84(15)		
Z	8		
**D_x / g cm^{-3}**	1.47439(9)		

Table S5 - Crystal data and Rietveld refinement information for *S*-Ni (structure type E).

**Empirical formula**	$C_6H_{15}Cl_3NNi_{0.5}O$	**Radiation**	Cu K*α*
**Formula weight**	252.896	***2θ* / °**	3−60
**Space group**	*C*2	***T* / *K***	298
**Unit cell dimensions**		**B_{eq} / Å^2**	6.7(3)
a / Å	17.1405(8)	**R_{wp} (%)**	7.07
b / Å	9.1339(4)	**R_{Bragg} (%)**	2.24
c / Å	15.1043(8)	**R_p (%)**	5.42
β / °	102.236(3)	**GOF**	1.35
***V* / Å^3**	2311.0(2)		
Z	8		
**D_x / g cm^{-3}**	1.45371(12)		

Table S6 - Crystal data and Rietveld refinement information for *Rac*-Cd (structure type F).

**Empirical formula**	$C_6H_{15}Cd_{0.5}Cl_3NO$	**Radiation**	Mo K*α*
**Formula weight**	279.756	***2θ* / °**	2−30
**Space group**	$Fm\bar{3}m$	***T* / *K***	437
**Unit cell dimensions**		**B_{eq} / Å^2**	
a / Å	13.56	$CdCl_4$	23
***V* / Å^3**	2496	CTA^+	10
Z	8	**R_{wp} (%)**	4.75
**D_x / g cm^{-3}**	1.489	**R_{Bragg} (%)**	1.15
		**R_p (%)**	3.77
		**GOF**	1.10

Table S7 - Crystal data and Rietveld refinement information for *S*-Cd (structure type F).

**Empirical formula**	$C_6H_{15}Cd_{0.5}Cl_3NO$	**Radiation**	Mo K*α*
**Formula weight**	279.756	***2θ* / °**	2−30
**Space group**	$Fm\bar{3}m$	***T* / *K***	449
**Unit cell dimensions**		**B_{eq} / Å^2**	25
a / Å	13.64	**R_{wp} (%)**	5.05
***V* / Å^3**	2535	**R_{Bragg} (%)**	5.73
Z	8	**R_p (%)**	3.27
**D_x / g cm^{-3}**	1.466	**GOF**	2.19

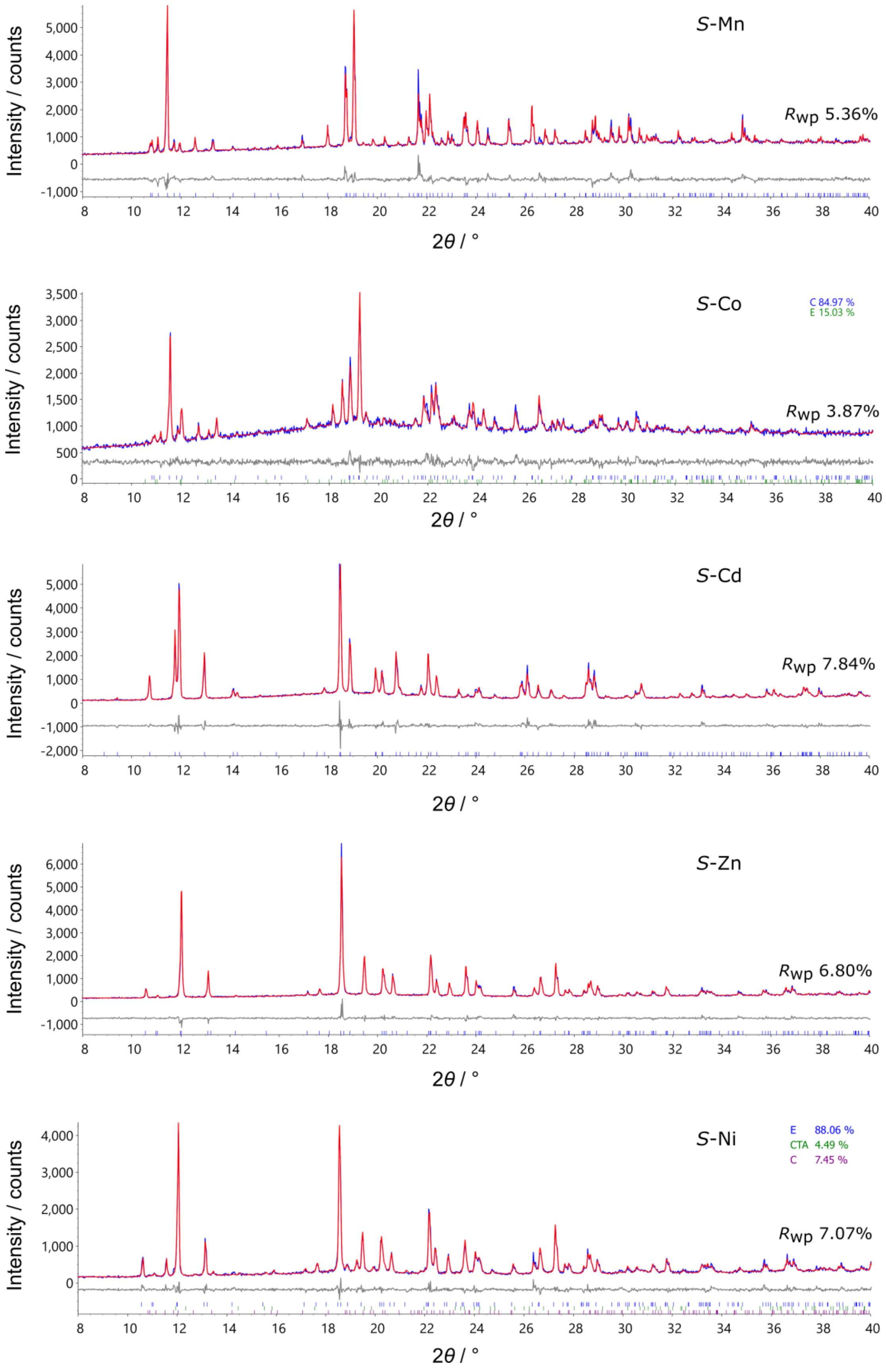

Figure S5 - Powder X-ray diffraction data (blue) of the newly discovered crystal structures with Rietveld refinement fits (red) using structural models solved from SXRD. Difference curves shown in grey. Minor peak discrepancies observed here but not in the Pawley fits of Figure S6 are likely due to texture or preferred orientation effects in the lightly-ground samples.

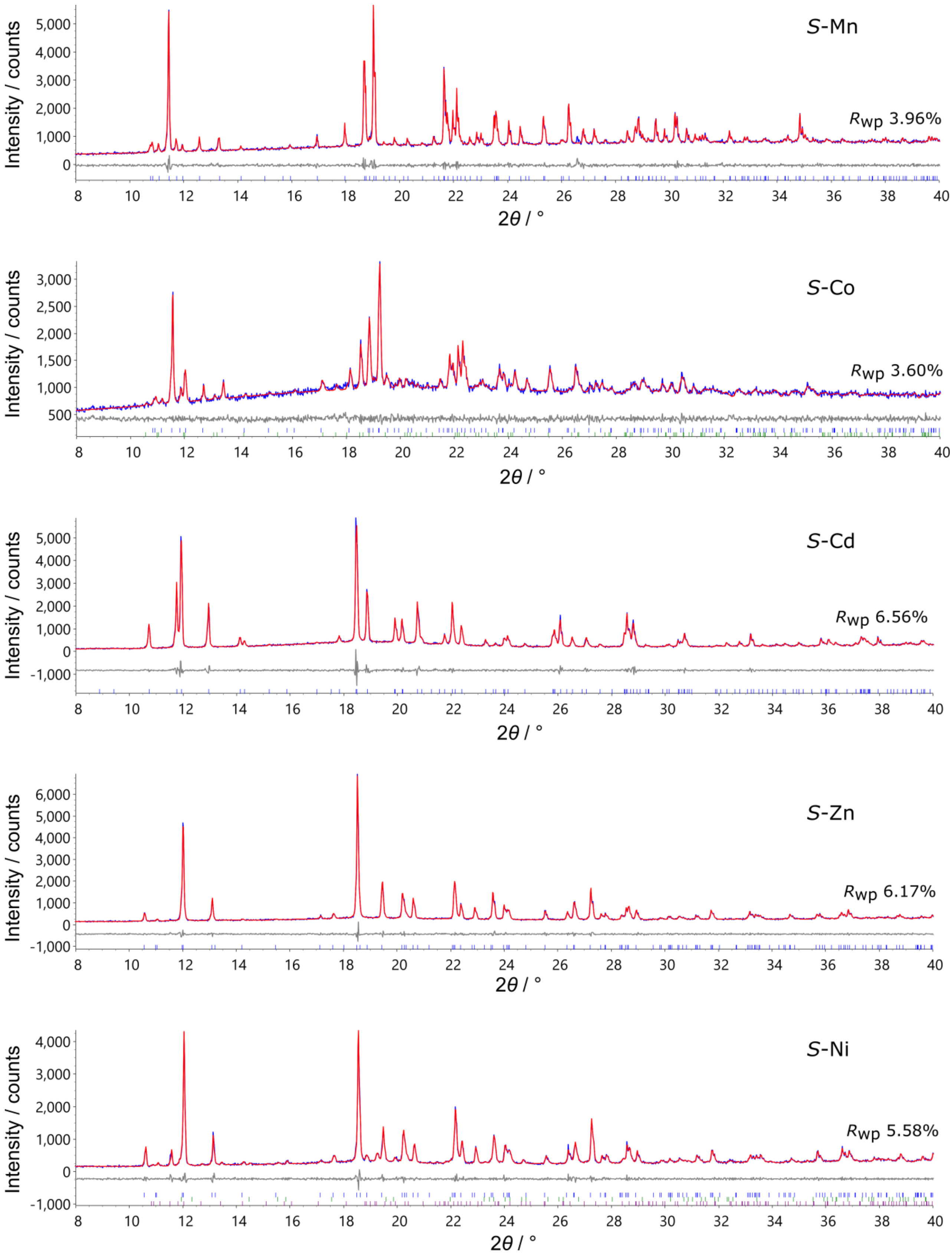

Figure S6 - Powder X-ray diffraction data (blue) of the newly discovered crystal structures with Pawley refinement fits (red) using the cells from Figure S5.

Table S8 - Crystal data and structure refinement details for *S*-Mn.

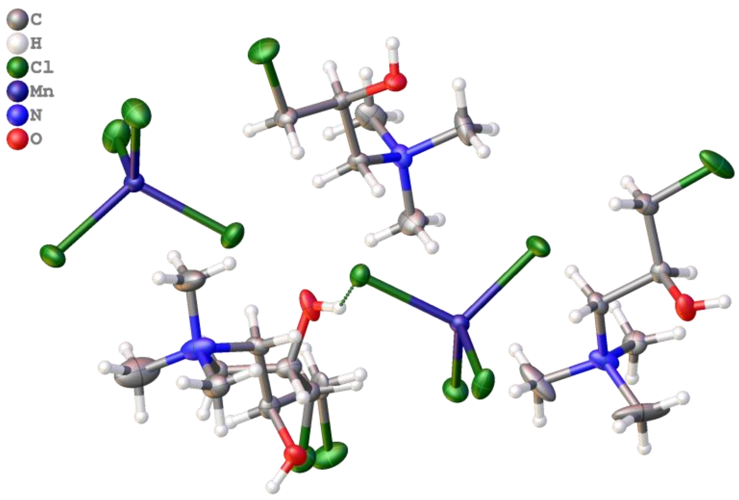

Identification code	S-Mn
Empirical formula	$C_6H_{15}Cl_3Mn_{0.5}NO$
Formula weight	251.019
Temperature/K	120
Crystal system	trigonal
Space group	$P3_221$
a/Å	9.4127(3)
b/Å	9.4127(3)
c/Å	66.730(3)
α/°	90
β/°	90
γ/°	120
Volume/Å^3	5120.1(3)
Z	18
ρ_{calc}g/cm^3	1.465
μ/mm^{-1}	1.292
F(000)	2343.0
Crystal size/mm^3	0.292 × 0.215 × 0.144
Radiation	Mo Kα (λ = 0.71073)
2Θ range for data collection/°	5 to 59.82
Index ranges	$-13 \leq h \leq 12$, $-13 \leq k \leq 13$, $-90 \leq l \leq 92$
Reflections collected	67194
Independent reflections	9596 [R_{int} = 0.0499, R_{sigma} = 0.0416]
Data/restraints/parameters	9596/6/366
Goodness-of-fit on F^2	1.046
Final R indexes [I>=2σ (I)]	R_1 = 0.0565, wR_2 = 0.0977
Final R indexes [all data]	R_1 = 0.0624, wR_2 = 0.0999
Largest diff. peak/hole / e Å^{-3}	0.97/-1.10
Flack parameter	0.011(7)

Table S9 - Fractional atomic coordinates (×10^4) and equivalent isotropic displacement parameters (Å^2×10^3) for *S*-Mn. U_{eq} is defined as 1/3 of the trace of the orthogonalised U_{IJ} tensor.

**Atom**	***x***	***y***	***z***	**U(eq)**
Mn1	-3046.4(6)	3616.4(6)	5594.93(7)	17.48(10)
Cl4	-4892.2(13)	2936.9(13)	5328.02(14)	31.3(2)
Cl5	-2985.3(13)	1258.3(12)	5696.75(14)	29.6(2)
Cl6	-3776.0(10)	4774.8(10)	5861.95(12)	22.18(16)
Cl7	-393.2(12)	5695.9(12)	5491.76(14)	31.1(2)
Mn2	-9458.8(8)	0	6666.666667	21.71(15)
Cl8	-8468.7(12)	-897.2(13)	6398.31(14)	31.8(2)
Cl9	-9591.6(16)	2346.7(13)	6569.67(17)	41.0(3)
Cl1	-4003(2)	-2177(2)	6216.1(3)	41.5(5)
Cl1A	-3524(6)	-1333(6)	6283.8(6)	37.0(11)
O1	-1873(4)	1627(5)	6226.4(5)	25.0(8)
O1A	-5122(12)	1681(11)	6115.0(12)	30(2)
N1	-3492(4)	3452(4)	6444.1(6)	37.4(8)
C1	-4699(14)	-672(13)	6176.9(17)	25(2)
C1A	-4490(30)	-510(30)	6206(4)	20(6)
C2	-3483(6)	868(6)	6307.4(7)	20.6(10)
C2A	-4049(16)	1279(15)	6219(2)	23(3)
C3	-4213(6)	1990(6)	6292.0(8)	20.0(10)
C3A	-4062(15)	1552(13)	6439.8(17)	19(2)
C4	-1852(5)	4708(5)	6370.6(6)	31.8(9)
C5	-3369(7)	3127(6)	6656.9(8)	59.1(15)
C6	-4649(6)	4101(6)	6426.5(8)	46.6(13)
Cl2	-8555.8(14)	5686.0(12)	6049.86(17)	38.0(2)
O2	-8710(3)	3511(3)	5699.4(4)	24.2(5)
N2	-9045(4)	110(3)	5798.9(4)	21.0(6)
C7	-7865(5)	4237(4)	6044.8(5)	24.7(7)
C8	-8854(4)	2865(4)	5894.6(5)	18.6(6)
C9	-8088(4)	1771(4)	5895.8(5)	18.7(6)
C10	-9382(6)	237(5)	5581.5(5)	36.7(10)
C11	-8038(6)	-701(5)	5815.1(6)	33.6(9)
C12	-10642(5)	-910(5)	5906.5(6)	33.7(9)
Cl3	-8228.4(13)	-656.3(14)	4815.46(16)	43.6(3)
O3	-5071(3)	-516(3)	4972.5(4)	27.6(6)
N3	-5676(4)	-3031(4)	5320.8(5)	26.9(7)
C13	-7747(5)	-934(5)	5065.8(5)	24.4(7)
C14	-6592(4)	-1610(4)	5066.0(5)	18.2(6)
C15	-6229(5)	-1802(4)	5283.8(5)	20.7(7)
C16	-4105(6)	-2572(7)	5213.4(10)	70(2)
C17	-6953(5)	-4716(4)	5258.2(6)	25.3(8)
C18	-5431(6)	-3068(5)	5543.1(7)	50.9(14)

Table S10 - Crystal data and structure refinement details for *S*-Co.

Identification code	S-Co
Empirical formula	$C_6H_{15}Cl_3Co_{0.5}NO$
Formula weight	253.016
Temperature/K	120.00
Crystal system	trigonal
Space group	$P3_221$
a/Å	9.3727(3)
b/Å	9.3727(3)
c/Å	66.173(3)
α/°	90
β/°	90
γ/°	120
Volume/Å^3	5034.3(3)
Z	18
ρ_{calc}g/cm^3	1.502
μ/mm^{-1}	1.491
F(000)	2361.1
Crystal size/mm^3	0.192 × 0.174 × 0.164
Radiation	Mo Kα (λ = 0.71073)
2Θ range for data collection/°	3.7 to 59.14
Index ranges	-12 ≤ h ≤ 13, -13 ≤ k ≤ 12, -95 ≤ l ≤ 92
Reflections collected	54570
Independent reflections	9188 [R_{int} = 0.0752, R_{sigma} = 0.1032]
Data/restraints/parameters	9188/382/366
Goodness-of-fit on F^2	1.052
Final R indexes [I>=2σ (I)]	R_1 = 0.0864, wR_2 = 0.1588
Final R indexes [all data]	R_1 = 0.1203, wR_2 = 0.1731
Largest diff. peak/hole / e Å^{-3}	1.18/-1.77
Flack parameter	0.018(10)

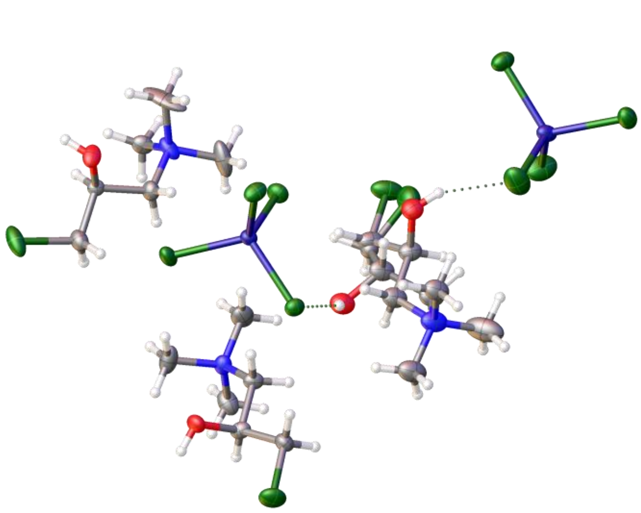

Table S11 - Fractional atomic coordinates (×10[4]) and equivalent isotropic displacement parameters (Å[2]×10[3]) for *S*-CO. U_{eq} is defined as 1/3 of the trace of the orthogonalised U_{IJ} tensor.

**Atom**	***x***	***y***	***z***	**U(eq)**
Co1	2988.2(11)	6667.5(11)	6071.04(13)	20.48(19)
Co2	-605.5(15)	-605.5(15)	5000	27.4(3)
Cl3	3704(2)	8489(2)	5811.6(3)	26.6(4)
Cl6	4765(2)	7776(2)	6331.9(3)	32.8(4)
Cl5	2932(2)	4331(2)	5971.1(3)	30.4(4)
Cl4	419(2)	6100(3)	6172.4(3)	31.5(4)
Cl9	-1546(3)	-2416(3)	5261.6(3)	36.7(5)
Cl2	8513(3)	14235(3)	5612.9(3)	42.3(5)
Cl7	8210(3)	7661(3)	6845.6(3)	46.2(6)
Cl10	-416(3)	1810(3)	5095.2(4)	55.5(7)
Cl1	3967(5)	1821(4)	5462.0(8)	49.9(11)
O00C	5059(6)	4583(8)	6694.5(7)	31.3(12)
O2	8683(7)	12222(6)	5967.5(7)	28.6(11)
N2	8965(7)	9104(7)	5870.0(8)	24.5(12)
N00F	5622(7)	2656(8)	6343.1(9)	28.8(14)
C00G	6567(8)	4994(8)	6598.1(10)	19.4(14)
N1	3452(8)	6955(9)	5219.5(10)	39.0(12)
C8	8814(9)	11699(9)	5769.2(10)	24.0(15)
C00J	6888(9)	2212(9)	6407.5(11)	28.5(16)
C00K	6194(10)	4442(9)	6378.3(10)	26.9(15)
C9	8019(9)	9831(9)	5771.1(11)	24.1(15)
C7	7804(9)	12082(9)	5618.4(11)	27.5(16)
C00N	7725(10)	6861(10)	6595.3(11)	31.1(17)
C12	9308(12)	9584(11)	6090.2(10)	40(2)
C11	10591(10)	9696(11)	5761.6(12)	37.1(19)
C4	1793(10)	6518(11)	5293.3(13)	36.5(16)
O1	1798(10)	3452(10)	5435.7(11)	33.6(17)
C10	7955(12)	7287(10)	5852.6(13)	41(2)
C1	4530(20)	3927(19)	5485(3)	35(2)
C00U	5374(13)	2360(13)	6119.8(13)	56(3)
C5	4619(11)	8774(10)	5241.1(15)	46.6(18)
C2	3463(14)	4346(14)	5355.2(18)	31.5(16)
C00Y	4035(11)	1550(13)	6453.1(19)	77(4)
C3A	4153(14)	6174(14)	5370.5(19)	35.2(15)
C2A	4030(30)	5300(40)	5443(4)	35(3)
C14	4030(30)	5580(30)	5218(4)	36(2)
C1A	4750(40)	4180(30)	5476(7)	27(6)
Cl1A	3504(13)	2123(13)	5385.1(14)	55(3)
O1A	5060(20)	6770(20)	5553(3)	39(4)
C3	3352(13)	6528(12)	5004.5(17)	69(3)

Table S12 - Crystal data and structure refinement details for *S*-Cd.

Identification code	S-Cd
Empirical formula	$C_6H_{15}Cd_{0.5}Cl_3NO$
Formula weight	279.756
Temperature/K	120.00
Crystal system	monoclinic
Space group	P2
a/Å	9.8943(6)
b/Å	9.3092(6)
c/Å	12.4610(7)
α/°	90
β/°	96.188(2)
γ/°	90
Volume/Å^3	1141.07(12)
Z	4
ρ_{calc}g/cm^3	1.628
μ/mm^{-1}	1.667
F(000)	564.4
Crystal size/mm^3	0.215 × 0.174 × 0.164
Radiation	Mo Kα (λ = 0.71073)
2Θ range for data collection/°	4.14 to 63.04
Index ranges	-14 ≤ h ≤ 14, -13 ≤ k ≤ 13, -18 ≤ l ≤ 18
Reflections collected	26185
Independent reflections	7510 [R_{int} = 0.0275, R_{sigma} = 0.0264]
Data/restraints/parameters	7510/1/229
Goodness-of-fit on F^2	1.038
Final R indexes [I>=2σ (I)]	R_1 = 0.0181, wR_2 = 0.0397
Final R indexes [all data]	R_1 = 0.0195, wR_2 = 0.0404
Largest diff. peak/hole / e Å^{-3}	0.36/-0.29
Flack parameter	-0.007(7)

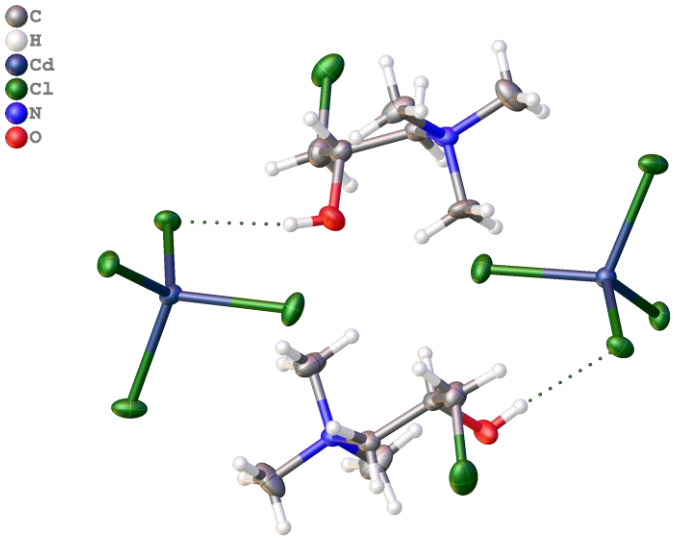

Table S13 - Fractional atomic coordinates (×10[4]) and equivalent isotropic displacement parameters ($Å^2×10^3$) for *S*-Cd. U_{eq} is defined as 1/3 of the trace of the orthogonalized U_{IJ} tensor.

Atom	*x*	*y*	*z*	U(eq)
Cd1	5000	2542.88(7)	5000	17.81(4)
Cd2	10000	7156.82(7)	0	19.37(4)
Cl4	7028.9(4)	4056.9(4)	4887.3(4)	28.1(1)
Cl6	10224.3(4)	8448.3(4)	1715.2(4)	25.64(9)
Cl3	4540.1(4)	1204.8(4)	3301.0(3)	24.47(9)
Cl1	6932.1(5)	677.8(4)	119.4(4)	40.53(12)
Cl5	7992.2(5)	5564.5(4)	-22.1(4)	30.21(11)
Cl2	7637.2(6)	9293.1(4)	5055.4(4)	37.55(11)
N1	10175.0(14)	2753.2(16)	2533.9(11)	19.3(3)
N2	4803.8(14)	6902.1(15)	2467.6(11)	19.5(3)
O1	7487.5(12)	1159.1(12)	2647.7(10)	28.8(3)
C3	9085.9(15)	2299.0(19)	1646.8(12)	19.5(3)
C10	5058(2)	5423.8(19)	2068.6(16)	29.2(4)
C2	7637.8(16)	2316.3(17)	1940.1(13)	20.5(3)
O2	7758.1(13)	6676.1(13)	2532.6(11)	33.1(3)
C1	6638.7(16)	2215.7(19)	926.1(14)	27.7(4)
C12	4828(2)	7943.8(19)	1562.4(14)	30.7(5)
C7	8180.7(18)	7926.9(19)	4184.3(15)	31.2(4)
C8	7215.6(17)	7764.3(18)	3152.5(14)	23.7(4)
C6	9966(2)	4263(2)	2884.2(19)	35.1(5)
C4	10241(2)	1780(2)	3500.8(15)	36.5(5)
C11	3418.1(18)	6933(2)	2845.3(16)	35.7(5)
C9	5817.2(15)	7300.6(19)	3424.7(12)	19.0(3)
C5	11501.5(17)	2667(3)	2070.0(18)	41.6(6)

Table S14 - Crystal data and structure refinement details for *S*-Zn.

Identification code	S-Zn
Empirical formula	$C_6H_{15}Cl_3NOZn_{0.5}$
Formula weight	256.248
Temperature/K	120.00
Crystal system	monoclinic
Space group	C2
a/Å	16.9521(4)
b/Å	9.0391(2)
c/Å	14.9881(4)
α/°	90
β/°	101.8307(8)
γ/°	90
Volume/Å^3	2247.87(9)
Z	8
ρ_{calc}g/cm^3	1.514
μ/mm^{-1}	1.813
F(000)	1061.3
Crystal size/mm^3	0.118 × 0.117 × 0.077
Radiation	Mo Kα (λ = 0.71073)
2Θ range for data collection/°	4.92 to 63.24
Index ranges	-24 ≤ h ≤ 24, -13 ≤ k ≤ 13, -22 ≤ l ≤ 22
Reflections collected	32092
Independent reflections	7503 [R_{int} = 0.0329, R_{sigma} = 0.0321]
Data/restraints/parameters	7503/1/217
Goodness-of-fit on F^2	1.021
Final R indexes [I>=2σ (I)]	R_1 = 0.0266, wR_2 = 0.0521
Final R indexes [all data]	R_1 = 0.0320, wR_2 = 0.0544
Largest diff. peak/hole / e Å^{-3}	0.33/-0.31
Flack parameter	-0.006(4)

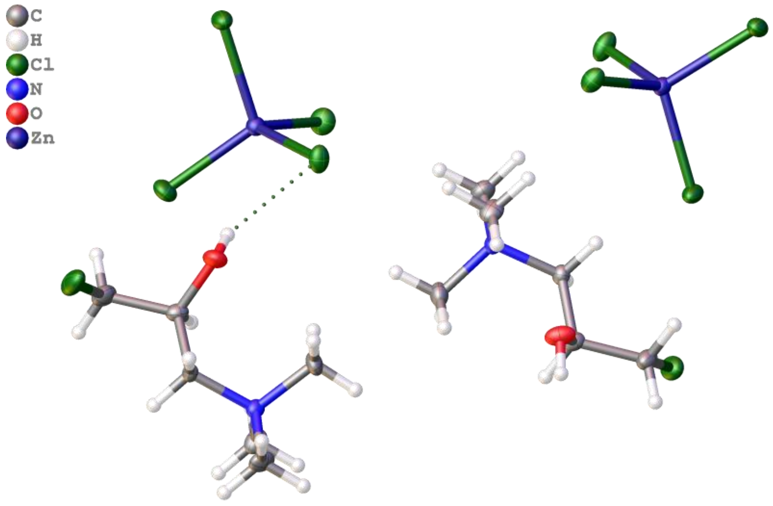

Table S15 - Fractional atomic coordinates (×10[4]) and equivalent isotropic displacement parameters (Å[2]×10[3]) for *S*-Zn. U_{eq} is defined as 1/3 of the trace of the orthogonalised U_{IJ} tensor.

**Atom**	***x***	***y***	***z***	**U(eq)**
Zn1	-10000	188.90(19)	5000	16.11(7)
Cl3	-9136.2(3)	-1329.1(5)	5962.2(3)	23.6(1)
Cl4	-10730.4(3)	1426.5(5)	5845.9(4)	26.04(11)
Zn2	-10000	-4482.84(19)	10000	15.37(7)
Cl5	-9378.3(3)	-5836.5(5)	9068.5(3)	22.6(1)
Cl6	-9063.5(3)	-3079.2(5)	10925.1(3)	25.87(11)
Cl1	-6778.6(3)	-933.1(6)	3402.5(4)	37.91(15)
O1	-7575.4(7)	-829.3(14)	5112.5(9)	24.9(3)
N1	-6082.5(9)	651.6(17)	6369.1(10)	15.7(3)
C1	-7251.4(12)	580(2)	3870.7(14)	27.6(5)
C2	-7168.7(10)	444(2)	4893.6(14)	19.0(4)
C3	-6284.1(10)	318(2)	5356.7(11)	15.9(4)
C4	-5206.5(11)	315(2)	6704.6(14)	24.7(5)
C5	-6555.7(13)	-281(2)	6903.2(14)	25.0(5)
C6	-6216.8(13)	2265(2)	6543.8(14)	22.8(4)
Cl2	-8344.1(3)	1867.9(5)	11541.8(4)	28.95(11)
O2	-7442.0(7)	-901.3(13)	9924.1(10)	25.3(3)
N2	-8891.9(9)	-63.8(17)	8626.0(11)	16.4(3)
C7	-7819.2(12)	345(2)	11165.5(13)	23.2(4)
C8	-7870.9(10)	348(2)	10137.1(13)	17.6(4)
C9	-8751.4(10)	223(2)	9649.5(12)	16.2(4)
C10	-9761.0(11)	298(2)	8238.3(14)	23.7(4)
C11	-8366.3(13)	900(2)	8180.4(14)	23.3(4)
C12	-8762.9(13)	-1662(2)	8432.2(15)	23.9(5)

Table S16 - Crystal data and structure refinement details for *S*-Ni.

Identification code	S-Ni
Empirical formula	$C_6H_{15}Cl_3NNi_{0.5}O$
Formula weight	252.896
Temperature/K	120.00
Crystal system	monoclinic
Space group	C2
a/Å	16.9483(6)
b/Å	9.0380(3)
c/Å	14.9863(5)
α/°	90
β/°	101.8272(11)
γ/°	90
Volume/Å^3	2246.85(13)
Z	8
ρ_{calc}g/cm^3	1.495
μ/mm^{-1}	1.584
F(000)	1048.0
Crystal size/mm^3	0.11 × 0.1 × 0.07
Radiation	MoKα (λ = 0.71073)
2Θ range for data collection/°	4.912 to 59.986
Index ranges	-23 ≤ h ≤ 23, -12 ≤ k ≤ 12, -21 ≤ l ≤ 21
Reflections collected	29407
Independent reflections	6542 [R_{int} = 0.0325, R_{sigma} = 0.0273]
Data/restraints/parameters	6542/1/223
Goodness-of-fit on F^2	1.049
Final R indexes [I>=2σ (I)]	R_1 = 0.0308, wR_2 = 0.0735
Final R indexes [all data]	R_1 = 0.0341, wR_2 = 0.0757
Largest diff. peak/hole / e Å^{-3}	0.71/-0.43
Flack parameter	-0.089(6)

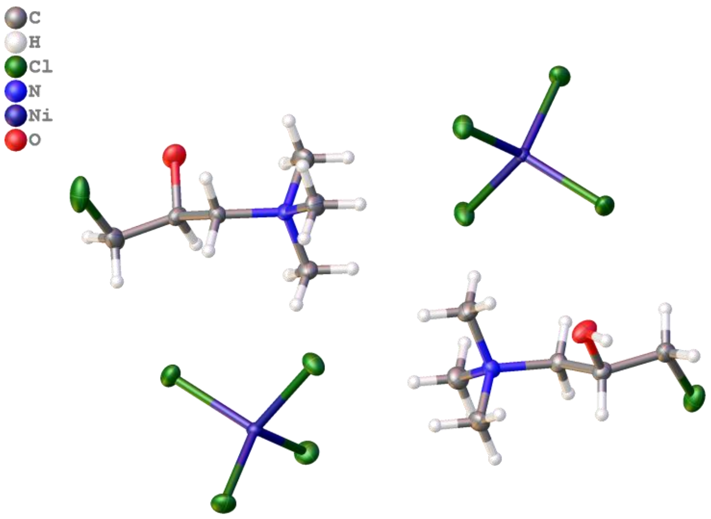

Table S17 - Fractional atomic coordinates (×10[4]) and equivalent isotropic displacement parameters (Å[2]×10[3]) for *S*-Ni. U_{eq} is defined as 1/3 of the trace of the orthogonalised U_{IJ} tensor.

**Atom**	***x***	***y***	***z***	**U(eq)**
Ni1	5000	6280.4(5)	5000	13.32(13)
Cl1	5730.3(6)	7517.3(11)	4153.6(7)	28.7(2)
Cl2	4136.0(6)	4761.7(11)	4037.6(6)	26.2(2)
Ni2	5000	1608.6(5)	0	12.67(12)
Cl3	4064.1(6)	3011.7(11)	-924.7(6)	28.8(2)
Cl4	4378.2(5)	253.5(11)	931.3(6)	25.49(19)
Cl5	3343.8(6)	7958.5(13)	-1541.1(7)	31.9(2)
O1	2440.8(15)	5190(3)	79(2)	27.8(5)
N1	3894.2(19)	6029(4)	1374(2)	19.7(6)
C1	3751(2)	6312(4)	353(2)	19.1(7)
C2	2870(2)	6451(4)	-136(3)	20.1(7)
C3	2816(2)	6430(5)	-1164(3)	25.9(8)
C4	3367(3)	6986(5)	1823(3)	26.8(8)
C5	4762(2)	6393(5)	1764(3)	27.4(8)
C6	3762(3)	4433(5)	1568(3)	27.8(9)
Cl6	6779.7(6)	159.2(17)	6598.5(7)	40.8(3)
O2	7577.9(15)	259(3)	4887.1(18)	27.8(5)
N2	6082.3(18)	1749(4)	3631(2)	18.4(6)
C7	6284(2)	1409(4)	4645(2)	18.3(7)
C8	7166(2)	1546(5)	5109(3)	21.0(7)
C9	7254(3)	1674(5)	6131(3)	31.4(9)
C10	6218(3)	3363(5)	3454(3)	25.5(8)
C11	5205(2)	1411(5)	3298(3)	27.2(8)
C12	6559(3)	807(5)	3099(3)	27.5(9)
