# Supplementary material for: Correction to “Potential Multiaxial Molecular Ferroelectricity through Chiral Cation Replacement”
Source: Cryst Growth Des. 2026 Jun 1;26(12):4778. doi: 10.1021/acs.cgd.6c00647 (PMC13281391; doi:10.1021/acs.cgd.6c00647)

checkCIF/PLATON report

Structure factors have been supplied for datablock(s) 23srv123

THIS REPORT IS FOR GUIDANCE ONLY. IF USED AS PART OF A REVIEW PROCEDURE FOR PUBLICATION, IT SHOULD NOT REPLACE THE EXPERTISE OF AN EXPERIENCED CRYSTALLOGRAPHIC REFEREE.

No syntax errors found. CIF dictionary Interpreting this report

Datablock: 23srv123

```
Bond precision:   C-C = 0.0055 A           Wavelength=0.71073

Cell:             a=16.9483(6)     b=9.0380(3)          c=14.9863(5)
                  alpha=90         beta=101.8272(11)    gamma=90
Temperature:      120 K

                  Calculated                 Reported
Volume            2246.85(13)                2246.85(13)
Space group       C 2                        C 1 2 1
Hall group        C 2y                       C 2y
Moiety formula    2(C6 H15 Cl N O), Cl4 Ni   0.5(Cl4 Ni), C6 H15 Cl N O
Sum formula       C12 H30 Cl6 N2 Ni O2       C6 H15 Cl3 N Ni0.50 O
Mr                505.77                     252.89
Dx,g cm-3         1.495                      1.495
Z                 4                          8
Mu (mm-1)         1.584                      1.584
F000              1048.0                     1048.0
F000'             1053.11
h,k,lmax          23,12,21                   23,12,21
Nref              6560[ 3471]                6542
Tmin,Tmax         0.840,0.895                0.292,0.338
Tmin'             0.840

Correction method= # Reported T Limits: Tmin=0.292 Tmax=0.338
AbsCorr = MULTI-SCAN
```

```
Data completeness= 1.88/1.00       Theta(max)= 29.993

                                                  wR2(reflections)=
R(reflections)= 0.0308( 6158)                     0.0757( 6542)

S = 1.049                  Npar= 223
```

The following ALERTS were generated. Each ALERT has the format
test-name_ALERT_alert-type_alert-level.
Click on the hyperlinks for more details of the test.

Alert level C

```
PLAT041_ALERT_1_C Calc. and Reported SumFormula    Strings  Differ     Please Check
              Calc: C12 H30 Cl6 N2 Ni O2
              Rep.: C6 H15 Cl3 N Ni0.50 O
PLAT042_ALERT_1_C Calc. and Reported MoietyFormula Strings  Differ     Please Check
              Calc: 2(C6 H15 Cl N O), Cl4 Ni
              Rep.: 0.5(Cl4 Ni), C6 H15 Cl N O
PLAT911_ALERT_3_C Missing FCF Refl Between Thmin & STh/L=    0.600          9 Report
                0  2  0,  -3  1  1,   2  2  1,  -2  0  2,   0  0  2,   4  0  2,
               -2  2  3,   1  1  3,  -4  0  4,
```

Alert level G

```
PLAT045_ALERT_1_G Calculated and Reported Z Differ by a Factor ...      0.500 Check
PLAT111_ALERT_2_G ADDSYM Detects New (Pseudo) Centre of Symmetry .         91 %Fit
PLAT112_ALERT_2_G ADDSYM Detects New (Pseudo) Symm. Elem        c          91 %Fit
PLAT113_ALERT_2_G ADDSYM Suggests Possible Pseudo/New  Space-group       C2/c Check
              Check Model Parameter Symmetry for Reflection Data Support
PLAT791_ALERT_4_G Model has Chirality at C2         (Sohncke SpGr)          S Verify
PLAT791_ALERT_4_G Model has Chirality at C8         (Sohncke SpGr)          S Verify
PLAT794_ALERT_5_G Tentative Bond Valency for Ni1       (II)      .       2.06 Info
PLAT794_ALERT_5_G Tentative Bond Valency for Ni2       (II)      .       2.04 Info
PLAT910_ALERT_3_G Missing FCF Reflection(s) Below Theta(Min)[Deg]=       2.46 Note
                0  0  1,
PLAT913_ALERT_3_G Missing # of Very Strong Reflections in FCF ....          3 Note
               -3  1  1,   4  0  2,  -2  2  3,
PLAT933_ALERT_2_G Number of HKL-OMIT Records in Embedded .res File          1 Note
               -2  0  2,
PLAT967_ALERT_5_G Note: Two-Theta Cutoff Value in Embedded .res ..       60.0 Degree
PLAT969_ALERT_5_G The 'Henn et al.' R-Factor-gap value ..........       2.789 Note
              Predicted wR2: Based on SigI**2  2.71 or SHELX Weight  7.22 Note
PLAT978_ALERT_2_G Number C-C Bonds with Positive Residual Density.          3 Info
```

---

0 **ALERT level A** = Most likely a serious problem - resolve or explain
0 **ALERT level B** = A potentially serious problem, consider carefully
3 **ALERT level C** = Check. Ensure it is not caused by an omission or oversight
14 **ALERT level G** = General information/check it is not something unexpected

3 ALERT type 1 CIF construction/syntax error, inconsistent or missing data

```
  5 ALERT type 2 Indicator that the structure model may be wrong or deficient
  3 ALERT type 3 Indicator that the structure quality may be low
  2 ALERT type 4 Improvement, methodology, query or suggestion
  4 ALERT type 5 Informative message, check
```

It is advisable to attempt to resolve as many as possible of the alerts in all categories. Often the minor alerts point to easily fixed oversights, errors and omissions in your CIF or refinement strategy, so attention to these fine details can be worthwhile. It is up to the individual to critically assess their own results and, if necessary, seek expert advice.

PLATON version of 23/04/2026; check.def file version of 30/03/2026

---

duplicate check

No duplication found

Datablock 23srv123 - ellipsoid plot

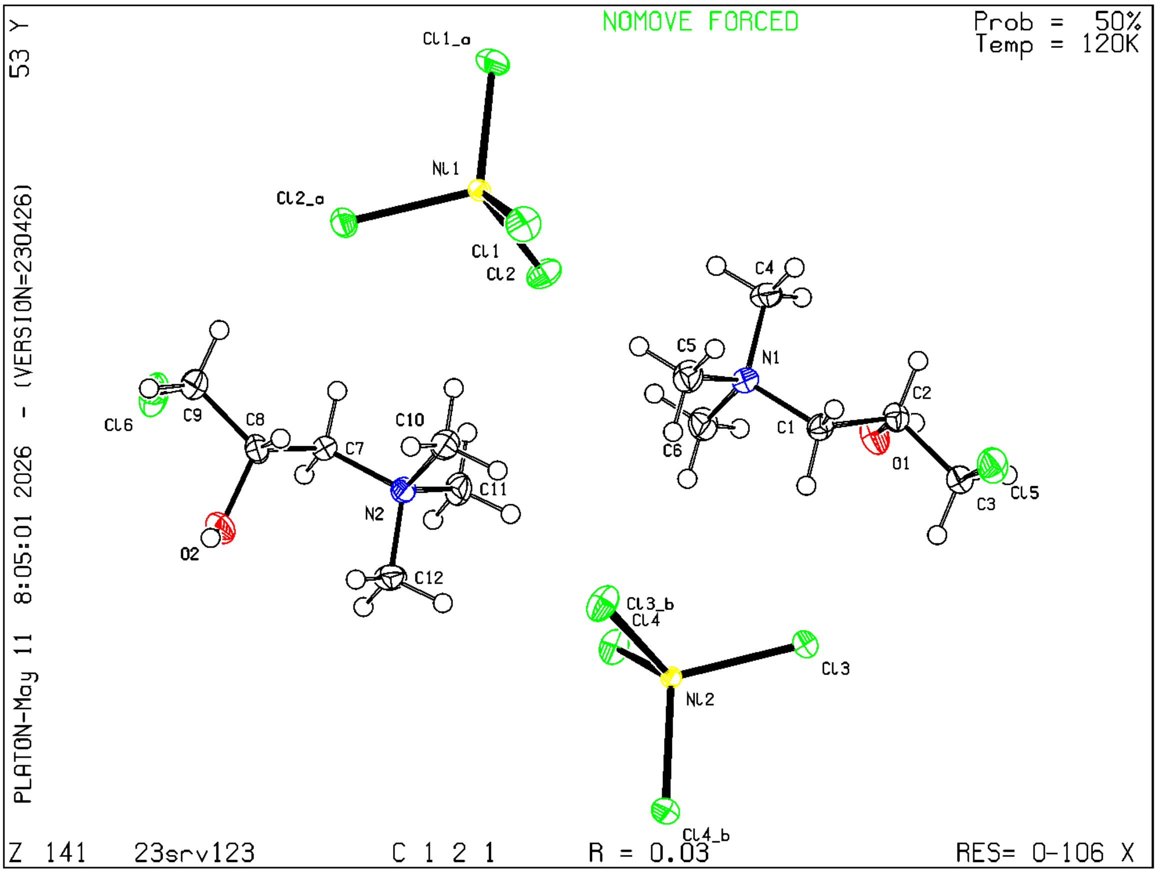

Supplement: Supplementary file 2 [file cg6c00647_si_002.zip › cg5c00666_si_002_2/SXRD/S-Ni_checkcif.pdf]
